# Evolution of the vertebrate insulin receptor substrate (*Irs*) gene family

**DOI:** 10.1186/s12862-017-0994-z

**Published:** 2017-06-23

**Authors:** Ahmad Al-Salam, David M. Irwin

**Affiliations:** 1 Department of Laboratory Medicine and Pathobiology, Faculty of Medicine, University of Toronto, 1 King’s College Circle, Toronto, ON M5S 1A8 Canada; 2 Banting and Best Diabetes Centre, University of Toronto, Toronto, ON Canada

**Keywords:** Insulin receptor substrate, Gene duplication, Protein evolution, Episodic evolution, Phylogeny, Vertebrate, Pseudogene

## Abstract

**Background:**

Insulin receptor substrate (Irs) proteins are essential for insulin signaling as they allow downstream effectors to dock with, and be activated by, the insulin receptor. A family of four Irs proteins have been identified in mice, however the gene for one of these, *IRS3*, has been pseudogenized in humans. While it is known that the *Irs* gene family originated in vertebrates, it is not known when it originated and which members are most closely related to each other. A better understanding of the evolution of *Irs* genes and proteins should provide insight into the regulation of metabolism by insulin.

**Results:**

Multiple genes for Irs proteins were identified in a wide variety of vertebrate species. Phylogenetic and genomic neighborhood analyses indicate that this gene family originated very early in vertebrae evolution. Most *Irs* genes were duplicated and retained in fish after the fish-specific genome duplication. *Irs* genes have been lost of various lineages, including *Irs3* in primates and birds and *Irs1* in most fish. Irs3 and Irs4 experienced an episode of more rapid protein sequence evolution on the ancestral mammalian lineage. Comparisons of the conservation of the proteins sequences among *Irs* paralogs show that domains involved in binding to the plasma membrane and insulin receptors are most strongly conserved, while divergence has occurred in sequences involved in interacting with downstream effector proteins.

**Conclusions:**

The *Irs* gene family originated very early in vertebrate evolution, likely through genome duplications, and in parallel with duplications of other components of the insulin signaling pathway, including insulin and the insulin receptor. While the N-terminal sequences of these proteins are conserved among the paralogs, changes in the C-terminal sequences likely allowed changes in biological function.

**Electronic supplementary material:**

The online version of this article (doi:10.1186/s12862-017-0994-z) contains supplementary material, which is available to authorized users.

## Background

The intracellular actions of insulin are initiated by the binding of the hormone insulin to its specific cell surface receptor, the insulin receptor [1, 2]. The insulin receptor is a heterotetrameric protein consisting of two extracellular alpha subunits and two transmembrane beta subunits that are connected by disulfide bridges [3, 4]. The binding of insulin to the extracellular alpha subunits of the receptor induces a conformational change that activates the intracellular tyrosine kinase domain found in the beta subunits [5, 6]. Once the tyrosine kinase activity is triggered, the insulin receptor autophosphorylates key tyrosine residues (Tyr-1158, Tyr-1162, and Tyr1163, in the human sequence) in the intracellular portion of the beta subunit [7]. Phosphorylation of these sites then allows interactions with docking proteins, which are also subsequently tyrosine phosphorylated by the insulin receptor tyrosine kinase activity [8], and downstream signaling via SH-2 domain-containing proteins to yield physiological responses [2]. Insulin can initiate several different signaling pathways that regulate metabolic responses, cell survival, growth, and differentiation [1, 2, 9].

Docking proteins are key molecules as they allow the aggregation of components of signaling cascades [7]. The first insulin receptor docking protein identified in mammalian cells was Insulin receptor substrate (Irs1) [10], with three additional docking proteins (Irs2, Irs3, and Irs4) subsequently characterized and found to share similarity in their sequences with Irs1 [11–13]. The four characterized members of the Irs protein family share similar protein architectures, with fairly well conserved N-terminal pleckstrin homology (PH) and phosphotyrosine binding (PTB) domains located near their N-termini and having relatively long C-terminal extensions [14–18]. The C-terminal extensions, which show lower levels of similarity than the N-terminal region, contain multiple tyrosine phosphorylation motifs (as well as serine/threonine phosphorylation motifs) that interact with multiple signaling proteins [14–18]. The PH and PTB domains aid in targeting Irs proteins to the plasma membrane and insulin receptor, respectively [19, 20], while differences in the tyrosine phosphorylation motifs in the C-terminal sequences of the Irs proteins allow interactions with distinct downstream signaling pathways [15, 18]. Only three of the four Irs proteins found in the mouse are functional in humans, as the *IRS3* gene sequence has been pseudogenized [21]. Intriguingly, Irs3, at only 494 amino acids in length, is less than half the size of the other three characterized Irs proteins, which are about 1200–1300 amino acids in length [10–13]. Compared to the other Irs proteins, Irs3 has a shorter C-terminal domain but retains similar-sized PH and PTB domains [12, 18]. Additional proteins containing both the PH and PTB domains have been identified (i.e., Dok4 and Dok5) that interact with the insulin receptor, however these proteins lack C-terminal extension with multiple phosphotyrosine motifs [22]. While Irs proteins were initially identified due to their interaction with the insulin receptor, they also interact, as docking proteins, with receptors for other growth factors, such as the insulin growth factor 1 receptor (IGF1R) and the insulin-related receptor (IRR), that also contain intracellular tyrosine domains [23, 24].

Irs proteins exert their unique functions through a combination of tissue-specific expression and differential binding of downstream signaling proteins [14–18, 25]. Irs1 is found in many classical targets of insulin action and is important for insulin sensitivity and embryonic and post-natal body growth [26]. Irs2 is found in an overlapping set of tissues with Irs1, however appears to have a more important role in mediating the neuronal effects of insulin [27] and the growth and survival of pancreatic beta-cells [28]. On the other hand, the function of Irs4 has been difficult to identify as genetic knockouts of this gene have little physiological effect [29]. However, when these knockouts are combined with a brain-specific Irs2 knockout, unique changes in energy regulation and glucose homeostasis are observed [30]. Irs3 is not essential for growth or glucose metabolism [31] and its expression is restricted to white adipocyte tissue in mice [12, 32] (and is absent in humans [21]), suggesting a possible, but non-essential, role for this protein in adipose tissue in rodents. In contrast to other Irs proteins, the PH domain of Irs3 has an additional role in targeting Irs3 to the nucleus, in addition to the plasma membrane, a localization necessary for Irs3 induced glucose uptake [33]. Loss of the *Irs3* gene on the human lineage indicates that the function of this gene is not essential in some mammals, and raises questions about the necessity of multiple Irs proteins.

A single Irs-like protein, named Chico, has been found in *Drosophila melanogaster* that also interacts with the *Drosophila* insulin receptor [34]. Like the mammalian Irs proteins, Chico is a large protein of about 1000 amino acids in length that contains PH and PTB domains near its N-termini and multiple phosphotyrosine motifs in its C-terminal region [34]. Only a few studies have examined the origin and evolution of the vertebrate *Irs* gene family, where it has been concluded that these genes diverged on the vertebrate lineage but these studies have reached differing conclusions concerning the relationships among the 4 Irs proteins [17, 35–37]. A number of questions remain to be answered. While it appears that the *Irs* genes duplicated and diverged from each other on the vertebrate lineage, before the mouse-human divergence, how early in vertebrate evolution this occurred is currently unknown. Did the duplications occur very early in vertebrate evolution in parallel with the duplications of other members of the insulin signaling pathway such as insulin [38] and the insulin receptor [39, 40]? *Irs3* was lost on the human (primate) lineage [21]. Was this loss a unique event, or has this gene been lost on other lineages? Have other *Irs* genes been lost on other vertebrate lineages? Which gene(s) are best conserved (i.e., potentially most essential), both in terms of retention in genomes and in conservation of their sequences within vertebrates? Why is the Irs3 protein sequence much shorter than for other Irs proteins? When did the protein become smaller? Here we show that the *Irs* genes duplicated very early in vertebrate evolution, likely at a similar time as the origin of the insulin and insulin receptor gene families [38–40] and as a consequence of the two rounds of genome duplications that occurred in the vertebrate ancestor [41, 42]. Our analyses also show that the *Irs3* has been lost on multiple independent lineages, and that the genes for other Irs proteins, including *Irs1* and *Irs2*, have occasionally been lost. The length of the Irs3 protein was reduced on the early tetrapod lineage, after divergence for fish, and was followed by a period of rapid sequence evolution in an early mammalian ancestor. Intriguingly, *Irs4* also experienced an episode of rapid evolution, in parallel with *Irs3*, early in mammalian evolution.

## Results

### Number of insulin receptor substrate (*Irs*) genes in vertebrate genomes

To estimate the number of insulin receptor substrate (*Irs*) genes in the genomes of diverse vertebrate species, we conducted *BLAST* searches [43] of 64 diverse vertebrate genomes in the *Ensembl* database [44]. As the *Ensembl* database does not include a species repressing the class Chondrichthyes (cartilaginous fish), we also searched the Elephant shark genome [45], thus the genomes of a total of 65 vertebrate species, representing all vertebrate classes, were examined. Genes were named (see Additional file 1: Table S1) based on orthology-paralogy relationships derived from sequence similarity as well as our phylogenetic and genomic location analyses described below. The numbers of species searched and the numbers of each type of *Irs* gene found in the different groups of vertebrates is summarized in Table 1. Sequences belonging to all four types of *Irs* genes were found in diverse representatives of mammals, reptiles, amphibians, lobe-finned fish, and bony fish (Table 1 and Additional file 1: Table S1). Within the bony fish, a single copy of each *Irs* gene was identified in the spotted gar (Additional file 1: Table S1), a species that diverged before the fish-specific genome duplication [46]. Among the remaining species of bony fish, all of which experienced the fish-specific genome duplication, most had two copies of the *Irs2*, *Irs3*, and *Irs4*-like genes, but only a few had *Irs1* genes (Table 1 and Additional file 1: Table S1). Of the fish species that are descendants of the fish-specific genome duplication, only two (zebrafish and cavefish/Mexican tetra) had an *Irs1*-like gene, and both of these species had only a single copy of this gene, in contrast to the duplicate copies of the other *Irs* genes found in these (and other) fish species (Additional file 1: Table S1). No *Irs3* genes were found in birds or the single representative of cartilaginous fish, although *Irs1*, *Irs2*, and *Irs4* were identified in both groups (Table 1 and Additional file 1: Table S1). A single genomic sequence encoding an incomplete *Irs*-like coding region was found in the lamprey (jawless fish), which showed some affinity to *Irs2* sequences, but its orthology could not be confidently assessed (Table 1 and Additional file 1: Table S1).

**Table 1:** Numbers of *Irs*-like genes found in diverse vertebrates in the genome and coding sequence databases

	Species^a^	*Irs1* ^b^	*Irs2* ^b^	*Irs3* ^b^	*Irs4* ^b^
Mammals	43 | 81	42 | 81 (73)	39 | 73 (28)	31 | 38 (29)	42 | 78 (51)
Birds	5 | 54	5 | 51 (4)	5 | 32 (5)	0 | 0 (0)	5 | 54 (4)
Reptiles	2 | 5	2 | 5 (4)	2 | 5 (1)	2 | 5 (4)	2 | 5 (4)
Amphibians	1 | 2	1 | 2 (1)	1 | 1 (1)	1 | 1 (1)	1 | 2 (2)
Lobe-finned fish	1 | 1	1 | 1 (1)	1 | 1 (1)	1 | 1 (1)	1 | 1 (0)
Bony fish	11 | 25	3 | 5 (5)	21 | 44 (43)	21 | 49 (46)	21 | 47 (29)
Cartilaginous fish	1 | 1	1 | 1 (1)	1 | 1 (1)	0 | 0 (0)	1 | 1 (1)
Jawless fish	1 | 0	0 | 0 (0)	1 | 0 (0)	0 | 0 (0)	0 | 0 (0)
Total	65 | 167	55 | 146 (89)	71 | 157 (80)	56 | 94 (81)	73 | 188 (91)

^a^Number of species with identified genes (or searched if no genes were found): Number of species with genome sequences searched | Number of species searched only for coding sequences

^b^Number of unique genes or coding sequences found for each gene: Number of genomic sequence | Number of coding sequences (Number of full-length or near full-length sequences)

Many of the *Irs* genes identified in our searches of the *Ensembl* database were incomplete (i.e., did not predict complete open reading frames). Some of the incomplete coding sequences contained unsequenced gaps (Ns) in the genome assemblies, while others could have been due to sequencing errors or pseudogenization. To complement the sequences identified from the *Ensembl* database, a *BLAST* search [42] was conducted of the *NCBI* database [47] to identify *Irs* coding sequences (Table 1 and Additional file 2: Table S2). Searches of the *NCBI* database identified a larger number (167) of vertebrate species with *Irs* coding sequences than the *Ensembl* database, but many of these are from species do not contain near complete genome sequences (e.g., *Xenopus laevis*), thus the full complement of *Irs* genes in these species might not have been found. A second limitation of our *NCBI* searches was that we only identified *Irs*-like sequences that had been annotated as coding sequences (i.e., if the gene was not annotated or was a pseudogene it would not be found) (see Additional file 1: Table S1 and Additional file 2: Table S2). The total number of vertebrate species with identified *Irs*-like genes was 172 (59 in both *Ensembl* and *NCBI*, 1 in both the Elephant Shark Genome project and *NCBI*, 107 only in *NCBI*, and 5 only in *Ensembl*, see Table 1 and Additional file 1: Table S1 and Additional file 2: Table S2). The distribution of the *Irs*-like gene paralogs among vertebrate classes identified in the *NCBI* database was similar to that seen with the *Ensembl* database (Table 1 and Additional file 2: Table S2).

### Phylogeny of vertebrate insulin receptor substrate (*Irs*) genes

To better establish the orthology-paralogy relationships among the identified *Irs* genes, and determine when duplications of the *Irs* genes occurred, phylogenetic relationships of the sequences were established using maximum likelihood [48, 49] and Bayesian approaches [50, 51]. A total of 341 full-length, or near-full length (those missing only short portions of sequence at the N- or C-termini of their predicted proteins), *Irs*-like coding sequences from 172 vertebrate species (including 89 *Irs1*, 80 *Irs2*, 81 *Irs3*, and 91 *Irs4* sequences (Table 1 and Additional file 1: Table S1, Additional file 2: Table S2 and Additional file 3: Figure S1) were used in this analysis. Maximum likelihood phylogenetic analysis of putative *Irs* orthologs yielded topologies consistent with the expected species topologies (Additional file 4: Figure S2, Additional file 5: Figure S3, Additional file 6: Figure S4 and Additional file 7: Figure S5; similar results were obtained using Bayesian methods, results not shown), suggesting that the analyzed genes were orthologous.

**Fig. 1:**
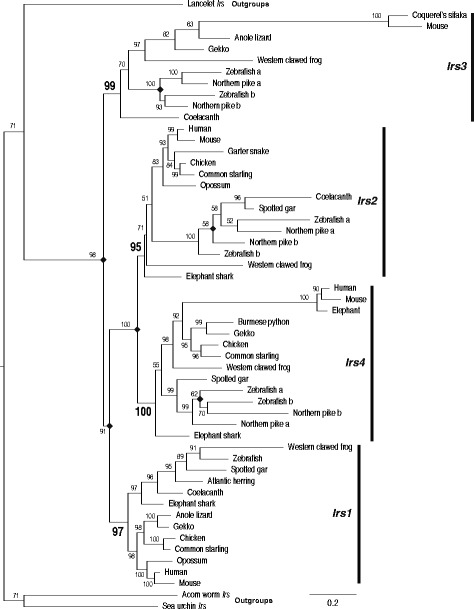
Phylogeny of vertebrate Insulin receptor substrate (*Irs*) gene family sequences. Phylogeny of *Irs* sequences from diverse vertebrate species generated by Maximum likelihood using *IQ-tree* [49]. A similar phylogeny was generated by Bayesian methods [50, 51] (see Additional file 8: Figure S6). Phylogeny was rooted with sequences from acorn worm, purple sea urchin, and Florida lancelet (labeled as outgroups). Selected *Irs* sequences were chosen to represent the diversity of vertebrates, with different vertebrate *Irs* genes identified on the right. Similar results were obtained if other *Irs* sequences were used. Numbers at the nodes bootstrap support. Branch lengths are proportional to the inferred amount of change, with the scale bar at the bottom right. Diamonds indicate gene duplication events

The relationship among the *Irs* paralogs was established using these full-length *Irs* sequences and rooted using *Irs*-like sequences from three non-vertebrate outgroup species (see Additional file 2: Table S2). Both the Maximum liklihood (Fig. 1) and Bayesian (Additional file 8: Figure S6) analyses demonstrated that each of the four *Irs* ortholog groups form strongly supported monophyletic clades that diverged from each other prior to the divergence of jawed and jawless vertebrates. Both analyses displayed the same relationships among the paralogs, with *Irs2* and *Irs4* being most closely related, then both grouping with *Irs1*, and *Irs3* genes being the most distantly related group (Fig. 1 and Additional file 8: Figure S6). Like our finding of distinct Irs paralogs in most classes of vertebrates (see Table 1), these results indicate that the *Irs* gene family originated early in vertebrate evolution. An intriguing feature of both analyses was that the mammalian *Irs3* and *Irs4* sequences have longer ancestral mammalian lineages, suggesting episodes of more rapid sequence evolution for these genes in the early mammal.

### Origin of vertebrate insulin receptor substrate (*Irs*) genes

**Fig. 2:**
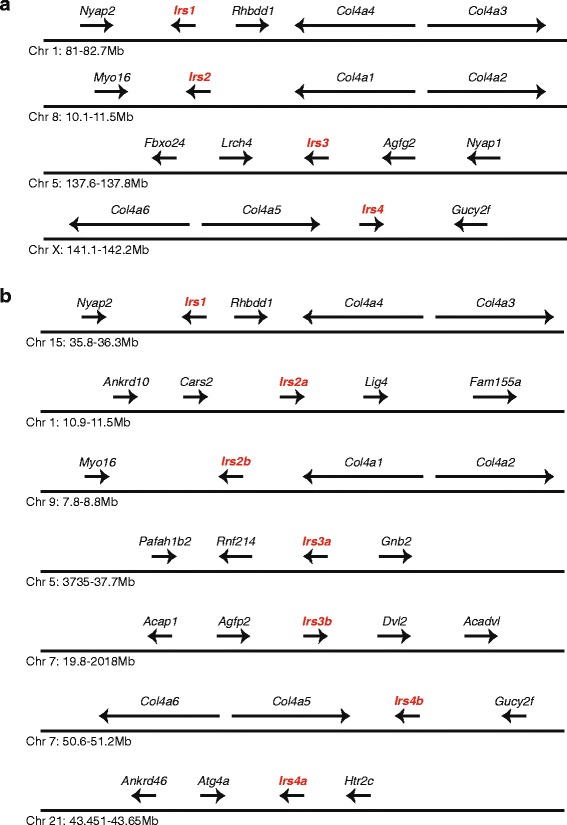
Genomic organization of genes near *Irs* genes in the mouse and zebrafish genomes. The relative organization and orientation of genes near insulin receptor substrate (*Irs*) genes in (**a**) mouse and (**b**) zebrafish. Chromosomes and genomic locations are from Ensembl [44] (see Additional file 1: Table S1). *Irs* genes are labeled in red. Gene sizes and distances between genes are not to scale. Arrowheads indicate direction of transcription. Gene symbols are: *Irs1–4*, insulin receptors substrates 1–4; *Col4a1–6*, collagen, type IV, alpha1–6; *Rhbdd1*, rhomboid domain containing 1; *Nyap2*, Neuronal tyrosine-phophorylated phosphoinositide 3-kinase adaptor 2; *Myo16*, Myosin XVI; *Fbxo24*, F-box protein 24; *Lrch4*, Leucine-rich repeats and calponin homology (CH) domain containing 4; *Agfp2*, ArfGAP with FG repeats 2; *Nyap1*, Neuronal tyrosine-phosphorylated phosphoinositide 3-kinase adaptor 1; *Gucy2f*, Guanylate cyclase 2f; *Ankrd10*, Ankyrin repeat domain 10; *Ankrd46*, Ankyrin repeat domain 46; *Cars2*, Cysteinyl-tRNA synthetase 2; *Lig4*, Ligase IV, DNA, ATP-dependent; *Fam155a*, Family with sequence similarity 155, member A; *Pafah1b2*, Platelet-activating factor acetylhydrolase, isoform 1b, subunit 2; *Rnf214*, Ring finger protein 214; *Gnb2*, Guanine nucleotide binding protein (G protein), beta 2; *Acap1*, ArfGAP with coiled-coil, ankyrin repeat and PH domains 1; *Dvl2*, Dishevelled segment polarity protein 2; *Acadvl*, Acyl-Coenzyme A dehydrogenase, very long chain; *Atg4a*, Autophagy related 4A, cysteine peptidase; *Htr2c*, 5-hydroxytryptamine (serotonin) receptor 2C

Our phylogenetic analysis indicates that the *Irs* gene family originated early in vertebrate evolution; however, due to the absence of full-length gene sequences from the lamprey, we were unable to show whether any of the duplications preceded the earliest divergence within this group. Genome duplications occurred prior to the divergence of jawed and jawless vertebrates and explain the presence of multiple gene families in vertebrate genomes [41, 42, 52]. With genome duplications, paralogous genome segments are created where different chromosome share sets of paralogous genes [41, 52]. To determine whether the *Irs* genes originated through genome duplications we examined the genomic neighborhoods surrounding the four mouse *Irs* genes. As shown in Fig. 2a, the mouse *Irs1*, *Irs2*, and *Irs4* genes are each found adjacent to a pair of collagen type IV genes (*Col4a4* and *Col4a23*, *Col4a1* and *Col4a2*, and *Col4a5* and *Col4a6*, respectively) on different chromosomes. The same arrangement was found for the human *IRS1*, *IRS2*, and *IRS4* genes (results not shown). The *Irs3* gene, on the other hand, is not located near any collagen gene (Fig. 2a). Whether this difference seen in the genomic neighborhood for *Irs3* reflects changes in genomic organization after genomic duplications, or whether *Irs3* originated via a different mechanism cannot be determined at this time. However, these results do suggest that the *Irs1*, *Irs2*, and *Irs4* genes originated via genome duplications in an early vertebrate, and as *Irs3* diverged earlier from the other *Irs* genes, this supports origin of this gene family at or before the genome duplications on the early vertebrate lineage.

### Duplication of *Irs* genes in Bony fish

Duplicate copies of *Irs2*, *Irs3*, and *Irs4* were found in most species of bony fish examined (Table 1 and Additional file 1: Table S1 and Additional file 2: Table S2). Bony fish experienced an additional genome duplication not shared by other vertebrates [53, 54], thus duplicated *Irs* genes would be expected. Duplicated *Irs* genes were not found in the genome of the spotted gar, a species that diverged from other bony fish prior to the fish-specific genome duplication [46]. Phylogenetic analysis of the *Irs2*, *Irs3*, and *Irs4* sequences (Additional file 5: Figure S3, Additional file 6: Figure S4 and Additional file 7: Figure S5) demonstrated that the duplications of these genes occurred early in bony fish evolution consistent with the fish-specific genome duplication. When the genomic neighborhoods surrounding the zebrafish *Irs* genes were examined, only one of the fish duplicates (*Irs1*, *Irs2b*, *Irs3b*, and *Irs4b*) was located in a genomic neighborhood orthologous to those seen in mice (Fig. 2b), while the second paralogous gene (*Irs2a*, *Irs3a*, and *Irs4a*) resided in genomic regions with no similarity in gene composition to the genomic region found in mice.

### Loss of the *Irs3* Gene on the primate lineage

While mice have 4 *Irs* genes, only 3 functional *Irs* genes are found in humans, as *Irs3* contains mutations that introduce a stop codon and delete part of the coding sequence [21]. Genomic sequences similar to *Irs3* were identified in a number of primate genomes in the *Ensembl* database; however, intact coding sequences could only be predicted for the Tree shrew and the Mouse lemur (Additional file 1: Table S1). Similarly, searches of the *NCBI* database for coding sequences similar to *Irs3* only identified potentially functional *Irs3* coding sequences in three primate species, the Mouse lemur, Coquerel’s sifaka, and Sunda flying lemur (Additional file 2: Table S2). Complete coding sequences could be predicted for the Mouse lemur and Coquerel’s sifaka but the sequences from the other two primates contained unsequenced gaps. Importantly, all four of these species with potentially intact *Irs3* gene sequences are early branching lineages within primates [55]. Alignment of the *Irs3* genomic sequences from diverse primates (see Additional file 9: Figure S7) using *MultiPipMaker* [56, 57] demonstrated that the sequences were not well conserved as a large number of frameshift mutations were identified along with large deletions, including those previously identified in the human *IRS3* pseudogene sequence [21]. These results suggest that *Irs3* was inactivated early in primate evolution, but after divergence of the Mouse lemur and Coquerel’s sifaka. When *MultiPipMaker* alignments were generated using the human sequence as the master sequence (results not shown), an *Alu* repetitive element that disrupts the human *IRS3* coding region [21] was found to be shared by *Irs3* sequences from primates that lack an intact coding sequence, suggesting that the insertion of this element into the gene occurred at about the same time as the pseudogenization of the gene.

### Loss of the *Irs3* Gene in birds

In addition to the absence of *Irs1* in most bony fish and *Irs3* in most primates, another notable group of animals that lack a specific *Irs* gene is birds, where no *Irs3* coding or gene sequences were identified (Table 1 and Additional file 1: Table S1 and Additional file 2: Table S2). In contrast to primates, where genomic sequences similar to *Irs3* were found containing mutations that disrupt the coding sequences (see above), genomic sequences similar to *Irs3* were not found in any of the bird genomes examined (Additional file 1: Tables S1). To exclude the possibility that the avian *Irs3* sequences had rapidly evolved, and thus were not detectable in the *BLAST* searches [38], we attempted to use genomic neighborhoods to identify these genes. However, searches for the genes that flank the mammalian *Irs3* gene (i.e., *Lrch4* and *Agfg2*, see Fig. 2) also failed to find orthologs of these genes (results not shown). These results suggest that the *Irs3* genomic region, including adjacent genes, had been deleted from the genomes of birds.

### Episodic evolution of vertebrate insulin receptor substrate (*Irs*) genes

Visual inspection of the phylogenies generated from the *Irs* coding sequences, using both single gene (Additional file 4: Figure S2, Additional file 5: Figure S3, Additional file 6: Figure S4 and Additional file 7: Figure S5) and gene family (Fig. 1 and Additional file 8: Figure S6) phylogenies, suggested accelerated evolution on the mammalian ancestral lineages for *Irs3* and *Irs4*. Branch lengths displayed in our phylogenetic analysis are proportional to the number of inferred nucleotide substitutions. For both *Irs3* and *Irs4*, mammals have accumulated more changes than sequences from the other vertebrate classes, suggesting that these genes experienced accelerated evolution early in mammalian evolution. To determine whether the longer branches are due to increased numbers of amino acid substitutions in the Irs3 and Irs4 protein sequences we conducted relative rate tests [58] with protein sequences encoded by *Irs* genes from four different mammalian species (if available) and 6 non-mammalian species (Additional file 10: Table S3). For all relative rate comparisons, the mammalian Irs3 and Irs4 protein sequences accumulated significantly higher numbers of amino acid substitutions compared to protein sequences from a diverse array of non-mammalian species. In contrast, only a small number of the comparisons with Irs1 displayed significantly higher numbers of amino acid substitution on the mammalian lineage, with none being significantly higher on the mammalian lineage for Irs2, although there were a few cases of significantly higher numbers on the non-mammalian lineage for this protein (Additional file 10: Table S3). These results show that the proteins encoded by *Irs3* and *Irs4*, but not *Irs1* or *Irs2*, have accumulated increased numbers of amino acid substitutions on the mammalian lineage.

### Changes in the lengths of vertebrate insulin receptor substrate (Irs) proteins

We then examined whether changes in the rate of amino acid sequence evolution resulted in changes in the structure of the Irs proteins. Previously, it had been reported that mouse Irs3 is much shorter than any other mouse Irs proteins, or Irs1, Irs2, and Irs4 proteins from other species [10–13]. To determine whether this was a general feature of Irs3 proteins or was specific to a subgroup of species we calculated the lengths of Irs proteins from species representing diverse groups of vertebrates (Table 2). Lengths of Irs proteins from species not listed in this table were generally similar to their most closely related representative shown in the table (results not shown). Most Irs protein sequences have a length of about 1000–1300 amino acids, except Irs3 from tetrapods (amphibians, reptiles, and mammals, Table 2). Irs3 proteins from zebrafish and coelacanth (and other fish) have length similar to those of the other Irs proteins. These observations suggest that the length of Irs3 progressively shortened from a full-length sequence of 1000–1300 amino acids, which was retained in fish, to one of ~800 residues that is found today in amphibians (*Xenopus*), to ~600 residues and found in reptiles (garter snake), to ~500 residues found in mammals (mouse) (Table 2). Most of the reduction in Irs3 protein length occurred on the lineages leading to tetrapods (ancestor of amphibians and mammals) and amniotes (ancestor of reptiles and mammals), and not on the lineage leading to mammals. This suggests that the reduction in the length of Irs3 is not associated with the accelerated protein sequence evolution observed for this sequence in the early mammalian lineage. Irs4, which also experienced increased rates of amino acid sequence evolution on the lineage leading to mammals, does not show any major changes in protein length among vertebrate classes, nor do Irs1 or Irs2 (Table 2).

**Table 2:** Lengths of Irs proteins from representative vertebrate speceis

Protein	Human	Mouse	Snake^a^	Chicken	*Xenopus*	Coelacanth	Gar^a^	Zebrafish a	Zebrafish b	Shark^b^
Irs1	1242	1231	1186	1178	1091	1076	>1085^c^	1099	NA^b^	1099
Irs2	1338	1321	1105	1148	1006	1069	1069	1032	1062	>1082
Irs3	NA^b^	495	662	NA^b^	809	1034	NA^b^	1181	1245	NA^b^
Irs4	1257	1216	1191	1164	1077	NA^b^	1120	1158	1051	1200

^a^Snake is garter snake; Gar is spotted gar; Shark is elephant shark

^b^NA, not applicable, gene was not found, incomplete, or absent

^c^Spotted gar Irs1 is missing part of its C-terminus; Elephant shark Irs2 is missing part of its N-terminus

### Conservation of Irs protein sequences

**Fig. 3:**
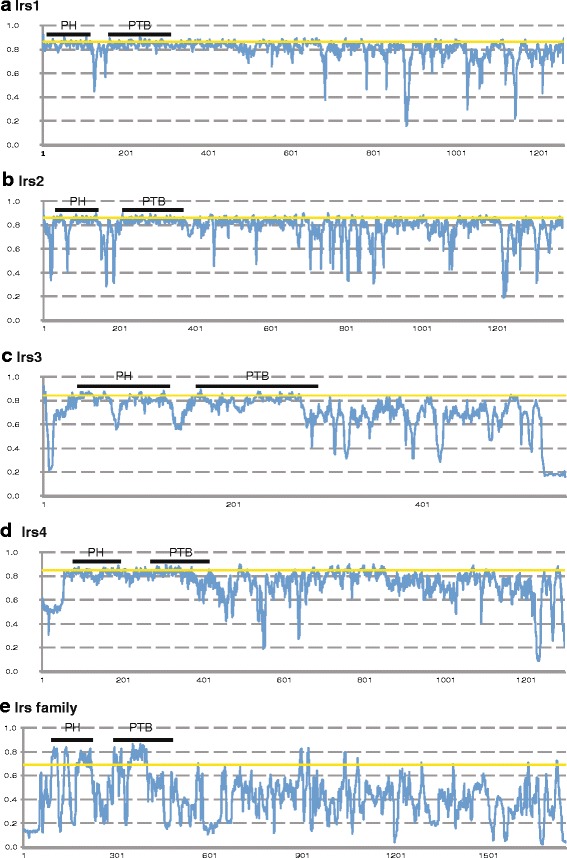
Conservation of Irs protein sequences. JS divergence scores for aligned Irs protein sequences from 10 vertebrate species. (**a**) Irs1, (**b**) Irs2, (**c**) Irs3, (**d**) Irs4, (**e**) all Irs family members. JS scores are presented in Additional file 11: Table S4. Position in alignment is shown at the bottom of each graph. The locations of the PH and PTB are shown as bars near the top of each graph. JS scores above the *yellow horizontal line* are in the top 10% of JS scores for that alignment

Since each of the Irs proteins have differing roles in insulin signaling [14–18, 25], we examined whether these roles generated differences in the constraints acting across the Irs protein sequences. To avoid lineage-specific effects, we only examined Irs protein sequences from species where full-length sequences for all four *Irs* genes had been identified (see Additional file 1: Table S1 and Additional file 2: Tables S2). As such, the four genes would then have existed in parallel in the same genomes for their entire evolutionary history and therefore have likely experienced similar evolutionary pressures at the genomic level. A total of 10 species, 9 mammals and one amphibian (mouse, rat, golden hamster, prairie vole, prairie deer mouse, Coquerel’s sifaka, mouse lemur, Mouflon sheep, killer whale, and *Xenopus tropicalis*), were found to have complete coding sequences for all 4 Irs proteins (Additional file 1: Table S1 and Additional file 2: Tables S2). If we included sequences from zebrafish, where the single *Irs1* sequence was used and one of the two paralogs for *Irs2*, *Irs3*, and *Irs4* were selected we obtained similar results for the following analyses (results not shown). Conservation of sequences was assessed using Jenson-Shannon Divergence (JS) scores [59] (Additional file 11: Table S4) and was plotted for each Irs protein and the complete set of four Irs proteins in Fig. 3. Irs1 has the highest average conservations score (0.75) followed by Irs2 (0.73), Irs4 (0.67), and Irs3 (0.45). These results suggest that the episodes of more rapid sequence evolution seen on the early mammalian lineages for Irs3 and Irs4 (see above) resulted in an acceleration relative to all Irs proteins, and that the non-mammalian Irs3 and Irs4 sequences might be evolving at rates similar to those for Irs1 and Irs2. As expected, the PH and PTB domains show strong conservation in all 4 Irs proteins, with the protein sequences between and flanking these domains generally showing lower levels of conservation (Fig. 3 and Additional file 11: Table S4). Strong conservation of the PH and PTB domains might be expected as all Irs proteins interact with plasma membranes and insulin receptors, the functions of these domains [19, 20]. Many locations in the C-terminal extensions of the 4 Irs proteins also display high levels of conservation, however it appears that Irs3, and to some extent Irs4, have lower levels than the other two proteins. In comparisons of all 4 Irs proteins, 9 short regions show high levels of conservation, as indicated by having JS score in the top 10% (Fig. 3e). This suggests that only limited parts of the C-terminal region have functions that are conserved across all *Irs* gene family members, while regions that are not conserved across all family members, but conserved within orthologs might have ortholog-specific functions.

### Tyrosine phosphorylation of Irs protein sequences

Phosphorylation of tyrosine residues in Irs proteins, especially those in the C-terminal extension, is important for signaling [2, 8, 9]. Potential tyrosine phosphorylation sites were predicted [60] for the Irs protein sequences from the 10 vertebrates that had complete sequences for all 4 family members (Table 3). Approximately 60% of the tyrosine residues in any Irs protein sequence were predicted to be phosphorylation sites. As expected, being the shortest Irs protein, Irs3 has least number of tyrosine residues (average 17.2 residues per sequence) and putative tyrosine phosphorylation sites (average of 10.1), compared to the other Irs proteins (Irs1: 33.8 and 20.4, Irs2: 36.6 and 20.1, Irs4: 31.2 and 15.8, tyrosine and putative tyrosine phosphorylation sites, respectively). Irs3 also showed the lowest conservation of tyrosine residues (10/17.2 = 58%) and putative tyrosine phosphorylation sites (5/10.1 = 50%) in protein alignments. Lower levels of conservation were also seen for Irs4 (tyrosine residues: 17/ 31.2 = 54%, and tyrosine phosphorylation sites: 9/15.8 = 57%). In contrast, conservation of both tyrosine residues (Irs1: 27/33.8 = 80%, Irs2: 25/36.6 = 68%) and putative tyrosine phosphorylation sites (Irs1: 16/20.4 = 78%, and Irs2: 13/20.1 = 65%) were higher for Irs1 and Irs2 (see Table 3 and Additional file 12: Figure S8). Only 4 tyrosine residues were conserved across all Irs sequences in all 10 species (see Additional file 12: Figure S8), with two of these being predicted tyrosine phosphorylation sites for all sequences. Of these four conserved residues, the two sites that are not conserved putative tyrosine phosphorylation sites are located in the PH domain, while the two putative tyrosine phosphorylation sites that are conserved are in the C-terminal extension (residues 608 and 628, 649 and 671, 350 and 361, and 672 and 689 in mouse Irs1, Irs2, Irs3, and Irs4, respectively) (see Additional file 12: Figure S8). The two putative tyrosine phosphorylation sites located in the C-terminal extension are located in regions that have strong conservation among the four Irs protein sequences (Additional file 12: Figure S8).

**Table 3:** Tyrosine phosphoryation of Irs proteins

	Irs1		Irs2		Irs3		Irs4	
Species	Y^a^	pY^b^	Y	pY	Y	pY	Y	pY
Mouse	34	20	36	21	17	10	29	13
Rat	35	22	36	21	18	13	30	17
Golden hamster	34	20	37	20	14	8	31	15
Prairie vole	34	21	37	20	17	11	34	13
Prarie deer mouse	34	20	37	20	18	12	27	15
Coqurel’s sifka	34	20	38	21	16	7	33	15
Mouse lemur	34	20	38	20	16	8	31	16
Mouflon sheep	34	19	38	20	14	8	30	15
Killer whale	33	20	38	21	17	8	28	15
*Xenopus tropicalis*	32	22	31	17	25	16	39	24
Average	33.8	20.4	36.6	20.1	17.2	10.1	31.2	15.8
Conserved^c^	27	16	25	13	10	5	17	9

^a^Number of tyrosine residues in the sequence

^b^Number of tyrosine residues predicted to be phosphorylated

^c^Number of residues conserved across the 10 sequences

## Discussion

### Origin of the *Irs* gene family

While multiple *Irs*-like genes have been previously characterized in several mammalian species [10–13, 17, 36], only a few non-mammalian *Irs*-like genes have been identified, which limited the ability to resolve when this gene family originated and how the different genes are related to each other [17, 35–37]. Here, our searches have identified a large number of *Irs*-like genes from a diverse array of vertebrate classes, which should allow better estimation of the time when this gene family originated and how the different genes are related to each other. Searches of vertebrate genomes identified multiple *Irs*-like sequences in the genomes of representative species for all vertebrate classes except Agnatha (Jawless fish) (Table 1 and Additional file 1: Table S1 and Additional file 2: Tables S2). However, given the low coverage of the sea lamprey somatic genome [61] and the loss of DNA in this species due to genomic remodeling in somatic tissue [62], *Irs*-like sequences may have been missed in this jawless fish. These observations suggest that the *Irs* gene family originated early in vertebrate evolution, and possibly before the earliest divergence of extant vertebrate species.

Phylogenetic analyses of the sequences (Fig. 1 and Additional file 8: Figure S6) strengthened this conclusion, demonstrating that the multiple genes originated early in vertebrate evolution and were not due to parallel duplications on diverse lineages. Analysis of genomic neighborhoods is a powerful tool for identifying orthologs [63], especially in gene families where multiple sequences have similar levels of similarity to a putative ortholog, where only the true othologs share genomic neighborhoods [64]. In this context, we used genomic neighborhoods to confirm the orthology of many of the diverse *Irs* genes found in vertebrates. When the genomic locations of *Irs*-like genes were examined (Fig. 2), three of the 4 *Irs* genes were found to be in genomic neighborhoods that shared similar gene contents. The sharing of paralogus genes among genomic neighborhoods is consistent with these genes originating through genome duplications [65], which suggests that at least 3 of the 4 *Irs* genes originated via the two rounds of genome duplication that occurred in the common ancestral vertebrate lineage [41, 42]. Interestingly, both the insulin [38] and the insulin receptor [39, 40] gene families originated very early in vertebrate evolution, and potentially via the same genome duplications. Irs proteins not only interact with the insulin receptor, but also with other receptors, including the Insulin growth factor I (IGF-1) receptor and the Insulin-related receptor (Irr) [23, 24]. These observations suggest that duplications of the genes for the ligands, receptors, and docking proteins could lead to increased specialization in these signaling pathways, and the possibility to evolve new functions.

### Change in number of *Irs* genes

While *Irs* gene originated very early in vertebrate evolution, the number of *Irs* genes is found to vary between species. Similar variations in the numbers of genes within gene families involved in insulin signaling in vertebrates have previously been reported [38, 66, 67]. Early studies demonstrated that the *Irs3* gene was lost on the human lineage [21], and our analysis indicates that it was possibly inactivated by the insertion of a repetitive DNA element early in primate evolution (results not shown). *Irs3* genes were also lost on the lineage leading to birds. A number of genes involved in insulin-regulated metabolism have been lost in the chicken [68], some of which have been shown to be missing in wide variety of birds (e.g., Resistin [64]), suggesting that the loss of Irs3 might have been part of an adaptation by birds to their new locomotive style. Teleost fish experienced a genome duplication [53], however rapid loss of many of the duplicates occurred [54]. Here we found duplicated copies of *Irs2*, *Irs3*, and *Irs4* in most teleost fish genomes, but most of these species have lost both copies of *Irs1* (see Table 1 and Additional file 1: Table S1 and Additional file 2: Tables S2). The presence of multiple *Irs* genes, and the overlap in the functions of the Irs proteins [14–18] suggests a degree of redundancy among these genes allowing species to adapt to the loss of one (or more) of these genes.

### Evolution of Irs proteins

Duplication of genes should allow the specialization of distinct proteins to unique biological roles [69, 70], thus duplication of the *Irs* genes might have allowed the evolution of novel regulatory roles for the insulin signaling pathway. While all Irs proteins are involved in insulin signaling, they each appear to have unique, but to some extent overlapping, biological roles [14–18]. Changes in the numbers of *Irs* genes also shows that the genes have retained a degree of redundancy and have not completely sub-functionalized since their origin. Despite the overlap in function, differences in evolutionary patters can be seen among the *Irs* genes. Irs3 and Irs4 both experienced episodes of more rapid protein sequence evolution on the common ancestral lineage leading to mammals (Additional file 10: Table S3), which suggests either a temporary relaxation of evolutionary constraints on these sequences on this lineage or that the rapid evolution was driven by positive selection. Both patterns of evolution could have resulted in changed biological functions for these proteins, and might explain why Irs3 and Irs4 might have functions that are less essential than Irs1 or Irs2. *Irs3* is non-essential as loss of this gene in humans is tolerated [21], and our data shows that a number of primates, birds and potentially other vertebrates can survive without this gene. Knockout of *Irs4* has little physiological effect [29], while *Irs1* or *Irs2* knockout mice have much more pronounced physiological defects [26, 27, 71, 72].

Further evidence for the diversification of the function of the Irs proteins is derived from the conservation plots. When each Irs protein is individually examined, areas of strong sequence conservation are seen across the entire protein sequence, although to a lower extent for Irs3, which might be due to the rapid evolution on the early mammalian lineage (Fig. 3a-d). However, when conservation is examined across the family of Irs proteins (Fig. 3e), most of the conservation is concentrated in the regions encoding the PH and PTB domains, sequences that are important for localizing these proteins to the plasma membrane [19] and insulin receptors [20], respectively. The plasma membrane localization, and insulin receptor interactions of these proteins have been conserved, but the C-terminal extension, which allow interaction with downstream signaling partners [15, 18], show greater levels of divergence to account for changes in downstream functions. However, there are a few areas of the C-terminal extension that are strongly conserved among all Irs, including two putative tyrosine phosphorylation sites that have been shown to be important in Irs1 and Irs2 for interactions with phosphatidylinositol 3-kinase (PI3K) [73–76], a key downstream signaling protein of insulin receptors [76]. Thus, interaction with PI3K appears to be conserved among all Irs proteins, but changes in interactions with other signaling proteins might explain the differences in biological function of the different Irs proteins.

## Conclusions

Here we have shown that the *Irs* gene family originated early in vertebrate evolution, with at least three of the genes likely generated during the two rounds of genome duplication that occurred in the vertebrate ancestor. Most groups of vertebrates have retained all 4 *Irs* genes, although some groups have lost genes, including primates and birds that have lost *Irs3* and most fish that have lost *Irs1*. Duplication of *Irs* genes is only seen in fish that have experienced the fish-specific genome duplication, leading to duplicated *Irs2*, *Irs3*, and *Irs4* genes. This suggests that while there are redundancies in the function of *Irs* gene, thus can tolerate the loss of a gene, gain of *Irs* genes is likely harmful, except when other genes in the insulin signaling pathway are duplicated. This conclusion is agreement with the finding of an increased number of retained duplicated genes involved in signal transduction pathways found in fish after the fish-specific genome duplications [77]. The protein sequences of Irs1 and Irs2 are strongly conserved across vertebrates while Irs3 and Irs4 show lower levels of conservation. In addition to lower sequence conservation, the length of Irs3 progressively shorted along the lineage leading to mammals. Comparisons among the paralogous Irs sequences shows that most of the sequence is well conserved within a paralog, but only the PH and TTB domains, those responsible for binding to plasma membranes and the insulin receptor, are conserved between paralogs. Only a few regions within the C-terminal extensions of these proteins are conserved among Irs paralogs, suggesting that divergence in these sequences has allowed divergence in function.

## Methods

### Database searches

Molecular sequence databases maintained by *Ensembl* [44] and the National Center for Biotechnology Information (*NCBI*) [47] were searched in January 2016 for insulin receptor substrate (*Irs1*, *Irs2*, *Irs3*, and *Irs4*)-like coding sequences. We initially searched the databases using the *tBLASTn* algorithm [43] using previously characterized mouse Irs1, Irs2, Irs3, and Irs4 protein sequences as queries. Putative Irs-like protein sequences identified were then used in subsequent tBLASTn searches. We also investigated the elephant shark (the sole representative of cartilaginous fish with a near-complete genome sequence) genome generated by the *Elephant Shark Genome Project* [45, 78]. All sequences that had E-scores below 0.01 were examined. Sequences identified by *BLAST* were used in reciprocal *BLASTx* searches of the mouse proteomes to ensure that their best matches were Irs-like sequences.

To examine genomic neighborhoods near *Irs*-like genes genomic comparisons were conducted using *PipMaker* and *MultiPipMaker* [56, 57]. Genes neighboring the *Irs*-like genes were identified from the genome assemblies in *Ensembl* [44] and the *Elephant Shark Genome Project* [78]. The organization of genes adjacent to the *Irs*-like genes was used to determine whether the genes of interest reside in conserved genomic neighborhoods.

### Phylogenetic analysis

Phylogenies of vertebrate *Irs*-like gene coding sequences were generated using full-length, or near full-length (i.e., missing a short part of their N- or C-termini), *Irs1*, *Irs2*, *Irs3*, and *Irs4* coding sequences from diverse vertebrate and outgroups (see Additional file 1: Table S1 and Additional file 2: Tables S2) and outgroups. *Irs*-like coding sequences were aligned using *MAFFT* [79] as implemented at the *Guidance* web site [80, 81], using default parameters. Similar results were obtained if *Clustal Omega* [82] was used as the alignment program. DNA sequence alignments were based on codons to retain protein alignments. The reliability of the alignments was examined using *Guidance* [80, 81] and trimmed alignments using sites that had values above the default cut-off of 0.93 were generated.

Phylogenetic trees of the sequences were generated using Bayesian methods with *MrBayes* 3.2 [50, 51, 83], maximum likelihood with *IQ-tree* [49, 84], and neighbor-joining distance approaches with *MEGA6.06* [85]. Bayesian trees were generated from coding sequences with *MrBayes* 3.2 using parameters selected by *ModelFinder* [86], whose results are presented in Additional file 13: Figure S9. *MrBayes* was run for 2,000,000 generations with four simultaneous Metropolis-coupled Monte Carlo Markov chains sampled every 100 generations. The average standard deviation of split frequencies dropped to less than 0.02 for all analyses. The first 25% of the trees were discarded as burn-in with the remaining samples used to generate the consensus trees. Trace files generated by *MrBayes* were examined by *Tracer* [87] to verify if they had converged. Maximum likelihood trees, constructed with 1000 replications by the ultrafast approximation [88], were generated with *IQ-tree* [49] on the *IQ-tree* webserver [84] using parameters for the substitution model suggested by *ModelFinder* [86]. The maximum likelihood search was initiated from a tree generated by *BIONJ* and the best tree was identified after heuristic searches using the nearest neighbor interchange (NNI) algorithm. *MEGA6.06* [85] was used to construct bootstrapped (1000 replications) neighbor-joining distance trees, using either Maximum Composite Likelihood distances for the DNA sequences or JTT distances for the proteins sequences. Similar results were obtained, but with lower confidence (bootstrap or posterior probabilities) intervals if alternative outgroups were used (results not shown).

With respect to orthology-paralogy issues, choice of outgroup, alignment method (*MAFFT* [79] or *Clustal* [82]), or the use of full-length or trimmed (based on *Guidance* scores [81]) alignments had little influence on the key findings of these analyses. Methods that relied on shorter sequences (i.e., trimmed alignments or protein sequences) or simpler models of sequence evolution (i.e., neighbor-joining or parsimony) tended to yield weaker support for the earlier diverging lineages, but none of our analyses were in significant conflict with the key inferences of the phylogeny presented in Fig. 2 or Additional file 11: Figure S8.

### Analysis of protein sequence conservation

Conservation of proteins sequences was assessed using Jenson-Shannon (JS) divergence scores [62] on the *JS Divergence* web server [89], using a window size of 3 and the BLOSUM62 matrix as background. Putative tyrosine phosphorylation sites in the protein sequences were predicted using *NetPhos* [63, 90].

## Additional files

Additional file 1: Table S1. This file is in Excel format. Genomic locations of *Irs*-like genes in sequenced vertebrate genomes identified from the *Ensembl* database. (XLSX 40 kb)

Additional file 2: Table S2. This file is in Excel format. Accession numbers of *Irs*-like coding sequences from vertebrates and outroup species identified from the NCBI database. (XLSX 218 kb)

Additional file 3: Figure S1. This file is in Word format. Coding sequences for full-length and near full-length Irs genes from diverse vertebrates and outgroups. (PDF 200 kb)

Additional file 4: Figure S2. This file is in PDF format. Phylogeny of vertebrate *Irs1* sequences. (PDF 172 kb)

Additional file 5: Figure S3. This file is in PDF format. Phylogeny of vertebrate *Irs2* sequences. (XLSX 58 kb)

Additional file 6: Figure S4. This file is in PDF format. Phylogeny of vertebrate *Irs3* sequences. (XLSX 53 kb)

Additional file 7: Figure S5. This file is in PDF format. Phylogeny of vertebrate *Irs4* sequences. (DOCX 627 kb)

Additional file 8: Figure S6. This file is in PDF format. Phylogeny of the vertebrate *Irs* gene family rooted with non-vertebrate *Irs*-like genes. (PDF 525 kb)

Additional file 9: Figure S7. This file is in PDF format. Alignment of primate *Irs3* genomic sequences. (PDF 518 kb)

Additional file 10: Table S3. This file is in Excel format. Relative rates of evolution of *Irs* genes in mammals and non-mammals. (PDF 518 kb)

Additional file 11: Table S4. This file is in Excel format. JS Divergence Scores from alignments of Irs proteins from 10 vertebrates. (PDF 499 kb)

Additional file 12: Figure S8. This file is in PDF format. Alignment of Irs protein sequences with conserved residues highlighted. (PDF 450 kb)

Additional file 13: Figure S9. This file is in PDF format. *ModelFinder* results for the coding sequences used in the phylogenetic analyses. (PDF 224 kb)
