# Supplementary material for: Evolution of the vertebrate insulin receptor substrate (Irs) gene family
Source: BMC Evol Biol. 2017 Jun 23;17:148. doi: 10.1186/s12862-017-0994-z (PMC5482937; doi:10.1186/s12862-017-0994-z)
Supplement: Supplementary file 4 — This file is in PDF format. Phylogeny of vertebrate Irs1 sequences. (PDF 172 kb) [file 12862_2017_994_MOESM13_ESM.pdf]

A Irs1

Best-fit model according to BIC: K3Pu+I+G4

List of models sorted by BIC scores:

Model	LogL	AIC	w-AIC	AICc	w-AICc	BIC	w-BIC
K3Pu+I+G4	-37061.1078	74486.2156	+ 0.0944	74520.1148	+ 0.1316	75518.5728	+ 0.7881
TIM+I+G4	-37058.6823	74483.3646	+ 0.3927	74517.6538	+ 0.4505	75521.3941	+ 0.1923
HKY+I+G4	-37069.2419	74500.4839	- 0.0001	74533.9956	- 0.0001	75527.1688	- 0.0107
TVM+I+G4	-37058.8578	74485.7156	+ 0.1212	74520.3972	+ 0.1143	75529.4174	- 0.0035
TN+I+G4	-37066.9425	74497.8850	- 0.0003	74531.7843	- 0.0004	75530.2423	- 0.0023
TPM3u+I+G4	-37068.1624	74500.3247	- 0.0001	74534.2240	- 0.0001	75532.6820	- 0.0007
TPM3+I+G4	-37068.1731	74500.3463	- 0.0001	74534.2455	- 0.0001	75532.7035	- 0.0007
GTR+I+G4	-37056.6877	74483.3753	+ 0.3907	74518.4518	+ 0.3023	75532.7494	- 0.0007
TPM2u+I+G4	-37068.5323	74501.0646	- 0.0001	74534.9638	- 0.0001	75533.4218	- 0.0005
TPM2+I+G4	-37068.5328	74501.0656	- 0.0001	74534.9648	- 0.0001	75533.4228	- 0.0005
TIM3+I+G4	-37066.0278	74498.0555	- 0.0003	74532.3447	- 0.0003	75536.0850	- 0.0001
TIM2+I+G4	-37067.2053	74500.4107	- 0.0001	74534.6999	- 0.0001	75538.4402	- 0.0000
SYM+I+G4	-37126.2243	74616.4487	- 0.0000	74650.3479	- 0.0000	75648.8059	- 0.0000
TIM3e+I+G4	-37133.9153	74627.8305	- 0.0000	74660.9571	- 0.0000	75648.8432	- 0.0000
TNe+I+G4	-37155.7739	74669.5478	- 0.0000	74702.2917	- 0.0000	75684.8881	- 0.0000
TIMe+I+G4	-37152.2099	74664.4198	- 0.0000	74697.5464	- 0.0000	75685.4325	- 0.0000
TVMe+I+G4	-37150.8461	74663.6923	- 0.0000	74697.2040	- 0.0000	75690.3772	- 0.0000
TIM2e+I+G4	-37155.6599	74671.3197	- 0.0000	74704.4463	- 0.0000	75692.3324	- 0.0000
K2P+I+G4	-37176.5262	74709.0524	- 0.0000	74741.4160	- 0.0000	75718.7204	- 0.0000
K3P+I+G4	-37173.1008	74704.2016	- 0.0000	74736.9455	- 0.0000	75719.5420	- 0.0000
K3Pu+G4	-37284.3723	74930.7446	- 0.0000	74964.2563	- 0.0000	75957.4296	- 0.0000
TVM+G4	-37277.9702	74921.9403	- 0.0000	74956.2295	- 0.0000	75959.9699	- 0.0000
TPM3+G4	-37285.7080	74933.4159	- 0.0000	74966.9276	- 0.0000	75960.1009	- 0.0000
TPM3u+G4	-37285.7081	74933.4162	- 0.0000	74966.9279	- 0.0000	75960.1011	- 0.0000
HKY+G4	-37290.7158	74941.4317	- 0.0000	74974.5583	- 0.0000	75962.4443	- 0.0000
GTR+G4	-37277.1066	74922.2132	- 0.0000	74956.8949	- 0.0000	75965.9151	- 0.0000
TIM+G4	-37285.9029	74935.8059	- 0.0000	74969.7051	- 0.0000	75968.1631	- 0.0000
TIM3+G4	-37285.9369	74935.8737	- 0.0000	74969.7729	- 0.0000	75968.2309	- 0.0000
TPM2u+G4	-37290.5128	74943.0255	- 0.0000	74976.5372	- 0.0000	75969.7105	- 0.0000
TPM2+G4	-37290.5128	74943.0257	- 0.0000	74976.5374	- 0.0000	75969.7106	- 0.0000
TN+G4	-37290.6346	74943.2692	- 0.0000	74976.7809	- 0.0000	75969.9542	- 0.0000
TIM2+G4	-37290.6645	74945.3290	- 0.0000	74979.2282	- 0.0000	75977.6862	- 0.0000
SYM+G4	-37335.4868	75032.9736	- 0.0000	75066.4853	- 0.0000	76059.6585	- 0.0000
TIM3e+G4	-37344.1919	75046.3837	- 0.0000	75079.1276	- 0.0000	76061.7241	- 0.0000
TVMe+G4	-37354.6588	75069.3177	- 0.0000	75102.4443	- 0.0000	76090.3303	- 0.0000
TNe+G4	-37371.3725	75098.7450	- 0.0000	75131.1087	- 0.0000	76108.4131	- 0.0000
TIMe+G4	-37367.9417	75093.8834	- 0.0000	75126.6273	- 0.0000	76109.2237	- 0.0000
TIM2e+G4	-37371.3713	75100.7426	- 0.0000	75133.4865	- 0.0000	76116.0830	- 0.0000
K2P+G4	-37387.6280	75129.2560	- 0.0000	75161.2418	- 0.0000	76133.2517	- 0.0000
K3P+G4	-37384.0399	75124.0798	- 0.0000	75156.4435	- 0.0000	76133.7479	- 0.0000
F81+I+G4	-38679.7916	77719.5832	- 0.0000	77752.7098	- 0.0000	78740.5958	- 0.0000
TIM3+I	-38828.6167	78021.2333	- 0.0000	78055.1326	- 0.0000	79053.5906	- 0.0000
TN+I	-38835.0529	78032.1058	- 0.0000	78065.6175	- 0.0000	79058.7907	- 0.0000
TIM+I	-38832.2237	78028.4474	- 0.0000	78062.3466	- 0.0000	79060.8046	- 0.0000
GTR+I	-38825.7646	78019.5293	- 0.0000	78054.2109	- 0.0000	79063.2311	- 0.0000
TIM2+I	-38834.8893	78033.7787	- 0.0000	78067.6779	- 0.0000	79066.1359	- 0.0000
JC+I+G4	-38884.6959	78123.3917	- 0.0000	78155.3775	- 0.0000	79127.3875	- 0.0000
HKY+I	-38885.6008	78131.2017	- 0.0000	78164.3283	- 0.0000	79152.2143	- 0.0000
K3Pu+I	-38882.1184	78126.2367	- 0.0000	78159.7484	- 0.0000	79152.9217	- 0.0000
TPM3u+I	-38882.9664	78127.9329	- 0.0000	78161.4446	- 0.0000	79154.6178	- 0.0000
TPM3+I	-38882.9715	78127.9429	- 0.0000	78161.4546	- 0.0000	79154.6278	- 0.0000
F81+G4	-38891.8452	78141.6904	- 0.0000	78174.4343	- 0.0000	79157.0307	- 0.0000
TPM2u+I	-38885.3706	78132.7413	- 0.0000	78166.2530	- 0.0000	79159.4262	- 0.0000
TPM2+I	-38885.3714	78132.7428	- 0.0000	78166.2545	- 0.0000	79159.4277	- 0.0000
TVM+I	-38880.0721	78126.1441	- 0.0000	78160.4333	- 0.0000	79164.1737	- 0.0000
TNe+I	-38949.2109	78254.4217	- 0.0000	78286.7854	- 0.0000	79264.0898	- 0.0000
TIM3e+I	-38947.7245	78253.4490	- 0.0000	78286.1929	- 0.0000	79268.7894	- 0.0000
TIMe+I	-38948.1376	78254.2753	- 0.0000	78287.0192	- 0.0000	79269.6156	- 0.0000
TIM2e+I	-38949.2392	78256.4785	- 0.0000	78289.2224	- 0.0000	79271.8188	- 0.0000
SYM+I	-38946.1234	78254.2468	- 0.0000	78287.7585	- 0.0000	79280.9318	- 0.0000
K2P+I	-39015.4993	78384.9986	- 0.0000	78416.9844	- 0.0000	79388.9944	- 0.0000
K3P+I	-39014.4135	78384.8270	- 0.0000	78417.1907	- 0.0000	79394.4951	- 0.0000
TVMe+I	-39012.0125	78384.0251	- 0.0000	78417.1517	- 0.0000	79405.0377	- 0.0000

JC+G4	-39097.8152	78547.6304 - 0.0000	78579.2408 - 0.0000	79545.9539 - 0.0000
F81+I	-40408.5758	81175.1515 - 0.0000	81207.8954 - 0.0000	82190.4919 - 0.0000
JC+I	-40535.4801	81422.9601 - 0.0000	81454.5705 - 0.0000	82421.2836 - 0.0000
GTR	-42668.0602	85702.1205 - 0.0000	85736.4097 - 0.0000	86740.1500 - 0.0000
TIM3	-42695.1474	85752.2948 - 0.0000	85785.8065 - 0.0000	86778.9797 - 0.0000
SYM	-42713.0640	85786.1280 - 0.0000	85819.2546 - 0.0000	86807.1407 - 0.0000
TIM2	-42709.2339	85780.4679 - 0.0000	85813.9796 - 0.0000	86807.1528 - 0.0000
TIM3e	-42721.1774	85798.3549 - 0.0000	85830.7185 - 0.0000	86808.0229 - 0.0000
TN	-42730.9523	85821.9045 - 0.0000	85855.0311 - 0.0000	86842.9172 - 0.0000
TIM	-42730.3111	85822.6222 - 0.0000	85856.1339 - 0.0000	86849.3071 - 0.0000
TNe	-42766.3174	85886.6347 - 0.0000	85918.6205 - 0.0000	86890.6305 - 0.0000
TIM2e	-42763.5235	85883.0469 - 0.0000	85915.4105 - 0.0000	86892.7150 - 0.0000
TIMe	-42764.8343	85885.6685 - 0.0000	85918.0322 - 0.0000	86895.3366 - 0.0000
TVM	-42796.9935	85957.9871 - 0.0000	85991.8863 - 0.0000	86990.3443 - 0.0000
TPM3u	-42816.3968	85992.7935 - 0.0000	86025.9201 - 0.0000	87013.8062 - 0.0000
TPM3	-42816.6065	85993.2131 - 0.0000	86026.3397 - 0.0000	87014.2257 - 0.0000
TPM2	-42858.3573	86076.7145 - 0.0000	86109.8411 - 0.0000	87097.7272 - 0.0000
TPM2u	-42858.3991	86076.7982 - 0.0000	86109.9248 - 0.0000	87097.8108 - 0.0000
HKY	-42871.4414	86100.8827 - 0.0000	86133.6266 - 0.0000	87116.2231 - 0.0000
K3Pu	-42870.6205	86101.2410 - 0.0000	86134.3676 - 0.0000	87122.2537 - 0.0000
TVMe	-42896.1006	86150.2013 - 0.0000	86182.9452 - 0.0000	87165.5416 - 0.0000
K2P	-42948.3723	86248.7445 - 0.0000	86280.3549 - 0.0000	87247.0680 - 0.0000
K3P	-42946.8573	86247.7146 - 0.0000	86279.7004 - 0.0000	87251.7104 - 0.0000
F81	-44289.3810	88934.7621 - 0.0000	88967.1257 - 0.0000	89944.4302 - 0.0000
JC	-44381.6115	89113.2230 - 0.0000	89144.4604 - 0.0000	90105.8742 - 0.0000

SUBSTITUTION PROCESS

Model of substitution: K3Pu+F+I+G4

Rate parameter R:

```
  A-C: 1.0000
  A-G: 4.5418
  A-T: 0.6837
  C-G: 0.6837
  C-T: 4.5418
  G-T: 1.0000
```

State frequencies: (empirical counts from alignment)

```
  pi(A) = 0.221
  pi(C) = 0.3233
  pi(G) = 0.264
  pi(T) = 0.1917
```

Rate matrix Q:

A	-1.094	0.2139	0.7933	0.08669
C	0.1462	-0.8415	0.1194	0.5759
G	0.664	0.1462	-0.937	0.1268
T	0.09996	0.9713	0.1747	-1.246

Model of rate heterogeneity: Invar+Gamma with 4 categories
Proportion of invariable sites: 0.4083
Gamma shape alpha: 0.7233

Category	Relative_rate	Proportion
0	0	0.4083
1	0.1338	0.1479
2	0.6326	0.1479
3	1.577	0.1479
4	4.416	0.1479

Relative rates are computed as MEAN of the portion of the Gamma distribution falling in the category.

B Irs2

Best-fit model according to BIC: TPM2u+I+G4

List of models sorted by BIC scores:

Model	LogL	AIC	w-AIC	AICc	w-AICc	BIC	w-BIC
TPM2u+I+G4	-37565.4173	75458.8346	+ 0.0927	75493.7282	+ 0.1272	76352.2658	+ 0.4371
TPM2+I+G4	-37565.4221	75458.8442	+ 0.0923	75493.7378	+ 0.1266	76352.2754	+ 0.4350
TIM2+I+G4	-37562.9478	75455.8956	+ 0.4031	75491.2376	+ 0.4420	76354.7746	+ 0.1247
TVM+I+G4	-37563.4013	75458.8027	+ 0.0942	75494.5961	+ 0.0824	76363.1294	- 0.0019
GTR+I+G4	-37561.1871	75456.3742	+ 0.3173	75492.6222	+ 0.2212	76366.1486	- 0.0004
HKY+I+G4	-37576.6532	75479.3063	- 0.0000	75513.7548	- 0.0000	76367.2898	- 0.0002
TN+I+G4	-37572.9708	75473.9415	- 0.0000	75508.8352	- 0.0001	76367.3727	- 0.0002
K3Pu+I+G4	-37573.2368	75474.4736	- 0.0000	75509.3672	- 0.0001	76367.9048	- 0.0002
TIM+I+G4	-37570.1249	75470.2498	- 0.0003	75505.5917	- 0.0003	76369.1287	- 0.0001
TPM3u+I+G4	-37575.1797	75478.3595	- 0.0000	75513.2531	- 0.0000	76371.7907	- 0.0000
TPM3+I+G4	-37575.1845	75478.3691	- 0.0000	75513.2627	- 0.0000	76371.8003	- 0.0000
TIM3+I+G4	-37572.1629	75474.3258	- 0.0000	75509.6677	- 0.0000	76373.2047	- 0.0000
TIM3e+I+G4	-37638.5682	75601.1364	- 0.0000	75635.1429	- 0.0000	76483.6721	- 0.0000
SYM+I+G4	-37637.3234	75602.6467	- 0.0000	75637.5403	- 0.0000	76496.0779	- 0.0000
TVMe+I+G4	-37652.4550	75630.9099	- 0.0000	75665.3584	- 0.0000	76518.8934	- 0.0000
TPM2u+G4	-37662.2263	75650.4526	- 0.0000	75684.9011	- 0.0000	76538.4361	- 0.0000
TPM2+G4	-37662.3571	75650.7141	- 0.0000	75685.1626	- 0.0000	76538.6976	- 0.0000
TNe+I+G4	-37672.7641	75667.5282	- 0.0000	75701.0957	- 0.0000	76544.6161	- 0.0000
TIM2+G4	-37662.2305	75652.4610	- 0.0000	75687.3546	- 0.0000	76545.8922	- 0.0000
TVM+G4	-37658.8967	75647.7935	- 0.0000	75683.1354	- 0.0000	76546.6724	- 0.0000
TIM2e+I+G4	-37671.1428	75666.2856	- 0.0000	75700.2921	- 0.0000	76548.8213	- 0.0000
TIMe+I+G4	-37672.7707	75669.5413	- 0.0000	75703.5478	- 0.0000	76552.0770	- 0.0000
GTR+G4	-37659.0645	75650.1290	- 0.0000	75685.9224	- 0.0000	76554.4557	- 0.0000
HKY+G4	-37674.2248	75672.4496	- 0.0000	75706.4560	- 0.0000	76554.9853	- 0.0000
K3Pu+G4	-37670.9718	75667.9436	- 0.0000	75702.3920	- 0.0000	76555.9270	- 0.0000
TPM3u+G4	-37671.7805	75669.5611	- 0.0000	75704.0095	- 0.0000	76557.5445	- 0.0000
TPM3+G4	-37671.7903	75669.5806	- 0.0000	75704.0290	- 0.0000	76557.5640	- 0.0000
TN+G4	-37672.2657	75670.5315	- 0.0000	75704.9799	- 0.0000	76558.5149	- 0.0000
TIM+G4	-37668.8899	75665.7799	- 0.0000	75700.6735	- 0.0000	76559.2111	- 0.0000
TIM3+G4	-37669.6211	75667.2422	- 0.0000	75702.1358	- 0.0000	76560.6734	- 0.0000
K2P+I+G4	-37684.6949	75689.3898	- 0.0000	75722.5216	- 0.0000	76561.0300	- 0.0000
K3P+I+G4	-37684.6953	75691.3906	- 0.0000	75724.9581	- 0.0000	76568.4785	- 0.0000
TIM3e+G4	-37733.1795	75788.3589	- 0.0000	75821.9265	- 0.0000	76665.4469	- 0.0000
SYM+G4	-37731.9635	75789.9269	- 0.0000	75824.3754	- 0.0000	76677.9104	- 0.0000
TVMe+G4	-37747.1759	75818.3518	- 0.0000	75852.3583	- 0.0000	76700.8876	- 0.0000
TNe+G4	-37768.0604	75856.1208	- 0.0000	75889.2526	- 0.0000	76727.7610	- 0.0000
TIM2e+G4	-37766.4461	75854.8922	- 0.0000	75888.4597	- 0.0000	76731.9801	- 0.0000
TIMe+G4	-37768.1004	75858.2007	- 0.0000	75891.7683	- 0.0000	76735.2887	- 0.0000
K2P+G4	-37780.3495	75878.6989	- 0.0000	75911.3982	- 0.0000	76744.8914	- 0.0000
K3P+G4	-37780.3504	75880.7008	- 0.0000	75913.8327	- 0.0000	76752.3411	- 0.0000
F81+I+G4	-38774.4174	77872.8349	- 0.0000	77906.8413	- 0.0000	78755.3706	- 0.0000
JC+I+G4	-38847.8292	78013.6585	- 0.0000	78046.3577	- 0.0000	78879.8509	- 0.0000
F81+G4	-38866.8691	78055.7382	- 0.0000	78089.3058	- 0.0000	78932.8262	- 0.0000
JC+G4	-38940.5969	78197.1937	- 0.0000	78229.4635	- 0.0000	79057.9384	- 0.0000
GTR+I	-39395.2398	79122.4797	- 0.0000	79158.2731	- 0.0000	80026.8064	- 0.0000
TIM2+I	-39407.0384	79142.0768	- 0.0000	79176.9704	- 0.0000	80035.5080	- 0.0000
TIM3+I	-39415.7389	79159.4778	- 0.0000	79194.3714	- 0.0000	80052.9090	- 0.0000
TN+I	-39425.7449	79177.4897	- 0.0000	79211.9382	- 0.0000	80065.4732	- 0.0000
TIM+I	-39424.8360	79177.6721	- 0.0000	79212.5657	- 0.0000	80071.1033	- 0.0000
TVM+I	-39432.3314	79194.6629	- 0.0000	79230.0048	- 0.0000	80093.5418	- 0.0000
TPM3+I	-39447.6397	79221.2793	- 0.0000	79255.7278	- 0.0000	80109.2628	- 0.0000
TPM3u+I	-39447.6470	79221.2940	- 0.0000	79255.7424	- 0.0000	80109.2775	- 0.0000
TIM3e+I	-39455.3703	79232.7405	- 0.0000	79266.3081	- 0.0000	80109.8285	- 0.0000
SYM+I	-39450.0157	79226.0314	- 0.0000	79260.4798	- 0.0000	80114.0149	- 0.0000
TPM2u+I	-39454.2048	79234.4095	- 0.0000	79268.8580	- 0.0000	80122.3930	- 0.0000
TPM2+I	-39454.2609	79234.5217	- 0.0000	79268.9702	- 0.0000	80122.5052	- 0.0000
HKY+I	-39466.8369	79257.6739	- 0.0000	79291.6803	- 0.0000	80140.2096	- 0.0000
K3Pu+I	-39466.1520	79258.3040	- 0.0000	79292.7525	- 0.0000	80146.2875	- 0.0000
TIM2e+I	-39491.1413	79304.2826	- 0.0000	79337.8501	- 0.0000	80181.3705	- 0.0000
TNe+I	-39495.0965	79310.1930	- 0.0000	79343.3248	- 0.0000	80181.8332	- 0.0000
TIMe+I	-39494.7584	79311.5168	- 0.0000	79345.0844	- 0.0000	80188.6048	- 0.0000
TVMe+I	-39497.6315	79319.2629	- 0.0000	79353.2694	- 0.0000	80201.7986	- 0.0000
K2P+I	-39542.7638	79403.5276	- 0.0000	79436.2268	- 0.0000	80269.7200	- 0.0000

K3P+I	-39542.4090	79404.8180 - 0.0000	79437.9498 - 0.0000	80276.4582 - 0.0000
F81+I	-40544.1500	81410.2999 - 0.0000	81443.8675 - 0.0000	82287.3879 - 0.0000
JC+I	-40598.4200	81512.8401 - 0.0000	81545.1098 - 0.0000	82373.5848 - 0.0000
GTR	-42027.0906	84384.1813 - 0.0000	84419.5232 - 0.0000	85283.0603 - 0.0000
TVM	-42068.3007	84464.6014 - 0.0000	84499.4950 - 0.0000	85358.0326 - 0.0000
TIM3	-42075.2664	84476.5328 - 0.0000	84510.9812 - 0.0000	85364.5162 - 0.0000
TPM3u	-42109.0436	84542.0872 - 0.0000	84576.0936 - 0.0000	85424.6229 - 0.0000
TPM3	-42109.0803	84542.1607 - 0.0000	84576.1671 - 0.0000	85424.6964 - 0.0000
SYM	-42112.3351	84548.6703 - 0.0000	84582.6767 - 0.0000	85431.2060 - 0.0000
TIM3e	-42122.6247	84565.2493 - 0.0000	84598.3812 - 0.0000	85436.8895 - 0.0000
TIM2	-42163.2091	84652.4181 - 0.0000	84686.8666 - 0.0000	85540.4016 - 0.0000
TVMe	-42192.3188	84706.6375 - 0.0000	84740.2051 - 0.0000	85583.7255 - 0.0000
TN	-42204.6044	84733.2088 - 0.0000	84767.2153 - 0.0000	85615.7446 - 0.0000
TIM	-42203.2961	84732.5923 - 0.0000	84767.0407 - 0.0000	85620.5757 - 0.0000
TIM2e	-42227.6273	84775.2545 - 0.0000	84808.3863 - 0.0000	85646.8947 - 0.0000
TNe	-42235.5146	84789.0292 - 0.0000	84821.7285 - 0.0000	85655.2217 - 0.0000
TIMe	-42235.2675	84790.5351 - 0.0000	84823.6669 - 0.0000	85662.1753 - 0.0000
TPM2u	-42232.4589	84788.9179 - 0.0000	84822.9243 - 0.0000	85671.4536 - 0.0000
TPM2	-42232.6638	84789.3276 - 0.0000	84823.3340 - 0.0000	85671.8633 - 0.0000
HKY	-42264.1695	84850.3390 - 0.0000	84883.9066 - 0.0000	85727.4270 - 0.0000
K3Pu	-42263.0426	84850.0852 - 0.0000	84884.0917 - 0.0000	85732.6210 - 0.0000
K2P	-42312.7499	84941.4997 - 0.0000	84973.7695 - 0.0000	85802.2444 - 0.0000
K3P	-42312.4719	84942.9437 - 0.0000	84975.6430 - 0.0000	85809.1362 - 0.0000
F81	-43270.2443	86860.4887 - 0.0000	86893.6205 - 0.0000	87732.1289 - 0.0000
JC	-43326.6720	86967.3441 - 0.0000	86999.1874 - 0.0000	87822.6410 - 0.0000

SUBSTITUTION PROCESS

Model of substitution: TPM2u+F+I+G4

Rate parameter R:

A-C: 1.2830
A-G: 4.5975
A-T: 1.2830
C-G: 1.0000
C-T: 4.5975
G-T: 1.0000

State frequencies: (empirical counts from alignment)

pi(A) = 0.2328
pi(C) = 0.3089
pi(G) = 0.2664
pi(T) = 0.1919

Rate matrix Q:

A	-1.111	0.2357	0.7284	0.1464
C	0.1777	-0.8609	0.1584	0.5248
G	0.6367	0.1837	-0.9345	0.1141
T	0.1777	0.8446	0.1584	-1.181

Model of rate heterogeneity: Invar+Gamma with 4 categories
Proportion of invariable sites: 0.2775
Gamma shape alpha: 0.7751

Category	Relative_rate	Proportion
0	0	0.2775
1	0.1249	0.1806
2	0.5494	0.1806
3	1.315	0.1806
4	3.547	0.1806

Relative rates are computed as MEAN of the portion of the Gamma distribution falling in the category.

C Irs3

Best-fit model according to BIC: TPM2u+I+G4

List of models sorted by BIC scores:

Model	LogL	AIC	w-AIC	AICc	w-AICc	BIC	w-BIC
TPM2u+I+G4	-11704.5770	23745.1540	- 0.0199	23885.3614	+ 0.1379	24476.3957	+ 0.2849
TPM2+I+G4	-11704.5959	23745.1918	- 0.0195	23885.3992	+ 0.1353	24476.4335	+ 0.2796
TIM2+I+G4	-11701.6144	23741.2288	+ 0.1417	23883.4565	+ 0.3575	24476.8232	+ 0.2301
TPM2u+G4	-11709.3772	23752.7544	- 0.0004	23890.9613	- 0.0084	24479.6435	+ 0.0562
TPM2+G4	-11709.3878	23752.7757	- 0.0004	23890.9826	- 0.0083	24479.6648	+ 0.0556
TIM+I+G4	-11703.4953	23744.9905	+ 0.0216	23887.2182	+ 0.0545	24480.5849	+ 0.0351
TIM2+G4	-11706.9558	23749.9116	- 0.0018	23890.1190	- 0.0128	24481.1533	+ 0.0264
GTR+I+G4	-11697.9391	23737.8781	+ 0.7569	23884.2065	+ 0.2457	24482.1778	- 0.0158
TN+I+G4	-11708.3463	23752.6925	- 0.0005	23892.9000	- 0.0032	24483.9343	- 0.0066
TVM+I+G4	-11702.1434	23744.2868	+ 0.0307	23888.5547	+ 0.0279	24484.2338	- 0.0057
TIM3+I+G4	-11706.6846	23751.3693	- 0.0009	23893.5970	- 0.0022	24486.9637	- 0.0014
GTR+G4	-11703.9772	23747.9545	- 0.0049	23892.2225	- 0.0045	24487.9015	- 0.0009
TVM+G4	-11707.2788	23752.5576	- 0.0005	23894.7853	- 0.0012	24488.1520	- 0.0008
K3Pu+I+G4	-11710.8652	23757.7304	- 0.0000	23897.9378	- 0.0003	24488.9721	- 0.0005
TIM+G4	-11711.3943	23758.7887	- 0.0000	23898.9961	- 0.0002	24490.0304	- 0.0003
HKY+I+G4	-11716.5259	23767.0519	- 0.0000	23905.2588	- 0.0000	24493.9410	- 0.0000
TN+G4	-11716.5838	23767.1676	- 0.0000	23905.3745	- 0.0000	24494.0567	- 0.0000
K3Pu+G4	-11717.6138	23769.2277	- 0.0000	23907.4346	- 0.0000	24496.1168	- 0.0000
TIM3+G4	-11715.4619	23766.9238	- 0.0000	23907.1312	- 0.0000	24498.1655	- 0.0000
TPM3u+I+G4	-11715.9328	23767.8656	- 0.0000	23908.0730	- 0.0000	24499.1073	- 0.0000
TPM3+I+G4	-11716.0413	23768.0827	- 0.0000	23908.2901	- 0.0000	24499.3244	- 0.0000
HKY+G4	-11723.4627	23778.9253	- 0.0000	23915.1514	- 0.0000	24501.4618	- 0.0000
TPM3+G4	-11723.0818	23780.1636	- 0.0000	23918.3705	- 0.0000	24507.0527	- 0.0000
TPM3u+G4	-11723.0824	23780.1648	- 0.0000	23918.3717	- 0.0000	24507.0539	- 0.0000
SYM+I+G4	-11793.8587	23923.7174	- 0.0000	24063.9248	- 0.0000	24654.9591	- 0.0000
SYM+G4	-11802.4512	23938.9023	- 0.0000	24077.1092	- 0.0000	24665.7914	- 0.0000
TIM3e+I+G4	-11806.4984	23944.9969	- 0.0000	24081.2229	- 0.0000	24667.5334	- 0.0000
TVMe+I+G4	-11807.0323	23948.0645	- 0.0000	24086.2714	- 0.0000	24674.9536	- 0.0000
TIM3e+G4	-11814.3252	23958.6504	- 0.0000	24092.9151	- 0.0000	24676.8342	- 0.0000
TVMe+G4	-11815.8279	23963.6558	- 0.0000	24099.8819	- 0.0000	24686.1923	- 0.0000
TIMe+I+G4	-11824.3117	23980.6234	- 0.0000	24116.8494	- 0.0000	24703.1598	- 0.0000
TIM2e+I+G4	-11828.6695	23989.3391	- 0.0000	24125.5651	- 0.0000	24711.8755	- 0.0000
TIMe+G4	-11833.5128	23997.0256	- 0.0000	24131.2903	- 0.0000	24715.2095	- 0.0000
K3P+I+G4	-11834.7754	23999.5508	- 0.0000	24133.8155	- 0.0000	24717.7347	- 0.0000
TNe+I+G4	-11837.5864	24005.1728	- 0.0000	24139.4375	- 0.0000	24723.3567	- 0.0000
TIM2e+G4	-11838.3120	24006.6240	- 0.0000	24140.8887	- 0.0000	24724.8078	- 0.0000
K3P+G4	-11843.8514	24015.7027	- 0.0000	24148.0254	- 0.0000	24729.5339	- 0.0000
TNe+G4	-11846.5872	24021.1744	- 0.0000	24153.4971	- 0.0000	24735.0056	- 0.0000
K2P+I+G4	-11848.1071	24024.2143	- 0.0000	24156.5370	- 0.0000	24738.0455	- 0.0000
K2P+G4	-11856.8883	24039.7765	- 0.0000	24170.1765	- 0.0000	24749.2551	- 0.0000
F81+I+G4	-12070.7182	24473.4364	- 0.0000	24609.6624	- 0.0000	25195.9729	- 0.0000
F81+G4	-12079.3243	24488.6486	- 0.0000	24622.9133	- 0.0000	25206.8325	- 0.0000
JC+I+G4	-12097.7041	24521.4082	- 0.0000	24651.8082	- 0.0000	25230.8868	- 0.0000
JC+G4	-12106.4522	24536.9045	- 0.0000	24665.4008	- 0.0000	25242.0304	- 0.0000
GTR+I	-12194.7311	24729.4622	- 0.0000	24873.7302	- 0.0000	25469.4092	- 0.0000
SYM+I	-12206.4019	24746.8037	- 0.0000	24885.0106	- 0.0000	25473.6928	- 0.0000
TIM2+I	-12207.4450	24750.8900	- 0.0000	24891.0974	- 0.0000	25482.1317	- 0.0000
TVM+I	-12210.9640	24759.9279	- 0.0000	24902.1557	- 0.0000	25495.5223	- 0.0000
TVMe+I	-12221.0460	24774.0920	- 0.0000	24910.3181	- 0.0000	25496.6285	- 0.0000
TPM2+I	-12234.7536	24803.5072	- 0.0000	24941.7140	- 0.0000	25530.3963	- 0.0000
TPM2u+I	-12234.7540	24803.5079	- 0.0000	24941.7148	- 0.0000	25530.3970	- 0.0000
TIM3+I	-12232.5914	24801.1829	- 0.0000	24941.3903	- 0.0000	25532.4246	- 0.0000
TIM+I	-12234.3067	24804.6135	- 0.0000	24944.8209	- 0.0000	25535.8552	- 0.0000
TIM3e+I	-12247.5247	24825.0494	- 0.0000	24959.3141	- 0.0000	25543.2332	- 0.0000
TPM3+I	-12243.2841	24820.5682	- 0.0000	24958.7751	- 0.0000	25547.4573	- 0.0000
TPM3u+I	-12243.2846	24820.5691	- 0.0000	24958.7760	- 0.0000	25547.4582	- 0.0000
TN+I	-12243.3303	24820.6606	- 0.0000	24958.8675	- 0.0000	25547.5497	- 0.0000
TIM2e+I	-12251.6062	24833.2123	- 0.0000	24967.4770	- 0.0000	25551.3962	- 0.0000
K3Pu+I	-12253.3334	24840.6668	- 0.0000	24978.8737	- 0.0000	25567.5559	- 0.0000
HKY+I	-12262.2329	24856.4659	- 0.0000	24992.6919	- 0.0000	25579.0024	- 0.0000
TIMe+I	-12269.5123	24869.0247	- 0.0000	25003.2894	- 0.0000	25587.2085	- 0.0000
K3P+I	-12284.1286	24896.2571	- 0.0000	25028.5799	- 0.0000	25610.0884	- 0.0000
TNe+I	-12288.5018	24905.0035	- 0.0000	25037.3262	- 0.0000	25618.8347	- 0.0000

K2P+I	-12303.1952	24932.3904 - 0.0000	25062.7904 - 0.0000	25641.8689 - 0.0000
JC+I	-12532.4891	25388.9781 - 0.0000	25517.4745 - 0.0000	26094.1041 - 0.0000
F81+I	-12525.3294	25380.6589 - 0.0000	25514.9236 - 0.0000	26098.8427 - 0.0000
GTR	-12620.8880	25579.7761 - 0.0000	25722.0038 - 0.0000	26315.3704 - 0.0000
TVM	-12627.8107	25591.6215 - 0.0000	25731.8289 - 0.0000	26322.8632 - 0.0000
SYM	-12644.2744	25620.5487 - 0.0000	25756.7748 - 0.0000	26343.0852 - 0.0000
TVMe	-12660.3856	25650.7712 - 0.0000	25785.0359 - 0.0000	26368.9551 - 0.0000
TPM3u	-12673.0593	25678.1186 - 0.0000	25814.3446 - 0.0000	26400.6550 - 0.0000
TPM3	-12673.0643	25678.1285 - 0.0000	25814.3546 - 0.0000	26400.6650 - 0.0000
TIM3	-12670.0064	25674.0128 - 0.0000	25812.2197 - 0.0000	26400.9019 - 0.0000
TIM3e	-12687.5810	25703.1621 - 0.0000	25835.4848 - 0.0000	26416.9933 - 0.0000
TIM2e	-12741.6041	25811.2083 - 0.0000	25943.5310 - 0.0000	26525.0395 - 0.0000
TIM2	-12732.0851	25798.1701 - 0.0000	25936.3770 - 0.0000	26525.0592 - 0.0000
TPM2	-12751.3422	25834.6845 - 0.0000	25970.9105 - 0.0000	26557.2210 - 0.0000
TPM2u	-12751.3450	25834.6900 - 0.0000	25970.9160 - 0.0000	26557.2264 - 0.0000
TIMe	-12763.0300	25854.0600 - 0.0000	25986.3828 - 0.0000	26567.8912 - 0.0000
TIM	-12760.0418	25854.0835 - 0.0000	25992.2904 - 0.0000	26580.9726 - 0.0000
K3P	-12778.2931	25882.5862 - 0.0000	26012.9862 - 0.0000	26592.0647 - 0.0000
K3Pu	-12772.1009	25876.2019 - 0.0000	26012.4279 - 0.0000	26598.7384 - 0.0000
TNe	-12782.2717	25890.5434 - 0.0000	26020.9434 - 0.0000	26600.0220 - 0.0000
TN	-12776.6329	25885.2657 - 0.0000	26021.4918 - 0.0000	26607.8022 - 0.0000
K2P	-12797.5754	25919.1508 - 0.0000	26047.6471 - 0.0000	26624.2767 - 0.0000
HKY	-12788.6981	25907.3961 - 0.0000	26041.6608 - 0.0000	26625.5800 - 0.0000
JC	-13020.3052	26362.6104 - 0.0000	26489.2221 - 0.0000	27063.3838 - 0.0000
F81	-13019.2185	26366.4370 - 0.0000	26498.7598 - 0.0000	27080.2682 - 0.0000

SUBSTITUTION PROCESS

Model of substitution: TPM2u+F+I+G4

Rate parameter R:

A-C: 1.1236
A-G: 3.6249
A-T: 1.1236
C-G: 1.0000
C-T: 3.6249
G-T: 1.0000

State frequencies: (empirical counts from alignment)

pi(A) = 0.2045
pi(C) = 0.2845
pi(G) = 0.3258
pi(T) = 0.1852

Rate matrix Q:

A	-1.232	0.2305	0.8516	0.15
C	0.1657	-0.8846	0.2349	0.4839
G	0.5346	0.2051	-0.8732	0.1335
T	0.1657	0.7435	0.2349	-1.144

Model of rate heterogeneity: Invar+Gamma with 4 categories
Proportion of invariable sites: 0.1458
Gamma shape alpha: 0.6881

Category	Relative_rate	Proportion
0	0	0.1458
1	0.08387	0.2136
2	0.4189	0.2136
3	1.077	0.2136
4	3.102	0.2136

Relative rates are computed as MEAN of the portion of the Gamma distribution falling in the category.

D Irs4

Best-fit model according to BIC: TIM3+I+G4

List of models sorted by BIC scores:

Model	LogL	AIC	w-AIC	AICc	w-AICc	BIC	w-BIC
TIM3+I+G4	-21104.9047	42583.8094	+ 0.5828	42677.5587	+ 0.7948	43489.5906	+ 0.8420
TPM3u+I+G4	-21110.7285	42593.4570	- 0.0047	42686.0855	- 0.0112	43494.3944	+ 0.0762
TPM3+I+G4	-21110.7581	42593.5162	- 0.0045	42686.1447	- 0.0109	43494.4537	+ 0.0740
GTR+I+G4	-21103.2915	42584.5830	+ 0.3959	42680.5990	+ 0.1738	43500.0517	- 0.0045
TIM3e+I+G4	-21121.3867	42610.7734	- 0.0000	42701.1851	- 0.0000	43502.0234	- 0.0017
TVM+I+G4	-21107.7813	42591.5626	- 0.0121	42686.4411	- 0.0094	43502.1876	- 0.0015
SYM+I+G4	-21121.3255	42614.6511	- 0.0000	42707.2796	- 0.0000	43515.5886	- 0.0000
TN+I+G4	-21122.1052	42616.2103	- 0.0000	42708.8388	- 0.0000	43517.1478	- 0.0000
TIM2+I+G4	-21120.0751	42614.1502	- 0.0000	42707.8995	- 0.0000	43519.9314	- 0.0000
TIM3+G4	-21123.5335	42619.0670	- 0.0000	42711.6955	- 0.0000	43520.0045	- 0.0000
TIM+I+G4	-21122.0272	42618.0543	- 0.0000	42711.8036	- 0.0000	43523.8356	- 0.0000
TPM3+G4	-21129.2733	42628.5466	- 0.0000	42720.0625	- 0.0000	43524.6403	- 0.0000
TPM3u+G4	-21129.3145	42628.6291	- 0.0000	42720.1450	- 0.0000	43524.7228	- 0.0000
TPM2u+I+G4	-21126.7353	42625.4707	- 0.0000	42718.0992	- 0.0000	43526.4082	- 0.0000
TPM2+I+G4	-21126.7799	42625.5597	- 0.0000	42718.1882	- 0.0000	43526.4972	- 0.0000
HKY+I+G4	-21130.6459	42631.2918	- 0.0000	42722.8078	- 0.0000	43527.3856	- 0.0000
TIM3e+G4	-21138.6504	42643.3008	- 0.0000	42732.6164	- 0.0000	43529.7070	- 0.0000
TVM+G4	-21125.0247	42624.0493	- 0.0000	42717.7987	- 0.0000	43529.8306	- 0.0000
GTR+G4	-21122.4763	42620.9526	- 0.0000	42715.8311	- 0.0000	43531.5776	- 0.0000
K3Pu+I+G4	-21130.5480	42633.0960	- 0.0000	42725.7245	- 0.0000	43534.0335	- 0.0000
SYM+G4	-21138.6178	42647.2356	- 0.0000	42738.7516	- 0.0000	43543.3294	- 0.0000
TVMe+I+G4	-21138.6648	42647.3295	- 0.0000	42738.8455	- 0.0000	43543.4233	- 0.0000
TN+G4	-21142.8146	42655.6292	- 0.0000	42747.1452	- 0.0000	43551.7230	- 0.0000
TIM2+G4	-21139.9244	42651.8488	- 0.0000	42744.4773	- 0.0000	43552.7863	- 0.0000
TPM2+G4	-21145.0208	42660.0417	- 0.0000	42751.5576	- 0.0000	43556.1354	- 0.0000
TPM2u+G4	-21145.0600	42660.1200	- 0.0000	42751.6360	- 0.0000	43556.2137	- 0.0000
TIM+G4	-21142.4987	42656.9975	- 0.0000	42749.6260	- 0.0000	43557.9350	- 0.0000
HKY+G4	-21150.5016	42669.0032	- 0.0000	42759.4149	- 0.0000	43560.2532	- 0.0000
K3Pu+G4	-21150.2423	42670.4845	- 0.0000	42762.0005	- 0.0000	43566.5783	- 0.0000
TVMe+G4	-21156.6591	42681.3183	- 0.0000	42771.7299	- 0.0000	43572.5682	- 0.0000
TNe+I+G4	-21169.7111	42705.4221	- 0.0000	42794.7378	- 0.0000	43591.8284	- 0.0000
TIMe+I+G4	-21169.0738	42706.1476	- 0.0000	42796.5593	- 0.0000	43597.3976	- 0.0000
TIM2e+I+G4	-21169.6614	42707.3229	- 0.0000	42797.7346	- 0.0000	43598.5729	- 0.0000
K2P+I+G4	-21184.4535	42732.9069	- 0.0000	42821.1347	- 0.0000	43614.4694	- 0.0000
K3P+I+G4	-21183.8357	42733.6713	- 0.0000	42822.9870	- 0.0000	43620.0775	- 0.0000
TNe+G4	-21187.7163	42739.4327	- 0.0000	42827.6605	- 0.0000	43620.9952	- 0.0000
TIMe+G4	-21187.0838	42740.1676	- 0.0000	42829.4832	- 0.0000	43626.5738	- 0.0000
TIM2e+G4	-21187.6151	42741.2301	- 0.0000	42830.5458	- 0.0000	43627.6364	- 0.0000
K2P+G4	-21202.9679	42767.9358	- 0.0000	42855.0840	- 0.0000	43644.6546	- 0.0000
K3P+G4	-21202.4192	42768.8384	- 0.0000	42857.0662	- 0.0000	43650.4009	- 0.0000
F81+I+G4	-21906.4755	44180.9510	- 0.0000	44271.3627	- 0.0000	45072.2010	- 0.0000
F81+G4	-21925.6343	44217.2686	- 0.0000	44306.5843	- 0.0000	45103.6749	- 0.0000
JC+I+G4	-21934.4505	44230.9010	- 0.0000	44318.0491	- 0.0000	45107.6197	- 0.0000
JC+G4	-21952.3877	44264.7754	- 0.0000	44350.8520	- 0.0000	45136.6504	- 0.0000
GTR+I	-21964.8989	44305.7978	- 0.0000	44400.6763	- 0.0000	45216.4228	- 0.0000
SYM+I	-22003.2482	44376.4963	- 0.0000	44468.0123	- 0.0000	45272.5901	- 0.0000
TIM3+I	-22001.3403	44374.6807	- 0.0000	44467.3092	- 0.0000	45275.6182	- 0.0000
TIM3e+I	-22016.2970	44398.5940	- 0.0000	44487.9096	- 0.0000	45285.0002	- 0.0000
TVM+I	-22004.9399	44383.8799	- 0.0000	44477.6292	- 0.0000	45289.6611	- 0.0000
TIM2+I	-22015.5735	44403.1471	- 0.0000	44495.7756	- 0.0000	45304.0846	- 0.0000
TPM3u+I	-22035.7685	44441.5371	- 0.0000	44533.0530	- 0.0000	45337.6308	- 0.0000
TPM3+I	-22035.7725	44441.5451	- 0.0000	44533.0610	- 0.0000	45337.6388	- 0.0000
TN+I	-22050.9787	44471.9573	- 0.0000	44563.4733	- 0.0000	45368.0510	- 0.0000
TIM+I	-22047.7143	44467.4286	- 0.0000	44560.0571	- 0.0000	45368.3661	- 0.0000
TVMe+I	-22055.0205	44478.0410	- 0.0000	44568.4527	- 0.0000	45369.2910	- 0.0000
TIM2e+I	-22072.0220	44510.0440	- 0.0000	44599.3596	- 0.0000	45396.4502	- 0.0000
TPM2+I	-22073.0261	44516.0522	- 0.0000	44607.5681	- 0.0000	45412.1459	- 0.0000
TPM2u+I	-22073.0269	44516.0539	- 0.0000	44607.5698	- 0.0000	45412.1476	- 0.0000
TNe+I	-22084.6613	44533.3227	- 0.0000	44621.5505	- 0.0000	45414.8852	- 0.0000
TIMe+I	-22082.7131	44531.4262	- 0.0000	44620.7418	- 0.0000	45417.8324	- 0.0000
HKY+I	-22100.6647	44569.3294	- 0.0000	44659.7411	- 0.0000	45460.5794	- 0.0000
K3Pu+I	-22097.6869	44565.3737	- 0.0000	44656.8897	- 0.0000	45461.4675	- 0.0000
K2P+I	-22136.2307	44634.4614	- 0.0000	44721.6095	- 0.0000	45511.1801	- 0.0000

K3P+I	-22134.2769	44632.5538 - 0.0000	44720.7817 - 0.0000	45514.1163 - 0.0000
F81+I	-22812.5006	45991.0012 - 0.0000	46080.3168 - 0.0000	46877.4074 - 0.0000
JC+I	-22834.7669	46029.5338 - 0.0000	46115.6104 - 0.0000	46901.4088 - 0.0000
GTR	-22850.1749	46074.3497 - 0.0000	46168.0991 - 0.0000	46980.1310 - 0.0000
TIM3	-22884.4119	46138.8238 - 0.0000	46230.3397 - 0.0000	47034.9175 - 0.0000
TVM	-22890.7385	46153.4770 - 0.0000	46246.1055 - 0.0000	47054.4145 - 0.0000
TPM3u	-22919.4192	46206.8384 - 0.0000	46297.2501 - 0.0000	47098.0884 - 0.0000
TPM3	-22919.5024	46207.0047 - 0.0000	46297.4164 - 0.0000	47098.2547 - 0.0000
SYM	-22927.2017	46222.4033 - 0.0000	46312.8150 - 0.0000	47113.6533 - 0.0000
TIM3e	-22938.2656	46240.5312 - 0.0000	46328.7590 - 0.0000	47122.0937 - 0.0000
TVMe	-22989.5995	46345.1991 - 0.0000	46434.5147 - 0.0000	47231.6053 - 0.0000
TIM2	-23006.8977	46383.7955 - 0.0000	46475.3114 - 0.0000	47279.8892 - 0.0000
TN	-23038.6443	46445.2886 - 0.0000	46535.7003 - 0.0000	47336.5386 - 0.0000
TIM	-23035.7416	46441.4832 - 0.0000	46532.9992 - 0.0000	47337.5769 - 0.0000
TIM2e	-23048.2626	46460.5252 - 0.0000	46548.7530 - 0.0000	47342.0876 - 0.0000
TNe	-23059.0061	46480.0122 - 0.0000	46567.1603 - 0.0000	47356.7309 - 0.0000
TIMe	-23056.7850	46477.5700 - 0.0000	46565.7978 - 0.0000	47359.1325 - 0.0000
TPM2	-23069.4687	46506.9374 - 0.0000	46597.3491 - 0.0000	47398.1874 - 0.0000
TPM2u	-23069.4849	46506.9699 - 0.0000	46597.3816 - 0.0000	47398.2199 - 0.0000
HKY	-23094.2596	46554.5192 - 0.0000	46643.8348 - 0.0000	47440.9254 - 0.0000
K3Pu	-23091.3800	46550.7600 - 0.0000	46641.1716 - 0.0000	47442.0099 - 0.0000
K2P	-23119.3710	46598.7421 - 0.0000	46684.8187 - 0.0000	47470.6170 - 0.0000
K3P	-23117.1354	46596.2707 - 0.0000	46683.4188 - 0.0000	47472.9894 - 0.0000
F81	-23775.8679	47915.7358 - 0.0000	48003.9636 - 0.0000	48797.2983 - 0.0000
JC	-23799.8716	47957.7431 - 0.0000	48042.7563 - 0.0000	48824.7744 - 0.0000

SUBSTITUTION PROCESS

Model of substitution: TIM3+F+I+G4

Rate parameter R:

A-C: 1.6638
A-G: 4.3668
A-T: 1.0000
C-G: 1.6638
C-T: 6.7858
G-T: 1.0000

State frequencies: (empirical counts from alignment)

pi(A) = 0.2427
pi(C) = 0.2583
pi(G) = 0.2805
pi(T) = 0.2185

Rate matrix Q:

A	-0.9186	0.2108	0.6007	0.1072
C	0.1981	-1.154	0.2289	0.7272
G	0.5199	0.2108	-0.8378	0.1072
T	0.1191	0.8597	0.1376	-1.116

Model of rate heterogeneity: Invar+Gamma with 4 categories
Proportion of invariable sites: 0.1648
Gamma shape alpha: 0.8079

Category	Relative_rate	Proportion
0	0	0.1648
1	0.1164	0.2088
2	0.4913	0.2088
3	1.148	0.2088
4	3.033	0.2088

Relative rates are computed as MEAN of the portion of the Gamma distribution falling in the category.

E Irs paralogs

Best-fit model according to BIC: TIM2+I+G4

List of models sorted by BIC scores:

Model	LogL	AIC	w-AIC	AICc	w-AICc	BIC	w-BIC
TIM2+I+G4	-16079.3675	32384.7350	+ 0.7800	32442.7620	+ 0.9193	32873.3875	+ 0.9962
GTR+I+G4	-16078.6334	32387.2668	+ 0.2200	32447.6288	+ 0.0807	32884.5681	- 0.0037
TN+I+G4	-16092.7977	32409.5955	- 0.0000	32466.4764	- 0.0000	32893.9237	- 0.0000
TIM+I+G4	-16091.1465	32408.2930	- 0.0000	32466.3201	- 0.0000	32896.9456	- 0.0000
TIM3+I+G4	-16091.6646	32409.3291	- 0.0000	32467.3562	- 0.0000	32897.9817	- 0.0000
TPM2+I+G4	-16097.7445	32419.4890	- 0.0000	32476.3699	- 0.0000	32903.8172	- 0.0000
TPM2u+I+G4	-16098.3768	32420.7537	- 0.0000	32477.6346	- 0.0000	32905.0819	- 0.0000
TIM2+G4	-16101.5357	32427.0714	- 0.0000	32483.9523	- 0.0000	32911.3996	- 0.0000
TVM+I+G4	-16097.3660	32422.7320	- 0.0000	32481.9193	- 0.0000	32915.7089	- 0.0000
GTR+G4	-16099.3216	32426.6433	- 0.0000	32485.8306	- 0.0000	32919.6202	- 0.0000
TN+G4	-16115.6483	32453.2966	- 0.0000	32509.0454	- 0.0000	32933.3004	- 0.0000
TIM+G4	-16113.4045	32450.8091	- 0.0000	32507.6900	- 0.0000	32935.1373	- 0.0000
TPM2+G4	-16116.7598	32455.5196	- 0.0000	32511.2685	- 0.0000	32935.5235	- 0.0000
TPM2u+G4	-16116.7983	32455.5967	- 0.0000	32511.3456	- 0.0000	32935.6005	- 0.0000
TIM3+G4	-16115.5361	32455.0723	- 0.0000	32511.9532	- 0.0000	32939.4005	- 0.0000
TPM3u+I+G4	-16119.4997	32462.9994	- 0.0000	32519.8803	- 0.0000	32947.3276	- 0.0000
TPM3+I+G4	-16119.8312	32463.6625	- 0.0000	32520.5434	- 0.0000	32947.9907	- 0.0000
TVM+G4	-16116.6825	32459.3650	- 0.0000	32517.3921	- 0.0000	32948.0176	- 0.0000
HKY+I+G4	-16123.4228	32468.8457	- 0.0000	32524.5946	- 0.0000	32948.8495	- 0.0000
K3Pu+I+G4	-16122.0571	32468.1141	- 0.0000	32524.9950	- 0.0000	32952.4423	- 0.0000
TPM3+G4	-16142.5004	32507.0007	- 0.0000	32562.7496	- 0.0000	32987.0045	- 0.0000
TPM3u+G4	-16142.5675	32507.1350	- 0.0000	32562.8838	- 0.0000	32987.1388	- 0.0000
HKY+G4	-16146.8416	32513.6833	- 0.0000	32568.3141	- 0.0000	32989.3627	- 0.0000
TIM3e+I+G4	-16148.0472	32516.0944	- 0.0000	32570.7252	- 0.0000	32991.7738	- 0.0000
K3Pu+G4	-16145.0067	32512.0134	- 0.0000	32567.7623	- 0.0000	32992.0172	- 0.0000
SYM+I+G4	-16144.4130	32512.8261	- 0.0000	32569.7070	- 0.0000	32997.1543	- 0.0000
TIM3e+G4	-16170.4260	32558.8520	- 0.0000	32612.3788	- 0.0000	33030.2072	- 0.0000
SYM+G4	-16167.0233	32556.0466	- 0.0000	32611.7955	- 0.0000	33036.0505	- 0.0000
TVMe+I+G4	-16168.6524	32559.3047	- 0.0000	32615.0536	- 0.0000	33039.3086	- 0.0000
TIMe+I+G4	-16188.2595	32596.5190	- 0.0000	32651.1499	- 0.0000	33072.1985	- 0.0000
TVMe+G4	-16189.9464	32599.8929	- 0.0000	32654.5237	- 0.0000	33075.5723	- 0.0000
TNe+I+G4	-16194.5266	32607.0533	- 0.0000	32660.5801	- 0.0000	33078.4084	- 0.0000
TIM2e+I+G4	-16194.4322	32608.8645	- 0.0000	32663.4954	- 0.0000	33084.5440	- 0.0000
K3P+I+G4	-16207.6655	32633.3310	- 0.0000	32686.8578	- 0.0000	33104.6861	- 0.0000
K2P+I+G4	-16214.3612	32644.7224	- 0.0000	32697.1589	- 0.0000	33111.7531	- 0.0000
TIMe+G4	-16211.7306	32641.4612	- 0.0000	32694.9880	- 0.0000	33112.8163	- 0.0000
TNe+G4	-16217.5779	32651.1558	- 0.0000	32703.5923	- 0.0000	33118.1865	- 0.0000
TIM2e+G4	-16217.5415	32653.0830	- 0.0000	32706.6098	- 0.0000	33124.4381	- 0.0000
K3P+G4	-16230.8343	32677.6687	- 0.0000	32730.1052	- 0.0000	33144.6994	- 0.0000
K2P+G4	-16237.0727	32688.1455	- 0.0000	32739.5055	- 0.0000	33150.8519	- 0.0000
F81+I+G4	-16604.7409	33429.4818	- 0.0000	33484.1126	- 0.0000	33905.1613	- 0.0000
JC+I+G4	-16614.4367	33442.8734	- 0.0000	33494.2334	- 0.0000	33905.5798	- 0.0000
F81+G4	-16627.8107	33473.6215	- 0.0000	33527.1483	- 0.0000	33944.9766	- 0.0000
JC+G4	-16637.3639	33486.7279	- 0.0000	33537.0250	- 0.0000	33945.1099	- 0.0000
GTR+I	-16860.0023	33948.0047	- 0.0000	34007.1920	- 0.0000	34440.9816	- 0.0000
TVM+I	-16882.2133	33990.4265	- 0.0000	34048.4535	- 0.0000	34479.0791	- 0.0000
SYM+I	-16938.0403	34098.0805	- 0.0000	34153.8294	- 0.0000	34578.0844	- 0.0000
TIM2+I	-16946.7898	34117.5796	- 0.0000	34174.4605	- 0.0000	34601.9078	- 0.0000
TIM3+I	-16961.3188	34146.6377	- 0.0000	34203.5186	- 0.0000	34630.9659	- 0.0000
TVMe+I	-16976.5817	34173.1634	- 0.0000	34227.7943	- 0.0000	34648.8429	- 0.0000
TIM3e+I	-16983.3542	34184.7084	- 0.0000	34238.2352	- 0.0000	34656.0635	- 0.0000
TPM3+I	-16977.4043	34176.8087	- 0.0000	34232.5575	- 0.0000	34656.8125	- 0.0000
TPM3u+I	-16977.4174	34176.8348	- 0.0000	34232.5837	- 0.0000	34656.8386	- 0.0000
TPM2+I	-17006.8148	34235.6295	- 0.0000	34291.3784	- 0.0000	34715.6333	- 0.0000
TPM2u+I	-17006.8148	34235.6296	- 0.0000	34291.3784	- 0.0000	34715.6334	- 0.0000
TIM+I	-17015.3520	34254.7040	- 0.0000	34311.5849	- 0.0000	34739.0322	- 0.0000
TIM2e+I	-17029.4288	34276.8576	- 0.0000	34330.3844	- 0.0000	34748.2127	- 0.0000
TN+I	-17036.3112	34294.6225	- 0.0000	34350.3713	- 0.0000	34774.6263	- 0.0000
TIMe+I	-17058.7810	34335.5621	- 0.0000	34389.0889	- 0.0000	34806.9172	- 0.0000
TNe+I	-17073.1605	34362.3211	- 0.0000	34414.7576	- 0.0000	34829.3519	- 0.0000
K3Pu+I	-17065.0601	34352.1203	- 0.0000	34407.8691	- 0.0000	34832.1241	- 0.0000
HKY+I	-17083.9665	34387.9330	- 0.0000	34442.5639	- 0.0000	34863.6125	- 0.0000
K3P+I	-17097.3042	34410.6084	- 0.0000	34463.0449	- 0.0000	34877.6391	- 0.0000

K2P+I	-17111.6370	34437.2739 - 0.0000	34488.6339 - 0.0000	34899.9803 - 0.0000
F81+I	-17445.3305	35108.6610 - 0.0000	35162.1877 - 0.0000	35580.0161 - 0.0000
JC+I	-17463.7919	35139.5837 - 0.0000	35189.8808 - 0.0000	35597.9658 - 0.0000
GTR	-17503.1970	35232.3941 - 0.0000	35290.4211 - 0.0000	35721.0467 - 0.0000
TVM	-17512.9014	35249.8028 - 0.0000	35306.6837 - 0.0000	35734.1310 - 0.0000
TIM3	-17569.3058	35360.6116 - 0.0000	35416.3605 - 0.0000	35840.6155 - 0.0000
TPM3u	-17574.6743	35369.3486 - 0.0000	35423.9795 - 0.0000	35845.0281 - 0.0000
TPM3	-17574.6759	35369.3517 - 0.0000	35423.9826 - 0.0000	35845.0312 - 0.0000
SYM	-17661.1555	35542.3110 - 0.0000	35596.9419 - 0.0000	36017.9905 - 0.0000
TIM3e	-17687.2348	35590.4695 - 0.0000	35642.9060 - 0.0000	36057.5003 - 0.0000
TVMe	-17695.2827	35608.5653 - 0.0000	35662.0921 - 0.0000	36079.9205 - 0.0000
TIM2	-17789.2906	35800.5812 - 0.0000	35856.3301 - 0.0000	36280.5851 - 0.0000
TIM2e	-17834.8084	35885.6168 - 0.0000	35938.0533 - 0.0000	36352.6476 - 0.0000
TIM	-17826.7758	35875.5515 - 0.0000	35931.3004 - 0.0000	36355.5554 - 0.0000
TPM2	-17831.7862	35883.5724 - 0.0000	35938.2033 - 0.0000	36359.2519 - 0.0000
TPM2u	-17831.7868	35883.5736 - 0.0000	35938.2045 - 0.0000	36359.2531 - 0.0000
TIMe	-17846.0274	35908.0549 - 0.0000	35960.4914 - 0.0000	36375.0857 - 0.0000
TN	-17842.6723	35905.3446 - 0.0000	35959.9755 - 0.0000	36381.0241 - 0.0000
TNe	-17859.6745	35933.3490 - 0.0000	35984.7090 - 0.0000	36396.0555 - 0.0000
K3Pu	-17860.6241	35941.2482 - 0.0000	35995.8791 - 0.0000	36416.9277 - 0.0000
K3P	-17876.0623	35966.1246 - 0.0000	36017.4846 - 0.0000	36428.8310 - 0.0000
HKY	-17875.9701	35969.9403 - 0.0000	36023.4670 - 0.0000	36441.2954 - 0.0000
K2P	-17889.6522	35991.3044 - 0.0000	36041.6016 - 0.0000	36449.6865 - 0.0000
F81	-18211.6533	36639.3067 - 0.0000	36691.7432 - 0.0000	37106.3375 - 0.0000
JC	-18229.1293	36668.2585 - 0.0000	36717.5063 - 0.0000	37122.3162 - 0.0000

SUBSTITUTION PROCESS

Model of substitution: TIM2+F+I+G4

Rate parameter R:

A-C: 1.0410
A-G: 2.3035
A-T: 1.0410
C-G: 1.0000
C-T: 5.1647
G-T: 1.0000

State frequencies: (empirical counts from alignment)

pi(A) = 0.2555
pi(C) = 0.2427
pi(G) = 0.2937
pi(T) = 0.2081

Rate matrix Q:

A	-0.8352	0.1842	0.4931	0.1579
C	0.1939	-1.191	0.2141	0.7832
G	0.429	0.1769	-0.7576	0.1516
T	0.1939	0.9138	0.2141	-1.322

Model of rate heterogeneity: Invar+Gamma with 4 categories
Proportion of invariable sites: 0.1461
Gamma shape alpha: 0.7185

Category	Relative_rate	Proportion
0	0	0.1461
1	0.09151	0.2135
2	0.4358	0.2135
3	1.091	0.2135
4	3.066	0.2135

Relative rates are computed as MEAN of the portion of the Gamma distribution falling in the category.

Additional file 13: Supplementary Figure 9. Results of *ModelFinder* for (A) *Irs1*, (B) *Irs2*, (C) *Irs3*, (D) *Irs4*, and (E) family of *Irs* paralogs. *ModelFinder* results used for constructing phylogenetic trees by Bayesian and Maximum likelihood methods presented in Figure 1 and in Additional files 4-8: Supplementary Figures 2-6. AIC, w-AIC: Akaike information criterion scores and weights. AICc, w-AICc: Corrected AIC scores and weights. BIC, w-BIC: Bayesian information criterion scores and weights. Plus signs denote the 95% confidence sets. Minus signs denote significant exclusion.
