# Supplementary material for: Evolution of the vertebrate insulin receptor substrate (Irs) gene family
Source: BMC Evol Biol. 2017 Jun 23;17:148. doi: 10.1186/s12862-017-0994-z (PMC5482937; doi:10.1186/s12862-017-0994-z)
Supplement: Supplementary file 7 — This file is in PDF format. Phylogeny of vertebrate Irs4 sequences. (DOCX 627 kb) [file 12862_2017_994_MOESM3_ESM.docx]

>Hom_sa1 [Human (Homo sapiens) IRS1]

ATGGCGAGCCCTCCGGAGAGCGATGGCTTCTCGGACGTGCGCAAGGTGGGCTACCTGCGCAAACCCAAGAGCATGCACAAACGCTTCTTCGTACTGCGCGCGGCCAGCGAGGCTGGGGGCCCGGCGCGCCTCGAGTACTACGAGAACGAGAAGAAGTGGCGGCACAAGTCGAGCGCCCCCAAACGCTCGATCCCCCTTGAGAGCTGCTTCAACATCAACAAGCGGGCTGACTCCAAGAACAAGCACCTGGTGGCTCTCTACACCCGGGACGAGCACTTTGCCATCGCGGCGGACAGCGAGGCCGAGCAAGACAGCTGGTACCAGGCTCTCCTACAGCTGCACAACCGTGCTAAGGGCCACCACGACGGAGCTGCGGCCCTCGGGGCGGGAGGTGGTGGGGGCAGCTGCAGCGGCAGCTCCGGCCTTGGTGAGGCTGGGGAGGACTTGAGCTACGGTGACGTGCCCCCAGGACCCGCATTCAAAGAGGTCTGGCAAGTGATCCTGAAGCCCAAGGGCCTGGGTCAGACAAAGAACCTGATTGGTATCTACCGCCTTTGCCTGACCAGCAAGACCATCAGCTTCGTGAAGCTGAACTCGGAGGCAGCGGCCGTGGTGCTGCAGCTGATGAACATCAGGCGCTGTGGCCACTCGGAAAACTTCTTCTTCATCGAGGTGGGCCGTTCTGCCGTGACGGGGCCCGGGGAGTTCTGGATGCAGGTGGATGACTCTGTGGTGGCCCAGAACATGCACGAGACCATCCTGGAGGCCATGCGGGCCATGAGTGATGAGTTCCGCCCTCGCAGCAAGAGCCAGTCCTCGTCCAACTGCTCTAACCCCATCAGCGTCCCCCTGCGCCGGCACCATCTCAACAATCCCCCGCCCAGCCAGGTGGGGCTGACCCGCCGATCACGCACTGAGAGCATCACCGCCACCTCCCCGGCCAGCATGGTGGGCGGGAAGCCAGGCTCCTTCCGTGTCCGCGCCTCCAGTGACGGCGAAGGCACCATGTCCCGCCCAGCCTCGGTGGACGGCAGCCCTGTGAGTCCCAGCACCAACAGAACCCACGCCCACCGGCATCGGGGCAGCGCCCGGCTGCACCCCCCGCTCAACCACAGCCGCTCCATCCCCATGCCGGCTTCCCGCTGCTCGCCTTCGGCCACCAGCCCGGTCAGTCTGTCGTCCAGTAGCACCAGTGGCCATGGCTCCACCTCGGATTGTCTCTTCCCACGGCGATCTAGTGCTTCGGTGTCTGGTTCCCCCAGCGATGGCGGTTTCATCTCCTCGGATGAGTATGGCTCCAGTCCCTGCGATTTCCGGAGTTCCTTCCGCAGTGTCACTCCGGATTCCCTGGGCCACACCCCACCAGCCCGCGGTGAGGAGGAGCTAAGCAACTATATCTGCATGGGTGGCAAGGGGCCCTCCACCCTGACCGCCCCCAACGGTCACTACATTTTGTCTCGGGGTGGCAATGGCCACCGCTGCACCCCAGGAACAGGCTTGGGCACGAGTCCAGCCTTGGCTGGGGATGAAGCAGCCAGTGCTGCAGATCTGGATAATCGGTTCCGAAAGAGAACTCACTCGGCAGGCACATCCCCTACCATTACCCACCAGAAGACCCCGTCCCAGTCCTCAGTGGCTTCCATTGAGGAGTACACAGAGATGATGCCTGCCTACCCACCAGGAGGTGGCAGTGGAGGCCGACTGCCGGGACACAGGCACTCCGCCTTCGTGCCCACCCGCTCCTACCCAGAGGAGGGTCTGGAAATGCACCCCTTGGAGCGTCGGGGGGGGCACCACCGCCCAGACAGCTCCACCCTCCACACGGATGATGGCTACATGCCCATGTCCCCAGGGGTGGCCCCAGTGCCCAGTGGCCGAAAGGGCAGTGGAGACTATATGCCCATGAGCCCCAAGAGCGTATCTGCCCCACAGCAGATCATCAATCCCATCAGACGCCATCCCCAGAGAGTGGACCCCAATGGCTACATGATGATGTCCCCCAGCGGTGGCTGCTCTCCTGACATTGGAGGTGGCCCCAGCAGCAGCAGCAGCAGCAGCAACGCCGTCCCTTCCGGGACCAGCTATGGAAAGCTGTGGACAAACGGGGTAGGGGGCCACCACTCTCATGTCTTGCCTCACCCCAAACCCCCAGTGGAGAGCAGCGGTGGTAAGCTCTTACCTTGCACAGGTGACTACATGAACATGTCACCAGTGGGGGACTCCAACACCAGCAGCCCCTCCGACTGCTACTACGGCCCTGAGGACCCCCAGCACAAGCCAGTCCTCTCCTACTACTCATTGCCAAGATCCTTTAAGCACACCCAGCGCCCCGGGGAGCCGGAGGAGGGTGCCCGGCATCAGCACCTCCGCCTTTCCACTAGCTCTGGTCGCCTTCTCTATGCTGCAACAGCAGATGATTCTTCCTCTTCCACCAGCAGCGACAGCCTGGGTGGGGGATACTGCGGGGCTAGGCTGGAGCCCAGCCTTCCACATCCCCACCATCAGGTTCTGCAGCCCCATCTGCCTCGAAAGGTGGACACAGCTGCTCAGACCAATAGCCGCCTGGCCCGGCCCACGAGGCTGTCCCTGGGGGATCCCAAGGCCAGCACCTTACCTCGGGCCCGAGAGCAGCAGCAGCAGCAGCAGCCCTTGCTGCACCCTCCAGAGCCCAAGAGCCCGGGGGAATATGTCAATATTGAATTTGGGAGTGATCAGTCTGGCTACTTGTCTGGCCCGGTGGCTTTCCACAGCTCACCTTCTGTCAGGTGTCCATCCCAGCTCCAGCCAGCTCCCAGAGAGGAAGAGACTGGCACTGAGGAGTACATGAAGATGGACCTGGGGCCGGGCCGGAGGGCAGCCTGGCAGGAGAGCACTGGGGTCGAGATGGGCAGACTGGGCCCTGCACCTCCCGGGGCTGCTAGCATTTGCAGGCCTACCCGGGCAGTGCCCAGCAGCCGGGGTGACTACATGACCATGCAGATGAGTTGTCCCCGTCAGAGCTACGTGGACACCTCGCCAGCTGCCCCTGTAAGCTATGCTGACATGCGAACAGGCATTGCTGCAGAGGAGGTGAGCCTGCCCAGGGCCACCATGGCTGCTGCCTCCTCATCCTCAGCAGCCTCTGCTTCCCCGACTGGGCCTCAAGGGGCAGCAGAGCTGGCTGCCCACTCGTCCCTGCTGGGGGGCCCACAAGGACCTGGGGGCATGAGCGCCTTCACCCGGGTGAACCTCAGTCCTAACCGCAACCAGAGTGCCAAAGTGATCCGTGCAGACCCACAAGGGTGCCGGCGGAGGCATAGCTCCGAGACTTTCTCCTCAACACCCAGTGCCACCCGGGTGGGCAACACAGTGCCCTTTGGAGCGGGGGCAGCAGTAGGGGGCGGTGGCGGTAGCAGCAGCAGCAGCGAGGATGTGAAACGCCACAGCTCTGCTTCCTTTGAGAATGTGTGGCTGAGGCCTGGGGAGCTTGGGGGAGCCCCCAAGGAGCCAGCCAAACTGTGTGGGGCTGCTGGGGGTTTGGAGAATGGTCTTAACTACATAGACCTGGATTTGGTCAAGGACTTCAAACAGTGCCCTCAGGAGTGCACCCCTGAACCGCAGCCTCCCCCACCCCCACCCCCTCATCAACCCCTGGGCAGCGGTGAGAGCAGCTCCACCCGCCGCTCAAGTGAGGATTTAAGCGCCTATGCCAGCATCAGTTTCCAGAAGCAGCCAGAGGACCGTCAGTAG

>Pan_tr1 [Chimpanzee (Pan troglodytes) Irs1]

ATGGCGAGCCCTCCGGAGAGCGATGGCTTCTCGGACGTGCGCAAGGTGGGCTACCTGCGCAAACCCAAGAGCATGCACAAACGCTTCTTCGTACTGCGCGCGGCCAGCGAGGCTGGGGGCCCGGCGCGCCTCGAGTACTACGAGAACGAGAAGAAGTGGCGGCACAAGTCGAGCGCCCCCAAACGCTCGATCCCCCTTGAGAGCTGCTTCAACATCAACAAGCGGGCTGACTCCAAGAACAAGCACCTGGTGGCTCTCTACACCCGGGACGAGCACTTTGCCATCGCGGCGGACAGCGAGGCCGAGCAAGACAGCTGGTACCAGGCTCTCCTACAGCTGCACAACCGTGCTAAGGGCCACCACGACGGAGCTGCGGCCCTCGGGGCGGGAGGTGGTGGGGGCAGCTGCAGCGGCAGCTCCGGCCTTGGTGAGGCTGGGGAGGACTTGAGCTACGGTGACGTGCCCCCAGGACCCGCATTCAAAGAGGTCTGGCAAGTGATCCTGAAGCCCAAGGGCCTGGGTCAGACAAAGAACCTGATTGGTATCTACCGCCTTTGCCTGACCAGCAAGACCATCAGCTTCGTGAAGCTGAACTCGGAGGCAGCGGCCGTGGTGCTGCAGCTGATGAACATCAGGCGCTGTGGCCACTCGGAAAACTTCTTCTTCATCGAGGTGGGCCGTTCTGCCGTGACGGGGCCCGGGGAGTTCTGGATGCAGGTGGATGACTCTGTGGTGGCCCAGAACATGCACGAGACCATCCTGGAGGCCATGCGGGCCATGAGTGATGAGTTCCGCCCTCGCAGCAAGAGCCAGTCCTCGTCCAACTGCTCTAACCCCATCAGCGTCCCCCTGCGCCGGCACCATCTCAACAATCCCCCGCCCAGCCAGGTGGGGCTGACCCGCCGATCGCGCACTGAGAGCATCACCGCCACCTCCCCGGCCAGCATGGTGGGCGGGAAGCCAGGCTCCTTCCGTGTCCGCGCCTCCAGTGACGGCGAAGGCACCATGTCCCGCCCAGCCTCGGTGGACGGCAGCCCTGTGAGTCCCAGCACCAACAGAACCCACGCCCACCGGCATCGGGGCAGCGCCCGGCTGCACCCCCCGCTCAACCACAGCCGCTCCATCCCCATGCCGGCTTCCCGCTGCTCGCCTTCGGCCACCAGCCCGGTCAGTCTGTCGTCCAGTAGCACCAGTGGCCATGGCTCCACCTCGGATTGTCTCTTCCCACGGCGATCTAGTGCTTCGGTGTCTGGTTCCCCCAGCGATGGCGGTTTCATCTCCTCGGATGAGTATGGCTCCAGTCCCTGCGATTTCCGGAGTTCCTTCCGCAGTGTCACTCCGGATTCCCTGGGCCACACCCCACCAGCCCGCGGTGAGGAGGAGCTAAGCAACTATATCTGCATGGGTGGTAAGGGGCCCTCCACCCTGACCGCCCCCAACGGTCACTACATTTTGTCTCGGGGTGGCAATGGCCACCGCTGCACCCCAGGAACAGGCTTGGGCACGAGTCCAGCCTTGGCTGGGGATGAAGCAGCCAGTGCTGCAGATCTGGATAATCGGTTCCGAAAGAGAACTCACTCGGCAGGCACATCCCCTACCATTAACCACCAGAAGACCCCGTCCCAGTCCTCAGTGGCTTCCATTGAGGAGTACACAGAGATGATGCCTGCCTACCCACCAGGAGGTGGCAGTGGAGGCCGACTGCCGGGACACAGGCACTCCGCCTTCGTGCCCACCCACTCCTACCCAGAGGAGGGTCTGGAAATGCACCCCTTGGAGCGTCGGGGGGGGCACCACCGCCCAGACAGCTCCACCCTCCACACGGATGATGGCTACATGCCCATGTCCCCAGGGGTGGCCCCAGTGCCCAGCGGCCGAAAGGGCAGTGGAGACTATATGCCCATGAGCCCCAAGAGCGTATCTGCCCCACAGCAGATCATCAATCCCATCAGACGCCATCCCCAGAGAGTGGACCCCAATGGCTACATGATGATGTCCCCCAGCGGCGGCTGCTCTCCTGACATTGGAGGTGGCCCCAGCAGCAGCAGCAGCAGCAATGCCGTCCCTTCCGGGACCAGCTATGGAAAGCTGTGGACAAACGGGGTAGGGGGCCACCACTCTCATGTCTTGCCTCACCCCAAACCCCCAGTGGAGAGCAGCGGTGGTAAGCTCTTACCTTGCACAGGTGACTACATGAACATGTCACCAGTGGGGGACTCCAACACCAGCAGCCCCTCCGACTGCTACTACGGCCCTGAGGACCCCCAGCACAAGCCAGTCCTCTCCTACTACTCATTGCCAAGATCCTTTAAGCACACCCAGCGCCCCGGGGAGCCGGAGGAGGGTGCCCGGCATCAGCACCTCCGCCTTTCCACTAGCTCTGGTCGCCTTCTCTATGCTGCAACAGCGGATGATTCTTCCTCTTCCACCAGCAGCGACAGCCTGGGTGGGGGATACTGCGGGGCTAGGCTGGAGCCCAGCCTTCCACATCCCCACCATCAGGTTCTGCAGCCCCATCTGCCTCGAAAGGTGGACACAGCTGCTCAGACCAATAGCCGCCTGGCCCGGCCCACGAGGCTGTCCCTGGGGGATCCCAAGGCCAGCACCTTACCTCGGGCCCGAGAGCAGCAGCAGCAGCAGCCCTTGCTGCACCCTCCAGAGCCCAAGAGCCCGGGGGAATATGTCAATATTGAATTTGGGAGTGATCAGCCTGGCTACTTGTCTGGCCCGGTGGCTTCCCACAGCTCACCTTCTGTCAGGTGTCCATCCCAGCTCCAGCCAGCTCCCAGAGAGGAAGAGACTGGCACTGAGGAGTACATGAAGATGGACCTGGGGCCGGGCCGGAGGGCAGCTTGGCAGGAGAGCACTGGGGTCGAGATGGGCAGACTGGGCCCTGCACCTCCCGGGGCTGCTAGCATTTGCAGGCCTACCCGGGCAGTGCCCAGCAGCCGGGGTGACTACATGACCATGCAGATGAGTTGTCCCCGTCAGAGCTACGTGGACACCTCGCCAGCTGCCCCTGTAAGCTATGCTGACATGCGGACAGGCATTGCTGCAGAGGAGGTGAGCCTGCCCAGGGCCACCATGGTTGCTGCCTCCTCATCCTCAGCAGTCTCTGCTTCCCCGACTGGGCCTCAAGGGGCAGCAGAGCTGGCTGCCCACTCGTCCCTGCTGGGGGGCCCACAAGGACCTGGGGGCATGAGCGCCTTCACCCGGGTGAACCTCAGTCCTAACCGCAACCAGAGTGCCAAAGTGATCCGTGCAGACCCACAAGGGTGCCGGCGGAGGCATAGCTCCGAGACTTTCTCCTCAACACCCAGTGCCACCCGGGTGGGCAACACAGTGCCCTTTGGAGCGGGGGCAACAGTAGGGGGCGGTGGCGGTAGCAGCAGCAGCAGCAGCGAGGATGTGAAACGCCACAGCTCTGCTTCCTTTGAGAATGTGTGGCTGAGGCCTGGGGAGCTTGGGGGAGCCCCCAAGGAGCCAGCCCAACTGTGTGGGGCTGCTGGGGGTTTGGAGAATGGTCTTAACTACATAGACCTGGATTTGGTCAAGGACTTCAAACAGTGCCCTCAGGAGTGCACCCCTGAACCGCAGCCTCCCCCACCCCCACCCCCTCATCAACCCCTGGGCAGCGGTGAGAGCAGCTCCACCCGCCGCTCAAGTGAGGATTTAAGCGCCTATGCCAGCATCAGTTTCCAGAAGCAGCCAGAGGACCGTCAGTAG

>Pan_pa1 [Pygmy chimpanzee (Pan paniscus) Irs1]

ATGGCGAGCCCTCCGGAGAGCGATGGCTTCTCGGACGTGCGCAAGGTGGGCTACCTGCGCAAACCCAAGAGCATGCACAAACGCTTCTTCGTACTGCGCGCGGCCAGCGAGGCTGGGGGCCCGGCGCGCCTCGAGTACTACGAGAACGAGAAGAAGTGGCGGCACAAGTCGAGCGCCCCCAAACGCTCGATCCCCCTTGAGAGCTGCTTCAACATCAACAAGCGGGCTGACTCCAAGAACAAGCACCTGGTGGCTCTCTACACCCGGGACGAGCACTTTGCCATCGCGGCGGACAGCGAGGCCGAGCAAGACAGCTGGTACCAGGCTCTCCTACAGCTGCACAACCGTGCTAAGGGCCACCACGACGGAGCTGCGGCCCTCGGGGCGGGAGGTGGTGGGGGCAGCTGCAGCGGCAGCTCCGGCCTTGGTGAGGCTGGGGAGGACTTGAGCTACGGTGACGTGCCCCCAGGACCCGCATTCAAAGAGGTCTGGCAAGTGATCCTGAAGCCCAAGGGCCTGGGTCAGACAAAGAACCTGATTGGTATCTACCGCCTTTGCCTGACCAGCAAGACCATCAGCTTCGTGAAGCTGAACTCGGAGGCAGCGGCCGTGGTGCTGCAGCTGATGAACATCAGGCGCTGTGGCCACTCGGAAAACTTCTTCTTCATCGAGGTGGGCCGTTCTGCCGTGACGGGGCCCGGGGAGTTCTGGATGCAGGTGGATGACTCTGTGGTGGCCCAGAACATGCACGAGACCATCCTGGAGGCCATGCGGGCCATGAGTGATGAGTTCCGCCCTCGCAGCAAGAGCCAGTCCTCGTCCAACTGCTCTAACCCCATCAGCGTCCCCCTGCGCCGGCACCATCTCAACAATCCCCCGCCCAGCCAGGTGGGGCTGACCCGCCGATCGCGCACTGAGAGCATCACCGCCACCTCCCCGGCCAGCATGGTGGGCGGGAAGCCAGGCTCCTTCCGTGTCCGCGCCTCCAGTGACGGCGAAGGCACCATGTCCCGCCCAGCCTCGGTGGACGGCAGCCCTGTGAGTCCCAGCACCAACAGAACCCACGCCCACCGGCATCGGGGCAGCGCCCGGCTGCACCCCCCGCTCAACCACAGCCGCTCCATCCCCATGCCGGCTTCCCGCTGCTCGCCTTCGGCCACCAGCCCGGTCAGTCTGTCGTCCAGTAGCACCAGTGGCCATGGCTCCACCTCGGATTGTCTCTTCCCACGGCGATCTAGTGCTTCGGTGTCTGGTTCCCCCAGCGATGGCGGTTTCATCTCCTCGGATGAGTATGGCTCCAGTCCCTGCGATTTCCGGAGTTCCTTCCGCAGTGTCACTCCGGATTCCCTGGGCCACACCCCACCAGCCCGCGGTGAGGAGGAGCTAAGCAACTATATCTGCATGGGTGGTAAGGGGCCCTCCACCCTGACCGCCCCCAACGGTCACTACATTTTGTCTCGGGGTGGCAATGGCCACCGCTGCACCCCAGGAACAGGCTTGGGCACGAGTCCAGCCTTGGCTGGGGATGAAGCAGCCAGTGCTGCAGATCTGGATAATCGGTTCCGAAAGAGAACTCACTCGGCAGGCACATCCCCTACCATTACCCACCAGAAGACCCCGTCCCAGTCCTCAGTGGCTTCCATTGAGGAGTACACAGAGATGATGCCTGCCTACCCACCAGGAGGTGGCAGTGGAGGCCGACTGCCGGGACACAGGCACTCCGCCTTCGTGCCCACCCACTCCTACCCAGAGGAGGGTCTGGAAATGCACCCCTTGGAGCGTCGGGGGGGGCACCACCGCCCAGACAGCTCCACCCTCCACACGGATGATGGCTACATGCCCATGTCCCCAGGGGTGGCCCCAGTGCCCAGCGGCCGAAAGGGCAGTGGAGACTATATGCCCATGAGCCCCAAGAGCGTATCTGCCCCACAGCAGATCATCAATCCCATCAGACGCCATCCCCAGAGAGTGGACCCCAATGGCTACATGATGATGTCCCCCAGCGGCGGCTGCTCTCCTGACATTGGAGGTGGCCCCAGCAGCAGCAGCAGCAGCAGCAATGCCGTCCCTTCCGGGACCAGCTATGGAAAGCTGTGGACAAACGGGGTAGGGGGCCACCACTCTCATGTCTTGCCTCACCCCAAACCCCCAGTGGAGAGCAGCGGTGGTAAGCTCTTACCTTGCACAGGTGACTACATGAACATGTCACCAGTGGGGGACTCCAACACCAGCAGCCCCTCCGACTGCTACTACGGCCCTGAGGACCCCCAGCACAAGCCAGTCCTCTCCTACTACTCATTGCCAAGATCCTTTAAGCACACCCAGCGCCCCGGGGAGCCGGAGGAGGGTGCCCGGCATCAGCACCTCCGCCTTTCCACTAGCTCTGGTCGCCTTCTCTATGCTGCAACAGCGGATGATTCTTCCTCTTCCACCAGCAGCGACAGCCTGGGTGGGGGATACTGCGGGGCTAGGCTGGAGCCCAGCCTTCCACATCCCCACCATCAGGTTCTGCAGCCCCATCTGCCTCGAAAGGTGGACACAGCTGCTCAGACCAATAGCCGCCTGGCCCGGCCCACGAGGCTGTCCCTGGGGGATCCCAAGGCCAGCACCTTACCTCGGGCCCGAGAGCAGCAGCAGCAGCAGCCCTTGCTGCACCCTCCAGAGCCCAAGAGCCCGGGGGAATATGTCAATATTGAATTTGGGAGTGATCAGCCTGGCTACTTGTCTGGCCCGGTGGCTTCCCACAGCTCACCTTCTGTCAGGTGTCCATCCCAGCTCCAGCCAGCTCCCAGAGAGGAAGAGACTGGCACTGAGGAGTACATGAAGATGGACCTGGGGCCGGGCCGGAGGGCAGCTTGGCAGGAGAGCACTGGGGTCGAGATGGGCAGACTGGGCCCTGTACCTCCCGGGGCTGCTAGCATTTGCAGGCCTACCCGGGCAGTGCCCAGCAGCCGGGGTGACTACATGACCATGCAGATGAGTTGTCCCCGTCAGAGCTATGTGGACACCTCGCCAGCTGCCCCTGTAAGCTATGCTGACATGCGGACAGGCATTGCTGCAGAGGAGGTGAGCCTGCCCAGGGCCACCATGGTTGCTGCCTCCTCATCCTCAGCAGTCTCTGCTTCCCCGACTGGGCCTCAAGGGGCAGCAGAGCTGGCTGCCCACTCGTCCCTGCTGGGGGGCCCACAAGGACCTGGGGGCATGAGCGCCTTCACCCGGGTGAACCTCAGTCCTAACCGCAACCAGAGTGCCAAAGTGATCCGTGCAGACCCACAAGGGTGCCGGCGGAGGCATAGCTCCGAGACTTTCTCCTCAACACCCAGTGCCACCCGGGTGGGCAACACAGTGCCCTTTGGAGCGGGGGCAACAGTAGGGGGCGGTGGCGGTAGCAGCAGCAGCAGCAGCGAGGATGTGAAACGCCACAGCTCTGCTTCCTTTGAGAATGTGTGGCTGAGGCCTGGGGAGCTTGGGGGAGCCCCCAAGGAGCCAGCCCAACTGTGTGGGGCTGCTGGGGGTTTGGAGAATGGTCTTAACTACATAGACCTGGATTTGGTCAAGGACTTCAAACAGTGCCCTCAGGAGTGCACCCCTGAACCGCAGCCTCCCCCACCCCCACCCCCTCATCAACCCCTGGGCAGCGGTGAGAGCAGCTCCACCCGCCGCTCAAGTGAGGATTTAAGCGCCTATGCCAGCATCAGTTTCCAGAAGCAGCCAGAGGACCGTCAGTAG

>Gor_go1 [Gorilla (Gorilla gorilla gorilla) Irs1]

ATGGCGAGCCCTCCGGAGAGCGATGGCTTCTCGGACGTGCGCAAGGTGGGCTACCTGCGCAAACCCAAGAGCATGCACAAACGCTTCTTCGTACTGCGCGCGGCCAGCGAGGCTGGGGGCCCGGCGCGCCTCGAGTACTACGAGAACGAGAAGAAGTGGCGGCACAAGTCGAGCGCCCCCAAACGCTCGATCCCCCTTGAGAGCTGCTTCAACATCAACAAGCGGGCTGACTCCAAGAACAAGCACCTGGTGGCTCTCTACACCCGGGACGAGCACTTTGCCATCGCGGCGGACAGCGAGGCCGAGCAAGACAGCTGGTACCAGGCTCTCCTACAGCTGCACAACCGTGCTAAGGGCCACCACGACGGAGCTGCGGCCCTCGGGGCGGGAGGTGGTGGGGGCAGCTGCAGCGGCAGCTCCGGCCTTGGTGAGGCTGGGGAGGACTTGAGCTACGGTGACGTGCCCCCAGGACCCGCATTCAAAGAGGTCTGGCAAGTGATCCTGAAGCCCAAGGGCCTGGGTCAGACAAAGAACCTGATTGGTATCTACCGCCTTTGCCTGACCAGCAAGACCATCAGCTTCGTGAAGCTGAACTCGGAGGCAGCGGCCGTGGTGCTGCAGCTGATGAACATCAGGCGCTGTGGCCACTCGGAAAACTTCTTCTTCATCGAGGTGGGCCGTTCTGCCGTGACGGGGCCCGGGGAATTCTGGATGCAGGTGGATGACTCCGTGGTGGCCCAGAACATGCACGAGACCATCCTGGAGGCCATGCGGGCCATGAGTGATGAGTTCCGCCCTCGCAGCAAGAGCCAGTCCTCGTCCAACTGCTCTAACCCCATCAGCGTCCCCCTGCGCCGGCACCATCTCAACAATCCCCCGCCCAGCCAGGTGGGGCTGACCCGCCGATCGCGCACTGAGAGCATCACCGCCACCTCCCCGGCCAGCATGGTGGGCGGGAAGCCAGGCTCCTTCCGTGTCCGCGCCTCCAGTGACGGCGAAGGCACCATGTCCCGCCCAGCCTCGGTGGACAGCAGCCCTGTGAGTCCCAGCACCAACAGAACCCACGCCCACCGGCATCGGGGCAGCGCCCGGCTGCACCCCCCGCTCAACCACAGCCGCTCCATCCCCATGCCGGCTTCCCGCTGCTCGCCTTCGGCCACCAGCCCGGTCAGTCTGTCGTCCAGTAGCACCAGTGGCCATGGCTCCACCTCGGATTGTCTCTTCCCACGGCGATCTAGTGCTTCGGTGTCTGGTTCCCCCAGCGATGGCGGTTTCATCTCCTCGGATGAGTACGGCTCCAGTCCCTGCGATTTCCGGAGTTCCTTCCGCAGTGTCACTCCGGATTCCCTGGGCCACACCCCACCAGCCCGCGGTGAGGAGGAGCTAAGCAACTATATCTGCATGGGTGGCAAGGGGCCCTCCACCCTGACCGCCCCCAACGGTCACTACATTTTGTCTCGGGGTGGCAATGGCCACCGCTACACCCCAGGAACAGGCTTGGGCACGAGTCCAGCCTTGGCTGGGGATGAAGCAGCCAGTGCTGCAGATCTGGATAATCGGTTCCGAAAGAGAACTCACTCGGCAGGCACATCCCCTACCATTACCCACCAGAAGACCCCGTCCCAGTCCTCAGTGGCTTCCATTGAGGAGTATACAGAGATGATGCCTGCCTACCCACCAGGAGGTGGCAGTGGAGGCCGACTGCCGGGACACAGGCACTCCGCCTTCGTGCCCACTCACTCCTACCCAGAGGAGGGTCTGGAAATGCACCCCTTGGAGCGTCGGGGGGGGCACCACCGCCCAGAGAGCTCCACCCTCCACACGGATGATGGCTACATGCCCATGTCCCCAGGGGTGGCTCCAGTGCCCAGCGGCCGAAAGGGCAGTGGAGACTATATGCCCATGAGCCCCAAGAGCGTGTCTGCCCCACAGCAGATCATCAATCCCATCAGACGCCATCCCCAGAGAGTGGACCCCAATGGCTACATGATGATGTCCCCCAGCGGCGGCTGCTCTCCTGACATTGGAGGTGGCCCCAGCAGCAGCAGCAGCAGCAGCAGCAGCAATGCCATCCCTTCTGGGACCAGCTATGGAAAGCTGTGGACAAACGGGGTAGGGGGTCACCACTCTCATGTCTTGCCTCACCCCAAACCCCCAGTGGAGAGCAGTGGTGGTAAGCTCTTACCTTGCACAAGTGACTACATGAACATGTCACCAGTGGGGGACTCCAACACCAGCAGCCCCTCCGACTGCTACTACGGCCCTGAGGACCCCCAGCACAAGCCAGTCCTCTCCTACTACTCATTACCAAGATCCTTTAAGCACACCCAGCGCCCCGGGGAGCCGGAGGAGGGTGCCCGGCATCAGCACCTCCGCCTTTCCACTAGCTCTGGTCGCCTTCTCTATGCTGCAACAGCGGATGATTCTTCCTCTTCCACCAGCAGCGACAGCCTGGGTGGGGGATACTGCGCCGCTAGGCTGGAGCCCAGCCTTCCACATCCCCACCATCAGGTTCTGCAGCCCCATCTGCCTCGAAAGGTGGACACGGCTGCTCAGACCAATAGCCGCCTGGCCCGGCCCACGAGGCTGTCCCTGGGGGATCCCAAGGCCAGCACCTTACCTCGGGCCCGAGAGCAGCAGCAGCAGCAGCAGCCCTTGCTGCACCCTCCAGAGCCCAAGAGCCCGGGGGAATATGTCAATATTGAATTTGGGAGTGATCAGCCTGGCTACTTGTCTGGCCCGGTGGCTTCCCACAGCTCACCTTCTGTCAGGTGTCCATCCCAGCTCCAGCCAGCTCCCAGAGAGGAAGAGACTGGCACTGAGGAGTACATGAAGATGGACCTGGGGCCGGGCCGGAGGGCAGCCTGGCAGGAGAGCACTGGGGTCGAGATGGGCAGACTGGGCCCTGCACCTCCCGGGGCTGCTAGCATTTGCAGGCCTACCCGGGCAGTGCCCAGCAGCCGGGGTGACTACATGACCATGCAGATGAGTTGTCCCCGTCAGAGCTACGTGGACACCTTGCCAGCTGCCCCTGTAAGCTATGCTGACATGCGGACAGGCATTGCTGCAGAGGAGGTGAGCCTGCCCAGGGCCACCATGGCTGCTGCCTCCTCATCCTCAGCAGCCTCTGCTTCCCTGACTGGGCCTCAAGGGGCAGCAGAGCTGGCTGCCCACTCGTCCCTGCTGGGGGGCCCACAAGGACCTGGGGGCATGAGCGCCTTCACCCGGGTGAACCTCAGTCCTAACCGCAACCAGAGTGCCAAAGTGATCCGTGCAGACCCACAAGGGTGCCGGCGGAGGCATAGCTCCGAGACTTTCTCCTCAACACCCAGTGCCACCCGGGTGGGCAACACAGTGCCCTTTGGAGCGGGGGCAGCAGTAGGGGGCGGTGGCGGTAGCAGCAGCAGCAGCGAGGATGTGAAACGCCACAGCTCTGCTTCCTTTGAGAATGTGTGGCTGAGGCCTGGGGAGCTTGGGGGAGCCCCCAAGGAGCCAGCCCAACTGTGTGGGGCTGCTGGGGGTTTGGAGAATGGTCTTAACTACATAGACCTGGATTTGGTCAAGGACTTCAAACAGTGCGCTCAGGAGTGCACCCCTGAACCGCAGCCTCCCCCACCCCCACCCCCTCATCAACCCCTGGGCAGCGGTGAGAGCAGCTCCATCCGCCGCTCAAGTGAGGATTTAAGCGCCTATGCCAGCATCAGTTTCCAGAAGCAGCCAGAGGACCGTCAGTAG

>Pon_ab1 [Orangutan (Pongo abelii) Irs1]

ATGGCGAGCCCTCCGGAGAGCGATGGCTTCTCGGACGTGCGCAAGGTGGGCTACCTGCGCAAACCCAAGAGCATGCACAAACGCTTCTTCGTACTGCGCGCTGCCAGCGAGGCTGGGGGCCCGGCGCGCCTCGAGTACTACGAGAACGAGAAGAAGTGGCGGCACAAGTCGAGCGCCCCCAAACGCTCGATCCCCCTTGAGAGCTGCTTCAACATCAACAAGCGGGCTGACTCCAAGAACAAGCACCTGGTGGCTCTCTACACCCGAGACGAGCACTTTGCCATCGCGGCGGACAGCGAGGCCGAGCAAGACAGCTGGTACCAGGCTCTCCTACAGCTGCACAACCGTGCTAAGGGCCACCACGACGGAGCTGCTGCCCTCGGTGCGGGAGGTGGTGGGGGCAGCTGCAGTGGCAGCTCCGGCCTTGGTGAGGCTGGGGAGGACTTGAGCTACGGTGACGTGCCCCCAGGACCCGCATTCAAAGAGGTCTGGCAGGTGATCCTGAAGCCCAAGGGCCTGGGTCAGACAAAGAACCTGATTGGTATCTACCGCCTCTGCCTGACCAGCAAGACCATCAGCTTCGTGAAGCTGAACTCGGAGGCAGCCGCCGTGGTGCTGCAGCTGATGAACATCAGGCGCTGTGGCCACTCGGAGAACTTCTTCTTCATCGAGGTGGGCCGTTCTGCCGTGACGGGTCCCGGGGAGTTCTGGATGCAGGTGGATGACTCCGTGGTGGCCCAGAACATGCACGAGACCATCCTGGAGGCCATGCGGGCCATGAGTGATGAGTTCCGCCCTCGCAGCAAGAGCCAGTCCTCGTCCAACTGCTCTAACCCCATCAGCGTCCCCCTGCGCCGGCACCATCTCAACAATCCCCCGCCCAGCCAGGTGGGGCTGACCCGCCGATCGCGCACTGAGAGCATCACCGCCACCTCCCCGGCCAGCATGGTGGGCGGGAAGCCAGGCTCCTTCCGTGTCCGCGCCTCCAGTGACGGCGAAGGCACTATGTCCCGCCCAGCCTCGGTAGACGGCAGCCCTGTGAGTCCCAGCACCAACAGAACCCACGCCCACCGGCATCGGGGCAGCGCCCGGCTGCACCCCCCGCTCAACCACAGCCGTTCCATCCCCATGCCGGCTTCCCGCTGCTCGCCTTCGGCCACCAGCCCGGTCAGTCTGTCGTCCAGTAGCACCAGTGGCCATGGCTCCACCTCGGATTGTCTCTTCCCACGGCGATCTAGTGCTTCGGTGTCTGGTTCCCCCAGCGATGGCGGTTTCATCTCCTCAGATGAGTATGGCTCCAGTCCCTGTGATTTTCGGAGTTCCTTCCGCAGCGTCACTCCGGATTCCCTGGGCCACACCCCACCAGCCCGCGGTGAGGAGGAGCTAAGCAACTATATCTGCATGGGTGGCAAGGGGCCCTCCACCCTGACCGCCCCCAACGGTCAATACGTTTTGTCTCGGGGTGGCAATGGCCACCGCTACACCCCAGGAACAGGCTTGGGCACGAGTCCAGCCTTGGCTGGGGATGAAGCAGCCAGTGCTGCAGATCTGGATAATCGGTTCCGAAAGAGAACTCACTCGGCAGGCACATCCCCTACCATTACCCACCAGAAGACCCCGTCCCAGTCCTCAGTGGCTTCCATTGAGGAGTATACAGAGATGATGCCTGCCTACCCACCAGGAGGTGGCAGTGGAGGCCGACTGCCGGGACACAGGCACTCCGCCTTCGTGCCCACCCACTCCTACCCAGAGGAGGGTCTGGAAATGCACCCCTTGGAGCGTCGGGGGGGCCACCACCGCCCAGACAGCTCCACCCTCCACACTGATGATGGCTACATGCCCATGTCCCCAGGGGTGGCCCCAGTGCCCAGCAGCCGAAAGGGCAGTGGAGACTATATGCCCATGAGTCCCAAGAGCGTGTCTGCCCCACAGCAGATCATCAATCCCATCAGACGCCATCCCCAGAGAGTGGACCCCAATGGCTACATGATGATGTCCCCCAGCGGTGGCTGCTCTCCTGACATTGGAGGTGGCCCCAGCAGCAGCAGCAGCAGCAGCAATGCTGTCCCTTCCGGGAGCAGCTATGGAAAGCTGTGGACAAACGGGGTAGGGGGCCACCACTCTCATGTCTTGCCTCACCCCAAACCCCCAGTGGAGAGCAGCAGTGGTAAGCTCTTACCTTGTACAGGTGACTACATGAACATGTCACCAGTGGGGGACTCCAACACCAGCAGCCCCTCCGACTGCTACTACGGCCCTGAGAACCCCCAGCACAAGCCAGTCCTCTCCTACTACTCATTGCCAAGATCCTTTAAGCACACCCAGCACCCTGGGGAGCCGGAGGAGGGTGCCCGGCATCAGCACCTCCGCCTTTCCACTAGCTCTGGTCGCCTTCTCTATGCTGCAACAGCGGATGATTCTTCCTCCTCCACCAGCAGTGACAGCCTGGGTGGGGGATACTGCGGGGCTAGGCTGGAGCCCAGCCTTCCACATCCCCACCATCAGGTTCTGCAGCCCCATCTGCCTCGAAAGGTGGACACAGCTGCTCAGACCAATAGCCGCCTGGCCCGGCCCACGTTGCTGTCCCTGGGGGATCCCAAGGCCAGCACCTTACCTCGGGCCCGAGAGCAGCAGCAGCAGCAGCAGCAGCAGCAGCAGCAGCCCCTGCTGCACCCTCCAGAGCCCAAGAGCCCAGGGGAATATGTCAATATTGAATTTGGGAGTGATCAGCCTGGCTACTTGTCTGGCCCGGTGGCTTCCCACAGCTCACCTTCTGTCAGGTGTCCATCCCAGCTCCAGCCAGCTCCCAGAGAGGAAGAGACTGGCACTGAGGAGTACATGAAGATGGACCTGGGGCCGGGCCGGAGGGCAGCCTGGCAGGAGAGCACTGGGGTCGAGATGGGCAGACTGGGCCCCGCACCTCCCGGGGCTGCTAGCATTTGCAGGCCTACCCGGGCAGTGCCCAGCAGCCGGGGTGACTACATGACCATGCAGATGAGTTGTCCCCGTCAGAGCTACGTGGACACCTCGCCAGCTGCCCCTGTAAGCTATGCTGACATGCGGACAGGCATTGCTGCAGAGGAGGTGAGCCTGCCGAGGGCCACCATGGCTGCTGCCTCCTCATCCTCAGCAGCCTCTGCTTCCCCGACTGGGCCTCAAGGGGCAGCAGAGCTGGCTGCCCACTCGTCCCTGCTGGGGGGCCCACAAGGACCTGGGGGCATGAGCGCCTTCACCCGGGTGAACCTCAGTCCTAACCGCAACCAGAGTGCCAAAGTGATCCGTGCAGACCCACAAGGGTGCCGGCGGAGGCATAGCTCCGAGACCTTCTCCTCAACACCCAGTGCCACTCGGGTGGGCAACACAGTGCCCTTTGGAGCAGGGGCAGCAGTAGGGGGCGGTGGCGGTAGCAGCAGCAGCAGCGAGGATGTGAAACGCCACAGCTCTGCTTCCTTTGAGAATGTGTGGCTGAGGCCTGGGGAGCTTGGGGGAGCCCCCAAGGAGCCAGCCCAACTGTGTGGGGCTGCTGGGGGTTTGGAGAATGGTCTTAACTACATAGACCTGGATTTGGTCAAGGACTTCAAACAGCGCCCTCAGGAGTGCACCCCTGAACCGCAGCCTCCCCCACCCCCACCCCCTCATCAACCCCTGGGCAGCGGTGAGAGCAGCTCCACCCGCCGCTCAAGTGAGGATTTAAGCGCCTATGCCAGCATCAGTTTCCAGAAGCAGCCAGAGGACCGTCAGTAG

>Nos_le1 [Gibbon (Nomascus leucogenys) Irs1]

ATGGCGAGCCCTCCGGAGAGCGATGGCTTCTCGGACGTGCGCAAGGTGGGCTACCTGCGCAAACCCAAGAGCATGCACAAACGCTTCTTCGTACTGCGCGCGGCCAGCGAGGCTGGGGGCCCGGCGCGCCTCGAGTACTACGAGAACGAGAAGAAGTGGCGGCACAAGTCGAGCGCCCCCAAACGCTCGATCCCCCTTGAGAGCTGCTTCAACATCAACAAGCGGGCTGACTCCAAGAACAAGCACCTGGTGGCTCTCTACACCCGGGACGAGCACTTTGCCATCGCGGCGGATAGCGAGGCCGAGCAAGACAGCTGGTACCAGGCTCTCCTACAGCTGCACAACCGTGCTAAGGGCCACCACGACGGAGCTGGGGCCCTCGGGGCGGGAGGTGGCGGGGGCAGCTGCAGCGGCAGCTCCGGCCTTGGTGAGGCTGGGGAGGACTTGAGCTACGGTGACGTGCCCCCAGGACCCGCATTCAAAGAGGTCTGGCAGGTGATCCTGAAGCCCAAGGGCCTGGGTCAGACAAAGAACCTGATTGGTATCTACCGCCTCTGCCTGACCAGCAAGACCATCAGCTTCGTGAAGCTGAACTCGGAGGCAGCCGCCGTGGTGCTGCAGCTGATGAACATCAGGCGCTGTGGCCACTCGGAGAACTTCTTCTTCATCGAGGTGGGCCGTTCTGCTGTGACGGGGCCCGGGGAGTTCTGGATGCAGGTGGATGACTCCGTGGTGGCCCAGAACATGCATGAGACCATCCTGGAGGCCATGCGGGCCATGAGTGACGAGTTCCGCCCTCGCAGCAAGAGCCAGTCCTCGTCCAACTGCTCTAACCCCATCAGCGTGCCCCTGCGCCGGCACCATCTCAACAACCCCCCGCCCAGCCAGGTGGGGCTGACCCGCAGATCGCGCACTGAGAGCATTACTGCCACCTCCCCGGCCAGCATGGTGGGCGGGAAGCCAGGCTCCTTCCGTGTCCGCGCCTCCAGTGACGGCGAAGGCACCATGTCCCGCCCAGCCTCGGTGGACGGCAGCCCTGTGAGTCCCAGCACCAACAGAACCCACGCCCACCGGCATCGGGGCAGCGCCCGGCTGCATCCCCCGCTCAACCACAGCCGCTCCATCCCCATGCCGGCTTCCCGCTGCTCGCCTTCGGCCACCAGCCCGGTCAGTCTGTCGTCCAGCAGCACCAGTGGCCATGGCTCCACCTCGGATTGTCTCTTCCCACGGCGATCTAGTGCTTCGGTGTCTGGTTCCCCCAGCGATGGCGGTTTCATCTCCTCGGATGAGTATGGCTCCAGTCCCTGCGATTTCCGGAGTTCCTTCCGCAGTGTCACTCCGGATTCCCTGGGCCACACCCCACCAGCCCGCGGTGAGGAGGAGCTAAGCAACTATATCTGCATGGGTGGCAAGGGGCCTTCCACCCTGACCGCCCCCAACGGTCACTATATTTTGTCTCGGGGTGGCAATGGCCACCGCTACGCCCCAGGAACAGGCTTGGGCACGAGTCCAGCCTTGGCTGGGGATGAAGCAGCCAGTGCTGCAGATCTGGATAATCGGTTCCGAAAGAGAACTCACTCGGCAGGCACATCCCCTACCATTACCCACCAGAAGACCCCGTCCCAGTCCTCAGTGGCTTCCATTGAGGAGTATACAGAGATGATGCCTGCCTACCCACCAGGAGGTGGCAGTGGAGGCCGACTGCCGGGACACAGGCACTCCGCCTTCGTGCCCACCCACTCCTACCCAGAGGAGGGTCTGGAAATGCACCCCTTGGAGCGTCGGGGGGGCCACCACCGCCCAGACAGCTCCACCCTCCACACTGATGATGGCTACATGCCCATGTCCCCAGGGGTTGCCCCAGTGCCCAGCAGCCGAAAGGGCAGTGGAGACTATATGCCCATGAGCCCCAAGAGTGTGTCTGCCCCACAGCAGATCATCAATCCCATCAGACGCCATCCCCAGAGAGTGGACCCCAATGGCTACATGATGATGTCTCCCAGTGGCGGCTGCTCTCCTGACATTGGAGGTGGCCCCAGCAGCAGCAGCAGCAATGCCGTCCCTTCCGGGAGCAGCTATGGAAAGCTGTGGACAAACGGGGTAGGGGGCCACCACTCTCATGTCTTGCCTCACCCCAAACCCCCAGTGGAGAGCAGTGGTGGTAAGCTCTTACCTTGCACAGGTGACTACATGAACATGTCACCAGTGGGGGACTCCAACACCAGCAGCCCCTCCGACTGCTACTATGGCCCTGAGGACCCCCAGCACAAGCCAGTCCTCTCCTACTACTCATTGCCAAGATCCTTTAAGCACACCCAGCGCCCTGGAGAGCCGGAGGAGGGTGCCCGGCATCAGCACCTCCGTCTTTCCACTAGCTCTGGTCGCCTTCTCTATGCTGCAACAGCGGATGATTCTTCCTCTTCCACCAGCAGTGACAGCCTGGGTGGGGGATACTGTGGGGCTAGGCTGGAGCCCAGCCTTCCACATCCCCACCATCAGGTTCTGCAGCCCCATCTGCCTCGAAAGGTGGACACAGCTGCTCAGACCAATAGCCGCCTGGCCCGGCCCACGAGGCTGTCCCTGGGGGATCCCAAGGCCAGCACCTTACCTCGGGCCCGAGAGCAGCAGCAGCAGCAGCAGCAGCAGCCCCTGCTGCACCCTCCAGAACCCAAGAGCCCAGGGGAATATGTCAATATTGAATTCGGGAGTGATCAGCCTGGCTACTTGTCTGGCCTGGTGGCTTCCCACAGCTCACCTTCTGTCAGGTGTCCATCCCAGCTCCAGCCAGCTCCCAGAGAGGAAGAGACTGGCACTGAGGAGTACATGAAGATGGACCTGGGGCCGGGCCGGAGGGCAGCCTGGCAGGAGAGCACTGGGGTCGAGATGGGCAGACTGGGCCCCGCACCTCCTGGGGCTGCTAGCATTTGCAGGCCTACCCGGGCAGTGCCCAGCAGCCGGGGTGACTACATGACCATGCAGATGAGTTGTCCCCGTCAGAGCTACGTGGACACCTCGCCAGCTGCCCCTGTAAGCTATGCTGACATGCGGACAGGCATTGCTGCCGAGGAATTGAGCCTGCCCAGGGCCACCATGGCTGCTGCCTCCTCATCCTCTCAGCCTCTGCTTCACGACTGGGCCTTCAAGGTGCACAGAGCTGGCTGCCCACTTCGTCCCTGCTGGGGGGCCCAAAGGACTGGGGGCTTGAGCGCCTTCACCCGGGTGAACCTCAGTCCTAACCGCAACCAGAGTGCCAAAGTGATCCGTGCAGACCCACAAGGGTGCCGGCGGAGGCATAGCTCTGAGACCTTCTCCTCAACACCCAGTGCCACCCGGGTGAGCAACACAGTGCCCTTTGGAGCGGGGGCAGCAGTAGGGGGTGGTGGCGGTAGCAGCAGCAGCAGCAGCGAGGATGTGAAACGCCACAGCTCTGCTTCCTTTGAGAATGTGTGGCTGAGGCCTGGGGAGCTTGGGGGAGCCCCCAAGGAGCCAGCCCAACTGTGTGGGGCTGCTGGGGGTTTGGAGAATGGTCTCAACTACATAGACCTGGATTTGGTCAAGGACTTCAAACAGCGCCCTCAGGAGTGCACCCCTGAACCGCAGCCTCCCCCACCCCCACCCCCTCATCAACCCCTGGGCAGCGGTGAGAGCAGCTCCACCCGCTGCTCAAGTGAGGATTTAAGCGCCTATGCCAGCATCAGTTTCCAGAAGCAGCCAGAGGACCGTCAGTAG

>Mac_mu1 [Rhesus monkey (Macaca mulatta) Irs1]

ATGGCGAGCCCTCCGGAGAGCGATGGCTTCTCGGACGTGCGCAAGGTGGGCTACCTGCGCAAACCCAAGAGCATGCACAAACGCTTCTTCGTACTGCGCGCGGCCAGCGAGGCTGGGGGCCCGGCGCGCCTCGAGTACTACGAGAACGAGAAGAAGTGGCGGCACAAGTCGAGCGCCCCCAAACGCTCGATCCCCCTTGAGAGCTGCTTCAACATCAACAAGCGGGCTGACTCCAAGAACAAGCACCTGGTGGCTCTCTACACCCGGGACGAGCACTTTGCCATCGCGGCGGACAGCGAGGCGGAGCAAGACAGCTGGTACCAGGCTCTCCTACAGCTGCACAACCGTGCCAAGGGCCACCACGACGGAGCTGCGGCCCTCGGGGCGGGAGGTGGCGGGGGCAGCTGCAGTGGCAGCTCCGGCGTTGGTGAGGCTGGGGAGGACTTGAGCTACGGTGACGTGCCCCCAGGACCCGCGTTCAAAGAGGTCTGGCAGGTGATCCTGAAGCCCAAGGGCCTGGGTCAGACAAAGAACCTGATTGGTATCTACCGCCTCTGCCTGACCAGCAAGACCATCAGCTTCGTGAAGCTGAACTCGGAGGCAGCCGCCGTGGTGCTGCAGCTGATGAACATCAGGCGCTGCGGCCACTCGGAGAACTTCTTCTTCATCGAGGTGGGCCGTTCTGCCGTGACGGGGCCCGGGGAGTTCTGGATGCAGGTGGATGACTCCGTGGTGGCCCAGAACATGCACGAGACCATCCTGGAGGCCATGCGGGCTATGAGCGATGAGTTCCGCCCTCGCAGCAAGAGTCAGTCCTCGTCCAACTGCTCCAACCCCATCAGCGTCCCCCTGCGCCGGCACCATCTCAACAACCCCCCGCCCAGCCAGGTGGGGCTGACCCGACGATCTCGCACTGAGAGCATCACTGCCACCTCCCCGGCCAGCATGGTGGGCGGGAAACCAGGCTCCTTCCGCGTCCGCGCCTCCAGTGACGGCGAAGGCACCATGTCCCGCCCAGCCTCGGTGGATGGCAGCCCTGTGAGTCCGAGCACCAACAGAACCCACGCCCACCGGCATCGGGGCAGCGCCCGGCTGCACCCCCCGCTCAACCACAGCCGCTCCATCCCCATGCCAGCTTCCCGCTGCTCGCCTTCGGCCACCAGCCCCGTCAGTCTGTCGTCCAGCAGCACCAGCGGCCATGGCTCCACCTCGGATTGTCTCTTCCCACGGCGATCTAGTGCTTCGGTGTCTGGTTCTCCCAGCGATGGCGGTTTCATCTCCTCAGATGAGTATGGCTCCAGTCCCTGCGATTTCCGGAGTTCCTTCCGCAGTGTGACTCCGGATTCCCTGGGCCACACCCCACCAGCCCGCGGTGAGGAGGAGCTAAGCAACTATATCTGCATGGGCGGCAAGGGGCCCTCCACCCTGACCGCCCCCAACGGTCACTACATTTTGTCTCGGGGTGGCAATGGCCACCGCTACACCCCAGGAACAGGCTTGGGCACGAGTCCAGCCTTGGCTGGGGATGAAGCATCCAGTGCTGCGGATCTGGATAATCGCTTCCGAAAGAGAACTCACTCTGCAGGCACATCCCCTACCATTACCCACCAGAAGACCCCGTCCCAGTCCTCAGTGGCTTCCATTGAGGAGTATACAGAGATGATGCCTGCCTACCCACCAGGAGGTGGCAGTGGAGGCCGACTGCCGGGACACAGGCACTCTGCCTTCGTGCCCACCCACTCCTACCCAGAGGAGGGTCTGGAAATGCACCCCTTGGAGCGTCGGGGGGGCCACCACCGCCCAGACAGCTCTACCCTCCACACTGATGATGGCTACATGCCCATGTCCCCAGGGGTGGCCCCAGTGCCCAGCAGCCGAAAGGGCAGTGGAGACTATATGCCCATGAGCCCCAAGAGCGTGTCTGCCCCACAGCAGATCATCAATCCCATCAGACGCCATCCCCAGAGAGTGGACCCCAATGGCTACATGATGATGTCCCCCAGCGGTGGCTGCTCTCCTGACATTGGAGGTGGCCCCAGCAGCAGCAGCAGCAGTGCTGTCCCTTCTGGGAGCAGCTATGGAAAGCTGTGGACAAACGGGGTAGGGGGCCACCACTCTCATGTCTTGCCTCACCCCAAACCCCCAGTGGAGAGCAGCGGTGGCAAGCTCTTACCTTGCACAGGTGACTACATGAACATGTCACCAGTGGGGGACTCCAACACCAGCAGCCCCTCTGACTGCTACTATGGCCCTGAGGACCCCCAGCACAAGCCAGTCCTCTCGTACTACTCATTGCCAAGATCCTTTAAGCACACCCAGCGCCCTGGGGAGCCGGAGGAGGGTGCCCGGCATCAGCACCTCCGCCTTTCTGCTAGCTCTGGTCGCCTTCTCTATGCTGCAACAGTGGATGATTCTTCCTCCTCCACCAGCAGCGACAGCCTGGGTGGGGGATACTGTGGGGCTAGGCTGGAGCCCAGCCTTCCACATCCCCACCATCAGGTTCTGCAGCCCCATCTGCCTCGAAAGGTGGACACAGCTGCTCAGACCAATAGCCGCCTGGCCCGGCCTACGAGGCTGTCCCTGGGGGATCCCAAGGCCAGCACCTTACCTCGGGCCCGAGAGCAGCAGCAGCAGCAGCAGCAGCAGCAGCAGCAGCAACAGCAGCAACAGCAGCCCCTGCTGCACCCTCCGGAGCCCAAGAGCCCAGGGGAATATGTGAATATTGAATTTGGGAGTGATCAGCCTGGCTACTTGTCTGGCCCGGTGGCTTCCCGTAGCTCACCTTCTGTCAGGTGTCCATCCCAGCTCCAGCCAGCTCCCAGAGAGGAAGAGACTGGCACTGAGGAGTACATGAAGATGGACCTGGGGCCGGGCCGGAGGGCAGCCTGGCAGGAGAGCACTGGGGTCGAGATGGGCAGACTGGGGCCCGCACCTCCCGGGGCTGCTAGCATTTGCAGGCCTACCCGGGCAGTGCCCAGCAGCCGGGGTGACTATATGACCATGCAGATGAGTTGTCCCCGCCAGAGCTATGTGGACACCTCGCCAATTGCCCCTGTAAGCTATGCTGACATGCGGACAGGCATTGCTGCAGAGGAGGTGAGCCTGCCCAGGGCCACCATGGCTGCTGCCTCCTCATCCTCAGCAGCCTCTGCTTCCCCCACTGGGCCTCAAGGGGCAGCAGAGCTGGCTGCCCACTCATCCCTGCTGGGGGGCCCACAAGGACCTGGGGGCATGAGCGCCTTCACCCGGGTGAACCTCAGTCCTAACCGCAACCAGAGTGCCAAAGTGATCCGTGCAGACCCACAAGGGTGCCGGAGGAGGCATAGCTCCGAGACCTTCTCCTCAACACCCAGTGCCACCCGGGTGGGCAACACAGTGCCCTTTGGAGCCGGGGCAGCAATAGGGGGTGGTGGCGGTAGCAGCAGCAGCAGCGAGGATGTGAAACGTCACAGCTCTGCTTCCTTTGAGAATGTGTGGCTGAGGCCTGGGGAACTTGGGGGAGCCCCCAAGGAGCCAGCCCAACTGTGTGGGGCTGCTGGGGGTTTGGAGAATGGTCTTAACTACATAGATCTGGATTTGGTCAAGGACTTCAAACAGCGCCCTCAGGAGTGCAGCCCTCACCCACAGCCTCCCCCACCCCCACCCCCTCATCAACCCCTGGGTAGCAGTGAGAGCAGCTCCACCCGCCGCCGCTCAAGTGAGGATTTAAGCGCCTATGCCAGCATCAGTTTCCAGAAGCAGCCAGAGGACCTTCAGTAG

>Man_le1 [Drill (Mandrillus leucophaeus) Irs1]

ATGGCGAGCCCTCCGGAGAGCGATGGCTTCTCGGACGTGCGCAAGGTGGGCTACCTGCGCAAACCCAAGAGCATGCACAAACGCTTCTTCGTACTGCGCGCGGCCAGCGAGGCTGGGGGCCCGGCGCGCCTCGAGTACTACGAGAACGAGAAGAAGTGGCGGCACAAGTCGAGCGCCCCCAAACGCTCGATCCCCCTTGAGAGCTGCTTCAACATCAACAAGCGGGCTGACTCCAAGAACAAGCACCTGGTGGCTCTCTACACCCGGGACGAGCACTTTGCCATCGCGGCGGACAGCGAGGCGGAGCAAGACAGCTGGTACCAGGCTCTCCTACAGCTGCACAACCGTGCCAAGGGCCACCACGACGGAGCTGCGGCCCTCGGGGCGGGAGGTGGCGGGGGCAGCTGCAGTGGCAGCTCCGGCCTTGGTGAGGCTGGGGAGGACTTGAGCTACGGTGACGTGCCCCCAGGACCCGCGTTCAAAGAGGTCTGGCAGGTGATCCTGAAGCCCAAGGGCCTGGGTCAGACAAAGAACCTGATTGGTATCTACCGCCTCTGCCTGACCAGCAAGACCATCAGCTTCGTGAAGCTGAACTCGGAGGCAGCCGCCGTGGTGCTGCAGCTGATGAACATCAGGCGCTGCGGCCACTCGGAGAACTTCTTCTTCATCGAGGTGGGCCGTTCTGCCGTGACGGGGCCCGGGGAGTTCTGGATGCAGGTGGATGACTCCGTGGTGGCCCAGAACATGCACGAGACCATCCTGGAGGCCATGCGGGCTATGAGCGATGAGTTCCGCCCTCGCAGCAAGAGCCAGTCCTCGTCCAACTGCTCCAACCCCATCAGCGTCCCCCTGCGCCGGCACCATCTCAACAACCCCCCGCCCAGCCAGGTGGGGCTGACCCGACGATCTCGCACTGAGAGCATCACTGCCACCTCCCCGGCCAGCATGGTGGGCGGGAAACCAGGCTCCTTCCGCGTCCGCGCCTCCAGTGACGGCGAAGGCACCATGTCCCGCCCAGCCTCGGTGGATGGCAGCCCTGTGAGTCCGAGCACCAACAGAACCCACGCCCACCGGCATCGGGGCAGTGCCCGCCTGCACCCTCCGCTCAACCACAGCCGCTCCATCCCCATGCCAGCTTCCCGCTGCTCGCCTTCGGCCACCAGCCCCGTCAGTCTGTCGTCCAGCAGCACCAGCGGCCATGGCTCCACCTCGGATTGTCTCTTCCCACGGCGATCTAGTGCTTCGGTGTCTGGTTCTCCCAGCGATGGCGGTTTCATCTCCTCAGATGAGTATGGCTCCAGTCCCTGCGATTTCCGGAGTTCCTTCCGCAGTGTCACTCCGGATTCCCTGGGCCACACCCCACCAGCCCGCGGTGAGGAGGAGCTAAGCAACTATATCTGCATGGGCGGCAAGGGGCCCTCCACCCTGACCGCCCCCAACGGTCACTACATTTTGTCTCGGGGTGGCAATGGTCACCGCTACACCCCAGGAACAGGCTTGGGCACGAGTCCAGCCTTGGCTGGGGATGAAGCATCCAGTGCTGCAGATCTGGATAATCGCTTCCGAAAGAGAACTCACTCTGCAGGCACATCCCCTACCATTACCCACCAGAAGACCCCGTCCCAGTCCTCAGTGGCTTCCATTGAGGAGTATACAGAGATGATGCCTGCCTACCCACCAGGAGGTGGCAGTGGAGGCCGACTGCCGGGACACAGGCACTCTGCCTTCGTGCCCACCCACTCCTACCCAGAGGAGGGTCTGGAAATGCACCCCTTGGAGCGTCGGGGGGGCCACCACCGCCCAGACAGCTCTACCCTCCACACTGATGATGGCTACATGCCCATGTCCCCAGGGGTGGCCCCAGTGCCCAGCAGCCGAAAGGGCAGTGGAGACTATATGCCCATGAGCCCCAAGAGCGTGTCTGCCCCACAGCAGATCATCAATCCCATCAGACGCCATCCCCAGAGAGTGGACCCCAATGGCTACATGATGATGTCCCCCAGCGGTGGCTGCTCTCCTGACATTGGAGGTGGCCCCAGCAGCAGCAGCAGCAGCAGTGCCGTCCCTTCCGGGAGCAGCTATGGAAAGCTGTGGACAAACGGGGTAGGGGGCCACCACTCTCATGTCTTGCCTCACCCCAAACCCCCAGTGGAGAGCAGCGGTGGCAAGCTCTTACCTTGCACAGGTGACTACATGAACATGTCACCAGTGGGGGACTCCAACACCAGCAGCCCCTCCGACTGTTACTATGGCCCTGAGGACCCACAGCACAAGCCAGTCCTCTCGTACTACTCATTGCCAAGATCCTTTAAGCACACCCAGCGCCCTGGGGAGCCGGAGGAGGGTGCCCGGCATCAGCACCTCCGCCTTTCCGCTAGCTCTGGTCGCCTTCTCTATGCTGCAACAGCGGATGATTCTTCCTCCTCTACCAGCAGCGACAGCCTGGGTGGGGGATACTGTGGGGCTAGGCTGGAGCCCAGCCTTCCACATCCCCACCATCAGGTTCTGCAGCCCCATCTGCCTCGAAAGATGGACACAGCTGCTCAGACCAATAGCCGCCTGGCCCGGCCTACGAGGCTGTCCCTGGGGGATCCCAAGGCCAGCACCTTACCTCGGGCCCGAGAGCAGCAGCAGCAGCAGCAGCAGCAGCAGCAACAGCAGCAACAGCAGCCCCTGCTGCACCCTCCGGAGCCCAAGAGCCCAGGGGAATATGTGAATATTGAATTTGGGAGTGATCAGCCTGGCTACTTGTCTGGCCCGGTGGCTTCCCGTAGCTCACCTTCTGTCAGGTGTCCATCCCAGCTCCAGCCAGCTCCCAGAGAGGAAGAGACTGGCACTGAGGAGTACATGAAGATGGACCTGGGGCCGGGCCGGAGGGCAGCCTGGCAGGAGAGCACTGGGGTCGAGATGGGCAGACTGGGCCCCGCACCTCCCGGGGCTGCTAGCATTTGCAGGCCTACCCGGGCAGTGCCCAGCAGCCGGGGTGACTATATGACCATGCAGATGAGTTGTCCCCGCCAGAGCTATGTGGACACCTCGCCAATTGCCCCTGTAAGCTATGCTGACATGCGGACAGGCATTGCTGCAGAGGAGGTGAGCCTGCCCAGGGCCACCATGGCTGCTGCCTCCTCATCCTCAGCAGCCTCTGCTTCCCCCACTGGGCCTCAAGGGGCAGCAGAGCTGGCTGCCCACTCATCCCTGCTGGGGGGCCCACAAGGACCTGGGGGCATGAGCGCCTTCACCCGGGTGAACCTCAGTCCTAACCGCAACCAGAGTGCCAAAGTGATCCGTGCAGACCCACAAGGGTGCCGGAGGAGGCATAGCTCCGAGACCTTCTCCTCAACACCCAGTGCCACCCGGGTGGGCAACACAGTGCCCTTTGGAGCCGGGGCAGCAATAGGGGGTGGTGGCGGTAGCAGCAGCAGCAGCGAGGATGTGAAACGTCACAGCTCTGCTTCCTTTGAGAATGTGTGGCTGAGGCCTGGGGAACTTGGGGGAGCCCCCAAGGAGCCAGCCCAACTGTGTGGGGCTGCTGGGGGTTTGGAGAATGGTCTTAACTACATAGATCTGGATTTGGTCAAGGACTTCAAACAGCGCCCTCAGGAGTGCACCCCTCAACCGCAGCCTCCCCCACCCCCACCCCTTCATCAACCCCTGGGTAGCAGTGAGAGCAGCTCCACCCCCCGCCGCTCAAGTGAGGATTTAAGCGCCTATGCCAGCATCAGTTTCCAGAAGCAGCCAGAGGACCTTCAGTAG

>Pap_an1 {Olive baboon (Papio anubis) Irs1]

ATGGCGAGCCCTCCAGAGAGCGATGGCTTCTCGGACGTGCGCAAGGTGGGCTACCTGCGCAAACCCAAGAGCATGCACAAACGCTTCTTCGTACTGCGCGCGGCCAGCGAGGCTGGGGGCCCGGCGCGCCTCGAGTACTACGAGAACGAGAAGAAGTGGCGGCACAAGTCGAGCGCCCCCAAACGCTCGATCCCCCTTGAGAGCTGCTTCAACATCAACAAGCGGGCTGACTCCAAGAACAAGCACCTGGTGGCTCTCTACACCCGGGACGAGCACTTTGCCATCGCGGCGGACAGCGAGGCGGAGCAAGACAGCTGGTACCAGGCTCTCCTACAGCTGCACAACCGTGCCAAGGGCCACCACGACGGAGCTGCGGCCCTCGGGGCTGGAGGTGGCGGGGGCAGCTGCAGTGGCAGCTCCGGCCTTGGTGAGGCTGGGGAGGACTTGAGCTACGGTGACGTGCCCCCAGGACCCGCGTTCAAAGAGGTCTGGCAGGTGATCCTGAAGCCCAAGGGCCTGGGTCAGACAAAGAACCTGATTGGTATCTACCGCCTCTGCCTGACCAGCAAGACCATCAGCTTCGTGAAGCTGAACTCGGAGGCAGCCGCCGTGGTGCTGCAGCTGATGAACATCAGGCGCTGCGGCCACTCGGAGAACTTCTTCTTCATCGAGGTGGGCCGTTCTGCCGTGACGGGGCCCGGGGAGTTCTGGATGCAGGTGGATGACTCCGTGGTGGCCCAGAACATGCACGAGACCATCCTGGAGGCCATGCGGGCTATGAGCGATGAGTTCCGCCCTCGCAGCAAAAGCCAGTCCTCGTCCAACTGCTCCAACCCCATCAGCGTCCCCCTGCGCCGGCACCATCTCAACAACCCCCCGCCCAGCCAGGTGGGGCTGACCCGACGATCTCGCACTGAGAGCATCACTGCCACCTCCCCGGCCAGCATGGTGGGCGGGAAACCAGGCTCCTTCCGCGTCCGCGCCTCCAGTGACGGCGAAGGCACCATGTCCCGCCCAGCCTCGGTGGATGGCAGCCCTGTGAGTCCGAGCACCAACAGAACCCACGCCCACCGGCATCGGGGCAGTGCCCGGCTACACCCCCCGCTCAACCACAGCCGCTCCATCCCCATGCCAGCTTCCCGCTGCTCGCCTTCGGCCACCAGCCCCGTCAGTCTGTCGTCCAGCAGCACCAGCGGCCATGGCTCCACCTCGGATTGTCTCTTCCCACGGCGATCTAGTGCTTCGGTGTCTGGTTCTCCCAGCGATGGCGGTTTCATCTCCTCAGATGAGTATGGCTCCAGTCCCTGCGATTTCCGGAGTTCCTTCCGCAGTGTCACTCCGGATTCCCTGGGCCACACCCCACCAGCCCGCGGTGAGGAGGAGCTAAGCAACTATATCTGCATGGGCGGCAAGGGGCCCTCCACCCTGACCGCCCCCAACGGTCACTACATTTTGTCTCGGGGTGGCAATGGTCACCGCTACACCCCAGGAACAGGCTTGGGCACGAGTCCAGCCTTGGCTGGGGATGAAGCATCCAGTGCTGCGGATCTGGATAATCGCTTCCGAAAGAGAACTCACTCTGCAGGCACATCCCCTACCATTACCCACCAGAAGACCCCATCCCAGTCCTCAGTGGCTTCCATTGAGGAGTATACAGAGATGATGCCTGCCTACCCACCAGGAGGTGGCAGTGGAGGCCGACTGCCGGGACACAGGCACTCTGCCTTCGTGCCCACCCACTCCTACCCAGAGGAGGGTCTGGAAATGCACCCCTTGGAGCGTCGGGGGGGCCACCACCGCCCAGACAGCTCTACCCTCCACACTGATGATGGCTACATGCCCATGTCCCCAGGGGTGGCCCCAGTGCCCAGCACCCGAAAGGGCAGTGGAGACTATATGCCCATGAGCCCCAAGAGCGTGTCTGCCCCACAGCAGATCATCAATCCCATCAGACGCCATCCCCAGAGAGTGGACCCCAATGGCTACATGATGATGTCCCCCAGCGGTGGCTGCTCTCCTGACATTGGAAGTGGCCCCAGCAGCAGCAGCAGCAGTGCCGTCCCTTCCGGGAGCAGCTATGGAAAGCTGTGGACAAACGGGGTAGGGGGCCACCACTCTCATGTCTTGCCTCACCCCAAACCCCCAGTGGAGAGCAGCGGTGGCAAGCTCTTACCTTGCACAGGTGACTACATGAACATGTCACCAGTGGGGGACTCCAACACCAGCAGCCCCTCCGACTGCTACTATGGCCCTGAGGACCCGCAGCACAAGCCAGTCCTCTCGTACTACTCATTGCCAAGATCCTTTAAGCACACCCAGCGCCCTGGGGAGCCGGAGGAGGGTGCCCGGCATCAGCACCTCCGCCTTTCCGCTAGCTCTGGTCGCCTTCTCTATGCTGCAACAGCGGATGATTCTTCCTCCTCTACCAGCAGCGACAGCCTGGGTGGGGGATACTGTGGGGCTAGGCTGGAGCCCAGCCTTCCACATCCCCACCATCAGGTTCTGCAGCCCCATCTGCCTCGAAAGGTGGACACAGCTGCTCAGACCAATAGCCGCCTGGCCCGGCCTACGAGGCTGTCCCTGGGGGATCCCAAGGCCAGCACCTTACCTCGGGCCCGAGAGCAGCAGCAGCAGCAGCAGCAGCAACAGCAGCAACAGCAGCCCCTGCTGCACCCTCCGGAGCCCAAGAGCCCAGGGGAATATGTGAATATTGAATTTGGGAGTGATCAGCCTGGCTACTTGTCTGGCCCGGTGGCTTCCCGTAGCTCACCTTCTGTCAGGTGTCCATCCCAGCTCCAGCCAGCTCCCAGAGAGGAAGAGACTGGCACTGAGGAGTACATGAAGATGGACCTGGGGCCGGGCCGGAGGGCAGCCTGGCAGGAGAGCACTGGGGTCGAGATGGGCAGACTGGGCCCCGCACCTCCCGGGGCTGCTAGCATTTGCAGGCCTACCCGGGCAGTGCCCAGCAGCCGGGGTGACTATATGACCATGCAGATGAGTTGTCCCCGCCAGAGCTATGTGGACACCTCGCCAATTGCCCCTGTAAGCTATGCTGACATGCGGACAGGCATTGCTGCAGAGGAGGTGAGCCTGCCCAGGGCCACCATGGCTGCTGCCTCCTCATCCTCAGCAGCCTCTGCTTCCCCCACTGGGCCTCAAGGGGCAGCAGAGCTGGCTGCCCACTCATCCCTGCTGGGCGGCCCACAAGGACCTGGGGGCATGAGCGCCTTCACCCGGGTGAACCTCAGTCCTAACCGCAACCAGAGTGCCAAAGTGATCCGTGCAGACCCACAAGGGTGCCGGAGGAGGCATAGCTCCGAGACCTTCTCCTCAACACCCAGTGCCACCCGGGTGGGCAACACAGTGCCCTTTGGAGCCGGGGCAGCAATAGGGGGTGGTGGCGGTAGCAGCAGCAGCAGCGAGGATGTGAAACGTCACAGCTCTGCTTCCTTTGAGAATGTGTGGCTGAGGCCTGGGGAACTTGGGGGAGCCCCCAAGGAGCCAGCCCAACTGTGTGGGGCTGCTGGGGGTTTGGAGAATGGTCTTAACTACATAGATCTGGATTTGGTCAAAGACTTCAAACAGCGCCCTCAGGAGTGCACCCCTCAACCGCAGCCTCCCCCACCCCCACCCCCTCATCAACCCCTGGGTAGCAGTGAGAGCAGCTCCACCCGCCGCCGCTCAAGTGAGGATTTAAGCGCCTATGCCAGCATCAGTTTCCAGAAGCAGCCAGAGGACCTTCAGTAG

>Chl_sa1 [African green monkey (Chlorocebus sabaeus) Irs1]

ATGGCGAGCCCTCCGGAGAGCGATGGCTTCTCGGACGTGCGCAAGGTGGGCTACCTGCGCAAACCCAAGAGCATGCACAAACGCTTCTTCGTACTGCGCGCGGCCAGCGAGGCTGGGGGCCCGGCGCGGCTCGAGTACTACGAGAACGAGAAGAAGTGGCGGCACAAGTCGAGCGCCCCCAAACGCTCGATCCCCCTTGAGAGCTGCTTCAACATCAACAAGCGGGCTGACTCCAAGAACAAGCACCTGGTGGCTCTCTACACCCGGGACGAGCACTTTGCCATCGCGGCGGACAGCGAGGCGGAGCAAGACAGCTGGTACCAGGCTCTCCTACAGCTGCACAACCGTGCCAAGGGCCACCACGACGGAGCTGCGGCCCTCGGGGCGGGAGGTGGCGGGGGCAGCTGCAGCGGCAGCTCCGGCCTTGGTGAGGCTGGGGAGGACTTGAGCTACGGTGACGTGCCCCCAGGACCCGCGTTCAAAGAGGTCTGGCAGGTGATCCTGAAGCCCAAGGGCCTGGGTCAGACAAAGAACCTGATTGGTATCTACCGCCTCTGCCTGACCAGCAAGACCATCAGCTTCGTGAAGCTGAACTCGGAGGCAGCCGCCGTGGTGCTGCAGCTGATGAACATCAGGCGCTGCGGCCACTCGGAGAACTTCTTCTTCATCGAGGTGGGCCGTTCTGCCGTGACGGGGCCCGGGGAGTTCTGGATGCAGGTGGATGACTCCGTGGTGGCCCAGAACATGCATGAGACCATCCTGGAGGCCATGCGGGCTATGAGCGATGAGTTTCGCCCTCGCAGCAAGAGCCAGTCCTCGTCCAACTGCTCCAACCCCATCAGCGTCCCCCTGCGCCGGCACCATCTCAACAACCCCCCGCCCAGTCAGGTGGGGCTGACCCGCCGATCTCGCACTGAGAGCATCACCGCCACCTCCCCGGCCAGCATGGTGGGCGGGAAGCCAGGCTCCTTCCGCGTCCGCGCCTCCAGTGACGGCGAAGGCACCATGTCCCGCCCAGCCTCGGTGGATGGCAGCCCTGTGAGTCCGAGCACCAACAGAACCCACGCCCACCGGCATCGGGGCAGCGCCCGGCTGCATCCCCCGCTCAACCACAGCCGCTCCATCCCCATGCCAGCTTCCCGCTGCTCGCCTTCGGCCACCAGCCCCGTCAGTCTGTCGTCCAGCAGCACCAGCGGCCATGGCTCCACCTCGGATTGTCTCTTCCCACGGCGCTCTAGTGCTTCGGTGTCTGGTTCTCCCAGCGATGGCGGTTTCATCTCCTCAGATGAGTATGGCTCCAGTCCCTGCGATTTCCGGAGTTCCTTCCGCAGTGTCACTCCGGATTCCCTGGGCCACACCCCACCAGCCCGCGGTGAGGAGGAGCTAAGCAACTATATCTGCATGGGCGGCAAGGGGCCCTCCACCCTGACCGCCCCCAACGGTCACTACATTTTGTCTCGGGGTGGCAATGGCCACCGCTACACCCCAGGAACAGGCTTGGGCACGAGTCCAGCCTTGGCTGGGGATGAAGCATCCAGTGCTGCGGATCTGGATAATCGCTTCCGAAAGAGAACTCACTCTGCAGGCACATCCCCTACCATTACCCACCAGAAGACCCCGTCCCAGTCCTCAGTGGCTTCCATTGAGGAGTACACAGAGATGATGCCTGCCTACCCACCAGGAGGTGGCAGTGGAGGCCGACTGCCGGGACACAGGCACTCTGCCTTCGTGCCCACCCACTCCTACCCAGAGGAGGGTCTGGAAATGCACCCCTTGGAGCGTCGGGGGGGCCACCACCGCCCAGACAGCTCTACCCTCCACACTGATGATGGCTACATGCCCATGTCCCCAGGGGTGGCCCCAGTGCCCAGCAGCCGAAAGGGCAGTGGAGACTATATGCCCATGAGCCCCAAGAGCGTGTCTGCCCCACAGCAGATCATCAATCCCATCAGACGCCATCCCCAGAGAGTGGACCCCAATGGCTACATGATGATGTCCCCCAGCGGTGGCTGCTCTCCTGACATTGGAGGTGGCCCCAGCAGCAGCAGCAGCAGCACCGTCCCTTCCGGGAGCAGCTATGGAAAGCTGTGGACAAACGGGGTAGGGGGCCACCACTCTCAGGTCTTGCCTCACCCCAAACCCCCAGTGGAGAGCAGCGGTGGCAAGCTCTTACCTTGCACAGGTGACTACATGAACATGTCACCAGTGGGGGACTCCAACACCAGCAGCCCCTCCGACTGCTACTATGGCCCTGAGGACCCCCAGCACAAGCCAGTCCTCTCGTACTACTCATTGCCAAGATCCTTTAAGCACACCCAGCGCCCCGGGGAGCCGGAGGAGGGTGCCCGGCATCAGCACCTCCACCTTTCCGCTAGCTCTGGTCGCCTTCTCTATGCTGCAACAGCGGATGATTCTTCCTCCTCCACCAGCAGCGACAGCCTGGGTGGGGGATACTGTGGGGCTAGGCTGGAGCCCAGCCTTCCACATCCCCACCATCAGGTTCTGCAGCCCCATCTGCCTCGAAAGGTGGACACAGCTGCTCAGACCAATAGCCGCCTGGCCCGGCCTACGAGGCTGTCCCTGGGGGATCCCAAGGCCAGCACCTTACCTCGGGCCCGAGAGCAGCAGCATCAGCAGCAGCAGCAGCAACAGCAGCAACAGCAACAGCAGCCCCTGCTGCACCCTCCGGAGCCCAAGAGCCCAGGGGAATATGTGAATATTGAATTTGGGAGTGATCAGCCTGGCTACTTGTCTGGCCCAGTGGCTTCCCGTAGCTCACCTTCTGTCAGGTGTCCATCCCAGCTCCAGCCAGCTCCCAGAGAGGAGGAGACTGGCACTGAGGAGTACATGAAGATGGACCTGGGGCCGGGCCGGAGGGCAGCCTGGCAGGAGAGCACTGGGGTCGAGATGGGCAGACTGGGCCCCGCACCTCCCGGGGCTGCTAGCATTTGCAGGCCTACCCGGGCAGTGCCCAGCAGCCGGGGTGACTATATGACCATGCAGATGAGTTGTCCCCGCCAGAGCTATGTGGACACTTCGCCAATTGCCCCTGTAAGCTATGCTGACATGCGGACAGGCATTGCTGCAGAGGAGGTGAGCCTGCCCAGGGCCACCATGGCTGCTGCCGCCTCATCCTCAGCAGCCTCTGCTTCCCCCACTGGGCCTCAAGGGGCAGCAGAGCTGGCTGCCCACTCATCCCTGCTGGGGGGCGCACAAGGACCTGGGGGCATGAGTGCCTTCACCCGGGTGAACCTCAGTCCTAACCGCAACCAGAGTGCCAAAGTGATCCGTGCAGACCCACAAGGGTGCCGGAGGAGGCATAGCTCCGAGACCTTCTCCTCAACACCCAGTGCCACCCGGGTGGGCAACACAGTGCCCTTTGGAGCCGGGGCAGCAATAGGGGGCAGTGGCGGTAGCAGCAGCAGCAGCGAGGATGTGAAACGCCACAGCTCTGCTTCCTTTGAGAATGTGTGGCTGAGGCCTGGGGAACTTGGGGGAGCCCCCAAGGAGCCAGCCCAACTGTGTGGGGCTGCTGGGGGTTTGGAGAATGGTCTTAACTACATAGATCTGGATTTGGTCAAGGACTTCAAACAGCGCCCTCAGGAGTGCACCCCTCAGCCGCAGCCTCCTCCACCCCCACCCCCTCATCAACCCCTGGGTAGCAGTGAGAGCAGCTCTACCCGCCGCTCAAGTGAGGATTTAAGCGCCTATGCCAGCATCAGTTTCCAGAAGCAGCCAGAGGACCTTCAGTAG

>Mac_ne1 [Pig-tailed macaque (Macaca nemestrina) Irs1]

ATGGCGAGCCCTCCGGAGAGCGATGGCTTCTCGGACGTGCGCAAGGTGGGCTACCTGCGCAAACCCAAGAGCATGCACAAACGCTTCTTCGTACTGCGCGCGGCCAGCGAGGCTGGGGGCCCGGCGCGCCTCGAGTACTACGAGAACGAGAAGAAGTGGCGGCACAAGTCGAGCGCCCCCAAACGCTCGATCCCCCTTGAGAGCTGCTTCAACATCAACAAGCGGGCTGACTCCAAGAACAAGCACCTGGTGGCTCTCTACACCCGGGACGAGCACTTTGCCATCGCGGCGGACAGCGAGGCGGAGCAAGACAGCTGGTACCAGGCTCTCCTACAGCTGCACAACCGTGCCAAGGGCCACCACGACGGAGCTGCGGCCCTCGGGGCGGGAGGTGGCGGGGGCAGCTGCAGTGGCAGCTCCGGCGTTGGTGAGGCTGGGGAGGACTTGAGCTACGGTGACGTGCCCCCAGGACCCGCGTTCAAAGAGGTCTGGCAGGTGATCCTGAAGCCCAAGGGCCTGGGTCAGACAAAGAACCTGATTGGTATCTACCGCCTCTGCCTGACCAGCAAGACCATCAGCTTCGTGAAGCTGAACTCGGAGGCAGCCGCCGTGGTGCTGCAGCTGATGAACATCAGGCGCTGCGGCCACTCGGAGAACTTCTTCTTCATCGAGGTGGGCCGTTCTGCCGTGACGGGGCCCGGGGAGTTCTGGATGCAGGTGGATGACTCCGTGGTGGCCCAGAACATGCACGAGACCATCCTGGAGGCCATGCGGGCTATGAGCGATGAGTTCCGCCCTCGCAGCAAGAGCCAGTCCTCGTCCAACTGCTCCAACCCCATCAGCGTCCCCCTGCGCCGGCACCATCTCAACAACCCCCCGCCCAGCCAGGTGGGGCTGACCCGACGATCTCGCACTGAGAGCATCACTGCCACCTCCCCGGCCAGCATGGTGGGCGGGAAACCAGGCTCCTTCCGCGTCCGCGCCTCCAGTGACGGCGAAGGCACCATGTCCCGCCCAGCCTCGGTGGATGGCAGCCCTGTGAGTCCGAGCACCAACAGAACCCACGCCCACCGGCATCGGGGCAGCGCCCGGCTGCACCCCCCGCTCAACCACAGCCGCTCCATCCCCATGCCAGCTTCCCGCTGCTCGCCTTCGGCCACCAGCCCCGTCAGTCTGTCGTCCAGCAGCACCAGCGGCCATGGCTCCACCTCGGATTGTCTCTTCCCACGGCGATCTAGTGCTTCGGTGTCTGGTTCTCCCAGCGATGGCGGTTTCATCTCCTCAGATGAGTATGGCTCCAGTCCCTGCGATTTCCGGAGTTCCTTCCGCAGTGTCACTCCGGATTCCCTGGGCCACACCCCACCAGCCCGCGGTGAGGAGGAGCTAAGCAACTATATCTGCATGGGCGGCAAGGGGCCCTCCACCCTGACCGCCCCCAACGGTCACTACATTTTGTCTCGGGGTGGCAATGGCCACCGCTACACCCCAGGAACAGGCTTGGGCACGAGTCCAGCCTTGGCTGGGGATGAAGCATCCAGTGCTGCGGATCTGGATAATCGCTTCCGAAAGAGAACTCACTCTGCAGGCACATCCCCTACCATTACCCACCAGAAGACCCCGTCCCAGTCCTCAGTGGCTTCCATTGAGGAGTATACAGAGATGATGCCTGCCTACCCACCAGGAGGTGGCAGTGGAGGCCGACTGCCGGGACACAGGCACTCTGCCTTCGTGCCCACCCACTCCTACCCAGAGGAGGGTCTGGAAATGCACCCCTTGGAGCGTCGGGGGGGCCACCACCGCCCAGACAGCTCTACCCTCCACACTGATGATGGCTACATGCCCATGTCCCCAGGGGTGGCCCCAGTGCCCAGCAGCCGAAAGGGCAGTGGAGACTATATGCCCATGAGCCCCAAGAGCGTGTCTGCCCCACAGCAGATCATCAATCCCATCAGACGCCATCCCCAGAGAGTGGACCCCAATGGCTACATGATGATGTCCCCCAGCGGTGGCTGCTCTCCTGACATTGGAGGTGGCCCCAGCAGCAGCAGCAGCAGTGCCGTCCCTTCTGGGAGCAGCTATGGAAAGCTGTGGACAAACGGGGTAGGGGGCCACCACTCTCATGTCTTGCCTCACCCCAAACCCCCAGTGGAGAGCAGTGGTGGCAAGCTCTTACCTTGCACAGGTGACTACATGAACATGTCACCAGTGGGGGACTCCAACACCAGCAGCCCCTCTGACTGCTACTATGGCCCTGAGGACCCCCAGCACAAGCCAGTCCTCTCGTACTACTCATTGCCAAGATCCTTTAAGCACACCCAGCGCCCTGGGGAGCCGGAGGAGGGTGCCCGGCATCAGCACCTCCGCCTTTCTGCTAGCTCTGGTCGCCTTCTCTATGCTGCAACAGCGGATGATTCTTCCTCCTCCACCAGCAGCGACAGCCTGGGTGGGGGATACTGTGGGGCTAGGCTGGAGCCCAGCCTTCCACATCCCCACCATCAGGTTCTGCAGCCCCATCTGCCTCGAAAGGTGGACACAGCTGCTCAGACCAATAGCCGCCTGGCCCGGCCTACGAGGCTGTCCCTGGGGGATCCCAAGGCCAGCACCTTACCTCGGGCCCGAGAGCAGCAGCAGCAGCAGCAGCAGCAGCAGCAACAGCAGCAACAGCAGCCCCTGCTGCACCCTCCGGAGCCCAAGAGCCCAGGAGAATATGTGAATATTGAATTTGGGAGTGATCAGCCTGGCTACTTGTCTGGCCCGGTGGCTTCCCGTAGCTCACCTTCTGTCAGGTGTCCATCCCAGCTCCAGCCAGCTCCCAGAGAGGAAGAGACTGGCACTGAGGAGTACATGAAGATGGACCTGGGGCCGGGCCGGAGGGCAGCCTGGCAGGAGAGCACTGGGGTCGAGATGGGCAGACTGGGCCCCGCACCTCCCGGGGCTGCTAGCATTTGCAGGCCTACCCGGGCAGTGCCCAGCAGCCGGGGTGACTATATGACCATGCAGATGAGTTGTCCCCGCCAGAGCTATGTGGACACCTCGCCAATTGCCCCTGTAAGCTATGCTGACATGCGGACAGGCATTGCTGCAGAGGAGGTGAGCCTGCCCAGGGCCACCATGGCTGCTGCCTCCTCATCCTCAGCAGCCTCTGCTTCCCCCACTGGGCCTCAAAGGGCAGCAGAGCTGGCTGCCCACTCATCCCTGCTGGGGGGCCCACAAGGACCTGGGGGCATGAGCGCCTTCACCCGGGTGAACCTCAGTCCTAACCGCAACCAGAGTGCCAAAGTGATCCGTGCAGACCCACAAGGGTGCCGGAGGAGGCATAGCTCCGAGACCTTCTCCTCAACACCCAGTGCCACCCGGGTGGGCAACACAGTGCCCTTTGGAGCCGGGGCAGCAATAGGGGGTGGTGGCGGTAGCAGCAGCAGCAGCGAGGATGTGAAACGTCACAGCTCTGCTTCCTTTGAGAATGTGTGGCTGAGGCCTGGGGAACTTGGGGGAGCCCCCAAGGAGCCAGCCCAACTGTGTGGGGCTGCTGGGGGTTTGGAGAATGGTCTTAACTACATAGATCTGGATTTGGTCAAGGACTTCAAACAGCGCCCTCAGGAGTGCACCCCTCAACCGCAGCCTCCCCCACCCCCACCCCCTCATCAACCCCTGGGTAGCAGTGAGAGCAGCTCCACCCGCCGCCACTCAAGTGAGGATTTAAGCGCCTATGCCAGCATCAGTTTCCAGAAGCAGCCAGAGGACCTTCAGTAG

>Col_an1 [Black and white colobus (Colobus angolensis palliatus) Irs1]

ATGGCGAGCCCTCCTGAGAGTGATGGCTTCTCGGACGTGCGCAAGGTGGGCTACCTGCGCAAACCCAAGAGCATGCACAAACGCTTCTTCGTACTGCGTGCGGCCAGCGAGGCTGGGGGCCCGGCGCGCCTCGAGTACTACGAGAACGAGAAGAAGTGGCGGCACAAGTCGAGCGCCCCCAAACGCTCGATCCCCCTTGAGAGCTGCTTCAACATCAACAAGCGGGCTGACTCCAAGAACAAGCACCTGGTGGCTCTCTACACCCGGGACGAGCACTTTGCCATTGCGGCGGACAGCGAGGCGGAGCAAGACAGCTGGTACCAGGCTCTCCTACAGCTACACAACCGTGCCAAGGGTCACCACGACGGAGCTGCGGCCCTCGGGGCGGGAGGTGGCGGGGGCAGCTGCAGCGGCAGCTCCGGCCTTGGTGAGGCTGGGGAGGACTTGAGCTACGGTGACGTGCCCCCAGGACCCGCGTTCAAAGAGGTCTGGCAGGTGATCCTGAAGCCCAAGGGCCTGGGTCAGACAAAGAACCTGATTGGTATCTACCGCCTCTGCCTGACCAGCAAGACCATCAGCTTCGTGAAGCTGAACTCGGAGGCAGCCGCCGTGGTGCTGCAGCTGATGAACATCAGGCGCTGCGGCCACTCGGAGAACTTCTTCTTCATCGAGGTGGGCCGTTCTGCCGTGACGGGGCCCGGGGAGTTCTGGATGCAGGTGGATGACTCCGTGGTGGCCCAGAACATGCACGAGACCATCCTGGAGGCCATGCGGGCTATGAGCGATGAGTTCCGCCCTCGCAGCAAGAGCCAGTCCTCGTCCAACTGCTCCAACCCCATCAGCGTCCCCCTGCGCCGGCACCATCTCAACAACCCCCCGCCCAGCCAGGTGGGGCTGACCCGCCGATCTCGCACTGAGAGCATCACCGCCACCTCCCCGGCCAGCATGGTGGGCGGGAAGCCAGGCTCCTTCCGCGTCCGCGCCTCCAGTGACGGCGAAGGCACCATGTCCCGCCCAGCCTCGGTGGACGGCAGCCCTGTGAGTCCGAGCACCAACAGAACCCACGCCCACCGGCATCGGGGCAGCGCCCGGCTGCACCCCCCGCTCAACCACAGCCGCTCCATCCCCATGCCAGCTTCCCGCTGCTCGCCTTCGGCCACCAGCCCCGTCAGTCTGTCTTCCAGCAGCACCAGTGGCCATGGCTCCACCTCGGATTGTCTCTTCCCACGGCGATCTAGTGCTTCGGTGTCTGGTTCCCCCAGCGATGGCGGTTTCATCTCCTCAGATGAGTATGGCTCCAGTCCCTGCGATTTCCGGAGTTCCTTCCGCAGTGTCACTCCGGATTCCCTGGGCCACACCCCACCAGCCCGCGGTGAGGAGGAGCTAAGCAACTATATCTGCATGGGCGGCAAGGGGCCCTCCACCCTGACCGCCCCCAATGGTCACTACATTTTGTCTCGGGGTGGCAATGGTCACCGCTACACCCCAGGAACAGGCTTGGGCACGAGTCCAGCCTTGGCTGGGGATGAAGCATCCAGTGCTGCGGATCTGGATAATCGCTTCCGAAAGAGAACTCACTCTGCAGGCACATCCCCTACCATTACCCACCAGAAGACCCCGTCCCAGTCCTCAGTGGCTTCCATTGAGGAGTATACAGAGATGATGCCTGCCTACCCACCAGGAGGTGGCAGTGGAGGCCGACTGCCGGGACACAGGCACTCTGCCTTCGTGCCCACCCACTCCTACCCAGAGGAGGGTCTGGAAATGCACCCCTTGGAGCGTCGGGGGGGCCACCACCGCCCAGACAGCTCTACCCTCCACACTGATGATGGCTACATGCCCATGTCCCCAGGGGTAGCCCCAGTGCCCAGCAGCCGAAAGGGCAGTGGAGACTATATGCCCATGAGCCCCAAGAGCGTGTCTGCCCCACAGCAGATCATCAATCCCATCAGACGCCATCCCCAGAGAGTGGACCCCAATGGCTACATGATGATGTCCCCCAGCGGCGGCTGCTCTCCTGACATTGGAGGTGGCCCCAGCAGCAGCAGCAGCAGTGCCATCCCTTCTGGGAGCAGCTATGGAAAGCTGTGGACAAACGGGGTAGGGGGCCACCACTCTCATGTCTTGCCTCACCCCAAACCCCCAGTGGAGAGCAGCAGTGGCAAGCTCTTACCTTGCACAGGTGACTACATGAACATGTCACCAGTGGGGGACTCCAACACCAGCAGCCCCTCCGACTGCTACTATGGCCCTGAGGACCCCCAGCACAAGCCAGTCCTCTCGTACTACTCATTGCCAAGATCCTTTAAGCACACTCAGCGCCCCGGGGAGCTGGAGGAGGGTGCCCGGCATCAGCATCTCCGCCTTTCCACTAGCTCTGGTCGCCTTCTCTATGCTGCAACAGCAGATGATTCTTCCTCCTCCACCAGCAGTGACAGCCTGGGTGGGGGATACTGTGGGGCTAGGCTGGAGCCCAGCCTTCCACATCCCCACCATCAGGTTCTGCAGCCCCATCTGCCTCGAAAGGTGGACACAGCTGCTCAGACCAATAGCCGCCTGGCCCGGCCTACAAGGCTGTCCCTGGGGGATCCCAAGGCCAGCACCTTACCTCGGGCCCGAGAGCAGCAGCAGCAGCAACAGCAGCAGCCCCTGCTGCACCCTCCAGAGCCCAAGAGCCCAGGGGAATATGTGAATATTGAATTTGGGAGTGATCAGCCTGGCTACTTGTCTGGTCCGGTGGCTTCCCGTAGCTCACCTTCTGTCAGGTGTCCATCCCAGCTCCAGCCAGCTCCCAGAGAGGAAGAGACTGGCACTGAGGAGTACATGAAGATGGACCTGGGGCCGGGCCGGAGGGCAGCCTGGCAGGAGAGCACTGGGGTCGAGATGGGCAGACTGGGCCCCGCACCTCCCGGGGCTGCTAGCATTTGCAGGCCTACCCGGGCAGTGCCCAGCAGCCGGGGTGACTATATGACCATGCAGATGAGTTGTCCCCGCCAGAGCTATGTGGACACCTCGCCAATTGCCCCTGTAAGCTATGCTGACATGCGGACAGGCATTGCTGCAGAGGAGGTGAGCCTGCCCAGGGCCACCATGGCTGCTGCCTCCTCATCCTCAGCAGCCTCTGCTTCCCCCACTGGGCCTCAAGGGGCAGCAGAGCTGGCTGCCCACTCATCCCTGCTGGGGGGCACACAAGGACCTGGGGGCATGAGCGCCTTCACCCGGGTGAACCTCAGTCCTAACCGCAACCAGAGTGCCAAAGTGATCCGTGCAGACCCACAAGGGTGCCGGAGGAGGCACAGCTCCGAGACCTTCTCCTCAACACCCAGTGCCACCCGGGTGGGCAACACAGTGCCCTTTGGAGCCGGGACAGCAATAGGGGGCGGTGGCGGTAGCAGCAGCAGCAGCGAGGATGTGAAACGCCACAGCTCTGCTTCCTTTGAGAATGTGTGGCTGAGGCCTGGGGAACTTGGGGGAGCCCCCAAGGAGCCAGCCCAACTGTGTGGGGCTGCTGGGGGTTTGGAGAATGGTCTTAACTACATAGATCTGGATTTGGTCAAGGATTTCAAACAGCGCCCTCAGGAGTGCACCCCTCAACCGCAGCCTCCCCCACCCCCACCCCCTCATCAACCCCTGAGTAGCAGTGAGAGCAGCTCCACCCGCCGCGGCTCAAGTGAGGATTTAAGCGCCTATGCCAGCATCAGTTTCCAGAAGCAGCCAGAAGACCTTCAGTAG

>Cer_at1 [Sooty mangabey (Cercocebus atys) Irs1]

ATGGCGAGCCCTCCGGAGAGCGATGGCTTCTCGGACGTGCGCAAGGTGGGCTACCTGCGCAAACCCAAGAGCATGCACAAACGCTTCTTCGTACTGCGCGCGGCCAGCGAGGCTGGGGGCCCGGCGCGCCTCGAGTACTACGAGAACGAGAAGAAGTGGCGGCACAAGTCGAGCGCCCCCAAACGCTCGATCCCCCTTGAGAGCTGCTTCAACATCAACAAGCGGGCTGACTCCAAGAACAAGCACCTGGTGGCTCTCTACACCCGGGACGAGCACTTTGCCATCGCGGCGGACAGCGAGGCGGAGCAAGACAGCTGGTACCAGGCTCTCCTACAGCTGCACAACCGTGCCAAGGGCCACCACGACGGAGCTGCGGCCCTCGGGGCGGGAGGTGGCGGGGGCAGCTGCAGTGGCAGCTCCGGCCTTGGTGAGGCTGGGGAGGACTTGAGCTACGGTGACGTGCCCCCAGGACCCGCGTTCAAAGAGGTCTGGCAGGTGATCCTGAAGCCCAAGGGCCTGGGTCAGACAAAGAACCTGATTGGTATCTACCGCCTCTGCCTGACCAGCAAGACCATCAGCTTCGTGAAGCTGAACTCGGAGGCAGCCGCCGTGGTGCTGCAGCTGATGAACATCAGGCGCTGCGGCCACTCGGAGAACTTCTTCTTCATCGAGGTGGGCCGTTCTGCCGTGACGGGGCCCGGGGAGTTCTGGATGCAGGTGGATGACTCCGTGGTGGCCCAGAACATGCACGAGACCATCCTGGAGGCCATGCGGGCTATGAGCGATGAGTTCCGCCCTCGCAGCAAGAGCCAGTCCTCGTCCAACTGCTCCAACCCCATCAGCGTCCCCCTGCGCCGGCACCATCTCAACAATCCCCCGCCCAGCCAGGTGGGGCTGACCCGACGATCTCGCACTGAGAGCATCACTGCCACCTCCCCGGCCAGCATGGTGGGCGGGAAACCAGGCTCCTTCCGCGTCCGCGCCTCCAGTGACGGTGAAGGCACCATGTCCCGCCCAGCCTCGGTGGATGGCAGCCCTGTGAGTCCGAGCACCAACAGAACCCACGCCCACCGGCATCGGGGCAGTGCCCGGCTGCACCCCCCGCTCAACCACAGCCGCTCCATCCCCATGCCAGCTTCCCGCTGCTCGCCTTCGGCCACCAGCCCCGTCAGTCTGTCGTCCAGCAGCACCAGCGGCCATGGCTCCACCTCGGATTGTCTCTTCCCACGGCGATCTAGTGCTTCGGTGTCTGGTTCTCCCAGCGATGGCGGTTTCATCTCCTCAGATGAGTATGGCTCCAGTCCCTGCGATTTCCGGAGTTCCTTCCGCAGTGTCACTCCGGATTCCCTGGGCCACACCCCACCAGCCCGCGGTGAGGAGGAGCTAAGCAACTATATCTGCATGGGCGGCAAGGGGCCCTCCACCCTGACCGCCCCCAACGGTCACTACATTTTGTCTCGGGGTGGCAATGGTCACCGCTACACCCCAGGAACAGGCTTGGGCACGAGTCCAGCCTTGGCTGGGGATGAAGCATCCAGTGCTGCGGATCTGGATAATCGCTTCCGAAAGAGAACTCACTCTGCAGGCACATCCCCTACCATTACCCACCAGAAGACCCCGTCCCAGTCCTCAGTGGCTTCCATTGAGGAGTATACAGAGATGATGCCTGCCTACCCACCAGGAGGTGGCAGTGGAGGCCGACTGCCGGGACACAGGCACTCTGCCTTCGTGCCCACCCACTCCTACCCAGAGGAGGGTCTGGAAATGCACCCCTTGGAGCGTCGGGGGGGCCACCACCGCCCAGACAGCTCTACCCTCCACACTGATGATGGCTACATGCCCATGTCCCCAGGGGTGGCCCCAGTGCCCAGCAGCCGAAAGGGCAGTGGAGACTATATGCCCATGAGCCCCAAGAGCGTGTCTGCCCCACAGCAGATCATCAATCCCATCAGACGCCATCCCCAGAGAGTGGACCCCAATGGCTACATGATGATGTCCCCCAGCGGTGGCTGCTCTCCTGACATTGGAGGTGGCCCCAGCAGCAGCAGCAGCAGTGCTGTCCCTTCCGGGAGCAGCTATGGAAAGCTGTGGACAAACGGGGTAGGGGGCCACCATTCTCATGTCTTGCCTCACCCCAAACCCCCAGTGGAGAGCAGCAGTGGCAAGCTCTTACCTTGCACAGGTGACTACATGAACATGTCACCAATGGGGGACTCCAACACCAGCAGCCCCTCCGACTGCTACTATGGCCCTGAGGACTCGCAGCACAAGCCAGTCCTCTCGTACTACTCATTGCCAAGATCCTTTAAGCACACCCAGCGCCCTGGGGAGCCGGAGGAGGGTGCCCGGCATCAGCACCTCCGCCTTTCCGCTAGCTCTGGTCGCCTTCTCTATGCTGCAACAGCGGATGATTCTTCCTCCTCCACCAGCAGCGACAGCCTGGGTGGGGGATACTGTGGGGCTAGGCTGGAGCCCAGCCTTCCACATTCCCACCATCAGGTTCTGCAGCCCCATCTGCCTCGAAAGGTGGACACAGCTGCTCAGACCAATAGCCGCCTGGCCCGGCCTACGAGGCTGTCCCTGGGGGATCCCAAGGCCAGCACCTTACCTCGGGCCCGAGAGCAGCAGCAGCAGCAGCAGCAGCAGCAGCAGCAGCAGCAACAGCAGCAACAGCAGCCCCTGCTGCACCCTCCGGAGCCCAAGAGCCCAGGGGAATATGTGAATATTGAATTTGGGAGTGATCAGCCTGGCTACTTGTCTGGCCCGGTGGCTTCCCGTAGCTCACCTTCTGTCAGGTGTCCATCCCAGCTCCAGCCAGCTCCCAGAGAGGAAGAGACTGGCACTGAGGAGTACATGAAGATGGACCTGGGGCCGGGCCGGAGGGCAGCCTGGCAGGAGAGCACTGGGGTCGAGATGGGCAGACTGGGCCCCGCACCTCCCGGGGCTGCTAGCATTTGCAGGCCTACCCGGGCAGTGCCCAGCAGCCGGGGTGACTATATGACCATGCAGATGAGTTGTCCCCGCCAGAGCTATGTGGACACCTCGCCAATTGCCCCTGTAAGCTATGCTGACATGCGGACAGGCATTGCTGCAGAGGAGGTGAGCCTGCCCAGGGCCACCATGGCTGCTGCCTCCTCATCCTCAGCAGCCTCTGCTTCCCCCACTGGGCCTCAAGGGGCAGCAGAGCTGGCTGCCCACTCATCCCTGCTGGGGGGCCCACAAGGACCTGGGGGCATGAGCGCCTTCACCCGGGTGAACCTCAGTCCTAACCGCAACCAGAGTGCCAAAGTGATCCGTGCAGACCCACAAGGGTGCCGGAGGAGGCATAGCTCCGAGACCTTCTCCTCAACACCCAGTGCCACCCGGGTGGGCAACACAGTGCCCTTTGGAGCCGGGGCAGCAATAGGGGGTGGTGGCGGTAGCAGCAGCAGCAGCGAGGATGTGAAACGTCACAGCTCTGCTTCCTTTGAGAATGTGTGGCTGAGGCCTGGGGAACTTGGGGGAGCCCCCAAGGAGCCAGCCCAACTGTGTGGGGCTGCTGGGGGTTTGGAGAATGGTCTTAACTACATAGATCTGGATTTGGTCAAGGACTTCAAACAGCACCCTCAGGAGTGCACCCCTCAACCGCAGCCTCCCCCACCCCCACCCCCTCATCAACCCCTGGGTAGCAGTGAGAGCAGCTCCACCCCCCGCCGCTCAAGTGAGGATTTAAGCGCCTATGCCAGCATCAGTTTCCAGAAGCAGCCAGAGGACCTTCAGTAG

>Mac_fa1 [Crab-eating macaque (Macaca fascicularis) irs1]

ATGGCGAGCCCTCCGGAGAGCGATGGCTTCTCGGACGTGCGCAAGGTGGGCTACCTGCGCAAACCCAAGAGCATGCACAAACGCTTCTTCGTACTGCGCGCGGCCAGCGAGGCTGGGGGCCCGGCGCGCCTCGAGTACTACGAGAACGAGAAGAAGTGGCGGCACAAGTCGAGCGCCCCCAAACGCTCGATCCCCCTTGAGAGCTGCTTCAACATCAACAAGCGGGCTGACTCCAAGAACAAGCACCTGGTGGCTCTCTACACCCGGGACGAGCACTTTGCCATCGCGGCGGACAGCGAGGCGGAGCAAGACAGCTGGTACCAGGCTCTCCTACAGCTGCACAACCGTGCCAAGGGCCACCACGACGGAGCTGCGGCCCTCGGGGCGGGAGGTGGCGGGGGCAGCTGCAGTGGCAGCTCCGGCGTTGGTGAGGCTGGGGAGGACTTGAGCTACGGTGACGTGCCCCCAGGACCCGCGTTCAAAGAGGTCTGGCAGGTGATCCTGAAGCCCAAGGGCCTGGGTCAGACAAAGAACCTGATTGGTATCTACCGCCTCTGCCTGACCAGCAAGACCATCAGCTTCGTGAAGCTGAACTCGGAGGCAGCCGCCGTGGTGCTGCAGCTGATGAACATCAGGCGCTGCGGCCACTCGGAGAACTTCTTCTTCATCGAGGTGGGCCGTTCTGCCGTGACGGGGCCCGGGGAGTTCTGGATGCAGGTGGATGACTCCGTGGTGGCCCAGAACATGCACGAGACCATCCTGGAGGCCATGCGGGCTATGAGCGATGAGTTCCGCCCTCGCAGCAAGAGTCAGTCCTCGTCCAACTGCTCCAACCCCATCAGCGTCCCCCTGCGCCGGCACCATCTCAACAACCCCCCGCCCAGCCAGGTGGGGCTGACCCGACGATCTCGCACTGAGAGCATCACTGCCACCTCCCCGGCCAGCATGGTGGGCGGGAAACCAGGCTCCTTCCGCGTCCGCGCCTCCAGTGACGGCGAAGGCACCATGTCCCGCCCAGCCTCGGTGGATGGCAGCCCTGTGAGTCCGAGCACCAACAGAACCCACGCCCACCGGCATCGGGGCAGCGCCCGGCTGCACCCCCCGCTCAACCACAGCCGCTCCATCCCCATGCCAGCTTCCCGCTGCTCGCCTTCGGCCACCAGCCCCGTCAGTCTGTCGTCCAGCAGCACCAGCGGCCATGGCTCCACCTCGGATTGTCTCTTCCCACGGCGATCTAGTGCTTCGGTGTCTGGTTCTCCCAGCGATGGCGGTTTCATCTCCTCAGATGAGTATGGCTCCAGTCCCTGCGATTTCCGGAGTTCCTTCCGCAGTGTCACTCCGGATTCCCTGGGCCACACCCCACCAGCCCGCGGTGAGGAGGAGCTAAGCAACTATATCTGCATGGGCGGCAAGGGGCCCTCCACCCTGACCGCCCCCAACGGTCACTACATTTTGTCTCGGGGTGGCAATGGCCACCGCTACACCCCAGGAACAGGCTTGGGCACGAGTCCAGCCTTGGCTGGGGATGAAGCATCCAGTGCTGCGGATCTGGATAATCGCTTCCGAAAGAGAACTCACTCTGCAGGCACATCCCCTACCATTACCCACCAGAAGACCCCGTCCCAGTCCTCAGTGGCTTCCATTGAGGAGTATACAGAGATGATGCCTGCCTACCCACCAGGAGGTGGCAGTGGAGGCCGACTGCCGGGACACAGGCACTCTGCCTTCGTGCCCACCCACTCCTACCCAGAGGAGGGTCTGGAAATGCACCCCTTGGAGCGTCGGGGGGGCCACCACCGCCCAGACAGCTCTACCCTCCACACTGATGATGGCTACATGCCCATGTCCCCAGGGGTGGCCCCAGTGCCCAGCAGCCGAAAGGGCAGTGGAGACTATATGCCCATGAGCCCCAAGAGCGTGTCTGCCCCACAGCAGATCATCAATCCCATCAGACGCCATCCCCAGAGAGTGGACCCCAATGGCTACATGATGATGTCCCCCAGCGGTGGCTGCTCTCCTGACATTGGAGGTGGCCCCAGCAGCAGCAGCAGCAGTGCCGTCCCTTCTGGGAGCAGCTATGGAAAGCTGTGGACAAACGGGGTAGGGGGCCACCACTCTCATGTCTTGCCTCACCCCAAACCCCCAGTGGAGAGCAGCAGTGGCAAGCTCTTACCTTGCACAGGTGACTACATGAACATGTCACCAGTGGGGGACTCCAACACCAGCAGCCCCTCTGACTGCTACTATGGCCCTGAGGACCCCCAGCACAAGCCAGTCCTCTCGTACTACTCATTGCCAAGATCCTTTAAGCACACCCAGCGCCCTGGGGAGCCGGAGGAGGGTGCCCGGCATCAGCACCTCCGCCTTTCTGCTAGCTCTGGTCGCCTTCTCTATGCTGCAACAGCGGATGATTCTTCCTCCTCCACCAGCAGCGACAGCCTGGGTGGGGGATACTGTGGGGCTAGGCTGGAGCCCAGCCTTCCACATCCCCACCATCAGGTTCTGCAGCCCCATCTGCCTCGAAAGGTGGACACAGCTGCTCAGACCAATAGCCGCCTGGCCCGGCCTACGAGGCTGTCCCTGGGGGATCCCAAGGCCAGCACCTTACCTCGGGCCCGAGAGCAGCAGCAGCAGCAGCAGCAGCAGCAGCAGCAGCAGCAACAGCAGCAACAGCAGCCCCTGCTGCACCCTCCGGAGCCCAAGAGCCCAGGGGAATATGTGAATATTGAATTTGGGAGTGATCAGCCTGGCTACTTGTCTGGCCCGGTGGCTTCCCGTAGCTCACCTTCTGTCAGGTGTCCATCCCAGCTCCAGCCAGCTCCCAGAGAGGAAGAGACTGGCACTGAGGAGTACATGAAGATGGACCTGGGGCCGGGCCGGAGGGCAGCCTGGCAGGAGAGCACTGGGGTCGAGATGGGCAGACTGGGCCCCGCACCTCCCGGGGCTGCTAGCATTTGCAGGCCTACCCGGGCAGTGCCCAGCAGCCGGGGTGACTATATGACCATGCAGATGAGTTGTCCCCGCCAGAGCTATGTGGACACCTCGCCAATTGCCCCTGTAAGCTATGCTGACATGCGGACAGGCATTGCTGCAGAGGAGGTGAGCCTGCCCAGGGCCACCATGGCTGCTGCCTCCTCATCCTCAGCAGCCTCTGCTTCCCCCACTGGGCCTCAAGGGGCAGCAGAGCTGGCTGCCCACTCATCCCTGCTGGGGGGCCCACAAGGACCTGGGGGCATGAGCGCCTTCACCCGGGTGAACCTCAGTCCTAACCGCAACCAGAGTGCCAAAGTGATCCGTGCAGACCCACAAGGGTGCCGGAGGAGGCATAGCTCCGAGACCTTCTCCTCAACACCCAGTGCCACCCGGGTGGGCAACACAGTGCCCTTTGGAGCCGGGGCAGCAATAGGGGGTGGTGGCGGTAGCAGCAGCAGCAGCGAGGATGTGAAACGTCACAGCTCTGCTTCCTTTGAGAATGTGTGGCTGAGGCCTGGGGAACTTGGGGGAGCCCCCAAGGAGCCAGCCCAACTGTGTGGGGCTGCTGGGGGTTTGGAGAATGGTCTTAACTACATAGATCTGGATTTGGTCAAGGACTTCAAACAGCGCCCTCAGGAGTGCAGCCCTCAACCGCAGCCTCCCCCACCCCCACCCCCTCATCAACCCCTGGGTAGCAGTGAGAGCAGCTCCACCCGCCGCCGCTCAAGTGAGGATTTAAGCGCCTATGCCAGCATCAGTTTCCAGAAGCAGCCAGAGGACCTTCAGTAG

>Rhi_ro1 [Snub-nosed monkey (Rhinopithecus roxellana) Irs1]

ATGGCGAGCCCTCCGGAGAGCGATGGCTTCTCGGACGTGCGCAAGGTGGGCTACCTGCGCAAACCCAAGAGCATGCACAAACGCTTCTTCGTACTGCGCGCGGCCAGCGAGGCTGGGGGCCCGGCGCGCCTCGAGTACTACGAGAACGAGAAGAAGTGGCGGCACAAGTCGAGCGCCCCCAAACGCTCTATCCCCCTTGAGAGCTGCTTCAACATCAACAAGCGGGCTGACTCCAAGAACAAGCACCTGGTGGCTCTCTACACCCGGGACGAGCACTTTGCCATTGCGGCGGACAGCGAGGCGGAGCAAGACAGCTGGTACCAGGCTCTCCTACAGCTACACAACCGTGCCAAGGGTCACCACGACGGAGCTGCGGCCCTCGGGGCGGGAGGTGGCGGGGGCAGCTGCAGCGGCAGCTCCGGCCTTGGTGAGGCCGGGGAGGACTTGAGCTACGGTGACGTGCCCCCAGGACCCGCGTTCAAAGAGGTCTGGCAGGTGATCCTGAAGCCCAAGGGCCTGGGTCAGACAAAGAACCTGATTGGTATCTACCGCCTCTGCCTGACCAGCAAGACCATCAGCTTCGTGAAGCTGAACTCGGAGGCAGCCGCCGTGGTGCTGCAGCTGATGAACATCAGGCGCTGCGGCCACTCGGAGAACTTCTTCTTCATCGAGGTGGGCCGTTCTGCCGTGACGGGGCCCGGGGAGTTCTGGATGCAGGTGGATGACTCCGTAGTGGCCCAGAACATGCACGAGACCATCCTGGAGGCCATGCGGGCTATGAGCGATGAGTTCCGCCCTCGCAGCAAGAGCCAGTCCTCGTCCAACTGCTCCAACCCCATCAGCGTCCCCCTGCGCCGGCACCATCTCAACAACCCCCCGCCCAGCCAGGTGGGGCTGACCCGCCGATCTCGCACTGAGAGCATCACCGCCACCTCCCCGGCCAGCATGGTGGGCGGGAAGCCAGGCTCCTTCCGCGTCCGCGCCTCCAGTGACGGCGAAGGCACCATGTCCCGCCCAGCCTCGGTGGATGGCAGCCCTGTGAGTCCGAACACCAACAGAACCCACGCCCACCGGCATCGGGGCAGCGCCCGGCTGCACCCCCCGCTCAACCACAGCCGCTCCATCCCCATGCCAGCTTCCCGCTGCTCGCCTTCGGCCACCAGCCCCGTCAGTCTGTCGTCCAGCAGCACCAGTGGCCATGGCTCCACCTCGGATTGTCTCTTCCCACGGCGATCTAGTGCTTCGGTGTCTGGTTCCCCCAGCGATGGCGGTTTCATCTCCTCAGATGAGTATGGCTCCAGTCCCTGCGATTTCCGGAGTTCCTTCCGCAGTGTAACTCCAGATTCCCTGGGCCACACCCCACCAGCCCGCGGTGAGGAGGAGCTAAGCAACTATATCTGCATGGGCGGCAAGGGGCCCTCCACCCTGACCGCCCCCAACGGTCACTACATTTTGTCTCGGGGTGGCAATGGCCACCGCCACACCCCAGGAACAGGCTTGGGCACGAGTCCAGCCTTGGCTGGGGATGAAGCATCCAGTGCTGCGGATCTGGATAATCGCTTCCGAAAGAGAACTCACTCTGCAGGCACATCCCCTACCATTACCCACCAGAAGACCCCGTCCCAGTCCTCAGTGGCTTCCATTGAGGAGTATACAGAGATGATGCCTGCCTACCCACCAGGAGGTGGCAGTGGAGGCCGACTGCCGGGACACAGGCACTCTGCCTTCGTGCCCACCCACTCCTACCCAGAGGAGGGTCTGGAAATGCACCCCTTGGAGCGTCGGGGGGGCCACCACCGCCCAGACAGCTCTACCCTCCACACTGATGATGGCTACATGCCCATGTCCCCAGGGGTGGCCCCAGTGCCCAGCAGCCGAAAGGGCAGTGGAGACTATATGCCCATGAGCCCCAAGAGCGTGTCTGCCCCACAGCAGATCATCAATCCCATCAGACGCCATCCCCAGAGAGTGGACCCCAATGGCTACATGATGATGTCCCCCAGCGGCGGCTGCTCTCCTGACATTGGAGGTGGCCCCAGCAGCAGCAGCAGTGCCATCCCTTCCGGGAGCAGCTATGGAAAGCTGTGGACAAACGGGGTAGGGGGCCACCACTCTCATGTCTTGCCTCACCCCAAACCCCCAGTGGAGAGCAGCGGTGGCAAGCTCTTACCTTGCACAGGTGACTACATGAACATGTCACCAGTGGGGGACTCCAACACCAGCAGCCCCTCCGACTGCTACTGTGGCCCTGAGGACCCCCAGCACAAGCCAGTCCTCTCGTACTACTCATTGCCAAGATCCTTTAAGCACACCCAGCGCCCCGGGGAGCCAGAGGAGGGTGCCCGGCATCAGCACCTCCGCCTTTCCACTAGCTCTGGTCGCCTTCTCTATGCTGCAGCAGCGGATGATTCTTCCTCCTCCACCAGCAGCGACAGCCTGGGTGGGGGATACTGTGGGGCTAGGCTGGAGCCCAGCCTTCCACATCCCCACCATCAGGTTCTGCAGCCCCATCTGCCTCGAAAGGTGGACACGTCTGCTCAGACCAATAGCCGCCTGGCCCGGCCTACAAGGCTGTCCCTGGGGGATCCCAAGGCCAGCACCTTACCTCGGGCCCGAGAGCAGCAGCAGCAGCAGCAGCAGCAGCAACAGCAGCCCCTGCTGCACCCTCCGGAGCCCAAGAGCCCAGGGGAATATGTGAATATTGAATTCGGGAGTGATCAGCCTGGCTACTTGTCTGGCCCGGTGGCTTCCCGTAGCTCACCTTCTGTCAGGTGCCCATCCCAGCTCCAGCCAGCTCCCAGAGAGGAAGAGACTGGCACTGAGGAGTACATGAAGATGGACCTGGGGCCGGGCCGGAGGGCAGCCTGGCAGGAGAGCACTGGGGTCGAGATGGGCAGACTGGGCCCCGCACCTCCCGGGGCTGCTAGCATTTGCAGGCCTACCCGGGCAGTGCCCAGCAGCCGGGGTGACTATATGACCATGCAGATGAGTTGTCCCCGCCAGAGCTATGTGGACACCTCGCCAATTGCCCCTGTAAGCTATGCTGACATGCGGACAGGCATTGCTGCAGAGGAGGTGAGCCTGCCCAGGGCCACCATGGCTGCTGCCTCCTCATCCTCAGCAGCCTCTGCTTCCCCCACTGGGCCTCAAGGGGCAGCAGAGCTGGCTGCCCACTCATCCCTGCTGGGGGGCCCACAAGGACCTGGGGGCATGAGCGCCTTTACCCGGGTGAACCTCAGTCCTAACCGCAACCAGAGTGCCAAAGTGATCCGTGCAGACCCACAAGGGTGCCGGAGGAGGCATAGCTCCGAGACCTTCTCCTCAACACCCAGTGCCACCCGGGTGGGCAACACAGTGCCCTTTGGAGCCGGGACAGCAATAGGGGGCGGTGGCGGCAGCAGCAGCAGCGAGGATGTGAAACGCCACAGCTCTGCTTCCTTTGAGAATGTGTGGCTGAGGCCTGGGGAACTTGGGGGAGCCCCCAAGGAGCCAGCCCAACTGTGTGGGGCTGCTGGGGGTTTGGAGAATGGTCTTAACTACATAGATCTGGATTTGGTCAAGGATTTCAAACAGCGCCCTCAGGAGTGCAGCCCTCAACCGCAGCCTCCCCCACCCCCACCTCCTCATCAACCCCTGGGTAGCAGTGAGAGCAGCTCCACCCGCCGCCGCTCAAGTGAGGATTTAAGCGCCTATGCCAGCATCAGTTTCCAGAAGCAGCCAGAGGACCTTCAGTAG

>Aot_na1 [Ma’s night monkey (Aotus nancymaae) Irs1]

ATGGCGAGCCCTCCGGAGAGCGAGGGCTTCTCGGACGTGCGCAAGGTGGGCTACCTGCGCAAACCCAAGAGCATGCACAAACGCTTCTTCGTACTGCGCGCGGCCAGCGAGGCTGGGGGCCCGGCGCGCCTCGAGTACTACGAGAACGAGAAGAAGTGGCGGCACAAGTCGAGCGCCCCCAAACGCTCGATCCCCCTTGAGAGCTGCTTCAACATCAACAAGCGTGCTGACTCCAAGAACAAGCACCTGGTGGCTCTCTACACCCGGGACGAGCACTTTGCCATCGCGGCGGACAGCGAGGCCGAACAAGACAGCTGGTACCAGGCTCTCCTGCAGCTGCACAACCGCGCCAAAGGCCACCACGATGGAGCTGCGGCCCTCGGGGCGGGAGGCTGCGGGAGCAGCTGCAGCGGCAGCTCGGGAGTTGGTGAGGCTGGGGAGGACCTGAGCTACGTGGACGGGCCCCCAGGACCCACGTTCAAAGAGGTCTGGCAGGTGATCCTGAAGCCCAAGGGCCTGGGTCAGACAAAGAACCTGATTGGTATCTACCGCCTCTGCCTGACAAGCAAGACCATCAGTTTCGTGAAGCTGAACTCGGAGGTAGCCGCTGTGGTGTTGCAGCTGATGAACATCAGGCGCTGTGGCCACTCGGAGAACTTCTTCTTTATCGAGGTGGGTCGTTCTGCCGTGACGGGGCCCGGGGAGTTCTGGATGCAGGTGGATGACTCCGTGGTGGCCCAGAACATGCACGAGACCATCCTGGAGGCCATGCGGGCCATGAGCGATGAATTCCGTCCTCGCAGCAAGAGCCAGTCCTCGTCCAACTGCTCCAACCCCATCAGCGTCCCCCTGCGCCGGCACCATCTCAACAACCTCCCGCCCAGCCAGGTGGGGCTGACCCGCCGATCACGCACCGAGAGCATCACCGCCACCTCCCCGGCCAGCATGGTGGGCGGGAAGCCAGGCTCCTTCCGTGTCCGCGCCTCCAGCGACGGTGAAGGCACCATGTCCCGCCCGGCCTCTGTGGACGGCAGCCCTGTGAGTCCCAGCACCAACAGAACCCACGCCCACCGGCATCGGGGCAGCGCGCGGCTGCACCCCCCGCTCAACCACAGCCGCTCCATCCCCATGCCAGCTTCCCGCTGCTCGCCTTCGGCCACCAGCCCGGTCAGTCTGTCATCCAGCAGCACCAGTGGCCATGGCTCCACCTCAGATTGTCTCTTCCCACGGCGGTCTAGTGCTTCGGTGTCTGGTTCCCCCAGCGATGGCGGTTTCGTCTCCTCGGATGAGTATGGCTCCAGTCCCTGCGATTTCCGGAGTTCCTTCCGCAGTGTCACTCCGGATTCCGTGGGTCACACCCCACCGGCCCGCGGTGAGGAGGAGCTAAGCAACTATATCTGCATGGGCGGCAAGGGGCCCTCCACCCTGACCGCCCCCAACGGTCACTACATTTTGTCTCGGGGTGGCAATGGCCACCGCTACACCCCAGGAACAGGCTTGGGCACGAGTCCAGCCTTGGCTGGGGATGAAGCAGCCAGTGCTGCCGATCTGGATAATCGGTTCCGAAAGAGAACTCACTCGGCAGGCACATCCCCTTCCATTTCCCACCAGAAGACCCCGTCTCAGTCCTCAGTGGCTTCCATTGAGGAGTATACAGAGATGATGCCTGTCTACCCACCAGGAGGTGGCAGTGGAGGCCGACTGCCGGGACACAGGCACTCCGCCTTTGTGCCCACCCATTCCTACCCTGAGGAGGGTCTGGAAATGCACCCCTTGGAGCGTCGGGGGGGCCACCATCGCCCAGAAAGCTCCACCCTCCACAGTGATGATGGCTACATGCCCATGTCCCCAGGGGTGGCCCCAGTGCCCAGCAGCCGAAAGGGCAGTGGAGACTATATGCCCATGAGCCCCAAGAGCGTGTCTGCCCCACAGCAGATCATCAACCCCATCAGACGCCATCCCCAGAGAGTGGACCCCAATGGCTACATGATGATGTCCCCCAGCAGCAGCTACTCCCCTGACATTGGAGGTGGCCCCGGCAGCAGCAGCAGCAGCAGCAGCAGGGCTGTCCCTTCCGGGAGCAGCTATGGAAAGCTGTGGACAAACGGGGTAGGGGGCCACCACGCTCATGTCTTGCCTCACCCCAAACCCCCAGTGGAGAGCAGCAGTGGCAAGCTCTTGCCTTGCACAGGTGACTACATGAACATGTCGCCAGTGGGGGACTCCAACACCAGCAGCCCCTCCGACTGCTACTACGGCCCTGAGGACCCCCAGCACAAGCCAGTCCTCTCCTACTACTCATTGCCAAGATCCTTTAAGCACACCCAGCTCCCCGGGGAGCTGGAGGAGGGTGCCCGGCATCAGCACCTTCGCCTTTCCGCTAGCTCTGGTCGCCTTCTCTATGCTGCAACAGTGGAAGATTCTTCCTCCTCCACCAGCAGCGATAGCCTGGGTGGGGGATACTGTGGGACTAGGATGGAGCCCAGCCTTCCACATTCCCACCATCAGGTTCTGCAGCCCCATCTGCCTCGAAAGGTGGACTCAGCTGCTCAGACCAATAGCCGCCTGGCCCGGCCCACGAGGCTGTCCCTGGGGGATCCCAAGGCCAGCACCTTACCTCGGGCCGGAGAGCAGCAGCAGCAACAGCAGCAGCAGCCCCTGCTGCACCCTCCGGAACCCAAGAGCCCAGGGGAATATGTGAATATTGAATTTGGGAGTGATCAGCCTGGCTACTTGTCTGGCCCTGTGGCTTCCCACAGCTCACCTTCTGTCAGGTGTCCATCCCAACTCCAGCCAGCTCCCAGAGAGGAAGAGACTGGCACTGAGGAGTACATGAAGATGGACTTGAGGCCAGGCCGGAGGGCAGCCTGGCAGGAGAGCACTGGGGTTGAGGTGGGCAGAGTGGGCCCCGCACCTCCCGGGGCTGCTAGCATTTGCAGGCCTACCCGGGCAGTGCCCAGCAGCCGGGGTGACTACATGACCATGCAGATGGGTTGTCCCCGTCAGAGCTACGTGGACACCTCACCAGTTGCCCCTGTGAGCTATGCTGACATGCGGACAGGCATTGCTGCAGAGGAAGTTAGCCTGCCCAGGGCCACCATGGCTGCTGCCTCAGCCTCAGCAGCCTCTGCTTCCCCCGCTGGGCCTCAAGGGGCAGCAGAGTTGGCTGCCCGCTCATCCCTGCTGGGGGAACTGCAAGGATCTGGGGGCATGAGCGCCTTCACCCGGGTGAACCTCAGTCCTAACCGCAACCAGAGTGCCAAAGTGATCCGTGCAGACGCACAAGGGTGCCGGAGGAGGCATAGCTCCGAGACCTTCTCCTCAACACCCAGTGCCACCCGGGTGGGCAACACGGTGCCCTTTGGAGCAGGGGCAGCAGGAGGGGGCAGTGGCAGTAGCAGCAGCAGCAAGGATGTGAAACGCCACAGCTCTGCTTCCTTTGAGAACGTGTGGCTGAGGCCTGGGGAGCTTGGGGGAGCCCCCAAGGAGCCAGCCCAACTGTGCGGGGCTGCTGGGGATTTGGAGAATGGTCTTAACTACATAGACCTGGATTTGGTCAAGGACTTCAAACAATGCCCTCAGGAGCGCACCCCTCAACCACAGCCTCCCCCACCCCCATCCCCACCCCCTCATCAACCCCTGGGCAGCAGTGAGAACAGTTCCACCCCCCGCTCCAGTGAGGATTTAAGCGCCTATGCCAGCATCAGTTTCCAGAAGCAGCCAGAGGACCGTCAGTAG

>Cal_ja1 [Marmoset (Callithrix jacchus) Irs1]

ATGGCAAGCCCTCTGGAGAGCGAGGGCTTCTCGGACGTGCGCAAGGTGGGCTACCTGCGCAAACCCAAGAGCATGCACAAACGCTTCTTCGTACTGCGCGCGGCCAGCGAGGCTGGGGGCCCGGCGCGCCTCGAGTACTACGAGAACGAGAAGAAGTGGCGGCACAAGTCGAGCGCCCCCAAACGCTCAATCCCCCTTGAGAGCTGCTTCAACATCAACAAGCGTGCTGACTCCAAGAACAAGCACCTGGTGGCTCTCTACACCCGGGACGAGCACTTTGCCATCGCGGCGGACAGCGAGGCCGAACAAGACAGCTGGTACCAGGCTCTCCTGCAGCTGCACAACCGCGCCAAGGGCCACCACGATGGAGCTGCGGTCCTCGGGGCGGGAGGCGGCGGGAGCAGCTGCAGCGGCAGCTCGGGAGTTGGTGAGGCTGGGGAGGACCTCAGCTACGGGGACGGGCCCCCAGGACCCACATTCAAAGAAGTCTGGCAGGTGATCCTGAAGCCCAAGGGCCTGGGTCAGACAAAGAACCTGATTGGTATATACCGCCTCTGCCTGACAAGCAAGACCATCAGCTTCGTGAAGCTGAACTCGGAGGTAGCGGCTGTGGTGCTGCAGCTGATGAACATCAGGCGCTGCGGCCACTCGGAGAACTTCTTCTTCATCGAGGTGGGTCGTTCTGCCGTGACGGGGCCTGGGGAGTTCTGGATGCAGGTGGATGACTCCGTGGTGGCCCAGAACATGCACGAGACCATCCTGGAGGCCATGCGGGCCATGAGCGATGAGTTCCGCCCTCGCAGCAAGAGCCAGTCCTCGTCCAACTGCTCCAACCCCATCAGCGTCCCCCTGCGCCGGCACCATCTCAACAACCTCCCGCCCAGCCAGGTGGGGCTGACACGCCGATCACGCACCGAGAGCATCACGGCCACCTCCCCAGCCAGCATGGTGGGCGGGAAACCAGGCTCCTTCCGGGTCCGCGCCTCCAGCGACGGTGAAGGCACCATGTCTCGCCCGGCCTCGGTGGACGGTAGTCCTGTGAGTCCCAGCACCAACAGAACCCACGCCCACCGGCACCGGGGCAGCGCACGGCTGCACCCCCCGCTCAACCACAGCCGCTCCATCCCCATGCCAGCTTCCCGCTGCTCGCCTTCGGCCACCAGCCCGGTCAGTCTGTCATCCAGCAGCACCAGTGGCCATGGCTCCACCTCAGATTGTCTCTTCCCACGGCGGTCTAGTGCTTCGGTGTCTGGTTCCCCCAGCGATGGCGGTTTTGTCTCCTCTGATGAGTATGGCTCCAGTCCCTGCGATTTCCGGAGTTCCTTTCGCAGTGTCACTCCGGATTCCGTGGGTCACACCCCACCGGCCCGCGGTGAGGAGGAGCTAAGCAACTATATCTGCATGGGCGGCAAGGGGCCCTCAACCCTGACTGCTCCCAACGGTCACTACATTTTGTCACGGGGTGGCAATGGCCACCGCTACACCCCAGGAACAGGCTTGGGCACTAGTCCAGCCTTGACTGGGGATGAAGCAGCCAGTGCTGCCGATCTGGATAATCGATTCCGAAAGAGAACTCACTCGGCAGGCACATCCCCTTCCATTTCCCACCAGAAGACACCGTCTCAGTCCTCAGTGGCTTCCATTGAGGAGTATACAGAGATGATGCCTGTCTACCCACCAGGAGGTGGCAGTGGAGGCCGACTGCCAGGACACAGGCACTCCGCCTTCATGCCCACCCATTCCTACCCTGAGGAGGGTTTGGAAATGCACCCCTTGGAGCGTCGGGGGGGCCACCATCGCCCAGAAAGCTCCACCCTCCACAGTGATGATGGCTACATGCCCATGTCCCCAGGGGTGGCCCCAGTGCCCAGCAGCCGAAAGGGCAGTGGAGACTATATGCCCATGAGCCCCAAGAGCGTATCTGCCCCACAGCAGATCATCAACCCCATCAGACGCCATCCCCAGAGAGTGGACCCCAATGGTTACATGATGATGTCCCCCAGCAGCAGCTACTCCCCTGACATTGGAGGTGGCCCTGGCAGCAGCAGCAGCAGCAGCAGCAGCAGGGCTGTCCCTTCCGGGAGCAGCTATGGAAAGCTGTGGACAAACGGGGTAGGGGGCCACCATGCTCATGTCTTGCCTCACCCCAAACCCCCAGTGGAGAGCAGCAGTGGCAAGCTCTTGCCTTGCACAGGTGACTACATGAACATGTCGCCAGTGGGGGACTCCAACACCAGCAGCCCCTCCGACTGCTACTACGGTCCTGAGGACCCCCAGCACAAGCCAGTCCTCTCCTACTACTCATTGCCAAGATCCTTTAAGCACACCCAGCTCCCCGGGGAGTTGGAGGAGGGTGCCCGGCATCAGCACCTTCGCCTTTCCGCTAGCTCTGGTCGCCTTCTCTATGCTGCAACAGTGGAAGATTCTTCCTCCTCCACCAGCAGCGATAGCCTGGGTGGGGGATACTGTGGGGCTAGGATGGAGCCCAGCCTTTCACATTCCCACCATCAGGTTCTGCAGCTCCATCTGCCTCGAAAGGTGGACTCAGCTGCTCAGACCAATAGCCGCCTGGCCCGGCCCACGAGGCTGTCCCTGGGGGATCCCAAGGCCAGCACCTTACCTCGGGCTGGAGAGCAACAGCAGCAGCAGCAGCAGCAGCAGCAGCAACAGCAACAGCAGCAGCCCCTGCTGCACCCTCCAGAGCCCAAGAGCCCAGGGGAATATGTGAATATTGAATTTGGGAGTGATCAGCCTGGCTACTTGTCTGGCCCTGTGGCTTCCCACAGCTCACCTTCTGTCAGGTGTCCATCCCAGCTCCAGCCAGCTCCCAGAGAGGAAGAGACTGGCACTGAGGAGTACATGAAAATGGACTTGAGGCCAGGCCGGAGGGCAGCCTGGCAGGAGAGCACTGGGGTTGAGGTGGGCAGAGTGGGCCCCGCACCTCCCGGGGTTGCTAGCATTTGCAGGCCTACCCGTGCAGTGCCCAGCAGTAGGGGTGACTACATGACCATGCAGATGGGTTGTCCCCATCAGAGCTACGTGGACACCTCACCAGTTGCCCCTGTGAGCTATGCTGACATGCGGACAGGCATTGCTGCAGAGGAGGTTAGCCTGCCCAGGGCCACCATGGCTGCTGCCTCCTCAGCCTCGGCAGCCTCTGCTTCCCCCGCTGGGCCTCAAGGGGTAGCAGAGCTGGCTGCCCGCTCATCCCTGCTGGGGGAACCGCAAGGACCTGGGGGCATGAGCGCCTTCACCCGGGTGAACCTCAGTCCTAATCGAAACCAGAGTGCCAAAGTGATCCGTGCAGACGCACAAGGGTGCCGAAGGAGGCACAGCTCCGAGACCTTCTCCTCAACACCCAGTGCCACCCGGCTGGGCAACACGGTGCCCTTTGGAGCAGGGGCAGCAGGAGGGGGCAGTGGCGGTAGCAGCAGCAGCAAGGATGTGAAACGCCACAGCTCTGCTTCCTTTGAGAATGTGTGGCTGAGGCCTGGGGAGCTTGGGGGAGCCCCTAAGGAGCCAGCCCAACTGTGTGGGGCTGCTGGGGATTTGGAGAATGGTCTTAACTACATAGACCTGGATTTGGTCAAGGACTTCAAACAATGCCCTCAGGAGCGCACCCCTCAACCACAGCCACCCCCACCCCCATCCCCACCCCCTCATGAACCCCTGGGCAGCGGTGAGAACAGCTCCACCCCCCGCTCCAGTGAGGATTTAAGCGCGTATGCCAGTATCAGTTTCCAGAAGCAGCCAGAGGACCATCAGTAG

>Sai_bo1 [Squirrel monkey (Saimiri boliviensis boliviensis) Irs1]

ATGGCGAGCCCTCCGGAGAGCGAGGGCTTCTCGGACGTGCGCAAGGTGGGCTACCTGCGCAAACCCAAGAGCATGCACAAACGCTTCTTCGTACTGCGCGCGGCCAGCGAGGCTGGGGGCCCGGCGCGCCTCGAGTACTACGAGAACGAGAAGAAGTGGCGGCACAAGTCGAGCGCCCCCAAACGCTCGATCCCCCTTGAGAGCTGCTTCAACATCAACAAGCGTGCTGACTCCAAGAACAAGCACCTGGTGGCTCTCTACACCCGGGACGAGCACTTTGCCATCGCGGCGGACAACGAGGCCGAACAAGACAGCTGGTACCAGGCTCTCCTGCAGCTGCACAACCGCGCCAAGGGCCACCACGATGGAACTGCGGCCCTCGGGGCGGGAGGCGGCGGGAGCAGCTGCAGCGGCAGCTCGGGAGTTGGTGAGGCTGGGGAGGACCTGAGCTACGGGGACGGGCCCCCAGGACCCACATTCAAAGAGGTCTGGCAGGTGATCCTGAAGCCCAAGGGCCTGGGTCAGACAAAGAACCTGATTGGTATCTACCGCCTCTGCCTGACAAGCAAGACCATCAGCTTCGTGAAGCTGAACTCGGAGGTAGCGGCTGTGGTGCTGCAGCTGATGAACATCAGGCGCTGCGGCCACTCGGAGAACTTCTTCTTTATCGAGGTGGGTCGTTCTGCCGTGACGGGGCCCGGGGAATTCTGGATGCAGGTGGATGACTCCGTGGTGGCCCAGAACATGCACGAAACCATCCTGGAGGCCATGCGGGCCATGAGCGATGAGTTCCGTCCTCGCAGCAAGAGCCAGTCCTCGTCCAATTGCTCTAACCCCATCAGCGTCCCCCTGCGCCGGCACCATCTCAACAACCTCCCGCCCAGCCAGGTAGGGCTGACCCGCCGATCACGCACCGAGAGCATCACCGCCACCTCCCCGGCCAGCATGGTGGGTGGGAAGCCAGGCTCCTTCCGTGTCCGCGCCTCCAGCGACGGTGAAGGCACCATGTCCCGCCCGGCCTCGGTGGACGGCAGCCCTGTGAGTCCCAGCACCAACAGAACCCACGCCCACCGGCATCGGGGCAGCGCGCGGCTGCACCCCCCGCTCAACCACAGCCGCTCCATCCCCATGCCAGCTTCCCGCTGCTCGCCTTCGGCCACCAGCCCGGTCAGTCTGTCATCCAGCAGCACCAGTGGCCATGGCTCCACCTCAGATTGTCTCTTCCCACGGCGGTCTAGTGCTTCGGTGTCTGGTTCCCCCAGCGATGGCGGTTTCGTCTCCTCGGATGAGTATGGCTCCAGTCCCTGCGATTTCCGGAGTTCCTTCCGCAGTGTCACTCCGGATTCCGTGGGTCACACCCCACCGGCCCGCGGTGAGGAGGAGCTGAGCAACTATATCTGCATGGGCGGCAAGGGGCCCTCCACCCTGACCGCCCCCAACGGTCACTATATTTTGTCTCGGGGTGGCAATGGCCACCGCTACACCCCAGGAACAGGCTTGGGCACGAGTCCAGCCTTGGCTGGGGATGAAGCAGCCAGTGCTGCCGATCTGGATAATCGGTTCCGAAAGAGAACTCACTCGGCAGGCACATCCCCTTCCATTTCCCACCAGAAGACCCCGTCTCAGTCCTCAGTGGCTTCCATTGAGGAGTATACAGAGATGATGCCTGTCTACCCACCAGGAGGTGGCAGTGGAGGCCGACTGCCGGGACACAGGCACTCCGCCTTCGTGCCCACCCATTCCTACCCTGAGGAGGGTCTGGAAATGCACCTCTTGGAGCGTCGGGGGGGCCACCATCGCCCAGAAAGCTCCACCCTCCACAGTGATGATGGCTACATGCCCATGTCCCCAGGGGTGGCCCCAGTGCCCAGCAGCCGAAAGGGCAGTGGAGACTATATGCCCATGAGCCCCAAGAGCGTGTCTGCCCCACAGCAGATCATCAACCCCATCAGACGCCATCCCCAGAGAGTGGACCCCAATGGCTACATGATGATGTCCCCCAGCAGCAGCTACTCCCCTGACATTGGAGGTGGCCCTGGCAGCAGCAGCAGCAGGGCTGTCCCTTCTGGGAGCAGCTATGGAAAGCTGTGGACAAACGGGGTAGGGGGCCACCACGCTCATGTCTTGCCTCACCCCAAACCCCCTGTGGAAAGCAGCAGTGGCAAGCTCTTGCCTTGCACAGGTGACTACATGAACATGTCGCCAGTGGGGGACTCCAACACCAGCAGCCCCTCTGACTGCTACTACGGCCCTGAGGACCCCCAGCACAAGCCAGTCCTCTCCTACTACTCATTGCCAAGATCCTTTAAGCACACCCAGCTCCCTGGGGAGCTGGAGGAGGGTGCCCGACATCAGCACCTTCGCCTTTCCGCTAGCTCTGGTCGCCTTCTCTATGCTGCAACAGTGGAAGATTCTTCCTCCTCCACCAGCAGCGATAGCCTGGGTGGGGGATATTGTGGGGCTAGGATGGAGCCCAGCCTTCCACATTCCCACCATCAGGTTCTGCAGCCTCATCTGCCTCGAAAGGTGGACTCAGCTGCTCAGACCAATAGCCGCCTGGCCCGGCCCACGAGGCTGTCCCTGGGGGATCCCAAGGCCAGCACCTTACCTCGGGCTGGAGAGCAGCAGCAGCAGCAGCAGCAGCAGCAGCCCCTGCTGCACCCTCCGGAGCCCAAGAGCCCAGGGGAATATGTGAATATTGAATTTGGGAGTGATCAGCCTGGCTACTTGTCTGGCCCTGTGGCCTCCCACAGCTCACCTTCTGTCAGGTGTCCATCCCAACTCCAGCCAGCTCCCAGAGAGGAAGAGACTGGCACTGAGGAGTACATGAAGATGGACTTGAGGCCAGGCCGGAGGGCAGCCTGGCAGGAGAGCACTGGGGTTGAGGTGGGCAGAGTGGGCCCTGCACCTTCTGGGGCTGCTAGCATTTGCAGGCCTACCCGAGCAGTGCCCAGCAGCCGGGGTGACTACATGACCATGCAGATGGGTTGTCCCCGTCAGAGCTACGTGGACACCTCACCAGTTGCCCCTGTGAGCTATGCTGACATGCGGACAGGCATTGCTGCAGAGGAGGTTAGCCTGCCCAGGGCCACCATGGCTGCTGCCTCCTCAGCCTCAGCAGCCTCTGCCTCCCCTGCTGGGCCTCAAGGGGCAGCAGAGCTGGCTGCCCAATCATCCCTGCTGGGGGAACCGCAAGGACCTGGGGGCATGAGTGCCTTTACCCCGGTGAACCTCAGTCCTAACCGCAACCAGAGTGCCAAAGTGATCCGTGCAGACGCACAAGGGTGCCGGAGGAGGCATAGCTCCGAGACCTTCTCCTCAACACCCAGTGCCACCCGGGTGGGCAACACGGTGCCCTTTGGAGCAGGGGCAGCAGGAGGGGGCAGTGGCGGTAGCAGCAGCAGCAAGGATGTAAAACGCCACAGCTCTGCTTCCTTTGAGAACGTGTGGCTGAGGCCTGGGGAGCTTGGGGGAGCCCCCAAGGAGCCAGCCCAACTGTGCGGGGCTGCCGGGGATTTGGAGAATGGTCTTAATTACATAGACCTGGATTTGGTCAAGGACATCAAACAATGCCCTCAGGAGCGAACCCCTCAACCACAGCCTCCCCCACCCCCATCCCCACCCTCTCATCAACCCCTGGGCAGCGGTGAGAACAGCTCCACCCCCCGCTCCAGTGAGGATTTAAGCACCTATGCCAGCATCAGTTTCCAGAAGCAGCCAGAGGACCGTCAGTAG

>Tar_sy1 [Tarsier (Tarsius syrichta) Irs1]

ATGGCGAGCCCTCCGGAGAGCGACGGCTTCTCGGACGTGCGCAAGGTGGGCTACCTCCGCAAACCCAAGAGCATGCACAAGCGCTTTTTCGTGCTGCGGGCGGCCAGCGAGGCTGGGGGCCCGGCGCGCCTCGAGTACTACGAGAACGAGAAGAAGTGGCGGCACAAGTCGAGCGCCCCCAAACGCTCGATTCCCCTCGAGAGCTGCTTCAACATCAACAAGCGGGCTGACTCCAAGAACAAGCACCTGGTGGCTCTCTACACTCGGGACGAGCACTTTGCCATCGCTGCGGACAGCGAGGCGGAGCAAGACAGCTGGTACCAGGCTCTCCTGCAGCTGCACAACCGCGCCAAGGGCCACCACGACGGGGCCACGGCACCCGGGGTGGGAGGCGGCGGGGGCAGCTGCAGCGGCAGCTCCGGCCTCGGCGAGGCTGGGGAGGACTTGAGCTACGGGGATGTGCCTCCAGGACCGGCGTTCAAAGAGGTCTGGCAGGTGATCCTGAAGCCCAAGGGCCTGGGTCAGACAAAGAACCTGATCGGCATCTACCGCCTCTGCCTGACCAGCAAGACCATCAGCTTCGTGAAGCTGAACTCGGAGGCAGCGGCTGTGGTGCTGCAGCTGATGAACATCAGGCGCTGCGGCCACTCGGAGAACTTCTTCTTCATTGAGGTGGGCCGTTCTGCTGTGACGGGGCCTGGCGAGTTCTGGATGCAGGTGGATGACTCGGTGGTGGCCCAGAACATGCACGAGACCATCCTGGAGGCCATGCGGGCCATGAGCGACGAGTTCCGTCCTCGCAGCAAGAGCCAGTCGTCGTCCAACTGCTCCAACCCTATCAGCGTCCCCCTGCGCAGGCACCATCTCAACAACCCCCCCCCCACCCAGGGGGGGCTCACCCGCCGATCACGCACCGAGAGCATCACCGCCACCTCCCCAGCCAGCATGGTGGGTGGGAAGCCAGGCTCCTTCCGCGTCCGCGCCTCCAGCGACGGGGAAGGCACCATGTCCCGCCCGGCCTCAGTGGACGGCAGCCCTGTGAGTCCCAGCACCAATAGGACCCACGCCCACCGGCATCGCGGCAGTGCCCGGCTACACCCGCCACTCAACCATAGCCGCTCCATCCCCATGCCATCTTCTCGCTGCTCGCCTTCGGCCACCAGCCCGGTCAGTCTGTCGTCCAGCAGTACCAGTGGCCACGGCTCCACCTCGGACTGCCTCTTCCCACGGCGGTCTAGTGCTTCTGTGTCCGGTTCCCCCAGCGATGGCGGGTTCATCTCCTCGGATGAGTATGGCTCCAGTCCCTGCGATTTCCGAAGTTCCTTCCGCAGTGTCACTCCGGACTCCCTGGGCCACACCCCGCCGGCCCGCGGTGAAGAGGAGCTGAGCAACTATATCTGCATGGGTGGCAAGGGAGCCTCTACCCTGACCGCCCCCAACGGTCACTACATTTTGTCTCGCGGTGGCAATGGCCACCGCTACATCCTAGGAGCTGGCTTGGGCACAAGTCCAGCCTTGGCTGGGGATGAAGCAACCAATGCTGCAGATCTGGATAATCGGTTCCGAAAGAGGACTCACTCGGCAGGCACATCCCCTACCATTTCCCACCAGAAGACCCCATCCCAGTCCTCAGTGGCTTCCATTGAGGAGTATACAGAGATGATGCCTGCCTACCCACCAGGAGGTGGCAGTGGAGGCCGACTGCCGGGCCACCGGCACTCTGCCTTCGTGCCCACCCACTCCTACCCCGAGGAGGGTCTGGAAATGCACCCCTTGGAGCGTCGGGGGGGCCACCATCGTCCGGACAGCTCCACCCTCCACACTGATGATGGCTACATGCCCATGTCCCCAGGGGTGGCCCCAGTGCCGGGCAGCCGAAAGGGCAGTGGGGACTATATGCCCATGAGCCCCAAGAGCGTGTCTGCCCCACAGCAGATCATCAACCCCATCAGACGCCATCCCCAGAGAGTGGACCCCAATGGCTACATGATGATGTCCCCCAGTGGCAGTTGCTCCCCTGACACTGGAGGTGGACCCAGCAGCAGCAGCAGCAGTGCTGCTCCTTCGGGGAGCGGCTATGGGAAGCTGTGGACAAATGGGGTAGGGGTCCACCACTCTCATGCCCTGCCTCATCCCAAACCCCCAGTGGAGAGCAGTGGTGGCAAGCTCTTGCCTTGCGCAGGTGACTACATGAACATGTCACCAGTGGGGGACTCGAACACCAGCAGCCCCTCTGACTGCTACTATGGGCCTGAGGACCCCCAGCACAAGCCAGTCCTCTCCTACTACTCATTGCCAAGGTCCTTCAAGCACACCCAGCGCCCTGGGGAGCTGGAGGAGGGTGCCCGGCACCAGCACCTCCGCCTTTCCGCTAGCTCTGGTCGCCTTCTCTATGCTACAACAGCAGAAGATTCTTCCTCATCCACCAGCAGTGACAGCCTGGGTGGGGGATACTGTGGTGCTAGGCCGGAGCCTGGCCTCCCACATCCCCACCATCAGATCCTGCAGTCCCATCTGCCTCGAAAGGTGGACACGGCTGCCCAGACCAACAGCCGCCTGGTTCGGCCCACAAGGCTGTCCCTGGGGGATCCCAAGGCCAGCACCTTACCTCGGGCCCGTGAGCAGCAGCAGCCGCCGCCGCCGCCTCAGCCTCCTCTGCCGCACCCTTCAGAGCCCAAGAGCCCAGGGGAATATGTGAATATTGAATTTGGGAGTGATCAGCCTGCCTACTTATCTGGCCCTGTGGCTTCACACAGCTCACCTTCTGTCAGGTGCCCATCCCAGCTCCAGCCAGCTCCCAGAGAAGAAGAGACTGGCACTGAGGAGTACATGAAAATGGATCTCGGGCCAGGCCGGAGGGCTACCTGGCAGGAGAGTGCCGGGGTCGAGATAGGCAGAGTGGGTCCTGCACCTCCTGGGGCTGCTAGCATTTGCAGGCCTACCCGGGCGGTGCCCAGCAGCCGGGGTGACTACATGACCATGCAGATGAGTTGTCCCCGTCAGAGCTACGTGGACACGTCACCAGTCGCCCCTGTCAGTTATGCTGACATGCGGACAGGCATTGCTTCAGAGGAGGTGAGCCTGCCCAGGGCCACTGCAGCTGCATCTGCCTCATCCTCCGCAGCTGCTACTTCCTCCACTGCGCCTCAAGGGACAGCTGAGCTGGCTACCCGCTCATCCCTGCTGGGGGGCCCACAGGGACCCGGGGGCATGAGCGCCTTCACTCGGGTGAACCTCAGTCCCAACTGCAACCAGAGTGCCAAAGTGATCCGTGCAGACCCGCAAGGGTGCCGGAGGAGGCATAGCTCCGAGACCTTCTCCTCCACACCTAGTTCCACTCGGGCAAGCAACACGGTGCCCTTCGGAGCAGGGGCTGCCATAGGGGGCGGTGGCGGTGGCGGCAGCAGCAGTGAGGATGTAAAACGCCACAGCTCTGCTTCCTTTGAGAACGTCTGGCTGAGGCCTGGGGAGCATGGGGGAGCCCTTAAGGAGCCAACCCAAGTATGTGGGGCTGCTGGGGGTTTGGAGAATGGTCTTAACTACATAGACCTGGATTTGGTCAAGGACTTCAAACAGCGCCCTCAGGAGCGTCCCCCTCAGCCGCAGCCTTCCCCGCCTACACCCCCTCATCAGCCCCTGGGCAGTGGTGAGAGCAGCTCCACCCGCCGCTCCAGTGAGGATTTAAGCGCCTATGCCAGCATCAGTTTCCAGAAGCAGCCAGAGGACCGCCAGTAG

>Pro_co1 [Coquerel's sifaka (Propithecus coquereli) Irs1]

ATGGCGAGCCCTCCGGAGACCGACGGCTTCTCGGACGTGCGCAAGGTGGGCTACCTGCGCAAACCCAAGAGCATGCACAAGCGCTTCTTCGTGCTGCGGGCGGCCAGCGAGGCTGGGGGCCCAGCTCGCCTGGAGTACTACGAGAACGAGAAGAAGTGGCGGCACAAGTCGAGCGCCCCCAAACGCTCGATCCCCCTTGAGAGCTGCTTCAACATCAACAAGCGGGCTGACTCCAAGAACAAGCACCTGGTGGCTCTCTATACCCGGGACGAGCACTTTGCCATCGCCGCGGACAGCGAGGCCGAGCAAGACAGCTGGTACCAGGCTCTCCTGCAGCTGCACAACCGTGCCAAGGGCCACCACGATGGGGCTGCAGCCCCCGGGGTGGGGGGCGGCGGGGGCAGCTGCAGCGGCAGCTCTGGCCTGGGCGAGGCTGGGGAGGACTTGAGCTACGGGGATGCGCCCCCAGGACCCGCCTTCAAAGAGGTCTGGCAGGTGATCCTGAAGCCCAAGGGCCTGGGTCAGACAAAGAACCTGATTGGCATCTACCGCCTCTGCCTGACCAGCAAGACCATTAGCTTCGTGAAGCTGAACTCGGAGGCAGCGGCCGTGGTGCTGCAGCTAATGAACATCAGGCGCTGCGGCCACTCGGAGAACTTCTTCTTCATCGAGGTGGGCCGTTCCGCCGTGACGGGGCCCGGGGAGTTCTGGATGCAGGTGGATGACTCGGTGGTGGCCCAGAACATGCACGAGACCATCCTGGAGGCCATGCGGGCCATGAGTGATGAGTTTCGCCCTCGCAGCAAGAGCCAGTCCTCATCCAACTGCTCCAACCCCATCAGCGTCCCCCTGCGCAGGCACCATCTCAACAACCCCCCGCCCAGCCAGGTGGGGCTGACCCGCCGATCGCGCACGGAGAGCATCACCGCCACCTCTCCAGCCAGCATGGTGGGCGGGAAGCCAGGTTCCTTTCGCGTCCGCGCCTCCAGCGACGGGGAAGGCACCATGTCCCGCCCGGCCTCTGTGGACGGCAGCCCTGTGAGTCCTAGCACCAACAGGACCCACGCCCACCGGCATCGGGGCAGCGCCCGGCTGCACCCCCCGCTCAACCACAGCCGCTCTATCCCCATGCCTTCTTCTCGCTGCTCGCCTTCGGCCACCAGCCCCGTGAGTCTGTCGTCCAGCAGCACCAGTGGCCACGGCTCCACCTCAGACTGTCTGTTCCCACGGCGGTCTAGTGCTTCTGTGTCCGGGTCCCCCAGCGACGGCGGTTTTATCTCCTCTGATGAGTATGGCTCCAGTCCCTGCGATTTCCGAAGTTCTTTCCGCAGTGTCACTCCAGATTCCCTGGGCCACACCCCACCGGCCCGTGGTGAAGAGGAGCTGAGCAACTATATCTGCATGGGCGGCAAGGGGGCCTCCACCCTGACCGCCCCCAACGGTCACTACATTTTGTCTCGGAGTGGCAATGGCCACCGCTACATCCCAGGAGCTGGCTTGGGCACCAGTCCAGCCTTGGCTGGGGATGAAGCAGCCAGTGCTGCAGATCTGGATAATCGGTTCCGAAAGAGAACTCACTCGGCAGGCACATCCCCTACCATTTCCCACCAGAAGACCCCGTCCCAGTCCTCAGTGGCTTCCATTGAGGAGTATACAGAGATGATGCCTGCCTACCCACCAGGAGGTGGCAGTGGAGGCCGACTGCCGGGCCACCGGCACTCCGCCTTCGTACCCACCCACTCCTACCCGGAGGAGGGTCTGGAAATGCACCCCTTGGAGCCTCAGGGTGGCCACCACCGCCCAGATACCTCCACCCTCCACACCGACGATGGCTATATGCCCATGTCCCCGGGGGTGGCCCCAGTGCCCGGCAGCAGAAAGGGCAGTGGAGACTATATGCCCATGAGCCCCAAGAGCGTGTCTGCCCCCCAGCAGATCATCAACCCCATCAGACGCCATCCCCAGAGAGTGGACCCCAATGGCTACATGATGATGTCCCCCAGCGGCAGCTGCTCCCCTGACCTTGGAGGTGGGCCCAGCAGCAGCAGCAGTGCTGCCCCCTCTGGGAGCAGCTATGGGAAGCTTTGGACAAATGGTGTAGGGAGCCACCACTCTCCAGCCCTGCCCCACCCCAAGCCCCCCGTGGAGAGCAGTGGTGGCAAGCTCTTGCCTTGCACAGGTGACTACATGAACATGTCGCCAGTGGGGGACTCCAATACCAGCAGCCCCTCCGGCTGCTACTATGGCCCTGAGTACCCCCAGCAAAAGCCAGTCCTTTCCTACTACTCATTGCCAAGGTCCTTTAAGCACACCCAGCGTCCTGGGGAGCCGGAGGAGGGTGCCCGGCACCACCACCTCCGCCTTTCCTCTAGCTCTGGTCGCCTTCTCTATGCAGCAACAGCAGAAGATTCTTCCTCCTCCACCAGCAGCGACAGCCTTGGTGGGGGATACTGTGGGGCTAGGCTGGAGCCTGGTCTCCCGCATGCCCACCATCAGGTCCTGCAGCCCCATCTGCCTCGGAAAGTGGACACAGCTGCCCAGACCAACAGCCGCCTGGCTCGGCCCACGAGGCTATCCCTGGGGGATCCCAAGGCCAGCACCTTACCTCGGGCCCGAGAGCAGCAGCAGCCCCTGCTACACCCTCCAGAGCCCAAAAGCCCAGGGGAATATGTGAATATTGAATTTGGGAGTGATCAGCCTGGCTATTTATCTGGCCCCATGGCTTCCCACAGCTCGCCTTCGGTCAGGTGTCCATCCCAGCTCCAGCCAACTCCCAGAGAGGAAGAGACTGGCACTGAGGAGTACATGAAAATGGACCTGGGGCCGGGCCGGAGGGCAGCCTGGCCGGAGAGCTCCAGGGTTGAGATGGGCAGAGTGGGCCCCACACCTCCCGGGGCTACTAGTGTTTGCAGGCCCACCCGGGCAGTGCCCAGCAGCCGGGGTGACTACATGACCATGCAGATGGGTTGTCCCCGTCAGAGCTATGTGGACACCTCCCCGGTTGCCCCTGTCAGCTATGCCGACATGCGGACAGGCATGGCTGCACAGGAGGTGAGCCTGCCCAGGGCTGCCACGGCTGCTCCCTCCTCATCCTCAGCAGCCTCTGCTTCTCCTGCTGCACCTCAAGGGACAGCCGAGCTGGCTGCCCGATCATCCCTGCTAGGGGGCCCGCAGGGACCCGGGGGCATGAGTGCCTTCACCCGGGTGAACCTCAGTCCCAACCGCAACCAGAGTGCCAAAGTGATCCGTGCAGACCCACAAGGGTGCCGGAGGAGGCATAGCTCCGAGACCTTCTCCTCCACACCTAGTGCCACCCGGGTGGGCAACACAGTGCCCTTCGGAGGGGGGGCTGCAGCAGGGGCTAGCAGCGGAGGCGGCAGCAGCAGTGAGGATGTGAAACGCCACAGCTCTGCTTCCTTTGAGAATGTGTGGCTGAAGCCTGGGGAGCTTGGGGAAGCCCCCAAGGAGTCAGCCCAAGTGTGTGGAGCTGCCGGGGGTTTGGAGAATGGTCTTAACTATATAGACCTGGATTTGGTCAAGGACTTCAAGCAGCGCCCTCAGGAGCGCACCCCTCAACCGCAGCCTCCCCCACCAACGCCCCCTCATCAGCCCCTGGGCAGCGGCGAGAGCAGCTCCACCCGCCGCTCCAGTGAGGATTTAAGCGCCTATGCCAGCATCAGTTTCCAGAAGCAGCCAGAGGACCGCCAGTAG

>Mic_mu1 [Mouse lemur (Gray mouse lemur) Irs1]

ATGGCGAGCCCTCCGGAGACCGACGGCTTCTCGGACGTGCGCAAGGTTGGCTACCTGCGCAAACCCAAGAGCATGCACAAGCGCTTCTTCGTGCTGCGGGCGGCCAGCGAGGCAGGGGGCCCGGCGCGCCTGGAGTACTACGAGAACGAGAAGAAGTGGCGGCACAAGTCGAGCGCCCCCAAACGCTCGATCCCCCTTGAGAGCTGCTTCAACATCAATAAGCGGGCTGACTCCAAGAACAAGCACCTGGTGGCTCTCTATACCCGGGACGAGCACTTTGCCATCGCGGCGGACAGCGAGGCTGAGCAAGACAGCTGGTACCAGGCTCTCCTGCAGCTGCACAACCGTGCCAAGGGCCACCACGATGGGGCTGCAGCATCCGGGCTGGGAGGCGGCGGGGGCAGCTGCAGCGGCAGCTCTGGCCTGGGCGAGGCTGGGGAGGACTTGAGCTACGGGGATGCGCCCCCAGGACCCGCCTTCAAAGAGGTCTGGCAGGTGATCCTGAAGCCCAAGGGCCTGGGTCAGACAAAGAACCTGATTGGCATCTACCGCCTCTGCCTGACCAGCAAGACCATCAGCTTCGTGAAGCTGAACTCGGAGGCAGCCGCCGTGGTGCTGCAGCTAATGAACATCAGGCGCTGCGGCCACTCGGAGAACTTCTTCTTCATCGAGGTGGGCCGTTCCGCCGTGACGGGGCCGGGGGAGTTTTGGATGCAGGTGGATGACTCGGTGGTGGCCCAGAACATGCACGAGACCATCCTGGAGGCCATGCGGGCCATGAGTGATGAGTTCCGCCCTCGCAGCAAGAGCCAGTCCTCATCCAACTGCTCCAACCCCATCAGCGTCCCCCTGCGCAGGCACCATCTCAACAACCCCCCGCCCAGCCAGGTGGGGCTGACCCGCCGATCGCGCACCGAGAGCATCACCGCCACCTCCCCAGCCAGCATGGTGGGCGGAAAGCCGGGTTCCTTCCGCGTCCGCGCCTCCAGCGACGGCGAAGGCACCATGTCACGCCCGGCCTCGGTGGACGGCAGTCCTGTGAGTCCTAGCACTAACAGGACCCACGCCCACCGGCATCGGGGCAGCGCCCGGCTGCACCCCCCACTCAACCACAGCCGCTCTATCCCCATGCCTTCTTCTCGCTGCTCGCCTTCGGCCACCAGCCCGGTCAGTCTGTCGTCCAGCAGCACCAGTGGCCACGGCTCCACCTCGGACTGCCTGTTCCCACGGCGGTCTAGTGCTTCTGTGTCCGGGTCCCCCAGCGATGGCGGTTTCATCTCCTCGGACGAGTATGGCTCCAGTCCTTGCGATTTCCGAAGCTCTTTCCGCAGTGTCACTCCAGATTCCCTGGGCCACACCCCACCGGCCCGTGGTGAAGAGGAGCTGAGCAACTATATCTGCATGGGCGGCAAGGGGGCCTCCACCCTGACCGCCCCCAACGGTCACTACATTTTGTCTAGGGGTGGCAATGGCCACCGCTACATCCCGGGAGCTGGCTTGGGCACCAGTCCAGCCTTGGCTGGGGATGAAGCAGCCAGTGCTGCAGATCTGGATAATCGTTTCCGAAAGAGAACTCACTCGGCAGGCACATCCCCTACCATTTCCCACCAGAAGACCCCGTCCCAGTCCTCAGTGGCTTCCATCGAGGAGTATACGGAGATGATGCCTGCCTACCCACCAGGAGGTGGCAGTGGAGGCCGACTGCCGGGCCACCGGCACTCCGCCTTCGTGCCCACCCACTCCTACCCGGAGGAGGGTCTGGAAATGCACCCCATGGAGCCTCAGGGGGGCCACCACCGCCCAGATACCTCCACCCTCCACACCGACGATGGCTATATGCCCATGTCCCCGGGGGTGGCCCCAGTGCCCAGCAGCAGAAAGAGCAGTGGGGACTATATGCCCATGAGCCCCAAGAGCGTGTCTGCCCCCCAGCAGATCATCAACCCCATCAGACGCCATCCCCAGAGAGTGGACCCCAATGGCTACATGATGATGTCCCCCAGCGGCAGCTGCTCCCCTGACCTTGGAGGTGGGCCCAGCAGCAGCAGCAGTGCTGCCCCCTCTGGGAGCAGCTACGGGAAGCTTTGGACAAATGGTGTAGGGGGCCACCACTCTCCCGCCCTGCCCCACCCCAAGCCTCCAGTGGAGAGCAGTGGTGGCAAGCTCTTGCCTTGCACAGGTGACTACATGAACATGTCGCCAGTGGGGGACTCCAATACCAGCAGCCCCTCCGGCTGCTACTACGGCCCTGAATACCCCCAGCACAAGCCAGTCCTCTCCTACTACTCATTGCCAAGGTCCTTTAAGCACACTCAGCGTCCTGGGGAGCCAGAGGAGGGTGCCCGGCACCAGCACCTCCGCCTTTCCTCTAGCTCTGGTCGCCTTCTCTATGCTGCAGCGGCAGAAGATTCTTCCTCCTCCACCAGCAGCGACAGCCTTGGTGGGGGCTACTGTGGGGCTAGGCTGGAGCCTGGTCTCCCGCATGCCCACCACCATCAGGTCTTGCAGCCCCATCTGCCTCGGAAAGTGGACACAGCCGCCCAGACCAACAGCCGCCTGGCTCGGCCCACGAGGCTATCCCTGGGGGATCCTAAGGCCAGCACCTTACCTCGGGCCCGAGAGCAGCAGCAGCCCCTGCTGCACCCTCCGGAGCCCAAAAGCCCAGGGGAATATGTGAATATTGAATTTGGGAGTGATCAGCCTGGCTATTTATCTGGCCCCGTGGCTTCCCACAGCTCGCCTTCAGTCAGGTGTCCATCCCAGCTCCAGCCAACTCCCAGAGAGGAAGAGACTGGCACTGAGGAGTACATGAAAATGGACCTGGGGCCGGGCCGGAGGGCAGCCTGGCAGGAGAGCTCCGGGGTTGAGATGGGCAGAGTGGGCCCCGCACCTCCTGGGGCTACGAGCGTTTGCAGGCCCACCCGGGCAGTACCCAGCAGCCGTGGCGACTATATGACCATGCAGATGGGTTGTCCCCGTCAGAGCTACGTGGACACCTCCCCAGTTGCCCCGGTCAGCTATGCTGACATGCGGACAGGCATTGCTGCACGGGAGGTGAGCCTGCCCAGGGCTGCCACGGCTGCTCCCTCCTCATCCTCAGCAGCCTCTGCTTCTCCTGCTGCACCTCAAGGGACAGCTGAGCTGGCTGCCCAAGCATCCCTGCTAGGGGGCCCACAGGGACCCGGGGGCATGAGCGCCTTCACCCGGGTGAACCTCAGTCCCAACCGAAACCAGAGTGCCAAAGTGATCCGTGCAGACCCACAAGGGTGCCGGAGGAGGCATAGCTCCGAGACCTTCTGCTCCACACCTAGTGCCACCCGGGCGGGCAACACAGTGCCCTTAGGAGGGGGGGCTGCAGTAGGGGCTAGCAGCGGAGGCGGCAGCAGCAGCAGTGAAGATGGGAAACGCCACAGCTCTGCTTCCTTTGAGAATGTGTGGCTGAAGCCTGGGGAGCTTGGGGAAGCCCCCAAGGAGTCAGCCCAAGTGTGTGGGGCTGCTGGGGGTTTGGAGAATGGTCTTAACTACATAGACCTGGATTTGGTCAAGGACTTCAAGCAGCGCTCTCAGGAGCGCCCTGCTCAACCACAGCCTCCCCTACCAACACCCCCTCGTCAGCCCCTGGGCAGCGGGGAAAGCAGCTCCACCCGCCGCTCCAGTGAGGATTTAAGCGCCTATGCCAGCATCAGTTTCCAGAAGCAGCCAGAGGACCGCCAGTAG

>Oto_ga1 [Galago (Otolemur garnettii) Irs1]

ATGGCGAGCCCTCCGGAGACCGACGGCTTCTCGGACGTGCGCAAGGTTGGCTACCTGCGCAAACCCAAGAGCATGCACAAGCGCTTTTTCGTGCTGCGAGCGGCCAGCGAGGCTGGGGGCCCGGCGCGCCTGGAGTACTACGAGAACGAGAAGAAGTGGCGGCACAAGTCGAGCGCCCCCAAACGCTCGATCCCCCTTGAGAGCTGCTTCAACATCAACAAGCGGGCTGACTCCAAGAACAAGCACCTGGTGGCTCTCTACACCCGGGACGAACACTTCGCCATCGCAGCCGACAGTGAGGCCGAGCAAGACAGCTGGTACCAGGCTCTCCTGCAGCTGCACAACCGTGCCAAGGGCCACCACGATGCGGCTGCAGCTCCAGGAGTGGGAGGCGGTGGGGGCAGCTGCAGCAGCAGCTCAGCCCTGGGCGAGGCTGGGGAGGACTTGAGCTATGGGGATGTGCCCCCAGGACCTGCCTTCAAAGAGGTCTGGCAGGTGATCCTGAAGCCCAAGGGCCTGGGTCAGACAAAGAACCTAATTGGCATCTACCGCCTCTGCCTGACCAGCAAGACCATCAGCTTCGTGAAGCTGAACTCGGAGGCCGCTGCCGTGGTGCTGCAGCTGATGAACATCAGGCGCTGCGGCCACTCGGAGAACTTCTTCTTCATCGAGGTGGGCCGTTCCGCTGTGACAGGGCCTGGGGAATTCTGGATGCAGGTGGATGACTCGGTGGTGGCCCAGAATATGCATGAGACCATCCTGGAGGCCATGCGGGCTATGAGTGATGAGTTCCGCCCTCGCAGCAAGAGCCAGTCCTCATCCAACTGCTCCAACCCCATCAGCGTCCCCCTGCGCAGGCACCATCTCAACAACCCCCCACCCAGCCAGGTGGGGCTGACCCGCCGGTCACGCACAGAGAGCATCACCGCCACTTCCCCTGCCAGCATAGTGGGTGGGAAGCCAGGTTCCTTTCGTGTCCGCGCCTCTAGCGACGGAGAAGGCACGATGTCCCGCCCGGCCTCTGTGGATGGTAGCCCTGTGAGCCCGAGCACCAACAGGACACATGCCCACCGGCATCGGGGCAGCGCCCGGCTGCACCCCCCACTCAACCACAGTCGCTCCATCCCCATGCCTTCCTCTCGATGCTCGCCTTCAGCCACCAGCCCGGTCAGTCTGTCGTCCAGCAGCACCAGTGGCCATGGCTCCACCTCGGACTGTCTCTTCCCGCGGCGGTCTAGCGCTTCTGTGTCTGGTTCCCCCAGTGATGGCGGTTTCATCTCCTCGGATGAGTATGGGTCTAGTCCCTGCGATTTCCGAAGTTCCTTCCGCAGTGTCACTCCAGATTCCCTAGGCCACACCCCACCAGCCCGTGGTGAGGAGGAGCTGAGCAACTATATCTGCATGGGTGGCAAGGGGACCTCCACCCTGACTGCCCCCAATGGCCACTACATTTTGTCCCGGGGTGGCAATGGCCACCGCTGCATTCCGGGAGCTGGCTTGGGCACCAGTCCAGCCTTGGCTGGGGATGAAGCAGCCTGCGCTGCAGATCTGGATAATCGGTTCCGAAAGAGAACTCACTCGGCAGGCACATCCCCCACCATTTCCCACCAGAAGACCCCGTCCCAGTCATCGGTGGCTTCCATTGAGGAGTATACGGAGATGATGCCCGCCTACCCACCAGGAGGTGGCAGTGGAGGCCGCCTGCCGGGCCACCGGCACTCCGCCTTTGTGCCCACCCACTCTTACCCTGAGGAGGGTCTGGAAATGCACCCCTTGGAGCGTCAAGGGGGCCATCACCGCCCCGACACCTCCAGCCTCCACACTGACGATGGCTATATGCCCATGTCTCCAGGGGTGGCCCCAGTGCCTGGCAGCAGAAAGGGCAGTGGGGACTATATGCCCATGAGCCCCAAGAGCGTGTCTGCCCCCCAGCAGATCATCAACCCCATCAGACGCCATCCCCAGAGAGTGGACCCCAATGGCTACATGATGATGTCACCCAGTGGCAGCTGCTCCCCTGACCTTGGAGGTGGGCCCAGCAGCAGCAGCAGCAGCAGTGCTGCCCCCTCTGGGAGCAGCTATGGGAAGCTTTGGACAAATGGTGTTGGAGGCCACCACTCTCAAGCCCTGCCCCACCCCAAACCCCCTGGGGAAAGCAGCAGTGGCAAGCTTTTGCCTTGCACAGGGGACTACATGAACATGTCGCCAGTGGGGGACTCCAACACCAGCAGCCCCTCCAGCTGCTACTATGGCCCTGAGTACCCCCAGCACAAGCCAGTCCTCTCCTACTACTCATTGCCAAGGTCATTTAAGCACACCCAGCGCCCTGGGGAGTTAGAGGAGGGTGCCCGGCACCAGCACCTCCGCCTTTCCTCCAGCTCTGGCCGCCTTCTCTACGCTGCAGCAGCAGAAGATTCTTCTTCTTCCACCAGCAGCGACAGCCTGGGTGGGGGATACTGTGGGGCTAGGCAGGAGCCAGGACTCCCACATGCCCACCATCAAGTCTTGCAGCCCCATCTGCCTCGAAAAGTGGACACAGCTGCACAGACCAACAGTCGCCTGGCTCGGCCCACGAGGCTGTCCCTGGGGGATCCCAAGGCCAGCACCTTACCTCGGGCCCGAGAGCAGCAGCAGCCCCTGTTACACCCTGAGCCCAAGAGCCCAGGAGAATATGTGAATATTGAATTTGGGAGTGATCAGCCTGGCTATTTGTCTGGCCCGGTGGCTTCCCACAGCTCACCTTCAGTCAGGTGTCCATCCCAGCTCCAACCAATTCCCAGGGAGGAAGAGACTGGCACTGAGGAGTACATGAAAATGGACCTGGGGCCAGGCCGAAGGGCAGCGTGGCAGGAGAGCACTGGTGTTGAGATGGGCAGAGTGGGCCCAGCGCCTCCTGGGGCTGCTAGTGTTTGCAGGCCCACCCGAGCAGTACCCAGCAGTCGAGGTGACTACATGACCATGCAGATGGGTTGTCCTCGTCAGAACTACGTGGACACCTCCCCAGTTGCCCCTGTCAGCTATGCTGACATGCGGACAGGCATTGCTGCAGAGGTGAGCCCACCCCGGGCTACCACAGCTGCTCCCTCGTCCTCAGCTGCCTCTGCTTCCCCTGCTGCACCTCAAGGGACAGCTGAACTGGCTGCTCGCTCATCCCTGCTAGGGGGCCCCCAGGGACCCGGGGGTATGAGCGCCTTCACCCGGGTAAACCTCAGTCCCAGCTGCAACCAGAGTGCCAAAGTGATCCGTGCAGACCCACAAGGGTGCCGGAGGAGGCATAGCTCGGAGACCTTCTCCTCTAAACCTAGTGCCACCCGGGCGGCCAACACCGTGCCCTTTGGAGGAGGGGCTGTAGTAGGGAGTAGTGGTGGCAGCAGCAGTGAGGATGTGAAACGCCACAGTTCTGCTTCCTTTGAGAATGTGTGGCTGAAGCCTGGGGAGCTTGGGGGAGCCCCCAAAGAACCAACCCAAGTGTGTGGGGCTGCTGGGGGTTTGGAGAATGGTCTTAACTATATAGACCTGGATTTGGTCAAGGACTTCAAGCAGCGCCCTCAGGAGCGCCCCCCTCAAGCGCAGCCTCCCCCACCCCCATCCCCTCATCAGCCCCTGGGCAGCGGTGAGAGCAGCTCCACCCGCCGCTCCAGTGAGGATTTAAGCGCCTATGCCAGCATCAGTTTCCAGAAGCAGCCAGAGGCCCGCCAGTAG

>Gal_va1 [Flying lemur (Galeopterus variegatus) Irs1]

ATGGCGAGCCCTCCGGAGACCGACAGCTTCTCGGACGTGCGCAAGGTGGGCTACCTGCGCAAACCCAAGAGCATGCACAAGCGCTTCTTCGTGCTGCGGGCGGCCAGCGAGGCGGGGGGCCCGGCGCGCCTCGAGTACTACGAGAACGAGAAGAAGTGGCGGCACAAGTCGAGCGCCCCCAAACGCTCGATCCCCCTCGAGAGCTGCTTCAACATCAACAAGCGGGCTGACTCCAAGAACAAGCACCTGGTGGCTCTCTACACCCGGGACGAACACTTTGCCATCGCGGCGGACAGCGAGGCCGAGCAAGACAGCTGGTACCAGGCTCTCCTGCAGCTGCACAACCGCGCTAAGGGCCACCACGATGGCGCCTCCGCCCCGGGGACTGGAGGCGGTGGGGGCAGCGGCAGCTCCGGCGTCGGGGAGGCTGGGGAGGACCTGAGCTATGGGGACGTGCCCCCAGGACCCGCGTTCAAGGAGGTCTGGCAGGTCATCCTAAAACCCAAGGGCCTGGGACAGACGAAGAACCTTATTGGCATCTACCGCCTATGCCTGACCAGCAAGACCATCAGCTTCGTGAAGCTGAACTCGGAGGCGGCGGCCGTGGTGCTGCAGCTGATGAACATCAGGCGCTGCGGCCACTCGGAGAACTTCTTCTTCATCGAGGTGGGCCGTTCCGCCGTGACCGGGCCCGGGGAGTTCTGGATGCAGGTGGATGACTCGGTGGTGGCCCAGAACATGCACGAGACCATTCTGGAGGCCATGCGGGCCATGAGCGATGAGTTCCGCCCTCGAAGCAAGAGCCAGTCTTCGTCCAACTGCTCCAACCCCATCAGCGTCCCCCTGCGCAGGCACCATCTCAACAACCCCCCGCCCAGCCAGGTGGGGCTGACCCGCAGGTCGCGCACGGAGAGCATCACCGCCACCTCCCCGGCCAGCATGGTGGGCGGGAAGCCGGGCTCCTTCCGTGTCCGCGCCTCCAGCGACGGCGAAGGCACTATGTCCCGCCCAGCCTCAGTGGACGGCAGCCCTGTGAGTCCCAGCACCAATCGGACCCACGCTCACCGTCATCGGGGCAGCTCCCGGCTGCACCCGCCGCTCAACCACAGCCGTTCCATCCCCATGCCTTCTTCTCGCTGCTCGCCTTCGGCCATCAGCCCGGTCAGTCTTTCGTCCAGCAGCACCAGTGGCCACGGCTCCACCTCGGACTGTCTCTTCCCACGGCGGTCTAGTGCATCTGTGTCCGGTTCCCCCAGCGATGGTGGTTTCATCTCCTCCGATGAGTATGGATCCAGTCCCTGTGATTTCCGAAGTTCCTTCCGCAGTGTCACTCCGGATTCCCTGGGCCACACCCCACCGGCCCGTGGTGAGGAGGAGCTGAGCAACTATATCTGCATGGGTGGCAAAGGAGCCTCCACTCTGGCTGCCCCCAATGGTCACTACATTTTGTCTCGAGGTGGCAACGGCCACCGCTGCCTCCCAGGAGCTGGCTTGGGCACCAGCCCAGCCTTGGCTGGGGATGAAGCATCCAGTGCGGCAGAGTTGGATAATAGGTTCCGAAAGAGAACTCACTCGGCGGGCACATCCCCTACCATTTCCCACCAGAAGACACCGTCCCAGTCTTCAGTGGCTTCCATTGAGGAGTATACAGAGATGATGCCTGCCTACCCACCAGGAGGTGGCAGTGGAGGCCGGCTACCGGGCTACCGGCACTCCGCCTTCGTGCCCACCCACTCCTACCCTGAGGAGGGTCTGGAAGGGCACCCCTTGGAGCGTCGTGGGGGCCACCACCGCCCAGACACCTCCACCCTCCACACTGATGATGGTTACATGCCCATGTCCCCAGGGGTGGCCCCCGTGCCTGCCAGCCAAAAGGGTAGTGGGGACTATATGCCCATGAGCCCCAAGAGCGTGTCTGCCCCACAGCAGATCATCAATCCTATCAGGCACCATCCCCAGAGAGTGGACCCCAATGGCTACATGATGATGTCCCCCAGTGGCAGCTGCTCCCCTGACATTGGAGGTGGGCCCAGCAGCAGAACTGCCCCTTCTGGGAGCAGCTATGGGAAGCTGTGGACCAATGGGGTAGGGGGCCACCACTCTCACGCCCTGCCCCACTCCAAACCCCCAGTGGAGAACAGTGGTAGCAAGCTCTTGCCTTGCACAGGTGACTACATGAACATGTCACCAGTGGGGGACTCCAACACCAGCAGCCCCTCCGACTGCTACTATGGCCCTGAGGACCCCCAGCACAAGCCAGTCCTCTCCTACTACTCATTGCCAAGGTCCTTTAAGCACACCCAGCGCCCTGGGGAGCCGGAGGACGGTGCCCGGCACCAGCATCTCCGTCTTTCCTCTAGCTCTGGTCGCCTTCTTTATGCTGCAGCAGCGGAAGATTCTTCCTCCTCCACCAGCAGCGACAGCCTGGGTGGGGGATACTGTGGGGCTAGGCTGGAGCCCGGCCTCCCACATCCCCACCGTCAGGTCTTGCAGCCCCATCTGCCTCGAAAGGTGGATACAGCTGCCCAAACCAACAGCCGCCTGGCTCGGCCCACGAGGTTGTCCCTGGGGGATCCCAAGGCCAGCACTTTACCACGGGTCCGAGAGCAGCAGCAACAACCCCTGTTGCACCCTCCGGAGCCCAAGAGCCCAGGGGAATATGTGAATATTGAATTCGGGAGTGATCAGCCTGGCTACTTATCTGGCCCTGTGGCTTCCCGCAGCTCTCCTTCCATCAGGTGTCCATCCCAGCTCCAGCCAGCTCCCAGAGAGGAAGAGACTGGCACCGAGGAGTACATGAACATGGACCTGGGGCCAGGCCGGAGGGCATCCTGGCGAGAGAGAGCTGGTGCGGAGATGGGCAGAGTGGGCCCTGTACCTCCAGGGGCTGCTAGCGTTTGCAGGCCCACCCGGGCGGTGCCCAGCAGCCGGGGTGACTACATGACCATGCAGATGGGTTGTCCCCGTCAGAGCTACGTGGACACCTCACCGGTCACCCCTGTCAGCTATGCTGACATGCGGACAGGCATTGCTGCAGAGGAGGCAAGCCTGCCCAGGGCCACAGCAGCTGCTCCCTCCTCATCCTCAGCAGCTACTGCTCCCCCTGCTGCGCCTCAAGGAGCAGCTGAGCTGGCTGCCCGCTCTTCCCTGCTGGCCCCGCAGGGACCCGGGGGCATGAGTGCCTTCACCCGGGTGAACCTCAGTCCCAACCGCAACCAGAGTGCCAAAGTGATCCGTGCAGACTCGCAAGGTTGCCGGAGGAGGCATAGCTCCGAGACCTTCTCCTCGACACCTAGTGCTACTCGGGCAGGCAGTGCGGTGACCTTCGGAGGGGTGACTGCAGTAGGAGGCGGTGGTGGTGGTGGTGGCAGCAGCAGCAGTGAGGATGTAAAACGCCACAGCTCTGCTTCTTTTGAGAATGTGTGGCTGAGGCCTGGGGAGCTTGGGGGATCCTCCAAGGAGCAAGCCCAAGGGTGTGGGGCTTCTGGAGGTTTGGAGAATGGTCTTAACTACATAGACCTGGATTTGGCCAAGGACGTCAAACAACGTCCTCAGGAGCGCCCCCCTCAACCGCAGCCACCCCCACCCCCGCCCACTCATCAGCCCTTGGGCAGCAGTGAGAGCATCGCGACCCACCGCTCCAGTGAGGATTTAAGCACCTACGCCAGCATCAGTTTCCAGAAGCAGCCAGAGGACCGCCAGTAG

>Mus_mu1 [Mouse (Mus musculus) Irs1]

ATGGCGAGCCCTCCGGATACCGATGGCTTCTCAGACGTGCGCAAGGTGGGCTACCTGCGCAAGCCCAAGAGTATGCATAAGCGCTTTTTCGTGCTGCGGGCGGCCAGCGAGGCCGGGGGCCCAGCGCGCCTGGAGTATTATGAGAACGAGAAGAAGTGGCGGCACAAGTCGAGCGCCCCCAAACGCTCGATCCCCCTCGAGAGCTGTTTCAACATCAACAAGCGGGCTGACTCCAAGAACAAGCACCTGGTGGCTCTCTACACCCGAGACGAACACTTTGCCATTGCCGCGGATAGCGAGGCTGAGCAAGACAGCTGGTACCAGGCTCTTCTGCAGCTGCATAATCGGGCAAAGGCCCACCATGACGGGGCTGGAGGAGGCTGCGGCGGTAGCTGCAGCGGCAGCTCTGGCGTTGGAGAGGCAGGGGAGGACTTGAGCTATGACACGGGTCCAGGACCCGCGTTCAAGGAGGTCTGGCAGGTTATCCTGAAACCCAAGGGCCTGGGTCAGACAAAGAACCTGATTGGCATCTACCGCCTCTGCCTGACCAGCAAGACCATCAGCTTTGTGAAGCTGAACTCCGAGGCAGCCGCTGTGGTGCTGCAGCTGATGAATATCAGACGCTGTGGCCACTCAGAGAACTTCTTCTTCATCGAGGTGGGGCGTTCCGCTGTGACAGGGCCTGGCGAGTTCTGGATGCAAGTGGATGACTCCGTGGTGGCCCAGAACATGCATGAGACCATTCTAGAGGCAATGAGGGCCATGAGCGATGAGTTTCGCCCTCGCAGCAAAAGCCAGTCTTCATCCAGTTGCTCCAACCCCATCAGCGTTCCCCTGCGCAGGCACCATCTCAACAACCCTCCACCCAGCCAGGTGGGACTGACTCGGAGATCTCGAACTGAGAGCATCACTGCCACCTCCCCTGCCAGTATGGTGGGTGGGAAACCAGGTTCCTTCCGGGTGCGTGCCTCCAGCGATGGCGAAGGCACCATGTCCCGTCCAGCATCAGTGGATGGCAGTCCTGTGAGCCCTAGCACCAACAGGACCCACGCCCATCGGCATCGAGGCAGCTCCAGGCTGCACCCCCCACTCAACCACAGCCGCTCCATCCCCATGCCTTCTTCTAGATGCTCACCTTCAGCCACCAGCCCAGTGAGTCTGTCATCTAGTAGTACCAGTGGCCATGGCTCCACTTCAGACTGTCTCTTCCCGAGGCGCTCTAGTGCTTCCGTGTCCGGTTCGCCTAGCGATGGCGGTTTCATCTCTTCTGATGAGTATGGTTCTAGTCCCTGCGATTTCCGAAGTTCCTTCCGCAGTGTCACCCCAGATTCCCTGGGGCACACCCCACCAGCCAGGGGTGAGGAGGAGCTGAGCAATTATATCTGCATGGGTGGCAAGGGAGCCTCCACCTTGGCTGCTCCCAATGGCCACTACATTTTGTCTAGGGGTGGCAACGGCCATCGCTACATCCCAGGTGCTAACTTGGGGACAAGCCCAGCGCTGCCTGGAGATGAAGCCGCGGGTGCAGCAGATCTGGATAACCGGTTTCGAAAGAGAACTCACTCCGCAGGCACATCTCCTACCATTTCCCATCAGAAGACCCCCTCACAGTCTTCAGTGGCTTCTATTGAGGAATATACAGAGATGATGCCCGCTGCCTACCCACCAGGAGGTGGCAGTGGAGGCCGACTGCCCGGCTACCGGCATTCCGCCTTCGTGCCCACCCACTCCTATCCCGAAGAGGGTCTAGAGATGCACCACTTGGAACGTCGTGGAGGCCACCACCGTCCAGACACCTCCAACCTCCACACTGATGATGGCTATATGCCCATGTCTCCTGGGGTGGCTCCAGTGCCCAGCAACCGCAAAGGAAATGGGGACTATATGCCCATGAGCCCCAAGAGTGTATCTGCCCCACAGCAGATCATTAACCCCATCAGACGCCACCCACAGAGAGTGGACCCCAATGGCTACATGATGATGTCACCCAGTGGTAGTTGCTCCCCTGACATTGGAGGTGGGTCCAGCAGCAGTAGCAGCATCAGCGCAGCCCCTTCTGGGAGCAGCTATGGGAAGCCATGGACAAATGGAGTAGGGGGGCACCATACTCATGCCCTTCCTCATGCCAAACCTCCTGTTGAGAGTGGTGGCGGTAAGCTCTTGCCTTGCACAGGTGACTACATGAACATGTCCCCAGTGGGAGATTCCAACACCAGCAGCCCCTCAGAATGCTACTATGGCCCAGAAGATCCCCAGCACAAGCCGGTCCTCTCTTACTACTCATTGCCAAGGTCCTTTAAGCACACCCAGCGCCCTGGAGAGCCAGAGGAGGGTGCCAGGCACCAGCATCTTCGTCTCTCTTCTAGCTCTGGACGCCTTCGCTATACCGCAACTGCCGAAGATTCCTCCTCTTCTACCAGCAGCGACAGTCTGGGTGGGGGTTACTGTGGGGCTAGGCCAGAGTCAAGCCTCACACATCCCCACCACCATGTCTTGCAGCCCCACCTGCCTCGAAAGGTAGACACAGCTGCACAGACCAACAGCCGCCTGGCTCGACCCACAAGGCTGTCCTTGGGGGATCCCAAGGCAAGCACCTTACCCCGGGTTCGAGAGCAGCAACAGCAGCAGCAGTCTTCCCTGCACCCTCCCGAGCCCAAAAGCCCAGGAGAATATGTGAATATTGAATTCGGGAGCGGCCAGCCTGGCTATTTAGCTGGCCCTGCAACTTCCCGTAGCTCTCCTTCAGTTCGATGTCCACCCCAGCTCCACCCAGCTCCTAGAGAAGAGACTGGCTCGGAAGAGTACATGAACATGGACTTGGGGCCAGGCCGGAGGGCAACCTGGCAGGAGAGTGGTGGAGTTGAGTTGGGCAGAATAGGCCCTGCACCTCCGGGGTCTGCTACGGTTTGCAGGCCAACCCGTTCGGTGCCAAATAGCCGTGGTGACTACATGACCATGCAGATAGGTTGTCCTCGTCAAAGCTATGTGGATACCTCACCAGTGGCCCCAGTCAGCTATGCTGACATGCGGACAGGCATTGCTGCAGAGAAGGCGAGCCTGCCTAGACCCACAGGAGCTGCTCCTCCTCCATCCTCCACAGCCTCTTCTTCTGTTACACCTCAAGGAGCCACCGCTGAGCAAGCTACTCACTCTTCCTTGCTGGGAGGCCCTCAGGGACCTGGGGGCATGAGTGCATTCACCAGGGTGAACCTCAGTCCCAACCATAACCAGAGTGCCAAAGTGATTCGCGCAGACACTCAAGGGTGCCGGAGGAGGCATAGCTCTGAGACCTTCTCAGCACCTACTCGGGCTGGCAATACGGTGCCCTTTGGAGCAGGGGCTGCAGTAGGGGGCAGCGGCGGTGGTGGTGGTGGCGGCAGTGAGGATGTAAAACGCCACAGCTCTGCATCCTTTGAGAATGTGTGGCTGAGACCTGGGGATCTAGGGGGAGTCTCCAAGGAGTCGGCTCCAGTGTGTGGGGCTGCTGGGGGTTTGGAGAAGAGTCTTAACTACATAGACCTGGATTTGGCCAAGGAGCGCTCTCAGGACTGCCCCTCTCAACAGCAGTCCCTACCACCCCCTCCCCCTCACCAGCCCTTAGGCAGCAATGAGGGCAACTCCCCAAGACGCTCCAGTGAGGATTTAAGCAACTATGCCAGCATCAGCTTCCAGAAGCAGCCAGAGGATCGTCAATAG

>Rat_no1 [Rat (Rattus norvegicus) Irs1]

ATGGCGAGCCCTCCGGATACCGATGGCTTCTCAGACGTGCGCAAGGTGGGTTACCTGCGCAAACCCAAGAGTATGCATAAGCGCTTTTTCGTGCTGCGGGCGGCCAGCGAGGCCGGGGGCCCGGCGCGCCTGGAGTATTATGAGAACGAGAAGAAGTGGCGGCACAAGTCGAGCGCCCCCAAACGCTCGATCCCCCTCGAGAGCTGTTTCAACATCAACAAGCGGGCTGACTCCAAGAACAAGCACCTGGTGGCTCTCTACACCCGAGACGAACACTTTGCCATTGCGGCGGATAGCGAGGCTGAACAAGACAGCTGGTACCAGGCTCTTCTGCAGCTGCATAATCGGGCAAAGGCCCACCATGACGGGGCTGGAGGAGGCTGCGGTGGTAGCTGCAGCGGCAGCTCTGGCGTCGGAGAGGCAGGGGAGGACTTGAGCTATGACACGGGCCCAGGACCCGCGTTCAAGGAGGTCTGGCAGGTTATCCTGAAACCCAAGGGCTTAGGTCAGACAAAGAACTTGATTGGTATCTACCGCCTCTGCCTGACCAGCAAGACCATCAGCTTTGTGAAGCTCAACTCTGAGGCTGCCGCTGTGGTGCTGCAGCTGATGAACATCAGACGCTGTGGCCACTCAGAGAACTTCTTCTTCATCGAGGTGGGGCGGTCAGCTGTGACCGGGCCCGGCGAGTTCTGGATGCAAGTGGATGACTCCGTGGTGGCCCAGAACATGCATGAGACCATTCTAGAGGCCATGCGGGCCATGAGCGATGAGTTTCGCCCGCGCAGCAAAAGCCAATCTTCATCCAGTTGCTCCAACCCCATCAGTGTTCCCCTGCGCAGGCACCATCTCAACAATCCTCCGCCCAGCCAAGTGGGGCTGACTCGGAGATCTCGAACTGAGAGCATCACTGCCACCTCCCCTGCCAGTATGGTGGGTGGGAAACCAGGTTCCTTCAGGGTGCGTGCCTCCAGCGATGGCGAAGGCACCATGTCCCGTCCAGCATCAGTGGATGGCAGTCCTGTGAGCCCTAGCACCAACAGGACCCACGCCCATCGGCATCGAGGCAGCTCCAGGTTGCACCCCCCACTCAACCACAGCCGCTCCATCCCTATGCCTTCTTCACGATGCTCCCCTTCAGCCACCAGCCCAGTGAGCCTGTCATCCAGTAGTACCAGTGGCCACGGCTCCACTTCAGACTGTCTCTTCCCGAGGCGCTCTAGTGCTTCCGTGTCCGGTTCTCCTAGCGATGGCGGTTTCATCTCTTCTGATGAGTATGGCTCTAGTCCCTGCGATTTCCGAAGTTCCTTCCGCAGTGTCACCCCAGATTCCCTGGGCCACACCCCACCAGCCAGGGGTGAGGAGGAGCTGAGCAACTATATCTGCATGGGTGGCAAGGGAGCCTCCACCTTGACAGCTCCCAATGGTCACTACATTTTGTCTAGGGGTGGCAACGGCCATCGCTACATCCCAGGTGCTACCATGGGGACAAGCCCGGCGCTGACTGGAGACGAAGCCGCTGGTGCAGCAGATCTGGATAACCGGTTTCGGAAGAGAACTCACTCGGCTGGCACGTCCCCCACCATATCCCACCAGAAGACCCCCTCGCAGTCCTCAGTGGTTTCTATTGAGGAATATACAGAGATGATGCCCGCTGCCTACCCACCAGGAGGTGGCAGTGGAGGCCGACTGCCCGGCTACCGGCATTCCGCCTTCGTGCCCACCCACTCCTATCCCGAGGAGGGTCTAGAGATGCACCACTTGGAACGTCGTGGGGGCCACCACCGTCCAGACTCCTCCAACCTCCACACCGATGATGGCTACATGCCCATGTCTCCCGGAGTGGCTCCAGTGCCCAGCAACCGCAAAGGAAATGGGGACTATATGCCCATGAGCCCCAAGAGTGTATCTGCCCCCCAGCAGATCATTAACCCCATCAGGCGCCACCCACAGAGAGTGGACCCCAATGGCTACATGATGATGTCTCCCAGTGGTAGTTGCTCTCCTGACATTGGAGGTGGGTCTTGCAGCAGTAGCAGCATCAGCGCAGCCCCTTCTGGGAGCAGCTATGGGAAGCCATGGACAAACGGAGTAGGGGGGCACCATACCCATGCCCTTCCCCATGCCAAACCTCCTGTTGAGAGCGGTGGTGGTAAGCTCTTGCCTTGCACTGGTGACTACATGAACATGTCGCCAGTGGGAGATTCCAACACCAGCAGCCCCTCAGAATGCTACTATGGCCCAGAAGATCCCCAGCACAAGCCTGTCCTCTCCTACTACTCATTACCAAGGTCCTTTAAGCACACCCAGCGCCCTGGGGAGCCAGAGGAGGGTGCCAGGCACCAGCATCTTCGTCTCTCTTCAAGCTCTGGACGCCTTCGCTATACCGCAACTGCCGAAGATTCCTCCTCTTCCACCAGCAGCGACAGCCTGGGTGGGGGTTACTGTGGGGCTAGGCCAGAGTCTAGCGTCACACATCCCCACCACCATGCCTTGCAGCCCCATCTGCCTCGAAAGGTAGACACAGCTGCACAGACCAACAGCCGCCTGGCTCGACCCACAAGGCTGTCCTTGGGGGATCCCAAGGCAAGCACTTTACCCCGGGTACGAGAGCAACAGCAGCAGCAGCAACAGCAGCAGCAGTCTTCCCTGCACCCTCCCGAGCCCAAAAGCCCAGGAGAATATGTGAATATTGAATTCGGGAGTGGCCAGCCAGGCTATTTAGCTGGCCCTGCAACTTCCCGTAGCTCCCCTTCAGTTCGATGTCTACCCCAGCTCCACCCAGCTCCCAGAGAAGAGACTGGCTCGGAAGAGTACATGAACATGGACTTGGGGCCAGGCCGGAGGGCAACCTGGCAGGAGAGTGGTGGAGTTGAGTTGGGCAGAGTAGGCCCTGCACCTCCAGGGGCTGCTTCCATTTGTAGGCCAACCCGGTCGGTGCCAAATAGCCGTGGTGATTACATGACCATGCAGATAGGTTGTCCTCGTCAAAGCTATGTGGATACCTCACCAGTGGCCCCAGTCAGCTATGCTGACATGCGGACAGGCATTGCTGCAGAGAAGGTGAGCCTGCCCAGAACCACAGGAGCTGCCCCCCCTCCATCCTCCACAGCCTCTGCTTCTGCTTCTGTTACACCTCAAGGGGCCGCTGAGCAGGCCGCTCACTCTTCCTTGCTGGGAGGCCCTCAGGGACCTGGGGGCATGAGCGCATTCACCAGGGTGAACCTAAGTCCCAACCATAACCAGAGTGCCAAAGTGATTCGTGCAGACACTCAAGGCTGCCGGAGGAGGCACAGCTCCGAGACCTTCTCGGCGCCTACGCGGGCTGGCAACACAGTGTCTTTTGGAGCAGGGGCTGCAGGAGGGGGCAGCGGTGGTGGCAGTGAGGATGTGAAACGCCACAGCTCTGCATCCTTTGAGAATGTGTGGCTGAGACCCGGGGATCTAGGGGGAGCATCCAAGGAGTCGGCTCCAGGGTACGGGGCTGCCGGGGGATTGGAGAAGAGTCTTAACTATATAGACTTGGATTTGGTCAAGGATGTTAAGCAGCACCCTCAAGACTGCCCCTCTCAACAGCAGTCCCTGCCACCCCCTCCCCCTCACCAACCCTTAGGCAGCAATGAGGGCAGCTCCCCAAGACGCTCCAGTGAGGATTTAAGCACCTATGCCAGCATCAACTTCCAGAAGCAACCAGAGGACCGTCAATAG

>Mes_au1 [Golden hamster (Mesocricetus auratus) Irs1]

ATGGCGAGCCCTCCGGATACCGACGGCTTCTCGGACGTGCGCAAGGTGGGCTACCTGCGCAAACCCAAGAGTATGCACAAGCGATTTTTCGTACTGCGGGCAGCCAGCGAGGCCGGGGGCCCGGCGCGCCTGGAATACTATGAGAACGAGAAGAAGTGGCGGCACAAGTCGAGCGCCCCCAAACGCTCGATCCCCCTCGAGAGCTGTTTCAACATCAACAAGCGGGCTGACTCCAAGAACAAGCACCTGGTGGCTCTCTACACCCGAGACGAACACTTTGCCATTGCGGCGGATAACGAGGCTGAGCAAGACAGCTGGTACCAGGCTCTTCTGCAGCTGCATAATCGAGCAAAGGCCCACCATGACGGCGCCGGAGGTGGAGGCTGCGGTGGTAGTTGCAGCGGCAGCTCTGGCGTCGGAGAGGCAGGGGAGGACTTGAGCTATGACACGGGCCCAGGACCCGCGTTCAAGGAGGTCTGGCAGGTTATCCTGAAACCCAAAGGTCTGGGTCAGACAAAGAACCTGATTGGCATCTACCGCCTCTGCCTGACCAGCAAGACCATCAGCTTTGTGAAGCTGAACTCCGAGGCAGCGGCTGTGGTGCTACAGCTAATGAACATCAGACGCTGTGGCCACTCAGAGAACTTCTTCTTCATCGAGGTGGGCCGTTCTGCCGTGACAGGGCCCGGCGAGTTCTGGATGCAAGTGGATGACTCCGTGGTAGCCCAGAACATGCATGAGACCATTCTAGAGGCCATGCGGGCCATGAGCGATGAGTTCCGCCCACGCAGCAAAAGCCAGTCTTCATCCAGTTGCTCCAACCCCATCAGTGTTCCCCTTCGCAGGCACCATCTCAACAATCCTCCACCCAGCCAGGTGGGGCTGACTCGCCGATCTCGCACCGAGAGCATCACTGCCACTTCCCCAGCCAGCATGGTGGGAGGGAAACCAGGTTCCTTCCGAGTGCGCGCCTCCAGCGATGGCGAAGGCACCATGTCCCGTCCAGCATCAGTGGATGGTAGTCCTGTGAGCCCTAGCACCAACAGGACCCATGCCCATCGGCATCGAGGGAGCTCCAGGCTGCATCCGCCCCTCAACCACAGCCGTTCCATCCCTATGCCTTCCTCCCGCTGCTCACCTTCAGCCACCAGCCCAGTGAGTCTGTCATCCAGTAGCACGAGTGGCCATGGCTCCACCTCAGACTGTCTCTTCCCGAGACGATCTAGTGCTTCCGTGTCTGGTTCTCCTAGTGATGGCGGTTTCATCTCTTCTGATGAGTACGGTTCTAGTCCCTGCGATTTCCGAAGTTCCTTCCGCAGTGTCACCCCAGATTCCTTGGGTCACACTCCACCAGCCAGGGGTGAGGAAGAGCTGAGCAACTATATCTGCATGGGTGGCAAGGGAGCCTCCACCTTGACTGCTCCCAATGGTCACTACATTTTGTCTAGGGGTGGCAATGGTCATCGCTACATCCCAGGTGCTAACTTGGGGACAAGTCCAGCGTTGACTGGGGATGAAGCCACCAGCGCAGCGGATCTGGATAACCGGTTTCGGAAGAGAACTCACTCGGCAGGCACGTCCCCTACAATTTCCCACCAGAAGACCCCCTCGCAGTCTTCAGTGGCTTCTATTGAGGAGTATACAGAGATGATGCCTGCTGCCTACCCACCAGGAGGGGGCAGTGGAGGCCGACTGCCCAGCTATCGGCATTCCGCCTTCGTGCCCACCCACTCCTATCCCGAAGAGGGTCTAGAGATGCACCCCTTGGAGCGTCGTGGGGGCCACCACCGTCCAGACACCTCCTCCTTGCACACTGATGATGGCTACATGCCCATGTCTCCCGGGGTGGCGCCAGTGCCAAGCAACCGTAAAGGAAATGGGGACTACATGCCCATGAGCCCCAAGAGTGTTTCTGCCCCACAGCAGATCATTAACCCCATCAGACGTCACCCTCAGAGAGTGGACCCCAATGGCTACATGATGATGTCTCCCAGTGGCAGTTGCTCCCCTGACATTGGAGGTGGGTCCAGCAGCAGCAGTGGTGCAGCCGCTTCTGGGAGCAGCTACGGGAAGCCATGGACAAATGGAGTAGGGGGGCACCATAGTCATGCCCTGCCTCATTCCAAACCCCCTATTGAGAGTGGCGGTGGCAAGCTCTTGCCTTGCACAGGTGACTACATGAACATGTCACCAGTGGGAGACTCCAACACCAGCAGCCCCTCAGAATGCTACTACGGCCCAGAAGATCCCCAGCACAAGCCGGTTCTCTCCTACTACTCATTGCCAAGGTCTTTTAAGCACACCCAGCGCCCTGGGGAGCCAGAGGAGGGTGCCAGGCACCAGCATCTTCGTCTCTCTTCTAGCTCTGGACGCCTTCTCTACACTGCAACTGCAGAAGATTCTTCCTCTTCTACCAGCAGTGACAGCTTGGGTGGGGGTTACTGTGGGGCTAGGCCAGAGGCTGGCCTCCCGCATCCCCACCACCATGCCTTGCAACCCCATGGGCCTCGAAAGGTAGACACGGCTGCGCAGACCAACCGCCGTCTGGCCCGGCCCACAAGGCTCTCCTTGGGAGATCCCAAGGCAAGCACCTTACCCCGGGTACGGGAGCAGCAGCAGCAGCAGCAACCTTCGCTGCACCCTCCGGAGCCCAAGAGCCCGGGGGAATATGTGAATATTGAATTCGGGAGTGGCCAGCATGGCTATTTAGCTGGCCCTGCAACTTCCCATAGCTCTCCTTCAGTTCGGTGTCCACCCCAGCTCCACCCAGCTCCCCGAGAAGAGACTGGCTCGGAAGAGTACATGAACATGGATTTGGGGCCAGGCAGAAGGGCAACCTGGCAGGAGAGTGGTGGGGCTGAGTTGGGTAGAGTAGGCCCTGCACCTCCAGGGGCTGCTACCATTGGCAGGCCAACCCGGGCAGTGCCAAGTAGCCGTGGTGACTACATGAGCATGCAGATAGGTTGTCCTCGTCAAAGCTATGTGGATACCTCACCAGTGGCCCCAGTCAGCTATGCTGACATGCGGACAGGCATTGCGGCCGAGAAGGTGAGCCTGCCCAGAACCACAGGGGCTGCTCCATCTGCATCCTCCACAGCCTCTGATTCTCCTGCTGCACCTCAAGGAGCAGCTGAGCAGGCTGCTCACTCTTCTCTGCTGGGAGGCCCTCAGGGACCTGGGGGCATGAGTGCATTCACCAGAGTGAACCTCAGTCCCAACCATAACCAGAGTGCCAAAGTGATTCGTGCAGACACTCAAGGCTGCCGGAGGAGGCACAGCTCCGAGACCTTCACAGCACCTACTCCAGCTGGCAACACGGTGTCCTTTGGAGCAGGGGCTGCAGTAGGGGGCAGTGGCGGGGGCAGCAGCAGCAGCAGTGAGGATGTAAAACGCCACAGCTCTGCATCATTTGAGAATGTGTGGCTGAGACCTGGGGATCTAGGGGGAGCCTCCAAGGAGACAGCTCAAGTGTGTGGGGCTGCTGGGGGTTTGGAGAGTAGTCTTAACTACATAGACCTGGATTTGGCCAAGGATGTTAGACAGCGTCCTCAGGAATGCCCTTCTCAACAGCAGCCTCTTCCACCCCCTGCCCCTCACCAGCCTCCAGGCAGCAACGAGGGCAGCTCCCCCAGACGCTCCAGTGAGGATTTAAGCACCTATGCCAGCATCAGCTTCCAGAAGCCGCCAGAGGACCATCAATAG

>Cri_gr1 [Chinese hamster (Cricetulus griseus) Irs1]

ATGGCGAGCCCTCCGGATACCGATGGCTTCTCGGACGTGCGCAAGGTGGGCTACCTGCGCAAACCCAAGAGTATGCACAAGCGCTTTTTCGTACTGCGGGCAGCCAGCGAGGCCGGGGGCCCGGCGCGCCTGGAATACTATGAGAACGAGAAGAAGTGGCGGCACAAGTCGAGCGCCCCCAAACGCTCGATCCCCTTGGAGAGCTGTTTCAACATCAACAAGCGGGCTGACTCCAAGAACAAGCACCTGGTGGCTCTCTACACCCGAGACGAACACTTTGCCATTGCGGCAGATAGCGAGGCTGAGCAAGACAGCTGGTACCAGGCTCTTCTGCAGCTGCATAACCGAGCAAAGGCCCACCATGACGGCGCTGGAGGTGGAGGCTGCGGCGGTAGTTGCAGCGGCAGCTCTGGCGTCGGAGAAGCAGGGGAGGACTTGAGCTATGACACGGGCCCAGGACCTGCGTTCAAGGAGGTCTGGCAGGTTATCCTGAAACCCAAAGGTCTGGGTCAGACAAAGAACTTAATTGGCATCTACCGCCTCTGCCTGACCAGCAAGACCATCAGCTTTGTGAAGCTCAACTCCGAGGCAGCGGCTGTGGTGCTACAGCTAATGAACATCAGACGCTGTGGCCACTCAGAGAACTTCTTCTTCATCGAGGTGGGGCGTTCTGCCGTGACAGGACCCGGCGAGTTCTGGATGCAAGTGGATGACTCCGTGGTAGCCCAGAACATGCATGAGACCATTCTAGAGGCCATGCGGGCCATGAGCGATGAGTTTCGCCCACGCAGCAAAAGCCAGTCTTCATCCAGTTGCTCCAACCCCATCAGTGTTCCCCTTCGCAGGCACCACCTCAACAACCCTCCACCCAGCCAGGTGGGGCTGACTCGCCGATCTCGCACTGAGAGCATCACCGCCACCTCCCCTGCCAGCATGGTGGGAGGGAAACCAGGTTCCTTCCGAGTGCGAGCCTCCAGCGATGGCGAAGGCACCATGTCCCGTCCAGCATCAGTGGATGGTAGTCCTGTGAGCCCGAGCACCAACAGGACCCACGCCCATCGGCACCGAGGGAGCTCCAGGCTTCACCCCCCACTCAACCACAGCCGCTCCATCCCTATGCCTTCTTCCCGCTGCTCACCTTCAGCCACCAGCCCAGTAAGTCTGTCATCCAGTAGCACTAGTGGCCATGGCTCCACCTCAGACTGTCTCTTCCCGAGACGATCTAGTGCTTCCGTGTCAGGTTCTCCTAGTGATGGCGGTTTCATCTCTTCTGATGAGTATGGTTCTAGTCCCTGCGATTTCCGCAGTTCCTTCCGCAGTGTCACCCCAGATTCCCTGGGTCACACCCCACCAGCCAGGGGTGAGGAAGAACTGAGCAACTATATCTGCATGGGTGGCAAGGGAGCCTCCACCCTGACTGCTCCCAATGGTCACTACATTTTGTCTAGGGGTGGCAATGGCCATCGCTACATCCCAGGTGCTAACTTGGGGACAAGCCCAGCGTTGACTGGGGATGAAGCCACCAGTGCAGCAGATCTGGACAACCGGTTTCGGAAGAGAACTCACTCCGCAGGCACGTCCCCTACCATTTCCCATCAGAAGACCCCCTCGCAGTCTTCAGTGGCTTCTATTGAGGAGTATACAGAGATGATGCCTGCTGCCTACCCACCAGGAGGTGGCAGTGGAGGCCGACTGCCCAGCTATCGGCATTCCGCCTTCGTGCCCACCCACTCCTACCCCGAAGAGGGTCTCGAGATGCACCCCTTGGAGCGTCGTGGGGGCCACCACCGTCCAGACACCTCCACCCTGCACACTGATGATGGCTACATGCCCATGTCTCCAGGGGTGGCGCCAGTGCCAAGCAACCGTAAAGGGAATGGGGACTATATGCCCATGAGCCCCAAGAGTGTCTCTGCCCCACAACAGATCATTAACCCCATCAGACGTCACCCTCAGAGAGTGGACCCCAATGGCTACATGATGATGTCTCCCAGTGGCAGTTGCTCCCCTGACATTGGAGGTGGGTCCAGCAGCAGCAGCGGTGCAGCCGCTTCGGGGAGTAGCTACGGGAAGCCATGGACAAATGGAGTAGGGGGGCACCATAGTCATGCCCTGCCTCATTCCAAACCCCCTACTGAGAGTGGCAGTGGCAAGCTCTTGCCCTGCACAGGCGACTACATGAACATGTCACCAGTGGGAGACTCCAACACCAGCAGCCCCTCAGAATGCTACTATGGCCCAGAAGATCCCCAGCACAAGCCAGTCCTCTCCTACTACTCATTGCCAAGGTCTTTCAAGCACACCCAGCGCCCTGGGGAGCCAGAGGAGGGTGCCAGGCACCAGCATCTTCGTCTCTCTTCTAGCTCTGGACGCCTTCTCTATGCTGCAACTGCAGAAGATTCTTCCTCTTCTACCAGCAGTGACAGCCTGGGTGGGGCTTACTGTGGGGCTAGGCCAGAGTCCGGCCTCCCACATCCCCACCACCATGTCGTGCAACCCCATGGGCCCCGAAAGGTAGACACGGCTGCACAGACCAACCGCCGCCTGCCCCGGCCCACAAGGCTCTCCTTGGGAGATCCCAAGGCAAGTACCTTTCCCAGGGTGTGGGAGCAGCAGCAGCCTTCGTTGCACCCTCCCGAGCCCAAGAGCCCAGGGGAATATGTGAATATTGAATTCGGGAGTGGCCAGCCTGGCTATTTAGCGGGCCCGGCGACTTCCCACAGCTCTCCTTCGGTTCGGTGTCCACCCCAGCTCCACCCAGCTCCCCGAGAAGAGACTGGCTCAGAAGAGTACATGAACATGGACTTGGGGCCAGGCCGGAGGGCAACGTGGCAGGAGAGTGGTGGGGTTGAGTTGGGTAGAGTAGGCCCTGCACCTCCAGGGGCTGCTACCATTTGCAGGCCAACCCGGTCAGTGCCAGGTAGCCGTGGTGACTACATGACCATGCAGATAGGTTGTCCTCGTCAAAGCTATGTGGATACCTCACCAGTGGCCCCAGTCAGCTATGCTGACATGCGGACAAGCATTGGCGCCGAGAAGGTGAGCCTGCCCAGAACCACAGGGGCTGCTCCATCTTCATCCTCCACAGCCTCTGCTTCTCCTGCTGCACCTGAAGGCGCAGCTGAGCAGGCTGCTCACTCTTCTCTGCTGGGAGGCCCTCAGGGACCTGGGGGCATGAGTGCATTCACCAGAGTGAACCTCAGTCCCAACCATAACCAGAGTGCCAAAGTGATTCGTGCAGACACTCAAGGCTGCCGGAGGAGGCACAGCTCCGAGACCTTCTCAGCACCTACTCGGGCTGGCAACACGGTGTCCTTTGGAGCAGGGGCTGCAGTAGGGGGCAGTGGCGGTGGAGCTGGCGGTGGCAGCAGCAGCAGTGAGGATGTAAAACGCCACAGCTCTGCATCATTTGAGAATGTGTGGCTGAGACCTGGGGATCTAGGGGGAGCCTCCAAGGAGACGGCTCAAGTGTGTGGGGCTGCTGGGGGTTTGGAGAGTAATCTTAACTACATAGACCTGGATTTAGCCAAGGATGTTAGACAGCGTCCTCAGGAATGCCCTTCTCAACAGCAGCCCCTTCCACCCCCTGCCCCTCACCAGCCCTCACGCAACAACGAGGGCAGCTCCCCCAGACGCTCCAGTGAGGATTTAAGCACCTATGCCAGCATCAACTTCCAGAAGCAGCCAGAGGACCATCAATAG

>Mic_oc1 [Prarie vole (Microtus ochrogaster) Irs1]

ATGGCGAGCCCTCCGGATACCGAAGGCTTCTCGGACGTGCGCAAGGTGGGTTACCTGCGCAAACCCAAGAGTATGCATAAGCGCTTTTTCGTGCTGCGAGCAGCCAGCGAGACAGGGGGCCCGGCACGCCTGGAGTACTATGAGAACGAGAAGAAGTGGAGGCATAAGTCGAGCGCCCCCAAACGCTCGATCCCCCTCGAGAGCTGTTTCAACATCAACAAGCGGGCTGACTCCAAGAACAAGCACCTGGTGGCTCTCTACACCCGAGACGAACACTTTGCCATTGCGGCAGATAGCGAGGCTGAGCAAGACAGCTGGTACCAGGCGCTTCTGCAGCTGCATAATCGAGCAAAGGCCCACCATGACGGGGCTGGAGGTGGAGGCTGCGGTGGTAGTTGCAGCGGCAGCTCTGGCGTCGGAGAAGCAGGGGAGGACTTGAGCTATGACACTGGACCAGGACCCGCGTTCAAGGAGGTCTGGCAGGTTATCCTGAAACCCAAGGGCCTGGGCCAGACAAAGAACCTGATTGGCATCTACCGTCTCTGCCTGACCAGCAAGACCATCAGCTTTGTGAAGCTGAACTCCGAGGCAGCGGCTGTGGTGCTGCAGCTAATGAACATCAGACGCTGTGGCCACTCAGAGAACTTCTTCTTCATTGAGGTGGGGCGTTCCGCCGTGACAGGGCCCGGTGAGTTCTGGATGCAAGTGGATGACTCCGTGGTAGCCCAGAATATGCACGAGACCATTCTAGAGGCCATGCGGGCCATGAGCGACGAGTTTCGCCCGCGCAGCAAAAGCCAGTCCTCATCCAGTTGCTCCAACCCCATCAGTGTTCCCCTTCGCAGGCACCATCTCAACAACCCTCCACCCAGCCAGGTGGGCCTGACTCGCCGATCTCGCACCGAGAGCATCACTGCAACCTCCCCTGCCAGCATGGTGGGTGGGAAACCAGGTTCCTTCCGGGTGCGCGCCTCTAGTGATGGCGAAGGCACCATGTCCCGTCCAGCATCAGTGGATGGCAGTCCTGTGAGCCCTAGCACCAACAGGACCCACGCCCATCGGCATCGAGGCAGCTCCAGGCTTCATCCTCCACTCAACCACAGCCGCTCCATCCCTATGCCTTCTTCCCGCTGCTCGCCTTCAGCCACCAGCCCAGTCAGTTTGTCATCCAGTAGCACTAGTGGCCATGGCTCCACCTCAGACTGTCTCTTCCCGAGGCGCTCCAGTGCTTCGGTGTCCGGTTCTCCTAGTGATGGCGGTTTCATCTCTTCTGATGAGTATGGTTCTAGTCCGTGCGATTTCCGAAGTTCCTTCCGCAGCGTCACCCCAGATTCCCTGGGTCACACCCCACCAGCCAGGGGTGAGGAAGAGCTGAGCAACTACATCTGCATGGGTGGCAAGGGAGCCTCCACCTTGACTGCTCCCAATGGACATTACATTTTGTCTAGGGGTGGTAATGGCCATCGCTACCTCCCAGGTGCTAACTTGGGGACAAGCCCGGCATTGACTGGGGATGAAGCCACCAGCGCAGCAGATCTGGATAACCGGTTTCGGAAGAGGACTCACTCCGCAGGCACGTCCCCTACCATCTCCCATCAGAAGACCCCCTCACAGTCTTCTGTGGCTTCTATTGAGGAGTATACGGAGATGATGCCCGCTGCCTACCCACCAGGAGGTGGCAGTGGAGGCCGACTGCCCAGCTACCGGCATTCCGCCTTCGTGCCCACCCACTCCTATCCCGAAGAGGGTCTGGAGATGCATCCCTTGGAGCGTCGTGGGGGCCACCACCGACCGGACACTTCTACCCTGCACACCGATGACGGCTACATGCCCATGTCTCCAGGGGTGGCTCCAGTGCCCAGCAACCGTAAAGGAAATGGGGACTATATGCCCATGAGCCCCAAAAGTGTATCCGCCCCACAGCAGATCATTAACCCCATCCGACGCCACCCTCAGAGAGTGGACCCCAATGGCTACATGATGATGTCTCCCAGTGGCAGTTGCTCCCCTGACATTGGTGGTGGTGGCTCCAGCAGCAGCAGCAGTGCCGCCCCGTCTGGGAGCAGCTATGGGAAGCCGTGGACAAACGGAGTAGGGGGACACCATCCTCATGCCCTGCCTCATTCCAAAGCCCCTGCTGAGAGTGGTGGCGGTAAGCTCTTGCCTTGCACAGGCGACTATATGAACATGTCACCCGTGGGAGACTCCAACACCAGCAGCCCCTCGGAATGCTACTACGGTCCCGATAATCCCCAGCACAAACCAGTCCTCTCCTACTACTCATTGCCAAGGTCTTTTAAGCACACCCAGCGCCCTGGGGAGCCAGAGGAGGGTGCCAGGCACCAGCATCTTCGTCTCTCTTCTAGCTCTGGACGCCTTCTCTACACCGCAACTGCGGAAGATTCTTCCTCTTCTACCAGCAGTGACAGCTTAGGTGGGAGTTACTGTGGGGCTAGGCCAGAGTCTGGCCTCACGCATCCCCACCACCATGTCTTGCAACCCCATCTGCCTCGAAAGGTAGACACAGCTGCACAGACCAACAACCGCCTGGCCCGGCCTACAAGGCTGTCCTTGGGAGATCCCAAGGCAAGCACCTTACCGCGGGTTCGGGAGCAGCCGCCGCAGCAGCCTTCGCTACACCCTCCTGAGCCCAAGAGCCCAGGGGAATATGTGAATATTGAATTCGGGAGTGGCCAGCCTGGCTATTTAGCTGGCCCTACGACTTCGAATAGCTCTCCTTCAGTTCGGTGTCCACCCCAGCTCCACCCAGCTCCCAGAGAAGAGACTGGCTCAGAAGAGTACATGAACATGGACTTGGGGCCAGGCCGGAGGGCAACCTGGCAGGAGAGTGGTGGCATTGAGTTGGGCAGAGTAGGCCCTGCACCTCCAGGGGCTGCTAACATTTGCAGGCCAACCCGGTCAGTGCCAAGTAGTCGTGGTGACTACATGACCATGCAGATAGGTTGTCCTCGTCAAAGCTATGTGGATACCTCACCAGTGGCCCCAGTCAGCTATGCTGATATGCGGACAGGCCTTGCTGCGGAGAAGGTGAGCCTGCCCAGAGCCGCAGGGGCTGCTCCATCTTCGTCCTCTACAGCTTCTGCTTCTCCTGCTGCACCTCAAGGAGCAGCTGAGCAGGCTGCTCACTCTTCCCTGCTGGGAGGCCCTCAGGGACCTGGGAGCGTGAGTGCATTCACCAGAGTGAATCTCAGTCCCAATCATAACCAGAGTGCCAAAGTGATTCGTGCAGACACTCAAGGCTGCCGGAGGAGACATAGCTCTGAGACCTTCTCGGCACCAACTCGGGCTGGTCACACAGTGTCCTTTGGAGCAGGGGCTGCAGGAGCGGGCAGTGGTGGTGGTGGCGGCAGCAGCGAGGATGTAAAACGCCACAGCTCTGCATCTTTTGAGAATGTGTGGCCGAGACCTGGGGATCTAGGGGGAGCCTCCAAGGAAACAGCTCAAGCGCGTGGAGCTGCTGGGGGTTTGGAGAATACTCTTAACTACATAGACCTGGATTTGGCCAAGGACGTGAAACAGCGTCCTCAGGAGTGCCCTTCTCAACAGCAGCCCCTACCACCCCCTCCCTCTCACCAGCCCTCAGGCAGTAATGAGGGCAGTTCCCCCAGACGCTCCAGTGAGGATTTAAGCACCTATGCCAGCATCAGCTTCCAGAAGCAGCCAGAGGACCGTCAGTAG

>Per_ma1 [Prairie deer mouse (Peromyscus maniculatus bairdii) Irs1]

ATGGCGAGCCCTCCGGATACCGATGGCTTCTCCGACGTGCGCAAGGTGGGCTACCTGCGCAAACCCAAGAGTATGCACAAGCGCTTTTTCGTGCTGCGGGCAGCCAGCGAGGCCGGGGGCCCGGCGCGCCTGGAGTACTATGAGAACGAGAAGAAGTGGCGGCACAAGTCGAGCGCCCCCAAACGCTCGATCCCCCTCGAGAGCTGTTTCAACATCAACAAGCGGGCTGACTCCAAGAACAAGCACCTGGTGGCTCTCTACACCCGAGACGAACACTTTGCCATTGCAGCAGATAGCGAGGCTGAGCAAGACAGCTGGTACCAGGCTCTTCTGCAGCTGCATAATCGAGCAAAGGCCCATCATGACGGGGCTGGAGGCTGCGGCGGTAGTTGCAGCGGCAGCTCCGGCGTCGGAGAGGCAGGGGAGGACTTGAGCTATGACACGGGCCCAGGACCTGCGTTCAAGGAGGTCTGGCAGGTTATCCTGAAACCCAAAGGCCTGGGTCAGACAAAGAACCTGATTGGCATCTACCGCCTCTGCCTGACCAGCAAGACCATCAGCTTTGTGAAGCTGAACTCCGAGGCAGCGGCTGTGGTGCTGCAGCTGATGAACATCAGACGCTGTGGACACTCAGAGAACTTCTTCTTCATCGAGGTGGGGCGTTCCGCCGTAACAGGGCCCGGCGAGTTCTGGATGCAAGTAGATGACTCCGTGGTAGCCCAGAACATGCATGAGACCATTCTAGAGGCCATGCGGGCCATGAGCGATGAGTTTCGCCCGCGCAGCAAAAGCCAGTCTTCATCCGGTTGCTCCAACCCCATCAGTGTCCCCCTTCGCAGGCACCATCTCAACAATCCTCCACCCAGCCAGGTGGGGCTGACTCGCCGATCTCGCACCGAGAGCATCACTGCCACCTCCCCTGCCAGCATGGTAGGTGGAAAACCAGGTTCCTTCCGGGTGCGTGCCTCCAGTGATGGCGAGGGCACCATGTCCCGTCCTGCATCAGTGGACGGCAGTCCGGTGAGCCCCAGCACCAACAGGACCCACGCCCATCGGCATCGAGGCAGCTCCAGGCTGCACCCCCCACTCAACCACAGCCGCTCCATCCCTATGCCTTCTTCCCGCTGCTCACCTTCAGCCACCAGCCCAGTAAGTCTGTCATCTAGTAGCACTAGTGGCCATGGTTCCACCTCAGACTGTCTCTTTCCGAGGCGCTCTAGTGCTTCTGTGTCCGGCTCTCCTAGTGATGGCGGTTTCATCTCTTCTGATGAGTACGGTTCTAGTCCCTGCGACTTCCGAAGTTCCTTCCGCAGTGTCACCCCAGATTCCTTGGGCCACACCCCACCAGCCAGGGGTGAGGAAGAGCTGAGCAACTATATCTGCATGGGTGGCAAGGGAGCCTCCACTTTGGCTGCTCCCAATGGTCACTACGTTTTGTCTAGGGGTGGCAATGGCCACCGCTACGTCCCAGGTGCTAGCTTGGGGACAAGCCCAGCGCTGACCGGGGATGAAGCCGCCAGCGCAGCGGATCTGGATAACAGGTTTCGGAAGAGAACTCACTCCGCGGGCACGTCCCCTACCATCTCTCACCAGAAGACCCCCTCGCAGTCTTCGGTGGCTTCTATTGAGGAATATACGGAGATGATGCCCGCTGCCTACCAACCAGGAGGTGGCAGTGGAGGCCGACTGCCCAGCTACCGGCATTCCGCCTTCGTGCCCACCCACTCCTATCCCGAAGAGAGTCTAGAGATGCACCCCTTGGAGCGTCGTGGGGGCCACCACCGTCCAGACACTGATGATGGCTACATGCCCATGTCTCCAGGGGTGGCTCCAGTGCCCAGCAACCGTAAAGGAAATGGGGACTATATGCCCATGAGCCCCAAAAGTGTGTCTGCCCCACAGCAGATCATTAACCCCATCAGACGCCACCCTCAGAGAGTGGACCCCAATGGCTACATGATGATGTCTCCCAGTGGCAGTTGCTCCCCTGACATTGGCGGTGGGTCCAGCAGCAGCAGCAGCGGCGCAGCCCCTTCTGGGAGCAGCTATGGGAAGCCGTGGGCAAATGGAGTAGGGGGGCACCACCCTCATGCCCTGCCTCATTCCAAACCTCCTGCCGAGAGTGGTGGTGGCAAGCTCTTGCCTTGCACAAGCGACTACATGAACATGTCACCAGTGGGAGACTCCAACACCAGCAGCCCCTCAGAATGCTACTACGGTCCAGAAGATCCCCAGCACAAGCCAGTCCTTTCCTACTACTCATTGCCAAGGTCTTTTAAGCACACCCAGCGCCCCGGGGAGCCGGAGGAGGGTGCCAGGCACCACCAGCATCTTCGTCTCTCTTCCAGCTCTGGACGCCTTCTCTACACTACAACTGCGGAAGATTCTTCCTCTTCGACCAGCAGTGACAGCCTGGGTGGGGGTTACTGTGGGGCTAGGCCAGAGTCTGGCCTCCCTCATCCCCACCACCATGTCTTGCAGCCCCATCTGCCTCGAAAGGTAGACACGGCTGCGCAGACCAACAGCCGCCTGGCCCGGCCCACAAGGCTGTCCTTGGGGGATCCCAAGGCAAGCACCTTACCGAGGGTAAGGGAGCAGCAGCAGCAGCAGCAGCAGCAGCAACAGCAGCAACAGCAGCAATCTTCTCTGCACCCTCCGGAGCCCAAGAGCCCAGGGGAATATGTGAATATTGAATTCGGGAGTGGCCAGCCTGGCTATTTAGCTGGCTCCGCGACTTCCCATAGCTCCCCTTCAGTCCGATGTCCACCCCAGCTCCACCCAGCTCCCAGAGAAGAGACTGGCTCTGAAGAGTACATGAACATGGACTTGGGGCCAGGCCGGAGGGCAACCTGGCAGGAGAGTGGTGGGGTTGAGTTGGGCAGAGTAGGCCCTGCACCTCCAGGGGCTGCTACCATTTGCAGGCCAACCCGGTCAGTGCCAAGTAGTCGCAGTGACTACATGACCATGCAGATAGGTTGTCCTCGTCAAAGCTATGTGGATACCTCCTCAGTGGCCCCAGTCAACTATGCTGACATGCGGACAGGCATTGCTGCAGAGAAGGTGAGCCTGCCCAGAACCACAGGGGCCGCTCCCTCTTCATCTTCCACAGCCTCTGCTTCTCCTGCTGCACCTCAAGGAGCAGCAGCTGAGCAGGCTGCTCCATCTTCCCTGCTGGGAGGCCCTCAGGGACCTGGGGGCATGAGTGCATTCACCAGAGTGAACCTCAGCCCCAACCATAACCAGAGTGCCAAAGTGATTCGTGCAGACACTCAAGGGTGCCGGAGGAGACATAGCTCTGAGACTTTCTCAGCACCTACTCATGCTGGCAACACGGTGTCCTTGGGAGCAGGGGCTGCAGTAGGGGGCAGTGGTGGCGGCGGCGGTGGCGGCAGCAGCAGCAGCAGTGAGGATGTAAAGCGCCACAGCTCGGCATCATTTGAGAACGTGTGGCTGAGACCTGGGGATCTAGCGGGAGCCTCCAAGGAGACGGCTCCAGTGTCCGGGGCTGCTGGGGGTTTGGAGAAAAGTCTTAACTACATAGACCTGGATTTGGCCAAGGACGTTAGACAGCGCCCTCCCCGGGAGTGCCCTTCTCAACAGCAGCCCCTACCGCCCCCTCCCCCTCACCAGCCCTCAGGCAGCAGAGAGGGCAGCTCCCCCAGACGCTCCAGTGAGGATTTAAGTACTTATGCCAGCATCAGCTTCCAGAAACCGCCAGAGGACCGTCAATAG

>Dip_or1 [Kangaroo rat (Dipodomys ordii) Irs1]

ATGGCGAGCCCTCCGGAGGCCGATGGCTTCTCGGACGTGCGCAAGGTGGGGTACCTGCGCAAACCCAAGAGCATGCACAAGCGTTTCTTCGTACTGCGGGCGGCCAGCGAGGCCGGGGGCCCGGCGCGCCTGGAGTACTACGAGAACGAGAAGAAGTGGCGGCACAAGTCGAGCGCCCCCAAACGCTCGATCCCCCTCGAGAGCTGCTTTAACATCAACAAGCGGGCCGACTCCAAAAACAAGCACCTGGTGGCTCTCTACACCCGAGACGAGCACTTTGCCATTGCGGCAGATAGCGAGGCCGAGCAAGACAGCTGGTATCAGGCGCTCCTGCTGTTGCATACCCGGGCCAAGGGTCACCACCACGACGGGAGCGCGGGCTCCGGGGCCGGGGGCAGCTGCAGCGGCAGCTCAGGCGTGGGGGAAGCCGGGGAGGATCTGAGCTTCGGAGAGACGCCCCCGGGACCCGCCTTCAAGGAGGTCTGGCAGGTGATCCTGAAACCCAAGGGCCTGGGGCAGACAAAGAACCTGATCGGCATCTACCGCCTCTGCCTGACCAGCAAGACCATCAGCTTCGTGAAGCTCAACTCGGAGGCCGCCGCCGTGGTGCTCCAGCTGATGAACATCCGCCGCTGCGGCCACTCGGAGAACTTCTTCTTCATCGAGGTGGGCCGTTCGGCCGTGACAGGGCCCGGCGAGTTCTGGATGCAGGTGGATGACTCGGTGGTGGCCCAGAACATGCACGAGACCATCCTGGAGGCCATGCGGGCCATGAGCGACGAGTTCCGGCCCCGCAGCAAGAGCCAGTCGTCCTCCAACTGCTCCAACCCCATCAGCGTCCCCCTGCGCCGACACCACCTCAACAATCCCCCGCCCAGCCAGGTGGGGCTGACACGCCGCTCGCGCACCGAGAGCATCACCGCCACCTCCCCGGCCAGCATGGTGGGCGGGAAGCCGGGCTCCTTCCGCGTGCGCGCCTCCAGCGACGGAGAAGGCACCATGTCCCGCCCGGCCTCGGTGGACGGCAGCCCCGTGAGTCCCAGCATCAACCGGACCCACGCGCACCGCCATCGGGGCAGCTCCCGGCTGCACCCCCCGCTCAACCACAGCCGGTCCATCCCCATGCCCTCTCGGTGCTCCCCGTCGGCCACCAGCCCCGTGAGCCTGTCGTCCAGTAGCACCAGCGGCCACGGCTCCACCTCGGACTGTCTCTTCCCACGGCGATCCAGCGCTTCCGTGTCCGGCTCCCCGAGCGATGGCGGCTTCATCTCTTCCGACGAGTACGGCTCGAGTCCTTGCGACTTCCGCAGTTCGTTCCGCAGCGTCACCCCCGATTCCCTGGGTCACACCCCACCGGCCCGGGGCGAGGAGGAGCTGAGCAACTACATCTGCATGGGTGGCAAGGGCGTCTCCACGCTGGCCGCTCCCAACGGCCACTACGTGCTGTCGCGCGGGGGCAATGGCCACCGCTACCTGCCGGGAGCCGCGGGCCTGGGCACCAGCCCGGCCCTGGCTGTGGACGAGGCCGCCGCCGTCGGGGCTGCGGATCTGGACAATCGGTTCCGGAAGAGAACCCACTCGGCCGGCACGTCCCCCACCATTTCCCACCAGAAGACCCCCTCGCAGTCGTCCACGGAGGAGTACACGGAGATGATGCCCGCCTACCCGCCAGGAGGGGGCAGTGGAGGCCGGCTGCCCGGCTACCGCCACTCGGCCTTCGTGCCCACCCACTCGTACCCCGAGGAAGGGCTGGAGCTGCATCCCTTGGAGCGCCGTGGGAGCCACCAACCCCACCAGCACCCCCGGCCCGACCACCACGCCGCCCTCCACACGGACGACGGCTACATGCCCATGTCCCCGGGGGTGGCCCCGCTGCCCACCGGCCGCAAGGGCACCGGGGACTACATGCCCATGAGCCCCAAGAGCGTGTCTGCCCCCCAGCAGATCATCAACCCCGTCAGACGCCATCCTCAGCGCGTGGACCCCAACGGCTACATGATGATGTCCCCCAGCGGCAGCTGCTCGCCCGACATCGGTGGGGGCGGCGGCGGCGGCGGCGGCGGGGTCAGCGGGAAGCTCTGGACGAATGGGGTCGGGGGTCACCACCCCCACGCCCTGGCGCACCCCAAGCCCCCTGCCGACAGCAGCAGCGGTGGCAAACTCTTGCCTTGCACCGGGGACTACATGAATATGTCCCCCGTGGGCGACTCCAACACCAGCAGCCCCTCCGACGGCTACTACGGCCCCGAGGACAAGCCCGTTCTGTCCTACTACTCATTGCCAAGGTCCTTCAAGCACACCCAGCGCCCCGGGGAGCCGGAGGAGGGTGCCCGCCCCCAGCACCTCCGCCTCTCCTCCAGCTCCGGCCGCCTTCTCTATGCCACCACGGCAGAAGATTCTTCGTCGTCCACCAGCAGCGACAGCCTCGGCGGGGGTTACTGTGGGCCTCGGCCCGAGCCCGGCCTTCCCCATCCCCACCATCCTGTCCTGCAGCCGCATCTACCACGAAAGGTGGACACCGCCGCGCAGACCCATAGTCGCCTGGCCAGACCCACCAGGCTCTCCCTGGGGCACCCCAAGGCCAGCACCTTACCACGGGCTCGTGAACAGCCGCTGCAGCCCCAGCAGCAGCAACAGCAGCAGCAGCAGCAGCAGCAGCAGCAGCCTCCAGAGCCCAAGAGCCCAGGGGAATATGTGAATATTGAATTTGGAAGTGAGCAGCCTGGCTACTTCTCTGGCCCCAGGGCTCCACATAGCTCCGGCCTTTCTGTCCGGTGCCCATCCCAGCTTCAGCCAGCTCCCAGAGAAGAGGAGACTGGCACTGAAGAGTACATGAACATGGACCTGGGGCCAGGCCGGAGGGCAGCCTGGCAGGAGAGTGGTGTGGTTGAGGTGGCCAAGGTCGCTGGGCCTGCCCCTCCAGGGGCTGCTAGCGTGTGCAGGCCTACCCGGGCCGTGCCCAGCAGCCGCGGCGATTACATGACCATGCAGATGCGTTGTTCCAGCCAGAGCTACGTGGACACCTCCCCAGTTGCCCCCGTCAGCTATGCTGACATGAGGACCGGCCTTGCTACAGTAGAGGACGTGAGCCTGCCCCGGGCCACGGCCGCTGCTCCCTCTGCCTCCGCAGCCGTGGCCGCCTCTCCCAGTGAGCCTGAAGGAGCAGCCGAGCTGCCTGGGCGGGCTTCCCTGCTGGGGCCTCCACAGGGACCCGGGGGCATCAGCGCCTTTACCAGGGTGAATCTCAGTCCCAACCGCAGCCAGAGTGCCAAAGTGATCCGTGCAGATCCACAAGGATGCCGGAGGAGGCATAGCTCGGAGACCTTCTCCTCCACTCCCGCTGCCACCCGGGCGGGCAACACGGTGCCCTTTGCAGCGGGGGCCGCAGTGGGGGGCAGCAGCAGCAGCAGCAGCAGCGAGGAGGTCAAGCGCCACAGCTCTGCCTCCTTTGAGAATGTGTGGCTGAGACCTGGGGAGCTAGGGGGAGGCCCCAAGGAGACTGCCCAAGGGTGCGGGGCCGCCGGGGGTGTGGAGAATGGTCTTAACTACATAGACCTGGATTTGGTCAAGGACTTCAAACAGCGCCCTCCGGAGCGTCCCCCACAGCAGCAGCCTCCCGCACCCCCAAACCCCCATCAGCCCGTGGGCAGCGGTGAGAGCCGCTCCACCCGCCGCTCCAGTGAGGATTTAAGCACCTATGCCAGCATCAGCTTCCAGAAGCAGCCAGAGGACCGGCAGTAG

>Jac_ja1 [Lesser Egyptian Jerboa (Jaculus jaculus) Irs1]

ATGGCGAGCCCTCCGGATGCCGACGGCTTCTCGGACGTGCGCAAGGTGGGCTACCTGCGCAAACCCAAGAGTATGCACAAGCGCTTCTTCGTGCTGCGGGCGGCCAGCGAGGCCGGGGGCCCCGCGCGCCTGGAGTACTACGAGAACGAGAAGAAGTGGCGGCACAAGTCGAGCGCCCCCAAACGCTCGATCCCCCTCGAGAGCTGCTTCAACATCAACAAGCGGGCCGACTCCAAGAACAAGCACCTGGTGGCCCTCTACACCCGGGACGAGCACTTTGCCATCGCGGCCGACAGCGAGGCCGAGCAAGACAGCTGGTACCAGGCTCTCCTGCAGCTGCACAACCGCGCCAAGAGCCACCACCACGATGGGGCCGCGGGCCCCGGGGGCGGAGGCGGCGGCAGCTGCAGCGGCAGCTCTGGCGTAGGTGAGGCCGGGGAGGACTTGAGCTTCGGAGACATGGCCCCGGGGCCTGCGTTCAAAGAGGTCTGGCAGGTGATCCTGAAACCCAAAGGCCTGGGTCAAACAAAGAACCTGATCGGCATCTACCGGCTCTGCCTGACCAGCAAGACCATCAGCTTCGTGAAGTTGAACTCGGAGGCGGCCGCGGTGGTGCTACAGCTGATGAACATCAGACGCTGCGGCCACTCAGAGAACTTCTTCTTCATCGAGGTAGGCCGTTCCGCAGTGACAGGGCCCGGGGAGTTCTGGATGCAGGTGGATGACTCTGTGGTGGCCCAGAACATGCACGAGACCATTCTGGAAGCCATGCGCGCCATGAGTGATGAGTTTCGTCCGCGCAGCAAGAGCCAGTCCTCATCCAACTGCTCCAATCCCATCAGTGTCCCCTTGCGCAGGCACCATCTCAACAACCCGCCGCCTAGCCAGGTGGGGCTGACCCGCAGGTCCCGGACGGAAAGCATCACCGCTACCTCCCCCGCCAGCATCGTAGGTGGGAAGCCAGGTTCCTTCCGCGTGCGCGCCTCCAGCGATGGCGAAGGTACCATGTCGCGCCCGGCCTCGGTGGATGGCAGCCCTGTGAGTCCCAGCACCAACAGGACCCACGCCCACCGGCATCGGGGCAGCTCCAGGCTGCACCCTCCACTCAACCACAGCCGCTCCATCCCAATGCCTTCTTCTCGCTGCTCCCCTTCAGCCACCAGCCCCGTGAGCCTGTCGTCCAGCAGCACCAGTGGTCACGGCTCCACCTCGGACTGTCTCTTCCCCAGGCGGTCTAGTGCTTCTGTGTCGGGTTCCCCCAGCGATGGCGGTTTCATCTCTTCCGACGAGTACGGCTCCAGTCCCTGCGATTTCCGGAGTTCCTACCGCAGTGTCACTCCAGATTCCCTGGGCCACACCCCGCCAGCACGAGCCGAGGAAGAGCTGAGCAACTACATCTGTATGGGCGGCAAGGGGCCCTCCACCCTGACCGCTCCCAATGGTCACTTCATTTTGTCCCGGGGTGGCAACGGCCACCGCTACATCCCGGGGTCTGGTTTGGGGACGAGCCCAGCCTTGGTGGGCGATGATGCTACCAGCGGGGCTGATCTAGATAATCGGTTCCGGAAGAGAACACACTCAGCTGGCACATCCCCTACCATTTCCCACCAGAAGACCCCCTCGCAGTCTTCAGTGGCTTCCATTGAGGAGTACACGGAGATGACACCAGCCTACCCGCCAGGAGGTGGCGGTGGAGGCCGGCTACCAGGCTACCGGCACTCTGCCTTCGTGCCCACCCATTCCTATCCAGAGGAGGGTCTAGAGCTGCACCCCCTGGAGCGCCGAGGGGGCCACCACCGTGCTGACACGTCCACCCTCCACACGGATGATGGCTACATGCCCATGTCCCCAGGGGTGGCTCCAGTGCCTAACAACCGGAAGGGCAATGGGGATTATATGCCCATGAGTCCGAAGAGTGTGTCTGCCCCGCAGCAGATCATCAACCCCATCAGACGCCACCCTCAGAGAGTGGACCCCAATGGCTACATGATGATGTCCCCCAGTGGCAGCTGCTCCCCTGATATTGGCGGTGGGTCCAGTGGCGGCAGCAGCGTCAGTGTGGCCCTTTCTGGCAGCGGCTATGGGAAGCTGTGGACAAATGGAGTAGGTGGCCACCACGCCCACGCGCTGCCTCATGCCAAGCCCCCAGCGGAGAGCAGCGGTGGCAAGCTCTTGCCCTGCACAGGTGACTACATGAACATGTCACCCGTGGGGGACTCCAACACCAGCAGTCCCTCTGACTGCTACTGTGGCCCCGAGGACCCCCAGCACAAGCCAGTTCTCTCCTACTACTCATTGCCGAGGTCCTTTAAGCACACCCAGCGCCCCGGCGAGCCAGAGGAGGGTGCCCGACACCAGCACCTTCGCCTCTCCTCTAGCTCTGGTCGCCTCCTCTACCCTGCGGCTGCCGAAGATTCGTCCTCTTCCACCAGCAGCGACAGCCTGGGCGGGGGTTACTGTGCGACTAGGCCGGAACCTGGCCTCCCACATCCTCACCATCATGTCCTGCAGCCGCACGGGCCTCGGAAAGTAGACACGGCTGCACAGACCAACAGTCGCCTGGCTCGGCCCACAAGGCTGTCACTGGGGGATCCCAAGGCCAGCACCTTGCCCCGGGCACGAGAGCAGCAACCACTGCAGCAGCAAGTCCTGGTGCATCCTCCAGAGCCCAAGAGCCCAGGGGAGTATGTGAATATTGACTTCGGGAGTGACCAGCGTGGCTACTTACCTGGACCCATGGCTTCCCACGGGCCCCCTTCCGTACGGTGTCCACCCCAGCTCCAGCCAGCTCCCAGAGAGGAAGAGACTGGCGCAGAGGAGTATATGAACATGGACTTGGGGCCAGGCCGGAGGACAACCTGGAAGGAGAGCGGTGCAGTGGAGACGGGTAGAGTGGGCCCTGCACCCCCGGGGGCTGCTAGCGTTTGCAGACCAACCCGGGCAGTGCCTAGCGGCCGGGGTGACTACATGACCATGCAGATGGGTTGTCCCCGTCAGAGTTACGTGGAGACCTCACCTGTGACCCCCATCAGCTACGCTGACATGCGGACAGGCGTTGCTACAGAAGAGGTGAGCTTGCCTGGAGCCACTGCAGCTGCCCCTCCTCCATCCTCAGCGGCATCTGCTCCCCCCACTGCACCTCAAGAAGCAGCAGAGATACCTGGTGCCCACTCTTCTCTGCTGGGGGGCCCGCAGGCACCTGGGGGCATGAGTGCCTTCACCAGGGTTAATCTCAGTCCCAACCGCAACCAGAGTGCCAAAGTGATCCGTGCCGATCCACAAGGGTGCCGGAGGAGGCACAGTTCCGAGACCTTCTCCTCAACACCCACCGCCACCCGGGTGGGCAACACCGTGCCCTGTGGAGCAGGGGCTGCAGGAGCAGGCGGCGGCGGCAGCGAGGATGTGAAACGCCACAGCTGCGCCTCCTTTGAGAATGTGTGGCTAAGACCTGGGGAGCTAGCGGGGGGCCCCAAGGAGACAGCGTGCGGGGCCGCTGGAGGTGTGGAGAATGGCCTTAACTACATAGATCTGGATTTGGTCAAGGACTTCAAACAGCGCCCTCAGGAGCGACCCCCCCAGCCACAACCTCCACCACCCCCAGCTCCTCACCAGCCCCTGGGCAGTAGTGAGGGCAGCGCCCCCAGCCGCTCCAGTGATGATTTAAGCACCTATGCCAGCATCAGTTTCCAGAAGCAGCCAGAGGACCGCCAGTAG

>Ict_tr1 [Squirrel (Ictidomys tridecemlineatus) Irs1]

ATGGCGAGTCCTCCAGATACCGAAGGTTTCTCGGACGTGCGCAAGGTGGGCTACCTGCGCAAGCCCAAGAGCATGCACAAGCGCTTCTTCGTGCTGCGGGCGGCCAGCGAGGCCGGGGGCCCTGCGCGCTTGGAGTACTACGAGAACGAGAAGAAGTGGAGGCACAAGTCGAGCGCCCCCAAACGCTCGATCCCCCTCGAGAGCTGCTTCAACATCAACAAACGTGCTGACTCCAAGAACAAGCACCTGGTGGCTCTCTATACCCGGGACGAGCACTTTGCCATTGCTGCGGACAGTGAGGCTGAGCAAGACAGCTGGTACCAGGCTCTCCTGCAGCTGCACAACCGTGCCAAAAGCCACCACCACGAAGGGGCCTCAGCCCCTGGAGCAGGAGGCAGTGGTAGCTGCAGTGGCAGCTTGGGCCTCGGCGAGGCTGGAGAGGACTTGAGCTATGGAGATGGGCCAGGACCTGCGTTTAAGGAGGTCTGGCAGGTGATCTTGAAGCCCAAGGGCCTGGGTCAGACAAAGAACCTCATTGGTATCTACCGCCTCTGCCTGACCAGCAAGACCATTAGCTTTGTGAAGCTGAACTCAGAGGCAGCGGCTGTGGTGCTACAGCTAATGAACATCAGACGCTGCGGCCACTCTGAAAACTTCTTTTTCATTGAGGTGGGCCGTTCTGCTGTGACAGGGCCTGGGGAGTTCTGGATGCAGGTGGATGACTCGGTGGTGGCCCAGAACATGCATGAGACTATCTTGGAGGCCATGCGGGCCATGAGTGATGAGTTTCGCCCTCGAAGCAAGAGCCAGTCCTCATCCAACTGCTCCAACCCCATCAGTGTCCCCTTGCGCAGGCACCATCTCAACAACCCCCCGCCCAGCCAGGTGGGGTTGACCCGCAGATCACGCACTGAAAGCATCACTGCCACCTCCCCTGCCAGTATGGTAGGTGGGAAGCCAGGATCCTTCCGTGTGCGCGCCTCCAGCGATGGTGAAGGCACCATGTCCCGCCCAGCCTCTGTGGATGGCAGCCCTGTGAGTCCTAGCACCAACAGAACCCACGCCCACCGGCATCGGGGCAGCTCCCGGCTGCACCCGCCTCTCAACCACAGTCGCTCCATCCCCATGCCTTCTTCTCGCTGCTCGCCTTCGGCAACCAGCCCAGTTAGTCTATCGTCCAGCAGCACCAGTGGCCATGGTTCCACTTCGGACTGTCTGTTTCCAAGGCGGTCTAGTGCTTCCGTGTCTGGTTCCCCCAGTGATGGCGGTTTCATCTCTTCAGATGAGTATGGCTCTAGCCCCTGCGACTTCCGAAGTTCCTTCCGCAGTGTCACTCCAGATTCCTTGGGCCACACCCCACCAGCCCGAGGCGAGGAGGAGCTGAGCAATTATATATGCATGGGTGGAAAGGGGGCCTCCACTCTAACAGCTCCCAATGGTCATTACATTTTGTCCCGAGGTGGTAATGGCCACCGGTACATCCCAGGAGCTGGCTTGGGCACCAGCCCAGCGTTGTTGACCGGGGATGAAGCTGCCAGTGCTGCTGATCTGGATAATAGGTTCCGGAAGAGAACCCACTCTGCAGGGACATCCCCTACCATTTCCCACCAGAAGACCCCATCCCAGTCCTCAGTGGCTTCCATTGAGGAGTATACAGAGATGATGCCTGCCTATCCGCCAGGAGGTGGCAGTGGAGGCCGACTGCCTGGCTACAGGCACTCTGCCTTCGTGCCCACCCATTCCTACCCTGAGGAGGGTCTAGAGATGCATGCCTTGGAGCACCGCAGCAGCCACCATCGCCCAGATAGTTCCACCCTCCACACTGATGATGGATACATGCCCATGTCCCCAGGAGTGGCCCCAGTGCCCAATAGCCGGAAGGGCAGTGGGGATTATATGCCCATGAGCCCCAAGAGCGTGTCTGCCCCGCAGCAGATCATCAACCCCATCAGACGTCATCCCCAGAGAGTGGACCCCAATGGCTACATGATGATGTCCCCCAGTGGCAGCTGCTCCCCTGACATTGGAGGTGGGTCCAGCAGCAGCAGCAGCATCAGTGCCGCCCCTTCTGGCAGTAGCTATGGGAAGCTATGGACAAATGGGGTCAGTGGGCACCACTCTCATGCCCTGTCTCACCCCAAGCCCCCTGTGGAGAGTGGGGGTGGCAAGCTCTTGCCTTGTACAGGTGACTACATGAACATGTCACCAGTGGGGGACTCCAACACCAGCAGCCCCTCTGACTGCTACTATGGCCCTGAGGATCCCCAGCACAAGCCAGTCCTTTCCTACTATTCATTGCCAAGGTCTTTTAAGCACACCCAGCGCCCTGGGGAGCCAGAGGAGGGTGCCCGGCACCAGCATCTCCGCCTCTCCTCTAGCTCTGGTCGCCTTCTCTACACTGCAACTGCAGAAGATTCTTCTTCCTCCACCAGCAGCGACAGCCTGGGTGGGGGTTACTGTGGGGCTAGGCCAGAGCCTGGCCTCCCACATCCCCACCATCATGTCTTGCAGCCCCATTTGCCTCGAAAAGTGGACACAGCTGCTCAGACCAACAGCCGCCTGGCCCGGCCCACAAGGCTATCTTTGGGGGATCCCAAGGCCAGCACCTTGCCTCGGGCTCGAGAACAGCAACAGCAGCAGCCCCTACTGCACCCTCCGGAGCCCAAAAGCCCAGGGGAATATGTGAATATTGAATTTGGGAATGACCAGCCTGGCTACTTATCTGGCCCTATGACTTCCCGCAGCTCACCTTCTGTTCGGTGTCCATCCCAGCTCCAGCCAGCTCCCAGAGAGGACGAGACTGGCACTGAGGAGTACATGAACATGGACTTGGGGCCAGGCCGGAGGGCAACCTGGCAGGAGAGCAGTGGGGGGGAGATGGGCAGAGTGGGCCCTGCACCTCCAGGGACTACTAGCATTTGCAGGCCTACCCGGGCAGTGCCCAGCAGCCGGGGTGACTACATGACCATGCAGATGGGTTGTCCCCGTCAGAGCTACGTGGACACCTCACCAGTTGCCCCTGTCAGCTACGCTGACATGCGGACAGGCATTGCTGCAGAGGAGGTGAAACTGCCCAGGGCCTCAGCGGCTGCTCCTCCTTCAGCCTCCAAAGCCTCTTCTTCCCCTGCTGCACCTCAAGGAGCAACTGAGCTTCCTGCCCACTCTTCCCTTTTGGGGGGCCCACAGGGACCTGGGGGCATTAGCGCCTTCACCCGGGTAAACCTCAGTCCCAACCGCAACCAGAGTGCCAAAGTGATCCGTGCAGATCCACAAGGGTGCCGGAGGAGGCATAGCTCAGAGACCTTCTCCTCAACACCTACTGCTTCCCGGGGGGCCAACACGGTGCCCTTTGGAGGAGGGGCTGCAGTAGAGGGCAGTGGCGGTGGTGGCAGCAACAGCAGTGAGGATGTCAAACGCCACAGCTCTGCTTCCTTTGAGAATGTCTGGCTGAGACCTGGGGAGCTCGGCGGAGCCCCCAAGGAGGTGGCCCAAGGGTGTGGAGCTGCTGGGGGTTTGGAGAATGGTCTTAACTACATAGACCTGGATTTGGTCAAGGACTTCAAACAGCGCCCTCAGGAGCGCCCCCCTCAACCACAGCCTCCCCCACCTCCACCCCCTCATCAGCCCCTGGGCAGTAGTGAGAGCAGCTCTACCAGCCGCTCCAGCGAGGATTTAAGCACCTATGCCAGCATCAGTTTCCAGAAGCAGCCAGAGGACCGCCAGTAG

>Oct_de1 [Degu (Octodon degus) Irs1]

ATGGCGAGCCCACCGGATACCGAGGGCTTCTCGGACGTGCGCAAGGTTGGCTACCTGCGCAAACCCAAAAGCATGCACAAGCGCTTTTTCGTGCTGCGGGCAGCCAGCGAGGCCGGGGGCCCGGCGCGTCTGGAGTACTATGAGAACGAGAAGAAGTGGCGGCACAAGTCGAGCGCCCCCAAACGCTCGATCCCCCTGGAGAGCTGCTTCAACATCAACAAGCGGGCTGACTCCAAGAACAAGCATCTGGTGGCTCTCTACACCCGGGATGAGCACTTTGCCATTGCGGCGGACAGCGAGGCCGAGCAAGACAGCTGGTACCAGGCTCTCCTGCAGCTGCACAACCGTGCCAAGGGCCACCACGACGGGGCTGCGGTGCCCGGAGCGGGAGGCTGTGGGGGCAGCTGCAGCGGCAGCTCCGGTCTTGGGGAGGCCGGGGAGGACTTGAGCTATGGAGATGGACCCCCAGGACCTGCGTTCAAGGAGGTCTGGCAGGTGATCCTGAAGCCCAAGGGCTTGGGTCAGACAAAGAACCTGATTGGCATCTACCGCCTCTGCCTGACCAGCAAGACCATCAGCTTCGTGAAGTTGAATTCTGAGGCAGCGGCCGTGGTGCTGCAATTGATGAACATCAGGCGTTGCGGCCACTCGGAGAACTTCTTCTTCATCGAGGTGGGCCGTTCTGCAGTGACGGGGCCTGGTGAGTTCTGGATGCAGGTAGATGACTCGGTGGTGGCCCAGAACATGCATGAGACGATCCTGGAAGCCATGCGGGCTATGAGCGATGAGTTTCGCCCCCGCAGCAAAAGCCAGTCTTCATCCAACTGCTCCAACCCCATCAGCGTCCCCCTGCGCCGGCACCATCTCAACAACCCTCCGCCCAGCCAGGTGGGACTGACCCGCCGGTCTCGCACGGAGAGCATCACTGCTACCTCTCCAGCCAGCATGGTGGGCGGGAAGCCTGGTTCCTTCCGCGTGCGCGCTTCCAGTGATGGTGAGGGCACCATGTCCCGTCCAGCCTCGGTGGATGGCAGCCCTGTGAGTCCTAGCACCAACAGAACGCATGCCCACCGGCATCGAGGCAGCTCCCGCCTGCATCCCCCACTCAACCACAGCCGCTCCATCCCCATGCCTTCCTCGCGCTGCTCACCTTCGGCCACCAGCCCCGTGAGCTTGTCGTCCAGCAGCACCAGTGGCCACGGCTCCACTTCAGACTGCCTCTTTCCAAGGCGCTCGAGCGCTTCTGTTTCTGGCTCCCCCAGTGACGGTGGTTTCATCTCTTCTGATGAATATGGCTCTAGTCCCTGTGATTTCCGAAGTTCTTTCCGCAGTGTCACCCCAGATTCCCTGGGCCACACCCCACCGGCCCGTGGAGAGGAGGAGCTGAGCAACTACATCTGCATGGGTGGCAAGGGGGCCTCCACCCTGACGGCTCCCAATGGTCACTACATTTTGTCTCGGGGTGGCAATGGCCACCGCTACATCCCAGGAGCGGGCTTGGGCACCAGCCCAGCATTGGCTGGGGATGAAATAGCCAGTGCTGCAGATCTGGATAATCGGTTTCGGAAGAGGACTCACTCAGCAGGGGCGTCCCCTACTATTTCCCACCAGAAGACCCCCTCCCAGTCCTCAGTGGCTTCCATTGAGGACTACACAGAGATGACACCTGCCTGCCCACCAGGAGGTGGCAGTGGAGGCCGACTGCCCAGCTACCGGCACTCCGCCTTTGTGCCCACCCACTCCTACCCAGAGGAGGGTCTAGAGATGCACCCCACAGAGCACCGTGGGGGTCACCACCGCCCAGACCCCTCCAGCCTCCACACAGATGATGGCTACATGCCCATGTCGCCAGGGGTGGCCCCTGTGCCTGGCAACCGAAAGGGCAGTGGGGACTATATGCCCATGAGTCCCAAGAGCGTGTCTGCCCCACAGCAGATCATCAACCCCATCAGACGCCACCCCCAGAGAGTAGACCCTAACGGCTACATGATGATGTCTCCCAGTGGGAGCTGCTCCCCTGACATGGGAGGTGGGTCGAGCAGCAGTAGCAGCATTAGCGTCGCCCCTTCCGGGGGTAGCTATGGGAAGCTGTGGACCAATGGAGTTGGAGGCCACCACTCTCATGCCCTGTCTCATCCCAAGGCCCCTCTGGAGAGCGGTAGTGGCAAGCTCTTGCCTTGTACAAGTGACTACATGAACATGTCGCCCGTTGGAGACTCCAATACCAGCAGCCCCTCTGAATGCTATTATGGCCCTGAGGACCCCCAGCACAAACCTGTCCTATCCTATTACTCATTGCCAAGGTCTTTCAAGCACACCCAACGCCCCGGGGAACTGGAGGAAGGAGCCCGGCATCAGCACCTCCGCCTTTCCTCCAGCTCTGGTCGTCTTCTCTACACTGCAGCAGCAGAAGATTCTTCCTCTTCCACCAGCAGCGACAGCCTGGGTGGGGGCTACTGTGGGGTGAGGCCAGAGCCTGGCCTCCCACACCCCCACCACCACGTCCTGCAGCCCCGTCTGCCCCGAAAGGTGGACACAGCTGCACAGACCCACAGCCGCCTGGCCCGGCCTACAAGGCTATCCCTGGGGGATCCCAAGGCCAGCACCTTACCTCGGGCTCGGGAGCAGCAACCACAGCCACCCCTGATACACTCTCCAGAGCCCAAAAGCCCAGGGGAATATGTAAATATTGAATTTGGGAGTGACCAGCCTGGCTACTTATCTGGTCCCATGGCTTCCCGCAGTGGCCCTTCTGTCCGGTGTCCCTCTCAGCTCCAGCCAGCTCCCAGAGAGAAGGAAACTGGCACCGAGGAGTACATGAATATGGACCTGGGCCCTGGCCGGAGGGCAGTCTGGCAGGAGGGTGGTGGGGCTGAGATGGGCAGAGTGGGCCCAGCACCTCCAGGGGCCACTAGTGTTTGCAGGCCTACCCGGGCAGTGCCTAGCAGCCAGGGTGACTACATGACCATGCAGATGGGTTGTCCTCGTCAGAGTTACGTGGACACCTCACCCGTCGCTCCTGTCAGCTATGCTGAGATGCGGACAGGAATTGCAGAAGAGGTGAGCCTGCCCAGGGCCACCATGGCCACTCTCTCTTCATCTGCAGCAGCCCCTGCTTCCCCCACACCACCTCCAGGAGCAGCTGAGTTGGCTGCCCACTCATCCCTGCTGGGGGGTCCACAGGGACCTGGGGGCACGAGCGCCTTCACCAGGGTAAATCTCAGTCCCAACCGCAACCAGAGTGCCAAAGTGATCCGTGCAGACCCACAAGGGTGTCGGAGGAGGCACAGCTCAGAAACCTTCTCCTCAACGCCTACTGCCACCCGGGTGGGCAACATGGTGCCCTTTGGAGGGGATGCTGCAGTCGGGAGCAGCGGTGGTGGCAGCAGCAGCAGTGAGGATGTGAAACGCCACAGCTCTGCGTCTTTTGAGAATGTGTGGCTAAGACCCGGGGAGCTAGGGGGAGCACCCAAGGACACAGCCCAAGTGTGTGGGGCAGCTGGGGGTTTGGAAAATGGTCTTAACTACATAGACCTGGATTTGGTCAAAAACTTGAAACAGCGCCCTCAGGAGCGCCCCCCTCAACTGCAGCCTCCCCTGCCCCCAGCCCCTCATCAGCCCCTGGGTGGCAGTGAGAGCAGCTCAACCAGCCGCTCCAGCGAGGATTTAAGCGCCTATGCCAGCATCAGTTTCCAGAAGCAGCCGGAGGACCACCAGTAG

>Het_gl1 [Naked mole rat (Heterocephalus glaber) Irs1]

ATGGCGAGCCCTCCAGATACCGATGGCTTCTCGGACGTGCGCAAGGTTGGCTACCTGCGCAAACCCAAGAGCATGCACAAGCGCTTCTTCGTGCTGCGGGCTGCCAGCGAGGCCGGGGGCCCGGCGCGCCTGGAGTACTACGAGAATGAGAAGAAGTGGCGGCACAAGTCGAGCGCCCCCAAACGCTCTATCCCCCTGGAGACCTGCTTCAACATCAACAAGCGGGCTGACTCCAAGAACAAGCACCTGGTGGCTCTCTACACCCGGGACGAGCACTTTGCCATTGCGGCGGACAGCGAGGCCGAGCAAGACAGCTGGTACCAGGCTCTCCTGCAGCTGCACAACCGAGCCAAGGGCCACCACGATGGGGCCGCAGTCCCCGGGGCCGGAGGCGGCGGGGGCAGCTGCAGCGGCAGCTCTGGCGTTGGCGAGGCCGGGGAGGACCTGAGCTACGGAGATGTGCCCCCCGGACCTGCGTTCAAGGAGGTCTGGCAGGTGATCCTGAAACCCAAGGGCTTGGGTCAGACAAAGAACCTGATCGGCATCTACCGCCTCTGCCTGACCAGCAAGACCATCAGCTTCGTGAAGCTGAACTCGGAGGCGGCGGCCGTGGTGCTGCAGCTGATGAACATCAGGCGCTGCGGCCACTCAGAGAACTTCTTCTTCATTGAGGTGGGCCGCTCCGCTGTGACAGGGCCCGGGGAGTTCTGGATGCAGGTGGACGACTCGGTGGTGGCCCAGAACATGCACGAGACCATCCTGGAAGCCATGCGGGCTATGAGCGACGAGTTTCGCCCCCGCAGCAAGAGTCAGTCCTCATCCAACTGCTCCAACCCCATCAGCGTTCCCCTGCGCAGGCACCATCTCAACAACCCCCCGCCCAGCCAGGTGGGACTGACCCGCCGGTCCCGCACCGAGAGCATCACCGCCACCTCTCCGGCCAGCATGGTGGGCGGGAAGCCCGGGTCTTTCCGCGTGCGCGCCTCCAGCGACGGTGAGGGCACCATGTCCCGGCCAGCCTCGGTGGATGGCAGTCCTGTGAGTCCCAGTACTAACAGGACCCATGCCCACCGGCATCGGGGCAGCTCCCGGCTGCACCCCCCACTCAACCACAGCCGCTCCATCCCCATGCCGTCTTCTCGCTGCTCACCCTCGGCCACCAGCCCCGTGAGCCTGTCCTCCAGCAGCACCAGCGGCCACGGCTCCACCTCAGACTGCCTCTTTCCCAGGCGCTCCAGTGCTTCGGTTTCAGGCTCCCCCAGCGATGGCGGTTTCATCTCTTCTGATGAGTACGGCTCGAGTCCCTGTGATTTCCGAAGTTCTTTCCGCAGTGTCACCCCGGACTCCCTGGGCCACACCCCACCAGCCCGTGGAGAGGAGGAGCTGAGCAACTACATCTGCATGGGCGGCAAGGGGGCCTCCACCCTGACGGCTCCCAATGGTCACTACATTTTGTCCCGGGGTGGCAATGGCCACCGCTACCTCCCCGGAGCTGGCTTGGGCACCAGCCCGGCGTTGGCTGGGGAGGAAGCCGCCAGTGCTGCGGACCTGGAGAATCGGTTCCGGAAGAGGACTCACTCAGCAGGGACGTCCCCGACCATCTCCCACCAGAAGACGCCCTCGCAGTCCTCCGTGGCGTCCATTGAGGAGTACACGGAGATGATGCCTGCCTACCCGCCAGGAGGTGGCAGTGGAGGCCGACTGCCCAGCTACCGGCACTCCGCCTTTGTGCCCACCCACTCCTACCCTGAGGAGGGCCTGGAGATGCACCCGATGGAGCAGCGTGGGGGCCACCCCCGCCCAGACAGCTCCGGCCTGCACACCGATGACGGCTACATGCCCATGTCTCCAGGGGTGGCCCCAGTGCCTGGCAGCCGAAAGGGCAGCGGGGACTATATGCCCATGAGTCCCAAGAGCGTGTCGGCCCCTCAGCAGATCATCAACCCTATCCGCCGCCATCCCCAGAGGGTCGACCCCAACGGCTACATGATGATGTCTCCCAGCGGCAGCTGCTCCCCGGACATCGGAGGCGGGTCCAGCAGCAGCAGCAGCATTAGCGCCGCCCCTTCCGGGAGTAGCTATGGGAAGCTGTGGACGAATGGAGTTGGGGGCCACCACTCTCATGCCCTGTCTCACCCCAGGCCCCCTGTGGAGAGCGGTGGCAGCAAGCTCCTGCCTTGTACCAGCGACTACATGAACATGTCGCCAGTTGGGGACTCCAACACCAGCAGCCCCTCTGACTGCTATTACGGCTCCGAGGACCCCCAGCACAAGCCCGTCCTCTCCTATTACTCATTGCCAAGGTCCTTTAAGCATACCCAGCGGCCTGGGGAGCCGGAGGAAGGTGCCCGGCACCAGCACCTCCGCCTCTCCTCCAGCTCTGGTCGTCTTCTCTACGCTGCAGCGGCGGAAGATTCTTCGTCCTCCACCAGCAGCGACAGTCTGGGTGGAGGTTACTGCGGGGCGAGGCCGGAGGCCGGTCCCCCGCATCCCCACCATCACGTCCTGCAGCCCCGTCTGCCTCGAAAGGTGGACACAGCTGCGCAGAGCAACAGCCGCCTGGCCCGGCCCACCAGGCTGTCCCTGGGGGATCCCAAGGCCAGCACCTTACCTCGGGCTCGGGAGCAGCAGCAGCCGCCCCTGGTGCACCCTCCAGAGCCCAAGAGCCCAGGAGAATATGTGAATATTGAATTTGGGAGCGGCCAGCCTGGCTACTTCTCTGGTCCCATGGCTTCCCGCAGTTTCCCTTCTGTCCGGTGTCAGCTCCAGCCGGCTCCCAGGGAGGAAGAGACTGGCACCGAGGAGTACATGAACATGGACCTGGGGCCTGGCCGGAGGGCAGCCTGGCAGGAGAGTAGTGGGGCAGAGACGGGCAGAGCGGGCCCTGCTCCCCCGGGGGCTGCCAGCGTTTGCAGGCCTACCCGGGCAGTGCCCAGCAGCCGGGGGGACTACATGACCATGCAGATGGGTTGTCCTCGCCAGAACTACGTGGACACCTCACCAGTCACGCCCGTCAGCTATGCTGACATGCGGACAGGCATTGCAGAGGAGGTGAGCCTGCCCAGGGCCACCGTGGCTGCCCCCTCTTCATCTTCGGCAGCCCCTGCTTCCCCCACAGCACCTCAAGGAGCAGCTGAGCTGGCGGCCCACTCCTCCCTGCTGGGGGGCCCACAGGGACCTGGGGGCGTGAGCGCCTTCACCAGGGTGAACCTCAGTCCCAACCGCAACCAGAGTGCCAAAGTGATCCGCGCGGACGCACCAGGGTGTCGGCGGAGGCATAGCTCCGAAACCTTCTCCTCAACACCTGCCGCCACCCGTGCGGGCAACACGGTGCCCTTTGGAGGGGGGGCTGCAGTAGGGGGCAGTGGTGGCAGCAGCAGCAGCAGCGAGGATGTGAAACGCCACAGTTCTGCTTCGTTTGAGAACGTGTGGCCAAGACCTGGGGAGCAAGGGGGTGCCCCCAAGGACACGGCCCAAGCTTGTGCAGCAGCTGGGGGTTTGGAAAATGGTCTTCACTACATAGACCTGGATTTGGTCAAAGACTTGAAACAGCGCCCTCAGGAGCGCCCCTCTCAGCTGCAGGCTCCTCTTCCCCCACCCCCTCATCATCCCCTGGGCAGCAGTGAGAGCAGCTCGACCAGCCGCTCCAGTGAGGATTTAAGCGCCTATGCCAGCATCAGTTTCCAGAAGCAGCCAGAGGACCGCCAGTAG

>Nan_ga1 [Blind mole rat (Nannospalax galili) Irs1]

ATGGCGAGCCCTCCGGATACTGATGGCTTCTCGGACGTGCGCAAGGTGGGCTACCTGCGCAAACCCAAGAGTATGCACAAGCGCTTTTTCGTGCTGCGGGCTGCCAGCGAGGCCGGGGGCCCGGCGCGACTGGAGTACTATGAGAACGAGAAGAAGTGGCGCCACAAGTCGAGCGCCCCCAAACGCTCGATCCCCCTCGAGAGCTGTTTCAACATTAACAAGCGAGCTGACTCCAAGAACAAGCACCTGGTGGCTCTCTACACCCGGGACGAGCACTTTGCCATTGCCGCGGATAGCGAGGCCGAGCAGGACAGCTGGTACCAGGCTCTCCTGCAGCTGCACAACCGAGCAAAGGCCCACCATGACGCAGCCCCCGGAGGAGGCGGCGGTAGCTGCAGCGGCAGCTCCGGCCTCGGGGAGGCAGGGGAGGACTTGAGCTATGGAGACACGGGCCCAGGACCTGCGTTCAAGGAGGTCTGGCAGGTGATCCTCAAACCCAAAGGCCTGGGTCAGACAAAGAACTTGATTGGCATCTACCGCCTCTGCCTGACCAGCAAGACCATCAGCTTCGTGAAGCTGAACACCGACGCAGCCGCTGTGGTGCTGCAGCTGATGAACATCAGACGCTGCGGCCACTCCGAGAACTTCTTCTTCATCGAGGTGGGCCGTTCAGCAGTGACAGGGCCTGGCGAGTTCTGGATGCAGGTAGATGACTCCGTGGTGGCCCAGAACATGCACGAGACCATCCTAGAGGCGATGCGGGCCATGAGTGATGAGTTTCGCCCTCGAAGCAAGAGTCAGTCTTCGTCCAATTGCTCCAACCCCATCAGCGTCCCCCTCCGCAGACACCATCTCAACAATCCTCCACCCAGCCAGGTGGGGCTGACCCGCCGATCGCGCACCGAGAGCATCACTGCCACCTCTCCTGCCAGCATGGCGGGAGGGAAGCCAGGCTCCTTCCGCGTGCGCGCCTCCAGCGATGGCGAAGGCACCATGTCCCGCCCGGCCTCGGTGGACGGCAGCCCCGTGAGTCCTAGCACCAACAGGACCCACGCCCATCGGCATCGTGGTAGCTCCAGGCTGCACCCCCCACTCAACCACAGCCGTTCCATCCCGATGCCTTCTTCTCGCTGCTCACCTTCAGCCACCAGCCCAGTTAGTCTGTCGTCCAGTAGCACCAGTGGCCACGGCTCCACCTCAGACTGTCTCTTTCCCCGGCGGTCCAGTGCCTCTGTGTCCGGCTCTCCCAGCGATGGCGGTTTCATTTCTTCTGATGAATATGGCTCTAGTCCCTGTGATTTCCGAAGTTCCTTCCGTAGTGTCACTCCAGATTCCCTGGGCCACACCCCACCGGCACGGGGCGAGGAGGAGCTGAGCAACTATATCTGCATGGGTGGCAAGGGGACCTCCACTTTGGCTGCCCCCAATGGTCACTACATTTTGTCTCGGGGTGGCAATGGTCACCGTTACATCCCAGGAGCTAACTTGGGGACAAGCCCAGCCTTGACTGGGGAGGAAGCCGCCAACGCTGCAGATCTGGATAACCGCTTTCGGAAGAGAACCCACTCAGCAGGGACATCGCCAACCATTTCTCACCAGAAGACCCCCTCACAGTCCTCTGTGGCTTCTATTGAGGAATATACAGAGATGATGCCAACCTACCCACCAGGAGGGGGCAGTGGAGGCCGACTGCCCAGTTACCGGCATTCTGCCTTTGTGCCCACCCACTCCTATCCCGAGGAGGGGCTAGAGATGCCCGCCTTAGAGCGTCGTGGTGGCCACCATCGTCCAGACTCCACCCTCCACACTGATGATGGCTACATGCCCATGTCCCCAGGGGTGGCTCCAGTACCCAGCAACCGAAAAGGAAATGGAGACTATATGCCCATGAGCCCCAAGAGTGTATCTGCCCCACAGCAGATCATCAACCCCATCAGACGCCACCCTCAGAGAGTTGACCCCAATGGCTACATGATGATGTCCCCCAGTGGCAGCTGCTCTCCTGACATTGGAGGCCGGTCCAGCAACAACAGCAGTGCAGCCCCTTCTGGGAGCAGCTATGGGAAGCTATGGACAAATGGCGTAGCAGGCCATCATTCTCATGCCCTGACTCATGCCAAACCTGTAGAGAGCAGCGGTGGCAAACTCTTGCCTTGCACAGGTGACTACATGAACATGTCCCCAGTGGGGGACTCCAACACTAGCAGCCCCTCAGACTGCTACTACGGCCCAGAGGACCCCCAGCACAAGCCTGTACTCTCTTACTACTCCTTACCAAGGTCCTTTAAGCACACCCAGCGCCCCAGGGAGTCAGAGGAGGGTGCTCGGCACCAGCATCTTCGCCTCTCCTCCAGCTCCGGTCGCCTTCTCTACACTGCAACTGCGGAAGACTCTTCCTCTTCTACCAGCAGTGACAGTGTTGGTGGGAGTTACTGTGGGGCTAGGCCCCACCACCATGTCCTGCAGCCCCATCTTCCTCGAAAGGTGGACACAGCTGCTCAGACCAACGGCCGCCTGCCTCGGCCCACAAAGCTATCCCTGGGGGATCTCAAGGCCAGCACCTTACCTCGGGCTCGAGAGCAACAGCAACAGCCACAACAAACTCCGCTGCACCCTCCTGAGCCCAAGAGTCCAGGGGAATATGTGAATATTGAATTTGGGAGTGACCAGCCTGGCTATTTAGCTGGCCCCATGACTTCCCGTAGCTCCCCTTCTGTTCGGTGTCCACCCCAGCTCCAGCCAGCTCCCAGAGAGGAAGAGACTGGCACTGAGGAGTACATGAACATGGACTTGGGGCCAGGCCGGAGGGCAGCCTGGCAGGAGAGTGGTGGGGAGATGGGCAGAGTGGGCCCGGCACCTCCAGGGGCTGCCACTGCTAGCAGGCCTACCCGGTTGGTGCCCAGTAGCCGGGGTGACTACATGACCATGCAGATGGGTCATCCTCGTCAGAGCTACGTGGATACCTCTCCAGTGGCCCCTGTCAGCTATGCTGACATGCGGACAGGCATTGCTACAGAGGAGGTGAGCCTGCCTAGAGCCACAGCAGCTGCTCCCTCGTCGTCTTCCACAGCCTCTGCTTCTCCCGCTGCGCCTCAAGGAGCAGCTGAGGTAGCTGCTCGCTCTTCCCTGCTGGGGGGTCCTCAGGGACCTGGGGGCATAAGCGCCTTCACCAGGGTGAACCTCAGTCCTAACCGCAACCAGAGTGCCAAAGTGATCCGAGCAGACCCACAAGGGTGCCGGAGGAGGCATAGCTCCGAGACATTCTCCTCAACCCCTACTGCCACCCGGGCTGGCAACACTGTGCCCTTTGGAGCAGGGGCTGCAGTAGGGGGCGGTGGCGGGGGCAGCAGCAGTGAGGATGTAAAACGCCACAGCTCTGCATCATTTGAAAACGTGTGGCTGAAACCTGGGGAGCCAGGGGGAGCTCCCAAGGAGCCGACTCAAGTGTGTGGGGCTGCTGCAGGGGTGGAGAATGGACTTAACTACATAGACCTGGATTTGGTCAAGGACTTCAAACAGCGCCCTCAGGAGCGCCCCCCTCAACAGCAGCCCCCATCTTCCCCAGCCCCTCACCAGCCCCCGGGCTGCAGTGAGGCGAGCCCCACCAGCCGCTCCAGCGAGGAATTAAGCGCCTATGCCAGCATCAGCTTTCAGAAGCAGCCAGAGGACCGTCAGTAG

>Cav_po1 [Guinea pig (Cavia porcellus) Irs1]

ATGGCGAGCCCACCGGATACCGATGGCTTCTCGGACGTGCGCAAGGTTGGCTACCTGCGCAAACCCAAGAGCATGCACAAGCGCTTCTTCGTGCTGCGAGCAGCTAGCGAGGCCGGGGGCCCTGCGCGCTTGGAGTACTACGAGAACGAGAAGAAGTGGCGGCACAAGTCGAGCGCCCCCAAACGCTCTATCCCTTTGGAGAGCTGTTTCAACATCAACAAGCGGGCTGACTCCAAGAACAAGCACCTGGTGGCTCTCTACACCCGAGATGAGCACTTTGCCATTGCGGCAGACAGCGAGGCTGAGCAAGACAGCTGGTACCAGGCTCTCCTGCAGCTGCACAACCGTGCCAAGGGCCACCACGACGGGGCCTCAGTCTCTGGTGCAGGAGGTGGTGGGGGCAGCTGCAGCGGCAGCTCAGGCCTTGGTGAGGCCGGGGAGGACTTGAGCTATGGAGATGCGCCCCCGGGACCTGCATTCAAGGAGGTCTGGCAGGTGATCCTGAAACCCAAGGGCTTGGGTCAGACAAAGAACCTGATTGGCATCTACCGTCTCTGCCTGACCAGCAAGACCATCAGCTTCGTGAAGCTGAACTCAGAAGCAGCAGCCGTGGTGCTCCAACTGATGAACATCAGGCGCTGCGGCCACTCTGAGAACTTCTTCTTTATTGAGGTGGGTCGTTCTGCAGTGACAGGGCCTGGGGAATTCTGGATGCAGGTGGATGACTCGGTGGTTGCCCAGAACATGCACGAGACTATCTTGGAAGCCATGCGGGCTATGAGTGATGAATTTCGCCCTCGCAGCAAGAGCCAGTCTTCATCCAACTGCTCCAACCCCATCAGCGTCCCCCTGCGCAGGCACCATCTCAACAACCCTCCGCCTAGCCAAGTGGGCCTGACCCGCCGATCTCGTACAGAGAGCATCACTGCCACCTCTCCAGCCAGCATGGTGGGCGGGAAACCGGGTTCCTTCCGGGTGCGTGCCTCCAGCGACGGTGAAGGCACCATGTCCCGGCCAGCTTCAGTGGATGGCAGTCCTGTGAGTCCTAGTACCAATAGGACCCATGCCCACCGGCATCGGGGCAGCTCCCGGCTACACCCCCCACTCAATCACAGCCGCTCCATCCCTATGCCTTCCTCACGTTGCTCACCCTCAGCCACCAGCCCAGTGAGTCTGTCCTCCAGCAGCACCAGTGGCCATGGCTCCACCTCGGACTGTCTCTTTCCAAGGCGCTCTAGTGCTTCTGTTTCTGGTTCTCCTAGTGATGGCGGTTTCATCTCTTCTGATGAGTATGGCTCTAGTCCCTGTGATTTCCGAAGTTCTTTCCGCAGTGTCACCCCGGATTCCTTGGGCCACACCCCACCAGCCCGTGGAGAGGAGGAGCTGAGCAACTACATCTGCATGGGTGGCAAGGGGGCCTCCACCCTGACGGCTCCCAATGGTCACTACATTTTGTCTCGGGGTGGCAATGGCCACCGCTACATCCCAGGAGCAGCAGGTTTGGGCACAAGCCCAGCCTTGACTGGGGATGAAGCAGCCAGTGCTGCAGATCTGGATAATCGGTTCCGAAAGAGGACTCACTCAGCAGGGACATCCCCTACCATTTCCCACCAGAAGACCCCTTCGCAGTCCTCAGTGGCTTCCATTGAGGAATACACAGAGATGATGCCTGCCTACCCACCAGGAGGTGGCAGTGGAGGCCGACTGCCCAGCTACCGGCACTCCGCCTTTGTGCCCACCCACTCCTACCCTGAGGAGGGTCTAGAGATGCACCCTATGGAGCAGCGTGGGGGCCACCACCGCCCAGACACCTCCAGCCTCCACACCGATGATGGCTACATGCCCATGTCTCCAGGGGTGGCCCCAGTGCCTGGCAACCGAAAGGGCAGTGGAGACTATATGCCCATGAGTCCCAAGAGTGTGTCTGCCCCACAGCAGATCATCAATCCCATCAGACGCCATCCCCAGAGAGTGGACCCCAATGGCTACATGATGATGTCTCCCAGTGGCAGCTGCTCCCCTGACATTGGAGGTGGGTCGAGTAGCAGTAGCAGCATTAGTGCTGCCCCTTCTGGAAGTAGCTATGGGAAACCATGGACAAATGGAGTTGGAGGCCACCACTCTCATGCTCTGCCTCACGCCAAGCCCCCTGTGGAGAGTGGTGGTGGCAAGCTCTTGCCTTGTACAAGTGACTACATGAACATGTCACCAGTTGGGGACTCCAACACCAGCAGCCCCTCTGACTGCTATTATGGCCCTGAGGACCCTCAGCACAAGCCAGTCCTCTCCTATTACTCTTTGCCAAGGTCTTTTAAGCACACCCAACGCCCCGGAGAGCCGGAGGAAGGTGCCCGGCATCAGCATCTCCGCCTCTCCTCCAGCTCTGGTCGTCTTCTCTACTCTGCAGCAGCAGAAGATTCTTCCTCTTCCACCAGCAGCGACAGCCTGGGTGGGGGTTACTGTGGGGCAAGGCCGGAGCCTGGGCTCCCTCACCCCCATCATCACATCCTGCAGCCCCGTCTGCCTCGAAAGGTGGACACGGCTGCACAGACCAACAGCCGCCTGGCCCGGCCTACACGGCTATCCCTGGGGGATCCCAAAGCCAGCACCTTACCTCGGGCTCGGGAGCAGCAACAACAGCCGCCCCCAATGCTCCCTCCAGAACCCAAGAGCCCAGGGGAATATGTGAATATTGAGTTTGGGAGTGATCAGCCTGGCTATTTATCTGGTCCCATGGCTTCCCGCAGTTCCCCTTCTGTCCGGTGTCCCTCACAGCTCCAGCCGGCTCCCAGAGAGGAAGAGACTGGCACCGAGGAGTACATGAACATGGACCTGGGACCTGGCCGGAGAGGGGTCTGGCAGGAAGGTGGTGGGTCTGAGATAGGCAGAGTGGGCCCTGCACCTCCAGGGGCCACTAGTGTTTGTAGGCCCACGCGGGCAGTGCCCAGCAGCCACAGCAGCCAGGGCGACTACATGACCATGCAGATGGGTTGTCCTCGTCAGAGCTATGTGGACACTTCACCGGTTGCCCCCGTCAGCTATGCTGACATTCGGACAGGCATTGCAGAGGAGGTGAGCCTGCCCAGGGCCACAGTGGCCGTTCTCTCTTCATCTGCAGCAGCCCCTGCTTCCCCCACGGCCCCCCAGGGAACAGCTGAGCTGGCTGCCCACTCTACCCTGCTGGGGGATCCACAGGGACCTGGGGGCATGAGCGCCTTCACCAGGGTGAACCTCAGTCCCAACCGCAACCAGAGTGCCAAAGTGATCCGCGCAGACCCACAAGGGTGTCGGAGGCGGCACAGCTCGGAAACCTTTTCCTCAACACCTACTGCTACCCGGGCGGGCAACACTGTGCCCTTTGGCGGGGGAGCTGCAGTAGGAGGCAGTGGTGGCAGCGGCAGCAGCAGTGAGGATGTGAAACGCCACAGTTCTGCTTCATTTGAGAATGTGTGGCTAAGACCTGGGGAGCTAGGGGGAGCCCCCAAGGACACTGCCCAAGTGTGTGGCGCGGCTGGGGGTTTGGAAAATGGTCTTAACTACATAGACCTGGATTTGGTCAAAAACTTGAAACAGCGCCCTCAGGAGCGCCCCCCTCAACTGCAGCCTGCCCTGCCCCCAGCCCCCCATAAGCCCCTGGGCAGCAGTGAGAGCAGCTCAACCAGCCGCTCCAGTGAGGATGTAAGCGCCTATGCCAGCATCAGTTTCCAGAAGCAGCCAGAGGACCGCCAGTAG

>Chi_la1 [Chinchilla (Chinchilla lanigera) Irs1]

ATGGCGAGCCCACCGGATACCGATGGCTTCTCGGACGTGCGCAAGGTTGGCTACCTGCGCAAACCCAAGAGCATGCACAAGCGCTTCTTCGTGCTGCGGGCAGCCAGCGAGGCCGGGGGCCCGGCGCGCCTGGAGTACTACGAGAACGAGAAGAAGTGGCGGCACAAGTCCAGCGCCCCCAAACGCTCTATCCCCCTGGAGAGCTGCTTCAACATCAACAAGCGGGCTGACTCCAAGAACAAGCACCTGGTGGCTCTCTACACCCGGGACGAGCACTTTGCCATTGCTGCGGATAGCGAGGCCGAGCAAGACAGCTGGTACCAGGCTCTTCTGCAGCTGCACAGCCGTGCCAAGGGCCACCACGACGGGGCCGCAGTCTCCGGAGCGGGAGGCGGCGGCGGGGGCAGCTGCAGCGGTAGCTCCGGCCTTGGTGAGGCCGGGGAGGACTTGAGCTATGGAGATGTGCCCCCAGGACCTGCGTTCAAGGAGGTCTGGCAGGTGATCCTGAAACCCAAGGGTTTGGGTCAAACAAAGAACCTGATTGGCATCTACCGCCTCTGCCTGACTAGCAAGACCATCAGCTTCGTGAAGCTGAACTCGGAGGCAGCGGCCGTGGTGCTGCAACTGATGAACATCAGGCGCTGTGGCCACTCGGAGAACTTCTTCTTCATTGAGGTGGGCCGTTCTGCAGTGACGGGGCCTGGGGAGTTCTGGATGCAGGTGGATGACTCTGTGGTGGCCCAGAACATGCACGAGACTATCCTGGAAGCCATGCGGGCTATGAGCGATGAGTTTCGCCCTCGCAGCAAGAGCCAGTCTTCATCCAACTGCTCCAACCCCATTAGCGTCCCCCTGCGCCGGCATCATCTCAACAACCCTCCGCCCAGCCAAGTGGGCCTGACCCGCCGATCTCGCACGGAGAGCATCACTGCCACTTCTCCAGCCAGCATGGTGGGCGGGAAGCCTGGTTCCTTCCGCGTGCGTGCCTCCAGCGATGGTGAAGGCACCATGTCCCGTCCAGCCTCGGTGGACGGCAGCCCTGTGAGCCCTAGTACCAACAGGACCCACGCCCACCGGCATCGGGGCAGCTCTCGGCTGCACCCCCCACTCAACCACAGCCGCTCCATCCCTATGCCTTCTTCGCGCTGCTCACCTTCGGCCACCAGCCCAGTGAGTCTGTCGTCCAGCAGCACCAGTGGCCATGGTTCCACCTCAGACTGTCTCTTTCCAAGGCGCTCTAGTGCTTCTGTTTCTGGCTCCCCCAGCGATGGCGGTTTCATCTCTTCCGATGAGTACGGCTCTAGTCCCTGTGATTTCCGAAGTTCTTTCCGCAGCGTCACCCCAGATTCCCTGGGCCACACCCCGCCAGCCCGTGGGGAGGAGGAGCTGAGCAACTACATCTGCATGGGTGGCAAGGGGGCTTCCACCCTGACGGCTCCCAATGGTCACTATATTTTGTCTCGGGGTGGCAATGGCCACCGCTATATCCCAGGAGCTGGCTTGGGTACCAGCCCAGCCTTGGCTGGGGATGAAGCAGCCAGTGCTGCAGATTTGGATAATCGGTTCCGGAAGAGGACTCACTCAGCAGGGGCATCCCCCACCATTTCCCACCAGAAGACTCCCTCGCAGTCCTCAGTGGCTTCCATTGAGGACTACACGGAGATGACGCCTGCCTGCCCACCAGGAGGTGGCAGTGGAGGCCGACTGCCCAGCTATCGGCACTCCGCCTTTGTGCCCACCCACTCCTACCCCGAGGAGGGTCTAGAGATGCACCCCCCAGAGCATCGTGGGGACCACCACCGCCCAGACACCTCCAGCCTCCACACTGACGACGGCTACATGCCCATGTCTCCAGGGGTGGCCCCAGTGCCTGGCAACCGAAAGGGCAGTGGGGACTATATGCCCATGAGTCCCAAGAGTGTGTCTGCCCCACAGCAGATCATCAACCCCATCAGACGCCATCCCCAGAGAGTGGACCCCAATGGCTACATGATGATGTCTCCCAGCGGCAGCTGCTCCCCTGACATCGGAGGCGGGTCGAGCAGCAGCAGTAGCATTAGTGCTGCCCCTTCCGGGGGTAGTTATGGGAAGCTGTGGACAAATGGAGTTGGAGGCCACCACTCTCACGCTCTGTCTCACCCCAAACCCCCTGTGGAGAGTGCTGGTGGCAAGCTCTTGCCTTGTACAAGTGACTACATGAATATGTCGCCAGTTGGGGACTCCAACACCAGCAGCCCCTCTGACTGCTATTATGGCCCTGAGGACCCCCAGCACAAGCCAGTCCTCTCCTATTACTCATTGCCAAGGTCTTTTAAGCACACCCAACGCCCCGGGGAGCTGGAGGAAGGTGCCCGGCATCAGCACCTCCGCCTCTCCTCCAGCTCTGGACGTCTTCTCTACGCTGCAGCAGCGGAAGATTCTTCCTCTTCCACCAGCAGCGACAGCCTGGGTGGGGGCTACTGTGGGGTGAGGCCGGAGCCTGGCCTCCCACATCCCCACCACCACGTCCTGCAGCCCCGTCTGCCCCGAAAGGTGGACACAGCTGCACAGACCAACAGCCGCCTGGCTCGGCCTACAAGGCTATCCCTGGGCGATCCCAAGGCCAGCACCTTACCTCGGGCTCGGGAGCAGCAGCCACAGCCGCCCCTGATGCACTCTCCAGAGCCCAAGAGCCCAGGGGAATACGTGAATATTGAATTTGGGAGTGAGCAACCTGGCTACTTATCTGGTCCCATGGCCTCCCACAGTTCCCCTTCTGTCCGGTGTCCCTCTCAGCTCCAGCTGGCTCCCAGGGAGAAGGAGACCGGCACCGAGGAGTACATGAACATGGACCTGGGGCCTGGCCGGAGGGCAGCCTGGCAGGCGGGTGGTGGGGCTGAGATGGGCAGAGTGGGCCCTGCACCTCCGGGGGCCGCTAGTGTTTGCAGACCTACCCGGGCGGTGCCCAGCAGCCAGGGTGACTACATGACCATGCAGATGGGTTGTTCTCGTCAGAGCTACGTGGACACCTCACCAGTCGCGCCTGTCAGCTACGCTGATATGCGGACAGGCATTGCAGAGGAGGTGAGCCTGCCCAGGGCCACGGTGGCCACTCTCTCTTCATCTGCAGCAGTCCCCGCTTCCCCCACGGGGCCTCAAGGAGCAGCTGAGCTGGCTGCCCACTCCTCCCTGCTCGGGGGCCCACAGGGACCTGGGGGCATGAGCGCCTTCACCAGGGTGAACCTCAGTCCCAACCACAACCAGAGTGCCAAAGTGATCCGCGCAGACCCACAAGGGTGTCGGAGGAGGCACAGCTCGGAAACCTTCTCCTCAACACCTACTGCCACCCGGGCGGGCAACGCGGTGCCCTTTGGAGGAGGGGCTGCTGTAGGGGGCAGCAGTGGTGGCAGCAGCAGCGAGGATGTGAAACGCCACAGTTCTGCATCGTTTGAGAACGTGTGGCTAAGACCTGGGGAGCTCGGGGGAGCCCCCAAGGACACTGCCCAAATGTGTGGGGCGGCCGGGGGTTTGGAAAATGGTCTTAACTACATAGACCTGGATTTGGTCAAAAACTTGAAACAGCGCCCTCAGGAGCGCCCCCCTCAGCTGCAGCCTCCCCTGCCCCCACCCCCTCATCAACCCCTGGGCGGCAGTGGGAGCAGCTCAACCAGCCGCTCCAGCGAGGATTTAAGCGCCTATGCCAGCATCAGTTTCCAGAAGAAGCCCGAGGACCGCCAGTAG

>Ory_cu1 [Rabbit (Oryctolagus cuniculus) Irs1]

ATGGCGAGCCCTCCGGAGACCGAAGGCTTCTCGGACGTGCGCAAGGTGGGCTACCTGCGCAAACCCAAGAGCATGCACAAGCGCTTTTTCGTGCTGCGGGCCGCCAGCGAGGCGGGGGGCCCGGCGCGCCTCGAGTACTACGAGAACGAGAAGAAGTGGCGGCACAAGTCGAGCGCCCCCAAACGCTCGATCCCCCTGGAGAGCTGCTTCAACATCAACAAGCGGGCTGACTCCAAGAACAAGCACCTGGTGGCTCTCTACACCCGGGACGAACACTTTGCCATTGCGGCAGACAGCGAGGCCGAGCAGGACAGCTGGTACCAGGCTCTCCTGCAGCTGCACAACCGCGCCAAGGGCCACCACGATGGGGCCTCGGCCACCGGGGCGGGAGGCGCTGGGGGCAGCTGCAGCGGCAGCTCTGGCCTGGGCGAGGCTGGAGAGGACTTGAGCTACGGGGACATGCCCCCAGGACCCGCCTTCAAGGAGGTCTGGCAGGTGATCTTGAAACCCAAAGGCCTGGGGCAGACAAAGAATTTGATCGGCATCTACCGCCTGTGCCTGACCAGCAAGACCATCAGCTTCGTGAAGCTGAACTCGGAGGCCGCGGCCGTGGTGCTGCAGCTGATGAACATCAGGCGCTGCGGCCACTCCGAGAACTTCTTCTTCATCGAGGTGGGCCGCTCCGCCGTGACGGGCCCCGGCGAGTTCTGGATGCAGGTGGATGACTCCGTGGTGGCCCAGAACATGCACGAGACCATCTTGGAGGCCATGCGGGCCATGAGCGACGAGTTCCGCCCTCGCAGCAAGAGCCAGTCCTCCTCCAACTGCTCCAACCCCATCAGCGTCCCCCTGCGCCGGCACCATCTCAACAACCCGCCGCCCAGCCAGGTGGGGCTGACGCGCCGCTCGCGCACCGAGAGCATCACCGCCACCTCCCCGGCCAGCATGGTGGGCGGGAAGCCAGGTTCCTTCCGGGTCCGCGCCTCCAGCGACGGCGAAGGCACCATGTCGCGCCCAGCCTCGGTGGACGGCAGCCCGGTGAGCCCCAGCACCAACAGGACCCACGCCCACCGGCATCGGGGCAGCTCCCGGCTGCACCCGCCGCTCAACCACAGCCGCTCCATACCCATGCCTTCTTCTCGCTGCTCGCCGTCGGCCACCAGCCCCGTCAGTTTGTCGTCCAGCAGCACCAGTGGCCACGGCTCCACCTCGGACTGTCTCTTCCCGCGGCGGTCGAGCGCCTCTGTGTCCGGCTCCCCCAGCGATGGTGGCTTCATCTCCTCCGACGAGTATGGCTCGAGTCCTTGCGACTTTCGAAGTTCCTTCCGCAGTGTCACTCCCGATTCCCTGGGCCACACCCCGCCAGCCCGCGGTGAGGAGGAGCTCAGTAACTACATCTGCATGGGCGGCAAAGGGGCCTCCACCCTGGCCGCCCCCAACGGTCACTACATTTTGGCTCGCGGAGGCAATGGCCACCGCTACATCCCCGGGGCGGGCCTGGGCGTGAGCCCCGCCCTGGCTGCGGAGGATGCAGCCAGCGCCGCCGACCTGGAAAATCGCTTCCGGAAGCGAACTCACTCGGCGGGCACCTCCCCTACCATTTCGCACCAGAAGACCCCATCGCAGTCCTCGGTGGAGTACACAGAGATGATGCCTGCCTACCCACCGGGAGGTGGCAGTGGAGGCCGACTGCCCGGCTACAGGCACTCCGCCTTCGTGCCCACCCACTCTTACCCCGAGGAGGGTCTGGAGATGCACCCCTTGGAGCGCCGCGGGGGCCACCACCGGCCAGACTCCTCCACTCTCCACACTGACGATGGCTACATGCCCATGTCCCCGGGGGTGGCCCCGGTGCCAGGGAGCAGCCGCAAGGGCAGTGGGGACTACATGCCCATGAGCCCCAAGAGTGTATCTGCCCCTCAACAGATCATCAACCCCATCAGACGCCACCCCCAGAGAGTGGACCCCAATGGCTACATGATGATGTCCCCCAGTGGCAGCTGCTCCCCTGACATTGGCGGTGGGCCCAGCAGCAGCAGTGGCAGCAGCGGCGTCAGCGCCGCCCCTTCCGGGAGCAGCTACGGCAAGCTGTGGACCAATGGGGTAGGGGGCCACCACTCTCATGCCCTGCCTCACCCCAAACCCCCTGCGGAGAGCAGTGGTGGCAAGCTCTTGCCTTGCACAGGTGAATACATGAACATGTCGCCAGTGGGGGACTCCAACACCAGCAGCCCCTCAGACTGCTACTATGGCCCTGAGGACCCCCAGCACAAGCCAGTCCTCTCCTACTACTCACTGCCAAGGTCCTTTAAGCACACCCAGCGGCCGGGGGAGCTGGAGGAAGGTGCCCGGCACCAGCACCTCCGCCTCTCCTCTAGCTCTGGTCGCCTTCTCTATGCTGCGACAGCTGAAGATTCTTCTTCTTCCACCAGCAGCGACAGCCTGGGTGGTGGATACTGTGGGGCTAGGCCAGAGCCTGGCCTCCCGCATCCCCACCATCATGTCCTACAGCCCCACCTGCCTCGAAAGGTGGACACAGCTGCTCAGACCAACAGCCGCCTGGCTCGGCCCACGAGGCTGTCCCTGGGGGATCCCAAGGCCAGTACCTTGCCTCGGGTTCGAGAGCAGCAGCAGCAGCAGCAGTCCCTGCCGCACCCTCCGGAGCCCAAGAGCCCAGGGGAGTATGTGAATATTGAATTTGGGAGTGATCAACCTGGCTACCTGTCTGGCCCTGTGGCTTCCCACAGCTCTCCCTCTATCAGGTGTCCACCCCAGCTCCAGCCAGCTCCCCGAGAGGAAGAGACTGGCACCGAGGAGTACATGAACATGGACCTGGGACCTGGCCGGAGGGCAACCTGGCAGGAGAACACAGGGGCTGACTTGGGCAGAGTGGGCCCTGCACCTCCTGGGGCAGCTAGCGTTTGCAGGCCCACCCGGGCAGTGCCCAGCAGCCGAGGCGACTACATGACCATGCAGATGGGTTGTTCCCGCCAGAGCTACGTGGATACCTCCCCAGTGGCCCCTGTCTGCTATGCTGACATGCGGGCAGGCATTGCTGCCGAGGAGGCCAGCCTGCCCCGGGCCACAGCGGCTGCTCCTTCCTCATCCTCAGCAGCCTCCTCTTCCCCCACTGAGCCTCAAGGTGCAGCTGAACTGGCTACTCGCTCCTCCCTGCTGGGAGGGCCACAAGGCCCCGGGGGCATGAGTGCCTTCACCCGGGTGACCCTCAGTCCGAACCGCAACCAGAGTGCCAAAGTGATCCGTGCAGACCCACAAGGGTGCCGGAGGAGGCATAGCTCAGAGACCTTCTCCTCTACACCCAGTGCCACCCGGGCGGGCAACACGGTGTCTTTCGGAGGGGGCGCTTCAGGAGGGGGCAGCGGTGGCAGCGGCAGTGAGGATGTGAAACGCCACAGCTCTGCTTCCTTTGAGAATGTGTGGCTGAGGCCCGGGGAGCTGGTGGGAGCCCCCAAGGAGACAGCCCAAGTGTGTGGGGCTGCTGGGGGTTTGGAGAATGGTCTTAACTACATAGACCTGGATTTGGTCAAGGACTTCAAACAGCGCCCTCAGGAGCGCCCCCCTCAGCCGCAGCCTCCCCAGCCTGTGCCCCCTCATCCTCATCAGCCCCTGGGCAGCGGTGAGAGCAGCTCCACCAGCCGCTCCAGTGAGGATTTAAGCGCCTATGCCAGCATCAGTTTCCAGAAGCAACCAGAGGACCGCCAGTAG

>Och_pr1 [Pika (Ochotona princeps) Irs1]

ATGGCGAGCCCTACGGAGACCGAAGGCTTTTCGGACGTGCGCAAGGTGGGCTACCTGCGCAAACCCAAGAGCATGCACAAGCGCTTCTTCGTGCTGCGGGCGGCCAGCGAGGCGGGGGGCCCCGCGCGCCTCGAGTACTACGAGAACGAAAAGAAGTGGCGGCACAAGTCGAGCGCCCCCAAACGCTCGATCCCCCTGGAGAGCTGCTTCAACATCAACAAGCGGGCTGACTCCAAAAATAAGCACCTGGTGGCTCTCTACACCCGGGACGAACACTTTGCCATTGCGGCCGACAGCGAGGCCGAGCAGGACAGCTGGTACCAGGCCCTCCTGCAGCTGCACAGCCGTGCCAAGGCCCACCACGATGGGGCCTCCGGCTCTGGGGCGGGAGGTGCCGGGGGCAGTTGCAGCGGCAGCTCTGGCCTGGGCGAGGCTGGGGAGGACCTGAGCTACGGAGACATGCCCCCGGCCTTCAAGGAGGTCTGGCAAGTGATCCTGAAACCCAAGGGTCTGGGTCAGACAAAGAATCTGATCGGCATCTACCGCCTGTGCCTCACCAGCAAGACCATCAGCTTCGTGAAGCTGAACTCGGAGGCCGCCGCTGTGGTGCTCCAGCTCATGAACATCCGACGTTGCGGCCACTCCGAGAACTTCTTCTTCATCGAGGTGGGCCGCTCCGCCGTGACTGGGCCAGGCGAGTTCTGGATGCAGGTGGATGACTCCGTGGTGGCCCAGAACATGCACGAGACCATTCTGGAGGCCATGAGGGCCATGAGCGACGAGTTCCGCCCTCGCAGCAAGAGCCAGTCCTCGTCCAACTGTTCCAACCCCATCAGCGTGCCCCTGCGCCGTCACCACCTCAACAACCCCCCGCCCAGCCAGGTGGGGCTTACGCGCCGCTCACGCACCGAGAGCATCACTGCCACCTCCCCAGCCAGCATGGTGGGCGGGAAACCAGGCTCCTTCCGGGTCCGCGCCTCCAGCGACGGCGAGGGCACTATGTCACGCCCGGCCTCAGTGGACGGCAGCCCCGTGAGCCCCAGCACCAACAGGACCCACGCCCACCGGCACCGGGGCAGCTCTCGGCTGCACCCACCGCTCAACCACAGCCGCTCCATACCCATGCCTTCTTCTCGCTGCTCGCCTTCAGCCACCAGTCCGGTCAGCCTGTCGTCCAGCAGTACCAGTGGCCATGGCTCCACCTCGGACTGCCTCTTTCCGCGGCGGTCGAGTGCCTCTGTGTCAGGTTCACCCAGCGATGGCGGCTTCATCTCTTCGGACGAGTATGGCTCCAGCCCTTGCGACTTCCGAAGTTCTTTCCGCAGTGTCACTCCGGATTCCTTGGGCCACACCCCGCCGGCCCGCAGCGAGGAGGAGCTCAGCAACTACATTTGCATGGGCGGCAAAGGGGCCTCCACCCTGGCCGCCCCCAATGGCCACTCTATTTTGCCTAGGGGAGGCAATGGCCACCGCTACATCCCCGGGGCGGGCCTGGGTACCAGCCCCGCCTTGGCTGCCTTGGCTGTGGATGAGGCAGCCGGGGCTGCGGACCTGGATAACCGTTTCCGAAAGAGAACCCACTCGGCGGGCACCTCCCCTACCATTTCTCACCAGAAGACCCCGTCGCAGTCCTCGGTGGCCTCCATTGAGGAGTACACGGAGATGATGCCAGCCTACCCACCAGGAGGTGGCAGTGGAGGCCGGCTACCCGGCTGCCGGCACTCCGCCTTTGTGCCCACCCACTCCTACCCGGAGGAGGGTCTGGAAATGCACTCTTTGGAGCGCCGTGGGCCCCCCCACCGGCAGGACACTGATGATGGCTACATGCCCATGTCCCCTGGTGTGGCCCCGGTGCCTGGTGGCCGCAAGGGCAGCGGAGACTACATGCCCATGAGCCCCAAGAGCGTGTCTGCTCCTCAGCAGATCATCAATGCCATCAGACGCCATCCCCAGCGAGTGGACCCTAATGGTTACATGATGATGTCCCCCAGTGGCAGCTGCTCCCCAGACGTTGCAGGTGGGCCCAGCAGCAGCAGCAGCAGCGGTGGTGGTGGAACCAGCAACATCAGTGCTACTGCTTCTGGGAGCAGCTATGGGAAGCTGTGGACCAATGGGGTGGCAGGCCACCACACCCATGCTCTGCCTTCCCCTAAACCCCCCGTGGAGAGCAGTGGTGGCAAGCTGTTACCTTGCACAGGTGAATACATGAACATGTCGCCAGTGGGTGACTCCAACACCAGCAGCCCCTCCGACTGCTACTACGGCCCCGAGGACACCCAGCACAAGCCAGCCCTCTCCTACTACTCGTTGCCAAGGTCCTTTAAGCACACCCAGCGTCCAGGGGAGCTGGAGGAAAGTGGCCGGCCCCAGCATCTCCGCCTTTCCTCCAGCTCTGGTCGCCTTCTCTACACTGCAACTGCTGAAGATTCTTCATCCTCCACCAGCAGTGACAGCCTGGGAGGCGGCTACTGTGGAGCCAGGCCGGACCCTGGCCTCCCACATCCCCACCATCACGTCTTGCAGCCCCACCTGCCCCGAAAGGTGGACACTGCTGCTCAGACCAACAGTCGCCTGGCACGGCCCACGAGGCTGTCCCTGGGGGATCCCAAGGCCAGCACCTTGCCTCGGGTCCGAGAGCAGCAACTGCAGCAGCAGCAAAAGCAGCAGCCCCTGCTGCTTCCTTCGGAGCCCAAGAGCCCAGGGGAATATGTGAATATTGAATTCGGGAGTGATCAGCCTGGCTACTTGTCAGGCCCTGTGGCTCCCCACAGCTCTCCCTCTATCAGGCCACAGCTCCAGCCAGCTCCCAGACAGGAGACGGCCAATGAGGAGTACATGAACATGGATCTGGGTCCTGGCCGGAGGGCCACTTGGCAGGAGAGTGCCGGGGCTGAGACAGGCAGAGTGGGCCCTGCCCCTCCTGGGGCTACTAGCGTCTGCAGGCCAACCCGGGCAGTGCCCAGCAGCCGAGGGGACTATATGACCATGCAGATGGGCTGTTCCCGCCAGAGCTATGTGGATACCTCCTCAGCTGCCCCTGTCTGCTACGCTGACATGCGGACAGGCATTGCTGCCCAGGAGGCCAGCCTGCCCCCGGCCGCTGTGGCTGCTCCTTCCCCAGCCTCGGCTGCCTCTTCTCCCTCTGCACCTCAAGGAGCCGCCGAAGTGGTGACCCGCTCTTCCCTGCTGGGGGCGCCTCAAGGAGCTGGGGGCGTGAGTGCCTTCACCCGGGTGACCCTGAGTCCCAGTCGCAACCAGAGTGCCAAAGTGATCCGTGCAGACCCACAGGGGTGCCGGAGGAGGCACAGCTCCGAGACCTTCTCCTCTACACCCAGTGCCACCCGGGCGGGCAACACTGTGTCCTTCGGAGGGGGTGCCGCAGGAGGGGGCAGTGGTGGTGGTGGCGGCAGCGAGGAGGTGAAACGCCACAGCTCTGCTTCCTTTGAGAATGTGTGGCTAAGGCCTGGGGAGCTCGTGGGAGCCCCCAGGGAGACAGGCCAAGTGTGTGGCCCTGCAGGGGCTGTGGAGAATGGTCTGAACTACATAGACCTGGATTTGGTCAAGGACGTCAAGCAGCGCCCTCAGCAGCGTCTCCCTCAGCCGCAGCCTCCTCCGCTCGCGCCCCCTCATCCTCACCAGCGCTCAGGCAGCAGCGAGAGCTGCTCCACCAGCCGCCCCAGTGAGGATTTAAGCACCTATGCCAGCATCAGTTTCCAGAAGCAGCCAGAGGACCGCCAGTAG

>Pan_ho1 [Chiru (Pantholops hodgsonii) Irs1]

ATGGCGAGCCCTCCGGAGACGGACGGCTTCTCGGACGTGCGCAAGGTGGGCTACCTGCGCAAACCCAAGAGCATGCACAAGCGATTCTTCGTGCTGCGGGCGGCCAGCGAGGCTGGGGGCCCGGCGCGCCTCGAGTACTACGAGAACGAAAAGAAGTGGCGGCACAAGTCGAGCGCCCCCAAACGCTCGATCCCCCTGGAGAGCTGCTTCAACATCAACAAGCGGGCGGACTCCAAGAACAAGCACCTGGTGGCCCTCTACACCCGGGACGAGCACTTTGCCATCGCGGCAGACAGTGAGGCCGAGCAGGACAGCTGGTACCAGGCCCTCCTGCAGCTGCACAACCGTGCCAAGGGCCACCACGACGGGGCCGCGGCGCCTGGGGCGGGAGGCGGCGGGGGCAGCTGCAGTGGCAGCTCCGGCCTCGGGGAGGCCGGGGAGGACTTGAGCTACGGGGACGTGCCCCCAGGACCCGCCTTCAAGGAGGTCTGGCAGGTGATCCTGAAACCCAAGGGCCTGGGTCAGACAAAGAACCTGATTGGCATCTACCGCCTCTGCCTGACCAGCAAGACCATCAGCTTCGTGAAGCTGAACTCGGAGGCGGCGGCCGTGGTGCTGCAGCTGATGAACATCAGGCGCTGTGGCCACTCAGAGAACTTCTTCTTCATCGAAGTGGGCCGTTCCGCAGTGACGGGCCCGGGGGAGTTCTGGATGCAGGTGGACGACTCCGTGGTGGCCCAGAACATGCATGAGACAATCCTGGAGGCCATGCGGGCCATGAGCGATGAGTTCCGCCCTAGAAGCAAGAGCCAGTCCTCCTCCAACTGCTCCAACCCCATTAGTGTGCCCCTGAGGAGGCACCACCTCAACAACCCTCCACCCAGCCAGGTGGGGCTGACCCGCCGCTCGCGCACCGAGAGCATCACGGCCACCTCCCCAGCCAGCTTGGTGGGCGGGAAGCAAGGCTCCTTCCGCGTGCGTGCCTCCAGTGATGGTGAAGGCACCATGTCTCGCCCTGCCTCAGTGGACGGCAGTCCTGTGAGTCCTAGCACCAACAGGACCCACGCCCACCGCCATCGAGGCAGCTCCCGGCTGCACCCGCCTCTCAATCACAGCCGCTCCATCCCCATGCCGTCTTCTCGCTGCTCGCCTTCCGCCACCAGCCCCGTCAGTCTGTCGTCCAGCAGCACGAGTGGCCACGGCTCCACCTCAGACTGCCTTTTCCCGCGGCGGTCTAGTGCTTCTGTGTCTGGTTCCCCCAGTGATGGCGGCTTCATCTCCTCCGATGAGTATGGCTCCAGTCCCTGCGATTTCCGAAGCTCCTTCCGCAGCGTCACCCCAGATTCCCTGGGCCACACCCCGCCCGCCCGGGGTGAGGAAGAGCTGAGCAACTACATCTGCATGGGAGGCAAGGGGGCCTCCACCCTCACTGCCCCCAATGGTCACTACATTTTGCCCCGGGGTGGCAATGGTCACCGTTACGTGCCAGCCGCAGGCTTGGGCACGAGCCCAGCCCTGCCAGGAGAAGAAGCAGCCGGTGCGGCAGATCTGGATAATCGGTTCCGAAAGCGGACTCACTCTGCGGGCACCTCGCCTACCATTTCCCACCAGAAAACCCCGTCTCAGTCCTCTGTGGCTTCCATTGAGGAATATACTGAAATGATGCCCGCCTACCCACCAGGAGGTGGCAGTGGAGGCCGAGTACCCAGCTACCGGCACTCCGCCTTCGTGCCCACCCATTCCTACCCAGAGGAGGGTCTGGAAATGCACCCCTTGGAGCGGCGTGTGGGCCACCACCGCCCAGACACCTCCAGCCTTCACACCGATGATGGCTACATGCCCATGTCCCCAGGGGTGGCCCCAGTGCCCGGCAGCCGGAAAGGCAGTGGGGACTACATGCCCATGAGCCCTAAGAGCGTGTCTGCCCCGCAGCAGATCATCAACCCCATCAGACGTCATCCCCAGAGAGTGGACCCCAACGGCTACATGATGATGTCCCCAAGCGGCAGCTGCTCTCCTGACATTGGAGGTGGGCCCAGCAGCGGTGGCAGCAGCAGCGGCGCCGCCCCTTCTGGGAGCAGCTATGGCAAGCTCTGGACAAACGGGGTAGGGGGCCACCACTCTCACGCCCCGCCACACCCCAAACTTCCGGCGGAGAGCGGTAGTGGCAAACTCTTGTCTTGTACAGGTGACTACATGAACATGTCGCCCGTTGGGGACTCCAACACCAGCAGCCCCTCCGACTGCTATTATGGCCCCGAGGACCCCCAGCACAAGCCCGTCCTCTCCTACTACTCATTGCCAAGGTCCTTTAAGCATACCCAGCGCCCGGGGGAGCTGGAGGAGCCCCGGCACCACCAGCACCTCCGCCTTTCCTCAAGTTCCGGTCGACTCCTCTACGCTGCGGCCGCAGAAGATTCCTCTTCGTCCACCAGCAGCGACAGCCTGGGCGGGGGATACTGTGGGGCTCGGCCAGAGCCTGGCCTCCCACATCTCCATCATCAGGTCCTTCAGGCCCATCTGCCTCGAAAGGTGGACACAGCTGCCCAGACCAACAACCGCCTGGCTCGGCCCACGAGGCTGTCTCTGGGGGATCCCAAGGCCAGCACTTTACCTCGGGCCCGAGAGCAGCAGCCGCCGCCCCCGTTGCTGCTCCCTCCGGAGCCTAAGAGCCCAGGGGAGTATGTGAATATTGAATTTGGGAGCGATCAGCCAGGCTACTTATCAGGCCCGGTGGCGTCCCACAGCTCGCCTTCCATCAGGTGTCCGTCCCAGCTCCAGCCAGCTCCCAGAGAGGAGGAGACGGGCGCAGAAGAGTATATGAACATGGACTTGGGGCCGGGCCGGAGGGCCACCTGGCAGGAGAGCGTGGGGGTCCAGCCGGGGAGGGTGGGCCCGGCGCCCCCTGGAGCTGCTAGCGTGTGTAGGCCGACTAGGGCAGTGCCCAGCAGCCGCGGCGACTATATGACCATGCAGATGGGCGGTCCCCGGCAGAGCTACGTGGACACCTCACCTGTCGCGCCCATCAGCTACGCCGACATGCGGACGGGTGTCGTGGAGGATGCCAGCCTGCCCGGGGCCACCGCGGACGCTCCCTCCTCATCCTCGACGGCCTCTGTTTCCTCCACTGCACCTCCGGGAACAGGGGAGCTGGCAGCCCGCTCGGCGCTGCTGGGGGGCCCGAGCGCCTTCACGCGGGTGAACCTCAGTCCCAACCGCAACCAGAGTGCCAAAGTGATCCGTGCCGACCCGCAAGGGTGCAGGAGGCGGCACAGCTCCGAGACCTTCTCCTCTACGCCTAGTGCCACACGGGTGGGCAACACGGTGCCCTTCGGAGCCGGGGCTGCCGTCGGGGGCAGCGGTGGCGGCAGCAGCAGCAGCGCGGAGGATGTCAAACGCCACAGCTCTGCTTCCTTCGAGAACGTGTGGCTGAGGCCTGGGGAGCTCGGGGGAGCCCCCAAGGAGCTGGCCCAAGTGTGCGGGGCCGCTGGGGGTTTGGAGAATGGTCTTAACTACATAGACTTGGATTTGGTCAAGGACTTCAAACAGCGCCCTCAAGAGCGCCCACCTCAACCGCAGCCTCCTCCACTCCCGGCCCCTCATCAGCCTCTGGGCAGTAGTCAGAGCGGCCCCACCAGCCGCTCCAGCGAGGATCTAAGCGCCTATGCCAGCATCACTTTCCAGAAGCAGCCAGAGGACCTCCAGTAG

>Bos_mu1 [Yak (Bos mutus) Irs1]

ATGGCGAGCCCTCCGGAGACTGACGGCTTCTCGGACGTGCGCAAGGTGGGCTACCTGCGCAAACCCAAGAGCATGCACAAACGATTCTTCGTGCTGCGGGCGGCCAGCGAGGCTGGGGGCCCGGCGCGCCTCGAGTACTACGAGAACGAGAAGAAGTGGCGGCACAAGTCGAGCGCCCCCAAACGCTCGATCCCCCTGGAGAGCTGCTTCAACATCAACAAGCGGGCGGACTCCAAGAACAAGCACCTGGTGGCCCTCTACACCCGGGACGAGCACTTTGCCATCGCGGCAGACAGCGAGGCCGAGCAGGACAGCTGGTACCAGGCCCTCCTGCAGCTGCACAACCGTGCCAAGGGCCACCACGACGGGGCCGCGGCGCCTGGGGCGGGAGGCGGCGGGGGCAGCTGCAGTGGCAGCTCCGGCCTCGGAGAGGCGGGTGAGGACTTGAGCTACGGGGACGTGCCCCCAGGACCCGCCTTCAAGGAGGTCTGGCAGGTGATCCTGAAACCCAAGGGCCTGGGTCAGACAAAGAACCTGATTGGCATCTACCGCCTCTGCCTGACCAGCAAGACCATCAGCTTCGTGAAGCTGAACTCGGAGGCGGCGGCCGTGGTGCTGCAGCTGATGAACATCAGGCGCTGCGGCCACTCAGAGAACTTCTTCTTCATCGAAGTGGGCCGTTCCGCAGTGACGGGCCCCGGGGAGTTCTGGATGCAGGTGGACGACTCCGTGGTGGCCCAGAACATGCACGAGACAATCCTGGAGGCCATGCGGGCCATGAGCGATGAGTTCCGCCCTAGAAGCAAGAGCCAGTCCTCCTCCAACTGCTCCAACCCCATCAGTGTGCCCCTGCGGAGGCACCACCTCAACAACCCTCCACCCAGCCAGGTGGGGCTGACCCGCCGCTCGCGCACCGAGAGCATCACAGCCACCTCCCCGGCCAGCTTGGTGGGCGGGAAGCAGGGCTCCTTCCGCGTGCGTGCCTCCAGTGATGGCGAAGGCACCATGTCTCGCCCCGCCTCAGTGGACGGCAGTCCTGTGAGTCCTAGCACCAACAGGACCCACGCCCACCGCCATCGAGGCAGCTCCCGGCTGCACCCGCCTCTCAACCACAGCCGCTCCATCCCCATGCCTTCTTCTCGCTGCTCGCCTTCCGCCACCAGCCCCGTCAGTCTGTCGTCCAGCAGCACGAGTGGCCATGGCTCCACCTCAGACTGCCTCTTCCCGCGGCGGTCTAGTGCTTCTGTGTCTGGTTCCCCCAGTGATGGCGGCTTCATCTCCTCCGATGAGTATGGCTCCAGTCCCTGCGATTTCCGAAGTTCCTTCCGCAGCGTCACCCCAGATTCCCTGGGCCACACCCCGCCCGCCCGGGGTGAGGAGGAGCTAAGCAACTACATCTGCATGGGAGGCAAGGGGGCCTCCACCCTCACTGCCCCCAATGGTCACTACATTTTGCCCCGGGGTGGCAATGGTCACCGCTACGTGCCAGCCGCCGGCTTGGGCACGAGCCCAGCCCTGCCAGGAGAAGAAGCAGCCGGTGCGGCAGATCTGGATAATCGGTTCCGAAAACGGACTCACTCTGCGGGCACCTCGCCTACCATTTCCCACCAGAAAACCCCGTCTCAGTCCTCTGTGGCTTCCATTGAGGAATATACCGAAATGATGCCCGCTTACCCACCAGGAGGTGGCAGTGGAGGCCGAGTACCCAGCTACCGGCACTCCGCCTTCGTGCCCACCCACTCCTACCCAGAGGAGGGTCTGGAAATGCACCCCTTGGAGCGGCGTGGGGGCCACCACCGCCCAGACACCTCCAGCCTTCACACTGATGATGGCTACATGCCCATGTCCCCAGGGGTGGCCCCAGTGCCCGGCAGCCGGAAAGGCAGTGGGGACTACATGCCCATGAGCCCCAAGAGCGTGTCTGCCCCGCAGCAGATCATCAACCCCATCAGACGCCATCCCCAGAGAGTGGACCCCAACGGCTACATGATGATGTCCCCAAGCGGCAGCTGCTCTCCTGACATTGGAGGTGGGCCCAGCAGCGGTGGCAGCAGCAGCGGTGCCGCCCCTTCTGGGAGCAGCTATGGCAAGCTTTGGACAAACGGGGTAGGGGGCCACCACTCTCACGCCCTGCCACACCCCAAACTTCCGGTGGAGAGCGGTAGTGGCAAACTCTTGTCTTGTACAGGTGACTACATGAACATGTCGCCCGTCGGGGACTCCAACACCAGCAGCCCCTCCGACTGCTATTATGGCCCCGAGGACCCCCAGCACAAGCCCGTCCTCTCCTACTACTCATTGCCAAGGTCCTTTAAGCACACCCAGCGTCCTGGGGAGCTGGAGGAGCCCCGGCACCACCAGCACCTCCGCCTTTCCTCAAGTTCCGGTCGACTCCTCTACACTGCGGCCGCAGAAGATTCCTCTTCGTCCACCAGCAGCGACAGCCTGGGCGGGGGATACTGTGGGGCTCGGCCGGAGCCCGGCCTCCCGCATCTCCATCATCAGGTCCTGCAGGCCCATCTGCCTCGAAAGGTGGACACAGCTGCCCAGACCAACAACCGCCTGGCTCGGCCCACGAGGCTGTCTCTGGGGGATCCCAAGGCCAGCACCTTACCTCGGGCCCGAGAGCAGCAGCCACCGCCCCCCTTGCTGCTCCCTCCGGAGCCCAAGAGCCCAGGGGAGTATGTGAATATTGAATTTGGGAGCGATCAGCCAGGCTACTTATCGGGCCCGGTGGCGTCCCACAGCTCGCCTTCCATCAGGTGTCCGTCCCAGCTCCAGCCAGCTCCCAGAGAGGAGGATACGGGCGCAGAAGAGTATATGAACATGGACCTGGGGCCGGGCCGGAGGGCCACCTGGCAGAAGAGCGTGGGGGTCCAGCCCGGCAGGGTGGGCCCGGCGCCCCCTGGAGCTGCTAGCGTGTGCAGGCCGACTAGGGCAGTGCCCAGCAGCTGCGGCGACTACATGACCATGCAGATGGGTGGTCCCCGGCAGAGCTACGTGGACACCTCACCTGTCGCGCCCATCAGCTACGCCGACATGCGGACGGGCGTCGTTGTGGAGAATGCCAGCTTGCCCGGGGCCACCGCGGCCGCTCCCTCCTCATCTTCGACAGCCTCTGTTTCCTCCACTGCGCCTCCTCCGGGAACAGGGGAGCTGGCGGCCCGCTCGGCCCTCCTGGGGGGCCCGAGCGCCTTCACGCGGGTGAACCTCAGTCCCAACCGCAACCAGAGTGCCAAAGTGATCCGTGCCGACCCTCAAGGGTGCAGGAGGCGGCACAGCTCCGAGACCTTCTCCTCTACGCCCAGTGCCACCCGGGTGGGCAACACGATGCCCTTCGGAGCTGGGGCTGCCGTCGGGGGCAGCGGTGGCGGCAGCAGCAGCAGCGCCGAGGATGTCAAACGCCACAGCTCTGCTTCCTTCGAGAACGTGTGGCTGAGGCCTGGGGAGCTCGGGGGAGCCCCCAAGGAGCTGGCCCAAGTGTGCGGGGCCGCTGGGGGTTTGGAGAATGGTCTTAACTACATAGACCTGGATTTGGTCAAGGACTTCAAACAGCGCCCTCAAGAGTGCCCACCTCAACCGCAGCCTCCTCCACTCCCGGCCCCTCATCAGCCTCTGGCCAGTAGTCAGAGCGGCCCCACCAGCCGCTCCAGCGAGGATCTAAGCGCCTATGCCAGCATCACTTTCCAGAAGCAGCCAGAGGACCTCCAGTAG

>Bub_bu1 [Water buffalo (Bubalus bubalis) Irs1]

ATGGCGAGCCCTCCGGAGACGGACGGCTTCTCGGACGTGCGCAAGGTGGGCTACCTGCGCAAACCCAAGAGCATGCACAAGCGATTCTTCGTGCTGCGGGCGGCCAGCGAGGCTGGGGGCCCTGCGCGCCTCGAGTACTACGAGAACGAGAAGAAGTGGCGGCACAAGTCGAGCGCCCCCAAACGCTCGATCCCCCTGGAGAGCTGCTTCAACATCAACAAGCGGGCGGACTCCAAGAACAAGCACCTGGTGGCCCTCTACACCCGGGACGAGCACTTTGCCATCGCGGCAGACAGCGAGGCCGAGCAGGACAGCTGGTACCAGGCCCTCCTGCAGCTGCACAACCGTGCCAAGGGCCACCACGACGGGGCCGCGGCGCCTGGGGCGGGAGGTGGCGGGGGCAGCTGCAGTGGCAGCTCCGGCCTCGGGGAGGCCGGTGAGGACTTGAGCTATGGGGACGTGCCCCCAGGACCCGCCTTCAAGGAGGTCTGGCAGGTGATCCTGAAACCCAAGGGCCTGGGTCAGACAAAGAACCTGATTGGCATCTACCGCCTCTGCCTGACCAGCAAGACCATCAGCTTCGTGAAGCTGAACTCGGAGGCGGCGGCCGTGGTGTTGCAGCTGATGAACATCAGGCGCTGCGGCCACTCAGAGAACTTCTTCTTCATCGAAGTGGGCCGTTCCGCAGTGACGGGCCCGGGGGAGTTCTGGATGCAGGTGGACGACTCCGTGGTGGCCCAGAACATGCACGAGACAATCCTGGAGGCCATGCGGGCCATGAGCGATGAGTTCCGCCCTAGAAGCAAGAGCCAGTCCTCCTCCAACTGCTCCAACCCCATCAGTGTGCCCCTGCGGAGGCACCACCTCAACAACCCTCCACCCAGCCAGGTGGGGCTGACCCGCCGCTCGCGCACCGAGAGCATCACAGCCACCTCCCCGGCCAGCTTGGTGGGCGGGAAGCAGGGCTCCTTCCGCGTGCGTGCCTCCAGTGATGGCGAAGGCACCATGTCTCGCCCCGCCTCAGTGGACGGCAGTCCTGTGAGTCCTAGCACCAACAGGACCCACGCCCACCGCCATCGAGGCAGCTCCCGGCTGCACCCGCCTCTCAACCACAGCCGCTCCATCCCCATGCCTTCTTCTCGCTGCTCGCCTTCTGCCACCAGCCCTGTCAGTCTGTCGTCCAGCAGCACGAGTGGCCATGGCTCCACCTCAGACTGCCTCTTCCCGCGGCGGTCTAGTGCTTCTGTGTCTGGTTCCCCCAGTGATGGCGGCTTCATCTCCTCCGATGAGTATGGCTCCAGTCCCTGCGATTTCCGAAGTTCCTTCCGCAGCGTCACCCCAGATTCCCTGGGCCACACCCCGCCCGCCCGGGGTGAGGAGGAGCTAAGCAACTATATCTGCATGGGAGGCAAGGGGGCCTCCACCCTCACTGCCCCCAATGGCCACTACATTCTGCCCCGGGGTGGCAATGGTCACCGCTACGTGCCAGCCGCCGGCTTGGGCACGAGCCCAGCCCTGTCAGGAGAAGAAGCAGCCGGTGCGGCAGATCTGGATAATCGGTTCCGAAAACGGACTCACTCTGCGGGCACCTCACCTACCATTTCCCACCAGAAAACCCCGTCTCAGTCCTCTGTGGCTTCCATTGAGGAATATACCGAAATGATGCCCGCTTACCCACCAGGAGGTGGCAGTGGAGGCCGAGTACCCGGCTACCGGCACTCCGCCTTCGTGCCCACCCACTCCTACCCAGAGGAGGGTCTGGAAATGCACCCCTTGGAGCGGCGTGGGGGCCACCACCGCCCAGACACCTCCAGCCTTCACACTGATGATGGCTACATGCCCATGTCCCCAGGAGTGGCCCCAGTGCCCGGCAGCCGGAAAGGCAGTGGGGACTACATGCCCATGAGCCCCAAGAGCGTGTCTGCCCCGCAACAGATCATCAACCCCATCAGACGCCATCCCCAGAGAGTGGACCCCAACGGCTACATGATGATGTCCCCAAGCGGCAGCTGCTCTCCTGACATTGGAGGTGGGCCCAGCAGCGGTGGCAGCAGCAGTGGTGCCGCCCCTTCTGGGAGCAGCTATGGCAAGCTTTGGACAAACGGGGTAGGGGGCCACCACTCTCACGCCCTGCCACACCCCAAACTTCCGGTGGAGAGCGGTAGTGGCAAACTCTTGTCTTGTGCAGGTGACTACATGAACATGTCGCCCGTCGGGGACTCCAACACCAGCAGCCCCTCCGACTGCTATTATGGCCCCGAGGACCCCCAGCACAAGCCCGTCCTCTCCTACTACTCATTGCCAAGGTCCTTTAAGCATACCCAGCGTCCTGGGGAGCTGGAGGAGCCCCGGCACCACCAGCACATCCGCCTTTCCACAAGTTCCGGTCGACTCCTCTACACTGCGGCCGCAGAAGATTCCTCTTCGTCCACCAGCAGCGACAGCCTGGGCGGGGGATACTGTGGGGCTCGGCCGGAGCCTGGCCTCCCGCATCTCCATCATCAGGTCCTGCAGGCCCATCTGCCTCGAAAGGTGGACACAGCTGCCCAGACCAACAACCGCCTGGCTCGGCCCACGAGGCTGTCTCTGGGGGATCCCAAGGCCAGCACCTTACCTCGGGCCCGAGAGCAGCAGCCGCCGCCCCCCTTGCTGCTCCCTCCGGAGCCCAAGAGCCCAGGGGAGTATGTGAATATTGAATTTGGGAGCGATCAGCCAGGCTACTTATCGGGCCCGGTGGCGTCCCACAGCTCGCCTTCCATCAGGTGTCCGTCCCAGCTCCAGCCAGCTCCCAGAGAGGAGGAGACGGGCGCAGAAGAGTATATGAACATGGACCTGGGGCCGGGCCGGAGGGCCACCTGGCAGGAGAGCGTGGGGGTCCAGCCGGGCAGGGTGGGCCCGGCGCCCCCTGGAGCTGCTAGCGTGTGTAGGCCGACTAGGGCAGTGCCCAGCAGCCGCGGCGACTACATGACCATGCAGATGGGCGGTCCCAGGCAGAGCTACGTGGACACCTCACCTGTCGCGCCCATCAGCTACGCCGACATGCGGACCGGCGTCGTTGTGGAGGATGCCAGCTTGCCCGGGGCCACTGCGGCCACTCCCTCCTCATCCTCGACGGCCTCTGTTTCCTCCACTGCGCCTCCGGGAACAGGAGAGCTGGCGGCCCGTTCGGCCCTCCTGGGGGGCCCGAGCGCCTTCACGCGGGTGAACCTCAGTCCCAACCGCAACCAGAGTGCCAAAGTGATCCGTGCCGACCCTCAAGGGTGCAGGAGGCGGCACAGCTCCGAGACCTTCTCCTCTACGCCCAGTGCCACCCGGGTGGGCAACACGATGCCCTTCGGAGCCGGGGCTGCCGTTGGGGGCAGCGGTGGCGGCAGTAGCAGCAGCGCTGAGGATGTCAAACGCCACAGCTCTGCTTCCTTCGAGAACGTGTGGCTGAGGCCTGGGGAGCTCGGGGGAGCCCCCAAGGAACTGGCCCAAGTGTGCGGGGCCGCTGGGGGTTTGGAGAATGGTCTTAACTACATAGACCTGGATTTGGTCAAGGACTTCAAACAGCGCCTTCAAGAGCGCCCACCTCAACCGCAGCCTCCTGCACTCCCGGCCCCTCATCAGCCTCTGGGCAGTAGTCAGAGCGGCCCCACCAGCCGCTCCAGCGAGGATCTAAGCGCCTATGCCAGCATCACTTTCCAGAAGCAGCCAGAGGACCTCCAGTAG

>Bos_ta1 [Cow (Bos taurus) Irs1]

ATGGCGAGCCCTCCGGAGACTGACGGCTTCTCGGACGTGCGCAAGGTGGGCTACCTGCGCAAACCCAAGAGCATGCACAAACGATTCTTCGTGCTGCGGGCGGCCAGCGAGGCTGGGGGCCCGGCGCGCCTCGAGTACTACGAGAACGAGAAGAAGTGGCGGCACAAGTCTAGCGCCCCCAAACGCTCGATCCCCCTGGAGAGCTGCTTCAACATCAACAAGCGGGCGGACTCCAAGAACAAGCACCTGGTGGCCCTCTACACCCGGGACGAGCACTTTGCCATCGCGGCAGACAGCGAGGCCGAGCAGGACAGCTGGTACCAGGCCCTCCTGCAGCTGCACAACCGTGCCAAGGGCCACCACGACGGGGCCGCGGCGCCTGGGGCGGGAGGCGGCGGGGGCAGCTGCAGTGGCAGCTCCGGCCTCGGGGAGGCCGGTGAGGACTTGAGCTACGGGGACGTGCCCCCAGGACCCGCCTTCAAGGAGGTCTGGCAGGTGATCCTGAAACCCAAGGGCCTGGGTCAGACAAAGAACCTGATTGGCATCTACCGCCTCTGCCTGACCAGCAAGACCATCAGCTTCGTGAAGCTGAACTCGGAGGCTGCGGCCGTGGTGCTGCAGCTGATGAACATCAGGCGCTGCGGCCACTCAGAGAACTTCTTCTTCATCGAAGTGGGCCGTTCCGCAGTGACGGGCCCCGGGGAGTTCTGGATGCAGGTGGACGACTCCGTGGTGGCCCAGAACATGCACGAGACAATCCTGGAGGCCATGCGGGCCATGAGCGATGAGTTCCGCCCTAGAAGCAAGAGCCAGTCCTCCTCCAACTGCTCCAACCCCATCAGTGTGCCCCTGCGGAGGCACCACCTCAACAACCCTCCACCCAGCCAGGTGGGGCTGACCCGCCGCTCGCGCACCGAGAGCATCACAGCCACCTCCCCGGCCAGCTTGGTGGGCGGGAAGCAGGGCTCCTTCCGCGTGCGTGCCTCCAGTGATGGCGAAGGCACCATGTCTCGCCCCGCCTCAGTGGACGGCAGTCCTGTGAGTCCTAGCACCAACAGGACCCACGCCCACCGCCATCGAGGCAGCTCCCGGCTGCACCCGCCTCTCAACCACAGCCGCTCCATCCCCATGCCTTCTTCTCGCTGCTCGCCTTCCGCCACCAGCCCCGTCAGTCTGTCGTCCAGCAGCACGAGTGGCCATGGCTCCACCTCAGACTGCCTCTTCCCGCGGCGGTCTAGTGCTTCTGTGTCTGGTTCCCCCAGTGATGGCGGCTTCATCTCCTCCGATGAGTATGGCTCCAGTCCCTGCGATTTCCGAAGTTCCTTCCGCAGCGTCACCCCAGATTCCCTGGGCCACACCCCGCCCGCCCGGGGTGAGGAGGAGCTAAGCAACTACATCTGCATGGGAGGCAAGGGGGCCTCCACCCTCACTGCCCCCAATGGTCACTACATTTTGCCCCGGGGTGGCAATGGTCACCGCTACGTGCCAGCCGCCGGCTTGGGCACGAGCCCAGCCCTGCCAGGAGAAGAAGCAGCCGGTGCGGCAGATCTGGATAATCGGTTCCGAAAACGGACTCACTCTGCGGGCACTTCGCCTACCATTTCCCACCAGAAAACCCCGTCTCAGTCCTCTGTGGCTTCCATTGAGGAATATACCGAAATGATGCCCGCTTACCCACCAGGAGGTGGCAGTGGAGGCCGAGTACCCAGCTACCGGCACTCCGCCTTCGTGCCCACCCACTCCTACCCAGAGGAGGGTCTGGAAATGCACCCCTTGGAGCGGCGTGGGGGCCACCACCGCCCAGACACCTCCAGCCTTCACACTGATGATGGCTACATGCCCATGTCCCCAGGGGTGGCCCCAGTGCCCGGCAGCCGGAAAGGCAGTGGGGACTACATGCCCATGAGCCCCAAGAGCGTGTCTGCCCCGCAGCAGATCATCAACCCCATCAGACGCCATCCCCAGAGAGTGGACCCCAACGGCTACATGATGATGTCCCCAAGCGGCAGCTGCTCTCCTGACATTGGAGGTGGGCCCAGCAGCGGTGGCAGCAGCAGCGGTGCCGCCCCTTCTGGGAGCAGCTATGGCAAGCTTTGGACAAACGGGGTAGGGGGCCACCACTCTCACGCCCTGCCACACCCCAAACTTCCGGTGGAGAGCGGTAGTGGCAAACTCTTGTCTTGTACAGGTGACTACATGAACATGTCGCCCGTCGGGGACTCCAACACCAGCAGCCCCTCCGACTGCTATTATGGCCCCGAGGACCCCCAGCACAAGCCCGTCCTCTCCTACTACTCATTGCCAAGGTCCTTTAAGCACACCCAGCGTCCTGGGGAGCTGGAGGAGCCCCGGCACCACCAGCACCTCCGCCTTTCCTCAAGTTCCGGTCGACTCCTCTACACTGCGGCCGCAGAAGATTCCTCTTCGTCCACCAGCAGCGACAGCCTGGGCGGGGGATACTGTGGGGCTCGGCCGGAGCCCGGCCTCCCGCATCTCCATCATCAGGTCCTGCAGGCCCATCTGCCTCGAAAGGTGGACACAGCTGCCCAGACCAACAACCGCCTGGCTCGGCCCACGAGGCTGTCTCTGGGGGATCCCAAGGCCAGCACCTTACCTCGGGCCCGAGAGCAGCAGCCACCGCCCCCCTTGCTGCTCCCTCCGGAGCCCAAGAGCCCAGGGGAGTATGTGAATATTGAATTTGGGAGCGATCAGCCAGGCTACTTATCGGGCCCGGTGGCGTCCCACAGCTCGCCTTCCATCAGGTGTCCGTCCCAGCTCCAGCCAGCTCCCAGAGAGGAGGATACGGGCGCAGAAGAGTATATGAACATGGACCTGGGGCCGGGCCGGAGGGCCACCTGGCAGAAGAGCGTGGGGGTCCAGCCCGGCAGGGTGGGCCCGGCGCCCCCTGGAGCTGCTAGCGTGTGCAGGCCGACTAGGGCAGTGCCCAGCAGCTGCGGCGACTACATGACCATGCAGATGGGTGGTCCCCGGCAGAGCTACGTGGACACCTCACCTGTCGCGCCCATCAGCTACGCCGACATGCGGACGGGCGTCGTTGTGGAGGATGCCAGCTTGCCCAGGGCCACCGCGGCCGCTCCCTCCTCATCTTCGACAGCCTCTGTTTCCTCCACTGCGCCTCCTCCGGGAACAGGGGAGCTGGCGGCCCGCTCGGCCCTCCTGGGGGGCCCGAGCGCCTTCACGCGGGTGAACCTCAGTCCCAACCGCAACCAGAGTGCCAAAGTGATCCGTGCCGACCCTCAAGGGTGCAGGAGGCGGCACAGCTCCGAGACCTTCTCCTCTACGCCCAGTGCCACCCGGGTGGGCAACACGATGCCCTTCGGAGCTGGGGCTGCCGTCGGGGGCAGCGGTGGCGGCAGCAGCAGCAGCGCCGAGGATGTCAAACGCCACAGCTCTGCTTCCTTCGAGAACGTGTGGCTGAGGCCTGGGGAGCTCGGGGGAGCCCCCAAGGAGCTGGCCCAAGTGTGCGGGGCCGCTGGGGGTTTGGAGAATGGTCTTAACTACATAGACCTGGATTTGGTCAAGGACTTCAAACAGCGCCCTCAAGAGTGCCCACCTCAACCGCAGCCTCCTCCACTCCCGGCCCCTCATCAGCCTCTGGCCAGTAGTCAGAGCGGCCCCACCAGCCGCTCCAGCGAGGATCTAAGCGCCTATGCCAGCATCACTTTCCAGAAGCAGCCAGAGGACCTCCAGTAG

>Ovi_arm1 [Mouflon sheep (Ovis aries musimon) Irs1]

ATGGCGAGCCCTCCGGAGACGGACGGCTTCTCGGACGTGCGCAAGGTGGGCTACCTGCGCAAACCCAAGAGCATGCACAAGCGATTCTTCGTGCTGCGGGCGGCCAGCGAGGCTGGGGGTCCGGCGCGCCTCGAGTACTACGAGAACGAAAAGAAGTGGCGGCACAAGTCGAGCGCCCCCAAACGCTCGATCCCCCTGGAGAGCTGCTTCAACATCAACAAGCGGGCGGACTCCAAGAACAAGCACCTGGTGGCCCTCTACACCCGGGACGAGCACTTTGCCATCGCGGCAGACAGCGAGGCCGAGCAGGACAGCTGGTACCAGGCCCTCCTGCAGCTGCACAACCGTGCCAAGGGCCACCACGACGGGGCCGCGGCGCCTGGGGCGGGAGGCGGCGGGGGCAGCTGCAGTGGCAGCTCCGGCCTCGGGGAGGCCGGGGAGGACTTGAGCTACGGGGACGTGCCCCCAGGACCCGCCTTCAAGGAGGTCTGGCAGGTGATCCTGAAACCCAAGGGCCTGGGTCAGACAAAGAACCTGATTGGCATCTACCGCCTCTGCCTGACCAGCAAGACCATCAGCTTCGTGAAGCTGAACTCGGAGGCGGCGGCCGTGGTGCTGCAGCTGATGAACATCAGGCGCTGTGGCCACTCAGAGAACTTCTTCTTCATCGAAGTGGGCCGTTCCGCAGTGACGGGCCCGGGGGAGTTCTGGATGCAGGTGGACGACTCCGTGGTGGCCCAGAACATGCATGAGACAATCCTGGAGGCCATGCGGGCCATGAGCGATGAGTTCCGCCCTAGAAGCAAGAGCCAGTCCTCCTCCAACTGCTCCAACCCCATTAGTGTGCCCCTGAGGAGGCACCACCTCAACAACCCTCCACCCAGCCAGGTGGGGCTGACCCGCCGCTCGCGCACCGAGAGCATCACGGCCACCTCCCCAGCCAGCTTGGTGGGCGGGAAGCAGGGCTCCTTCCGCGTGCGTGCCTCCAGTGATGGCGAAGGCACCATGTCTCGCCCTGCCTCAGTGGACGGCAGTCCTGTGAGTCCTAGCACCAACAGGACCCACGCCCACCGCCATCGAGGCAGCTCCCGGCTGCACCCGCCTCTCAATCACAGCCGCTCCATCCCCATGCCTTCTTCTCGCTGCTCGCCTTCCGCCACCAGCCCCGTCAGTCTGTCGTCCAGCAGCACGAGTGGCCACGGCTCCACCTCAGACTGCCTTTTCCCGCGGCGGTCTAGTGCTTCTGTGTCTGGTTCCCCCAGTGATGGCGGCTTCATCTCCTCCGATGAGTATGGCTCCAGTCCCTGCGATTTCCGAAGCTCCTTCCGCAGCGTCACCCCAGATTCCCTGGGCCACACCCCGCCCGCCCGGGGTGAGGAAGAGCTGAGCAACTACATCTGCATGGGAGGCAAGGGGGCCTCCACCCTCACTGCCCCCAGTGGTCACTACATTCTGCCCCGGGGTGGCAATGGTCACCGCTACGTGCCAGCCGCAGGCTTGGGCACGAGCCCCGCCCTGCCAGGAGAAGAAGCAGCCGGTGCGGCAGATCTGGATAATCGGTTCCGAAAGCGGACTCATTCTGCGGGCACCTCGCCTACCATTTCCCACCAGAAAACGCCGTCTCAGTCCTCTGTGGCTTCCATTGAGGAATATACTGAAATGATGCCCGCCTACCCACCAGGAGGTGGCAGTGGAGGCCGAGTACCCAGCTACCGGCACTCTGCCTTCGTGCCCACCCACTCCTACCCAGAGGAGGGTCTGGAAATGCACCCCTTGGAGCGGCGTGTGGGCCACCACCGCCCAGACACCTCCAGCCTTCACACCGATGATGGCTACATGCCCATGTCCCCAGGGGTGGCCCCAGTGCCCGGCAGCCGGAAAGGCAGTGGGGACTACATGCCCATGAGCCCTAAGAGCGTGTCTGCCCCGCAGCAGATCATCAACCCCATTAGACGTCATCCCCAGAGAGTGGACCCCAACGGCTACATGATGATGTCCCCGAGCGGCAGCTGCTCTCCTGACATTGGAGGTGGGCCCAGCAGCGGCGGCAGCAGCAGCGGCGCCGCCCCTTCTGGGAGCAGCTATGGCAAGCTCTGGACAAACGGGGTAGGGGGCCACCACTCTCACGCCCTGCCACACCCCAAACTTCCGGTGGAGAGCGGTAGTGGCAAACTCCTGTCTTGTACAGGTGACTACATGAACATGTCGCCCGTTGGGGACTCCAACACCAGCAGCCCCTCCGACTGCTATTATGGCCCCGAAGACCCCCAGCACAAGCCCGTCCTCTCCTACTACTCATTGCCAAGGTCCTTCAAGCATACCCAGCGCCCTGGGGAGCTGGAGGAGCCCCGGCACCACCAGCACCTCCGCCTTTCCTCAAGTTCCGGTCGACTCCTCTACGCTGCGGCCGCAGAAGATTCCTCTTCGTCCACCAGCAGCGACAGCCTGGGCGGGGGATACTGTGGGGCTCGGCCAGAGCCTGGCCTCCCACATCTCCATCATCAGGTCCTTCAGGCCCATCTGCCTCGAAAGGTGGACACAGCTGCCCAGACCAACAACCGCCTGGCTCGGCCCACGAGGCTGTCTCTGGGGGATCCCAAGGCCAGCACTTTACCTCGGGCCCGAGAGCAGCAGCAGCCGCCCCCGTTGCTGCTCCCTCCGGAGCCTAAGAGCCCAGGGGAGTATGTGAATATTGAATTTGGGAGCGATCAGCCAGGCTACTTATCAGGCCCGGTGGCGTCCCACAGCTCGCCTTCCATCAGGTGTCCGTCCCAGCTCCAGCCAGCTCCCAGAGAGGAGGAGACGGGCGCAGAAGAGTATATGAACATGGACCTGGGGCCGGGCCGGAGGGCCACCTGGCAGGAGAGCGTGGGGGTCCAGCCGGGCAGGGTGGGCCCGGCGCCCCCTGGAGCTGCTAGCGTGTGCAGGCCGACTAGGGCAGTGCCCAGCAGCCGCGGCGACTATATGACCATGCAGATGGGCGGTCCCCGGCAGAGCTACGTGGACACCTCACCTGTCGCGCCCATCAGCTACGCCGACATGCGGACGGGTGTCGTGGAGGATGCCAGCCTGCCCGGGGCCACCGCGGACGCTCCCTCCTCATCCTCGACGGCCTCTGTTTCCTCCACTGCACCTCCGGGAACAGGGGAGCTGGCGGCCCGCTCGGCCCTGCTGGGGGGCCCGAGCGCCTTCACGCGGGTGAACCTCAGTCCCAACCGCAACCAGAGTGCCAAAGTGATCCGTGCCGACCCGCAAGGGTGCAGGAGGCGGCACAGCTCTGAGACCTTCTCCTCTACGCCCAGTGCCACACGGGTGGGCAACACGGTGCCCTTCGGAGCCGGGGCTGCCGTCGCGGGCAGCGGTGGCGGCAGCAGCAGCAGCGCTGAGGATGTCAAACGCCACAGCTCTGCTTCCTTCGAGAACGTGTGGCTGAGGCCTGGGGAGCTTGGGGGAGCCCCCAAGGAGCTGGCCCAACTGTGCGGGGCCGCTGGGGGTTTGGAGAATGGTCTTAACTACATAGACTTGGATTTGGTCAAGGACTTCAAACAGCGCCCTCAAGAGCGCCCACCTCAACCGCAGCCTCCTCCACTCCCGGCCCCTCATCAGCCTCTGGGCAGTAGTCAGAGCGGCCCCACCAGCCGCTCCAGCGAGGATCTAAGCGCCTATGCCAGCATCACTTTCCAGAAGCAGCCAGAGGACCTCCAGTAG

>Orc_or1 [Killer whale (Orcinus orca) Irs1]

ATGGCGAGCCCTCCCGAGACCGACGGCTTCTCCGACGTGCGCAAGGTGGGCTACCTGCGCAAGCCCAAGAGCATGCACAAGCGATTCTTCGTGCTGCGGGCGGCCAGCGAGGCTGGGGGCCCGGCGCGCCTCGAGTACTACGAGAACGAGAAGAAGTGGCGGCACAAGTCGAGCGCCCCCAAACGCTCGATCCCCCTCGAGAGCTGCTTCAACATCAACAAGCGGGCCGACTCCAAGAACAAGCACCTGGTGGCCCTCTATACCCGGGACGAGCACTTTGCCATCGCGGCGGACAGCGAGGCCGAGCAGGACAGCTGGTACCAGGCCCTCCTGCAGCTGCACAACCGTGCCAAGGGCCACCACGACGGGGCCGCGGCTCCCGGGGCGGGAGGCGGCGGGGGCAGCTGCAGTGGCAGCTCCGGCCTTGGCGAGGCTGGGGAGGACTTGAGCTACGGGGACGTGCCCCCAGGACCCGCCTTCAAGGAAGTCTGGCAGGTGATCCTGAAACCCAAGGGCCTGGGTCAGACAAAGAACCTGATTGGTATCTACCGCCTCTGCCTGACCAGCAAGACCATCAGCTTCGTGAAGCTGAACTCGGAGGCTGCCGCGGTGGTGCTGCAGCTGATGAACATCAGGCGCTGTGGCCACTCAGAGAACTTCTTCTTCATCGAAGTGGGCCGTTCCGCAGTGACGGGACCCGGGGAGTTCTGGATGCAGGTGGATGACTCTGTGGTGGCCCAGAACATGCACGAGACGATCCTGGAAGCCATGCGGGCCATGAGCGATGAGTTCCGCCCTCGAAGCAAGAGCCAGTCCTCCTCCAACTGCTCCAACCCCATCAGTGTCCCCCTGCGCAGGCATCACCTCAACAACCCTCCACCCAGCCAGGTGGGGCTGACCCGCCGCTCGCGCACAGAGAGCATCACTGCCACCTCCCCGGCCAGCTTGGTGGGCGGGAAGCAGGGCTCCTTCCGGGTCCGCGCCTCTAGTGATGGCGAAGGCACCATGTCTCGCCCTGCCTCTGTGGACGGCAGTCCTGTGAGTCCCAGCACCAACAGGACCCATGCCCACCGGCATCGCGGCAGTTCCCGGCTGCACCCTCCTCTCAACCACAGCCGCTCCATCCCCATGCCTTCTTCTCGCTGCTCGCCTTCCGCCACCAGCCCGGTCAGCCTATCGTCCAGTAGCACCAGTGGCCACGGCTCCACCTCGGACTGTCTCTTCCCACGGCGGTCTAGTGCTTCCGTGTCCGGTTCCCCCAGTGATGGCGGTTTCATCTCCTCTGATGAGTATGGCTCCAGTCCCTGCGACTTCCGAAGTTCCTTCCGCAGTGTCACCCCGGATTCCCTGGGCCACACCCCGCCGGCCCGCGGTGAGGAGGAGCTGAGCAACTACATCTGCATGGGAGGCAAGGGGGCCTCCACCCTCACGGCCCCCAATGGTCACTACATTTTGCCTCGGAGTGGCAACGGTCCCCGCTACATCCCGGCAGCCGGCTTGGGCACGAGCCCAGCCCTGACAGGGGATGAAGCAGCCAGTGCTGCAGATCTGGACAATCGGTTCCGAAAGCGGACTCACTCTGCTGGCACGTCACCTACCATTTCCCACCAGAAGACCCCATCCCAGTCCTCTGTGGCCTCCATTGAGGAATATACCGAGATGATGCCTGCCTACCCACCAGGAGGTGGCAGTGGAGGCCGAGTGCCCAGCTACCGGCACTCCGCCTTCGTGCCCACCCACTCCTACCCAGAGGAGGGTCTGGAAATGCACCCCTTGGAGCGGCGTGTGGGCCACCACCGCCCAGACACCTCCAACCTCCACACCGATGATGGCTACATGCCCATGTCCCCAGGGGTGGCCCCAGTGCCCGGGAGCCGAAAGGGCAGTGGGGACTACATGCCCATGAGCCCCAAGAGCGTGTCTGCCCCTCAGCAGATCATCAACCCCATCAGACGCCATCCCCAGAGAGTGGACCCCAATGGCTACATGATGATGTCCCCAAGCGGCAGCTGCTCTCCTGACATTGGAGGCGGGCCCAGCAGCGGCGGCAGCAGCGGTGCCGCCCCTTCTGGGAGCAGCTATGGCAAGCTCTGGACGAATGGGGTAGGGGGCCACCACTCTCACGCCCTGCCACACCCCAAACTCCCTGTGGAGAGCAGTAGCGGCAAGCTGTTGTCTTGTACGGGTGACTACATGAACATGTCGCCAGTGGGGGACTCCAACACCAGCAGCCCTTCCGACTGCTACTGTGGGCCTGAGGACCCCCAGCACAAGACAGTCCTCTCCTACTACTCATTGCCAAGGTCCTTTAAGCACACCCAGCGCCCCGGGGAGCTGGAGGAGGGCGCCCGGCACCAGCACCTCCGCCTTTCCTCCAGCTCGGGTCGACTTCTCTATGCTGCGGCGGCGGAAGATTCCTCCTCGTCCACCAGCAGCGACAGCCTGGGCGGGGGATACTGCGCGGCTAGGCCCGAGCCCGGCCTCCCGCATCTCCACCATCAGGTCCTGCAGCCCCATCTGCCTCGAAAGGTGGACACAGCTGCCCAGACCAACAGCCGCCTGGCCCGGCCCACGAGGCTGTCCCTGGGGGATCCCAAGGCCAGCACCTTACCCCGGGCCCGAGAGCAGCCGCAGCCCCAGCCGCAGCCGCCGCCGCCCCTGCTGCCCCCTCCGGAGCCCAAGAGCCCAGGGGAATATGTGAATATTGAATTTGGGAGCGATCAGCCGGGCTACTTATCAGGCCCCGGGGCTTCCCACAGCTCGCCTTCTGTCCGGTGTCCATCCCAGCTCCAGCCGGCTCCCAGAGAGGAGGAGACGGGCGCGGAAGAGTACATGAACATGGACCTGGGGCCGGGCCGGAGGGCCGCCTGGCGGGAGAGCGCTGGGGTCCAGCCGGGCAGCATGGGCCCGGCACCCCCTGGAGCTGCTAGCGTGTGCAGGCCCACCAGGGCAGTGCCCAGTAGCCGGGGTGACTACATGACCATGCAGATGGGCTGTCCCCGGCAGAGCTACGTGGACACCTCGCCAGTCGCCCCTATCAGCTATGCGGACATGCGGACCGGCGGCGGTGGCCTCGTGGAGGAGGCCAGCCTGCCTGGGGCCGCCGCGGCCACGCCCTCCTCATCCTCGAAGGCCTCTGCTTCCCCCGCCGCGCCTCAAGGAGCAGAGGAGCTGGCGGCCCGCTCTTCCCTGCTGGGAGGCCCGCAGGGACCCGGGGGCTCGAGTGCCTTCACGCGGGTGAACCTCAGTCCCAACCGCAACCAGAGTGCCAAAGTGATCCGTGCCGACCCGCAAGGGTGCAGGAGGCGGCACAGCTCCGAGACCTTCTCCTCGACGCCCAGTGCCACGCGGGCAGGCAACACGGTGCCCTTCGGAGCGGGGGCTGCAGCCGGGGGCGGCGGTGGTGGCAGCAGCAGCACGGAGGACGTGAAACGCCACAGCTCTGCTTCCTTTGAGAACGTGTGGCTGCGGCCTGGGGAGCTCGGGGGAGCCCCCAAGGAGCTGGCCCAAGTGTGCGGGGCCGCCGGGGGTTTGGAGAATGGGCTTAACTACATAGACCTGGATTTGGTCAAGGACTTCAACCAGAGCCCTCAAGAGCGCCCCGCTCAGCCGCAGCCTTCTCCTCCCCCGCCCCCTCATCAGCCTCTGGGACGCAGCGAGAGCGGCTCCACCAGCCGCTCCAGCGAGGATTTAAGCGCCTATGCCAGCATCAGTTTCCAGAAGCCGCCAGAGGACCTCCAGTAG

>Lip_ve1 [Yangtze River dolphin (Lipotes vexillifer) Irs1]

ATGGCGAGCCCTCCCGAGACGGACGGCTTCTCCGACGTGCGCAAGGTGGGCTACCTGCGCAAGCCCAAGAGCATGCACAAGCGATTCTTCGTGCTGCGGGCGGCCAGCGAGGCTGGGGGCCCGGCGCGCCTCGAGTACTACGAGAACGAGAAGAAGTGGCGGCACAAGTCGAGCGCCCCCAAACGCTCGATCCCCCTCGAGAGCTGCTTCAACATCAACAAGCGGGCCGACTCCAAGAACAAGCACCTGGTGGCCCTCTATACCCGGGACGAGCACTTTGCCGTCGCGGCGGACAGCGAGGCCGAGCAGGACAGCTGGTACCAGGCCCTCCTGCAGCTGCACAACCGTGCCAAGGGCCACCACGACGGGGCCGCGGCCCCCGGGGCGGGAGGCGGCGGGGGCAGCTGCAGTGGCAGCTCCGGCCTTGGCGAGGCTGGGGAGGACTTGAGCTACGGGGACGTGCCCCCAGGACCCGCCTTCAAGGAAGTCTGGCAGGTGATCCTGAAACCCAAGGGCCTGGGTCAGACAAAGAACCTGATTGGCATCTACCGCCTCTGCCTGACCAGCAAGACCATCAGCTTCGTGAAGCTGAACTCGGAGGCTGCCGCGGTGGTGCTGCAGCTGATGAACATCAGGCGCTGTGGCCACTCAGAGAACTTCTTTTTCATCGAAGTGGGCCGTTCTGCCGTGACAGGACCCGGGGAGTTCTGGATGCAGGTGGATGACTCTGTGGTGGCCCAGAACATGCATGAGACGATCCTGGAAGCCATGCGGGCCATGAGCGATGAGTTCCGCCCTCGAAGCAAGAGCCAGTCCTCCTCCAACTGCTCCAACCCCATCAGTGTCCCCCTGCGCAGGCATCACCTCAACAACCCTCCGCCCAGCCAGGTGGGGCTGACCCGCCGCTCGCGCACAGAGAGCATCACTGCCACCTCCCCGGCCAGCTTGGTGGGCGGGAAGCAGGGCTCCTTCCGGGTCCGCGCCTCTAGTGATGGCGAAGGCACCATGTCTCGCCCTGCCTCTGTGGACGGCAGTCCTGTGAGTCCCAGCACCAACAGGACCCATGCCCACCGGCATCGCGGCAGTTCCCGGCTGCACCCGCCTCTCAACCACAGCCGCTCCATCCCCATGCCTTCTTCTCGCTGCTCGCCTTCCGCCACCAGCCCGGTCAGTCTGTCGTCCAGTAGCACCAGTGGCCACGGCTCCACCTCGGACTGTCTCTTCCCGCGGCGGTCTAGTGCTTCTGTGTCCGGTTCCCCCAGTGATGGCGGTTTCATCTCCTCTGATGAGTATGGCTCCAGTCCCTGCGACTTCCGAAGTTCCTTCCGCAGTGTCACCCCGGATTCCCTGGGCCACACCCCGCCGGCCCGCGGCGAGGAGGAGCTGAGCAACTACATCTGCATGGGAGGCAAGGGGGCCTCCACCCTCACGGCCCCCAATGGTCACTACATTTTGCCTCGGAGTGGCAACGGTCCCCGCTACATCCCGGCAGCCGGCTTGGGCACGAGCCCAGCCCTGACAGGGGATGAAGCAGCCAGTGCTGCAGATCTGGACAATCGGTTCCGAAAGCGGACTCACTCTGCTGGCACGTCGCCTACCATTTCCCACCAGAAGACCCCATCCCAGTCCTCTGTGGCCTCCATTGAGGAATATACCGAGATGATGCCTGCCTACCCACCAGGAGGTGGCAGTGGAGGCCGAGTGCCCAGCTACCGGCACTCCGCCTTCGTGCCCACCCACTCCTACCCAGAGGAGGGTCTGGAAATGCACCCCTTGGAGCGGCGTGTGGGCCACCACCGCCCAGACACCTCCAACCTCCACACCGATGATGGCTACATGCCCATGTCCCCAGGGGTGGCCCCAGTGCCCGGGAGCCGAAAGGGCAGTGGGGACTACATGCCCATGAGCCCCAAGAGCGTGTCTGCCCCTCAGCAGATCATCAACCCCATCAGACGCCATCCCCAGAGAGTGGACCCCAATGGCTACATGATGATGTCCCCAAGCGGCAGCTGCTCTCCTGACATTGGAGGCGGGCCCAGCAGCGGCGGCAGCAGCGGTGCCGCCCCTTCTGGGAGCAGCTATGGCAAGCTCTGGACGAATGGGGTAGGGGGCCACCACTCTCACGCCCTGCCACACCCCAAACTCCCTGTGGAGAGCAGTAGCGGCAAGCTGTTGTCTTGTACGGGTGACTACATGAACATGTCGCCAGTGGGGGACTCCAACACCAGCAGCCCTTCCAACTGCTACTATGGCCCTGAGGACCCCCAGCACAAGGCAGTCCTCTCCTACTACTCATTGCCAAGGTCCTTTAAGCACACCCAGCGCCCCGGGGAGCTGGAGGAGGGCGCCCGGCACCAGCACCTCCGCCTTTCCTCCAGCTCTGGTCGACTCCTCTATGCCGCGGCGGCAGAAGATTCCTCCTCGTCCACCAGCAGCGACAGCCTGGGTGGGGGATACTGCGCGGCTAGGCCCGAGCCTGGCTTCCCGCATCTCCACCATCAGGTCCTGCAGCCCCATCTGCCTCGAAAGGTGGACACAGCTGCCCAGACCAACAGCCGCCTGGCCCGGCCCACGAGGCTGTCCCTGGGGGATCCCAAGGCCAGCACCTTACCCCGGGCCCGAGAGCAGCCGCAGCCTCAGCCGCAGCCACCCCCGCCCCTGCTGCCCCCTCCGGAGCCCAAGAGCCCAGGGGAATATGTGAATATTGAATTTGGGAGCCATCAGCCGGGCTACTTATCAGGCCCGGGGGCTTCCCACGGCTCGCCTTCTGTCCGGTGTCCATCCCAGCTCCAGCCGGCTCCCAGAGAGGAGGAGACGGGCGCGGAAGAGTACATGAACATGGACCTGGGGCCGGGCCGGAGGGCCGCCTGGCGGGAGAGCGCTGGGGTCCAGCCGGGCAGCGTGGGCCCGGCGCCCCCTGGAGCTGCTAGCGTGTGCAGGCCCACCAGGGCAGTGCCCAGCAGCCGGGGTGACTACATGACCATGCAGTTGGGCTGTCCCCGGCAGAGCTACGTGGACACCTCGCCAGTCGCCCCCATCAGCTATGCGGACATGCGGACCGGCGGCGGCGGCGGCGGCGGCGGCGGCGGCCTCGGCCTCGTGGAGGAGGCCAGCCTGCCTGGGGCCGCCGCGGCCACGCCCTCCTCATCTTCGACGGCCGCGCCTCAAGGAGCAGAGGAGCTGGCGGAGCTGGCGGCCCGCTCGTCCCTGCTGGGGGGCCCGCAGGGACCCGGGGGCCCGAGTGCCTTCACGCGGGTGAACCTCAGTCCCAACCGCAACCAGAGTGCCAAAGTGATCCGTGCCGACCCGCAAGGGTGCAGGAGACGGCACAGCTCCGAGACCTTCTCGACGCCCAGTGCCACCCGGGCAGGCAACACGGTGCCCTTCGGAGCGGGGGCTGCAGCCGGGGGCGGCGGTGGTGGCAGCAGCAGCACAGAGGACGTGAAACGCCACAGCTCTGCTTCCTTTGAGAACGTGTGGCTGCGGCCTGGGGAGCTCGGGGGAGCCCCCAAGGAGCTGGCCCAAGTGTGCGGGGCCGCCGGGGGTTTGGAGAATGGGCTTAACTACATAGACCTGGATTTGGTCAAGGACTTCAACCAGAGCCCTCAAGAGTGCCCCGCTCAGCCGCAGCCTCCTCCTCCCCCGCCCCCTCATCAGCCTCTGGGACGCAGCGAGAGCGGCTCCACCAGCCGCTCCAGTGAGGATTTAAGCGCCTATGCCAGCATCAGTTTCCAGAAGCCGCCAGAGGACCTCCAGTAG

>Sus_sc1 [Pig (Sus scrofa) Irs1]

ATGGCGAGCCCTCCCGAGACGGATGGCTTCTCGGACGTGCGCAAGGTGGGCTACCTGCGCAAACCCAAGAGCATGCACAAGCGCTTTTTCGTGCTGCGGGCGGCCAGCGAGGCTGGGGGCCCCGCGCGCCTCGAGTACTACGAGAACGAGAAGAAGTGGCGGCACAAGTCGAGCGCCCCCAAACGCTCGATCCCCCTGGAGAGCTGCTTCAACATCAACAAGCGGGCGGATTCCAAGAACAAGCACCTGGTGGCCCTCTACACCCGGGACGAGCACTTTGCCATCGCGGCGGACAGCGAGGCCGAACAGGACAGCTGGTACCAGGCCCTCCTGCAGCTGCACAACCGTGCCAAGGGCCACCACGACGGGGCCGCAGGCCCCGGGGCGGGAGGCGGTGGGGGCAGCTGCAGTGGCAGCTCTGGCCTCGGCGAGGCTGGGGAGGACTTGAGCTACGGAGACGTGCCCCCAGGACCCGCCTTCAAGGAGGTTTGGCAGGTGATCCTGAAACCCAAAGGCCTGGGTCAGACAAAGAACCTGATTGGCATCTACCGCCTCTGCCTGACCAGCAAGACCATCAGTTTCGTGAAGCTGAACTCGGAGGCGGCGGCTGTGGTGCTGCAGCTGATGAACATCAGGCGCTGTGGCCACTCAGAGAACTTCTTCTTCATCGAAGTGGGCCGTTCTGCAGTGACGGGACCCGGGGAGTTTTGGATGCAGGTGGATGATTCTGTGGTGGCCCAGAATATGCATGAGACAATCCTGGAGGCCATGCGGGCCATGAGCGATGAGTTCCGCCCTCGAAGCAAGAGCCAGTCCTCCTCTAACTGCTCCAACCCCATCAGTGTACCTCTGCGCAGGCACCACCTCAACAACCCTCCGCCCAGCCAGGTGGGGCTGACCCGCCGCTCACGCACAGAGAGCATCACTGCCACCTCCCCGGCCAGCTTGGTGGGCGGGAAGCAGGGCTCCTTCCGCGTCCGTGCGTCCAGTGATGGGGAAGGCACCATGTCTCGCCCCGCCTCTGTGGACGGCAGTCCTGTGAGCCCTAGCACCAACAGGACTCACGCCCACCGGCATCGAGGCAGCTCCCGGCTGCACCCGCCTCTTAACCACAGCCGCTCTATTCCCATGCCTTCCTCTCGCTGCTCACCCTCCGCCACCAGCCCGGTCAGCCTGTCGTCCAGCAGCACCAGCGGCCATGGCTCCACCTCGGACTGTCTCTTCCCGCGGCGGTCTAGTGCTTCTGTGTCCGGGTCCCCCAGTGACGGCGGTTTCATCTCTTCCGATGAGTATGGCTCTAGCCCCTGTGATTTCCGAAGTTCCTTCCGCAGTGTCACGCCGGATTCCCTGGGCCACACCCCGCCCGCCCGCGGTGAGGAGGAGCTGAGCAACTACATCTGCATGGGGGGCAAGGGGGCCTCCACCCTCGCTGCCCCCAATGGCCACTACATTTTGCCTCGGGGTGGCAATGGCCACCGCTGCCTCCCAGGAGCTGGCTTGGGCACGAGCCCAGCCCTGACCGGGGAGGAAGCAGGCCCTGCATCTGATCTGGATAATCGGTTCCGAAAGCGGACTCACTCTGCTGGCACATCCCCTACTATTTCCCACCAGAAGACCCCATCCCAGTCCTCTGTGGCTTCCATTGAGGAATATACAGAGATGATGCCGGCCTACCCACCAGGAGGTGGCAGTGGAGGCCGGATGCCCAACTACCGGCACTCCGCCTTCGTGCCCACCCACTCCTACCCTGAGGAGGGTCTGGAAATGCACCCCTTGGAGCGGCGTGGGGGTCACCACCGCCAAGACACCTCCAGCCTCCACACCGATGATGGCTACATGCCCATGTCCCCAGGAGTAGCCCCAGTGCCCGGCACCCGAAAGGGCAGTGGGGACTACATGCCCATGAGCCCCAAGAGCGTGTCTGCCCCGCAGCAGATCATCAACCCCATCAGACGCCACCCCCAGAGAGTGGACCCCAATGGCTACATGATGATGTCCCCCAGCGGCAGCTGCTCTCCTGACATTGGAGGTGGTCCCAGCAGCGGGGGCAGCAGCAGCGGTGCCGCCCCTTCTGGGAGCAGCTATGGCAAGTTATGGACAAATGGGGTAGGGGGCCACCACTCTCACGCCCTGCCACACCCCAAACTCCCCGTGGAGAGCAGTAGTGGCAAGCTCTTGTCTTGTACAGGTGACTACATGAACATGTCGCCAGTGGGGGACTCCAACACCAGCAGCCCTTCCGACTGCTACTATGGCCCCGAGGATCCCCAGCACAAGCCAGTCCTCTCCTACTACTCATTGCCAAGGTCCTTCAAGCACACGCAGCGCCCCGGGGAGCTGGAGGAGAGCGCCAGGCACCAGCACCTCCGCCTTTCCTCCAGCTCCGGTCGGCTTCTCTATGCTGCGGCAGCAGAAGATTCGTCGTCATCCACCAGCAGCGACAGCCTGGGCGGGGGATACTGTGGGGCTAGGCCCGAGCCCGGCCTCCCGCATCTCCATCATCAGGTCCTGCAGCCCCATCTGCCTCGAAAGGTGGACACAGCAGCCCAGACCAACAACCGCCTGGCTCGGCCCACGAGGCTGTCCCTGGGGGATCCCAAGGCCAGCACCTTACCTCGGGCCCGAGAGCAGCCGCCACAGCCGGCCCTGCTGCACCCTCCGGAACCCAAAAGTCCAGGGGAATATGTGAATATTGAATTTGGGGGCGATCAGCCGGGCTACTTATCAGGCCCGGTGGCGTCCCACAGCGCGCCTTCGGTGAGATGTTCATCGCAGCTCCAGCCAGCTCCCAGAGAAGAGGAGACTGGCGCCGAAGAGTACATGAACATGGACCTGGGGCCAGGCCGGAGGGCGATCTGGCAGGAGAGCGCTGGGGTCCAGCCCGGCAGGGTGGGCCCCGCCCCCCCGGGAGCTGCTAGCGTGTGCAGGCCGACCCGGGCGGTGCCCAGCAGCCGGGGTGACTACATGACCATGCAGATGGGTTGTCCCCGTCAGAGCTACGTGGACACCTCGCCAGTGGCCCCCATCAGCTATGCCGATATGCGGACCGGCATCGTCGTGGAGGAGGCCAGCCTTCCCGGGGCCACAGCGGCCGCTCCCTCGGCGTCCTCGGCCACCTCTGCTTCCTCCGCCGCGCCCCAAGGAGCAGGGGAGCTGGCGGCCCGCTCTTCCCTGCTGGGGGGCCCGCAGGGACCCGGGGGCCTGAGCGCCTTCACACGGGTGAATCTCAGTCCCAACCGCAACCAGAGTGCCAAAGTGATCCGTGCCGACCCTCAAGGGTGCAGGAGGCGGCACAGCTCCGAGACCTTCTCCTCGACACCCAGTGCCACCCGGGCGGGCAACACGGTACCCTTCGGAGGGGGAGCTGCAGTTGGGGGCGGCGGTGGTGGCAGCAGCAGCACTGAGGATGTGAAACGCCACAGCTCTGCCTCCTTCGAGAACGTGTGGCTGAGGCCTGGGGAGCTTGGGGGAGCCCCCAAGGAGCTGGCCCAGATGTGCGGAGCAGCTGGGGGTTTGGAGAATGGTCTTAACTACATAGACCTGGATTTGGTCAAGGACTTCAAACAGCGCCCTCAAGAGCGCCCCCCTCAACCGCAGCCTCCTCCACCCCCTCCTCCTCATCAGCCTCTGGGCAACAGTGAGAGCAGCTCCACCAGCCGCTCCAGCGAGGATTTAAGCGCCTATGCCAGCATCAGTTTCCAGAAGCAGCCAGAGGACCTCCAGTAG

>Vic_pa1 [Alpaca (Vicugna pacos) Irs1]

ATGGCGAGCCCTCCCGAGACCGACGGCTTCTCGGACGTGCGCAAGGTGGGCTACCTGCGCAAACCCAAGAGCATGCACAAGCGCTTCTTCGTGCTGCGGGCGGCCAGCGAGGCGGGGGGCCCGGCGCGTCTCGAGTACTACGAGAACGAGAAGAAGTGGCGGCACAAGTCGAGCGCCCCCAAACGCTCGATCCCCCTCGAGAGCTGCTTCAACATCAACAAGCGGGCGGACTCCAAGAACAAGCACCTGGTAGCCCTCTACACCCGGGACGAGCACTTTGCCATCGCGGCGGACAGCGAGGCGGAGCAGGACAGCTGGTACCAGGCCCTCCTGCAGCTGCACAACCGAGCCAAGGGCCACCACGACGGGGCCGCGGCCCCCGGGGCGGGAGGCGGCGGGGGCAGCTGCAGTGGCAGCTCTGGCCTCGGCGAGGCTGGGGAGGACTTGAGCTACGGGGACGTGCCCCCAGGACCCGCCTTCAAGGAGGTCTGGCAGGTGATCCTGAAACCCAAGGGCCTGGGTCAGACAAAGAACCTGATTGGCATCTACCGCCTCTGCCTGACCAGCAAGACCATCAGCTTTGTGAAGCTGAACTCGGAGGCGGCGGCTGTGGTGCTGCAGCTGATGAACATCAGGCGCTGTGGGCACTCCGAGAACTTCTTCTTCATCGAGGTGGGCCGTTCCGCAGTGACGGGACCCGGGGAGTTCTGGATGCAGGTGGATGACTCTGTGGTGGCCCAGAACATGCACGAGACAATCCTGGAGGCCATGCGGGCCATGAGCGATGAGTTTCGACCTCGAAGCAAGAGCCAGTCCTCCTCCAACTGCTCCAACCCCATCAGTGTCCCCCTGCGCAGGCACCACCTCAACAACCCTCCACCCAGCCAGGTAGGGCTGACCCGCCGCTCTCGCACCGAGAGCATCACTGCCACCTCCCCTGCCAGTTTGGTGGGCGGGAAGCAGGGCTCCTTCCGCGTCCGTGCCTCCAGTGATGGCGAAGGCACCATGTCTCGCCCCGCCTCTGTGGACGGTAGTCCTGTAAGTCCTAGCACCAACAGGACCCACGCCCACCGGCATCGAGGCAGCTCCCGGCTGCACCCGCCTCTCAACCACAGCCGCTCTATCCCCATGCCTTCTTCTCGCTGCTCACCTTCCGCCACCAGCCCGGTCAGTCTGTCGTCTAGCAGCACCAGTGGCCACGGATCCACCTCGGACTGTCTCTTCCCGCGGCGGTCCAGTGCTTCTGTGTCCGGTTCCCCCAGCGATGGCGGTTTCATCTCCTCAGATGAGTATGGCTCCAGTCCCTGCGATTTCCGAAGTTCCTTTCGTAGTGTCACTCCGGATTCCCTGGGCCACACCCCACCGGCCCGCGGTGAGGAGGAGCTGAGCAACTACATCTGCATGGGAGGCAAGGGGCCCTCCACCCTCGCTGCCCCCAACGGTCACTACATTTTGCCTCGGGGTGGCAACGGTCACCGCTACATCCCAGGAGCTGGTTTGGGGACGAGCCCAGCCCTGACTGGAGATGAAGCAGCCAGTGCCGCGGATCTGGATAATCGGTTCCGAAAGCGAACTCACTCTGCTGGCACATCCCCAACTATTTCCCACCAGAAGACCCCATCCCAGTCCTCTGTGGCTTCCATTGAGGAATATACAGAGATGATGCCTGCCTACCCGCCAGGAGGTGGCAGTGGAGGCCGAGTGCCCAGCTACCGGCACTCCGCCTTCGTGCCCACCCATTCCTACCCTGAGGAGGGTCTAGAAATGCACCCCCTGGAGCGGCGTGGGGGCCACCACCGCCCAGACACCTCCAGCCTCCACACTGATGACGGCTACATGCCCATGTCCCCAGGGGTGGCTCCAGTGCCCGGCAGCCGAAAGGGCAGTGGAGACTACATGCCCATGAGCCCCAAGAGCGTGTCTGCCCCACAACAGATCATCAACCCCATCAGACGCCATCCTCAGAGAGTGGACCCCAATGGCTACATGATGATGTCCCCAAGCGGCAGCTGCTCTCCTGACCTTGGAGGTGGGCCTGGCAGTGGCGGCAGCGGCAGCGGTGCAGCCCCTTCTGGGAGCAGCTATGGCAAAATGTGGACAAACGGGGTAGGAGGCCACCACTCTCACGCCCTGCCACACCCCAAACTCCCCGTGGAGAGCAGTAGTGGCAAGCTCTTGTCTTGTACAGGTGACTACATGAACATGTCGCCAGTGGGGGATTCCAACACCAGCAGCCCTTCCGACTGCTACTATGGCCCTGAGGATCCCCAGCACAAGCCAGTCCTCTCCTACTACTCCTTGCCAAGGTCCTTTAAGCACACCCAGCGCCCGGGAGAGCTGGAGGAGAGCGCCCGGCACCAGCACCTCCGCCTCTCCTCCAGCTCTGGTCGACTCCTCTATGCCACAGCGGCAGAAGATTCCTCCTCCTCCACCAGCAGCGACAGCCTGGGCGGGGGATACTGTGGGGCTAGGCTGGAGCCCAGCCTCCCGCATCTCCACCATCAGGTCCTGCAGCCCCATCTGCCTCGAAAGGTGGACACAGCTGCCCAGACCAACAACCGCCTGGCCCGGCCCACGAGGCTGTCCCTGGGGGATCCCAAGGCCAGCACCTTACCTCGGGCCAGAGAGCAGCAGCCGCCGCCACCCTTGCTGCACCCTCCGGAGCCCAAGAGCCCAGGGGAATATGTGAATATTGAATTCGGGAGCGATCAGCCCGGCTACTTATCTGGCCCGGTGGCTCCCCACAGCTCGCCTTCTGTCAGGTGCCCATCCCAGCTCCAGCCAGCTCCCAGAGAGGAAGAGGCTGGCGCGGAAGAGTACATGAACATGGACCTGGGGCCCGGCAGGAGGGCCACCTGGCAGGAGAGCGCAGGGGTCCAGCCCGGGAGGGTGGGCCCCGCCCCCCCCGGAGCTGCTAGCATGTGCCGGCCGACCCGGGCAGTGCCTAGCAGCCGGGGTGACTATATGACCATGCAGATGGGCTGCCCCCATCAGAGCTACGTGGACACCTCGCCGGTTGCCCCCATCAGCTACGCCGATATGCGGACCGGCATCGTTGTGGAGGAGGCCAGCCTTCCCGGGGCCACAGCGGCCGCGCCCTCCTCATCCTCCACGACCTCTGCTCCCCCTGCAGCGCCTCAAGGAGCAGGGGAGCTGGCGGCCTGCTCGTCCCTGCTGGGGGGCCCGCAGGGACCTGGGGGCCCGAGCGCCTTCACGCGGGTGAACCTCAGTCCCAGCCGTAACCAGAGTGCCAAAGTGATCCGTGCCGACCCGCAAGGGTGCAGGAGGAGGCACAGCTCCGAGACCTTCTCCTCAACGCCCAGTGCCACTCGGGCGGGCAACACCCTGCCCTTCGGAGGCGGGGCTGCAGTCGGGGGCAGTGGTGGTGGCAGCAGCAGCGCTGAGGATGTGAAGCGCCACAGCTCTGCTTCCTTTGAGAACGTATGGCTGAGGCCTGGGGAGCTGGGGGGAGCCCCCAAGGAGCTGGCTCAAGTGTGCGGGGCAGCCGGGGGTTTGGAGAATGGTCTTAACTACATAGACCTGGATCTGGTCAAGGACTTCAAACAGCGCCCTCAGGAGAGACCCCCTCAACCGCAGCCTCCTCCACCCCCGACCCCCCATCAGCCTCTGGGCAGCGGCGAGAGCAGCTCCACCAGCCGCTCCAGCGAGGATTTAAGCGCCTATGCCAGCATCAGTTTCCAGAAGCAGCCAGAGGACCTCCAGTAG

>Cam_fe1 [Wild Bactrain camel (Camelus ferus) Irs1]

ATGGCGAGCCCTCCCGAGACCGACGGCTTCTCGGACGTGCGCAAGGTGGGCTACCTGCGCAAACCCAAGAGCATGCACAAGCGCTTCTTCGTGCTGCGGGCGGCCAGCGAGGCGGGGGGCCCGGCGCGTCTCGAGTACTACGAGAACGAGAAGAAGTGGCGGCACAAGTCGAGCGCCCCCAAACGCTCGATCCCCCTCGAGAGCTGCTTCAACATCAACAAGCGGGCGGACTCCAAGAACAAGCACCTGGTAGCCCTCTACACCCGGGACGAGCACTTTGCCATCGCGGCGGACAGCGAGGCGGAGCAGGACAGCTGGTACCAGGCCCTCCTGCAGCTGCACAACCGAGCCAAGGGCCACCACGACGGGGCCGCGGCCCCCGGGGCGGGAGGCGGCGGGGGCAGCTGCAGTGGCAGCTCTGGCCTCGGCGAGGCTGGGGAGGACTTGAGCTACGGGGACGTGCCCCCAGGACCCGCCTTCAAGGAGGTCTGGCAGGTGATCCTGAAACCCAAGGGCCTGGGTCAGACAAAGAACCTGATTGGCATCTACCGCCTCTGCCTGACCAGCAAGACCATCAGCTTTGTGAAGCTGAACTCGGAGGCGGCGGCTGTGGTACTGCAGCTGATGAACATCAGGCGCTGTGGGCACTCCGAGAACTTCTTCTTCATCGAGGTGGGCCGTTCCGCAGTGACGGGACCCGGGGAGTTCTGGATGCAGGTGGATGACTCTGTGGTGGCCCAGAACATGCATGAGACAATCCTGGAGGCCATGCGGGCCATGAGCGATGAGTTTCGACCTCGAAGCAAGAGCCAGTCCTCCTCCAACTGCTCCAACCCCATCAGTGTCCCCCTGCGCAGGCACCACCTCAACAACCCTCCACCCAGCCAGGTAGGGCTGACCCGCCGCTCTCGCACAGAGAGCATCACTGCCACCTCCCCTGCCAGCTTGGTGGGCGGGAAGCAGGGCTCCTTCCGCGTCCGTGCCTCCAGTGATGGCGAAGGCACCATGTCTCGCCCCGCCTCTGTGGACGGTAGTCCTGTAAGTCCTAGCACCAACAGGACCCACGCCCACCGGCATCGAGGCAGCTCCCGGCTGCACCCGCCTCTCAACCACAGCCGCTCTATCCCCATGCCTTCTTCTCGCTGCTCACCTTCTGCCACCAGCCCGGTCAGTCTGTCGTCTAGCAGCACCAGTGGCCACGGATCCACCTCGGACTGTCTCTTCCCGCGGCGGTCTAGTGCTTCTGTGTCCGGTTCCCCCAGCGATGGCGGTTTCATCTCCTCAGATGAGTATGGCTCCAGTCCCTGCGATTTCCGAAGTTCCTTTCGTAGTGTCACTCCGGATTCCCTGGGCCACACCCCACCGGCCCGCGGTGAGGAGGAGCTGAGCAACTACATCTGCATGGGAGGCAAGGGGCCCTCCACCCTCGCTGCCCCCAACGGTCACTACATTTTGCCTCGGGGTGGCAACGGTCACCGCTACATCCCAGGAGCTGGTTTGGGGACGAGCCCAGCCCTGACTGGAGATGAAGCAGCCAGTGCCGCGGATCTGGATAATCGGTTCCGAAAGCGAACTCACTCTGCTGGCACATCCCCTACCATTTCCCACCAGAAGACCCCATCCCAGTCCTCTGTGGCTTCCATTGAGGAATATACAGAGATGATGCCTGCCTACCCGCCAGGAGGTGGCAGCGGAGGCCGAGTGCCCAGCTACCGGCACTCCGCCTTCGTGCCCACCCATTCCTACCCTGAGGAGGGTCTAGAAATGCACCCCCTGGAGCGGCGTGGGGGCCACCACCGCCCAGACACCTCCAGTCTCCACACCGATGACGGCTACATGCCCATGTCCCCAGGGGTGGCTCCAGTGCCCGGCAGCCGAAAGGGCAGTGGAGACTACATGCCCATGAGCCCCAAGAGCGTGTCTGCCCCACAACAGATCATCAACCCCATCAGACGCCATCCTCAGAGAGTGGACCCCAATGGCTACATGATGATGTCCCCAAGCGGCAGCTGCTCTCCTGACCTTGGAGGTGGGCCTGGCAGTGGCGGCAGCGGCAGCGGTGCAGCCCCTTCTGGGAGCAGCTATGGCAAGCTGTGGACGAACGGGGTAGGAGGCCACCACTCTCACGCCCTGCCACACCCCAAACTCCCCGTGGAGAGCAGTAGTGGCAAGCTCTTGTCTTGTACAGGTGACTACATGAACATGTCGCCAGTGGGGGACTCCAACACCAGCAGCCCTTCCGACTGCTACTATGGCCCTGAGGATCCCCAGCACAAGCCAGTCCTCTCCTACTACTCCTTGCCAAGGTCCTTTAAGCACACCCAGCGCCCGGGAGAGCTGGAGGAGAGCGCCCGGCACCAGCACCTCCGCCTCTCCTCCAGCTCTGGTCGACTCCTCTATGCCACAGCGGCAGAAGATTCCTCCTCCTCCACCAGCAGCGACAGCCTGGGCGGGGGATACTGTGGGGCTAGGCTGGAGCCCAGCCTCCCGCATCTCCACCATCAGGTCCTGCAGCCCCATCTGCCTCGAAAGGTGGACACAGCTGCCCAGACCAACAACCGCCTGGCCCGGCCCACGAGGCTGTCCCTGGGGGATCCCAAGGCCAGCACCTTACCTCGGGCCAGAGAGCAGCAGCCGCCGCCCTTGCTGCACCCTCCGGAGCCCAAGAGCCCAGGGGAATATGTGAATATTGAATTCGGGAGCGATCAGCCCGGCTACTTATCTGGCCCAGTGGCTCCCCACAGCTCGCCTTCTGTCAGGTGCCCATCCCAGCTCCAGCCAGCTCCCAGAGAGGAAGAGGCTGGCGCGGAAGAGTACATGAACATGGACCTGGGGCCCGGCAGGAGGGCCACCTGGCAGGAGAGCGCAGGGGTCCAGCCCGGGAGGGTGGGCCCCGCCCCCCCCGGAGCTGTTAGCGTGTGCCGGCCGACCCGGGCAGTGCCTAGCAGCCGGGGTGACTACATGACCATGCAGGTGGGCTGCCCCCATCAGAGCTACGTGGACACCTCGCCGGTTGCCCCCATCAGCTACGCCGATATGCGGACCGGAATCGTTGTGGAGGAGGCCAGCCTTCCCGGGGCCACAGCGGCCGCGCCCTCCTCATCCTCCACGACCTCTGCTTCCCCTGCAGAGCCTCAAGGAGCCGGGGAGCTGGCGGCCTGCTCGTCCCTGCTGGGGGGCCCGCAGGGACCTGGGGGCCCGAGCGCCTTCACGCGGGTGAACCTCAGTCCCAGCCGTAACCAGAGTGCCAAAGTGATCCGTGCCGACCCGCAAGGGTGCAGGAGGAGGCACAGCTCCGAGACCTTCTCCTCAACGCCCAGTGCCACTCGGGCGGGCAACACCCTGCCCTTCGGAGGCGGGGCTGCAGTCGGGGGCAGTGGTGGTGGCAGCAGCAGCGCTGAGGATGTGAAGCGCCACAGCTCTGCTTCCTTTGAGAACGTGTGGCTGAGGCGTGGGGAGCTGGGGGGAGCCCCCAAGGAGCTGGCTCAAGTGTGCGGGGCAGCCGGGGGTTTGGAGAATGGTCTTAACTACATAGACCTGGATCTGGTCAAGGACTTCAAACAGCGCCCTCAGGAGCGACCCCCTCAACCGCAGCCTCCTCCACCCCCGCCCCCCCATCAGCCTCTGGGCAGCGGCGAGAGCAGCTCCACCAGCCGCTCCAGCGAGGATTTAAGCGCCTATGCCAGCATCAGTTTCCAGAAGCAGCCAGAGGACCTCCAGTAG

>Cam_dr1 [Arabian camel (Camelus dromedarius) Irs1]

ATGGCGAGCCCTCCCGAGACCGACGGCTTCTCGGACGTGCGCAAGGTGGGCTACCTGCGCAAACCCAAGAGCATGCACAAGCGCTTCTTCGTGCTGCGGGCGGCCAGCGAGGCGGGGGGCCCGGCGGCGCGTCTCGAGTACTACGAGAACGAGAAGAAGTGGCGGCACAAGTCGAGCGCCCCCAAACGCTCGATCCCCCTCGAGAGCTGCTTCAACATCAACAAGCGGGCGGACTCCAAGAACAAGCACCTGGTAGCCCTCTACACCCGGGACGAGCACTTTGCCATCGCGGCGGACAGCGAGGCGGAGCAGGACAGCTGGTACCAGGCCCTCCTGCAGCTGCACAACCGAGCCAAGGGCCACCACGACGGGGCCGCGGCCCCCGGGGCGGGAGGCGGCGGGGGCAGCTGCAGTGGCAGCTCTGGCCTCGGCGAGGCTGGGGAGGACTTGAGCTACGGGGACGTGCCCCCAGGACCCGCCTTCAAGGAGGTCTGGCAGGTGATCCTGAAACCCAAGGGCCTGGGTCAGACAAAGAACCTGATTGGCATCTACCGCCTCTGCCTGACCAGCAAGACCATCAGCTTTGTGAAGCTGAACTCGGAGGCGGCGGCTGTGGTACTGCAGCTGATGAACATCAGGCGCTGTGGGCACTCCGAGAACTTCTTCTTCATCGAGGTGGGCCGTTCCGCAGTGACGGGACCCGGGGAGTTCTGGATGCAGGTGGATGACTCTGTGGTGGCCCAGAACATGCATGAGACAATCCTGGAGGCCATGCGGGCCATGAGCGATGAGTTTCGACCTCGAAGCAAGAGCCAGTCCTCCTCCAACTGCTCCAACCCCATCAGTGTCCCCCTGCGCAGGCACCACCTCAACAACCCTCCACCCAGCCAGGTAGGGCTGACCCGCCGCTCTCGCACAGAGAGCATCACTGCCACCTCCCCTGCCAGCTTGGTGGGCGGGAAGCAGGGCTCCTTCCGCGTCCGTGCCTCCAGTGATGGCGAAGGCACCATGTCTCGCCCCGCCTCTGTGGACGGTAGTCCTGTAAGTCCTAGCACCAACAGGACCCACGCCCACCGGCATCGAGGCAGCTCCCGGCTGCACCCGCCTCTCAACCACACTCTCAACCACAGCCGCTCTATCCCCATGCCTTCTTCTCGCTGCTCACCTTCTGCCACCAGCCCGGTCAGTCTGTCGTCTAGCAGCACCAGTGGCCACGGATCCACCTCGGACTGTCTCTTCCCGCGGCGGTCTAGTGCTTCTGTGTCCGGTTCCCCCAGCGATGGCGGTTTCATCTCCTCAGATGAGTATGGCTCCAGTCCCTGCGATTTCCGAAGTTCCTTTCGTAGTGTCACTCCGGATTCCCTGGGCCACACCCCACCGGCCCGCGGTGAGGAGGAGCTGAGCAACTACATCTGCATGGGAGGCAAGGGGCCCTCCACCCTCGCTGCCCCCAACGGTCACTACATTTTGCCTCGGGGTGGCAACGGTCACCGCTACATCCCAGGAGCTGGTTTGGGGACGAGCCCAGCCCTGACTGGAGATGAAGCAGCCAGTGCCGCGGATCTGGATAATCGGTTCCGAAAGCGAACTCACTCTGCTGGCACATCCCCTACCATTTCCCACCAGAAGACCCCATCCCAGTCCTCTGTGGCTTCCATTGAGGAATATACAGAGATGATGCCTGCCTACCCGCCAGGAGGTGGCAGCGGAGGCCGAGTGCCCAGCTACCGGCACTCCGCCTTCGTGCCCACCCATTCCTACCCTGAGGAGGGTCTAGAAATGCACCCCCTGGAGCGGCGTGGGGGCCACCACCGCCCAGACACCTCCAGTCTCCACACCGATGACGGCTACATGCCCATGTCCCCAGGGGTGGCTCCAGTGCCCGGCAGCCGAAAGGGCAGTGGAGACTACATGCCCATGAGCCCCAAGAGCGTGTCTGCCCCACAACAGATTATCAACCCCATCAGACGCCATCCTCAGAGAGTGGACCCCAATGGCTACATGATGATGTCCCCAAGCGGCAGCTGCTCTCCTGACCTTGGAGGTGGGCCTGGCAGTGGCGGCAGCGGCAGCGGTGCAGCCCCTTCTGGGAGCAGCTATGGCAAGCTGTGGACGAACGGGGTAGGAGGCCACCACTCTCACGCCCTGCCACACCCCAAACTCCCCGTGGAGAGCAGTAGTGGCAAGCTCTTGTCTTGTACAGGTGACTACATGAACATGTCGCCAGTGGGGGACTCCAACACCAGCAGCCCTTCCGACTGCTACTATGGCCCTGAGGATCCCCAGCACAAGCCAGTCCTCTCCTACTACTCCTTGCCAAGGTCCTTTAAGCACACCCAGCGCCCGGGAGAGCTGGAGGAGAGCGCCCGGCACCAGCACCTCCGCCTCTCCTCCAGCTCTGGTCGACTCCTCTATGCCACAGCGGCAGAAGATTCCTCCTCCTCCACCAGCAGCGACAGCCTGGGCGGGGGATACTGTGGGGCTAGGCTGGAGCCCAGCCTCCCGCATCTCCACCATCAGGTCCTGCAGCCCCATCTGCCTCGAAAGGTGGACACAGCTGCCCAGACCAACAACCGCCTGGCCCGGCCCACGAGGCTGTCCCTGGGGGATCCCAAGGCCAGCACCTTACCTCGGGCCAGAGAGCAGCAGCCGCCGCCCTTGCTGCACCCTCCGGAGCCCAAGAGCCCAGGGGAATATGTGAATATTGAATTCGGGAGCGATCAGCCCGGCTACTTATCTGGCCCAGTGGCTCCCCACAGCTCGCCTTCTGTCAGGTGCCCATCCCAGCTCCAGCCAGCTCCCAGAGAGGAAGAGGCTGGCGCGGAAGAGTACATGAACATGGACCTGGGGCCCGGCAGGAGGGCCACCTGGCAGGAGAGCGCAGGGGTCCAGCCCGGGAGGGTGGGCCCCGCCCCCCCCGGAGCTGTTAGCGTGTGCCGGCCGACCCGGGCAGTGCCTAGCAGCCGGGGTGACTACATGACCATGCAGGTGGGCTGCCCCCATCAGAGCTACGTGGACACCTCGCCGGTTGCCCCCATCAGCTACGCCGATATGCGGACCGGAATCGTTGTGGAGGAGGCCAGCCTTCCCGGGGCCACAGCGGCCGCGCCCTCCTCATCCTCCACGACCTCTGCTTCCCCTGCAGAGCCTCAAGGAGCAGGGGAGCTGGCGGCCTGCTCGTCCCTGCTGGGGGGCCCGCAGGGACCTGGGGGCCCGAGCGCCTTCACGCGGGGGAACCTCAGTCCCAGCCGTAACCAGAGTGCCAAAGTGATCCGTGCCGACCCGCAAGGGTGCAGGAGGAGGCACAGCTCCGAGACCTTCTCCTCAACGCCCAGTGCCACTCGGGCGGGCAACACCCTGCCCTTCGGAGGCGGGGCTGCAGTCGGGGGCAGTGGTGGTGGCAGCAGCAGCGCTGAGGATGTGAAGCGCCACAGCTCTGCTTCCTTTGAGAACGTGTGGCTGAGGCCTGGGGAGCTGGGGGGAGCCCCCAAGGAGCTGGCTCAAGTGTGCGGGGCAGCCGGGGGTTTGGAGAATGGTCTTAACTACATAGACCTGGATCTGGTCAAGGACTTCAAACAGCGCCCTCAGGAGCGACCCCCTCAACCGCAGCCTCCTCCACCCCCGCCCCCCCATCAGCCTCTGGGCAGCGGCGAGAGCAGCTCCACCAGCCGCTCCAGCGAGGATTTAAGCGCCTATGCCAGCATCAGTTTCCAGAAGCAGCCAGAGGACCTCCAGTAG

>Equ_as1 [Ass (Equus asinus) Irs1]

ATGGCGAGCCCTCCGGAGACCGACGGCTTCTCGGACGTGCGCAAGGTGGGCTACCTGCGCAAACCCAAGAGCATGCACAAGCGCTTCTTCGTGCTGCGGGCGGCCAGCGAGGCGGGGGGCCCGGCGCGCCTCGAGTACTACGAGAACGAGAAGAAGTGGCGGCACAAGTCGAGCGCCCCCAAACGCTCGATCCCCCTCGAGAGCTGCTTCAACATCAACAAGCGGGCGGACTCCAAGAACAAGCACCTGGTGGCCCTCTACACCCGGGACGAGCACTTTGCTATCGCGGCGGACAGCGAGGCCGAGCAGGACAGCTGGTACCAGGCCCTCCTGCAGCTGCACAACCGTGCCAAGGGCCACCACGACGGGGCCGCGGCCCCTGGGGCGGGAGGCGGAGGGGGCAGCTGCAGCGGCAGCTCTGGCCTCGGCGAGGCAGGGGAGGACTTGAGCTACGGGGACGTGCCCCCAGGACCTGCGTTCAAAGAGGTCTGGCAGGTGATCCTGAAACCCAAGGGCCTGGGTCAGACAAAGAACCTGATTGGCATCTACCGCCTCTGCCTGACCAGCAAGACCATCAGCTTCGTGAAGCTGAACTCGGAGGCGGCGGCCGTGGTGCTGCAGCTGATGAACATCAGGCGCTGTGGCCACTCAGAGAACTTCTTCTTCATCGAAGTGGGCCGTTCCGCAGTGACGGGACCCGGGGAGTTCTGGATGCAGGTGGATGACTCTGTGGTGGCTCAGAACATGCACGAGACAATCCTGGAGGCCATGCGGGCCATGAGCGATGAGTTCCGCCCTCGCAGCAAGAGCCAGTCCTCGTCCAACTGCTCCAACCCCATCAGCGTACCCCTGCGCAGGCACCACCTCAACAACCCCCCGCCCAGCCAGGTGGGGCTGACTCGCCGCTCGCGCACCGAGAGCATCACCGCCACCTCCCCGGCCAGCATGGTGGGCGGGAAGCAGGGCTCCTTCCGTGTCCGTGCTTCCAGCGACGGCGAAGGCACTATGTCCCGTCCAGCTTCGGTGGATGGTAGTCCTGTGAGTCCTAGCACCAACAGGACCCACGCCCACCGGCATCGGGGCAGCTCTCGGCTGCACCCCCCTCTCAACCACAGCCGCTCCATCCCCATGCCTTCTTCTCGATGCTCACCTTCGGCCACTAGCCCGGTCAGTCTGTCGTCCAGCAGCACCAGTGGCCACGGCTCCACCTCAGACTGTCTCTTCCCGCGGCGGTCTAGTGCTTCTGTGTCCGGCTCCCCCAGCGATGGCGGTTTCATCTCCTCGGATGAGTATGGCTCCAGTCCCTGCGATTTCCGAAGTTCCTTCCGCAGTGTCACCCCAGATTCCCTGGGCCACACCCCACCGGCCCGGGGTGAGGAGGAGTTGAGCAACTACATCTGCATGGGAGGCAAGGGGGCCTCCACCCTCACTGCCCCCAATGGTCACTACATTTTGCCTCGGGGTGGCAATGGTCACCGCTACATCCCAGGAGCTGGCTTGGGCACGAGTCCAGCCCTGGCTGGAGATGAAGCAGCCAGTGCTGCAGATCTGGATAATCGGTTCCGAAAGCGGACTCACTCTGCTGGCACATCTCCTACCATTTCCCACCAGAAGACCCCATCCCAGTCCTCTGTGGCTTCCATTGAGGAATATACAGAGATGATGCCTGCCTACCCACCAGGAGGTGGCAGTGGAGGCCGACTGCCCGGCTACCGGCACTCGGCCTTTGTGCCCACCCATTCCTACCCTGAGGAGGGTCTGGAAATGCACCCTTTGGAGCGTGGTGGGGGCCACCACCGCCCAGACACCTCAACTCTCCACACTGATGATGGCTACATGCCCATGTCCCCAGGAGTGGCCCCAGTGCCTGGCAGCCGAAAGGGCAGTGGGGACTATATGCCTATGAGCCCCAAGAGCGTGTCTGCCCCACAGCAGATCATCAACCCCATCAGACGCCATCCCCAGAGAGTGGACCCCAATGGCTACATGATGATGTCCCCAAGCGGCAGCTGCTCCCCTGACATTGGAGGTGGGTCCAGCAGCAGCAGCAGTGCTGCCCCTTCTGGGAGCAGCTATGGGAAGCTATGGACAAATGGTGTAGGGGGCCACCACTCTCACGCCCTCCCACACCCCAAACTACCCATGGAGAGCAGTGGTAGCAAGCTCTTGTCTTGTACAGGTGACTACATGAACATGTCGCCAGTGGGGGACTCCAACACTAGCAGCCCCTCTGACTGCTACTATGGCCCTGAGGACCCCCAGCACAAGCCAGTACTCTCCTACTACTCATTGCCAAGGTCCTTTAAGCACACCCAGCGCCCTGGGGAGCTGGAGGAGACTGCCCGGCACCAGCACCTCCGCCTTTCCTCCAGCTCTGGTCGCCTTCTCTATGCTGCAGCAGCAGAAGATTCCTCTTCCTCCACCAGCAGTGACAGCCTGGGTGGGGGATACTGTGGGGCTAGATCTGAGCCCGGCCTCCCACATCATCTCCACCATCAGGTCCTGCAGCCTCATCTGCCTCGAAAGGTGGACACAGCTGCGCAGACCAACAGCCGCCTTGCTCGGCCCACAAGGCTGTCCCTGGGTGATCCCAAGGCCAGCACCTTACCTCGGGCCCGAGACCAGCAGCAGCAGCCACCCCTGCTGCACCCTCCGGAGCCCAAGAGCCCAGGGGAATATGTGAATATTGAATTTGGGAGTGATCAGCCAGGCTACTTATCTGGCCCCATGGCTCCCCACAGCTCGCCTTCTGTCAGGTGTCCATCCCAGCTCCAGCCAGCTCCCAGAGAGGAAGAGACTGGCCCTGAAGAATACATGAACATGGACTTGGGGCCGGGCCGGAGGGCAGCCTGGCAGGAGAGCGCTGGGGTCCAGCCTGGCAGAGTAGGCCCTGCGCCTCCTGGGTCTGCTAGCATGTGCAGGCCTACTCGGGCAGTGCCCAGCAGCCGGGGTGACTACATGACCATGCAGATGGGTTGTCCCCGTCAGAGCTACGTGGACACCTCGCCAGTTGCCCCCATCAGCTATGCTGACATGCGGACGGGCATTGTTGTGGAGGAGGTGAGCCTTCCCGGGGCCACAGCGGCTGCTCCTTCCTCAACCTCAGCAGCCTCTGCTTCTCCTGCTGCACCTCAAGGGGCAGGGGAGCTGGGGGCCCGCTCTTCCCTGCTGGGGGGCCCGCAGGGACCTGGTGGCATGAGCGCCTTCACCCGGGTGAACCTCAGCCCCAATCGCAACCAGAGTGCCAAAGTGATCCGTGCGGACCCGCAAGGGTGCCGGAGGCGGCATAGCTCCGAGACCTTCTCCTCGACACCTAGTGCCACCCGGGCGGGCAATATGGTGCCCTTCGGAGCGGGGGCTGTGGTGGGGAGCAGCGGTGGTGGCAGCAGCAGCACTGAGGATGTGAAGCGCCACAGCTCTGCTTCCTTTGAGAACGTGTGGCTGAGGCCTGGGGAGCTCGGGGGAGTCCCCAAGGAGCCAGCCCAAGTGTGCGGGGCTGCTGGGGGTTTGGAGAATGGTCTTAACTACATAGATCTGGATTTGGTCAAGGACTTCAAACAGCGCCCTCAGGAGCGCCCCTCTCAGCAGCAGCCTCCCCTACCCCCGGCCCCTCATCAGCCTCTTGGCAGCAGTGAGAGCAGCTCCACCAGCCGCTCCAGTGAGGATTTAAGCGCCTATGCCAGCATCAGTTTCCAGAAGCAGCCAGAGGACCTCCAGTAG

>Equ_pr1 [Przewalski's horse (Equus przewalskii) Irs1]

ATGGCGAGCCCTCCGGAGACCGACGGCTTCTCGGACGTGCGCAAGGTGGGCTACCTGCGCAAACCCAAGAGCATGCACAAGCGCTTCTTCGTGCTGCGGGCGGCCAGCGAGGCTGGGGGCCCGGCGCGCCTCGAGTACTACGAGAACGAGAAGAAGTGGCGGCACAAGTCGAGCGCCCCCAAACGCTCGATCCCCCTCGAGAGCTGCTTCAACATCAACAAGCGGGCGGACTCCAAGAACAAGCACCTGGTGGCCCTCTACACCCGGGACGAGCACTTTGCTATCGCGGCGGACAGCGAGGCCGAGCAGGACAGCTGGTACCAGGCCCTCCTGCAGCTGCACAACCGTGCCAAGGGCCACCACGACGGGGCCGCGGCCCCTGGGGCGGGAGGCGGAGGGGGCAGCTGCAGCGGCAGCTCTGGCCTCGGCGAGGCAGGGGAGGACTTGAGCTACGGGGACGTGCCCCCAGGACCTGCGTTCAAAGAGGTCTGGCAGGTGATCCTGAAACCCAAGGGCCTGGGTCAGACAAAGAACCTGATTGGCATCTACCGCCTCTGCCTGACCAGCAAGACCATCAGCTTCGTGAAGCTGAACTCGGAGGCGGCGGCCGTGGTGCTGCAGCTGATGAACATCAGGCGCTGTGGCCACTCAGAGAACTTCTTCTTCATTGAAGTGGGCCGTTCCGCAGTGACGGGACCCGGGGAGTTCTGGATGCAGGTGGATGACTCTGTGGTGGCTCAGAACATGCACGAGACAATCCTGGAGGCCATGCGGGCCATGAGCGATGAGTTCCGCCCTCGCAGCAAGAGCCAGTCCTCGTCCAACTGCTCCAACCCCATCAGCGTACCCCTGCGCAGGCACCACCTCAACAACCCCCCGCCCAGCCAGGTGGGGCTGACTCGCCGCTCGCGCACCGAGAGCATCACCGCCACCTCCCCGGCCAGCATGGTGGGCGGGAAGCAGGGCTCCTTCCGTGTCCGTGCTTCCAGCGACGGCGAAGGCACTATGTCCCGTCCAGCTTCGGTGGATGGCAGTCCTGTGAGTCCTAGCACCAACAGGACCCACGCCCACCGGCATCGGGGCAGCTCTCGGCTGCCCCCCCCTCTCAACCACAGCCGCTCCATCCCTATGCCTTCTTCTCGATGCTCACCTTCGGCCACTAGCCCGGTCAGTCTGTCGTCCAGCAGCACCAGTGGCCACGGCTCCACCTCAGACTGTCTCTTCCCGCGGCGGTCTAGTGCTTCTGTGTCCGGCTCCCCCAGCGATGGCGGTTTCATCTCCTCGGATGAGTATGGCTCCAGTCCCTGCGATTTCCGAAGTTCCTTCCGCAGTGTCACCCCAGATTCCCTGGGCCACACCCCACCGGCCCGGGGTGAGGAGGAGTTGAGCAACTACATCTGCATGGGAGGCAAGGGGGCCTCCACCCTCACTGCCCCCAATGGTCACTACATTTTGCCTCGGGGTGGCAATGGTCACCGCTACGTCCCAGGAGCTGGCTTGGGCACGAGTCCAGCCCTGGCTGGAGATGAAGCAGCCGGTGCTGCAGATCTGGATAATCGGTTCCGAAAGCGGACTCACTCTGCTGGCACATCTCCTACCATTTCCCACCAGAAGACCCCATCCCAGTCCTCTGTGGCTTCCATTGAGGAATATACAGAGATGATGCCTGCCTACCCACCAGGAGGTGGCAGTGGAGGCCGACTGCCCGGCTACCGGCACTCGGCCTTTGTGCCCACCCATTCCTACCCTGAGGAGGGTCTGGAAATGCACCCTTTGGAGCGTGGTGGGGGCCACCACCGCCCAGACACCTCAACTCTCCACACTGATGATGGCTACATGCCCATGTCCCCAGGAGTGGCCCCAGTGCCTGGCAGCCGAAAGGGCAGTGGGGACTATATGCCTATGAGCCCCAAGAGCGTGTCTGCCCCGCAGCAAATCATCAACCCCATCAGACGCCATCCCCAGAGAGTGGACCCCAATGGCTACATGATGATGTCCCCAAGCGGCAGCTGCTCCCCTGACATTGGAGGTGGGTCCAGCAGCAGCAGCAGTGCTGCCCCTTCTGGGAGCAGCTATGGGAAGCTATGGACAAATGGTGTAGGGGGCCACCACTCTCACGCCCTCCCACACCCCAAACTACCCATGGAGAGCAGTGGGAGCAAGCTCTTGTCTTGTACAGGTGACTACATGAACATGTCGCCAGTGGGGGACTCCAACACTAGCAGCCCCTCTGACTGCTACTACGGCCCTGAGGACCCCCAGCACAAGCCAGTACTCTCCTACTACTCATTGCCAAGGTCCTTTAAGCACACCCAGCGCCCTGGGGAGCTGGAGGAGACTGCCCGGCACCAGCACCTCCGCCTTTCCTCCAGCTCTGGTCGCCTTCTCTATGCTGCAGCAGCAGAAGATTCCTCTTCCTCCACCAGCAGTGACAGCCTGGGTGGGGGATACTGTGGGGCTAGATCCGAGCCCGGCCTCCCACATCATCTCCACCATCAGGTCCTGCAGCCTCATCTGCCTCGAAAGGTGGACACAGCTGCGCAGACCAACAGCCGCCTTGCTCGGCCCACGAGGCTGTCCCTGGGTGATCCCAAGGCCAGCACCTTACCTCGGGCCCGAGACCAGCAGCAGCAGCCACCCCTGCTGCACCCTCCGGAGCCCAAGAGCCCAGGGGAATATGTGAATATTGAATTTGGGAGTGATCAGCCAGGCTACTTATCTGGCCCCATGGCTCCCCACAGCTCGCCTTCTGTCAGGTGTCCATCCCAGCTCCAGCCAGCTTCCAGAGAGGAAGAGACTGGCCCTGAAGAATACATGAACATGGACTTGGGGCCGGGCCGGAGGGCAGCCTGGCAGGAGAGCGCTGGGGTCCAGCCTGGCAGAGTAGGCCCTGCGCCTCCTGGGTCTGCTAGCATGTGCAGGCCTACTCGGGCAGTGCCCAGCAGCCGGGGTGACTACATGACCATGCAGATGGGTTGTCCCCGTCAGAGCTACGTGGACACCTCGCCAGTTGCCCCCATCAGCTATGCTGACATGCGGACGGGCATTGTTGTGGAGGAGGTGAGCCTTCCTGGGGCCACAGCGGCTGCTCCTTCCTCAACCTCAGCAGCCTCTGCTTCTCCTGCTGCACCTCAAGGGGCAGGGGAGCTGGGGGCCCGCTCTTCCCTGCTGGGGGGCCCGCAGGGACCTGGTGGCATGAGCGCCTTCACCCGGGTGAACCTCAGCCCCAATCGCAACCAGAGTGCCAAAGTGATCCGTGCGGACCCGCAAGGGTGCCGGAGGCGGCATAGCTCCGAGACCTTCTCCTCGACACCTAGTGCCACCCGGGCGGGCAATATGGTGCCCTTCGGAGCGGGGGCTGTGGTGGGGAGCAGCGGTGGTGGCAGCAGCAGCACTGAGGATGTGAAGCGCCACAGCTCTGCTTCCTTTGAGAACGTGTGGCTGAGGCCTGGGGAGCTCGGGGGAGCCCCCAAGGAGCCAGCCCAAGTGTGCGGGGCTGCTGGGGGTTTGGAGAATGGTCTTAACTACATAGATCTGGATTTGGTCAAGGACTTCAAACAGCGCCCTCAGGAGCGCCCCTCTCAGCAGCAGCCTCCCCCACCCCCGGCCCCTCATCAGCCTCTTGGCAGCAGTGAGAGCAGCTCCACCAGCCGCTCCAGTGAGGATTTAAGCACCTATGCCAGCATCAGTTTCCAGAAGCAGCCAGAGGACCTCCAGTAG

>Cer_si1 [Rhinoceros (Ceratotherium simum simum) Irs1]

ATGGCGAGCCCTCCGGAGACCGACGGCTTCTCGGACGTGCGCAAGGTGGGCTACCTGCGCAAACCCAAGAGCATGCACAAGCGCTTCTTCGTGCTGCGGGCGGCCAGCGAGGCGGGGGGCCCGGCGCGCCTCGAGTACTACGAGAACGAGAAGAAGTGGCGGCACAAGTCGAGCGCCCCCAAACGCTCCATCCCCCTCGAGAGCTGCTTCAACATCAACAAGCGGGCGGACTCCAAGAACAAGCACCTGGTGGCCCTCTACACCCGGGACGAGCACTTTGCCATCGCGGCAGACAGCGAGGCCGAGCAGGACAGCTGGTACCAGGCCCTCCTGCAGCTGCACAACCGTGCCAAGGGCCACCACGACGGGGCCGCGGCCGCCGGGGCGGGAGGCGGCGGGGGCAGCTGCAGCGGCAGCTCGGGCCTCGGCGAGGCTGGGGAGGACTTGAGCTACGGGGACGTGCCCCCAGGACCCGCCTTCAAGGAGGTCTGGCAGGTGATCCTGAAACCCAAGGGCCTGGGTCAGACAAAGAACCTGATTGGCATCTACCGCCTCTGCCTGACGAGCAAGACCATCAGCTTCGTGAAGCTGAACTCGGAGGCGGCGGCCGTGGTGTTGCAACTGATGAACATCAGGCGCTGTGGCCACTCAGAGAACTTCTTCTTCATCGAAGTGGGCCGTTCCGCAGTGACGGGACCCGGGGAGTTCTGGATGCAGGTGGATGACTCGGTGGTGGCCCAGAACATGCACGAGACCATCCTGGAGGCCATGCGGGCTATGAGCGATGAGTTCCGCCCTCGCAGCAAGAGCCAGTCCTCGTCCAACTGCTCCAACCCCATCAGCGTCCCCCTGCGCAGGCACCACCTCAACAACCCCCCGCCCAGCCAGGTGGGGCTGACCCGCCGCTCGCGCACCGAGAGCATCACCGCCACCTCCCCGGCCAGCATGGTGGGCGGGAAGCAGGGCTCCTTCCGTGTCCGTGCCTCCAGCGACGGCGAAGGCACCATGTCCCGCCCGGCTTCAGTGGATGGCAGCCCTGTGAGTCCTAGCACCAACAGGACCCATGCCCACCGGCATCGGGGCAGCTCCCGGCTGCACCCCCCTCTCAACCACAGCCGCTCCATCCCCATGCCTTCTTCTCGCTGCTCGCCCTCCGCCACCAGCCCGGTCAGTCTGTCGTCCAGCAGCACCAGTGGCCATGGCTCCACCTCCGACTGTCTCTTCCCGCGGCGGTCCAGTGCTTCTGTGTCCGGCTCCCCCAGCGATGGCGGTTTCATCTCCTCGGATGAGTATGGCTCCAGTCCCTGCGATTTCCGAAGTTCCTTCCGCAGTGTCACCCCAGATTCCCTGGGCCACACCCCACCGGCCCGCGGTGAGGAGGAGTTGAGCAACTACATCTGCATGGGAGGCAAGGGGGCCTCCACCCTCACCGCCCCCAATGGTCACTACATTTTGCCTCGGGGTGGCAATGGTCACCGCTACATCCCCGGAGCTGGCTTGGGCACGAGCCCAGCCCTGGCTGGGGATGAAGCAGCCAGTGCTGCAGATCTGGATAATCGGTTCCGAAAGCGGACTCACTCTGCTGGCACATCCCCTACCATTTCCCACCAGAAGACCCCATCCCAGTCCTCCGTGGCTTCCATTGAGGAATATACAGAGATGATGCCTGCCTACCCACCAGGAGGTGGCAGTGGAGGCCGGCTGCCTGGCTACCGGCACTCCGCCTTCGTGCCCACCCACTCCTACCCTGAGGAGGGTCTGGAAATGCACCCTTTGGAGCGCCGGGGCCACCACCGCCCAGACACCTCCACCCTCCACACAGATGATGGCTACATGCCCATGTCCCCAGGGGTGGCCCCAGTGCCCAGCAACCGAAAGGGCAGTGGGGACTACATGCCCATGAGCCCCAAGAGCGTGTCTGCCCCACAGCAGATCATCAACCCCATCAGGCGCCATCCCCAGCGAGTGGACCCCAATGGCTACATGATGATGTCCCCAAGCGGCAGCTGCTCCCCTGACATTGGAGGTGGCTCCAGCAGCAGTAGCAGTGCTGTGCCTTCTGGGAGCAGCTATGGGAAGCTATGGACAAATGGTGTAGGGGGCCACCACTCTCATGCCCTGCCGCACCCCAAACTACCCATGGAGAGCAGTGGCAGCAAGCTCTTGTCTTGTACAGGTGACTATATGAACATGTCGCCAGTGGGGGACTCCAACACCAGCAGCCCTTCCGACTGCTACTACGGCCCTGAGGACCCCCAGCACAAGCCAGTCCTCTCCTACTACTCATTGCCAAGGTCCTTTAAGCACACCCAGCCCCCCGGGGAGCTGGAGGAGACTGCCCGGCACCAGCACCTCCGCCTTTCCTCCAGCTCTGGCCGCCTTCTCTATGCTGCAGCAGCAGAAGATTCCTCTTCCTCCACCAGCAGTGACAGCCTGGGTGGGGGGTACTGCGGGGCTAGGCCCGAGCCCGGCCTCCCGCATCATCTCCACCATCAGGTCCTGCAGCCGCATTTGCCTCGAAAGGTGGACACAGCTGCGCAGACCAACAGCCGCCTGGCTCGGCCCACGAGGCTGTCCCTGGGGGATCCCAAGGCCAGCACCTTACCTCGGGCCCGAGAGCAGCAGCAGCCACCCTTGCTGCACCTTCCGGAGCCCAAGAGCCCAGGGGAATATGTGAACATTGAATTTGGGACTGATCAGCCAGGCTACTTATCTGGCCCTGTGGCTTCCCACAGCTTGCCTTCTGTCAGGTGTCCATCCCAGCTCCAGCCAGCTCCCAGAGAGGAAGAGACTGGCACTGAAGAATACATGAACATGGACCTGGGGCCGGGCCGGAGGGCTACCTGGCAGGAGAGTGCTGGGGTCCAGCAGGGCAGAGTGGGCCCTGCACCTCCTGGGTCTGCTAGCATGTGCAGGCCTACTCGGGCAGTGCCCAGCAGCCGGGGTGACTACATGACCATGCAGATGGGTTGTCCCCGTCAGAGCTACGTGGACACCTCGCCAGTTGCCCCCATCAGCTATGCTGACATGCGGACGGGCATTGTCGTGGAGGAGGTGAGCCTTCCCGGGGCCACAGCGGCTGCTCCCTCCTCATCCTCAGCAGCCTCTGCTTCCCCCGCTGCACCTCAAGGAGCGGGGGACCTGGTGGCCTGCTCTTCCCTGCTGGGGGGCCCACAGGGACCCGGGGGTATGAGCGCCTTCACCCGAGTGAACCTCAGCCCCAACCGCAACCAGAGTGCCAAAGTGATCCGTGCGGACCCGCAAGGGTGCCGGAGGCGGCATAGCTCCGAGACCTTCTCCTCGACACCTAGTGCCACCCGGGTGGGCAATACGGTGCCCTTCGGAGCGGGGGCTGTAGTAGGGGGCAGTGGTGGTGGCAGCAGCAGCACTGAGGATGTGAAACGCCACAGCTCTGCTTCCTTTGAGAACGTGTGGCTGAGGCCTGGGGAGCTCGGGGGAGCCCCCAAGGAGCTGGCCCAAGTGTGCGGGGCTGCTGGGGGTTTGGAGAACGGTCTTAACTACATAGACCTGGATTTGGTCAAGGACTTCAAACAGCGCCCTCAGGAGCGCCCCCCTCAACAGCAGCCTCCCCCACCCCCGCCCCCTCATCAGCCTCTGGGCAGCAGTGGGAGCAGCTCCACCAGCCGCTCTAGCGAGGATTTAAGCGCCTATGCCAGCATCAGTTTCCAGAAGCAGCCAGAGGACCTCCAGTAG

>Can_lu1 [Dog (Canis lupus familiaris) Irs1]

ATGGCGAGCCCTCCGGAGACCGACGGCTTCTCGGACGTGCGCAAGGTGGGCTACCTGCGCAAACCCAAGAGCATGCACAAGCGCTTCTTCGTGCTGCGGGCGGCCAGCGAGGCGGGGGGCCCGGCGCGCCTCGAGTACTACGAGAACGAGAAGAAGTGGCGGCACAAGTCGAGCGCCCCCAAACGCTCGATCCCCCTCGAGAGCTGCTTCAACATCAACAAGCGGGCGGACTCCAAGAACAAGCACCTGGTGGCCCTTTACACCCGGGACGAGCACTTTGCCATCGCGGCGGACAGCGAGGCCGAGCAGGACAGCTGGTACCAGGCCCTCCTGCAGCTGCACAACCGGGCCAAGGGCCACCACGACGGCGCCTCGGCCCCCGGGGCGGGAGGCGGCGGGGGCAGCTGCAGCGGCAGCTCGGGCCTCGGGGAGGCCGGCGAGGACTTGAGCTACGGGGACGTGCCCCCGGGACCTGCGTTCAAGGAGGTCTGGCAGGTGATCCTGAAACCCAAGGGCCTGGGGCAGACAAAGAACCTGATTGGCATCTACCGCCTCTGCCTGACCAGCAAGACCATCAGCTTCGTGAAGCTGAACTCCGAGGCGGCGGCCGTGGTGCTGCAGCTGATGAACATCCGACGTTGCGGCCACTCGGAGAACTTCTTCTTCATCGAAGTGGGCCGTTCCGCAGTGACGGGACCCGGCGAGTTCTGGATGCAGGTGGATGACTCCGTGGTGGCCCAGAACATGCACGAGACCATCCTGGAGGCCATGCGGGCCATGAGCGACGAGTTCCGCCCTCGGAGTAAGAGCCAGTCCTCCTCCAACTGCTCCAACCCCATCAGCGTCCCCCTGCGCCGGCACCACCTCAACAACCCCCCTCCCAGCCAGGTGGGGCTGACGCGCCGCTCGCGCACCGAGAGCATCACCGCCACCTCTCCGGCCAGCATGGTGGGCGGGAAGCAGGGCTCCTTCCGTGTGCGCGCGTCCAGCGACGGCGAGGGCACCATGTCCCGCCCGGCCTCGGTGGACGGCAGCCCCGTGAGCCCGAGCACCACCAGGACCCACGCGCACCGGCATCGCGGCAGCTCCCGGCTGCACCCCCCGCTCAACCACAGCCGCTCCATCCCCATGCCTTCCTCTCGCTGCTCGCCTTCCGCCACCAGCCCGGTCAGCCTGTCGTCCAGCAGCACCAGTGGCCACGGCTCCACCTCGGACTGCCTCTTCCCCCGGCGCTCTAGTGCCTCTGTGTCGGGTTCCCCCAGCGACGGTGGTTTCATCTCCTCTGACGAGTACGGCTCGAGTCCCTGCGATTTCCGAAGTTCCTTCCGCAGTGTCACCCCGGATTCCCTGGGCCACACCCCCCCGGCCCGCGGCGAGGAGGAGCTGAGCAACTACATCTGCATGGGAGGCAAAGGGTCCTCCACCCTCACCGCCCCCAACGGTCACTACATTTTGCCTCGGGGTGGCAATGGCCACCGCTACATCCCGGGGGCTGGCTTGGGCACCAGCCCGGCCCTGGCTGCGGATGAAGCGGCCGCTGCGGCCGACCTGGATAACCGGTTCCGAAAGCGGACTCACTCCGCGGGCACATCCCCTACCATTTCCCACCAGAAGACCCCGTCCCAGTCTTCTGTGGCTTCCATTGAGGAGTACACGGAGATGATGCCTGCCTACCCGCCAGGAGGTGGCAGTGGAGGCCGACTGCCTGGCTACCGGCACTCTGCCTTCGTGCCCACCCACTCCTACCCCGAGGAGGGTCTGGAAATGCACCCTCTGGACAGGCGTGGGGGCCACCACCGGCCGGACGCCGCCGCCCTCCACACGGATGATGGCTACATGCCCATGTCCCCGGGAGTGGCACCGGTGCCCAGCAGCCGGAAGGGCAGTGGGGACTATATGCCCATGAGCCCCAAGAGCGTGTCCGCGCCGCAGCAGATCATCAACCCCATTAGACGCCATCCCCAGAGGGTGGACCCCAATGGCTACATGATGATGTCCCCAAGCGGCAGCTGCTCTCCTGACATTGGAGGTGGGCCCGGCAGCAGCAGCAGCGGCAGCGCCGCCCCTTCTGGGAGCAGCTATGGCAAGCTGTGGACAAACGGGGTAGGGGGCCACCACCCTCACGCCCTGCCGCACCCCAAACTCCCCGTGGAGAGCGGGAGTGGCAAGCTCCTGTCTTGTACCGGCGACTACATGAACATGTCGCCGGTGGGGGACTCCAACACCAGCAGCCCCTCCGACGGCTACTACGGCCCAGAGGACCCCCAGCACAAGCCAGTTCTCTCCTACTACTCATTGCCAAGGTCCTTTAAGCACACCCAGCGCCCTGGGGAGCTGGAGGAGAGCGCCCGGCACNAGCACCTCCGCCTCTCCTCCAGCTCGGGTCGTCTTCTCTACGCCGCGACGGCGGAAGATTCCTCCTCCTCCACCAGCAGCGACAGCCTGGGCCCAGGGGGATACTGTGGGGTCAGGCCGGATCCCGGCCTCCCGCATATCCACCATCAGGTCCTGCAGCCTCACCTGCCTCGGAAGGTGGACACGGCCGCGCAGACCAACAGCCGCCTGGCTCGGCCCACGAGGCTGTCCCTGGGGGACCCCAAGGCCAGCACCTTACCTCGGGTTCGAGAGCAGCAGCACCCGCCGCCCCTGCTGCACCCTCCGGAGCCCAAGAGCCCCGGGGAATATGTGAATATTGAGTTCGGGAGCGATCAGCCGGGCTACTTATCGGGGCCGGTGGCTGCCCGCAGCTCGCCTTCTGTCAGGTGCCCACCCCAGCTCCAGCCAGCTCCCCGCGAGGAAGAGACTGGCACCGAGGAGTACATGAACATGGACCTGGGGCCTGGCCGGAGGGCAGCCTGGCAGGAGGGTGCTGGGGTCCAGCCCGGCAGGGTGGGCCCCGCGCCCCCCGGGGCCGCTAGCGTGTGCAGGCCCACCCGGGCAGTGCCCAGCAGCCGGGGCGACTACATGACCATGCAGGTGGGCTGTCCCGGCCAGGGCTACGTGGACACCTCGCCAGTGGCCCCCATCAGCTACGCTGACATGCGGACAGGCATTGTCGTGGAGGAGGCCAGCCTGCCGGGGGCCACAGCGGCCGCCCCCTCCTCGGCCTCGGCAGCCTCGGCTTCCCCCACGGCGCCTCCAAAAGCGGGGGAGCTGGTGGCCCGCTCCTCCCTGCTGGGGGGCCCGCAGGGACCCGGGGGCATGAGCGCCTTCACCCGGGTGAACCTCAGCCCCAACCGCAACCAGAGTGCCAAAGTGATCCGCGCCGACCCGCAGGGGTGCCGGAGGCGGCATAGCTCTGAGACCTTCTCCTCCACGCCCAGTGCCACCCGGGCGGGCAACGCAGTGCCCTTCGGCGGGGGGGCGGCCCTGGGGGGCAGCGGTGGCGGCAGCAGCGCGGAGGATATGAAACGCCACAGTTCGGCTTCCTTTGAGAACGTGTGGCTGAGGCCTGGGGAGCTCGGGGGAGCCCCCAAGGAGCCGGCCCCGCACGCTGGGGCCGCCGGGGGTTTGGAGAATGGGCTTAACTACATAGACCTGGATTTGGTCAAGGACTTCAAACAGTGCTCTCAGGAGCGCCCCCCTCAACCGCAGCCGCCCCCGCCCCCGGCCCCTCATCAGCCTCTGGGCAGCAGTGAGAGCAGTTCAACCAGCCGCTCCAGCGAGGATCTAAGCGCCTATGCCAGCATCAGTTTCCAGAAGCAGCCAGAGGACCTCCAGTAG

>Mus_pu1 [Ferret (Mustela putorius furo) Irs1]

ATGGCGAGCCCTCCCGAGACCGACGGCTTCTCGGACGTGCGCAAGGTGGGCTACCTGCGCAAACCCAAGAGCATGCACAAGCGTTTCTTCGTGCTGCGGGCGGCCAGCGAGGCGGGGGGCCCGGCGCGCCTCGAGTACTACGAGAACGAGAAGAAGTGGCGGCACAAGTCGAGCGCCCCCAAACGCTCGATCCCCCTCGAGAGCTGTTTCAACATCAACAAGCGGGCCGACTCCAAGAACAAGCACCTGGTGGCCCTTTACACCCGGGACGAGCACTTTGCCATCGCGGCGGACAACGAGGCCGAGCAGGACAGCTGGTACCAGGCCCTCCTGCAGCTGCACAACCGGGCCAAGGGCCACCACGACGGGGCGGCAGCCCCCGGGGCAGGAGGAGGTGGGGGCAGCTGCAGTGGCAGCTCTGGCCTCGGGGAGGCTGGGGAGGACTTGAGTTACGGGGACGTGCCCCCAGGACCTGCGTTCAAGGAGGTCTGGCAGGTGATCCTGAAACCCAAGGGCCTGGGTCAGACAAAGAACCTGATTGGGATCTACCGTCTCTGCCTGACCAGCAAGACCATCAGCTTCGTGAAGCTGAACTCGGAGGCGGCGGCTGTGGTGCTGCAGCTGATGAACATCAGACGTTGCGGCCACTCGGAGAACTTCTTCTTCATCGAGGTGGGCCGTTCTGCAGTGACGGGACCTGGGGAGTTCTGGATGCAGGTGGATGACTCTGTGGTGGCCCAGAACATGCACGAGACAATTCTGGAGGCCATGAGGGCCATGAGCGATGAGTTCCGTCCTCGAAGTAAGAGCCAGTCCTCCTCCAACTGCTCCAACCCCATCAGCGTCCCCCTGCGCAGGCACCATCTCAACAACCCCCCGCCCAGCCAGGTGGGGCTGACGCGCCGCTCTCGCACGGAGAGCATCACCGCCACCTCTCCCGCCAGCATGGTGGGCGGCAAGCAGGGCTCCTTCCGTGTGCGCGCGTCCAGTGATGGCGAAGGCACCATGTCCCGGCCGGCCTCGGTGGACGGCAGCCCTGTGAGTCCTAGCACCAACAGGACCCACGCCCACCGGCATCGCGGCAGCTCCCGGCTACACCCTCCTCTCAACCACAGCCGCTCCATCCCCATGCCTTCCTCTCGCTGCTCACCTTCTGCCACTAGCCCGGTCAGTCTGTCGTCCAGCAGCACCAGTGGCCACGGCTCCACCTCGGACTGTCTTTTCCCACGGAGGTCTAGTGCTTCTGTGTCGGGATCCCCCAGCGATGGCGGCTTCATCTCCTCCGATGAGTATGGCTCCAGCCCTTGCGATTTCCGAAGTTCCTTCCGCAGTGTCACCCCGGATTCCCTGGGCCACACCCCACCAGCCCGAGGCGAGGAGGAGCTGAGCAACTACATCTGCATGGGGGGCAAAGGGTCCTCCACCCTTGCCGCCCCCAATGGTCACTACATTTTGCCTCGGGGTGGCAACGGTCACCGCTACATCCCAGGAACTAGTTTGGGCACGAGCCCAGCCCTGGCTGGGGATGAAGCAGCCAGTGCTACAGATCTGGATAATCGGTTCCGAAAGCGGACTCACTCTGCTGGCACGTCCCCTACCATTTCCCACCAGAAGACACCATCCCAGTCCTCTGTGGCTTCCATTGAGGAATACACGGAGATGATGCCTGCCTACCCACCAGGAGGTGGCAGTGGAGGCCGACTGCCTGGCTACCGGCACTCTGCCTTCGTGCCCACCCACTCCTACCCCGAGGAGGGTCTGGAAATGCACCCTTTGGACCGGCGTGGGGGCCACCACCGCCCAGACGCCTCCACTCTCCACACTGATGATGGCTACATGCCCATGTCCCCAGGAGTGGCGCCGGTGCCCAGCAGCCGAAAGGGCAGTGGGGACTACATGCCCATGAGCCCCAAGAGTGTGTCTGCACCCCAGCAGATCATCAACCCAATCAGACGCCATCCCCAGCGAGTGGACCCCAATGGCTACATGATGATGTCCCCGAGCGGCAGCTGCTCCCCTGACATTGGAGGTGGGCCCAGCAGTAGCACTGGCGCTGCCCCTTCTGGGGGCAGCTATGGCAAGCTGTGGACAAATGGGGTTGGGGGGCACCATGGTTCTGCCCTGCCGCATCCCAAGCTGCCTGGGGAGAGTGGCAGCAGCAAGCTCTTGTCTTGTACTGGTGATTACATGAACATGTCACCAGTGGGGGACTCCAACACCAGCAGCCCCTCCGACTGCTACTATGGCCCGGAAGACCCCCAGCACAAGCCAGTTCTCTCCTACTACTCATTGCCAAGGTCCTTTAAGCACACCCAGCGCCCTGGGGAGCTGGAGGAGAGTGCTCGGCACCAACACCTTCGCCTTTCCTCCAGCTCTGGTCGCCTTCTCTATGCTGCAGCGGCGGAAGATTCCTCCTCCTCCACCAGCAGCGACAGCCTGGGAGGGGGATACTGTGGGGTGAGGCCAGATCCTGGCCTCCCACATATCCACCATCAGGTCCTGCAGCCTCATCTGCCTCGAAAGGTGGACACAGCTGCCCAGACCAACAGCCGCCTGGCTCGGCCCACGAGGCTGTCGCTGGGGGATCCCAAGGCCAGCACCTTACCTCGGGTACGAGAGCAGCAGCAACAGCCGACCCTGCTGCACCCTCCAGAGCCCAAGAGCCCGGGGGAATATGTGAATATTGAATTTGGGAGTGATCAGCCGGGTTACTTGTCTGGTCCTGTGGCTTCCCGCAGCTCGCCTTCTGTCAGGTGTCCGTCCCAGCTCCAGCCAGCTCCCCGAGAGGAAGAGACTGGCACAGAGGAATACATGAACATGGACCTGGGGCCGGGCCGGAGGGCGACCTGGCAGGAGAGCACTGGGGTCCAGCCAGGCAGAGTGGGCCCTGCACCTCCTGGAGCTGCTAGCATGTGCCGGCCTACCCGGGCAGTGCCCAGCAGCCGGGGGGACTACATGACCATGCAGATGGGTTGTCCCGGGCAGAGCTACGTGGACACCTCACCAGTTGCCCCCATCAGCTATGCTGACATGAGGACAGGCGTTGTGGAGGAGGCCAGCCTTCCAGGGGCCACAGCAGCAGCTCCCTCATCCTCTGCAGCCTCTGCTTCCCCCACCACTGCGCCTCAGAAAGCAGGGGAGCTGATGGCTCGCTCTTCCCTGCTGGGAGCCCCACAGGGACCTGGGGCCATGAGCGCCTTCACCCGGGTCAACCTCAGTCCCAACCGGAACCAGAGTGCCAAAGTGATCCGTGCGGACCCACAAGGGTGCCGGAGGAGGCATAGCTCTGAGACCTTCTCCTCCACACCTAGTGCCACCCGGGCGGGCAACACGGTGCCCTTTGGAGGGGGGGCAGCTGTAGGGGGCAGCGGTGGTGGCAGCAGCAGCACGGAGGACATGAAACGCCACAGTTCTGCTTCCTTTGAGAATGTGTGGCTGAGGCCTGGGGAGCTTGGGGGAGCTGCCAAGGAGCCGGCCCCAGTGTGTGGGGCTGCTGGGGGTTTGGAGAATGGGCTTAACTACATAGACCTGGATCTGGTCAAGGACTTCAAACAGCGCCCTCAGGAGCGCCCCCCTCAACCGCAGCCTCCCCCGCCCCCACCCCCTCATCAGCCTCTGGGCAGCAGTGAGAGCAGCTCAACCAGCCGCTCCAGTGAGGATTTAAGCGCCTATGCCAGCATCAGTTTCCAGAAGCAGCCAGAGGACCTCCAGTAG

>Fel_ca1 [Cat (Felis catus) Irs1]

ATGGCGAGCCCTCCGGAGACCGAAGGCTTCTCGGACGTGCGCAAGGTGGGCTACCTGCGCAAACCCAAGAGCATGCACAAGCGCTTCTTCGTGCTGCGGGCGGCCAGCGAGGCGGGGGGCCCGGCGCGCCTCGAGTACTACGAGAACGAGAAGAAGTGGCGGCACAAGTCGAGCGCCCCCAAACGCTCGATCCCCCTGGAGAGCTGCTTCAACATCAACAAGCGGGCGGACTCCAAGAACAAGCACCTGGTGGCCCTCTACACCCGGGACGAGCACTTTGCCATCGCGGCGGACAGCGAGGCGGAGCAGGACAGCTGGTACCAGGCCCTCCTGCAGCTGCACAACCGGGCCAAGAGCCACCACGACGGGGCCGCGGCCCCCGGGGCAGGAGGCGGCGGGGGCAGCTGCAGTGGCAGCTCGGGCCTCGGCGAGGCTGGGGAGGACTTGAGCTACGGGGATGTGCCCCCAGGACCTGCGTTCAAGGAGGTCTGGCAGGTGATCCTGAAACCCAAGGGCCTGGGTCAGACAAAGAACCTGATTGGCATCTACCGTCTCTGCCTGACCAGCAAGACCATCAGCTTCGTGAAGCTGAACTCGGAGGCGGCGGCCGTGGTGCTGCAGCTGATGAACATCAGACGCTGCGGCCACTCCGAGAACTTCTTCTTCATCGAAGTGGGCCGTTCCGCGGTGACGGGACCTGGGGAGTTCTGGATGCAGGTGGATGACTCTGTGGTGGCCCAGAACATGCACGAGACAATCCTGGAGGCCATGCGGGCCATGAGCGACGAGTTCCGTCCTCGAAGTAAGAGCCAGTCCTCTTCCAACTGCTCCAACCCCATCAGCGTCCCGCTGCGCAGGCACCACCTCAACAACCCGCCACCCAGCCAGGTGGGGCTGACCCGCCGCTCTCGCACCGAGAGCATCACCGCCACCTCTCCGGCCAGCATGGTGGGCGGGAAGCAGGGCTCCTTCCGCGTGCGCGCGTCCAGCGACGGCGAGGGCACCATGTCCCGCCCGGCCTCGGTGGACGGCAGCCCCGTGAGTCCTAGCACCAACAGGACGCACGCGCACCGGCATCGCGGCAGCTCCCGGCTGCACCCGCCTCTCAACCACAGCCGCTCCATCCCCATGCCTTCGTCCCGCTGCTCGCCTTCTGCCACCAGCCCGGTCAGTCTGTCGTCCAGCAGCACCAGTGGCCACGGCTCCACCTCGGACTGCCTCTTCCCCCGGCGCTCTAGTGCTTCTGTGTCCGGTTCCCCCAGCGATGGCGGTTTCATCTCCTCGGACGAGTACGGCTCCAGTCCTTGCGATTTCCGAAGTTCCTTCCGCAGTGTCACCCCGGATTCCCTGGGCCACACCCCACCGGCCCGCGGGGAGGAGGAGCTGAGCAACTACATCTGCATGGGAGGCAAAGGATCCTCCACCCTCACCGCCCCCAATGGTCACTACATTTTGCCTCGGGGTGGCAATGGTCACCGCTACGTCGCCGGGGCCGGCCTGGGCACCAGCCCAGCCCTGGCCGGGGATGAAGGAGCTAGTGCAGCCGATCTGGATAATCGGTTCCGAAAGCGGACTCACTCGGCCGGCACGTCCCCTACCATTTCCCACCAGAAGACCCCATCGCAGTCCTCGGTGGCTTCCATTGAGGAGTACACGGAGATGATGCCTGCCTACCCGCCAGGAGGTGGCAGCGGAGGCCGCCTGCCCGGCTACCGGCACTCCGCCTTCGTGCCCACCCACTCCTACCCCGAGGAGGGTCTGGAGGTGCACCGGGGCCACCACCGCCCAGACACCTCCTCTCTCCACACTGATGATGGCTACATGCCCATGTCCCCAGGAGTGGCCCCAGTGCCCAGCAGCCGCAAGGGCAGCGGGGACTACATGCCCATGAGCCCCAAGAGCGTGTCTGCGCCACAGCAGATCATCAACCCCATCAGGCGCCATCCCCAGAGGGTGGATCCCAATGGCTACATGATGATGTCCCCTAGCGGCAGCTGCTCTCCTGACATCGGAGGTGGGCCCAGCAGCAGCGGCAGCGGCGCCGCCCCCTCTGGGAGCAGCTACGGGAAGCTGTGGACCAACGGGGTCGGGGGCCACCACCCTCACGCTCTGCCACATCCCAAACTCCCCGTAGAGAGTGGTGGCGGCAAGCTCTTGTCTTGTACTGGTGACTACATAAATATGTCGCCGGTGGGGGACTCCAATACCAGCAGCCCCTCCGACTGCTACTATGGCCAGGAGGACCCCCAGCACAAGCCAGTCCTCTCCTACTACTCGTTGCCAAGGTCCTTTAAGCACACCCAGCGCCCCGGGGAGCTGGAGGAGAGTGCCCGGCACCAGCACCTCCGCCTTTCCTCCAGCTCTGGTCGCCTTCTCTATGCCACCACCGCAGAAGATTCCTCCTCCTCTACCAGCAGCGACAGCCTGGGCGGCGGGTACTGTGGGGTCAGGCCCGATTCTGGCCTCCCGCACATCCACCATCAGGTCCTGCAGCCCCATCTGCCTCGAAAGGTGGACACAGCTGCCCAGACCAACAGCCGCCTGGCTCGGCCCACGAGGCTGTCCCTGGGGGATCCCAAGGCCAGCACCTTACCTCGGGTCCGAGAGCAGCAGCCACAGCCCCCGCTGCTGCACCCTCCGGAGCCCAAGAGCCCAGGGGAATATGTGAATATTGAGTTTGGGGGGGATCAGCCGGGCTACTTATCTGGTCCCGTGGCTTCCCGCGGCTCGCCTTCTGTCAGGTGCCCATCCCAGCTCCAGCCAGCCCCCAGGGAGGAAGAGACTGGCACTGAAGAGTACATGAACATGGACCTGGGGCCAGGCAGGAGGGCCACCTGGCAGGAGGGTGCCGGCGGCCAGCCAGGCAGAGTGGGCCCCACACCTCCTGGGGCGGCCAGCGTGTGCAGGCCCACCCGGGCCGTGCCCAGCAGCCGCGGTCACTACATGACCATGCAGATGGGCTGTCCCGGTCAGAGCTATGTGGACACCTCACCAGTTGCCCCCATCAGCTATGCTGACATGCGGACAGGCCTCGTTGCGGAGGAGGCGAGCCTTCCGGGGACCACGGCCGCCGCTCCCTCCTCATCCTCGGCAGCCTCGGCTTCCCCCACTGCGCCTCAAAAAGCAGGGGAGCTGGTGGCCCGCTCTTCCCTGCTGGGGGGCCCGCAGGGACCCGGAGGCATGAGCGCCTTCACCAGGGTGAACCTCAGCCCCAACCGGAACCAGAGTGCCAAAGTGATCCGTGCGGACCCGCAAGGGTGCCGGAGGCGGCACAGCTCTGAGACCTTCTCCTCGACACCCAGTGCCACCCGGGCGGGCAACACGGTGCCCTTCGGAGGGGGCGCTGCAGGAGGGGGCAGCGGTGGTGGCAACGGCAGCGCCGAGGAGGTGAAACGCCACAGTTCTGCTTCCTTCGAGAACGTGTGGCTGAGGCCTGGGGAGCTCGGGGGAGCCCCCAAGGAGCTGGCCCAAGTGTGCGGGGCCGCCGGGGGTTTGGAGAACGGGCTTAACTACATAGACCTGGATTTGGTCAAGGACTTCAAACAGCGCCCTCAGGAGCGCCCCCCTCAACCACAGCCGCCCCCTCCCCCGCCCCCTCATCAGCCTCTGGGCAGCAGCGAGGGCAGCTCCACCCGCCGCTCCAGCGAGGATTTAAGCGCCTATGCCAGCATCAGTTTCCAGAAGCAGCCAGAGGACCTCCAGTAG

>Lep_we1 [Weddell seal (Leptonychotes weddellii) Irs1]

ATGGCGAGCCCTCCGGAGACCGACGGCTTCTCGGACGTGCGCAAGGTGGGCTACCTGCGCAAACCCAAGAGCATGCACAAGCGCTTCTTCGTGCTGCGGGCGGCCAGCGAGGCGGGGGGCCCGGCGCGCCTCGAGTACTACGAGAACGAGAAGAAGTGGCGGCACAAGTCGAGCGCCCCCAAACGCTCGATCCCCCTCGAGAGCTGCTTCAACATCAACAAGCGGGCGGACTCCAAGAACAAGCACCTGGTGGCCCTTTACACCCGGGACGAGCACTTTGCCATCGCGGCGGACAGCGAGGCCGAGCAGGACAGCTGGTACCAGGCCCTCCTGCAGCTGCACAACCGGGCCAAGGGCCACCACGACGGGGCCGCGGCCCCCGGGGCAGGAGGCGGCGGGGGCAGCTGCAGCGGCAGCTCGGGCCTTGGGGAAGCTGGGGAGGACTTGAGCTACGGGGACGTGCCCCCAGGACCTGCGTTCAAGGAGGTCTGGCAGGTGATCCTGAAACCCAAGGGCCTGGGTCAGACAAAGAACTTGATTGGCATCTACCGTCTCTGCCTGACCAGCAAGACCATCAGCTTCGTGAAGCTGAACTCGGAGGCGGCGGCCGTGGTGCTGCAGCTGATGAACATCAGACGCTGCGGCCACTCAGAGAACTTCTTCTTCATCGAAGTGGGCCGTTCCGCAGTGACAGGACCTGGGGAGTTCTGGATGCAGGTGGATGACTCTGTGGTGGCCCAGAACATGCACGAGACCATCCTGGAGGCCATGCGGGCCATGAGCGATGAGTTCCGTCCTCGAAGTAAGAGCCAGTCCTCCTCCAACTGCTCCAACCCCATCAGCGTCCCCCTGCGCAGGCACCACCTCAACAACCCCCCGCCCAGCCAGGTGGGGCTGACGCGCCGCTCGCGGACCGAGAGCATCACCGCCACCTCACCGGCCAGCATGGTGGGCGGCAAGCAGGGCTCCTTCCGTGTGCGCGCGTCCAGCGACGGCGAAGGCACCATGTCCCGCCCGGCCTCGGTGGACGGCAGCCCCGTGAGTCCCAGCACCAACAGGACCCACGCCCACCGGCATCGCGGCAGCTCCCGGCTGCACCCCCCTCTCAACCACAGCCGCTCCATCCCCATGCCTTCTTCCCGCTGCTCGCCTTCCGCCACCAGCCCGGTCAGCCTGTCGTCCAGCAGCACCAGCGGCCACGGCTCCACCTCGGACTGTCTCTTCCCGCGGCGGTCTAGTGCTTCTGTGTCGGGTTCCCCCAGCGATGGCGGCTTCATCTCCTCCGATGAGTACGGCTCCAGCCCTTGCGATTTCCGAAGTTCCTTCCGCAGTGTCACCCCGGATTCCCTGGGCCACACGCCACCAGCCCGCGGTGAGGAGGAGCTGAGCAACTACATCTGCATGGGAGGCAAAGGGTCCTCCACCCTCACCGCCCCCAATGGTCACTACATTTTGCCTCGGGGTGGCAACGGTCACCGCTGCGTCCCAGGAGCTGGCTTGGGCACGAGCCCAGCCCTGGCTGGGGATGAAGCAGCCAGTGCTGTAGATCTGGATAATCGGTTCCGCAAGCGGACTCACTCCGCGGGCACATCCCCTACCATTTCCCACCAGAAGACCCCATCCCAGTCGTCTGTGGCTTCCATTGAGGAATATACAGAGATGATGATGCCTCCCTACCCGCCAGGAGGTGGCAGTGGAGGCCGGCTGCCTGGCTACCGGCACTCTGCCTTCGTGCCCACCCACTCCTACCCTGAGGAGGGTCTGGAAATGCACCCTTTGGACAGGCGTGGGGGCCACCACCGCCCAGACTCCTCCACTCTCCACACGGATGATGGCTACATGCCCATGTCCCCAGGAGTGGCGCCAGTGCCCAGCAGCCGAAAGGGCAGTGGCGACTATATGCCCATGAGCCCCAAGAGCGTGTCGGCGCCACAGCAGATCATCAACCCGATCAGACGCCATCCCCAGAGAGTGGACCCCAATGGCTACATGATGATGTCCCCAAGCAGCAGCTGCTCCCCGGACACTGGAGGCGGACCCAGCAGCAGCGCCGCCCCTTCTGGGAGCAGCTACGGCAAGCTGTGGACAAATGGGGTAGGGGGCCACCACGCTCACGCCCTGCCGCATCCCAAACTGCCCATGGAGAGCGGGAGTGGCAAGCTCTTGTCTTGTACCGGTGACTACATGAACATGTCGCCAGGGGGGGGCTCCAACACCAGCAGCCCCTCCGACTGCTACTACGGCCCGGAGGACCCCCAGCACAAGCCAGTTCTCTCCTACTACTCATTGCCAAGGTCCTTTAAGCACACCCAGCGGCCTGGGGAGCTGGAGGAGGGTGCCCGGCACCAGCACCTCCGCCTTTCCTCCAGCTCTGGCCGCCTTCTCTATGCTACAACGGCGGAAGATTCCTCCTCGTCCACCAGCAGCGACAGCCTGGGCGGGGGATACTGTGGGGTGAGGCCGGATCCTGGCCTCCCGCATGTCCACCATCAGGTCCTGCAGCCCCATCTGCCTCGAAAGGTGGACACAGCTGCCCAGACCCACAGCCGCCTGGCTCGGCCCACGAGGCTGTCCCTGGGGGACCCCAAGGCCAGCACCTTACCTCGGGTCAGGGAGCAACAGCAGCTCCCGCCCCTGCTGCGCCCTCCCCCGGAGCCCAAGAGCCCTGGCGAATATGTGAATATTGAATTTGGGAGTGAACAGCCAGCTTATTTATCTGGTCCTGTGGCTTCCCGCAGCTCGCCTTCTGTCAGGTGCCCATCCCAGCTCCCGCCAGCTCCCCGAGAGGACGAGACTGGCACCGAGGAATACATGAACATGGACCTGGGGCCAGGCCGGAGGGCGACCTGGCAGGAGAGCGCTGGGGTCCAGCCAGGCAGTGCGGGCCCTGCCCCTCCCGGTGCTGCTAGCATGTGCAGGCCTACGCGGGCAGTGCCCGGCAGCCGGGGGGACTACATGACCATGCAGATGGGCTGTCCTGGGCAGGGCTACGTGGACACCTCGCCAGTTGCCCCTGTCAGCTATGCCGACATGCGGACAGGCATTGTTGTGGAGGAGGCCAGCCTTCCAGGGGCCACAGCGGCCGCTCCCTCCTCATCCTCTGCAGCCTCTGCTTCCCCCACCGCGCCTCAAAAAGCAGGGGAGCTGGTGGCCCGCTCTTCCCTTCTGGGGGGCCCCCAGGGACCTGGGGGCGTGAGCGCCTTCACCCGGGTGAACCTCAGTCCCAATCGTACGCAGAGTGCCAAAGTGATCCGTGCGGACCCACAAGGGTGCCGGAGGCGGCATAGCTCTGAGACCTTCTCGTCCACGCCGAGTGCCACCCGGGCGGGCAACGCGGTGCCCTTTGGAGGGGGGGCTGCTGTAGGGGGCAGCGGTGCTGGCAGCAGCAGCACAGAGGATATGAAACGCCACAGTTCTGCTTCCTTTGAGAATGTGTGGCTGAGGCCTGGGGAGCTCGGGGGAGCCCCCAAGGAGCCGGCCCAAGTGTGCGGGGCTGCTGGGGGTTTGGAGAATGGGCTTAACTACATAGACCTGGATTTGGTCAAGGACTTCAAACAGCGCCCTCAGGAGCGCCCCCCTCAACCGCCCCTGCCCCCGCCCCCTCATCAGCCTCTGGGCAGCAGGGAGAGCCGCTCAAGCAGCCGCTCCAGCGAGGATTTAAGCGCGTATGCCAGCCTCAGTTTCCAGAAGCAGCCAGAGGACCTCCAGTAG

>Odo_ro1 [Pacific walrus (Odobenus rosmarus divergens) Irs1]

ATGGCGAGCCCTCCGGAGACCGACGGCTTCTCGGACGTGCGCAAGGTGGGCTACCTGCGCAAACCCAAGAGCATGCACAAGCGCTTCTTCGTGCTGCGGGCGGCCAGCGAGGCGGGGGGCCCGGCGCGCCTCGAGTACTACGAGAACGAGAAGAAGTGGCGGCACAAGTCGAGCGCCCCCAAACGCTCGATCCCCCTCGAGAGCTGCTTCAACATCAACAAGCGGGCGGACTCCAAGAACAAGCACCTGGTGGCCCTTTACACCCGGGACGAGCACTTTGCCATCGCGGCGGACAGCGAGGCCGAGCAGGACAGCTGGTACCAGGCCCTCCTGCAGCTGCACAACCGGGCCAAGGGCCACCACGACGGGGCCGCGGCCCCCGGGGCAGGAGGCGGCGGGGGCAGCTGCAGCGGCAGCTCGGGCCTTGGGGAGGCTGGGGAGGACTTGAGCTACGGGGACGTGCCCCCAGGACCTGCGTTCAAGGAGGTCTGGCAGGTGATCCTGAAACCCAAGGGCCTGGGTCAGACAAAGAACCTGATTGGCATCTACCGTCTCTGCCTGACCAGCAAGACCATCAGCTTCGTGAAGCTGAACTCGGAGGCGGCGGCCGTGGTGCTGCAGCTGATGAACATCAGACGCTGCGGCCACTCAGAGAACTTCTTCTTCATCGAAGTGGGCCGTTCCGCAGTGACAGGACCTGGGGAGTTCTGGATGCAGGTGGATGACTCTGTGGTGGCCCAGAACATGCACGAGACCATCCTGGAGGCCATGCGGGCCATGAGCGATGAGTTCCGTCCTCGAAGTAAGAGCCAGTCCTCCTCCAACTGCTCCAACCCCATCAGCGTTCCCCTGCGCAGGCACCACCTCAACAACCCCCCGCCCAGCCAGGTGGGGCTGACGCGCCGCTCGCGGACTGAGAGCATCACCGCCACCTCACCGGCCAGCATGGTGGGCGGCAAGCAGGGCTCTTTCCGTGTGCGTGCGTCCAGCGACGGCGAAGGCACCATGTCCCGCCCGGCCTCGGTGGACGGCAGCCCCGTGAGTCCCAGCACCAACAGGACCCACGCCCACCGGCATCGCGGCAGCTCCCGGCTGCACCCCCCTCTCAACCACAGCCGCTCCATCCCCATGCCTTCCTCCCGCTGCTCGCCTTCCGCCACCAGCCCGGTCAGCCTGTCGTCCAGCAGCACCAGCGGCCACGGCTCCACCTCGGACTGTCTCTTCCCGCGGCGGTCTAGTGCTTCTGTGTCGGGTTCCCCCAGCGATGGCGGCTTCATCTCCTCCGATGAGTACGGCTCCAGCCCTTGCGATTTCCGAAGTTCCTTCCGCAGTGTCACCCCGGATTCCCTGGGCCACACGCCACCGGCCCGCGGTGAGGAGGAGCTGAGCAACTACATCTGCATGGGAGGCAAAGGGTCCTCCACCCTCACCGCCCCCAATGGTCACTACATTTTACCTCGGGGTGGCAACGGTCACCGCTGCGTCCCAGGAGCTGGCTTGGGCACAAGCCCAGCCCTGGTTGGGGATGAAGCAGCCAGTGCTGCAGATCTGGATAATCGGTTCCGGAAGCGGACTCACTCCGCGGGCACATCCCCTACCATTTCCCACCAGAAGACCCCATCCCAGTCATCTGTAGCGTCCATTGAGGAATACACAGAGATGATGATGCCTGCCTACCCGCCAGGAGGTGGCAGTGGAGGCCGGCTGCCTGGCTACCGGCACTCTGCCTTCGTGCCCACCCACTCCTACCCTGAGGAGGGCCTGGAAATGCACCCTTTGGACAGGCGTGGGGGCCACCACCGCCCAGACTCCTCCGCTCTCCACACGGATGATGGCTACATGCCCATGTCGCCAGGAGTGGCGCCAGTGCCCAGCAGCCGAAAGGGCAGTGGCGACTATATGCCCATGAGCCCCAAGAGCGTGTCGGCGCCGCAGCAGATCATCAACCCTATCAGACGCCATCCCCAGAGAGTGGACCCCAATGGCTACATGATGATGTCCCCAAGCAGCAGCTGCTCCCCGGACACTGGAGGCGGACCCAGCAGCAGCGCTGCCCCTTCTGGGAGCTGCTACGGCAAGCTGTGGACAAATGGGGTCGGGGGCCACCACACTCACGCCCTGCCGCATCCCAAACTGCCCATGGAGAGCGGGAGTGGCAAGCTCTTGTCTTGTACTGGTGACTACATGAACATGTCGCCAGTGGGGGACTCCAACACCAGCAGCCCCTCCGACTGCTACTACGGCCCGGAGGACCCCCAGCACAAGCCAGTTCTCTCCTACTACTCATTGCCAAGGTCCTTTAAGCACACCCAGCGGCCTGGGGAGCTGGAGGAGGGTGCTCGGCACCTCCGCCTTTCCTCCAGCTCTGGCCGCCTTCTCTATGCTACAACGGCGGAAGATTCCTCCTCGTCCACCAGCAGCGACAGCCTGGGCGGGGGATACTGTGGGGTGAGGCCGGATCCTGGCCTCCCACATGTCCACCATCAGGTCCTGCAGCCCCATCTGCCTCGAAAGGTGGACACAGCTGCCCAGACCCACAGCCGCCTGGCTCGGCCCACGAGGCTGTCCCTGGGGGACCCCAAGGCCAGCACCTTACCTCGGGTCCGGGAACAACAGCAGCTCCCGCCCCTGCTGCGCCCCCCAGAGCCCAAGAGCCCCGGCGAATATGTGAATATTGAATTTGGGAGTGATCAGCCAGCTTACTTATCTGGTCCTGTGGCTTCCCGCAGCTCACCTTCTGTCAGGTGCCCATCCCAGCTCCAGCCAGCTCCCCGAGAGGATGAGACTGGCACTGAGGAATACATGAACATGGACCTGGGGCCAGGCCGGAGGGCGACCTGGCAGGAGAGCGCTGGGGTCCAGCCAGGCAGCGTGGGCCCTGCACCACCCGGGGCTGCTAGCATGTGCAGGCCTACCCGGGCAGTGCCCAGCAGCCGGGGGGACTACATGACCATGCAGATGGGTTGTCCTGGGCAGGGCTACGTGGACACCTCACCAGTTGCCCCCGTCAGCTATGCCGACATGCGGACAGGCATTGTTGTGGAGGAGGCCAGCCTTCCAGGGGCCACAGCGGCTGCTCCCTCCTCATCCTCTGCAGCCTCTGCTTCTCCCACCACGCCTCAAAAAGCAGGGGAGCTGGTGGCCCGCTCTTCCCTGCTGGGGGGCCCCCAGGGACCTGGGGGCATGAGCGCCTTCACCCGGGTGAACCTCAGTCCCAACCGCACGCAGAGTGCCAAAGTGATCCGTGCGGACCCACAAGGGTGCCGGAGGCGGCATAGCTCTGAGACCTTCTCCTCCACACCAAGTGCCACCCGGGCAGGCAACGCGGTGCCCTTTGGAGGGGGGGCTGCTGTAGGGGGCAGCAGTGCTGGTAGCAGCAGCACGGAGGATATGAAACGCCACAGTTCTGCTTCCTTTGAGAATGTGTGGCTGAGGCCTAGCGAGCTCGGGGGAGCCCCCAAGGAGCCGGCCCAAGTGTGCGGGGCTGCTGGGGGTTTGGAGAATGGGCTTAACTACATAGACCTGGATTTGGTCAAGGACTTCAAACAGCGCCCGCAGGAGCGCCCCCCTCAACCGCCCTCGCCCCCGCCCCCTCATCAGCCTCTGGGCAGCAGGGAGAGCCGCTCAAGCAGCCGCTCCAGCGAGGATTTAAGCACCTATGCCAGCATCAGTTTCCAGAAGCAGCCAGAGGACCTCCAGTAG

>Myo_lu1 [Little brown bat (Myotis lucifugus) Irs1]

ATGGCGAGCCCTACGGAGACCGAGGGCTTCTCGGACGTGCGCAAGGTGGGCTACCTGCGCAAACCCAAGAGCATGCACAAGCGCTTCTTCGTGCTGCGGGCGGCCAGCGAGGCGGGGGGCCCGGCGCGCCTGGAGTACTATGAGAACGAGAAGAAGTGGCGGCACAAGTCGAGCGCCCCCAAACGCTCGATCCCCCTCGAGAGCTGTTTCAACATCAACAAGCGGGCTGACTCCAAGAATAAGCACCTGGTGGCCCTCTACACCCGGGACGAGCACTTTGCGATCGCAGCGGACAGCGAGGCCGAGCAGGACAGCTGGTACCAGGCCCTCCTGCAGCTGCATAACCGCGCCAAGAGCCACCACGACGGGGCTTCGGCCCCCGGGACAGGAGGCGGCGGGGGCAGCTGCAGCGGCAGCTCTGGCGTTGGCGAGGCCGGGGAGGACCTGAGCTTCGGGGACAGCAACCCAGGGCCCGCCTTCAAAGAGGTCTGGCAGGTGGTCTTGAAACCCAAGGGCCTGGGTCAGACAAAGAACCTGATTGGTATCTACCGCCTCTGCCTGACCAGCAAGACCATCAGCTTCGTGAAGCTGAACACAGAGGCGGCCGCAGTGGTGCTGCAGCTGATGAACATCCGGCGCTGTGGTCACTCGGAGAACTTCTTCTTCATCGAAGTGGGCCGTTCCGCAGTGACCGGCCCCGGGGAGTTCTGGATGCAGGTGGAGGACTCGGTGGTGGCCCAGAGCATGCACGAGACCATCCTGGAGGCCATGCGGGCGATGAGCGATGAATTCCGGCCTCGCAGCAAGAGCCAGTCTTCCTCCAACTGCTCCAACCCCATCAGTGTCCCGCTGCGCAGGCACCACCTCAACAACCCTCCACCCAGCCAGGTGGGGCTGACCCGCCGCTCACGCACCGAGAGCATCACGGCCACCTCTCCAGCCAGCCTGGTGGGCGGCAAGCAGGGCTCCTTCCGTGTCCGCGCCTCCAGCGACGGAGAAGGCACCATGTCCCGCCCGGCCTCGGTGGACGGCAGCCCTGTGAGTCCTAGCACCAACAGGACCCACGCCCACAGGCATCGGGGCTGCTCGCGGCTGCACCCCCCACTCAACCACAGCCGATCCATACCCATGCCTTCTTCTCGCTGCTCGCCTTCTGCCACCAGCCCTGTCAGTCTGTCGTCCAGCAGCACCAGTGGCCATGGCTCCACCTCTGACTGTCTTTTCCCGCGGAGATCTAGTGCTTCTGTGTCTGGCTCCCCTAGTGATGGCGGTTTCATCTCTTCCGATGAGTATGGCTCCAGTCCCTGCGATTTCCGAAGTTCCTTCCGCAGTGTCACCCCAGATTCTCTGGGCCACACCCCACCGGCCCGCGGGGAGGAGGAGCTGAGCAACTACATCTGCATGGGAGGCAAGGGCGCCTCCACTCTCGCCGCCCCCAATGGTCACTACATTTTGCCTCGGAGTGGCAATGGTCACCGCTACCTCCCAGCCACGGGCTTGGGCACGAACTCGGCCCTGGCTGGGGATGAAGCAGCCAGCGCTGCAGATCTGGATAATCGGTTTCGCAAGCGGACTCACTCAGCTGGCACCTCCCCCACCATTTCCCACCAGAAGACCCCATCGCAGTCCTCCGTGGCTTCCATTGAGGAATATACAGAGATGATGCCTGCCTGCCCACCAGGAGGTGGCAGTGGAGGCCGACTGCCCGGCTACAGGCACTCCGCCTTCCTGCCCACCCAGTCCTACCCTGAGGAGGGTCTGGAAATGCACCCCCTGGAGCGCCGCGGAGGCCACAGCCGCCCAGACACCTCCACCCTCCACACGGATGATGGCTACATGCCCATGACCCCAGGAGTGGCCCCAGTGCCCGGCAGCCGGAAAGGCAGTGGGGACTACATGCCTATGAGCCCCAAGAGTGTGTCTTCCCCGCAGCAGATCATCAACCCCCTCAGACGCCATCCCCAGAGAGTGGACCCCAATGGCTACATGATGATGTCCCCAAGTGGCAGCAGCTCCCCAGATACTGGGGGTGGGCCCAGCAGCAGCAACAGTGCTGCCCCTTCTAGGAGTAGCTATGGGAAGCTGTGGACAAATGGGGTTGGGGGCCACCACGCCCACCCCCTGCCACCCTCCAAACTACCCTCGGAGAGCAGTGGTGGGAAGCTCTTGCCTTGTACAGGTGACTACATGAACATGTCACCGGTGGGGGACTCCAACACCAGCAGCCCCTCTGACTGCTACTGCGGCCCTGAGGACCCCCAGCACAAGTCAGTTCTCTCCTACTGCTCATTGCCAAGGTCCTTTAAGCACACCCAGCGCCCGGGGGACCTGGAGGAGACCGCCCGGCACCAGCACCTCCGCCTTTCCTCCAGCTCCGGTCGCCTTCTCTATGCTGCAGCAGCCGAAGATTCCTCCTCCTCCACCAGCAACGACAGCCTGGGTGGGGGATACTGTGGGGTGAGGCCCGAGCCCAGCCTCCCGCAACATCTCCACCATCAGGTCCTGCAGTCCCATCTGCCTCGAAAGGTGGACTCAGCTGCGCAGACCAACAGCCGCCTGGCTCGGCCCACGAGGCTTTCCCTGGGGGACCCCAAGGCCAGCACCTTACCTCGGGCTCGAGAGCAGCAGCAGCCGGCCCTACTGCTTCCTCCAGAGCCCAAGAGCCCAGGGGAATATGTGAATATCGAATTTGGGAGTGATCAGCCTGGCTACTTATCTGGTCCCGTGGCATCCCACAGCTCGCCTTCTGTCAGGTGTCCATCCCAGCTCCAGCCAGCTCCCAGGGAGGAAGAGTCTGGCACTGAAGAGTACATGAACATGGACCTGGGGCCTGGCCGGAGGGCTACCTGGCAGGAGAGCCCTGTGGTCCAGCTGAGCAGAGTGGGTCCTGCACCTCCAGGGACTGCCAGCACATGCAGGCCTACCCGGGCAGTGCCTAGCACTAGCACTCGGGGTGACTACATGACCATGCAGATGGGTTGTCCCCATCAGAGCTACGTGGACACCTCTCCAGTTGCCCCTGTCAGCTATGCTGATATTCGGACAGGCATTGTGGAGGAGGCGAGCCTTCCCGGGGCTATAGCGGCTGCTCCCTCCTCATCCTCAACAGCCTCTGCTTCCCCCACTGCGCCTCAAGGAGCAGGGGAGCTGGTGGCCCCGCAGGGGTCCGGGGGCACGAGTGCCTTCACCCGGGTGAACCTCAGTCCCAGTGGCAACCAGAGTGCCAAAGTGATCCGTGCAGACCCGCAAGGGTGCCGGAGGCGGCATAGCTCCGAGACCTTCTCCTCCATGCCTAGTACCCCCCGGGCCGGCAACATGGTGCCCTCAGGGGGGGCTGCAGTAGGGGGCAGCAGTGGAGGCAGCAGCAGCATTGAGGATGTGAAACGTCACAGCTCTGCTTCATTTGAGAACGTGTGGCTGAGGCCTGGGGAGCTCGGGGGAGCACCCAAAGAGCAGGCTCAAGTGTGTGGGGCTGCAGGGGGTTTGGAGAATGGTCTGAACTACATAGACCTGGATTTGGTCAAGGACTTTAAACAGCACCCCCAGGAGCACCCCCGTCAAGTGCAGCATCCCCAGCCCCCAACCCTTCGTCATCCTCCGGGCAGCAGTGAGAGCAGCTCTACCAGCCGCTCCAGGGAGGATGTAAGCGCCTATGCCAGCATCAGTTTTCAGAAGCAGCCAGAGGACCTCCAGTAG

>Pte_al1 [Black flying fox (Pteropus alecto) Irs1]

ATGGCGAGCCCTCCGGAGACCGACGGCTTCTCGGACGTGCGCAAGGTGGGCTACCTGCGCAAACCCAAGAGCATGCACAAGCGCTTCTTCGTGCTGCGGGCGGCCAGCGAGGCGGGGGGCCCGGCGCGCCTCGAGTACTATGAGAACGAGAAGAAGTGGCGGCACAAGTCGAGCGCCCCCAAACGCTCGATCCCCCTCGAGAGCTGTTTCAACATCAACAAGCGGGCGGACTCCAAGAACAAGCACCTGGTGGCCCTCTACACCCGGGACGAGCACTTTGCCATCGCGGCAGACAGCGAGGCTGAGCAGGACAGCTGGTACCAGGCCCTCCTCCAGCTGCACAACCGTGCCAAGGGCCACCACGACGGGGCCTCTGCCCTTGGGGCAGGAGGTGGCGGGAGCAGCTGCAGTGGCAGCTCGGGCCTTGGCGAGGCTGGGGAGGACTTGAGCTACGGGGACGGGCCCCCAGGACCCGCGTTCAAGGAGGTCTGGCAAGTGATCCTGAAGCCCAAGGGCCTGGGTCAGACAAAGAACCTGATTGGCATCTACCGCCTCTGCCTGACCAGCAAGACCATCAGCTTCGTGAAGCTGAACTCAGAGGCGGCGGCCGTGGTGCTGCAACTGATGAACATCCGGCGCTGTGGCCACTCAGAGAACTTCTTCTTCATCGAAGTGGGCCGTTCCGCAGTGACAGGACCTGGGGAGTTCTGGATGCAGGTGGATGACTCGGTGGTGGCCCAGAACATGCACGAGACAATCCTGGAGGCCATGCGAGCCATGAGCGATGAGTTCCGCCCTCGCAGCAAAAGCCAGTCCTCCTCCAACTGCTCCAACCCCATTACTGTCCCCCTGCGCAGGCATCACCTCAACAACCCGCCGCCTAGCCAGGTGGGGCTGACCCGCCGCTCGCGCACCGAGAGCATCACCGCCACCTCTCCCGCCAGCATGGTGGGCGGGAAGCAGGGCTCCTTCCGTGTCCGCGCCTCCAGCGATGGCGAAGGCACCATGTCCCGCCCGGCTTCGGTGGACGGCAGCCCCGTGAGTCCCAGCACCAACAGGACCCACGCCCACCGGCATCGGGGCAGCTCCCGGCTGCACCCCCCGCTCAACCACAGCCGTTCCATCCCCATGCCTTCTTCTCGCTGCTCGCCTTCGGCCACGAGCCCGGTCAGCCTGTCGTCTAGCAGCACCAGTGGCCACGGCTCCACCTCTGACTGCCTGTTCCCGCGGAGATCTAGTGCTTCTGTGTCGGGTTCCCCCAGCGATGGCGGTTTCATCTCTTCGGATGAATATGGCTCCAGTCCCTGCGATTTCCGAAGTTCCTTCCGCAGTGTCACCCCGGATTCCCTGGGCCACACTCCACCCGCCCGCGGAGAGGAAGAGCTGAGCAACTACATCTGCATGGGAGGCAAGGGGGCCTCCACTCTTACCGCCCCCAATGGGCACTACATTTTGCCTCGGGGTGGCAACGGTCACCGTTACATCCCAGGAGCCGGCTTGGGCATGAGCCCTGCCCTGGCTGGCGATGAAGCAGCCAGTGCTGCAGATCTGGATAACCGGTTCCGAAAGCGGACTCACTCTGCGGGTACATCTCCCACCATTTCCCACCAGAAGACCCCATCCCAGTCCTCCGTGGCTTCCATTGAGGAATATACAGAGATGATGCCTGCCTACCCGCCAGGAGGTGGCAGTGGAGGCCGGCTACCGGGGTACCGGCACTCCGCCTTCGTGCCCACCCAGTCCTACCCGGAGGAGGGTCTGGAAGGGCACCCCTTGGAGCGCCGTGGAGGCCACAACCGCCCAGACACCTCCACTCTCCACACTGATGATGGCTACATGCCGATGTCCCCAGGAGTGGCCCCAGTGCCCAGCAGCCGAAAAGGCAGTGGGGACTACATGCCCATGAGCCCCAAGAGTGTGTCTGCCCCGCAGCAGATCGTCAGACGCCATCCCCAGAGAGTGGATCCCAACGGCTACATGATGATGTCCCCGAGTGGCAGCTGCTCCCCTGACATTGGCGGTGGGCCCAGCAGCAGCAGCAGCAGCAGCGCCGCCCCTTCTGGGAGTAGTTATGGGAAGCCGTGGACTAATGGGGTAGGGGGCCACCACTCCCACGCCCCGCCGCACCCGAAACTACCCGCGGAGAGCAGCGGTAGCAAGCTCTTGTCTTGTACAGGTGACTACATGAACATGTCGCCAGTGGGAGACTCCAATACCAGCAGCCCCTCTGACTGCTACTATGGCCCTGAGGACCCCCAGCACAAGCCAGTTCTCTCCTACTACTCATTGCCAAGGTCCTTTAAGCACACCCAGCGCCCCGGGGAGCTGGAGGAGACTGCCCGGCACCAGCACCTCCGTCTTTCCTCCAGCTCTGGTCGCCTTCTCTATGCTGCAGCAGCCGAAGACTCCTCTTCCTCCACCAGCAGCGACAGTCTGGGCGGGGGACACTGCGGGGCAAGGCCTGAGCCCGGCCTCCCGCATCATCTCCACCATCAGGTCCTGCAGTCCCATCTGCCTCGAAAGGTGGACACGGCTGCGCAGACCAACAGCCGCCTGGCTCGGCCCACCAGACTGTCCCTGGGGGACCCCAAGGCCAGCACCTTACCTCGGGCCCGAGAGCAGCAGCAGCGGCCAGCCCTGCTGCACCCTCCGGAGCCCAAGAGCCCAGGGGAATATGTGAATATCGAATTTGGGTGTGATCAGCCAGGCTACTTATGTGGCCCCGTGGCATCCCACAGCTCGCCTTCCATCAGGTGTCCATCCCAGCTCCAGCCAGCTCCCAAAGAGGAAGAGACTGGCACTGAAGAGTATATGAACATGGACCTGGGGCCGGGCCGGAGGGCCACCTGGCAGGAGAGCCCTGGGGTCCAGCCTGGCAGAGTGGGCCCTGCACCTCCAGGGACTGCTAACATGTCCAGGCCGACCCGGGCAGTGCCTAGCGGCCGGGGTGACTACATGACCGTGCAGGTGGGTCGTCCCCGGCAGAGCTATGTGGACACCTCTCCAGTTGCCCCTGTCAGCTATGCTGACATTCGGACGGGCATCGTGGAGGAGGCCAGGCTTCCTGGGGCCCCAGCGGCTGCCCCCTCCTCATCCTCAGCAGCCCCTGCTTCACCCACTGTGCCTCAAGGAGCGGGGGAGCCGGAGGCTCGCTCTTCCCTGCTGGGGGGCCCGCAGGGACCCGGGGGCATGAGCGCCTTCACCAGGGTGAACCTTAGTCCCAGCTGCAACCAGAGTGCCAAAGTGATCCGCGCAGACCCACAAGCGTGCCGGAGGCGGCACAGCTCCGAGACCTTCTCCTCTACTCCTAATGCCACCAGGGCGGGCAACACGGTGCCCTTCGGAGGGGGGGCTGCAGTCGGGGGCACCGGTGGTGGCAGCGGCAGCAGCACTGAGGACGTGAAACGCCACAGCTCTGCTTCCTTTGAGAATGTGTGGCTGAGGCCTGGGGAGCTTGGGGGAGCCCCCAAGGAGCCGGCTCAGGTGTGTGGGACTGCGGGGGGTTTGGAGAATGGTCTCAACTACATAGACCTGGATTTGGTCAAGGACGTCAAACAGCGCCCTCAGGAGCGCCCCCCTCAATCGCAGCCTCCCGCACCCCCGCCCCAGCATCAGCCAATGGGCAGCAGTGAGAGCAGTTCCACCAACCGCTCCAGTGAGGATGTAAGCGCCTATGCCAGCATCAGCTTCCAGAAGCAGCCAGAGGACCTCCAGTAG

>Pte_va1 [Large flying fox (Pteropus vampyrus) Irs1]

ATGGCGAGCCCTCCGGAGACCGACGGCTTCTCGGACGTGCGCAAGGTGGGCTACCTGCGCAAACCCAAGAGCATGCACAAGCGCTTCTTCGTGCTGCGGGCGGCCAGCGAGGCGGGGGGCCCGGCGCGCCTCGAGTACTATGAGAACGAGAAGAAGTGGCGGCACAAGTCGAGCGCCCCCAAACGCTCGATCCCCCTCGAGAGCTGTTTCAACATCAACAAGCGGGCGGACTCCAAGAACAAGCACCTGGTGGCCCTCTACACCCGGGACGAGCACTTTGCCATCGCGGCAGACAGCGAGGCTGAGCAGGACAGCTGGTACCAGGCCCTCCTCCAGCTGCACAACCGTGCCAAGGGCCACCACGACGGGGCCTCTGCCCTTGGGGCAGGAGGTGGCGGGAGCAGCTGCAGTGGCAGCTCGGGCCTTGGCGAGGCTGGGGAGGACTTGAGCTACGGGGACGGGCCCCCAGGGCCCGCGTTCAAGGAGGTCTGGCAAGTGATCCTGAAGCCCAAGGGCCTGGGTCAGACAAAGAACCTGATTGGCATCTACCGCCTCTGCCTGACCAGCAAGACCATCAGCTTCGTGAAGCTGAACTCAGAGGCGGCGGCCGTGGTGCTGCAACTGATGAACATCCGGCGCTGTGGCCACTCAGAGAACTTCTTCTTCATCGAAGTGGGCCGTTCCGCAGTGACAGGACCTGGGGAGTTCTGGATGCAGGTGGACGACTCGGTGGTGGCCCAGAACATGCACGAGACAATCCTGGAGGCCATGCGAGCCATGAGCGATGAGTTCCGCCCTCGCAGCAAAAGCCAGTCCTCCTCCAACTGCTCCAACCCCATTACTGTCCCCCTGCGCAGGCATCACCTCAACAACCCACCGCCTAGCCAGGTGGGGCTGACCCGCCGCTCGCGCACGGAGAGCATCACCGCCACCTCTCCCGCCAGCATGGTGGGCGGGAAGCAGGGCTCCTTCCGTGTCCGCGCCTCCAGCGACGGCGAAGGCACCATGTCCCGCCCGGCTTCGGTGGACGGCAGCCCCGTGAGTCCCAGCACCAACAGGACCCACGCCCACCGGCATCGGGGCAGCTCCCGGCTGCACCCCCCGCTCAACCACAGCCGTTCCATCCCCATGCCTTCTTCTCGCTGCTCGCCTTCGGCCACCAGCCCGGTCAGCCTGTCGTCTAGCAGCACCAGTGGCCACGGCTCCACCTCTGACTGCCTGTTCCCGCGGAGATCTAGTGCTTCTGTGTCGGGTTCCCCCAGCGATGGCGGTTTCATCTCTTCGGATGAGTATGGCTCCAGTCCCTGCGATTTCCGAAGTTCCTTCCGCAGTGTCACCCCGGATTCCCTGGGCCACACTCCACCCGCCCGCGGAGAGGAAGAGCTGAGCAACTACATCTGCATGGGAGGCAAGGGGGCCTCCACTCTTACCGCCCCCAATGGGCACTACATTTTGCCTCGGGGTGGCAACGGTCACCGTTACATCCCAGGAGCCGGCTTGGGCATGAGCCCTGCCCTGGCTGGCGATGAAGCAGCCAGTGCTGCCGATCTGGATAACCGGTTCCGAAAGCGGACTCACTCTGCGGGGACATCTCCCACCATTTCCCACCAGAAGACCCCATCCCAGTCCTCCGTGGCTTCCATTGAGGAATATACAGAGATGATGCCTGCCTACCCACCAGGAGGTGGCAGTGGAGGCCGGCTGCCGGGGTATCGGCACTCCGCCTTCGTGCCCACCCAGTCCTACCCGGAGGAGGGTCTGGAAGGGCACCCCTTGGAGCGCCGTGGGGGCCACAACCGCCCAGACACCTCCACTCTCCACACTGATGATGGCTACATGCCGATGTCCCCAGGAGTGGCCCCAGTGCCCAGCAGCCGAAAAGGCAGTGGGGACTACATGCCCATGAGCCCCAAGAGTGTGTCCGCCCCGCAGCAGATCGTCAGACGCCATCCCCAGAGAGTGGATCCCAACGGCTACATGATGATGTCCCCGAGTGGCAGCTGCTCCCCTGACATTGGCGGTGGGCCCAGCAGCAGCAGCAGCAGCAGCGCCGCCCCTTCTGGGAGTAGTTATGGGAAGCCGTGGACTAATGGGGTAGGGGGCCACCACTCCCACGCCCCGCCGCACCCGAAACTACCCGCGGAGAGCAGCGGTAGCAAGCTCTTGTCTTGTACAGGTGACTACATGAACATGTCGCCAGTGGGAGACTCCAATACCAGCAGCCCCTCTGACTGCTACTACGGCCCTGAGGACCCCCAGCACAAGCCAGTTCTCTCCTACTACTCATTGCCAAGGTCCTTTAAGCACACCCAGCGCCCCGGGGAGCTGGAGGAGACTGCCCGGCACCAGCACCTCCGTCTTTCCTCCAGCTCTGGTCGCCTTCTCTATGCCGCAGCAGCCGAAGACTCCTCTTCCTCCACCAGCAGCGACAGTCTGGGCGGGGGACACTGCGGGGCAAGGCCTGAGCCCGGCCTCCCGCATCATCTCCACCATCAGGTCCTGCAGTCCCATCTGCCTCGGAAGGTGGACACGGCTGCGCAGACCAACAGCCGCCTGGCTCGGCCCACCAGACTGTCCCTGGGGGACCCCAAGGCCAGCACCTTACCTCGGGCCCGAGAGCAGCAGCAGCGGCCAGCCCTGCTGCACCCTCCGGAGCCCAAGAGCCCAGGGGAATATGTGAATATCGAATTTGGGTGTGATCAGCCAGGCTACTTATGTGGCCCCGTGGCATCCCACAGCTCGCCTTCCATCAGGTGTCCATCCCAGCTCCAGCCAGCTCCCAAAGAGGAAGAGACTGGCACTGAAGAGTATATGAACATGGACCTGGGACCGGGCCGGAGGGCCACCTGGCAGGAGAGCCCTGGGGGCCAGCCTGGCAGAGTGGGCCCTGCACCTCCAGGGACTGCTAACATGTCCAGGCCGACCCGGGCAGTGCCTAGCGGCCGGGGTGACTACATGACCGTGCAGGTGGGTCGTCCCCGGCAGAGCTATGTGGACACCTCTCCAGTTGCCCCTGTCAGCTATGCTGACATTCGGACGGGCATCGTGGAGGAGGCCAGGCTTCCTGGGGCCCCAGCGGCGGCTCCCTCCTCATCCTCAGCAGCCCCTGCTTCACCCACTGTGCCTCAAGGAGCGGGGGAGCCGGAGGCTCGCTCTTCCCTGCTGGGGGGCCCGCAGGGACCCGGGGGCATGAGCGCCTTCACCAGGGTGAACCTTAGTCCCAGCTGCAACCAGAGTGCCAAAGTGATCCGCGCAGACCCACAAGCGTGCCGGAGGCGGCACAGCTCCGAGACCTTCTCCTCTACTCCTAATGCCACCAGGGCGGGCAACACGGTGCCCTTCGGAGGGGGGGCTGCAGTCGGGGGCAGCGGTGGTGGCAGCAGCAGCAGCACTGAGGACGTGAAACGCCACAGCTCTGCTTCCTTTGAGAATGTGTGGCTGAGGCCTGGGGAGCTTGGGGGAGCCCCCAAGGAGCCGGCTCAGGTTTGTGGGACTGCGGGGGGTTTGGAGAATGGTCTCAACTACATAGACCTGGATTTGGTCAAGGACGTCAAACAGCGCCCTCAGGAGCGCCCCCCTCAATCGCAGCCTCCCGCACCCCCGCCCCAGCATCAGCCAATGGGCAGCAGTGAGAGCAGTTCCACCAACCGCTCCAGCGAGGATGTAAGCGCCTATGCCAGCATCAGCTTCCAGAAGCAGCCAGAGGACCTCCAGTAG

>Myo_br1 [Brandt's bat (Myotis brandtii) Irs1]

ATGGCGAGCCCTACGGAGACCGAGGGCTTCTCGGACGTGCGCAAGGTGGGCTACCTGCGCAAACCCAAGAGCATGCACAAGCGCTTCTTCGTGCTGCGGGCGGCCAGCGAGGCGGGGGGCCCGGCGCGCCTGGAGTACTATGAGAACGAGAAGAAGTGGCGGCACAAGTCGAGCGCCCCCAAACGCTCGATCCCCCTCGAGAGCTGTTTCAACATCAACAAGCGGGCTGACTCCAAGAATAAGCACCTGGTGGCCCTCTACACCCGGGACGAGCACTTTGCGATCGCAGCGGACAGCGAGGCCGAGCAGGACAGCTGGTACCAGGCCCTCCTGCAGCTGCATAACCGCGCCAAGAGCCACCACGACGGGGCTTCGGCCCCCGGGACAGGAGGCGGCGGGGGCAGCTGCAGTGGCAGCTCTGGCGTTGGCGAGGCTGGGGAGGACCTGAGCTTCGGGGACAGCAACCCAGGGCCCGCATTCAAAGAGGTCTGGCAGGTGGTCTTGAAACCCAAGGGCCTGGGTCAGACAAAGAACCTGATTGGTATCTACCGCCTCTGCCTGACCAGCAAGACCATCAGCTTCGTGAAGCTGAACACAGAGGCGGCCGCAGTGGTGCTGCAGCTGATGAACATCCGGCGCTGTGGTCACTCGGAGAACTTCTTCTTCATCGAAGTGGGCCGTTCCGCAGTGACCGGCCCGGGGGAGTTCTGGATGCAGGTGGAGGACTCGGTGGTGGCCCAGAGCATGCACGAGACCATCCTGGAGGCCATGCGGGCGATGAGCGATGAATTCCGGCCTCGCAGCAAGAGCCAGTCTTCCTCCAACTGCTCCAACCCCATCAGTGTCCCGCTGCGCAGGCACCACCTCAACAACCCTCCACCCAGCCAGGTGGGGCTGACCCGCCGCTCACGCACCGAGAGCATCACGGCCACCTCTCCAGCCAGCCTGGTGGGCGGGAAGCAGGGCTCCTTCCGTGTCCGCGCCTCCAGCGACGGGGAAGGCACCATGTCCCGCCCGGCCTCGGTGGACGGCAGCCCCGTGAGTCCCAGCACCAACAGGACCCACGCCCACCGGCATCGGGGCTGCTCGCGGCTACACCCCCCACTCAACCACAGCCGATCCATCCCCATGCCTTCTTCTCGCTGCTCGCCTTCGGCCACGAGCCCTGTCAGTCTGTCGTCCAGCAGCACCAGTGGCCATGGCTCCACCTCTGACTGTCTTTTCCCGCGGAGATCTAGTGCTTCTGTGTCTGGTTCCCCTAGTGATGGCGGTTTCATCTCTTCCGATGAGTATGGCTCCAGTCCCTGCGATTTTCGAAGTTCCTTCCGCAGTGTCACCCCAGATTCCCTGGGCCACACCCCACCGGCCCGCGGGGAGGAGGAGCTGAGCAACTACATCTGCATGGGAGGCAAGGGCGCCTCCACTCTCGCCGCCCCCAATGGTCACTACATTTTGCCTCGGGGTGGCAATGGTCACCGCTACCTCCCAGCCACGGGCTTGGGCACGAACTCGGCCCTGGCTGGGGATGAAGCAGCCAGCGCTGCAGATCTGGATAATCGGTTTCGCAAGCGGACTCACTCAGCTGGCACCTCCCCCACCATTTCCCACCAGAAGACCCCATCGCAGTCCTCCGTGGCTTCCATTGAGGAATATACAGAGATGATGCCTGCCTGCCCACCAGGAGGTGGCAGTGGAGGCCGACTGCCCGGCTACCGGCACTCCGCCTTCCTGCCCACCCAGTCCTACCCTGAGGAGGGTCTGGAAATGCACCCCCTGGAGCGCCGCGGAGGCCACAGCCGCCCAGACACCTCCACCCTCCACACGGATGATGGCTACATGCCCATGACCCCAGGAGTGGCTCCAGTGCCTGGCAGCCGGAAAGGCAGTGGGGACTACATGCCTATGAGCCCCAAGAGTGTGTCTTCCCCGCAGCAGATCATCAACCCCCTCAGACGCCATCCCCAGAGAGTGGACCCCAATGGCTACATGATGATGTCCCCAAGTGGCAGCAGCTCCCCAGATACTGGGGGTGGGCCCAGCAGCAGCAACAGTGCTGCCCCTTCTAGGAGTAGCTATGGGAAGCTGTGGACAAATGGGGTTGGGGGCCACCACGCCCACCCCCTGCCACCCTCCAAACTACCCTCGGAGAGCAGTGGTGGGAAGCTCTTGCCTTGTACAGGTGACTACATGAACATGTCACCGGTGGGGGACTCCAACACCAGCAGCCCCTCTGACTGCTACTGCGGCCCTGAGGACCCCCAGCACAAGTCAGTTCTCTCCTACTGCTCATTGCCAAGGTCCTTTAAGCACACCCAGCGCCCGGGGGACCTGGAGGAGACCGCCCGGCACCAGCACCTCCGCCTTTCCTCCAGCTCTGGTCGCCTTCTCTATGCTGCAGCAGCCGAAGATTCCTCCTCCTCCACCAGCAACGACAGCCTGGGTGGGGGATACTGTGGGGTGAGGCCCGAGCCCAGCCTCCCGCAACATCTCCACCATCAGGTCCTGCAGTCCCATCTGCCTCGAAAGGTGGACTCAGCTGCGCAGACCAACAGCCGCCTGGCTCGGCCCACGAGGCTTTCCCTGGGGGACCCCAAGGCCAGCACCTTACCTCGGGCTCGAGAGCAGCAGCAGCCGGCCCTACTGCTTCCTCCAGAGCCCAAGAGCCCAGGGGAATATGTGAATATCGAATTTGGGAGTGATCAGCCTGGCTACTTATCTGGTCCCGTGGCATCCCACAGCTCGCCTTCTGTCAGGTGTCCATCCCAGCTCCAGCCAGCTCCCAGGGAGGAAGAGTCTGGCACTGAAGAGTACATGAACATGGACCTGGGGCCTGGCCGGAGGGCTACCTGGCAGGAGAGCCCTGTGGTCCAGCCGAGCAGAGTGGGTCCTGCACCTCCAGGGACTGCCAGCACATGCAGGCCTACCCGGGCAGTGCCTAGCACTAGCACTCGGGGTGACTACATGACCATGCAGATGGGTTGTCCCCATCAGAGCTACGTGGACACCTCTCCAGTTGCCCCTGTCAGCTATGCTGATATTCGGACAGGCATTGTGGAGGAGGCGAGCCTTCCCGGGGCTATAGCGGCTGCTCCCTCCTCATCCTCAACAGCCTCTGCTTCCCCCACTGCGCCTCAAGGAGCAGGGGAGCTGGTGGCCCCGCAAGGGTCCGGGGGCACGAGTGCCTTCACCCGGGTGAACCTCAGTCCCAGTGGCAACCAGAGTGCCAAAGTGATCCGTGCAGACCCGCAAGGGTGCCGGAGGCGGCATAGCTCCGAGACCTTCTCCTCCATGCCTAGTACCCCCCGGGCCGGCAACATGGTGCCCTCAGGGGGGGCTGCAGTAGGGGGCAGCAGTGGTGGCAGCAGCAGCATTGAGGATGTGAAACGTCACAGCTCTGCTTCATTTGAGAACGTGTGGCTGAGGCCTGGGGAGCTCGGGGGAGCACCCAAAGAGCAGGCTCAAGTGTGTGGGGCTGCAGGGGGTTTGGAGAATGGTCTGAACTACATAGACCTGGATTTGGTCAAGGACTTCAAACAGCACCCCCAGGAGCACCCCCGTCAAGTGCAGCATCCCCAGCCCCCAACCCTTCGTCATCCTCCGGGCAGCAGTGAGAGCAGCTCTACCAGCCGCTCCAGGGAGGATGTAAGCGCCTATGCCAGCATCAGTTTTCAGAAGCAGCCAGAGGACCTCCAGTAG

>Ept_fu1 [Big brown bat (Eptesicus fuscus) Irs1]

ATGGCGAGCCCTACGGAGACCGAGGGCTTCTCGGACGTGCGCAAGGTGGGCTACCTGCGCAAACCTAAGAGCATGCACAAGCGCTTCTTCGTGCTGCGGGCGGCCAGCGAGGCGGGGGGCCCGGCGCGCCTGGAGTACTACGAGAATGAGAAGAAGTGGCGGCACAAGTCGAGCGCCCCCAAACGCTCGATCCCCCTCGAGAGCTGTTTCAACATCAACAAGCGGGCTGACTCCAAGAATAAGCACCTGGTGGCCCTCTATACCCGGGACGAGCACTTTGCTATCGCAGCGGACAGCGAGGCCGAGCAGGACAGCTGGTACCAGGCCCTCCTGCAGCTGCATAACCGTGCCAAGAGCCACCACGATGGGGCTTCGGCCCCCGGGACAGGAGGCGGCGGGGGCAGCTGCAGTGGCAGCTCCGGCGTGGGCGAGGCTGGGGAGGACCTGAGCTTCGGGGACAGCAACCCGGGGCCCGCATTCAAGGAGGTCTGGCAGGTGGTCTTGAAACCCAAGGGCCTGGGTCAGACAAAGAACCTGATTGGTATCTACCGCCTCTGCCTGACCAGCAAGACCATCAGCTTCGTGAAGCTGAACACAGAGGCGGCTGCAGTGGTGCTGCAGCTGATGAACATCCGGCGCTGTGGTCACTCGGAGAACTTCTTCTTCATCGAAGTGGGCCGTTCCGCCGTGACCGGACCCGGGGAGTTCTGGATGCAGGTGGATGACTCGGTGGTGGCCCAGAGCATGCACGAGACAATCCTGGAGGCCATGCGGGCTATGAGCGATGAATTCCGGCCTCGCAGCAAGAGCCAGTCTTCCTCCAACTGCTCCAACCCCATCAGTGTCCCCCTGCGCAGGCACCACCTAAACAACCCTCCGCCCAGCCAGGTGGGGCTGACCCGGCGTTCACGCACGGAGAGCATCACGGCCACCTCTCCAGCCAGCCTGGTGGGCGGGAAGCAGGGCTCCTTCCGCGTCCGTGCCTCCAGCGACGGGGAAGGCACCATGTCCCGCCCGGCTTCGGTGGACGGCAGCCCCGTGAGTCCTAGCACCAACAGGACGCACGCCCACCGGCATCGGGGCTGCTCGCGGCTGCACCCCCCACTCAACCACAGCCGATCCATCCCCATGCCTTCTTCTCGCTGCTCGCCTTCGGCCACCAGCCCTGTCAGCCTGTCCTCAAGCAGCACCAGTGGCCACGGCTCCACCTCGGACTGTCTTTTCCCGCGGAGATCGAGTGCTTCTGTGTCTGGTTCCCCCAGCGATGGCGGTTTCATCTCTTCGGATGAGTATGGCTCCAGCCCCTGCGATTTCCGAAGTTCCTTCCGCAGTGTCACCCCAGATTCCCTGGGCCACACCCCACCGGCCCGCGGGGAGGAGGAGCTGAGCAACTACATCTGCATGGGAGGCAAGGGGGCCTCCACTCTCGCCGCCCCCAATGGCCACTACATTTTGCCTCGGGGTGGCAATGGTCACCGCTACCTCCCGGCCGCTGGCCTGGGCACGAACTCGGCCCTGGCTGGGGATGAAGCAGCGGGTGCTGCAGATCTGGATAATCGGTTTCGCAAGCGGACTCACTCGGCTGGCACTTCCCCCACCATTTCCCACCAGAAGACCCCATCGCAGTCCTCCGTGGCTTCCATTGAGGAATATACAGAGATGATGCCTGCCTGCCCACCAGGAGGTGGCAGTGGAGGCCGACTGCCCGGCTACCGGCACTCCGCCTTCCTGCCCACCCAGTCCTACCCCGAGGAGGGTCTGGAAATGCACCCCCTGGAGCGCCGCGGAGGCCACAGCCGCCCCGACACCTCCACCCTCCACACGGATGACGGCTACATGCCCATGACCCCAGGAGTGGCCCCAGTGCCTGGCAGCCGGAAAGGCAGTGGGGACTACATGCCTATGAGCCCCAAGAGTGTGTCTTCCCCGCAGCAGATCATCAACCCCCTCAGGCGCCATCCCCAGAGAGTGGACCCCAATGGCTACATGATGATGTCCCCAAGTGGCAGCTGCTCCCCGGACACTGCGGGTGGGCCCAGCAGCAGCAGCAGCAACAGTGCTGCCCCTTCTAGGAGTAGCTATGGGAAGCTGTGGACAAATGGGGTCGGGGGCCACCACGCCCACCCCCTGCCACCCTCCTCCAAACTCCCCTCGGAGAGCAGTGGTGGGAAGCTCTTGCCTTGTACAGGTGACTACATGAACATGTCACCGGTGGGGGACTCCAACACCAGCAGCCCCTCTGACTGCTACTGCGGCCCTGAGGACCCCCAGCACAAGCCAGTTCTCTCCTACTACTCATTGCCAAGGTCCTTTAAGCACACCCAGCGGCCGGGGGACCTGGAGGAGACCGCCCGGCACCAGCACCTCCGCCTTTCCTCCAGCTCTGGTCGCCTTCTCTATGCTGCAGCAGCCGAAGATTCCTCTTCCTCCACCAGCAACGACAGCCTGGGTGGGGGATACTGTGGGGTGAGGCCCGAGCCCAGCCTCCCGCATCATCTGCACCATCAGGTCCTGCAGTCCCATCTGCCTCGAAAGGTGGACTCAGCTGCGCAGACCAACAGCCGCCTGGCTCGGCCCACGAGGCTTTCCCTGGGGGACCCCAAGGCCAGCACCTTACCCCGGGCTCGAGAGCAGCAGCAGCCGGCCCTCCTGCTTCCTCCGGAGCCCAAGAGCCCAGGGGAATATGTGAATATAGAATTTGGGAGTGATCAGCCTGGCTACTTATCTGGTCCCGTGGCATCCCACAGCTCACCTTCGGTCAGGTGTCCATCCCAGCTCCAGCCGGCTCCCAGGGAGGAAGAGTCTGGCACTGAAGAGTACATGAACATGGACCTGGGGCCTGGCCGGAGGGCTACCTGGCAGGAGAGCCCTGTGGTCCAGCCGAGCAGAGTGGGTCCTGCACCTCCAGGGACTGCCAGCACCTGCAGGCCTACCCGGGCAGTGCCTAGCACTAGCACTCGGGGTGACTACATGACCATGCAGATGGGGTGTCCCCATCAGAGCTATGTGGACACCTCCCCGGTTGCCCCTGTCAGCTATGCTGATATTCGGACAGGCATTGTGGAGGAGGCGAGCCTTCCCGGGGCTGCGGCGGCTGCTCCCTCCTCATCCTCTACAGCCTCTGCTTCCCCCGCTGCGCCTCAAGGAGCAGGGGAGCTGGTGGCCCCGCAGGGGTCCGGGGGCACGAGTGCCTTCACCCGGGTGAACCTCAGTCCCAGTGGCAACCAGAGTGCCAAAGTGATCCGTGCAGACCCGCAAGGGTGCCGGAGGCGGCATAGCTCCGAGACCTTCTCCTCCATGCCTAGTACCCCCCGGGCCGGCAACACGGTGCCCTCAGCGGGGGCTGCGGTAGGGGGCAGCAGTGGTGGCAGCAGCAGCATTGAGGATGTGAAGCGTCACAGCTCTGCTTCATTTGAGAACGTGTGGCTGAGGCCTGGGGAGCTCGGGGGAGCACCCAAAGAGCAGGCTCCAGTGTGTGGGGCTGCAGGGGGTTTGGAGAATGGTCTGAACTACATAGACCTGGATTTGGTCAAGGACTTCAAACAGCACCCCCAGGAGCACCCCCCTCAAGGGCAGCATCCCCAACCCCCAACCCTTCATCGTCCTCCGGGCAGCAGTGAGAGCAGCTCCTCTAGCCGCTCCAGGGAGGATGTAAGCGCCTACGCCAGCATCAGTTTTCAGAAGCAGCCAGAGGACCTCCAGTAG

>Myo_da1 [David's myotis (Myotis davidii) Irs1]

ATGGCGAGCCCTACGGAGACCGAGGGCTTCTCGGACGTGCGCAAGGTGGGCTACCTGCGCAAACCCAAGAGCATGCACAAGCGCTTCTTCGTGCTGCGGGCGGCCAGCGAGGCGGGGGGCCCGGCGCGCCTGGAGTACTACGAGAACGAGAAGAAGTGGCGGCACAAGTCGAGCGCCCCCAAACGCTCGATCCCCCTCGAGAGCTGTTTCAACATCAACAAGCGGGCTGACTCCAAGAATAAGCACCTGGTGGCCCTCTACACCCGGGACGAGCACTTTGCGATCGCAGCGGACAGCGAGGCCGAGCAGGACAGCTGGTACCAGGCCCTCCTGCAGCTGCATAACCGCGCCAAGAGCCACCACGACGGGGCCTCGGCCCCCGGGACAGGAGGCGGCGGGGGCAGCTGCAGTGGCAGCTCTGGCCTTGGCGAGGCTGGGGAGGACCTGAGCTTCGGGGACAGCAACCCCGGGCCCGCCTTCAAAGAGGTCTGGCAGGTGGTCTTGAAACCCAAGGGCCTGGGTCAGACAAAGAACCTGATTGGTATCTACCGCCTCTGCCTGACCAGCAAGACCATCAGCTTCGTGAAGCTGAACACAGAGGCGGCCGCAGTGGTGCTGCAGCTGATGAACATCCGGCGCTGTGGTCACTCGGAGAACTTCTTCTTCATCGAAGTGGGCCGTTCCGCGGTGACGGGCCCCGGGGAGTTCTGGATGCAGGTGGAGGACTCGGTGGTGGCCCAGAGCATGCACGAGACCATCCTGGAGGCCATGCGGGCGATGAGCGATGAATTCCGGCCTCGCAGCAAGAGCCAGTCTTCCTCCAACTGCTCCAACCCCATCAGTGTCCCGCTGCGCAGGCACCACCTCAACAACCCTCCACCCAGCCAGGTGGGGCTGACCCGCCGCTCACGCACCGAGAGCATCACGGCCACCTCTCCGGCCAGCCTGGTGGGCGGGAAGCAGGGCTCCTTCCGTGTCCGCGCCTCCAGCGACGGGGAAGGCACCATGTCCCGCCCGGCCTCAGTGGACGGCAGCCCCGTGAGTCCTAGCACCAACAGGACCCACGCCCACCGTCATCGGGGCTGCTCGCGGCTGCACCCCCCACTCAACCACAGCCGATCCATCCCCATGCCTTCTTCTCGCTGCTCGCCTTCGGCCACTAGCCCTGTCAGTCTGTCGTCCAGCAGCACCAGTGGCCATGGCTCCACCTCTGACTGTCTTTTCCCGCGGAGATCTAGTGCTTCTGTGTCTGGTTCCCCTAGTGATGGCGGTTTCATCTCTTCGGATGAGTATGGCTCCAGTCCCTGCGATTTCCGAAGTTCCTTCCGCAGTGTCACCCCAGATTCCCTGGGCCACACCCCACCGGCCCGCGGGGAGGAGGAGCTGAGCAACTACATCTGCATGGGAGGCAAGGGGGCCTCCACTCTCGCCGCCCCCAATGGTCACTACATTTTGCCTCGGGGTGGCAATGGTCACCGCTACCTCCCAGCCACGGGCTTGGGCACGAACTCGGCCCTGGCTGGGGATGAAGCAGCCGGCGCTACAGATCTGGATAATCGGTTTCGAAAGCGGACTCACTCAGCTGGCACCTCCCCCACCATTTCCCACCAGAAGACCCCATCGCAGTCCTCCGTGGCTTCCATTGAGGAATATACAGAGATGATGCCTGCCTGCCCACCAGGAGGTGGCAGTGGAGGCCGACTGCCCGGCTACCGCCACTCCGCCTTCCTGCCCACCCAGTCCTACCCTGAGGAGGGTCTGGAAATGCCCCCCCTGGAGCGCCGCGGAGGCCACAGCCGCCCAGACTCCTCCACCCTCCACACGGATGATGGCTACATGCCCATGACCCCAGGAGTGGCCCCAGTGCCTGGCAGCCGGAAAGGCAGTGGGGACTACATGCCTATGAGCCCCAAGAGTGTGTCTTCCCCGCAGCAGATCATCAACCCCCTCAGACGCCATCCCCAGAGAGTGGATCCCAATGGCTACATGATGATGTCCCCAAGTGGCAGTAGCTCCCCCGATACTGGGGGTGGACCCAGCAGCAGCAACAGTGCTGCCCCTTCTAGGAGTAGCTATGGGAAGCTGTGGACAAATGGGGTTGGGGGCCACCACTCCCACCCCCTGCCACCCTCCAAACTACCCTCGGAGAGCAGTGGTGGGAAGCTCTTGCCTTGTACAGGTGACTACATGAACATGTCACCGGTGGGGGACTCCAACACCAGCAGCCCCTCTGACTGCTACTGCGGCCCTGAGGATCCCCAGCACAAGTCCGTTCTCTCCTACTGCTCATTGCCAAGGTCCTTTAAGCACACCCAGCGCCCGGGGGACCTGGAGGAGACCGCCCGGCACCAGCACCTCCGCCTTTCCTCCAGCTCTGGTCGCCTTCTCTATGCTGCAGCAGCCGAAGATTCCTCCTCCTCCACCAGCAACGACAGCCTGGGTGGGGGATACTGTGGGGTGAGGCCCGAGGCCAGCCTCCCGCATCATCTCCACCATCAGGTCCTGCAGTCCCATCTGCCTCGAAAGGTGGACTCAGCTGCGCAGACCAACAGCCGCCTGGCTCGGCCCACGAGGCTTTCCCTGGGGGACCCCAAGGCCAGCACCTTACCTCGGGCTCGAGAGCAGCAGCAGCCGGCCCTACTGCTTCCTCCAGAGCCCAAGAGCCCAGGGGAATATGTGAATATCGAATTTGGGAGTGATCAGCCTGGCTACTTATCTGGTCCCGTGGCATCCCACAGCTCGCCTTCTGTCAGGTGTCCGTCCCAGCTCCAGCCAGCTCCCAGAGAGGAAGAGTCTGGCACTGAAGAGTACATGAACATGGATCTGGGGCCTGGCCGGAGGGCTACCTGGCAGGAGAGCCCTGTGGTCCAGCCGAGCAGAGTGGGTCCTGCACCTCCAGGGACTGCCAGCACATGCAGGCCTACCCGGGCAGTGCCTAGCACTAGCACTCGGGGTGACTACATGACCATGCAGATGGGTTGTCCCCATCAGAGCTACGTGGACACCTCTCCAGTTGCCCCTGTCAGCTATGCTGATATTCGGACAGGCATTGTGGAGGAGGCGAGCCTTCCCGGGGCTATAGCGGCTGCTCCCTCCTCATCCTCAACAGCCTCTGCTTCCCCCACTGCGCCTCAAGGAGCAGGGGAGCTGGCGGCCCCGCAGGGGTCCGGGGGCACGAGTGCCTTCACCCGGGTGAACCTCAGTCCCAGTGGCAACCAGAGTGCCAAAGTGATCCGGGCAGACCCGCAAGGGTGCCGGAGGCGGCATAGCTCCGAGACCTTCTCCTCCATGTCTAGTACCCCCCGGGCCGGCAACACGGTGCCCTCAGGGGGGGCTGCAGTAGGGGGCAGCAGTGGTGGCAGCAGCAGCATTGAGGATGTGAAACGTCACAGCTCTGCTTCATTTGAGAACGTGTGGCTGAGGCCTGGGGAGCTCGGGGGAGCACCCAAAGAGCAGGCTCAAGTGTGTGGGGCTGCAGGGGGTTTGGAGAATGGTCTGAACTACATAGACCTGGATTTGGTCAAGGACTTCAAACAGCACCTCCAGGAGCACCCCCCTCAAGTGCAGCATCCCCAGCCCCCAACCCTTCGTCATCCTCCGGGCAGCAGTGAGAGCAGCTCTACCAGCCGCTCCAGGGAGGATGTAAGCGCCTATGCCAGCATCAGTTTTCAGAAGCAGCCAGAGGACCTCCAGTAG

>Eri_eu1 [European hedgehog (Erinaceus europaeus) Irs1]

ATGGCGAGCCCCCCGGACACCGACGGCTTCTCGGACGTGCGCAAGGTGGGCTACCTGCGCAAACCCAAGAGCATGCACAAGCGCTTCTTCGTGCTGCGGGCGGCCAGTGAGGCGGGCGGCCCGGCGCGCCTCGAGTACTATGAGAATGAGAAGAAGTGGAGGCACAAGTCGAGCGCCCCCAAACGCTCGATCCCCCTGGAGAGCTGCTTCAACATCAACAAGCGGGCTGACTCCAAGAACAAGCACCTGGTGGCCCTCTACACCCGGGACGAGCACTTCGCCATCGCGGCCGACAGCGAGGCCGAGCAGGACAGCTGGTACCAGGCCCTCCTGCAGCTGCACAACCGGGCCAAGGGCCACCACCACGATGGCGCCGTGGCCTCCGGGGTGGGAGGCGGTGGCGGCGGGGGCAGCTGCAGCGGTGGCAGCTCGGGCCTGGGCGAGGCCGGGGAGGACCTGAGCTATGGGGACGTCCCCCCGGGGCCCGCGTTCAAGGAAGTCTGGCAGGTGATCCTGAAGCCCAAGGGCCTGGGGCAGACAAAGAACCTGATTGGTATCTACCGCCTCTGCCTGACCAGCAAGACCATCAGCTTCGTGAAGCTCAACTCCGAGGCGGCCGCCGTGGTGCTACAACTCATGAACATCCGCCGCTGTGGCCACTCTGAGAACTTCTTCTTCATCGAGGTGGGCCGCTCAGCCGTGACCGGCCCGGGGGAGTTCTGGATGCAGGTGGATGACTCCGTGGTGGCCCAGAACATGCACGAGACCATCCTGGAGGCCATGCGGGCCATGAGCGACGAGTTCCGCCCACGCAGCAAGAGCCAGTCGTCCTCCAACTGCTCCAACCCCATCAGCGTCCCCCTGCGACGGCACCACCTCAACAATCCCCCACCCAGCCAGGTGGGCCTGACCCGCCGCTCCCGCACTGAGAGCATCACCGCCACGTCCCCCGCTAGCCTGGTGGGCGGCAAGCAGGGCTCCTTCCGGGTGCGCGCGTCCAGCGACGGCGAGGGCACCATGTCCAGGCCAGCCTCGGTGGATGGCAGCCCTGTGAGCCCCAGCACTACTCGAACCCACGCCCACAGGCACCGAGGCAGCTCCCGGCTGCACCCACCTCTCAACCACAGCCGTTCCATCCCCATGCCTTCTTCTCGCTGCTCGCCCTCGGCCACCAGCCCGGTCAGCCTGTCGTCCAGCAGCACCAGTGGCCACGGCTCCACCTCCGACTGCCTCTTCCCGCGACGCTCCAGCGCCTCGGTGTCCGGCTCCCCCAGCGACGGTGGCTTCATCTCCTCCGATGAGTATGGCTCCAGCCCCTGCGACTTCAGGAGCTCCTTCCGCAGCGTCACCCCGGATTCCTTGGGCCATACCCCGCCAGCCCGCGGTGAGGAGGAGCTGAGTAACTACATCTGCATGGGAGGCAAGGGGGCTGCCACTTTGGCGGCCCCCAATGGCCACTACATCCTGCCTCGGGGTGGCAATGGGCATCGCTATGTCCCCGGTGCTGGCTTGGGTCTGAGCCCAGCGCTGGCCGGAGAGGAAGCGGCCACTGCTGCCGACCTGGAGAACCGCTTCCGAAAGCGGACCCATTCGGCTGGCACCTCGCCCACCATCTCCCACCAGAAGACGCCCTCTCAGTCCTCCGTGGCGTCCATCGAGGAATATACAGAGATGATGCCCGCCTACCCACCAGGAGGTGGCAGTGGAGGCCGGCTGCCTGGCTACCGGCATTCCGCCTTCGTGCCCACCCACTCATACCCGGAGGAGGGTCTGGAGATGCACCCCCTAGAGCGACGGGGGGGCCACCACCACCACCGCTCTGATGCCTCTGCCCTCCACACCGACGACGGCTATATGCCCATGTCCCCCGGGGTGGCCCCTGTGCCCAGCGGCAGCCGCAAGGGCAGCGAGGACTACATGCCCATGAGCCCCAAGAGTGTGTCGGCCCCGCAGCAGATCATCAACCCAGTCCGCCGCCACCCGCAGAGAGTGGACCCCAATGGCTACATGATGATGTCCCCCAGTGGAAGCTGCTCCCCTGACATCGGGGGTGGGCCTGGTGGTGGTGGGAACAGCACCACCCAGTCTGGGAGCACTTACGGCAAGATGTGGACAAACGGGGTAGGAGGGCACCACTCTCACGCCCTCCCGCACCCCAAACTGCCCTCTGAAAGCCGTGGCGGTGGGGGCAAGCTCCTGCCTTGCACAGGGGACTACATGAACATGTCGCCGGTGGGGGACTCCAACACCAGCAGCCCCTCCGACTGCTACTATGGCCCAGAAGACCCCCAGCACAAGTCGGTCCTCTCTTACTACTCCTTGCCAAGGTCTTTTAAGCACACCCAGCGGCCGGGGGAGTTGGAGGAGCCCCCCCGACACCAGCACCTCCGCCTGTCCTCCAGCTCGGGGCGCCTCCTCTACGCTGCTGCAGCTGCAGATGACTCTTCATCCTCCACCAGCAGCGACAGCCTGGGCGGGGGCTATTGTGGGGGGCGGCCTGAGCCCGGCCTCCCGCACCTGCACCACCAGGTGCTGCAGCCCCACTTGCCCCGCAAGGTGGACACAGCCGCCCAGACCAATAGCCGCCTCACCCGGCCCACCAGGCTGTCCCTAGGAGACCCCAAGGCCAGCACCTTACCGAGGGCCAGAGAGCAGCCCCCACCCCCACAGCCGCCCTCACTGGGCCACTACCCTCCCGAGCCCAAGAGCCCAGGGGAATACGTGAACATAGATTTTGGGAGCGAGCAGCCTGGCTACTTATCCTGCCCTGTGGCTTCGCACAGCTCGCCTGCGGTCAAGTGTCCAGCTCAGCTCCAGCTGGCTTCCCGCCAGGAAGAGACAGGCACAGAAGAATACATGAACATGGACTTGGGGCCCGGCCGGAGGGTGGCCTGGGAGGATAGCCCCAGAGGCCCACCAGGAAGAGGGGTCCCTGCCCCCCCAGGGGCCCCCAGCCTATGCAGACCGCAGCGGGCGGTGCCCGGCGGCCGGGGCGACTACATGACTATGCAGATGGGCTGTCCCCGCCAGAGCTACGTGGACATCTCACCTGTGGCCCCCGTGAGCTATGCGGACATGCGGACAGGCTTGGCGGAGGAGGCCAGCCTTCCTGGGGCCACAGCGGACACGCCCTGTTCATCTTCAGCTGCCTCGACTTCCCCCACGGCTCCTCCGGGAGGGTCTTCTTCCCTGTTGGGGGCCCCCCAGGGCCCCGGGAGCGTGAGCGCCTTCACCAGGGTGAACCTCAGTCCCAACCACAACCAGAGTGCCAAAGTGATCCGCGCCGACCCGCTAGGGTGCAGGAGGCGCCACAGCTCCGAGACCTTCTCCTCCACACCCAGTGCCACCCGGGCAGGCAACGCGGTGCCCTTCGGTGCGGGGGCTGCCATGGGGGCCAGCGGGGGCGGCAGCAGCAGCAAGGAGGACATGAAGCGCCACAGCTCGGCCTCCTTTGAGAACGTGTGGCTGAGGCCTGGGGAACTGGGGGGAGCCCCCAAGGAGCCAGCCCCTGTGTGCGGGGGCCCCGGGGGCGTGGAGAATGGGCTGAACTACATAGACCTGGATTTGGTTAAGGACTTCAAGCAGCGCCCTCAGGAGCGCCCCCCGCAACTGCGACCCCCGGCACCCCCATCCCCGCGCCAGCCTCTGGGCAGCAGTGAGAGCGGCTCTGCCAGCCGCTCCAGCGAGGATCTAAGCGCCTATGCCAGCATCAGTTTCCAGAAGCAGCCAGACGACCTCCAGTAG

>Con_cr1 [Star-nosed mole (Condylura cristata) Irs1]

ATGGCGAGCCCTCCGGAGACCGACGGCTTCTCGGACGTGCGCAAGGTGGGCTACCTGCGCAAACCCAAGAGCATGCACAAGCGCTTTTTCGTGCTGCGGGCGGCCAGTGAGACTGGGGGCCCCGCGCGCCTTGAGTACTATGAGAACGAGAAGAAGTGGCGGCACAAGTCGAGCGCCCCCAAACGCTCGATCCCCCTCGAGAGCTGCTTCAACATCAACAAGCGGGCTGACTCCAAGAACAAGCACCTGGTGGCCCTCTACACCCGGGACGAGCACTTTGCCATCGCAGCGGACAGCGAGGCCGAGCAGGACAGCTGGTACCAGGCCCTGCTGCAGCTGCACAACCGTGCCAAGGGCCACCACGACGGGGCCTCAGCGCCCGGCGCGGGCGTCGGTGGGGGCAGCTGCAGTGGCAGCTCTGGCCTCGGGGAGGCTGGGGAGGACTTGAGCTATGGGGACATGCCCCCAGGACCCGCGTTCAAGGAGGTCTGGCAGGTGATCCTGAAACCCAAGGGTCTGGGGCAGACAAAGAACCTGATTGGCATCTACCGTCTCTGCCTGACCAGCAAGACCATCAGCTTTGTGAAGTTGAACTCTGAGGCAGCGGCTGTGGTGCTGCAGCTGATGAACATCAGGCGCTGTGGCCACTCAGAGAACTTCTTCTTCATCGAAGTGGGCCGTTCTGCAGTGACTGGACCTGGGGAGTTCTGGATGCAGGTGGATGACTCCGTGGTGGCCCAGAACATGCACGAGACCATCCTGGAGGCCATGCGGGCCATGAGCGACGAGTTCCGCCCTCGAAGCAAGAGCCAGTCCTCCTCCAACTGCTCTAATCCCATCAGTGTCCCCCTGCGCCGGCACCACCTCAACAACCCGCCGCCCAGCCAAGTGGGGCTCACGCGCCGCTCGCGCACGGAGAGCATCACCGCCACCTCCCCCGCCAGCCTGGTGGGCGGGAAGCAGGGCTCCTTCCGTGTTCGGGCCTCCAGTGATGGCGAAGGCACGATGTCCCGCCCAGCCTCGGTGGATGGCAGCCCCGTGAGTCCCAGCACCAACCGGACCCACGCGCACCGGCACCGGGGCAGCTCCCGGCTGCACCCGCCTCTCAACCACAGCCGCTCTATCCCCATGCCCTCCTCTCGCTGCTCCCCTTCGGCCACCAGTCCGGTCAGCCTGTCCTCCAGCAGCACCAGCGGCCACGGCTCCACCTCCGACTGTCTCTTCCCACGGCGGTCCAGCGCTTCTGTGTCTGGGTCCCCCAGCGACGGGGGTTTCATCTCCTCGGATGAGTACGGCTCCAGCCCCTGCGATTTCCGAAGTTCTTTCCGCAGTGTCACCCCAGATTCGCTGGGCCACACCCCACCCGCCCGTGGTGAGGAGGAGCTAAGCAACTATATCTGCATGGGCGGCAAGGGCGTTTCCACTCTCGCCGCCCCCAATGGACACTACATTCTGCCTCGAGGTGGCAATGGTCACCGCTACATCCCAGGAACTAGTCTGGGGCTGAGCCCAGCCCTGGCTGGGGATGAAACGGCCGGTGCTGCAGATCTGGATAATCGGTTCCGAAAGCGGACTCACTCTGCTGGCACCTCCCCTACCATTTCCCACCAGAAGACCCCATCCCAGTCCTCCGTGGCTTCCATTGAGGAATATACAGAGATGATGCCTGCTTACCCGCCGGGAGGTGGCAGTGGCGGCCGGCTGCCGGGCTACCGGCAGTCGGCCTTCGTGCCCACCCACTCCTACCCTGAGGAGGGTCTGGAAATGCACCCCATGGAGCGCCGTGGGTGCCCTCACCGCCCAGATGCCTCCACCCTCCACACTGATGATGGCTACATGCCCATGTCCCCAGGGGTGGCCCCAGTGCCCAGCGGCCGGAAGGGCAGTGGCGAGTACATGCCCATGAGCCCTAAGAGCGTGTCTGCCCCGCAGCAGATCATCAACCCCATCAGACGCCATCCCCAGAGGGTGGACCCCAATGGCTACATGATGATGTCCCCAAGCGGCAGCTGCTCTCCCGACACTGGAGGTGGGCCCGGCAGCAGCAACGCCGCCCCTCCTGGGAGCAGCTATGGCAAGCTATGGACAAATGGGGTAGGGGCACATCACGCCCATGCCCTGCCGCACCCCAAACTTCCTGTGGAGAGCGGTGGTAGCAAGCTCTTGTCTTGTACAGGTGACTACATGAACATGTCACCAGTGGGGGACTCCAACACCAGCAGCCCCTCCGATTGCTACTACGGCCCAGAGGACCCCCAGCACAAGCCGGTCCTCTCCTACTACTCATTGCCAAGGTCCTTTAAGCACACCCAGCGCCCCGGGGAACTGGAGGAGAGTGCCCGGCACCAGCACCTCCGCCTCTCCTCCAGCTCTGGCCGGCTCCTCTGTGCTGCAGCGGCAGAAGATTCCTCCTCCTCCACCAGCAGTGACAGTCTGGGCGGGGGGTACTGTGGGGTGAGACCGGAGTCCGGCCTCCTGCATCTCCACCATCAGGTCTTGCAGCCCCATCTGCCCCAAAAGGTGGACACAGCTGCCCAGACCAACAGCCGCCTGACTCGGCCCACAAGGCTGTCCCTGGGGGATCCTAAGGCCAGCACCTTACCCCGGGCACGAGAGCAGCCGCCGCAGCCCCTGCTGCACCCTCCAGAGCCCAAGAGCCCAGGGGAGTACGTGAATATCGAATTTGGGAGTGATCAGCCAGGTTACCTGTCTGGCCCCACAGCTCCCCACAGCTCGCCTTCTGTCAGGTGTGCCTCCCAGCTGCAGCCAGCACCCAGGGAGGAAGAGTCCGGTACCGAAGAGTACATGAACATGGACCTGGGGCCGGGCCGGAGGGCTGCCTGGCAGGAGAGCCCGGGGGTGCAGGCGGGCAAAGTGGGTCCTGCACCCCCTGGGGCAGCTAGCATGTGCAGGCCCACCCGGGCGGTGCCCAGCAGCCGGGGGGACTACATGACCATGCAGATGGGCTGCTCCCGGCAGAGCTACGTGGACACCTCGCCAGTGGCCCCGGTCAGCTACGCTGACATGCGGACAGGCATTGTCGCGGAGGAGGCCAGCCTCCCAGGGGCAACAGCAGCTGCGCCCTCCTCATCCTCAGCAGCCTCTACTTCTCCCACTGCACCTCCAGGAGCTGGGGAGCTGGCAGCCCACTCTTCCCTGCTGGGGGGCCCTCAGGGACCTGCGGGCTTGAGCGCCTTCAGCCGGGTGAACCTCAGTCCCAACCGCAACCAGAGTGCCAAAGTGATCCGTGCAGACCCTCAAGGGTGCAGGAGGCGGCACAGCTCCGAGACCTTCTCTTCCACACCCAGTGCCACCCGGCCCTTCGGAGGGGGGGCCGCTGTAGGGGGCAGCGGTGGGGGCAGCAGCAGCACAGAGGATGTGAAACGCCACAGCTCTGCTTCCTTTGAGAACGTGTGGCTGAGGCCCGGGGAGCTTGGGGGAGCCCCCAAGGAGCCGGCCCAGGTGTGTGGGGCTGCTGGGGGTTTGGAGAATGGTCTTAACTACATAGACCTGGATTTGGTCAAGGACTTCAAACAGCACCCAAAGGAGCACTCTCCACAAGCGCAGACTCCCCCAACCTTGCCCCCTCATCAGCCTCTGGGCAGCAGCGAGAGCAGTTCCACCAGCCACTCCAGTGAGGATTTAAGCGCCTATGCCAGCATCAGTTTCCAGAAGCAGCCAGAGGACCTCCAGTAG

>Tri_ma1 [Florida manatee (Trichechus manatus latirostris) Irs1]

ATGGCGAGCCCTCCAGAGACCGACGGCTTCTCGGACGTGCGCAAGGTGGGCTACCTGCGCAAACCCAAAAGCATGCACAAGCGCTTCTTCGTGCTGCGGGCGGCCAGCGAGGCTGGGGGCCCGGCGCGCCTGGAGTACTACGAGAACGAGAAGAAGTGGCGGCACAAGTCGAGCGCCCCCAAACGCTCGATCCCCCTCGAGAGCTGCTTCAACATCAACAAGCGGGCGGACTCCAAGAACAAGCACCTGGTGGCTCTCTACACCCGGGACGAGCACTTTGCCATCGCGGCGGACAGCGAGGCCGAGCAGGACAGCTGGTACCAGGCCCTGCTGCAGCTGCACAACCGTGCCAAAGGGCACCACGATGTGGCTGCGGCCCCCGGGCTAGGAGGTGGGGGGAGCAGCTGCAGCGGCAGCGCCTGCCTTGGTGAGGCTGGGGAGGACTTGAGTTACGGAGATGTGCCCCCAGGCCCTGCCTTCAAGGAGGTCTGGCAGGTGATCCTGAAGCCCAAGGGCCTGGGGCAGACAAAGAACCTGATTGGCATCTACCGCCTCTGTCTGACCAGCAAGACCATCAGCTTCGTGAAGCTCAACTCAGAGGCGGCCGCAGTGGTGCTGCAGCTGATGAACATAAGGCGCTGCGGCCACTCAGAGAACTTCTTCTTCATCGAGGTGGGCCGCTCTGCCGTGACAGGGCCTGGGGAGTTCTGGATGCAGGTGGATGACTCGGTGGTGGCACAGAACATGCATGAGACTATCCTGGAGGCCATGCGGGCCATGAGCGATGAGTTCCGCCCTCGAAGCAAAAGCCAGTCCTCCTCCAACTGCTCCAATCCCATCAGCGTCCCCCTGCGCAGGCACCACCTCAACAACCCCCCTCCCAGCCAGGTGGGGCTGACCCGCCGCTCGCGCACGGAGAGCATCACCGCCACCTCCCCAGCCAGCATGGTGGGCGGGAAGCCTGGCTCCTTCCGGGTCCGCGCCTCCAGTGATGGCGAAGGCACCATGTCCCGCCCAGCCTCAGTGGACGGCAGCCCGGTGAGTCCGAGCACCAACAGGACCCACGCCCACCGGCATCGCGGCAGCTCCCGGCTGCATCCTCCGCTCAACCACAGCCGCTCCATCCCCATGCCTACTTCTCGCTGTTCACCTTCCGCCACCAGCCCGGTCAGTCTATCGTCCAGCAGCACGAGTGGCCACGGCTCCACCTCTGACTGCCTCTTCCCACGAAGGTCTAGTGCTTCTGTGTCCGGTTCCCCGAGCGATGGTGGCTTCATCTCTTCTGATGAGTATGGCTCTAGTCCTTGCGATTTCCGAAGTTCCTTCCGTAGTGTCACCCCGGATTCCCTGGGCCACACCCCCGGTGAGGAGGAGCTGAGCAACTATATCTGCATGGGCGGCAAGGGGGCCTCCACCCTGACTGCCCCCAATGGGCACTACATCTTGGCTCGGGGCGGCAATGGCCACCGCTACATCTCAGGAGCTAGTTTGGGCACGAGCCCAGCGCTGACTGCGGATGAAGCAGCCAATGCTGCAGATGTGGAAAATCGCTTCCGAAAAAGGACTCACTCCGCGGGCACGTCCCCCACCATTTCCCACCAGAAGACCCCGTCCCAGTCCTCCGTGGCGTCCATTGAGGAGTATACAGAGATGATGCCCGCCTACCCACCAGGAGGGGGCGGTGGAGGCCGACTGCCTGGCTACCGGCACTCTGCCTTCGTGCCCACGCACTCCTACCCGGAGGAGGGTCTGGAAATGCATCCCTTGGAGCGGCGTGGGGGCCACCACCGCCCAGACACCTCCACCCTCCATACCGATGATGGCTACATGCCCATGTCCCCAGGGGTGGCCCCAGTGCCCAGCAACCGGAAGGGCAGTGGGGACTACATGCCCATGAGTCCCAAAAGCGTGTCTGCCCCACAGCAGATCATCAACCCCATCAGACGCCATCCCCAGAGAGTAGACCCCAATGGCTACATGATGATGTCCCCAAGCGGCAGCTGCTCCCCTGACATTGGAGGTGGGCCCAGCAGCAGCAGCACTGCCCCTTCCGGGAGCAGCTATGGGAAGCTGTGGACAAATGGGGTAGGGGGCCACCATTCTCACCCCTTGCCTCACCCCAAACAACCCGTGGAAAGCAGTGGTGGCAAACTCTTGCCTTGCACAGGTGACTATATGAACATGTCACCGGTGGGGGACTCCAACACCAGCAGCCCCTCTGACTGTTACTATGGCCCCGAGGACCCCCAGCACAAGCCGGTCCTCTCCTATTACTCATTGCCAAGGTCCTTTAAGCACACCCAGCGCCCAGGGGAGCCAGAGGAGAGTGCCCGCCACCAGCACGCCCGACTTTCCTCCAGCTCTGGTCGTCTTCTCTATGCTGCAGCCGCGGAAGATTCTTCCTCCTCCACCAGCAGTGATAGCCTGGGAGGGGGATACTGTGGGCCTCGGCAGGAGCCTGGCCACCCGCATCTCCACCATCAGGTTCTGCAACCCCATCTGCCTCGAAAGGTGGACACGGCCGCCCAGACCAACAGCCGCCTGGCTCGGCCCACAAGGCTGTCTCTGGGGGATCCCAAGGCCAGCACCTTACCGCGGGCCCGCGAGCAGCCACAGCAGCCCCTGCTGCACCCTCCGGAGCCCAAAAGCCCAGGGGAATATGTGAATATTGAATTTGGGAGTGACCAGCCTGGCTACTTATCTGGCCCCGTGGCTTCGCACAGCTCGCCTTCTGTCAGGTGTCCAGCCCAGCTCCAGCCAGCCCCCAGAGAGGAAGAGACTGGTGCTGAGGAGTACATGAACATGGATCTGGGGCCAGGCCGGAGGGTGGCCTGGCAGGAGAGCACTGGGGTGGAGATGGGCAGAGTGGGCCCTGCGCCTCCTGGGACTGTGAGCATGTGCAGACCCACGAGGGCCGTGCCCAGCAGCCGGACTGATTACATGACCATGCAGATGGGCTGTTCCCGCCAGAGCTACGTGGACACCTCACCTGTCGCCCCTGTCAGCTATGCCGACATGCGGACGGGCATTGCTGCAGAGGACGTGAGCCTTCCGGGGGCCACAGCGGCTGCTCCCTCCTCATCCCCGGCAGCCTCTGCCTCCCCTTCTGCACCTCAAGGAGCAGCTGAGCTGGCTGCCTGCCCTTCCCTGCTGGGGGCCCCGCAGGGACCTGGAGGCGTGAGCGCCTTCACCCGGGTGAGCCTCAGCCCCAACCGCAACCAGAGTGCCAAAGTTATCCGTGCGGACCCGCAAGGGTGCCGGAGGAGGCATAGCTCCGAGACCTTCTCCTCAACACCCAGTGCCACCCGGGCAGGCAACGCCGTGCCCTTCGGAGGAGGAGCTGGGGCTGGGGCTGGGGGCAGCAGCGGTGCTAGCAGCAGCAGCAGTGAGGATGTGAAACGCCACAGTTCTGCTTCCTTTGAGAACGTGTGGCTGAGGCCTGGGGAGCTCGGGGGAACCCCCAAGGAGCCAGCCCAGGTGTGTGGGGCTGCTGGGGGCTTCGAGAATGGTCTTAACTACATAGACCTGGATTTGGTCAAGGACTTTAAACAGCGCCCTCAGGAGCGCCCCCCTCCGCCGCAGCCCCCTCCACCGCCGCCCCCTCATCAGCCCCTTGGCAGCAGCGAGAGCAGCTCGCCCAGCCGCTCCAGCGAGGATTTAAGCGCCTATGCCAGCATCAGTTTCCAGAAGCATCCAGAGGACCTCCAGTAG

>Chr_as1 [Cape golden mole (Chrysochloris asiatica) Irs1]

ATGGCGAGTCCTCCGGAGACCGACGGCTTCTCGGACGTGCGCAAGGTGGGCTACTTGCGCAAACCCAAGAGCATGCACAAGCGCTTCTTCGTGTTGCGGGCAGCCAGCGAGGCAGGGGGCCCGGCGCGTCTCGAGTACTACGAGAACGAGAAGAAGTGGAGGCACAAGTCGAGCGCCCCCAAACGCTCGATCCCCCTCGAGAGCTGCTTCAACATCAACAAGCGGGCGGATTCCAAGAACAAGCACCTGGTGGCTCTCTATACCCGGGACGAGCATTTCGCCATTGCGGCCGACAGCGAGGCCGAACAGGACAGCTGGTACCAGGCCCTGCTGCAGCTGCACAACCGTGCCAAGGGCCACCACGAAGGGGCTGTGGCACCCGGGCTGGGAGGTGGCGGGGGCAGCTGCAGCGGCAGTGCCTGCCTGGGCGAGGCTGGGGAGGACTTGAGTTACGGGGACGTGCCCCCAGGCCCTGCCTTCAAGGAGGTCTGGCAGGTGATCCTAAAGCCTAAAGGTCTGGGTCAGACAAAGAACCTGATTGGCATCTACCGCCTCTGTCTGACCAGCAAGACCCTCAGCTTCGTGAAGCTCAACTCGGAGGCGGCCGCCGTGGTGCTACAGCTGATGAACATCAGGCGCTGCGGCCACTCAGAGAACTTCTTCTTCATCGAGGTGGGCCGCTCCGCTGTGACGGGACCCGGGGAGTTCTGGATGCAGGTGGATGACTCTGTGGTGGCCCAGAACATGCATGAGACCATCCTGGAGGCCATGCGGGCCATGAGCGACGAGTTCCGCCCTCGCAGCAAGAGCCAGTCCTCCTCTAACTGTTCCAACCCCATCAGCGTCCCCCTGCGAAGGCATCACATCAATAACCCCCCACCCAGCCAGGTGGGACTGACCCGCCGCTCACGCACCGAGAGCATCACAGCCACCTCCCCGGCCAGCATGGTAGGTGGGAAGCCCGGCTCCTTCCGAGTTCGCGCCTCCAGTGACGGCGAAGGCACCATGTCCCGGCCAGCTTCGGTGGATGGCAGCCCCGTTAGTCCTAGCACCAACAGGACCCACGCCCACCGGCATCGGGGAAGCTCTCGGCTGCACCCTCCGCTCAACCACAGTCGCTCCATCCCCATGCCTACTTCTCGCTGCTCACCTTCCGCCACCAGCCCGGTCAGCTTATCTTCCAGCAGCACCAGTGGCCACGGCTCCACCTCGGACTGCCTCTTCCCGCGGCGGTCTAGTGCCTCTGTGTCCGGATCCCCCAGTGATGGTGGATTCATCTCTTCCGATGAGTATGGCTCCAGTCCTTGCGATTTCCGTAGTTCCTTCCGCAGTGTCACCCCGGATTCCCTGGGCCACACCCCACCTGCTCGCGGTGAGGAGGAGCTGAGCAACTACATTTGCATGGGTGGCAAGGGGGCCTCCACCCTGACTGCCCCCAACGGTCACTACATCTTGACCCGGGGCGGCAATGGCCACCGCTGCATCCCAGCAGCAAGTTTGGGAACGAGCCCCGCCCTGCCGTCAGAGGAAACAGCTGGTGCTGCAGATTTGGAGAATCGCTTCCGAAAGAGGACTCACTCTGCGGGCACATCCCCCACCATTTCCCACCAGAAGACCCCGTCCCAGTCCTCAGTGGTGTCCATAGAGGAGTATACCGAGATGATGCCCGTCTACCCAACAGGAGGGGGCAGCGGAGGCCGACTGCCCAGCTACCGGCACTCCGCCTTCGTGCCCACGCACTCCTACCCCGAGGAGGGGCTGGAGATGCATCCCTTGGAGCAGCGTGGGGGTCACCACCGCCCAGACGCGTCCACCCTCCACACCGATGATGGCTACATGCCCATGTCCCCAGGGGTGGCCCCAGTGCCCAGCAACCAAAAGGTCAGTGGTGACTACATGCCCATGAGCCCCAAGAGTGTGTCTGCCCCACAGCAGATCATCAACCCCACCAGACGCCATTCCCAGCGAGTGGACCCCAACGGCTACATGATGATGTCCCCAAGTGGCAGCTGCTCGCCTGACAATGGTGGTGGGCCCAGCGGAGGCGGCAACAGCCACAGTACTGCCCCTTCTGGAAACAGCTATGGGAAGCTGTGGACAAATGGGGTAGGGGGCCACCATTCTCCTCACCCCAAACAGGCTGTAGAAAGCAGTGGTGGCAAGCTCCTGCCTTGCACAGGTGACTACATGAACATGTCACCTGCGGGAGACTCTAACACCAGTAGCCCTTCGGACTGCTCCTATGGTCCCGAGGACCCCCAGCACAAGCCGCTCCTCTCCTATTACTCATTGCCAAGGTCCTTTAAGCACACCCAGCGCCCAGGGGAGCCAGAGGAAAGTGCCCGCCATCAGCACCTCCGGCTCTCCTCCAGCTCTGGACGCCTTCTCTATGCTGCAGCCCTGGATGATTCTTCGTCCTCCACCAGCAGTGATAGCCTGGGAGGGGGGTACTGTGGGCCTCGGCCAGAGCCTGGACACCCACATCTCCACCATCAGGTCCTGCAACCCCATCTGCCTCGAAAGGTGGACATGGCCGCCCAGACCAACAGCCGCCTGGCTCGGCCCACGAGGCTGTCTCTAGGGGATCCCAAAGCCAGCACCTTACCTCGGGCCCGTGAACAGCCTCAGCAGCCATTGCTGCTTCAGCCTCAGGAGCCCAAGAGCCCAGGGGAATATGTGAATATTGAGTTTGGGATTGAGCAGCCAGGCTACTTCTCTGGCCCTATGGCTTCACACCACACCTCACCGTCCATCAGGTGTCCATCACAGCTCCAACCAGCACCCAGAGAGGAAGAGACTGGTACTGAAGAGTACATGAACATGGACCTGGGCCCTGGACGGAGGGCAGCCTGGCAGGAGAGTGTGGGGACAGAGATGGACAGAGCAGGCCCGGCTCCACCTGGGACTGGGAATGTGTGCAGGCCCACGCGGGCTGTACCTAGCAGCCGAGGAGACTACATGACCATGCAGGTGGGCTATTCCCGGCAGAGTTATGTGGACACCTCGCCAGTGGCCCCGGTCAGCTATGCTGATATGCGGACAGGCGTTGTTGCAGAAGATGTGAGTCTTCCTGTGGCCACAGCGGCTGCTCCCTCATCCTCAGCAGCCTCGGCCTCCCCCACTGCACCTCAAGGAGCAGCTGAGCTGACTACTTGCCCTTCCCTGCTGGGGGGTCCACAGGGACCTGGGGGCATGAGCGCCTTCACCCGGGTTAACCTCAGTCCCAACTGCAACCAGAGTGCCAAAGTGATCCGTGCTGACCCGCAAGGCTGCCGGAGAAGGCACAGCTCTGAGACCTTCTCCTCAACACCCAGCGCCACCCGGGCAGGCAACATGATGCCCTTCGGAGGGGGCGCTACAGCAGGGGGGGCCGGCAGCAGTGCCAGCAGCAGTGAGGATGTGAAACGCCACAGTTCTGCTTCCTTTGAGAATGTGTGGCTGCGGCCGGGGGAGCTCGGGGGGACCCCTAAGGAGCTGGCCCAGGTGTGTGGGGCTGCTGGGGGCTTTGAGAATGGTCTTAACTACATAGACCTGGATTTGGTCAAGGACTTTAAACAGCGCCCTCAGGAGCGTCCCCCTCCCCCGGAGCCCCCTGCCCCGCCTCCCCCTCACCAGCCCCTTGGCAGCAACCAGAGCAGCTTGACCCGCCGTTCCAGCGAGGATTTAAGTGCTTATGCCAGCATCAGTTTCCAGAAGCAGCCAGAGGACCTCCAGTAG

>Ech_te1 [Small Madagascar hedgehog (Echinops telfairi) Irs1]

ATGGCGAGCCCTTCAGAGACCGACGGCTTCTCGGACGTGCGCAAGGTGGGCTACCTGCGCAAACCCAAGAGCATGCACAAGCGCTTCTTCGTGCTGCGAGCGGCCAGCGAGGCGGGGGGCCCTGCGCGCCTCGAGTACTACGAGAACGAGAAGAAGTGGCGGCACAAGTCGAGCGCCCCCAAACGCTCCATCCCCCTCGAAAGCTGTTTCAACATCAACAAGAGGGCGGACTCCAAGAACAAGCACCTCGTGGCTCTCTACACCCGCGACGAGCACTTTGCCATCGCGGCCGACAGCGAGGCGGAGCAGGAGAGCTGGTACCAGGCCCTGCTGCAGCTGCACAACCGGGCCAAGAGCCACCACGACGGGGCTCAGGCGCCGGGGCTGGGAGGCGGGGGAGGCAGCTGCAGCGGCAGCGCCTGCCTGGGGGAGGCTGGGGAGGACTTGAGCTATGGGGACGTGCCCCCGGGCCCTGCCTTCAAGGAGGTCTGGCAGGTGGTGCTGAAGCCCAAGGGCCTGGGACAGACAAAGAACCTGGTTGGCATCTATCGCCTCTGCCTGACCAGCAAGACCATCAGCTTCGTGAAGCTCAACTCCGAGGCGGCCGCCGTGGTGCTCCAACTGATGAACATCCGCCGCTGCGGCCACTCGGAGAACTTCTTCTTCATCGAGGTGGGCCGCTCTGCTGTGACCGGCCCGGGGGAGTTTTGGATGCAGGTGGATGACTCGGTGGTGGCGCAGAACATGCACGAGACCATCCTGGAGGCCATGCGGGCTATGAGCGATGAGTTCCGCCCTCGCAGCAAGAGCCAGTCCTCCTCCAACTGTTCCAATCCCATCAGCGTCCCCTTGCGCCGCCACCACCTCAACAACCCCCCGCCCAGCCAGGTGGGCCTGACGCGTCGCTCCCGTACCGAGAGCATCACCGCCACCTCCCCAGCCAGCATGGTGGGCGGGAAACCCGGCTCCTTCCGCGTGCGCGCCTCCAGCGATGGCGAAGGCACCATGTCGCGGCCGGCCTCCGTGGACGGCAGCCCTGTGAGTCCCAGCACCAACAGGGCCCACGCGCACCGCCATCGGGCCAGCTCCCGGCTGCACCCTCCGCTCAACCACAGCCGCTCCATCCCCATGCCGACTTCTCGCTGCTCGCCCTCGGCCACCAGCCCGGTCAGCCTGTCGTCCAGCAGCACCAGTGGCCACGGCTCCACTTCAGACTGCCTCTTCCCCCGGCGGTCCAGTGCCTCGGTGTCCGGCTCCCCCAGCGATGGCGGGTTCATTTCCTCGGATGAGTATGGCTCTAGCCCGTGCGATTTCCGCAGTTCCTTCCGCAGCGTCACACCGGATTCCCTGGGCCACACCCCGCCGGCTCGCTGCGAGGAGGAGCTGAGCAACTATATCTGCATGGGTGGCAAGGGGGCCTCCACGCTGACCGCCCCCAACGGTCACTACATCCTGGCTCGGGGCGGCTGTGGCCATCGCGGCGGCCCAGGAGCCAGCCTGGGCACGAGCCCTGCGCTGACTGCGGACGAGGCGGCCAGTGTGGCGGACTTGGAGAACCGCTTCCGAAAGAGGACTCACTCTGCGGGCACGTCCCCCACCATCTCTCACCAGAAGACCCCGTCCCAGTCCTCAGTGGCGTCCATCGAGGAATATACCGAGATGATGCCTGCCTACCCACCAGGAGGGGGCAGCGGAGGCCGACTGCCCAGCTACCGGCACTCCGCCTTTGTGCCCACGCACTCCTACCCAGAGGAAGGGTTGGAAATGCAGCGTGGGGGCCAGCAGCGCCCGGACACCACCACAGCCCTCCACCCGGACGATGGCTACATGCCCATGTCCCCGGGGGTGGCCCCAGTGCCCAGCAGCCGAAAGGGCAGCGACTATATGCCCATGAGCCCCAAGAGCGTGTCTGCCCCACAGCAGATCATCAACCCCATCAGGCGCCACCCGCAGAGAATCGACCCCAATGGCTACATGATGATGTCCCCCAGTGGCAGCTGCTCCCCCGACACCGGAGGGGGTCCCGGTGGCAGCAGCGGCGTTGTCCCTTCCTCGAGCAGCTTTGGGAAGCTGTGGACAAATGTGGTGGGGGGGCCCCATTCGCACGCCCTCCCTCACCCACAACCAGCCGCGGAGAGCAGCGGATGCAAGATCCTGCCTTGTGCAGGTGACTACATGAACATGGGGGACTCCAACACCAGCAGCCCCTCCGATTGCTACTACGGCCCCGAGGACCCCCAGCACAAGCTGGTCCTCTCCTACTACTCTTTGCCAAGGTCCTTTAAGCACACCCAGCGCCCAGGGGAGCCAGAGGAAGGTGCGAGGCACCAGCACCTCCGCCTCTCCGCCAGCTCCGGCCGCCTTCTCTATGCTGCAGCCGCCGAAGACTCTTCCTCCTCCACCAGCAGTGACAGCCTGGGAGGGGGCTACTGTGGCCCGCGGCCCGAGCCTGGCCACCCGCCTCTCCACCATCAGGTCCTGCAACCCCATCTGCCTCGAAAGGTGGACATGGCCGCTCAGACCAACAGCCGCCTGGCCCGGCCCACTAGGCTATCTCTGGGGGATCCTAAAGCCAGCACCTTACCCCGGGCCCGTGAGCAACCGCAAGAGCCCAAGAGCCCAGGGGAGTATGTGAATATTGAATTTGGCAGTGGCCAGCCTGGCTACCTGTCCGGCCCTGTGGCTTCACACAGCTTGCCTTCCGTAAATTGCCCAGCCCAGCTCCAGCCAGTGCCCCCGGAGGAAGAGACTGGTGCTGAGGAGTACATGAACATGGATTTGGGGCCTGGTCGGAGGGCAGCCTGGCAGGAAAGCGGCGGCATGGACGTGGGCCGAGTGGGCCCTGCGCCTCCCGGGTCTGTGAGCGTGTGCAGGCCCACGCGGGCCGTGCCCAGCAGCCGCGGGGACTACATGACCATGCAGGTGGGCTGTTCGCGGCAGAGCTACGTGGATACGTCGCCGGTGGCCCCTGTCAGCTATGCTGACATGCGGACGGGCCTGGCTGTAGAGGACATGAGCCTCCCCGGAGCCACAGCGGCGGCCCCCTCCTCAACCTCCGCAGCCTCGACCTCCCCCACTGCACCTCAAGGAGCTGCTGGACTGGCTGCCCGCCCTTCGCTGCTCGGGGGCCCTCCGGGCTCTGGGGGCCTGAGCGCTTTCACCGGGCTACACCTCAGCCCCAATCACAACCAGAGTGCCAAAGTTATCCGTGCGGACCCGCAGGGGTGCCGGAGGAGGCACAGCTCCGAGACCTTCTCCTCGGCACCCACTGCCACCAGGGCAGGCAACGTGGTGCCCTTCGGAGGGGGTGCTGCGGGAGGCGGTGTCAATGGCAGCAGCTATGGTGGCTGCAGTGGTGAGGATCCCAAGCGCCACAGCTCTGCCTCCTTTGAGAACATGTGGCTGAGGCCTGGGGAGTTTGGAGGAGTCCCCAAGGAGCCAGCCCAGGTGTGTGGAGGGACTGCGAGGGGCTTCGAGAATGGTCTTAACTACATAGACCTGGATTTGGTCAAGGACTTTCAACAGCGCGCTCCGGAGCGACCCCCAGCCCCGCGGCCCGCGCCAGAGCGACCCTCTCATCGGCAGCCCCTTGGCAGCAGTAGTGAGAGCAGCTTGACTCGCCGTGCCAGCGAAGATCTAAGCGCCTATGCCAGCATCAGTTTCCAGAAGCAGCCAGAAGACCTCCAGTAG

>Ele_ed1 [Cape elephant shrew (Elephantulus edwardii) Irs1]

ATGGCGAGCCCTCCGGAGACCGACGGCTTCTCGGACGTGCGCAAGGTGGGCTACCTGCGCAAACCCAAGAGCATGCACAAGCGCTTCTTCGTGCTGCGGGCGGCCAGCGAGGCGGGGGGACCGGCGCGCCTCGAGTACTACGAGAACGAGAAGAAGTGGCGGCACAAGTCGAGCGCCCCCAAACGCTCGATCCCCCTGGAGAGCTGCTTCAACATCAACAAGCGGGCGGACTCCAAGAACAAGCACCTGGTGGCTCTCTACACCCGCGACGAGCACTTTGCCATCGCGGCCGATAGCGAGGCCGAGCAGGACAGCTGGTACCAGGCTCTTCTGCAGCTGCACAACCGTGCCAAGGGGCACCACGATGGGGCCGTGGCGCCCGGGCTGGGCGGTGGTGGCGGCAGCTGCAGCGGCAGCGCCTGCCTGGGCGAGGCGGGCGAGGACTTGAGCTACGGAGACGTGCCCCCCGGCCCAGCCTTCAAGGAGGTCTGGCAGGTGATCCTGAAGCCCAAGGGCCTGGGCCAGACAAAGAACTTGATTGGCATCTACCGCCTCTGCCTGACCAGCAAGACCATCAGCTTCGTGAAACTCAACTCAGAGGCGGCGGCGGTGGTGCTGCAGCTGATGAACATCCGGCGTTGCGGTCACTCGGAGAACTTCTTCTTCATCGAGGTGGGCCGCTCGGCCGTCACGGGACCCGGGGAGTTCTGGATGCAGGTGGATGACTCCGTGGTGGCGCAGAACATGCACGAAACCATCCTGGAGGCCATGCGCGCCATGAGTGACGAGTTCCGCCCGCGAAGCAAGAGCCAGTCCTCCTCCAACTGCTCCAACCCCATCAGTGTCCCCATGCGCCGCCACCACCTCAACAACCCCCCGCCCAGCCAGGTGGGGCTGACACGCCGTTCACGCACGGAGAGCATCACCGCCACGTCCCCAGCCAGCATGGTGGGTGGCAAGCCTGGCTCCTTCCGGGTCCGCGCCTCCAGCGACGGGGAGGGCACCATGTCGCGCCCGGCCTCGGTGGACGGCAGCCCCGTGAGCCCCAGCACCAACCGGACGCACGCACACCGGCACCGGGGCAGCTCACGGCTGCACCCCCCGCTCAACCACAGTCGCTCCATCCCCATGCCCACGTCCCGCTGCTCGCCCTCGGCCACCAGCCCCGTCAGCCTGTCATCCAGCAGCACCAGTGGCCACGGTTCCACCTCGGATTGCCTGTTCCCACGCCGGTCCAGTGCGTCTGTGTCCGGCTCCCCCAGTGACGGAGGCTTCATCTCCTCGGATGAGTATGGCTCCAGTCCCTGTGATTTCCGGAGCTCCTACCGCAGCGTCACCCCAGATTCCCTGGGCCACACCCCACCGGCCCGGGGCGAGGAGGACTTGAGCAACTACATCTGCATGGGTGGCAAGGGGGCCTCCACGCTGACCGCCCCCAATGGGCACTACGTCTTGGCTCGGGGTGGCAATGGCCACCGGTGCCTGCCTGGTGCCAGCCTGGGCACCAGCCCGGCGCTGAGTGCTGAGGAGGCCGCCGGTGCTGCAGACCTGGAGAATCGCTTCCGAAAGAGGACGCACTCCGCGGGCACGTCTCCCACCATCTCCCACCAGAAGACCCCGTCGCAGTCCTCCGTGGCGTCCATTGAGGAGTACACGGAGATGATGCCCGCCTACCCTCCAGGAGGGGGCGGTGGAGGCCGCCTGCCGGGCTACCGGCACTCGGCCTTCGTGCCCACGCACTCCTACCCAGAGGGAGCGCTGGAGATGCACCCGCTGGAGCGCCGTGGGGGCCACTCCCGCCCAGACAGCGCCACGCTACAGGCCGAAGAGGGCTACATGCCCATGTCCCCAGGAGTGGCCCCCACGCCCAGCACCCGAAAGGGCAGCGGGGACTATATGCCCATGAGTCCCAAGAGTGTGTCCGCGCCCCAGCAGATCATCAACCCCATCAGGCGCCATCATCCCCAGCGGATGGACCCCAATGGGTACATGATGATGTCCCCGAGCGGGAGCTGCTCCCCGGACACCGGAGGCGGGCCCAGCGGCAGCGCAGCCCCTTCTGGGAGCGGCTATGGGAAGCTGTGGACGAATGGAGTCGGCCACCATTCGCACCCCAAAGCGCCCCTGGAGAGCAGCGGTGGCAAGCTCATGCCTTGCACAGGTGACTATATGAATATGTCGCCTGTGGGGGACTCCACCACCAGCAGCCCCTCCGACTGCTACTACGGCTCAGAGGACCCACAGCCCAAGCCGGTCCTCTCCTACTATTCATTGCCGAGGTCCTTTAAGCACAGCCAGCGCCCTGGGGAGCCCGAGGAAAGCACCCGGCACCAGCACCTCCGCCTCTCTTCCAGCTCCGGCCGACTCCTCTATGCGGCCACAGCGGAAGACTCTTCCTCCTCCACCAGCAGCGACAGCCTGGGCGGAGGGTACTGCGGGCCCAGACCCGAGCCTGGCCACCCGCACCACCACCCACAGGCCCTGCAACCCCACCTGCCTCGCAAGGTGGATATGGCCGCCCAGACCCACAGCCGCCTGGCCCGGCCCACGAGGCTGTCCCTGGGAGACCCCAAGGCCAGCACCTTACCTCGGGCACGTGAGCAGCCGCAGCCGCTGCACGCAACGGAGCCCAAGAGCCCCGGGGAGTATGTGAACATTGAGTTCGGGAGGGACCAGCCTGGCTACTTGACTGGCCCGGTGGCTGCACGCAACTCACCTGCGGTCAGTTGCCCGGCCCCGCTGCAGCCGGTGCCCCGAGAGGACGGGGCTGGCACGGAGGAGTACATGAACATGGACCTGGGACCTGGCCGGAGGGGGCCCTGGCAGGAGAGCCCAGGGCTAGAGGTGGGCAGAGCGGGCCCTGCGCCCCCCGGGGCGCTGAGCGTGTGCCGGCCCACGCGGGCTGTGCCCAGCAGCCGGGGTGACTATATGACCATGCAGATGGGCTGCGCCCGGCCGAGCTACGTGGACACCTCGCCAGTGGCCTCGGTCAGCTACGCTGACATGCGGACAGGCATCACTGCAGAGGACGCGAGCCTGCCCACGGCCCCCGCGGCCGCACCCTCCTCATCCTCAGCCACCTCGGCCTCGCCCGCTGCACTTCAGGGAGGAGCCGAGCTGCCTGTCCGCCCTTCCCTGCTGGGGGGCCCGCAGGGCGCAGGGGCCCTGAGCGCCTTCACCCGGGTCAACCTCAGCCCCAACCGCAACCAGAATGCCAAAGTGATCCGGGCGGACCCGCAGGGGTGCAGGCGGAGGCACAGCTCCGAGACCTTCTCGACGCCCAGTGCCACCCGGGTGGGCAACCCGGCGCCCTTGGCAGGGGTTGCTGCGGCCGCGGGGGACGGCGGAGCCAACAGCAATGAGGATGTGAAGCGCCACAGTTCTGCCTCCTTTGAGAACGTGTGGTTGAGGCCTGGAGAGCTCGGAGGAGCCCCCAAGGAGCCGGCCCAGGGATGCGGGGCTGTAGGGGGATTCGAGAATGGGCTTAACTACATAGACCTGGATTTGGTCAAGGACTTTAAACGGCGCCCCCAGGAGCGACCCCCTCCGCCACCCCCGCAGCCGCCACCCCCTCACCAGCCCCTGGGCCACAGCGAGAGCAGCGAGGATTTAAGCGCCTATGCCAGCATCAGTTTCCAGAAGCCGCCAGGCGACCTCCAGTAG

>Ory_af1 [Aardvark (Orycteropus afer afer) Irs1]

ATGGCGAGCCCTCCGGACGACGGCTTCTCGGACGTGCGCAAGGTGGGTTACCTGCGCAAACCCAAAAGCATGCACAAGCGCTTCTTCGTGCTGCGGGCGGCCAGCGAGGCGGGGGGCCCGGCGCGCCTCGAGTACTACGAGAACGAGAAGAAGTGGCGGCACAAGTCGAGCGCCCCCAAACGCTCGATCCCCCTCGAGAGCTGCTTCAACATCAACAAGCGGGCGGACTCCAAGAACAAGCACCTGGTGGCTCTCTATACCCGGGACGAGCACTTCGCCATCGCGGCCGACAGCGAGGCCGAGCAGGACAGCTGGTACCAGGCCCTGCTGCAGCTGCACAGCCGTGCCAAGGGGCACCACGACGGGGCCGTGGCGCCCGGCCTGGGAGGCGGCGGGGGCAGCTGCGGCGGCAGCGCCTGCCTGGGCGAGGCTGGGGAGGACCTGAGCTATGGGGACGTGCCCCCAGGCCCTGCCTTCAAGGAGGTCTGGCAGGTGATCCTGAAGCCCAAGGGCCTGGGGCAGACGAAGAACCTGATCGGCATCTACCGCCTCTGCTTGACCAGCAAGACCATCAGCTTCGTGAAGCTCAACTCGGAGGCGGCCGCCGTGGTGCTCCAACTGATGAACATCCGACGCTGTGGCCACTCGGAGAACTTCTTCTTCATCGAGGTGGGCCGCTCCGCGGTGACCGGGCCCGGGGAGTTCTGGATGCAGGTGGATGACTCGGTGGTGGCCCAGAACATGCACGAGACCATCCTGGAGGCCATGCGGGCCATGAGCGACGAGTTCCGCCCGCGCAGCAAGAGCCAGTCCTCCAACTGCTCCAACCCCATCAGCGTCCCCCTGCGCAGGCACCACCTCAACAACCCGCCCCCCAGCCAGGTGGGCCTGACGCGCCGCTCGCGCACCGAGAGCATCACTGCCACCTCCCCGGCCAGTATGGTGGGCGGCAAGCCAGGCTCCTTCCGGGTCCGCGCTTCCAGCGATGGCGAAGGCACCATGTCCCGCCCGGCCTCGGTGGACGGCAGTCCCGTGAGTCCGAGCACCAACAGGACGCACGCCCACAGGCATCGCGGCAGCTCCAGGCTGCATCCTCCGCTCAACCACAGCCGCTCCATCCCCATGCCTACTTCCCGCTGCTCGCCTTCGGCCACCAGCCCGGTCAGCCTCTCGTCCAGCAGCACCAGTGGCCATGGCTCCACCTCCGACTGCCTCTTCCCACGGCGGTCGAGTGCTTCGGTGTCCGGTTCCCCCAGCGATGGCGGGTTTATCTCCTCGGATGAGTATGGCTCCAGTCCCTGTGATTTCCGAAGTTCCTTCCGCAGTGTCACCCCGGATTCCCTGGGCCACACCCCACCAGCCCGCGGTGAAGAGGAGCTGAGCAACTATATCTGCATGGGCAGCAAGGGGACCTCCACTCTGACCGCCCCTAATGGCCACTACATCTTGGCTCGGGGCGGCAATGGCCACCGCTGCATCCCGGGCGCTAGCTTGGGTACCAGCCCGGCACTGCCTGCCGATGAAGCAGCCGCTACCGCGGATCTGGAGAATCGCTTCCGAAAGAGGACTCACTCTGCAGGCACGTCCCCCACCATTTCCCACCAGAAGACGCCGTCTCAGTCCTCCGTGGCGTCCATCGAGGAGTATACCGAGATGATGCCCGCCTACCCACCAGGAGGGGGCAGTGGAGGCCGGCTGCCCGGCTACCGGCACTCGGCCTTCGTGCCCACGCACTCCTACCCCGAGGAGGGTCTGGAAATGCATTCCTTGGAGCGGCGTGGGGGCCACCACCGCCCAGACGCCTCCACCCTCCACACCGACGATGGCTACATGCCCATGTCCCCCGGGGTGGCCCCAGTGCCCAGCGGCCGGAAGGGCAGCGGTGACTACATGCCCATGAGCCCCAAGAGTGTGTCTGCCCCACAGCAAATCATCAACCCCATCAGACGCCATCCGCAGAGAGTGGACCCCAATGGCTACATGATGATGTCCCCAAGCGGCAGCTGCTCCCCGGACATTGCAGGGGGCCCCAACAACAGCAGTGCTACCCCTTCAGGCAGCAGCTATGGGAAGCTGTGGACAAATGGCGTAGGGGGCCATCATTCTCACCCCCTCCCTCACCCCAAACCACCTGTGGAGAGCAGTGGTGGCAAGCTCTTGCCTTGCACAGGTGACTACATGAACATGTCACCGGTGGGGGACTCCAACACCAGCAGCCCCTCCGACTGCTACTATGGCCCCGAGGACCCCCAGCACAAGCCAGTCCTCTCCTACTACTCATTGCCAAGGTCCTTTAAGCACACCCAGCGCCCTGGGGAGCCAGAGGACAGTGCCCGGCACCAGCATCTGCGACTGTCCTCCAGCTCTAGCCGCCTTCTCTGTGCTGCCACGGCAGACGACTCTTCCTCCTCCACCAGCAGTGACAGTCTGGGAGGAGGATACTGTGGGCCTCGACCGGAGCCTGGCCACCCGCATCTCCACCATCAGGTCCTGCAACCCCATCTGCCTCGAAAGGTGGACATGGCTGCCCAGACCAACAGCCGCCTGGCTCGGCCCACAAGGCTGTCTCTGGGGGATCCTAAGGCCAGCACCTTGCCTCGGGCCCGCGAGCAGCCGCAGCAGCCCCTCCTGCACCCTCCGGAGCCCAAGAGCCCAGGGGAATATGTGAATATTGAATTTGGGAGTGACCAGCCTGGCTACTTATCTGGCCCCGTGGCTTCGCACAGCTCGCCTTCTGTCAGTTGTCCAGCCCAGCTCCAGCCAGCCCCCAGAGAGGAAGGGACCGGCACAGAGGAGTACATGAACATGGACCTGGGGCCAGGCCGCAGGGCGCCCTGGCAGGAGAGCGCCGGGGTGGAGATGGGCAGAGTGGGCGCTGCGCCTCCCGGGTCTGTGAGCGTCTGCAGGCCCACGCGGGCTGTGCCCAGCAGCCGCGGTGACTACATGACCATGCAGATGGGCTGTGCCCGGCAGAGCTACGTGGACACCTCGCCGGTCGCTCCTGTCAGCTATGCTGACATGCGGACGGGCATTGCTACAGAGGATGTGAGCCTTCCCGGGGCCACAGCAGCTGCTCCCTCCTCATCGTCAGCAGCCTCTGCTTCCCCCACTGCACCTCAAGGAGCAGCTGAGCTGGCTGGCCGCCCTTCACTGCTGGGGGGCCCACAGGGACCTGGTGGCATGAGCGCCTTCACCCGGGTGAACCTCAGCCCCAACCGTAACCAGAGTGCCAAAGTTATCCGGGCGGACCCTCAAGGGTGCCGGAGGAGGCATAGCTCCGAGACCTTCTCCTCGACACCCAGTGCCACCCGGGCAGGCAGCATTGTGCCCTTCGGAGGGGGTGCTGTGGCAGGGGGCAGTGGTGGCAGCAGGAGCGGCAGTGAGGATGTGAAGCGCCACAGTTCTGCTTCTTTTGAGAATGTGTGGCTGAGGCCCGGGGAGCTTGGGGCAGCCCCCAAGGAGCCAGCCCAGGTGTGTGGGGCCGCTGGGGGCTTCGAGAACGGTCTTAACTACATAGACTTGGATTTGGTCAAGGACTTTAAACAGCGCCCTCAGGAGTGCCCCCCTCCCCCACAGCCCCCTCCCCCGCTGCCCCCTCATCAGCCCCTTGGCAGCAGTGAGAGCAGCCTGACCAGCCGCTCCAGCGAGGACTTGAGCGCCTATGCCAGCATCAGTTTCCCCAAGCAGCCAGAGGATCTCCAGTAG

>Mon_do1 [Opossum (Monodelphis domestica) Irs1]

ATGGCGAGTCCACCGGAGGGGGACGGCTGCTTCTCAGATGTGCGGAAGGTGGGCTACCTCCGCAAACCCAAGAGCATGCACAAACGCTTCTTCGTCCTGCGGGCAGCTAGTGAGGCCGGGGGTCCAGCCCGCCTGGAGTACTACGAGAACGAAAAGAAGTGGAGGCACAAATCGGGTGCCCCCAAGCGCTCTATCCCGCTAGAGAGCTGCTTCAACATCAACAAGCGGGCTGACTCCAAGAACAAACACCTGGTAGCCCTCTACACCCGGGATGAGCATTTTGCCATTGCCGCGGACAGCGAGTCGGAGCAGGAGAGCTGGTACCAGGCGCTCCTGCAGCTCCACAACCGGGCCAAGGGGCATCACTTGCACCATTCCCACCACGAGGCTGCAGCTTTTGGAGTGGGAGGGGGAGGAGGAGGAGGGAGCTGCAGCGGCAGCTCTGGCCTGGGAGAAGGGGGTGAGGACAGTAGCTATGGTGAAATGGCCCCAGGACCCGCATTCAAGGAGGTTTGGCAAGTGATCCTGAAGCCTAAGGGCTTGGGGCAGACAAAGAACCTGATTGGTATCTACCGGCTCTGCTTGACCAGTAAGACCATCAGCTTTGTGAAGCTCAACTCAGAGGCAGCCGCAGTGGTCCTCCAATTGATGAACATTCGCAGGTGTGGCCACTCGGAAAACTTCTTTTTTATTGAGGTGGGGCGCTCTGCCGTGACTGGGCCTGGGGAGTTCTGGATGCAAGTGGATGACTCTGTAGTTGCCCAAAATATGCATGAGACTATCCTGGAAGCCATGCGGGCCATGAGTGAGGAGTTCCGGCCTCGGAGCAAGAGCCAATCGTCCTCTAACTGCTCCAACCCCATCAGTGTCCCTCTTCGCAGGCATCACCTCAATAACCCCCCACCCAGCCAAGTGGGGCTCACTCGGAGGTCCCGGACTGAGAGTGTCACTGCCACATCCCCAGCTAGTGTGGTTGGTGGGAAGCCCTGCTCTTTCCGTGTCCGGGCTTCTAGTGATGGAGAAGGCACCATGTCCCGGCCAGCTTCAGTGGACGGGAGCCCTGTGAGTCCCAGTACCAACCGGACCCATTCACACCGACATCGGGGCAGCTCCCGGTTGCATCCCCCTCTTAACCACAGCCGCTCCATCCCCATGCCCTCCTCCCGATGTTCCCCTTCAGCCACAAGTCCAGTCAGCTTGTCATCCAGCAGCACCAGTGGCCATGGATCAACTTCAGACTGCCTCTTCCCTAGGAGGTCCAGTGCCTCTGTGTCTGGCTCCCCCAGTGATGGGGGCTTCATCTCTTCCGATGAATATGGCTCCAGCCCTTGTGACTTCCGAAGTTCTTTCCGGAGTGTCACCCCTGATTCCTTGGGTCATACCCCACCAGCTAGGGGCGAGGAAGACCTGAGCAACTATATCTGCATGGGAGGCAAAAACACACCTAACTTGATGGCCCCCAATGGCCACTATAACCTCTCCAGGGGTGGCAATGGGCACCGGTATACCCCCGGAGCTGGCCCCAGCCCTGCTACAGTTGGAGAGGAGGCAGCCACTGCTGCAGACCTGGAGAAAAGTTTCAGAAAGAGAACTCACTCAGCAGGTACTTCTCCCACCATTTCCCACCAGAAGACCCCCTCACAATCCTCCGTTGCCTCCATTGAGGAGTACACAGAGATGATGCCTTCCTACCCACCCTGTTCCTCAGCGGGCACCGGGAACCGGATGCAAGCCTACCGGCACTCAGCCTTTGTGCCCACCCATTCCTATCCCGAGGAGTGTCTGGAAATCCATCCCTTAGAGGAGCGGGGAGGCCACCATCACCGGGGGGACACTCCCGGGCTACACACCGATGATGGCTACATGCCCATGTCTCCTGGGGTAGCCCCAGTGCCCAGCAGCCGAAAGAGTGGGGGAGATTACATGCCCATGAGCCCCAAGAGCGTGTCTGCCCCGCAGCAGATCATTAACCCTGGCCGGAGGCATTCCCAAAGGGTAGATCCCAATGGCTACATGATGATGTCCCCCAGTGGCAGCTGTTCCCCGGATGGCGCAGGGGGCTCAGGCAATGGCAATGCGGCCACCCCTGGGAACAGCTATGGCAAGTTATGGACAAACGGGGTTGGTGGCCACCATCACCATCACCACGGGCACCCCAAGCTCTCCATGGAGAGCAGTGATGGGAAGCTGCCCTGCAGCAGCGACTACATTAACATGTCCCCAGCCGGAGACTCGGCCACCAGCAGCCCCTCCGAGGGCTACTATGGACCAGATGACCCGCAGGGCAAGGCCATCTACTCCTACTACTCACTGCCTCGCTCCTTCAAGCACACCCAGCAGCAGCAGCGTCGAGGGGAGCCCGAAGATGGCGGCCGGCTCCACCACCTGCGCCTCTCTGCCAGCTCCGGCCGCCTTCTCTACGCCACAGCGGCGGAGGACTCGTCTTCTTCCGCCAGCAGTGACAGCCTGGGAGGAGGTGGAGGTGGCCAGGAAGGGGTGCACGGCCACCTCCACCATCAGGCCCTACACCAGCACCTGCCCCGAAAGATGGATCTAGTTGCCCAGACCAAGAGCCGCCTGACTCGGCCCACAAGACTCTCTCTGGATGGCCCCAAGGCCAGCACCTTACCCAGGGCTCGGGAGCAGCCCCAGCAGCCTCTTCTTCCTCCAGAGCCCAAGAGCCCGGGAGAATACGTGAATATTGAGTTTGTAGGTGAGCAGCCCAGCTACCCGCATGGCTCAGCCATCTCCCTCTGCTCTCCCACCGTCAGGTGCCCAGCCCCGCGCCAGCCGGCCCCCAGAGAGGAGGACCCTGGCTCCGAGGAATATATGAACATGGACTTGAGGCCTCCCAGGAGGCCGGCCTGCCAGGAAGGCTTTGGGTCGAAGGCGGGAAGAGCCTGCCCCCTGCCCACCGGGGTAGGTGGTGTGTGCAGGCCCAGCCGGGCACTGCCAAACAGCCAGGATTACGTGACCATGCAGGTGGGTGGGCCCTGCTCAGGCTGTGCCGATGCTTCTCTCAGCTATGTGGTGATGCAGACAAGCAGGGCTTCCGAGGAGTCCAGCGTCCCAGGCGCTGCCTCTGCCGCCCCTTCCCCAGCTTTTACTGCCGCCACCCCCTCCCTCCCCCAGCACCCAGGCCAGGCAGAGCTGGCTAGCCGCCCCTCCCTGCTAGGAGGTCCAAAGGGACCCGGAGGGATTAGCGCCTTCACTCGAGTGAACCTCAGCCCTAGCCGCAACCAGAGTGCCAAAGTGATCCGGGCTGACCCCCAAGGATGCCGGAGGCGGCACAGCTCCGAGACTTTTTCCTCCACCCCCAGTGCTGCCCGGGGCAGTAACGTTGCAGTGCCCTTTGGGGCGGCTGGCGCAGGTGGCTCTGGGACCAGCGGCGGCGGCAGCAGCGAAGATGTTAAACGCCACAGTTCTGCCTCCTTTGAAAATGTCTGGCTAAAGCCTGGGGATGTAGGGGGCCCACCCAGCAGGAAGGAGACCCCCCAGATGAGTGGGGGTGCCCCCGCGGGAGCAGCGGCCGGTTTGGAGAACGGACTCAACTACATCGACCTGGATTTGGTCAAGGATTTTAATCACCGGCCCCAGGAGTGCCCCCCTCTGCTGCAGCCTCCTCATCAGCCCTGTGGCAGTGGTGGGGGCAGTGGCAGCGGCTGTTCCAGTGAGGATCTAAGCGCCTATGCTAGCATCAGCTTTCAGAAGCAGCCAGAGGACCTTCCATAG

>Sar_ha1 [Tasmanian devil (Sarcophilus harrisii) Irs1]

ATGGCGAGCCCACCGGAGGGGGACGGCTGCTTCTCCGACGTGCGGAAGGTGGGCTACCTCCGCAAACCCAAGAGCATGCACAAACGCTTCTTCGTCCTGCGGGCGGCCAGTGAGGCCGGGGGGCCGGCCCGCCTGGAGTACTATGAGAACGAGAAGAAGTGGAGGCACAAATCCGGTGCCCCCAAGCGCTCTATCCCGCTGGAGAGCTGTTTCAACATCAACAAGCGGGCTGACTCCAAAAACAAACACCTGGTAGCCCTCTACACCCGGGACGAGCATTTCGCCATTGCCGCGGATAGCGAGTCGGAGCAGGAGAGCTGGTACCAGGCGCTCCTGCAGCTCCACAACCGGGCCAAGGGACATCACTTGCACCATTCCCACCACGAGGCTGCAGCTTTTGGAGTGGGAGGGGGAGGAGGCGGAGGGGGGAGCTGCAGCGGCAGCTCTGGCCTGGGAGAAGCCGGGGAGGACAGTAGCTATGGGGAAATGGCCCCAGGACCAGCATTCAAGGAGGTTTGGCAAGTGATCCTGAAGCCTAAGGGCTTGGGGCAGACAAAGAACCTGATTGGCATCTACCGGCTCTGCCTGACCAGTAAGACCATCAGCTTTGTGAAGCTGAACTCGGAGGCTGCCGCAGTGGTCCTCCAACTGATGAACATTCGCAGGTGTGGTCACTCCGAAAACTTCTTTTTTATTGAGGTGGGGCGCTCTGCGGTGACTGGACCTGGGGAGTTCTGGATGCAAGTGGATGATTCCGTGGTTGCCCAGAATATGCACGAGACCATCCTGGAAGCTATGCGGGCCATGAGTGAGGAATTCCGGCCTCGGAGCAAGAGCCAGTCGTCCTCCAACTGCTCCAACCCCATCAGCGTACCCTTACGCAGGCATCACCTCAATAATCCACCCCCCAGCCAGGTGGGGCTCACCCGCAGATCCCGGACTGAGAGCGTCACCGCCACATCACCCGCTAGTGTCGTGGGTGGGAAGCCCTGCTCTTTCCGAGTCCGGGCATCCAGTGATGGAGAAGGCACCATGTCCCGACCGGCCTCGGTGGATGGGAGTCCCGTAAGCCCCAGTACCAACCGGACTCATTCACACCGGCATCGGGGCAGCTCCCGCTTGCACCCGCCCCTCAACCACAGCCGCTCCATCCCGATGCCTTCCTCCCGATGTTCCCCTTCGGCCACGAGTCCGGTCAGCTTATCGTCCAGCAGCACCAGTGGCCACGGGTCGACCTCTGACTGTCTCTTCCCAAGGAGGTCCAGTGCTTCCGTGTCCGGCTCACCCAGCGATGGGGGTTTCATCTCTTCCGATGAATACGGCTCTAGCCCGTGTGACTTCCGAAGTTCCTTCCGCAGCGTCACTCCGGATTCCCTGGGTCACACCCCACCGGCCAGGGGGGAAGAAGATCTGAGCAACTATATCTGCATGGGAGGCAAAAACCCGCCTAACTTGACTGCCCCCAATGGCCACTATAACCTATCCAGAAGTGGCATCGTGCACCGCTATACCCCGGGAGCTGGCCCGAGTCCTGCCACTGTGGGAGATGAGGTAGCCACTGCTGCAGAGCTGGAGAAGAGTTTCCGCAAGAGAACACACTCAGCAGGTACTTCTCCTACTATTTCCCACCAGAAGACACCTTCTCAGTCCTCCGTAGCATCCATTGAAGAGTACACAGAGATGATGCCTTCCTACCCGCCCTGTTCAGCGGCTGGCAGTGGGAACCGGGGCCAAGCCTACCGCCACTCAGCCTTTGTGCCCACCCACTCCTATCCTGAGGAGTGTCTGGAGATCCATCCCTTGGAGGATCGGGGAGGCCACCACCATCACCGGGTGGATACTCCGGTGCTACATACAGACGACGGCTACATGCCCATGTCTCCTGGGGTGGCTCCGGTGCCCAGTGGCCGAAAGAGCGGTGGAGATTACATGCCCATGAGCCCCAAGAGCGTATCTGCTCCCCAGCAGATCATTAACCCCAGCAGGCGCCATTCGCAGAGGGTAGACCCCAATGGCTACATGATGATGTCTCCCAGTGGCAGCTGTTCCCCAGACAGTGCAGGTGGCTCTGGCAGCAGCAACACGGTCACTTCTGGGAACAGTTACGGCAAGTTATGGACAAACGGGGTGGGTGGCCACCACCACCACCACGTTCACCCGAAGTTCTCTGTGGAGAGCAGTGACGGCAAGCTACCTTGCAGCAGCGATTACATCAACATGTCCCCTGCTGGGGACTCTGTGACCAGCAGCCCCTCAGACGGTTACTATGGCCCAGATGATCCACAAAACAAGGCCATCTACTCTTACTACTCATTGCCAAGATCCTTCAAACACACTCAGCAGCAGCAGCAGCAACAGCAGNNTGAAGATGGTGGCCGGCTCCCTCACCTGCGCCTCTCGGTCAGCTCCGGTCGCCTCCTGTACGCCACTGCAGCCGAGGACTCTTCCTCTTCTGCCAGCAGTGATAGCCTGGGAGGAGGTGGAGGCACACAGGAGGGGGCCCATGGCCATCTCCATCATCAGGCCCTGCCACAGCACCTGCCCCGGAAAGCAGACATGGTTGCCCAGACCAAGAATCGCCTAACCCGGCCCACCAGGCTGTCTCTGGATGGCCCCAAGGCCAGCACCTTGCCTAGGGCTAGGGAGCAGCCACAGCAGCCTCTTCTGCCCCCAGAGCCCAAGAGCCCCGGAGAATATGTGAACATTGAGTTTGTAGGGGACCAGCCGGGCTACTCCCACGGCTCGGCCATCTCCCTCTGCTCGCCCACAGTCAGGTGCCCATCCCAGCGACAGCCGGCCCCCAGAGAAGATGAGGCTGGCTCAGAGGAATACATGAACATGGACTTGAGGCCTCCTCGGAGGCCTGTCTGTCAGGAAAGCTTTGTGGCAAAGGCTGGCAGAGCATGCCCCCTGCCCACCGGGGCGGGTGGGGTGTGTAGGCCCAGTCGCGTGGTGCCAAACAGCCAGGATTACGTGACCATGCAGGTGGGTGGGCCCTGTCCTGGCTGTGCAGATGCCTCCCTCAGCTATGTGGTCATGCAGGCAAGCAAGGCTTCGGAGGAGCCCAGTGTCCCAGCTGCTGCGGCTCCTGCCCCTTCCCTTCCTTTTACCGCAGCGCCCCCCTCTCTCCCCCAGCACCAAGGACAGGCAGAGCTGGCTAGCCGCCCATCCCTGTTAGGAGGTCCGAAGGGACCCGGAGGGATTAGCGCCTTTACCAGAGTGAACCTCAGTCCCAGCCGCAACCAGAGTGCCAAAGTGATCCGGGCTGACCCCCAAGGGTGCCGGAGGCGGCACAGCTCGGAGACCTTTTCCTCCACCCCTAGTGCTTCTCGAGGCAGTAACGTCGCGGTGCCCTTTGGGGCCGCTGCCGCAGGTGGCTCTGGGGCCACTGGCAGCAGCGAAGATGTTAAACGCCACAGTTCTGCCTCCTTTGAAAATGTCTGGCTAAAGCCTGGGGATGTCGGGGGCCCACCCAGCAGGAAGGAGACATCCCAGATGAGCGGGGCTGCAGCGGGTTTAGAAAATGGACTCAACTACATTGACCTGGATTTGGTCAAGGATTTTAATCACCGGCCCCAGGAGTGTCCCCCTCTGCTGCAGCCTCCTCATCAGCCCTGTGGCAGCGGCGGAGCCAGTGGGAACGGCTGCTCCAGTGAGGATCTAAGCGCCTATGCGAGCATTAGCTTTCAGAAGCAGCCCGAGGACCTTCCATAG

>Tha_si1 [Garter snake (Thamnophis sirtalis) Irs1]

ATGGCTAGCCCCACAGATAATAATGAGGGCTTCTTCTCCGATGTCAGGAAGGTGGGTTACTTGCGCAAACCCAAGAGCATGCATAAGCGCTTCTTCGTGCTGAGAGCAGCCAGTGAGTTGGGACCCGCCCGGCTGGAGTACTATGAGAATGAAAAGAAATGGAGGCACAAATCAGGGGCTCCCAAGCGCTCCATTCCTCTGGAGAGCTGCTTCAATATAAACAAGAGGGCAGATTCCAAAAATAAACATCTGGTGGCTCTCTACACCAAGGACGAGCACTTTGCCATTGCTGCTGACAATGAGCTTGAGCAAGAAAGCTGGTACCAGGCACTGCTGCAATTGCACAACCGTGCCAAGAGCCATCACCACCACCACCATCAGCACCATCACCATCACCACCACAGAGATGTCACAGTGGGAGGTGGGACTTTGGGAATGGGAGAGGCAGGGGAGGATAGCTATGGCGATACGGCTCCTGGCCCAGCTTTCAAAGAGGTCTGGCAAGTGATCCTGAAACCAAAGGGTTTGGGGCAAACCAAGAACCTGATCGGAATCTACCGCCTGTGCCTAACCAACAAGACCATCAGTTTTGTCAAGCTAAATTCCGATGCGGCAGCTGTTGTGTTGCAGCTGCTCAACATCCGGCGGTGTGGCCATTCGGAGAATTTTTTTTTCATTGAGGTGGGGCGCTCTGCTGTCACGGGACCAGGTGAGTTCTGGATGCAGGTAGACGACTCGGTAGTAGCACAGAATATGCATGAGACCATCTTGGAGGCTATGCGGGCAATGAGTGAGGAATTTCGGCCTCGGAGCAAAAGCCAGTCGTCTTCCAATTGCTCCAATCCCATATCTGTGCCTCTCCGCAGCAGGCATCATGTCAACAACCCACCCCCCAGCCAGGTGGGACTTACTCGTAGGTCCAGGACTGAGAGCATAACTGCCACTTCTCCTGCTGGCAGTGGCGTGGGAGGAGGGATTGGGGGCAGACCCAGCTCTTTCCGCGTCAGAGCCTCTAGTGATGGAGAAGGTACCATGTCCAGACCAGCTTCGGTGGATGGAAGTCCGATGAGTCCAAGTGCCAACCGATCCCACTCACATAGGCACCGTGGCAGCTCCAGGCTTCACCCCCCTCTCAACCACAGTCGCTCCATCCCAATGCCTTCCTCACGCTGTTCCCCTTCAGCCACTAGTCCAGTCAGCCTGTCATCCAGCAGTACCAGTGGCCATGGGTCAACTTCAGACTGTCTTTTCCCACGTCGATCCAGTGCCTCAGTCTCTGGCTCTCCCAGTGATGGAGGTTTCATCTCCTCTGATGAATATGAATCAAGCCCCTGTGACTTTCGCAGCTCGTTTCGTAGTGTTACCCCTGATTCCCTGGGACACACCCCTCCTGCCCGGGGAGATGAGGAGCTTAGCAACTATATATGTATGGGGGGGAAGGCTGCCTCATCCTGCTGCAGTATCACAACTCCTAATGGTCATTTTGCCCCCCGTGCCTGCCATCTGCAGCAGCAAAGTCGCTACCCTGCTGTCCCATGTTGCCTCCGACTTGGAAATGAGGATGTAGGTGATTTGGAAAAAAGCTTCAGAAAGCGGACACATTCTGCTGGAACCTCTCCTACCATCTCCCATCAGAAGACTCCTTCCCAGTCCTCCGTGACCTCCATTGAAGAGTATACTGAGATGCTTCCCTCGTATTCTTGTAGTGGCAACAAGTTTTCTTCTTATCGACATTCTGCCTTTGTGCCAACCCAGTCTTATCCCGAAGAGTGTCTAGAGATGCACCAGGTGGAGGGAAACCACCACCGGACCAACTCTGATGATGGCTACATGCCAATGTTGCCAGGAGTGGCACCTATGCCCAGTGGAGGTAGTACGTCCAAAGGTGGGGATTACATGCCCATGAGCCCCAAGAGTGTGTCTGCTCCACAACAGATCATCAATCCTGGGAGAGGAGGTCGCCATGTGCAGGCCATGGTGGACTCAAATGGCTACATGATGATGTCTCCCAGTGGCAGTTGTTCTCCAGACAGTGGTCCCACTGGCTACAGCAAGCTATGGATAAATGGGACTAATAATCATACAAAACTCTCAGTCGAGAGTAATGAGGGCAAGTTGCCAAGTGGAGGCAGTGATTATATTAATATGTCTCCAGCCAGTGGTTCTGCTACCAGCACCCCTCCAGACTGTTACTTTACCAGCCCAGGACCTCCAGGCCCAGAAGAACCACTAGTGCATGGTTCTGCCCAGTCCCAACATAAACCCATCTATTCCTATTTCTCCTTGCCACGTTCTTTCAAGCACAACCAACGCAAGACAGGCAAAGAAGAAAACGCCCAGATGCATATGTCCTTCAGCTCTGGCCGCTTGCTTTATGCCGCTGCTGAGGATTCTTCTTCCTCTACTAGTAGTGATAGCCTTGGCTGCCCTGGGGGACAAGAGGGTGGTGGGTATTCTCTTCCCATTAAAACACAGCCATTGCAGCCTGCTGCTTGTACTGTGGACACAGCTGTGCGGACCAAGAATCATCTGGCCAGACCTACTCGTTTGTCCCTGGATGGCCCAAAGGCCAGCACTTTGCCTCGTACTCGGGAACAGCTCCCAGAGCCCAAGAGCCCCGGAGAGTATGTGAACATTGAGTTTAAGCAACCAGTCTTCCCCTCACCTTTATCCTACGGAGAAACTGGCTCCTGTTCAGAGGAGTATATGAATATGGATTGGGGAACTGCTTGCCCAGCCAGCTTGGCCTCCATGCAGTCAAGCCGGAGTGGGACCACCCACAGTGGCAACCGGGACTATATGAGCATGCAGTTGAGTGGTGGAGCACCGTATGTAGTCTGTGCCCACACCCCATCACCTTCCTCCCCGGCCCTCCTACTGAGTTATTCTGAGAAAAGCCCACCACCCATGCAGCTGCCTTCTCCGCCTTCTCAGGCTCAGCCAAGCAATCTGGCTGAGATTTTACCCCATTCCTGCTCCTCATCTATGATTGGGGGCCCAGGTGCAAACAGCGCCTTTACACATGTCAGTCTTGGTCCCAATCGCAGCACCCAGAGTGCCAAAGTGATCCGTGCTGACCCCCAAGGAGGTCGGCGCCGCCATAGTTCTGAGACGTTTGCTTCTTCTGTCACTCCAAGTAGTAGTCTTGGAGCTTCAGCTCTACTCCATGGACCAGGAGGAGGTCCTGACGATCCAAAACGTCACAGTTCAGCCTCCTTTGAAAATGTCTGGCTCAAGCCTTCATCTGGGGCACTGGGAGTTGGTTCTTTGGGTCTTGGTACAGCATCACGGAGGGAGCAAGCAGCTAGCGGAACTGGGGGAGGCTTTGAGAATGGGCTGAATTACATTGACCTGGACTTAGTGAAGGATTTTAACCACAGCCAACATCACCATCGCAACCTCCACCCTCAGGAGAGTACTGGTCTGCTAGGAGTCAAGCAACCATCTCAGCAGCATCAGCCACCTAAATCTCCAAAGCAGCCTTGTGGGAGTAACCACTTGAGTGATGAATTAAGTGCATATGCCAGCATTAGCTTCCACAAGCATGAGGACATCCAGTAG

>Pyt_bi1 [Burmese python (Python bivittatus) Irs1]

ATGGCCAGCCCCACGGATAATAATGAGGGCTTCTTCTCAGATGTCAGGAAGGTGGGTTATTTGCGCAAACCCAAGAGCATGCATAAGCGCTTCTTCGTGCTGAGGGCAGCCAGTGAGTTGGGACCCGCCCGGCTGGAGTACTATGAGAACGAAAAGAAATGGAGACACAAATCGGGGGCCCCCAAGCGCTCGATTCCTCTGGAGAGTTGCTTCAACATAAACAAGAGGGCAGATTCCAAAAATAAGCATCTGGTGGCTCTCTACACCAAGGACGAGCACTTTGCCATTGCTGCTGACAATGAGCTTGAGCAAGAAAGCTGGTACCAGGCACTGCTGCAGTTGCACAACCGTGCCAAGAGCCATCACCACCATCATCACCACCACCACCATCACCACAGAGATGTCACAGTGGGAGGTGGGAGTTTGGGGATGGGAGAGGCAGGGGAGGATAGCTATGGTGACACAGCTCCTGGCCCAGCGTTCAAGGAAGTCTGGCAAGTGATCCTGAAACCAAAGGGTCTGGGCCAAACTAAGAACCTGATTGGAATCTACCGCCTGTGCCTAACCAACAAGACCATCAGTTTTGTCAAGCTGAATTCGGATGCTGCAGCTGTTGTGTTGCAGCTGCTCAACATCCGGCGGTGTGGCCACTCGGAGAACTTTTTTTTCATTGAGGTGGGGCGCTCTGCAGTCACTGGACCAGGTGAGTTCTGGATGCAGGTAGATGACTCAGTAGTAGCACAGAATATGCATGAGACCATCTTGGAGGCTATGCGGGCAATGAGTGAGGAATTCCGGCCTCGTAGCAAAAGCCAGTCGTCTTCCAATTGCTCCAATCCCATATCCGTGCCTCTCCGCAGCAGGCATCATGTCAACAACCCACCCCCCAGCCAGGTGGGACTTACCCGTAGGTCCAGGACTGAGAGCGTAACTGCCACTTCTCCTGCTGGCAGTGGTGTGGGAGGAGGGACAGGAGGCAGACCGAGCTCTTTCCGTGTCAGAGCCTCTAGCGATGGAGAAGGTACCATGTCCAGACCAGCTTCAGTGGATGGAAGTCCGATGAGTCCGAGTGCCAACCGAACCCACTCACATAGGCACCGCGGCAGCTCCAGGCTTCACCCCCCTCTCAACCACAGTCGCTCTATCCCAATGCCTTCCTCACGCTGTTCCCCTTCTGCCACTAGTCCGGTCAGCCTGTCATCCAGCAGTACCAGTGGCCATGGGTCCACTTCAGACTGTCTCTTCCCACGTCGATCCAGTGCATCAGTCTCTGGCTCTCCCAGTGACGGAGGTTTCATCTCCTCTGATGAATATGAATCAAGCCCCTGTGACTTCCGCAGCTCGTTTCGTAGTGTTACCCCTGATTCCCTGGGACACACTCCTCCTGCCCGGGGAGATGAGGAGCTTAGCAACTATATATGTATGGGGGGGAAGGCTGCCTCATCCTGCTGCAGTATCACAACTCCCAATGGTCATTTTGCCCCCCGCACCTGCCATCTGCAGCAGCAAACTCGCTACCCTGGTGTGCCATGCTGCCTTCGGCTTGGCAGTGAGGATGTGGGTGATTTGGAAAAAGGCTTCAGGAAACGGACACATTCTGCTGGAACCTCTCCTACCATCTCCCATCAGAAGACTCCTTCGCAGTCCTCAGTGACCTCCATTGAAGAATATACTGAGATGCTTCCCTCGTATTCCTGTGGTGGCAACAGGTTCTCTTCTTTCCGACATTCTGCCTTTGTGCCAACCCATTCTTACCCTGAGGAGTGTCTAGAGATGCACCAGATGGAGGGAAACCACCACCGGACCAACTCTGATGATGGCTACATGCCAATGTTACCAGGAGTGGCACCTCTGCCCAGTGGGGGTAGTACACCCAAAGGTGGAGATTACATGCCCATGAGCCCCAAGAGTGTGTCTGCTCCGCAGCAGATCATCAATCCTGGGAGAGGAGGTCGTCATGTACAGGCCATGGTGGACTCCAATGGCTACATGATGATGTCTCCTAGTGGCAGTTGTTCTCCAGACAATGGTCCCACTGGCTACAGCAAGCTGTGGATAAATGGGGCTAATAATCATACAAAACTCTCAGTGGAGAGTAATGAGGGGAAGCTGCCAAGTGGAGGCAGTGATTATATTAATATGTCTCCAGCCAGCGGTTCTGCTACCAGCACCCCTCCAGATTGTTACTTTACTAGCCCAGGACCTCCAGGCTCAGAAGAGCAACTAGTGCATGGTTCTGCCCAGTCCCAACATAAACCCATCTATTCCTATTTTTCCTTGCCACGTTCCTTCAAGCACACACAGCGCAAGACAGGCAAAGAAGAAAATGCCCAAATGCGTATGTCCTTCAGCTCTGGCCGCTTGCTTTATGCTGCTGCTGAGGACTCTTCTTCCTCGACTAGTAGTGATAGCCTTGGCTGTCCCGGAGGACCGGAGGGTGGTGGGTATCCTCTTCCCATCCAAACACAGCCATTGCAGCCTGCTGCTTGTACTGTGGAAACAGCTGTGCGGACCAAGAGCCATCTGGCCAGACCTACTTGTTTATCCCTGGATGGCCCAAAGGCCAGCACCTTGCCTCGCACTCGGGAACAGCTCCCAGAGCCCAAGAGCCCTGGAGAGTATGTGAACATTGAGTTTAAGCAACCAGTCTTCCCCTCACCTTTATCCTATGGAGAGGCTGGCTCCTGTTCAGAGGAGTATATGAATATGGATTGGGGAGCTGCTTGCCCAGTCAGCTTGGCCTCCATGCAGTCAAGCCGGAGTGGGACCACCCACAGCAGCAACCGGGACTATATGAGCATGCAGCTGAGTGGTGGAGCACAGTACGTAGTCTGTGCCCACACCCCATCACCTTCTTCCCCGGCCCTTCTGCTGAGTTATTCTGAGAAAAGCCCACCACCCATGCAGCTGCCTTCTCCACCTTCTCAGGCTCAGCCAGGCAAACTGGCTGAGGTTTTACCCCATTCCTGCTCCTCATCTATGATTGGGGCCCCGGGAACGAACAGCGCCTTTACACATGTCAGTCTTGGTCCCAGCCGTAGCACCCAGAGTGCCAAAGTGATCCGTGCTGACCCCCAAGGAGGTCGGCGGCGCCACAGTTCTGAGACATTTGCGTCTTCTGTCACTCCTAGTAGTAGTCTTGGAGCCTCAGCTCTACTCCATGGACCAGGAGGAGGTCCTGATGACCCAAAACGTCACAATTCAGCCTCCTTTGAAAACGTCTGGCTCAAACCTGCATCTGGGGCACTGGGGGTTGCTTCTTTGGGCCTTGGGACAGCAATGCGGAGGGAGCAAGCAGCTAGTGGATCTGGGGGAGGCTTTGAGAATGGGCTCAATTACATTGACCTGGACTTAGTGAAGGATTTTAACCACAGCCAACATCACCATCGCAGTCTCCACCCTCAGGAGAGTACTAGTCTGCTAGGGGTCAAGCAACCGCCCCAGCAGCATCAGCCACCTAAATCTCCAAAGCAGCCTTGTGGGAGTAACCACTTGAGTGATGAATTAAGTGCATATGCCAGCATTAGCTTCCACAAGCGGGAGGACATCCAGTAG

>Ano_ca1 [Anole lizard (Anolis carolinensis) Irs1]

ATGGCAAGCCCCACCGATCTTCATCACCACAGCGAGGGCTTCTTCTCCGATGTCAGGAAGGTGGGTTACTTGCGCAAACCCAAGAGCATGCACAAGCGTTTCTTCGTGCTGAGGGCAGCCAGTGAGTTGGGGCCTGCCCGCCTGGAATATTATGAGAATGAAAAGAAATGGAGACACAAATCAGGGGCCCCAAAGCGTTCGATCCCTCTGGAGAGCTGCTTCAACATCAACAAGAGGGCAGACTCCAAAAACAAGCATTTGGTGGCTCTCTACACCAAGGACGAGCACTTTGCCATTGCTGCTGACAGTGAGCCTGAACAGGAGAGTTGGTACCAGGCGCTGCTGCAACTGCACAACCGAGCCAAGAGCCACCATCACCAGCATCATCACCATCACCACTACCATGGGGATGTTACCGTGGGGGGTGGGAATATTGGAATGGGAGAGGCAGGAGAGGATAGCTATGGTGACATGGCTCCTGGCCCAGCGTTCAAAGAAGTTTGGCAAGTGATCTTGAAACCCAAAGGTCTGGGCCAGACTAAGAACCTGATTGGCATCTACCGCTTGTGCCTAACCAACAAAACCATCAGTTTTGTCAAGTTGAACTCTGACGCGGCAGCGGTGGTGCTGCAGCTGCTTAACATCCGGCGGTGTGGCCACTCTGAGAACTTTTTCTTCATTGAGGTGGGGCGCTCTGCAGTCACTGGACCAGGTGAGTTCTGGATGCAGGTAGATGACTCAGTGGTAGCACAGAATATGCATGAGACCATCTTGGAGGCTATGCGGGCAATGAGTGAAGAGTTCCGACCTCGTAGCAAAAGCCAGTCTTCTTCTAATTGCTCCAATCCTATTTCTGTGCCTCTCCGGAGCAGGCATCATGTCAACAACCCACCACCCAGTCAGGTGGGGCTCAGCCGTAGGTCTAGGACAGAAAGTGTGACTGCTACCTCCCCTGCTGGCAGTGGAGTGGGAGGTGGGGCAGGGGGAAAGCCCAATTCTTTCCGTGTCCGAGCATCCAGTGATGGAGAAGGCACAATGTCAAGGCCAGCATCAGTGGATGGAAGTCCAGTGAGTCCTAGTGCGAATCGGACCCACTCACATAGGCACCGCGGCAGCTCCAGACTTCATCCTCCTCTCAACCACAGTCGCTCCATCCCAATGCCTTCCTCACGTTGCTCTCCTTCTGCCACAAGCCCCGTCAGCCTCTCATCCAGCAGTACCAGTGGTCATGGCTCCACTTCAGACTGCCTGTTTCCACGTCGGTCCAGTGCTTCAGTCTCAGGCTCCCCCAGTGATGGGGGCTTCATCTCCTCTGATGAGTATGGATCAAGTCCTTGTGACTTCCGCAGCTCTTTCCGAAGTGTAACCCCTGATTCCCTGGGACACACACCACCGGCCAGGGGAGATGAGGATCTTAACAACTATATATGCATGGGAGGGAAAGCTGTCTCTTCCTGCTGCAGCATCACTGCTCCTAATGGGCAATTTGCCCCACGCCCTTGCCACCCTCAGCAGCAAAAACCCTATACTCCTTGCTGTCCCCGGCTTGGCAGTGAGGATGTGGGTGATGTGGAGAAAGGTTTCAGGAAACGGACACATTCAGCTGGCACTTCCCCTACCATCTCCCACCAGAAGACCCCCTCGCAGTCTTCAGTAGCTTCCATTGAAGAATATACTGAGATGCTCCCCTCATATTCCTGTGGTGGTAGCAGGCTCCCTTCTTTTCGACATTCTGCTTTTGTGCCAACCCATTCTTACCCTGAAGAGTGTATAGAAATGCACCAAATGGAGGGTAACCACCACCGGACCAACTCTACCCCACACACTGATGATGGCTACATGCCAATGTTGCCAGGAGTTGCTCCTTTGCCTAATGGTGGAAATACTTCCAAAGGTGGTGATTATATGCCCATGAGTCCCAAGAGTGTGTCTGCCCCACAGCAGATCATCAACCCTGGAAGAGGAGGCCGCCATGTCCAAGCCATGGTGGACTCCAATGGCTACATGATGATGTCTCCAAGTGGCAGTTGTTCTCCAGATAGTGGTCCCAGTGGTTACAGCAAACTCTGGCTGAATGGGACAAGTCATCACCCCAAACTTTCGGTGGAGAGCAATGAGGGGAAGCTGCCAACTGGTGGCAGTGATTACATTAACATGTCTCCAGCCAGTGGCTCTGCTACCAGCACCCCTCCAGATTGCTTCTTCACTGGCTCAGGAACTCCAGGCCCAGAAGAACCATCAGTGCAGGGTCCCACCCAGTCCCACCACAAGCCCATTTATTCCTACTTCTCCCTGCCACGCTCCTTCAAGCACATGCAACGTAGGACCGGTGAAGAGGCAAATACTCAGATGCGCCTGTCCTTCAGTTCTGGCCGCTTGCTGTATGCTGCTGCTGAGGACTCTTCCTCATCCACTAGTAGTGATAGCCTTGGTGGCCCTGGGGGACAGGAGGGTGTTGGGTATTCTGTTCTTACACAGCCATTACAGCAGCCTGCTCCTTCTTGTCCTGTGGACACAGCAACGCTGGCCAAGAGCCGTTTGGCTAGACCTACCCGTCTGTCCTTGGATGGCACAAAGGCCAGCACTTTGCCTCGTGCTAGGGAACAGCTCCCGGAGCCCAAAAGCCCTGGTGAGTATGTGAACATTGAATTCAAGCAGCCGCCCTTCCCATCACCTCTGCCTCATGGAGATACTGGCTCCAGTTCTGAGGAGTATATGAATATGGACTGGAGGACTGCCTGCCCAGCCAGCTTAGCACCAGGGCAGGCAAGCCAGGGAGGGGTGGGCCACATTGGTGGCCGAGACTATAGGAGCATGCAATTGGGCAGCGGAGGGCACTATGTGGTTTGTGCCCATACCCCCTCTCCTTCTCCTCCAGCTGTTCTGCTCAGTTATGCTGATATGAGGATGGGTCGTGGTAGGCCAGAGAAAAGCACACCTCCTATGGGGCTGCCCTCCCCTCATCCTCAGGCCCAGCCAGGGGATTTGGCTTCAGTCTTGCCCCACTCCTGTTCTTCATCCATGATTGGGGGCCCAGGAATGACCAGCGCCTTTACACATGTCAGCCTCAGCCCTAGCCACAGCACCCAGAGTGCCAAAGTGATCCGTGCTGACCCCCAGGGAGGCCGGCGACGCCATAGTTCTGAGACTTTTGTTTCCTCCACCACCCCTAGTGGTGGCCTGGGGGCCCCAGCTGTACCCCATGGTCCTGGAGGGGGGCCTGATGATGTGAAGCGCCACAGCTCAGCTTCTTTTGAAAATGTCTGGCTGAAGCCCATATCTGGAGAGCTGGGATCTTCCTCTGGCCACCGTATGGCAATCAGGAGGGAGCAGATAGCTGGCGGGACTGGAGGAGGTACTGAAAATGGCCTCAATTACATTGACTTGGACTTAGTGAAGGATTTTAATCGCCACCAACATCCTCATCCTCAGGAGAGCACTTCCCTGCTAGGGGTCAAGCAGCCAACGCAGCAGCAACAGCAGCACCAGCCACCTAAATCTCCAAATCAGCCTTGTGGGAGTAGCCACTTGAGTGATGAATTAAGTGCATATGCCAGCATTAGCTTCCACAAGCGGGAGGACACCCAGTAG

>Gek_ja1 [Gekko (Gekko japonicus) Irs1]

ATGGCCAGCCCCACGGAGGCCAACGAGAGCTTCTTCTCCGATGTCAGGAAGGTTGGCTACTTGCGCAAACCCAAGAGCATGCACAAGCGCTTCTTCGTGCTCAGGGCGGCCAGCGAGTCGGGACCCGCCCGTCTGGAGTACTACGAGAACGAGAAGAAATGGAGGCACAAGTCCGGGGCCCCCAAGCGCTCGATCCCGCTGGAGAGCTGCTTCAACATCAACAAAAGGGCGGACTCCAAAAATAAGCATCTGGTGGCTCTCTACACCAAGGACGAGCACTTTGCCATCGCTGCCGACAGCGAGCCCGACCAGGAGAGTTGGTACCAGGCGCTGCTGCAACTGCACAACCGGGCCAAGAGCCATCACTACCAGCACCACCACCACCATGGGGACGTTGCGGTTGGAGGGGGGAGTGTCGGGGTGGGAGAGGCGGGGGAGGAGAGCTACGGGGAGATGGCCCCTGGCCCGGCCTTCAAAGAAGTTTGGCAGGTGATTTTGAAACCGAAAGGTCTGGGCCAGACCAAGAATTTGATTGGTATCTACCGCCTGTGCCTAACCAACAAGACCATCAGTTTTGTGAAGCTGAACTCTGACGCTGCCGCGGTGGTGCTGCAGCTGCTCAACATACGGCGCTGTGGCCACTCAGAGAACTTTTTCTTCATTGAGGTGGGGCGCTCTGCAGTCACTGGACCAGGTGAGTTCTGGATGCAGGTAGATGATTCAGTGGTAGCACAGAACATGCACGAGACCATCTTGGAGGCTATGCGGGCTATGAGCGAGGAGTTCCGGCCCCGTAGCAAAAGCCAGTCTTCCTCCAATTGCTCCAACCCCATTTCTGTGCCTCTCCGCAGCAGGCATCATGTCAACAACCCCCCGCCCAGCCAGGTGGGGCTCAGCCGTAGGTCCAGGACTGAGAGCGTGACTGCCACCTCTCCTGCTGGCAGTGGTGTGGGAGGTGGAGGAGGGACGGGCAAGCCCAATTCTTTCCGTGTCAGAGCCTCTAGTGATGGAGAAGGTACCATGTCCAGGCCAGCTTCAGTGGATGGAAGCCCCGTGAGTCCAAGTGCCAACCGGACCCACTCACATAGGCACCGTGGAAACTCCAGGCTTCATCCTCCTCTCAACCACAGCCGCTCCATCCCAATGCCTACTTCACGTTGCTCCCCTTCTGCCACGAGTCCAGTCAGCCTGTCATCCAGCAGTACCAGTGGCCACGGCTCAACGTCTGACTGCCTCTTTCCACGTAGGTCCAGTGCTTCAGTTTCTGGTTCCCCCAGTGACGGGGGCTTCATCTCCTCTGATGAGTACGGATCCAGCCCCTGTGACTTCCGGAGCTCTTTCCGCAGTGTTACCCCAGACTCCCTGGGACATACCCCACCTGCCCGGGGGGAGGAGGAGCTTAGCAACTATATTTGTATGGGGGGTAAGGCTACTTCATCCTGCTGCAATGTCACAGCCCCTAATGGTCATTTCACCCCCCGCACCTGCCATCCTCAGCAGCAGACTCGCTATCCTGGTGTCCCATGCTGCCCCCGGCTTGGTAGCGAGGATGCAGGTGATCTGGATAAAGGCTTCAGGAAACGGACTCATTCAGCTGGTACTTCCCCAACCATCTCCCACCAGAAGACACCCTCACAGTCCTCGGTGGCCTCCATTGAGGAATATACTGAGATGCTCCCCTCGTATTCCTGCGGTGGTGGCAGTCGGCTCTCCTCCTACCGACATTCTGCCTTTGTGCCAACTCATTCTTACCCTGAGGAGTGTCTAGAGATGCACCACGTGGAGGGCAGCCACCACCAGACCAGTTCTACCCCACACACTGATGATGGCTACATGCCCATGTTGCCGGGGGTGGCCCCTGTCCCTAATGGTGGTACTGCTCCCAAAGGTGGTGATTATATGCCCATGAGCCCCAAGAGTGTATCTGCCCCACAGCAGATCATCAACCCTGGGAGAGGTGGCCGTCATGTACAGGCCATGGTGGACTCCAATGGCTACATGATGATGTCTCCAAGTGGGAGTTGTTCTCCGGACAGTGGTCCCACTGGCTACAGCAAGCTATGGATGAATGGGACACGACACCATCCCAAGTTATCCATGGAGAGTAATGAGGGAAAGTTGCCAAGTGGGGGCAGCGATTACATCAATATGTCTCCAGCCAGTGGGTCTGCTACCAGCACTCCTCCAGATTGCTACTTCACCAGCTCAGGACCTCCAGGCCCAGAAGAACCATCCATGCAAGGCCCCGCTCAGTCCCACCACAAACCCATCTACTCTTATTTCTCTCTCCCACGCTCCTTCAAACATGTGCAGCGCAGGACAGGCGAAGAGGGCGGCACCCAGATGCGCATGTCTCTCAGCTCCGGCCGCTTACTGTATGCTGCTGCTGCTGAGGACTCTTCCTCTTCCACTAGTAGCGATAGCCTTGGTGGCCCTGCAGGTCAGGAGGGTGGTGGGTTTTCTGTTCCTCCCCAAACACAGCCTTTGCAGCCTGCTGCTTCTTGTACTGCGGACATGGCTGTGCGGACCAAGAGCCGCCTGGCCAGACCCACCCGTCTGTCTTTGGACGGCCCTAAGGCCAGCACGTTGCCTCGTGCTCGGGAGCAGCTCCCGGAGCCCAAAAGCCCTGGTGAGTATGTGAACATTGAATTCAAGCAGCCGGCCTTTCCATCACTGTCATCCCATGGAGATGCTGGATCCAGTTCAGAGGAGTATATGAATATGGATTGGGGAGCTGCCTGCCCCTCCAGCTTGGCATCCGGGCAGTCAGGTCGGGGCGGGGCAGTTCCCACTGGTGGCCGAGACTACATGAGCATGCAGGTGGGCAGTGGGGGGCAGTATATAGTGTGTGCCCATACCCCCTCACCTTCCCCTCCGGCCCTTTTGCTCAGCTATGCCGATATGAGGATGGGCCGTGGTAGGCCAGAGAAAAGCCCACCTGCTGTGGGGCTACCTTCCCCACGTCCCCAGACTCAACCAGGTGAGCTGGCCGCAGCCTTGCCCCACCCCTGCTCCTCATCCATGGTGGGGGGCCTAGGGATGAGCAGCACCTTCTCCCACGTCAGCCCCAGCCGCAGCAGCCAGAGTGCCAAAGTGATCCGCGCTGACCCGCAGGGAGGTCGGCGGCGTCACAGTTCGGAGACATTTGCTTCCTCTACCACCCCTAGTGGTGGCCTTGGTGCCTCAGCCCTGCCCCACGGACCAGGAGGAGGTCCTGACGAGGCAAAACGCCACAGCTCAGCCTCCTTTGAAAACGTCTGGCTCAAGCCCGGGGACCTGGGGGCCTCCTTCGGCCTTGGCACGGCCATCAGGAGTGAGCCGGCTGCGGGTGGGACTGGAGGAGGCGGCGTCGAGAATGGGCTCAATTACATCGACTTGGACTTAGTGAAGGATTTTAATCAGCGCCAGCATCACCACCCCCACCCTCAGGAGAGCACTTCTCTGCTAGGGGGCAAGCAGACGACGCAGCAGCATCAGCCACCTAAATCTCCTAATCAGCCTTGTGGGAGTAGCCACTTGAGTGATGAATTAAGTGCATATGCCAGCATCAGCTTCCACAAGCGGGAGGACAGTAGCTGA

>Gal_ga1 [Chicken (Gallus gallus) Irs1]

ATGGCCAGCCCCACAGATAACAACGAGGGCTTCTTCTCGGATGTCAGAAAGGTGGGTTACTTGCGCAAACCCAAGAGCATGCATAAGCGCTTTTTCGTGCTAAGGGCAGCCAGCGAGTCTGGACCCGCCCGGCTGGAGTATTATGAGAATGAGAAGAAATGGAGACACAAGTCGGGGGCCCCCAAGCGCTCCATCCCATTGGAAAGCTGCTTCAACATCAACAAGCGGGCTGACTCCAAGAACAAGCACCTGGTGGCCCTCTACACCAAGGACGAGCACTTTGCCATCGCAGCTGACAGTGAACTGGAACAAGAGAGCTGGTACCAAGCGCTGCTGCAGTTGCACAACAGGGCCAAGGGCCACCACCACCTCCACCACCACCACCATCACCACCACAGCGATGTCACCTTCGGGGGCAGCAGCGTGGGGCTGGGGGAAGCAGGTGAGGACAGCTATGGCGAGGTAGCCCCTGGTCCGGCTTTTAAGGAAGTTTGGCAAGTAATTCTGAAGCCTAAGGGGCTAGGCCAGACAAAGAACCTGATTGGCATCTACCGCCTGTGCCTGACTAACAAGACCATCAGCTTTGTGAAGCTGAATTCGGATGCGGCTGCTGTGGTGCTGCAGCTGCTCAATATCCGCCGCTGCGGACACTCCGAGAACTTCTTCTTCATCGAGGTGGGACGCTCGGCGGTGACCGGGCCTGGGGAGTTCTGGATGCAGGTGGACGACTCGGTGGTGGCGCAGAACATGCATGAAACCATCCTGGAGGCCATGCGAGCCATGAGCGAGGAATTCCGACCCCGCAGCAAGAGCCAGTCCTCCTCCAACTGTTCCAATCCCATCTCCGTGCCCCTTCGTAGCAGGCACCACATCAACAACCCTCCGCCCAGCCAAGTGGGGCTCAGTCGCCGGTCCAGGACTGAGAGTGTTACAGCCACCTCCCCAGCTGGCGGTGGGGGCGGAGGTATGGGTGGCAAACCCAGCTCTTTCCGGGTCCGCGCATCCAGCGATGGGGAAGGCACCATGTCAAGGCCTGCCTCGGTGGATGGTAGCCCTGTTAGCCCCAGTGCCAACCGAACCCACTCGCACAGACACCGTGGCAACTCCAGGCTCCATCCTCCGCTCAACCACAGCCGTTCCATCCCGATGCCTTCCTCACGCTGCTCCCCGTCAGCCACCAGCCCAGTCAGCCTGTCGTCCAGCAGCACCAGTGGCCACGGCTCCACGTCAGACTGCCTGTTTCCACGAAGGTCCAGTGCTTCGGTTTCTGGCTCCCCTAGTGATGGCGGATTTATTTCTTCTGATGAATATGGTTCTAGCCCATGTGACTTCCGCAGCTCTTTTCGCAGCGTGACCCCGGATTCGCTAGGACACACCCCACCAGCTCGGGGCGATGAAGAGCTCAACTACATCTGCATGGGGGGGAAGGCCGCCTCGTCCTGCTGCAGCCTGGCAGCTCCCAATGGCCACTTCATCCCACGCACCTGCCACCCGCAGCAGCAGCCCCGCTACCCCAGCACGTCGTGCTGTCCCCGAGCCGGTAGCGAGGACGTTGCTGACTTGGACAAGGCATTCAGGAAACGGACTCACTCTGCAGGCACTTCGCCCACCATCTCCCACCAGAAGACGCCTTCCCAGTCTTCGGTGGCCTCCATTGAGGAGTATACGGAGATGTTGCCTTCTTACCCCTGCGGCGGCAGCCGGCTGCCCTCCTACCGGCACTCGGCCTTCGTGCCCACTCACTCCTACCCTGAGGAGTGTCTGGAGATGCACCACCTGGATGGCAGCCATCATCGGACCAACTCCGCTCCGCACACAGATGATGGCTACATGCCCATGTCCCCCGGTGTTGCCCCCTTGCCCAGCGGTGGGGCTGCCCCCAAGGGCGGTGACTACATGCCCATGAGCCCCAAGAGCGTGTCGGCCCCGCAGCAGATCATCAACCCTGGCAGAGGGGGCCGCCACCCTCCAGCCACGGTGGACTCCAACGGCTACATGATGATGTCCCCTAGCGGCAGCTACTCCCCGGACAGCGGCTCTGCGGGCTACGGCAAGATCTGGACGAACGGTGCCGGCCACCACCCGAAGCTCTCGGTGGAGAGCAACGAAGGGAAGCTGCCCTGCGGCGGCGGCGACTATATCAACATGTCCCCGGCCAGCGGCTCCACCACCAGCACGCCGCCCGACTGCTACTTCGGGGCGGCGGGGCAGCCGGGCGTCGAGGAGGCGGCCGCGGCCCTCCACAAGCCCATCTACTCCTACTTCTCGCTGCCGCGCTCCTTCAAGCACGTGCACCGGCGGGGCGGCGGGCCGGCGGGCGAGGAGGGCAGTCCCCAGCCCCGCGTGGCGCTCGGCTCCGGCCGCCTCCTCTACGCCGCCGAGGACTCGTCCTCCTCAACCAGCAGCGACAGCCTGGGCGGCCCCGGCGGCCCCGAGGGTCCCGCGTCGCACTCGCAGCCCCCGCGCAAGGTGGACACGGCCGTGCAGACCAAAGGCCGCCTGGCGCGACCCACGCGGCTGTCGCTGGGCGGCCCCAAGGCCAGCACCCTGCCGCGGGCCCGCGAGCAGCCCCCGCTGCTGCTGCCCCCGGAGCCCAAGAGCCCCGGCGAGTACGTGAACATCGAGTTCGTCCCCGGGGACAAGCCGCCTTTCCCCTCGGCCGCGCCGGGGCTGCCACGGCCGCCGGGCGGGGAGGCCGCCGAGGAGTACATGAACATGGAGCTGGGGCCGCCCCGCGCCCGCTGCCCCGGCGCCTTCGCCGCCGCCGCCGCACGGCCGGGTCGCGGCGCGGCTCCCCCCGGACGGGACTACGTGAGCATGCAGCTGGGGGGCTCCTGCTCGGACTGCGCCGACAGCCCCTCGCCCTCCTCGCCCGCCCCGCTGCTCGGCTACGCCGACGTGCGGGCGGGCCGCTCCGCCGCCGAGAAGCCGCCGCCGGCCGCCGCAGCTTCCCCCGAGCTGCCGCGGCCCCCGGCCGAGCTGGCGGCGGCCCCGCCGCGCTCCTCCTCCCTGCTCGGGGGCCCCGGCGCGGGCAGCGCCTTCACCCGCGTCAGCCTCAGCCCCGGCCGTAACCAGAGCGCCAAGGTGATCCGCGCCGACCCGCAGGGCGGCCGCCGGCGGCACAGCTCCGAGACTTTCTCGTCCACGCCGAGCGCCGCCCGCGGGGCGGCAGGCGGCGGCGGCGGCGGCGGCGGCCCCGGGGCGCCCTTCCCCTGCGGCGGCGCGGGGGGCGCCGAGGAGGTGAAGCGCCACAGCTCGGCCTCCTTCGAGAACGTGTGGCTGCGGCCCGCCGCCGGGGAGCCGCCCTCCGCGAGTCGGGGGCCGGGGGCCGCGCTGGAGAACGGACTCAACTACATCGACCTGGACTTGGTGAAGGATTGCAGCCACCGCCGCCACCACCTGCACCCCCCCGCGGAGGGCGCCTCCGGCCCGGGGGGGAAACCGCCGCAGCCCCGCTCCCCGCGCGGGAGCAGCCACTCCAGCGACGACCTGAGCGCGTACGCCAGCATCAGCTTCCAGAAGCGGGAGGAGCCCTAG

>Stu_vu1 [Common starling (Sturnus vulgaris) Irs1]

ATGGCCAGCCCCACGGATAATAACGAGGGCTTCTTCTCAGATGTCAGAAAGGTGGGTTACTTGCGCAAACCCAAGAGCATGCATAAACGATTTTTCGTGCTGAGGGCAGCCAGTGAGTCTGGACCCGCCCGACTGGAGTATTACGAGAATGAGAAGAAATGGAGACACAAGTCAGGGGCCCCCAAACGCTCCATCCCACTAGAAAGCTGCTTCAACATCAACAAACGGGCTGACTCCAAGAACAAGCACCTGGTGGCCCTGTACACCAAGGACGAGCACTTTGCCATTGCAGCTGACAGTGAACCTGAGCAGGAGAGCTGGTACCAAGCGCTGCTGCAGTTGCACAACAGGGCCAAGGGCCACCACCACCTTCATCACCATCACCACCACCACCACAGTGATGTCACTTTTGGAGGCAGCAGCACAGGACTGGGGGAAGCAGGTGAGGACAGTTATGGTGAGGTAGCCCCTGGTCCAGCTTTTAAGGAAGTTTGGCAAGTAATTCTGAAGCCTAAGGGCCTAGGCCAGACAAAGAACCTGATTGGCATCTACCGCCTGTGCCTGACTAACAAGACCATCAGCTTTGTAAAGCTGAATTCAGATGCGGCTGCTGTGGTGCTGCAGCTGCTCAATATCCGCCGCTGTGGTCACTCTGAGAACTTCTTCTTCATTGAGGTGGGACGCTCAGCTGTCACTGGACCCGGTGAGTTCTGGATGCAAGTGGATGACTCAGTGGTGGCACAGAACATGCATGAAACTATCCTGGAGGCCATGCGAGCCATGAGTGAGGAATTCCGGCCCCGAAGCAAGAGCCAGTCCTCCTCAAACTGTTCCAACCCGATCTCTGTGCCCCTTCGCAGGCACCACGTCAACAACCCTCCCCCGAGCCAAGTGGGGCTCAGTCGGCGGTCCAGGACAGAGAGCGTCACGGCCACCTCTCCTGCCGGCGGTGGAGGTACAGGTGGCAAACCCAGCTCTTTCCGGGTTCGAGCATCGAGTGACGGGGAAGGCACGATGTCAAGACCTGCCTCTGTGGATGGTAGCCCAGTTAGTCCCAGTGCTAACCGGACTCATTCACACAGACACCGCGGCAACTCCAGGCTCCATCCTCCACTCAACCACAGCCGGTCCATCCCAATGCCTTCCTCACGCTGCTCTCCTTCAGCCACCAGTCCAGTCAGCCTGTCATCCAGCAGCACTAGTGGCCATGGCTCCACCTCAGACTGCCTGTTTCCACGAAGGTCTAGTGCTTCAGTTTCTGGCTCCCCTAGTGACGGTGGATTTATTTCTTCTGATGAGTATGGTTCAAGCCCGTGTGACTTTCGCAGCTCTTTTCGCAGTGTAACCCCTGATTCATTGGGACACACCCCCCCTGCCCGGGGTGATGAAGACCTCAACTATATCTGCATGGGAGGGAAGACCACCTCTTCTTGCTGCAGCCTGGCAGCACCCAATGGCCATTTCATCCCACGCACCTGCCACCCTCAGCAGCAGCCCCGTTATCCTAGTACACCCTGCTGTCCTCGAGGTGGTAGTGAGGATGTTGCTGACTTGGAGAAGGCATTCAGAAAGAGGACTCACTCTGCAGGCACTTCACCCACCATCTCCCACCAGAAGACACCCTCACAGTCTTCGGTGGCCTCTATTGAGGAGTACACGGAGATGCTGCCTTCTTACCCCTGTGGCAGCAGCAGGCTGCCCTCCTACCGCCACTCAGCCTTTGTGCCCACTCACTCCTACCCAGAGGAGTGCCTGGAGATGCACCACATGGACAGTGGCCATCATCGGACCAACTCCGCCCCACACACGGATGATGGCTACATGCCTATGTCACCTGGTGTAGCCCCTGTGCCCAGTGGTGGGGGGCCTCCCAAGGGTGGTGACTATATGCCCATGAGTCCTAAGAGTGTGTCGGCCCCACAGCAGATCATCAACCCTGGCAGGGGTGGCCGCCACCCTCCAGCCACAGTGGACTCTAATGGCTACATGATGATGTCCCCCAGTGGCAGCTACTCCCCCGACAGCAGCTCTGCGGGCTACGGCAAGATCTGGACAAATGGTGCCGGCCACCACCCGAAACTCTCAGTGGAGAGCAACGAAGGGAAGCTCCCCTGCGGCGGCAGCGACTATATCAACATGTCCCCAGCCAGCGGCTCCACCACCAGCACTCCGCCCGACTGCTACTTCGGGGCTGCGGGGCAGCCGGGGGGCGAGGAGGCGGCCACCGTAGCGCACCACAAACCCATCTACTCCTACTTCTCGCTGCCACGCTCCTTCAAGCACGTGCACCGGCGGGGCGGCAGCAGGCCGGCGGGGGCAGCCGAGGAGTGCAGCCCTCAGCCCCGCATCGCGCTCGGCTCCAGCCGCCTCCTTTACGCGGCCGAGGACTCGTCCTCCTCCACCAGCAGCGACAGCCTGGGCGGCGGCGGCGGCCTCGAGGGCGGCCCCCCTCCTCAAGCGCAACCCCCGCGCAAGGTGGACACGGCCGTGCAGACCAAGGGCCGCCTGGCGCGCCCCACGCGGCTGTCGCTGGGTGGCCCCAAGGCTAGCACCCTGCCGCGGGCCCGGGAGCAGCCCCCGGTGCTGCTGCCCCCGGAGCCCAAGAGCCCCGGCGAGTACGTGAACATCGAGTTCATCGGCGGCGACAAGCCGCCCTTCCCCTCGGCTGCCCTGAGCATGGCCCTGCCGCCGCCGGGCAGCGAGGCCGCCGAGGAGTACATGAACATGGAGCCGGGCCCGCCGCGGGCCCCCTGCCCGCCCGGCTTCCCCACCGCGCGGGCGGCCCGGCTCGGCCGGGACTACGTGGCGATGCAGCGGGGGGGCGCCGCGGCCGGCGGGGGCTCCTGCTCGGACTACGCGGACAGCCCATCCCCCGGCTCGCCCGCCCGCCTGCTCAGCTACGCCGAGGTGCGGGGGGGCCGCGCCGGCCCCGAAAAGCCCCCGCCGGCGGCCGCCACCGCTGCGTCCCCGGAGCTGCCGCGGCCGCCGGCCGAGCTTTCGGCGGCGCCGCCGCGCTCCTCCTCCCTGCTGGGGGGCCTAGGCGCGGGCAGCGCCTTCACCCGCGTCAGCCTCAGCCCCGGCCGCAACCAGAGCGCCAAGGTGATCCGCGCCGACCCGCAGGGCGGCCGGCGGCGGCACAGCTCCGAGACCTTCTCCTCCACGCCGAGCGCGGCCCGCGGAGGGGCGGGCGGGCTCGGGGCACCCTTCCCGTGCGGTGGCGCGGGGGGCGCCGAGGAGGTGAAGCGGCACAGCTCTGCCTCCTTCGAAAACGTGTGGCTGCGGCCCGGCGCGGGGGAGGCGGCCGCCCGCCGGGACCCGGGGGCCGCGCCCGCCCTGGAGAACGGGCTCAACTACATCGACCTGGACTTGGTGAGGGACTGCGGCCACCGCCGCCACCACAACCCGCACGCCTCCGCTGAGGCGAAGCAGCCGCTGCCGCCGAAGCCCCCGGGCCAGCAGCGCGGGAGTGGCCACTGTGGCGAGGATCTGAGCGCGTACGCCAGCATCAGCTTCCAGAAGCGGGAGGAGATGTAG

>Pse_hu1 [Tibetan ground-tit (Pseudopodoces humilis) Irs1]

ATGGCCAGCCCCACGGATAATAACGAGGGCTTCTTCTCAGATGTCAGAAAGGTGGGTTACTTGCGCAAACCCAAGAGCATGCATAAACGATTTTTTGTGCTGAGGGCAGCCAGTGAGTCTGGACCCGCCCGGCTGGAGTATTACGAGAATGAGAAGAAATGGAGACACAAGTCAGGGGCACCCAAGCGCTCCATCCCACTAGAAAGCTGCTTCAACATCAACAAACGGGCTGACTCCAAGAACAAGCACCTGGTGGCCCTCTACACCAAGGACGAGCACTTTGCCATTGCAGCTGACAGTGAACCTGAGCAGGAGAGTTGGTACCAAGCACTGCTGCAGTTGCACAACAGGGCCAAGGGCCACCACCACCTCCATCACCATCACCATCACCACCACAGTGATGTCACTTTTGGAGGCAGCAGCACAGGACTGGGGGAAGCAGGTGAAGACAGCTATGGTGAGGTAGCCCCTGGTCCAGCTTTTAAGGAAGTTTGGCAAGTAATTCTGAAGCCGAAGGGCCTAGGGCAGACAAAGAACCTGATTGGCATCTACCGCCTGTGCCTGACTAACAAGACCATCAGCTTTGTAAAGCTGAATTCAGATGCGGCTGCTGTGGTGCTGCAGCTGCTCAATATCCGCCGCTGTGGTCACTCTGAGAACTTCTTCTTCATTGAGGTGGGACGCTCAGCTGTCACTGGACCTGGTGAGTTCTGGATGCAAGTGGATGACTCAGTGGTGGCACAGAACATGCATGAAACTATCCTGGAGGCCATGCGAGCTATGAGTGAGGAATTCCGGCCCCGCAGCAAGAGCCAGTCTTCCTCAAACTGTTCCAACCCGATCTCTGTGCCCCTTCGCAGGCACCATGTCAACAACCCTCCACCGAGCCAAGTGGGGCTCAGTCGGCGGTCCAGGACTGAGAGTGTCACGGCCACTTCTCCTGCCGGTGGTGGAGGTACAGGTGGCAAACCCAGCTCTTTCCGGGTTCGAGCATCGAGTGATGGGGAAGGCACAATGTCAAGACCTGCCTCTGTGGATGGTAGCCCCGTTAGTCCCAGTGCTAACCGGACTCATTCACACAGACACCGTGGCAACTCCAGGCTCCATCCTCCACTCAACCACAGCCGGTCCATCCCAATGCCTTCCTCGCGCTGCTCTCCTTCAGCCACCAGTCCGGTCAGCCTGTCATCCAGCAGCACTAGTGGCCACGGCTCCACCTCAGACTGCCTGTTTCCACGAAGGTCTAGTGCTTCAGTTTCTGGCTCCCCTAGTGATGGTGGATTTATTTCTTCTGATGAGTATGGTTCAAGCCCGTGTGACTTTCGCAGCTCTTTTCGCAGTGTAACCCCTGATTCATTGGGACACACCCCTCCTGCCCGGGGTGATGAAGACCTCAACTACATCTGCATGGGAGGGAAGGCCACGTCTTCATGCTGCAGCCTGGCAGCACCCAATGGCCATTTCATCCCACGCACCTGCCACCCTCAGCAGCAGCCCCGCTATCCTAGTACACCCTGCTGTCCTCGAGGTGGTAGTGAGGAGGTTGCCGACTTGGAGAAGGCATTCAGAAAGCGGACTCACTCTGCAGGCACTTCACCCACTATCTCCCATCAGAAGACTCCCTCCCAGTCTTCAGTGGCCTCCATTGAGGAGTACACGGAGATGCTGCCTTCTTACCCCTGTGGCAGCAGCAGGCTGCCCTCCTACCGCCACTCAGCCTTTGTGCCCACTCACTCCTACCCAGAGGAGTGCCTGGAGATCCACCACATGGACAATGGCCGTCATCGGACCAACTCCACCCCGCACACGGATGATGGCTACATGCCCATGTCGCCTGGTGTAGCCCCTGTGCCCAGTGGTGGGGGGCCTCCCAAGGGTGGTGACTATATGCCCATGAGTCCTAAGAGTGTGTCGGCCCCACAGCAGATCATCAACCCTGGCAGGGGTGGCCGCCACCCTCCAGCCACAGTGGACTCTAATGGCTACATGATGATGTCCCCCAGTGGCAGCTACTCCCCCGACAGCAGCTCTGCGGGCTATGGCAAGATCTGGACGAATGGTGCCGGCCACCACCCAAAACTCTCAGTGGAGAGCAACGAAGGGAAGCTCCCTTGCGGCGGCAGCGACTATATCAACATGTCCCCAGCCAGCGGCTCCACCACCAGCACTCCGCCCGACTGCTACTTCGGGGCTGCGGGGCAGCCGGGGGTCGAGGAGGCGGCCACCGTAGCGCACCACAAACCCATCTACTCCTACTTCTCGCTGCCACGCTCCTTCAAGCACGTGCACCGGCGGGGCGGCAGCAGGCCGGCGGGGGCAGCCGAGGAGGGCAGTCCTCAGCCCCGCATCGCGCTCGGTTCCAGCCGCCTCCTTTACGCGGCCGAGGACTCGTCCTCCTCCACCAGCAGCGACAGCCTGGGCGGCGGCGGCGGCCCCGAGGGCGGCCCCCCGCCTCAAGCGCAGCCCCCGCGCAAGGTGGACACGGCCGTGCAGACCAAGGGCCGCCTGGCGCGCCCCACGCGGCTGTCGCTGGGTGGCCCCAAGGCCAGCACCCTGCCGCGGGCCCGGGAGCAGCCCCCGGTGCTTCTGCCCTCCGAGCCCAAGAGCCCCGGCGAGTACGTGAACATCGAGTTCATCGCCGGCGAGAAGCCGCCCTTCCCCTCGGCTGCTCTGAGCATGGCCCTGCCGCCGCCGGGCAGCGAGGCCGCCGAGGAGTACATGAACATGGAGCCGGGCCCGCCGCGGGCCCCCTGCCCGCCCGGCTTCGCCACCGCGCGGGCGGCCCGGTTCGGCCGGGACTACGTGGCGATGCAGCGGGGGGGCGCCGCGGCCGCTGGGGGCTCCTGCTCGGACTACGCGGACAGCCCGTCCCCCGGCTCGCCCGCCCGCCTGCTCAGCTACGCCGAGGTGCGGGGGGGCCGCGCCGGCCCCGAAAAGCCCCCGCCGGCTGCCGCCACCGCTGCGTCTCCGGAGCTGCCGCGGCCGCCGGCCGAGCTGTCGGCGGCGCCGCCGCGCTCCTCCTCCCTGCTCGGGGGCCTGGGCGCGGGCAGCGCCTTCACCCGCGTCAGCCTCAGCCCCGGCCGCAACCAGAGCGCCAAGGTGATCCGCGCCGACCCGCAGGGCGGCCGGCGGCGACACAGCTCTGAGACCTTCTCCTCCACGCCGAGCGCGGCCCGCGGAGCGGCGGGAGGGCTCGGGGCACCCTTCCCGAGCGGCAGCGCGGGGGGCGCCGAGGAGGTGAAGCGGCACAGCTCGGCCTCCTTCGAGAACGTGTGGCTGCGGCCCGGAGCGGGGGAGGCGGCCGCCCGCCGGGACCCGGGGGCCGCGCCCGCCCTGGAGAACGGGCTCAACTACATCGACCTGGACTTGGTGAGGGATTGCGGCCACCGCCGCCACCACCACCCGCACACCCCCGCCGAGGCGAAGCAGCCGCTGCCGCCGAAGCCCCCGGGCCAGCAGCGCGGGAGTGGCCACTGTGGCGAGGATCTGAGCGCGTACGCCAGCATCAGCTTCCAGAAGCGGGAGGAGATGTAG

>Cal_pu1 [Ruff (Calidris pugnax) Irs1]

ATGGCTAGCCCCACAGATAATAACGAGGGCTTCTTCTCAGATGTCAGAAAGGTGGGTTACTTGCGCAAACCCAAGAGCATGCATAAACGCTTTTTCGTGCTGAGGGCAGCCAGCGAGTCTGGACCCGCCCGGCTGGAGTATTATGAGAATGAGAAGAAATGGAGACACAAGTCAGGGGCCCCCAAGCGCTCCATCCCACTAGAAAGCTGCTTCAACATCAACAAACGGGCTGACTCCAAGAACAAGCACCTGGTGGCCCTCTACACCAAGGACGAGCACTTTGCCATTGCAGCTGACAGCGAGCCTGAACAGGAGAGCTGGTACCAAGCGCTGCTGCAGTTGCACAACAGGGCCAAGGGCCACCACCACCTCCATCACCATCACCACCACCACCACAGCGACGTCACCTTTGGAGGCAGCAATGCGGGACTAGGGGAAGCAGGTGAGGACAGCTATGGTGAGGTAGCCCCTGGCCCGGCTTTTAAGGAAGTTTGGCAAGTAATTCTGAAGCCTAAGGGCCTAGGCCAGACAAAGAACCTGATTGGCATCTACCGCCTGTGCCTGACTAACAAGACCATCAGCTTTGTGAAGCTGAATTCAGATGCGGCTGCTGTGGTGCTGCAGCTGCTCAATATCCGTCGCTGTGGTCACTCTGAGAACTTCTTCTTCATTGAGGTGGGGCGCTCGGCGGTCACTGGGCCCGGTGAGTTCTGGATGCAGGTGGATGACTCGGTGGTGGCGCAGAACATGCACGAAACCATCTTGGAGGCCATGCGAGCAATGAGCGAGGAATTCCGACCCCGCAGCAAGAGCCAGTCCTCCTCAAACTGTTCCAACCCCATCTCTGTGCCCCTTCGCAGCAGGCATCACGTCAACAACCCTCCACCCAGCCAAGTGGGCCTCAGCCGCCGGTCCAGGACTGAGAGTGTCACGGCCACCTCTCCTGCTGGTGGTGGGGGTGGAGGTACGGGTGGCAAACCCAGCTCTTTCCGGGTTCGAGCGTCGAGTGATGGGGAAGGCACGATGTCGAGGCCTGCCTCTGTGGATGGTAGCCCAGTTAGTCCCAGTGCCAACCGGACCCATTCGCATAGGCACCGTGGCAACTCCAGGCTCCATCCTCCACTCAACCATAGCCGGTCCATCCCAATGCCTTCCTCGCGCTGCTCTCCTTCAGCCACCAGTCCAGTCAGCCTGTCATCCAGCAGTACCAGTGGCCATGGCTCCACCTCAGACTGCCTGTTTCCTCGAAGGTCTAGTGCTTCGGTGTCTGGCTCCCCTAGCGATGGTGGATTTATTTCTTCTGATGAGTATGGTTCCAGCCCGTGTGACTTCCGCAGCTCCTTTCGCAGTGTGACCCCAGATTCATTGGGACACACCCCACCAGCTCGGGGAGATGAAGAGCTCAACTACATCTGCATGGGGGGGAAGGCCACCTCCTCTTGCTGCAGCCTGGCAGCCCCCAATGGCCACTTCATCCCACGCACCTGCCACCCACAGCAGCAGCCCCGCTACCCTAGCACACCGTGCTGTCCTCGAGGTGGTAGCGAGGAGGTTGCCGACTTGGAGAAGGCATTCAGGAAGCGGACTCACTCTGCAGGCACATCGCCCACCATCTCCCACCAGAAGACACCCTCACAGTCTTCGGTGGCCTCCATTGAGGAGTACACAGAGATGCTGCCTTCTTACCCCTGTGGTGGCAGCCGGCTGCCCTCCTACCGGCACTCAGCCTTTGTGCCTACTCACTCCTACCCAGAGGAGTGTCTGGAGATGCACCACCTGGATGGCGGCCATCATCGGACCAACTCCGCCCCGCACACGGATGATGGCTACATGCCCATGTCACCTGGTGTAGCCCCTGTGCCCAGCGGTGGGGGGCCCCCCAAGGGTGGTGACTACATGCCCATGAGTCCTAAGAGCGTGTCGGCCCCACAGCAGATCATCAACCCTGGCAGGGGGGGCCGCCACCCTCCAGCCACGGTGGACTCCAACGGGTACATGATGATGTCCCCCAGTGGCAGCTACTCCCCCGACAGCGGTTCTGCGGGCTATGGCAAGATCTGGACAAATGGCGCAGGCCACCACCCGAAACTCTCGGTGGAGAGCAACGAAGGGAAGCTCCCCTGTGGTGGCAGTGACTACATCAACATGTCCCCAGCCAGCGGCTCCACCACCAGCACCCCTCCTGACTGCTACTTTGGAGGCGCGGGGCAGCCGGGCGTTGAGGAGGCCGCCACCGCGGCCCACCACAAACCCATCTACTCCTACTTCTCGCTGCCGCGCTCCTTCAAGCACGTGCACCGGCGGGGTGGCGGCGGGGCGGCAGGCGAGGAGGGCAGCCCCCAGCCCCGCATGGCTCTGGGCTCCAGCCGCCTCCTCTACGCCGCCGAGGACTCGTCCTCCTCCACCAGCAGCGACAGCCTGGGCGGCGGCGGCGGCACCGAGGGGGGCCCCCCGCCGCAGGCGCAGCCCCCGCGCAAGGTGGACACGGCCGTGCAGACCAAGGGCCGCCTGGCGCGACCCACCCGGCTGTCGCTGGGCGGCCCCAAGGCCAGCACCCTGCCGCGGGCCCGGGAGCAGCCCCCGCTCCTCCTGCCCCCGGAGCCCAAGAGCCCCGGCGAGTACGTGAACATCGAGTTCATCGCCGGCGAGAAGCCGGCCTTCCCTTCGTCCGCCCTGGGGCTGGGCCTGCCGCCGCCGCCGGGCAACGAGGGCGCCGAGGAGTACATGAACATGGAGCTGGGGCCGCCGCGGGCCCCCTGCCCCTCCGGCTTCGCCGCCGCCCGGGCGGTCTCCGCAGCCCGCTCCGGCCGAGGCGCGGCCCCCGGCGGCCGGGACTACGTGACGATGCAGCTGGGGGGCGCCGCCGGCGGGGCCTCCTGCTCGGACTGCGCCGACAGCCCTTCCCCCTGCTCGCCTGCCCTCCTGCTCAGCTACGCCGACGTGCGGGCGGGTCGCTCCGCCGCCGAGAAGCCCCCTCCGGCGGCGACGGCTTCCCCGGAGCTGTCGCGGCCGCCGGCCGAGCTGTCGGCGGCGCCGCCGCGCTCCTCCTCCCTGCTGGGGGGTCCCGGCGCGGGCAGCGCCTTCACCCGCGTCAGCCTCAGCCCCGGCCGCAACCAGAGCGCCAAGGTGATCCGCGCCGACCCGCAGGGCGGCCGCCGGCGGCACAGCTCCGAGACCTTCTCCTCCACGCCCAGCGCCGCCCGCGGAGCGGCGGGCAGCGGCGGCGGGCCCGGGGCGGCCTTCCCCTGCGGCGGGGCGGGGGGCGCCGAGGAGGTGAAGCGGCACAGCTCGGCCTCCTTCGAGAACGTCTGGCTGCGGCCCGCCGCGGGGGAGCCCCGCCGGGAGCCGGGGGCCGCGCCCGCCCTGGAGAACGGGCTCAACTACATCGACCTGGACTTGGTGAAGGACTTCAGTCACCGCCGCCGCCACCACCACCTCCACCCCCCCGCCGAGGGCGCCGCTCTGCCGGGGGGCAAGCAGCCGCCGCCGAAGCCCCCGGGCCAGCCCCGCGGGAGCGGCCACTCCAGCGACGACATGAGCGCGTACGCCAGCATCAGCTTCCAGAAGCGGGAGGAGATGTAG

>Xen_tr1 [Western clawed frog (Xenopus tropicalis) Irs1]

ATGGCTAGCCCCACAGATCCACAGGCTCAGGAGAACTTTTCTGATGTCAGAAAGGTGGGCTATCTAAGGAAACCTAAGAGCATGCACAAGAGGTTTTTTGTGCTCAGGTCTGCCAGTGAGTCAGGTTTGGCCCGTTTGGAGTATTATGAAAATGAGAAAAAGTGGAGGCATAAGTCTGGGGCCCCAAAGAGGTCAATCCCTCTTGAGAGCTGCTTTAATATTAACAAGAGGGCTGATTCAAAAAACAAACATTTGGTTGCTTTGTACACAAAGGAGGAATGCTTTGCAATTGCTGCTGAATGTGAACAAGAACAGGATGGTTGGTATCAGGCTCTAGTGGATCTTCACAACCGTGGTAAGACCCACCACCAACACCATAACCATGACGGTGCAACCAATGGTGTACATGATGGTTTAAATGGAGATGACGTTTATGGGGAGGTTACTCCTCCAGGACTGGCCTTCAAAGAGGTATGGCAGGTGATCATGAAGCCCAAGGGTTTAGGGCAACTTAAGAATTTAGTTGGTATTTACCGCCTGTGCCTCACCAACCGAACAATAAGTTTGGTAAAACTGAACTCTGATGCAGCCGCTGTGGTATTGCAACTTATGAATATAAGGAGATGTGGGCATTCAGAAAATTTTTTCTTTATAGAAGTGGGTCGTTCAGCAGTCACCGGAGCTGGTGAATTCTGGATGCAAGTGGATGATTCAGTAGTTGCTCAAAACATGCATGAAACCATACTAGAAGCTATGAAAGCTCTGAGCGATGAATTTCGGCCTCGGAGTAAGAGTCAGTCTTCATCTAACTGCTCTAATCCTATATCTGTACCTCTTAGGAGGCATCACCTTAATCATCCACCACCCAGCCAGGTAGGACTAAATAGAAGAGCTCGTACAGAGAGTGTTACTGCCACATCACCTGCAGGGGGTGTGGCAAAGCATGGATCATCCTCCTCTTTCAGAGTCCGGGCATCTAGTGATGGAGAAGGAACAATGTCAAGGCCAGCCTCCATGGAAGGGAGCCCAGTAAGTCCCAGTGCTAGCAGAGCTCAGTCGCACCGGCATCGGGGAAGTTCTCGACTTCACCCGCCTCTTAACCATAGTCGGTCTATACCAATGCCAGCAACTCGCTGCTCTCCATCGGCTACCAGCCCTGTCAGCTTGTCCTCCAGTAGTACCAGTGGACATGGTTCCACTTCTGATTGCATGTGCCCTCGAAGATCCAGTGCCTCTGTTTCTGGTTCACCCAGTGATGGTGGTTTTATTTCTTCAGATGAATATGGGTCTAGCCCTTGTGACTTTAGAAGTTCTTTTCGCAGTGTAACCCCAGATTCTTTGGGCCACACTCCACCAGCTAGGGAAGAAGAACTAAATAACTATATTTGCATGGGCAAATCCGGTAGTCATCTCCAGAGAAGTCAGCAGCAGAGGTACCAGCTAAGTCGAGGGGAAGAGCACCCTGACTTTGATAAGGTTTTTAGAAAGAGGACTCACTCCTCAGGAACATCACCTCCTACAGTTTCACACCAGAAGACACCATCACAGTCCTCTATTGAGGAATATACAGAGATGATGCCTGCTCATCCTGTTCGTTTAACATCATTTAGACATTCTGCATTTGTACCCACCTACTCCTATCCAGAAGAGTGCCTGGACCTCCATTTAGAGGGTAGCAGAGCTAACCACACAGATGACGGATACATGCCTATGTCACCTGGTGTTGCTCCTGTGCCTACAAAATCAAATGACTATATGCCTATGAGCCCTAAGAGTGTGTCGGCCCCTCAACAGATCATTAACCCAAGACGGCACTCTGCTGTAGATTCAAATGGTTATATGATGATGTCACCCAGTGGTAGCTGTTCTCCGGATAGCACCAATTACAGCAAGATATGGACCAATGGTACCAATCCAAAGTTGTCCATAGATAGCATTGAGGGCAAACTGCCTTGCAGTGACTACATTAATATGTCTCCTGCTAGTGGCTCTACTACAAGCACGCCTCCAGACTCTTATCTGAATTCTATTGAAGAGTCGACCAAGCCTGTATATTCCTACTTTTCCCTGCCCAGGTCCTTCAAACATGTTCACAGGAAAAGTGAAGATGGCAATTTGCGCATCACTGCAAATTCTGGACATAATCTATACACTGAGGACTCTTCCTCCTCTTCCACAAGCAGTGATAGTCTAGGGGGGCAGGACCCTCAGCAGCCTAGAAAAGGGGAGACCTGCATTCAAGGGAAGAGATTGACCAGACCCACCAGGCTTTCATTGGAAAACAGCAGTAAGGCCAGCACACTGCCAAGGGCGAGAGAACCTGCTTTGCCCCCAGAGCCAAAAAGTCCTGGGGAGTATGTTAATATAGAGTTTAACGACAAGGTATTTTCAGGGGGCTTGATGCCTTCCATGTGTCCCCTTCCTTTTGTGCAGAGCAGAGTTGTGCCTCAAAGAGAGAACTTGTCTGAATACATGAACATGGATCTTGGAGTTTGGAGAGCTAAAACTTCCTATGCTTCCACCTCATCTTCATCATATGAACCACCATACAAGCCGGTCAGCTCAGTGTGCCCTACTGAAACTTGCAGTAGTAGTCGACCACCTATTAGAGGAAAGACAAACTCACGAGACTATATGAGCATGCAACTTAGTGCTCTGTGTTCAGATTACAGCCAAGTTCCACCTACCAGAATAACTGCCAAACCCATTACTCTTTCTTCTAATAAAAGTAATTATGCAGAGATGTCAAGTGGCGGAGTTTCTGATAATATTCCTGCTATTCCCCAAACTTCTAACTCAAGCCTATCTGAAGCATCTCGCTCTTCCCTTTTGGGCCAGGGCTCTGGCCCTAGCGCCTTTACTCGGGTCAGTCTAAGCCCAAACCGCAATCAGAGTGCCAAAGTCATCAGGGCCGGGGACCCACAGGGTAGGAGGAGGCACAGCTCAGAGACATTCTCCTCTACTCCTACTACAGCCAGAGTTACTTCTGGCCCAGTTTCTGGGGAGGATGTCAAGAGACATAGTTCTGCCTCCTTTGAAAATGTGTGGCTAAAACCAGGAGAGATTGCCAGGAGAGATTCCCTTCAGCCTTCTGATCACACACATAATGGCCTGAATTATATTGACTTAGATCTAGCTAAGGACTTGAGTGGCCTGGATCATTGCAACTCCCACCAATCTGGAGTAAGCCACCCATCAGATGACCTGAGCACATACGCCAGCATCACTTTCCACAAGCTAGAGGAGCACCGGAATCAGGCAGAAACAGAAGAATAA

>Lat_ch1 [Coelacanth (Latimeria chalumnae) Irs1]

ATGGCCAGCCTCACTGAGAATGAGAGCTTTTCGGATGTGAGGAAGGTAGGTTACTTAAGGAAACCCAAAAGCATGCACAAGAGGTTTTTCGTCCTGCGGGCTGCAACTGAAGCGGGTCCCGCCAGGCTGGAATACTATGAGAACGAGAAGAAATGGAAGCATAAGTCGGGGGCACCCAAGCGATCCGTCCCATTGGAGACTTGCTTCAACATCAACAAGAGGTCCGACTCCAAGAACAAGTATCTGGTGGCACTGTACACCAAGGACGAGTACTTTGCCATCGCAGCCGACAGCGATCAGGAGCAGGAAACTTGGTACCAAGCCCTGGTGGATTTGCACAACAGAGGTAAGGTCCACGATGCGGCGGGGGGTAGTGGACCCGCCGACGGGGAAGACAGCTATGGAGAGCTGACCCCTGGCCCCGCGTTCAAAGACGTCTGGCAGGTGATCCTGAAACCAAAGGGCTTGGGGCAAACTAAAAACCTGATCGGAATTTACAGGTTGTGCCTGACCAACAAAACCATCAGCTTTGTGAAGTTGAATTCAGACGCGGCCGCTGTGGTTTTGCAGCTGATGAATATTCGGAGATGCGGACACTCGGAGAATTTCTTCTTCATTGAAGTGGGCAGGTCTGCAGTGACTGGACCCGGGGAGTTTTGGATGCAGGTAGATGATTCTGTTGTAGCACAGAACATGCACGAGACTATCCTGGAAGCCATGAAGGCGATGAGTGAGGAGTTTCGTCCAAGGAGCAAAAGTCAATCGTCTTCCAATTGTTCCAACCCCATCTCGGTGCCTCTCAGAAGGCACCATATTAACAACCCTCCTCCTAGCCAAGTCGGGCTCAGCAGGAGATCAAGAACTGAAAGTGTCCCTGCTACATCACCTGCTGGGGCAAGTAAGCAGAGTTCGTTTAGAGTAAGAGCTTCAAGCGATGGAGAGGGGACCATGTCCAGGCCAGCATCCGTAGATGGTAGCCCAGTGAGCCCTAGTGCTACAAGGCCACATTCTCACAGACATAGGGGCAGCTCAAGGCTGCATCCTCCTTTGAATCACAGCAGGTCCATCCCTATGTCATCTTCACGCTGCTCACCTTCTGCCACAAGTCCTGTTAGCTTATCCTCCAGTAGTACTAGTGGCCACGGATCTACTTCAGATTGCCTCTTCCCTAGGCGTTCAAGTGCATCAGTGTCTGGCTCTCCCAGTGATGGTGGTTTCATTTCTTCTGATGAGTATGGGTCAAGTCCTTGTGACTTTAGGAATTCTTTCAGAAGCGTAACCCCTGATTCTTTGGGGCACACTCCTCCTGCACGGGAAGAAGAGCTTAACAATTACATCTGTATGGGAAAGCCAAATCTTGTGCCAAATGGACATTACAAGAGGAGCCATCAGAGATACATGCCTACTAGAGGAGAAGAAGCAGATTTAGAAAAAAGTTTTAGAAAAAGGACCCATTCTGCTGGTACTTCACCTACTATATCTCATCAAAAGACCCCCTCCCAGTCTTCCACAGCATCTTTGGAAGAGTATACTGAAATGATGCCTTCATACCCTTGCAGCCGCTTGCCATCATACAGGCACTCAGCTTTTGTTCCTACTCATTCATATCCTGAGGAATGTTTGGATCTTCATATAGAAGGCAACCGAGCTAATCCTAGGGATGATGGCTATATGCCCATGTCACCAGGAGTAGCTCCTATACCTAACAAAAATGGTGATTATGTGCCCATGAGTCCAAAAAGTGTATCTGCTCCCCAACAGATCATTAATCCAAGGCGGCATTCCCAAATGGACTCAAATGGTTATATGATGATGTCTCCTAGTGGCAGCTGCTCTCCAGATAGTACAAACTATGGAAAAATATGGACCAATGGTGCAAATCCTAAACTTTCAGTTGATAGCAATGATGGGAAACTGCCTTGTGGTGACTATATCAATATGTCACCTGTAAGTGGATCAACAACCAGTACGCCACCCGATTGCTGTTTTAACCCAGTAGATGAACCTCCTAAACCCATTTATTCCTACTTCTCATTACCTCGATCTTTCAAACATATGCAGAGAAAAAATGATGAAACTCAGTTACGCATCTCTGTAAGTTCAGGTCGACTAATGTATGGAGAGGATTCTTCATCCTCCACTAGCAGTGACAGTTTGGGAGGCCAAGACAGTGAGCATCAAACAGTTAATATCAGAAAAGTTGAGTCCTGTGTCCAGACAAAAGGAAGACTTATACGGCCAACAAGGCTAACACTTGATAATGCTAAAGCAAGCACATTGCCCAGAACTCGTGAGCACCCGCTGCCACCTGAGCCCAAAAGCCCTGGGGAGTATGTCAACATTGAATTTGGTGACAAAGCATTTTTACCAAGTTCTGTGTCATTTTGCTCCTCTGCGTATACAGAAAATAATTCTACTCGACAAAGGGAAAATTTGTCAGAATACATGAATATGGATTTAGGGGCACGTGCAGGCAAGCCAAGTTTTGTATCAAAATCTTCAGTGGACATTACAAGCTCTGCATCTGGCTGTGCTACCAGTACATGCAGAACAAACAGAGGGCAGCCAAGCTGTGATTATGTGAGTATGCAGTTAGGCGCCACCTGCCCGAACTTTTCAGAATCACCAGTTATGATTAAGTATACAGAGATGGCAACTGGTGTTGCTAAAACATCTCCAAGATCCACTTCGCCAAAGCATGAAATACCTTCATTAAACAGCATTTCTGATGCAGCACTTCCAAGTTTGGTGGGGCAGAGTTCAGGAATGAGTGCTTTCACTAGAGTAAATCTAAGTCCTAATCGAAATCAAGGTGCCAAAGTAATTCGTGCTGATCCCCAAGGAAGAAGGCGGCATAGTTCGGAGACATTTTCTTCAACACCCAGTGCCAGTGGTGTGGCCTTATCTTATGTAGATGATGTTAAACGACATAGTTCTGCTTCTTTTGAAAATGTCTGGATTAAACAAGCAGATTCTACAGTTTCTGTTAAGAAGGAGCAGCTAAGTGATGTCAGTTCACTGAACAGTGCAGCAGCATTTGAAAACGGTCTGAATTACATTGATCTGGACTTAGCCAAGGATTTTAACAACCAGGAGCGGACCACCCTCCAGCCTAAATCCCAGAATCAGCCTTGTGGAAGTAACTCCAGTGATGATTTGAGTGCGTATGCAAGCATCAGCTTTCAGAAACCAGAAGATCTCCGAATCAATCCAGCAAAAAGAGAAGAGTGA

>Lep_oc1 [Spotted gar (Lepisosteus oculatus) Irs1]

ATGGCAAGCCCGACTACAGACCACGATTGTTTTTCGGACGTGAAGAAGGTGGGTTACTTGAGGAAACCCAAAAGCATGCATAAGAGATTTTTTGTTTTGCGGACTGCGAGCGTTTCAGGACCCGCGAGGCTGGAGTACTACGAAAATGAGAAAAAATGGAGACACAAGTCCGGAGCTCCCAAAAGGTCTATTGCGTTGGAAAGCTGCTTCAACATTAACAAGAGAGCGGACTCCAAAAATAAGTACCTAGTGGCTTTATATACTAAGGACGAGTATTTTGCAATTGCGGCCGATAGTGAGCCGGAGCAGGATTTGTGGTACCAAGCATTAGTAGAGCTACACAACAGAGGTAAGATTCACGATTCTGCCGGGGGCAGCGGATTTGGGGAAGACACCTACGGAGAGTCCAGGCCAGGACCAGCTTTTAAAGAAGTCTGGCAAGTTATTTTAAAACCAAAAGGCCTCGGGCAAACGAAGAATTTAATTGGGATTTACAGATTGTGCCTGACTAATAAAACTATCAGCTTTGTGAAACTGAACTCCGACGCAGCCGCCGTAGTTTTGCAGTTGATGAACATAAGGAGATGTGGACACTCTGAAAATTTCTTCTTCATCGAGGTTGGTAGATCTGCAGTTACGGGACCCGGGGAGTTCTGGATGCAGGTGGACGATTCGGTTGTAGCGCAGAATATGCATGAAACCATTTTGGAAGCGATGAAAGCAATGAGCGAGGAATTTCGCCCCAGGAGTAAAAGTCAATCTTCCTCCAACTGCTCCAACCCTATCTCTGTGCCCCTGAGAAGGCACCATCACAACAACCCTCCTCCTAGCCAAGTGGGACTCAGCAGGAGGTCGAGGACTGAAAGCATTACTGCAACTTCACCTGCAGGTCCAGGGAAACACGGTAACTCTTTCAGAGTGAGAGCGTCCAGCGACGGAGAGGGAACCATGTCCAGGCCTGCGTCGGTTGATGGGAGCCCTGTTAGTCCCAGTACCACTAGAACACATTCTCACAGGCACAGGGGCAGCTCCAGACTTCACCCGCCCTTAAACCACAGCAGATCTATTCCCATGCCCTCCTCGCGCTGTTCGCCATCAGCCACCAGCCCGGTCAGCTTGTCCTCGAGCAGCACAAGCGGCCACGGCTCCACCTCTGACTGCCTTTACCCCCGGCGCTCCAGCGCCTCCGTCTCGGGCTCTCCGAGCGACGGCGGCTTCATCTCCTCGGATGAGTACGGGTCCAGCCCCTGCGACTTCAGGAATTCTTTCAGGAGCGTCACCCCTGACTCTCTCGGGCACACCCCCCCTGCAAGGGAAGAGGAGCTGAATAATTACATCAGCATGGTGAAACCCAACCTCTTGCCCAATGGACATCATAATAGAAGTCACCAGCGATGCACCCCATCGAAGGTGGAAGAGGCCGAACTGGAAAAGGGATTCAGGAAGCGGACCCATTCTTCTGGAACTTCATCCCCAACTATATCCCATCAGAAGACTCCTTCCCAGTCATCCACAGCTTCCTTGGAAGAATACACGGAGATGATGCCAACGTACCAGTGTCGACTGTATAGGCATTCAGCCTTTGTGCCAACTCACTCTTACCCAGAGGAGTGCTTGGATCTCCATATTGAGGGCAGCAGAACGAACCACACAGATGATGGCTACATGCCCATGTCCCCGGGGGTAGCTCCTGTGCCTGCTAAAACTGATGACTACATGCCAATGAGCCCTAAAAGTGTGTCTGCGCCACAGCAAATAATTAACCCAAGGCAGCATCCAAGAGTGGACTCGAATGGCTACATGATGATGTCCCCCAGTGGCAGCTGCTCTCCAGACAACACAAACTATGGCAAAATATGGACCAATGGAGCAAATCCCAAGCTTTCAGTGGAGAGCAACGAAGGCAAAGTTTCATGCGGCGACTACATTAATATGTCCCCAGCAAGCGGCTCAACGACCAGCACTCCCCCGGACTGCTACTTCAACCCAGTCGAGGAGCCGCCCAAGCCCCTCTATTCCTACTTCTCTTTACCTCGCTCGTTTAAGCACGCGCACAGGAAAGCCGCCGAGAGCCAGCTGCGCATCTCCGTGAGCTCGGGCCGCCTCGTCTACGGCGAGGACTCGTCCTCGTCGACGAGCAGCGACAGCCTGGGGGGGCAGGAGAACGGGCAGCAGGCGGTCAAGCCCAAGAAAGCGGATGAGTACGCGCAGGCCAAGGGGAGGCTGGCGCGGCCGACCCGCCTCTCCCTGGACAACAACAACAAGGCCAGCACGCTCCCCCGCACGCGCGAGCACCCCTTCCCACCGGAGCCCAAGAGCCCGGGAGAGTACGTCAACATAGAGTTCAATGACAAGTCCTTCTCGGCAAGCCTGGCTTCCTTGTTCTCCCCCGTGTGTGCAGGGAGTTCCCCCGCGAGGCCGGAGCAGAATTCCTCGGAGTATATGAACATGGACCTTGGTGCTCATGGTAGCAAGCCAAGCTACCCATCGAAACCCACAGCGGACTCTACAGGCTGTGCTGCAGACTATGCAGTTACCGCTGCTGCTGCTGCAGCTGTCCCCGCTGCTGCTGCATGCAGAGCAAGCAGAGGACAGCAGAGCTGCGACTACGTGAGCATGCAGCTGAGCGCCCCCAGCGCAGGGTGCGCCGAAGCACCCATGTTGAGCTACACCGAAATGGGGACTGGAGCTGCTAAGACACCGACCCAGTCCCTCTCACCTCACCCAGAAATGCCCCCATTGAGTAGCGTCTCCAGTGCCGCGTGCTCCAGTTTAATGAGCCAGATGTCGGGGACAAGCGCTTTTACCAGAGTCAACTCGAGCCCCAACAGGATTCAAGGTGCCAAAGTGATCCGTGCTGATCCCCAGGGAAGGAGGCGGCACAGTTCAGAGACCTTTTCCTCCACACCTGCTGCTAGTGGTGGTGTGGCGTTACTCTGCGGGGATGATGTTAAAAGACACAGCTCGGCATCGTTTGAAAATGTCTGGCTTAAGCAGGGGGAATCGTATGTCTGTTTAAGAGAGGAGCAGCAGCCTGGTGTCAGTTCACTGAACAGTGCGACTCCATTTGAGAATGGTCTCAATTACATTGATCTGGACTTAGTCAAGGATTTTAACAACCAAGAGTGGACCTCCCTCCAGCCTAAATCCTCAAATCAGCCTTGTGGAAGTACCTCTGGTGATGATTTAAGTGCATATGCAAGCATCAGCTTTCAAAAACCAGATGAGATAAGGACTAATCCAGCAAAAAGAGAAGNNNNN

>Dan_re1 [Zebrafish (Danio rerio) Irs1

ATGGCAAGCCCGACTACGGAGCAAGGATGTTTTTCAGACGTGAAAAAGGTGGGTTATTTAAGGAAACCCAAAAGCATGCACAAAAGGTTTTTTGTACTGAGGGCGGCGAGCGCCTCCGGACCGTCCCGACTGGAGTACTATGAAAACGAGAAGAAGTGGAGACACAAGTCTGGAGCGCCGAAAAGATCCATTCCGCTGGAGAGCTGCTTTAACATCAACAAAAGAGCGGATTCAAAAAACAAACATCTCGTCGCGCTTTATACCAAGGATGAGTACTTTGCTATCGCTGCTGACAGCGAGATCGAGCAAGAGTCATGGTATGAAGCCCTGGTTGATCTCCATAACAGAGGTAAGGTCCACGAAACTGCCCCGGGCCGTGGAGTTGGCGAGGATAATTACGGGGAAGCCACGCCGGGACCTGCCTTTAAAGAGGTTTGGCAAGTTATATTGAAACCAAAGGGTCTGGGTCACACAAAGAACTTGATTGGAGTTTACAGATTATGCCTCACCAATAAGACTATCAGCTTTGTGAAACTGAATTCGGATGCAGCAGCTGTGGTTTTGCAGTTGATGAACATTAGGAGATGTGGTCACTCTGAAAATTTCTTTTTTATTGAAGTCGGCCGATCTGCTGTGACAGGCCCCGGGGAGTTTTGGATGCAAGTGGATGACTCTGTTGTGGCTCAGAACATGCATGAAACTATATTAGAAGCCATGAAAGCCATGAGTGAGGAGTTCAGACCCCGCAGCAAAAGCCAGTCATCTTCTAACTGCTCAAACCCCATCTCAGTGCCTGTCAGGCGGCATCATCACAATAATCCTCCCCCTAGTCAAGTTGGCTTAGGCAGGCGCTCTCGGGCGGAGAGTGTGACGGCCACTTCTCCTGTTAGGCCTGGCAAACACAGCCATTCGTTTAGAGTGAGAGCATCTAGTGATGGAGAGGGCACCATGTCCAGGCCGGCATCTGTGGACGGGAGTCCAAGCAGCCCGAGCAATGCCCGACCTCAATCGCAACGGCACAGAGGGGGATCCAGACTTCACCCGCCACTCAATCACAGCAGATCAATACCCACGCCCACCTCACGGTGCTCCCCCTCAGCCATCAGCCCCATCAGCCTGTCCTCCAGCAGCACGAGCGGGCATGGCTCCACATCTGACTGCCTCTTTCCACGTCGCTCTAGTGCCTCGATATCTGGATCACCCAGCGATGGCGGATTCATCTCCTCAGATGAATATGGATCCAGCCCCTGTGACTTTCGAAGCTCGTTTCGTAGCGTGACTCCAGACTCGCTTGGCCACACACCACCTGCCAGGGAGGAAGAGATAAACAACTACATCTGCATGGCAAAGCCCGGTTCACTTCCTGGTGCCGGGAGCCAGTCACGAAGCCAATCTAGAGGAACACCGTCAAGACTAGAGGAGCCTGAGTTGGAAAAATGTTTTCGGAAAAGAACTCATTCTTCTGGTGCTTCGCCGCCAACTCCATGCCATCAGAAAACTCCCTCACAATCGCCTGCTGCATCTTTAGAGGAATACACTGTGATGATGCCTACATACCCACGAAGCCGCTCTGCATCATCGTCATCATCAACATATAGGCACTCATTCATGCCAACACATTCATACCCAGAGGAAGGTACAGATGCATCACAAGCAAAAGATGAAAGGGGGCCAGATCACAAAGATGACGGCTATATGCCCATGCTGCCAGGCGTTGCGCCTGCAACCCCTACAACCAAAAGCAGTGACTACATGCCCATGAGTCCAAAAAGTGTGTCTGCACCACAGCAAATTATCAACCCCAGGCAACACTCGCATGTCGACTCCAATGGTTACATGATGATGTCACCAAGTGGCAGCTGCTCTCCAGATGGCACAACAAACTATGGCAAGATCTGGACGAATGGCATCAACCCTAAGCTCTCCGTAGAGAGTATGGAAGGTAAAGTGTCGTCATGTGGAGATTACATAAACATGTCTCCAGCTAGTTGCTCAACAACAAGTACGCCTCCAGACTGCTTTTTCAACCCTGTGGAAGATCCTCCCAGGCCTATGTATGCTTACTACTCTTTGCCCCGCTCATTCAAACATGCTAACAGGAAGCAGGATCAGAGCCCGCTTCGAGTGTCACTGGGCTCGAGTCGATTAGCATACGCTGACTCCTCTTCATCTTCAGCAAGCAGTGACAGCTTGGGTGGTCAAAGCAATTCCCAGCAGTCTGCTGTCAAACCCAAAAGGTCAGAGGGCAACGGTAGGCTGACACGTCCAACACGGCTGTCACTGGACGCCAGCAAAGCAAGTACTCTTCCACGCACACGTGAGAGCCCCTTCCCTCCTGAGCCTAAAAGTCCTGGGGAGTATGTCAACATTGAGTTCAACGACAAGGCATTTTCAGCGAGCTTAGCATCGCTGTTCCCACCTGTTTATTCAGTAAGTGATGCTGAGGCCCCGTCGGATCTTTCATCATCTGAGTACATGAACATGCAGCTGGGATCCCAGGCCAGACGGCTGTGCTCCGAATTGCAGACTTCCTCTTCCAGTTCAGATTATGCCATAATCACACCGTCAAACTCTGTATCCTCGCCTCCACTGCCATGTGAGAGCATATGTGAACGTGACTACATCAGCATGCAGTTAGCATCCTGCACTGGCTTTCCTGACACACAATTGATGCTAATGACCGAGGCCCTGCCAGATGCAGATGCCCCTCCTTGTTCTGTATCACCCAATCATGACGTGACGACATTAAGCGGCGCTCCTGGAAGACCAGCTCTGATTAGCCCTTTATCAGGTCTGAGTGCATTCACACGGGTTAACTCCACTTCCGGACGAAACCAGGGCGCTAAAGTAATACGTGCTGACCCACAGGGTCGACGGCGGCACAGTTCAGAGACTTTTGCATCCACAGCGACCAGAGGGTCAGTCGACACATCGAGCACCTGTCAATCAGGGGATGTAAAGCGCCACAGCTCCGCCTCCTTCGAGAATGTGTGGTTGAGACCTGATCAGGCCTCTGCTGCTTCTACCGCTCCATCTGCATCGACAGTAACCAACGGACGAAGGGAAACCGCTTCAGGCCAAATTCCCACATTAACAAACCATGACCAGAACGGCCTGAACTACATTGATCTGGATCTAATGCAGAATGGAGAGCAACATGTTCCTGATTGGAATGCTTTCCAGTCTCGGCCGGTGGAGCTGGGAGCTGTTGAAGGTATAGAAGAGCTCAGTGCCTATGCCAGTATAAACTTCCAGAAATCAGATGAGACCAGAGGGAATCTCACACACAGAGAAGAATGA

>Sal_sa1 [Atlantic salmon (Salmo salar) Irs1]

ATGGCTAGCCCAACTACAGAGCAACAGGGTTGTTTTTCAGATGTAAGAAAGGTGGGTTATTTAAGGAAACCTAAAAGCATGCACAAGAGGTTTTTTGTCCTGCGGGATGCTAGTGCTGCAGGACCCTCCAGGTTGGAGTACTACGAAAATGAAAAAAAATGGAGACACAAGTCCGGGGTGCCTAAAAGGTCAATCCTGCTGGAGAGCTGCTTCAACATAAATAAAAGGGCAGATTCAAAAAACAAATACCTGGTGGCTCTTTACACCAAGGATGAGTATTTCTCTATTGCAGCGGACAGCGAGCAGGAACAAGACAGGTGGCACCAAGCACTAGTGGACCTACACAACAAAGGTAAAGTTCATGATGCTGCTGTTGGTGGCAGTGGGATGGCAGAGGAGAATTATGGAGAAGAAACCATGCCAGAACCTGCCTTTAAAGAAGTCTGGCAAGTTATTTTGAAACCAAAGGGCCTGGGACACACCAAAAATTTTATTGGGATTTACAGATTGTGCCTAACAAATAAAACCATCAGCTTTGTGAAATTGAACTCCGACGCAGCGGCGGTCGTGCTGCAATTAATGAATATAAGGCGATGCGGTCATTCAGAGAATTTATTTTTCATTGAAGTGGGAAGATCCGCAGTCACAGGACCAGGGGAATTTTGGATGCAAGTGGACGACTCTGTTGTTGCTCAAAATATGCATGAAACCATCCTGGAGGCGATGAAAGCGATGAGTGAGGAATTTCGTCCCCGGAGCAAAAGCCATTCCTCGTCAAACTGCTCCAACCCAATCTCTGTGCCTTTGAGAAGGCATCACCACAACAACTTGCCACCTAGCCAAGTGGGACTGGGAAGGAGGTCCCGGACTGAGAGTGTGACGGCCACCTCGCCTGCTGGCCCGGGAAAGCACAGTCATTCATTCAGAGTGAGAGCCTCTAGTGATGGCGAAGGAACCATGTCCAGACCAGCCTCAGTGGATGGGAGCCCCTGTGCTGCCAGGACACAATCTCACAGACACAGAGGGGCCTCCCGCCTCCACCCTCCCCTCAACCACAGCAGGTCTATCCCCATGCCCTCTTCGCGCTGCTCCCCCTCAGCAATCAGCCCAGTTAGCCTGTCCTCCAGCAGTACGAGCGGGCACGGCTCCACTTCAGACTGTCTCTACCCCTGCCGCTCCAGTGCCTCCATATCTGGCTCTCCTATTGACGGGGGATTCATTTCCTCTGATGAGTATGGATCCAGCCCCTGTGACTTCAGGTGCTCCTTCCGCAGTGTCACACCTGACTCCTTGGGTCACACACCGCCCGCCAGGGAGGAAGAACTCAACAACTACATCTGCATGGCGAAGTCTGCAACTCTTCTGAGGGGCCACTGTGGCTGCAGCCCCCACCCCCATGGCACGCCATCCCACCTGGACGAGCCTGAGCTGGAGAAGTGCTTCAGGAAACGGACACACTCCTCGAGCACATCTCCCCTGACAGTGTGCCACCAGAAGACCCCCTCTCAGTCCTCCACAGTATCGCTGGAGGAGTACACAGTAATGCAGCCTGCATACTCGTCATGCAGCCGAGCATCCAGCTACAGGCACTCTGCCTTCATGCCCACACACTCATACCCAGAGGAGGGCATAGACATCTCCTTAGAGGGCAACAGGGTGAGCCAAAAAGATGATGGCTACATGCCCATGACCCCAGGTGTGGCACCTGCCACAGGTAAAAGTGCCGACTACATGCCCATGAGCCCCAAAATTGTGTCAGTGCCACAGCAGATTAACTCCCACCAGCACCCCAAAATGGACTCCAATGGGTACATGATGATGTCACCCAGCGGGAGCTGCTCACCAGACACCACAAACTACGGTCAAATCTGGACCAATGGGGTCAACCCTAAGCTGTCTGTCCAGAGCACGGAGGATAAATTGTTGTCGCGCGGGGATTACATGAACATGTCACCGGCGAGCTGCTCCATGACTAGCACGCCGCCAGACTGCTACATCAACCTGGTAGAGGATCCACCCAAGTCCATGTATGCCTACTTTTCTTTGCCTCGCTCTTTTGAACACAGTCACAGGAAGCTGGACCAGAACCCCCTTCACCTTTCGCTCAGCTCTGGACACCTGGCCTTCGGACACTCTTTAGCCTCCTCCACGAGCAGCGACCGTCTGATGGGACAGGGCTACAGTGGTCAGCCCGTGGTCAAGCCCAAGATAGCTGATGTGGACAGCAGGCTGACCAGGCCCACGCGCCTCTCTCTGGAGGGCAACAAAGCCAGCACCCTGCCTCGCACCCATGAGTGCCCCTTCCCTGCAGATCCCAAGAGCCCCGGGGAGTATGTCAACATAGAGTTCAACGACAAGGCCTTCTCAGCTGGCTTGGCCACTCACTTCTCCCCTGTGTTCCCAGGGAGTGGCCCAGAGAGCCTAGCAGAGATTTCCTCAGACTACATGAACATGCACCTGGGTGCTCAGGGCAGGGGCATTCCCAGCTGGGCAGCCAAACCCCTAGCCTCCTGCACAGATGGCTATACTATAGTGGCCCCTGTGGCTCCCTCCTCCTCTGTGATCCCCTGTGAGAACCTCTGTAGACGGGACTACAGCAGGGCACAGACTGACTACTCTGACACACAGATGATTCTGAACGCTCCGATGTCGACCATTGATGTCACCCCCTTCTCTGTGTCCCCTCCAAGCCAAGACCTGTCTGTGCTGGGTGCGGGTCCCAGTGCTGTGCCCTTAGGACTAATGGGCCCTCTCTCTGGGCTGAGTGCTTTCACCAGGGTTAACTCTAGTTTCATCTGGAACCAGGGAGCTAAATTGATCTGCGCTGACCAGCAGGGCCGGCGCCGGCACAGCTCCGAGACCTTCTCTTCCACGAGAGTCCTGGTCAGCGCTGCCTCGCCCTCCGTGGAGGAAGTGAAACGTCACAGCTCCTCCTCTTTCGAGAATGTGTGGCTGAAGCCGGGAGAATCCCCATTATCTGCCAACGGGAGGAGAGAAAGCCCATCAGGGCCCAGCTCTAACGGACAGAACCAGAACAGGCTGAACTACATCGACCTGGACCTGGCTCAGGACCAGAACCTCCCAGAGTGGAGTTCCCTCTGGGCCAGAGCCAGGGACACGCAAGGTGGGAGTGCCCCAGAGGACCTGCGCGCCTATGCCAGCATTAGTTTTCAGAAAGCAGACGAGTCGAGGATGAACCCCGCTCACAGAGAAGNNNNN

>Ast_me1 [Mexican tetra (Astyanax mexicanus) Irs1]

ATGGCAAGTCCCTCTGCAGAGCAGGGATGTTTCTCAGACGTGAGAAAGGTGGGTTATTTAAGGAAACCGAAAAGCATGCACAAAAGGTTTTTTGTCCTGCGGGCGGCGAGCTCTGCTGGACCTTCCCGGCTGGAGTACTACGAGAACGAGAAGAAATGGAGACACAAGTCTGGGGCTCCTAAAAGGTCAATTCCACTTGAGAACTGCTTCAACATAAACAAAAGAGCGGATTCCAAAAACAAATACCTTGTAGCGCTCTACACCAAGGACGAGTACTTCGCAATCGCTGCAGACAACGAGCTGGAGCAGGACACATGGTATCAGGCCCTTGTAGACCTTCACAACAGAGGTAAGGTCCATGAAAGCGCCGGGAGCAACAAAACCGGAATTGGGGAGGATAATTATGGTGAAATTACACCGGGTCCTGCCTTCAAAGAAGTGTGGCAGGTTATATTGAAACCAAAGGGACTTGGGCAGACAAAAAACCTGACTGGGGTTTACAGATTATGTCTAACCAACAAGACTTTGGGCTTTGTAAAACTAAATTCAGATGCAGCAGCAGTGGTGCTTCAGCTAATGAACATTCGAAGATGTGGCCACTCTGAAAATTTCTTCTTTATAGAAGTTGGCCGGTCTGCAGTGACTGGGCCAGGTGAGTTCTGGATGCAGGTGGACGACTCTGTTGTGGCTCAGAACATGCACGAAACCATTTTAGAAGCAATGAAAGCAATGAGTGAAGAGTTTCGACCACGCAGCAAAAGCCAGTCCTCGTCAAACTGCTCCAACCCCATTTCTGTGCCAGTCAGGACAAGGCATCATCACAACAACCCTCCCCCGAGCCAGGTCGGGCTGGGAAGGCGCTCCAGGGCTGAAAGTGTGACCGCCACCTCTCCTGCAGGCGCAAGCAAACACAGCCAGTCATTCAGAGTGAGAGCATCCAGTGATGGAGAGGGTACCATGTCAAGACCTGCATCTGTAGATGGGAGCCCGAGTAGCCCATGCACCGCAAGACCTCATTCTCAGAGGCATAGAGGTGGATCCAGGTTGCATCCACCATTAAACCATAGCAGATCCATTCCCATGCCCTCATCACGATGCTCCCCATCAGCTGTCAGCCCAGTCAGCCTGTCTTCCAGCAGCACCAGTGGACATGGGTCTACCTCCGACTGCCTTTTCCCCAGACGCTCCAGTGCCTCGATCTCAGGATCACCCAGCGACGGGGGCTTCATCTCTTCTGACGAATATGGTTCCAGTCCGTGCGATTTTCGGAGCTCGTTTCGCAGCGTAACCCCTGATTCACTTGGACATACACCTCCAGCCAAGGAGGAGGAGCTGAACAACTATATCTGCATGGCCAAACCTGGTTCACCGACTAGCAATGGGAGCCAGTCGAGGAGTCATTCCAGAGGTACACCAAATCGCCTGGATGAACCAGAGCTGGAGAAATGTTTCAGAAAAAGAACTCATTCTTCTGGCACATCACCCCCAACATTAAGCCACCAGAAAACAACTTTGCAATCATCTGCTGCTACGCTGGACGAATACACAGTCATGACTCCTGCTTATTCACATAGCCGATCTGCATCCTCCTCCTCGTCATCTTCAACATATAGGAGCTCTTTCATGCCCACACGCTTGTATCCAGAAGAAAGTTTGGAAATGCAACCAGCAGAAGGCAGCAATGGGTCAAGTCACAAAGATGATGGTTACATGCCGATGTCACCAGGTGTAGCTCCGTCTGTGGTAATGGGAAAGACTGGTGACTATATGCCCATGAGTCCCAAAAGCGTATCAGCCCCACAGCAGATTGTCAACCCACGACAGCATTCGCGAGTCGACACAAACGGCTACATGATGATGTCCCCAAGTGGTAGCTGTTCGCCAGATGTTTCATCAAACTACAGAAAGATTTGGAGCAATGGTGTTAACCCCAAGCTCTCAGTTGAGAGCACTGAGGAGAAAGTGTCCTGTGGAGACTACATAAACATGTCTCCTGCTAGCTGCTCCACCATCGGAACTCCTCCGGACTGCTACTTCAATCCTGTTGATGAGCCATCCAGGCCAGCCTATGCATACTTCTCCTTGCCTCGCTCCTTTAAACACGCCAACAGGAAACAGGACCAGAGTCCGTTACGGGTGTCGCTTGGCACTAGTCGCTTGGCTTGCACAGAATCTTCCTCGTCTTCAGCTAGCAGTGACAGTCTGGGTGGGCAGTGCAGTTCACACCCACCTGCTGTCAGGCCCAAAAGGTCAGAGGTCAGCTCTAGACTGGCACGACCAACGAGGCTGTCCCTGGATGCTAACAAAGCCAGCACACTTCCACGAATGCGCGAAAGCCCGTTTCCTCCTGAGCCTAAAAGTCCTGGAGAGTATGTCAACATTGAATTTAACGACAAGGCATTTTCAGCAAGCTTGGCTTCCTTATTTTCACCAGTATTTACTGGCAATGGAAATGATATCCAATCTGTGCAGTCATCCACTGACTACATGAACATACAACTGGGTTCAACAAGCTCGCTCTCAAGCTCCAGAATACCATCGTCCTGTTCTGACTATGCAGTAATTGCTCCATCAGAGACTTCTGCTTCTCAGCCTCTGCCTTGTGAAAGCATTTGTGAACGTGACTATATCAGCATGCAACTCGCCGTGTCTTGTTCAGGCTATCCTAATTCCCAGATGATGCTCATGCACTCTTCCTTACCAGACTCGGATGCCCCTCACTGCTCAGATTCACCCAATGATGGCATCTTAACCCTCAGTGCAGGTCCTGGTAGGCCTGGTCTTGGTCCTCTGACGGGTCTCAGCGCTTTCACCAGAATAAATTCCATTCCTGCCCGGGACCAGGGTGCTAAAGTGATCCGGGCCGACCCACAAGGCAGGAGACGGCACAGCTCTGAGACGTTTGCCTCCACAACGACCAAAGGGGCGGTAGGCACAACAGCGGGCATTCAGTCTGAAGATGTCAAGCGCCACAGCTCTGCCTCGTTTGAGAACGTTTGGCTAAAGCCTGTTGAGGCTGCTGTTACAACAACTACTACTACTTTTGCTTCTTCCTCTCCAGCTGCCAGCACATCAAGTGGACCGAGAGAAACATCAACAGCTGCCACCTTGGCCCAGCAGAAAAATAATGGGCTGAACTACATCGATCTGGATCTGGCGCAGAATCGACTGCAGGCTGTTCCGGACTGGAGCTTGTTCCAGGCCCGACAGGCGGAGCTGGATGCAACAGGAGGAGCGGAGGAACTTAGTGCCTATGCCAGTATCAGCTTCCAGAAAGCAGATGAGACCAGAGGAAACCTCACTCACAGAGAAGAGTGA

>Clu_ha1 [Atlantic herring (Clupea harengus) Irs1]

ATGGCTAGTCCAACCTCAGAGCAGGGTTGTTTTTCGGACGTAAGAAAGGTGGGATATTTAAGGAAGCCTAAAAGCATGCACAAGAGGTTTTTTGTCCTGCGCGTCGAGAGTGCGGCGGGACCATCCCGGCTGGAGTACTACGAGAATGAGAAGAAATGGAGACACAAGTCCGGAGCTCCTAAACGTTCCATACCGCTGGAGAGTTGTTTCAACATCAACAAGCGAGCGGATTCCAAAAACAAATTTCTGGTTGCTCTATACACTAAGGACGAGTACTTTGCTATTGCTGCCGACAGCGAGACCGAGCAGGATGCCTGGTACCAAGCACTGGTAGACCTGCACAACAGAGGTAAAATCCACGACACACCCGCAGGCAACGGAATAGAAGAGGATAATTACAGCGAGGCTTCGCCGGGACCTGCCTTCAAAGAGGTCTGGCAGGTTATTTTGAAACCAAAGGGCCTGGGACACACGAAGAACCTCATTGGGATTTACAGACTATGCCTGACGAACAAAACTATCAGCTTTGTGAAGCTGAACTCAGACGCTGCTGCCGTTGTTCTGCAGCTGATGAACATCCGCCGATGCGGCCACTCTGAGAACTTCTTTTTCATCGAGGTGGGGCGCTCCGCAGTGACCGGCCCCGGTGAGTTCTGGATGCAGGTGGACGATTCAGTTGTGGCACAGAATATGCACGAAACCATCCTGGAAGCCATGAAAGCAATGAGTGAAGAGTTCCGGCCCCGCAGCAAGAGCCAGTCGTCCTCCTCTCACTGTTCCAACCCCATCTCGGTGCCTGTACGCCGGCATCATCATAACAACCCCCCGCCAAGTCAGGTGGGAATGGGACGTCGCTCGCGGGCGGAGAGTGTGACCGCCACATCCCCAGCCGGTGCAGGCAAGCACAGTCACTCATTCCGCGTTCGTGCATCCAGTGATGGGGAGGGCAGCATGTCGCGGCCGGCTTCAGTGGACGGTAGCAGTCCCAGCAGTCCCTGTATTGGCGGATCACGCACGCAGTCCTACCGGCAGCGTGCAGGGGGGTCTGCACGTCTACACCCACCCCTCAATCACAGTCGCTCTATCCCCATGGCATCAAGTTCACGCTGCTCCCCGTCAGCCGTGAGCCCTGTCAGCTTGTCGTCCAGCAGCACCAGCGGTCATGGCTCCACCTCTGATGGTCTGTTCCCACGGCGATCCAGTGCCTCCATCTCAGGGTCGCCCAGCGATGGTGGCTTTGTGTCATCGGATGAGTACGGCTCGAGCCCCGGAGACTTCAGGGGAGGAGGGTTCCGGAGTGCTACACCTGACTTCCTTGGTCACACCCCACCTGCAAGGGAGGAGGAGCCTTTGGTTTCGGATTACATCACCATGACCCAGACACCGCGGCGACCAGACGACCCTGAGCCAGAAAAGAGCTTCCGCAAGCGAACCCACTCCTCAGGTGCTGCGTCACCCCCGGTGACCTGCCACCATCAGAAGACGCCTTCTCAGTCATCGGCGACGTCGCTGGAGGAGTATGCAGTCATGCTGCCCGCTTACCCCTGCACCCGCACCACCCCTTCTGCCCCAGCAACGCCCTCTCCCTCACCCTCATTAACATCGGCAACCTGCCGGCACTCTGCATTTGTTACCCCACACTCATACCCAGAGGAGGGGCTAGAAGTGGTAGGGGGTGGGCCCTCAGCCAAGGATGATGGCTACATGCCCATGTCGCCTGGTGTTGCTCCCACCTCTGTCATCTCCATGACAAGAGTCACCACAGGTACAGTGTCTGGGGACGACTACATGCCCATGAGCCCCAAGAGTGTGTCTGCACCGCAGCAGATCGTCAACCCCTCACCGAGGCACCGTCTCCATGAAAACGGTGATGTGGATCCTAGTGGCTATATGATGATGTGGCCAAGCAGCAGCTGCTCACCCGACGGCCCTGGAAGGCTCTTGAACGGTGGCGTTTCAGCGGGCCGTGTGACAGCCCCTTTGGCGCCCCCTTCAGGCGACTACATGAACATGTCTCCCGCCAGCGGCTCAGCAACCAGCACCCCTCCAGACTGTTACTTCAGCACCGGCACAGGTGGATACTTCTCCCTGCCACGCTCCTTTAAGCATGCACACCGCAAGCAGCCTGATCACGCCTCCTCCTCCTCATCACCACGCCTGTCGTTAGGCGCCGGGCGCCGTGCCTACACCGACTCTTCTTCCTCCTCTGCTAGCAGCGATAGTCTGGGTGGTGGTGGTGGCGGCGGTGGAGTCGGCGGCGGCGGCAGTCACGGCAACAGCCAGCATCCGACCCGGCAGCAGCCGCCTCTGGGCCGCTCCAAACGCCTGTCAGAGACGGGGGAGAGTCGTCTGGCGCGACCCACACGTCTGGCCCTGGAGGGGGGACGGGCGAGCACGCTTCCCCGCTCACGCCAGACTCCCTACCAACCTGAGCCCCGCAGCCCAGGAGAGTATGTCAACATTCAGTTCAGCGATCAATCCTTCACCTCCAGCCTTGTAGCTCTCTTCCCTGCCGCTGAAAGGCCTGCCTCCTCTGATGGATCAGCCAAGCTCTTCCCTTCCTCTGATAGGGTAGCTGAGCGGTTCCCGGTCTCTAAAAAGCCAGTTGAGCTCTCTGCCCCACCTCCCTCCTCCACTGACTACATGAACATGCAGTTAGGGTCTTCAAATGGGCTTTCAGTCACGGAGGTAGCGACCTCTGACATCCATGACCTGTCCGACTATGCAGTAGTGACCCCTGGCCCAGTTCCTCCCGCCTCTGCTGCGTTGCCATGCGACAGCATCTGTCAGCGCGACTACCTGAGCATGCAGGTCTTCTCTGGATCTCCCGTGATGCTCCTCAACCCCTCCTCCTTGCCTGACGACCCGCCCACTGGTCCCTCCACCCATTCCATCTCGCCCAATGGCAGTGAGATGCCGGGGATTGGCGGCCCACTGATTGGCCCCCTTTCTGGCCTCAGCGCCTTCACACGTGTCTCCTCAGTAACACCCCCCAGCCGAAGCCAAGGCATCACCAAGGTGATCCGTGCCGATCCCCAGGGGCGGCGGCGACACAGCTCGGAAACCTTCGCTTCCACAACGACCAAGAGCCCGGTCGGCACGGCGACCACCCCAACGTGCCCTGCCACAGATGAAGTAAAGCGCCACAGCTCTGCCTCCTTTGAGAACGTCTGGCTTAAACCCGGCGGAGAGCCCATCTCCATGACGACATCCCCAGCGGCAGCCGCTGCATCTGCGGCTGGCACGAGGAGATCAGGGGGAGAGACGAGTCCAATGGCGGCCCTCCTGACCCGGAACCAGAACGGGCTGAACTACATCGACCTGGACCTGGCCCACGGACGTGAGCAGCTGCCGGCTGATTGGAGCACGTTCAGCACCAAGGCTGGAGATGTGGGAGTAGGCGGATCATCTGAGGAGCTGAATGCCTACGCCAGCATCAGCTTCAGCTCGCCACAAGAAAACAGAGAAGAGTGA

>Cal_mi1 [Elephant shark (Callorhinchus milii) Irs1]

ATGGCGAGTCCGCCGCCGGAGCCCAGCGCCGAGAGCTTCTCGGACGTGAGGAAGGTGGGCTACTTGAGGAAACCCAAGAGCATGCACAAGAGGTTCTTCGTGCTGCGGGCGCCGAGCGAGCAGGGGCCGGCCCGGCTGGAGTACTACGAGAACGAGAAGAAATGGAGGCACAAGGCGAGTGCCCCGAAGCGCTCGATCCACCTGGACTGCTGCTTCAACATCAACAAGAGGGCCGACTCGAAGAACAAGCACCTGGTGGCTCTCTACACCAAGGACGAGTACTTCGCCATCGCCACCGACTGCGAGGGCGAGCAGGAAAGTTGGTACCAGGCGCTGGTCGAGCTCCACAACAAGGGCAAGGTCCCCGAGGCCGGGGTCGGGAGCGATCGGGAACGGGACGGTGGCGGGACGGCCGCCAACGTGGTCGTCGTCGGCGGCGGCGGCGGCCTCGGCGCGGGAGAAGAAAACTACGGAGAGCTGAATCCGGGGCCGGCTTTCAAAGAAGTTTGGCAAGTGGTCCTCAAGCCCAAGGGCTTGGGACAGACCAAGAACCTGGTCGGGATTTACAGGTTGTGTTTGACCAACAAGACCATCAGTTTGGTGAAGCTCAACTCGGATGCTGCGGCCGTGGTCCTGCAGCTCATGAACATCCGCAGATGTGGACATTCAGAAAATTTCTTCTTCATTGAGGTGGGCAGATCCGCCGTGACCGGACCCGGAGAGTTTTGGATGCAGGTGGATGATTCTGTGGTGGCTCAGAACATGCACGAAACCATCCTGGAAGCCATGAAGGCCATGAGTGAGGAATTTCGACCACGGAGCAAAAGTCAGTCGTCTTCCAGTTCCAACCCGATCTCGGTGCCTCTGAGGAGACATCTGAACAACCCGCCCCCGAGCCAGGTTGGATTGAGCCGGAGGTCCAGGACCGAGAGCATCACTGCCACCTCACCTGCTGGTAAACACAACTCGTTCCGGGTACGAGCTTCCAGTGATGGCGAAGGCACCATGTCCAGACCCGCCTCTGTTGACGGCAGTCCAGTTAGCCCCAGTGCCACAAGGACACATTCTCACCGTCACCGAGGTAGTTCTAGGCTGCATCCTCCTCTCAATCACAGCAGATCTATTCCTATGCCATCCACGCGTTGTTCCCCTTCAGCCACGAGTCCAGTTAGTTTATCATCCAGCAGCACTAGTGGTCATGGATCCACTTCTGACAGCCTTTTTCCTAGGCGCTCCAGCGCATCCATCTCAGGATCTCCCAGTGATGGGGGATTTATTTCTTCAGATGAGTATGGATCCAGTCCGTGTGACTTCAGAAATTCCTTCAGAAGCGTCACTCCAGATTCCTTGGGACATACTCCCCCTGCCAAAGAGGAGCAGGACTTAAATAATTACATCTGCATGGGGAAGCATTCGAACATGGTGCCAAATGGTCACTACTACAGGGGCAGTCAAAGGTGGACTCCAACTAAAATTGAAGAGGCCGATTCAGAAAAAGGATTGAGGAAGAGGATGCATTCGGCAGGTACTTCACCCCCCACAGTGGCACACCAGAAGACCCCTTCCCAATCTTCTGCATCATTAGAAGAGTATACAGAGATGACCCCATGTTTCCCTGGCAGCCGTTTATCATCCTGCAAAAATTCTGCTTTTGTACCCACTCATTCGTACCCCGAGGAGTGTTTAGATCTTCACCTGGGAGAAGGCAATCGCTTTAGTCAAGTGGATGATGGTTACATGCCCATGTCACCTGGAGTAGCCCCTGTGCCTAATAAAAATGACTACATGCCAATGAGTCCGAAAAGTGTGTCTGCTCCACAACAGATTATTAATCCACGGCAGCACTCCCGAATAGATTCAAATGGTTACATGATGATGTCACCTAGTGGTAGTTGCTCTCCGGAAAATGTGAACTATGGAAAAATATGGACCAACGGAGGGAATTCTAAACTTTCAGTGGAGAGCAATGATGGGAAACTGTCCTGTAGCGATTACATTAACATGTCTCCTGCAAGTGGTTCGACTACAAGCACTCCACCAGACTGCTTCTTCAATCCAGTGGAAGAGCCTCCCAAACCAGTTTATTCCTACTTTTCTCTGCCTCGTTCTTTTAAGCATGTGAACAGAAAATCCGAGGAGTCACGGTTACGAATTTGTGTCAGTTCAGTTCGCCTGCTCCATGCAGAGGATTCTTCCTCTTCCACAAGTAGTGACAGTATAGGAGGGCATGATGCTGCGCATCAGGCAGCAAACTCTCTTAAAAGGGAAGCCTGCCTACAGAAGAAAGGTAAACTTGTACGGCCCACAAGACTGTCGCTTGATAACACTAAGGCGAGCACGCTCCCCAGGACACGAGAGCATCCCCTTCCACCAGAACCTAAAAGCCCAGGTGAATATGTAAATATTGAGTTTAGTGACAAAACCTATTCTTCCAGTTCATTGTCGCTCATTGAGTCCACTGCAGTCACAGGCAGCCATCCTGTTCGACAAAGGCCATACATGTCTGAGTACATGAACATGAATTTAAGAAGGGATCTGCCCAAATTTAGTTACGTAACAAAACCTTCAGTAGACATCCCAGTCTCTGGTGCCGTCTGCACCTCCAGCACCTGGAGAACAGACAGAGGACAGGCAAACTGTGATTATGTGAGCATGCAGCTTCCTGTCACCTTGGCTGACTACTCAGAGACGCCGATCATGAGCAACTATGCCGAGATGACAGTCGGTGGAGGTACAACATCTTCGAAGTCTGTACTACCTCAGCAAGAAACCAACACGCTGAACAGTGTTCCAAACGCAGCATGTGCAAGTCTGATGGACCAGATTTCAGGGAGGAGTGCCTTCACTGTACTAAATCTCAGCCCCAATCGCAACCAAGGTGCCAAAGTTATCCGTGTTGATCCTCAAGGAAGAAGACGGCACAGTTCAGAGACTTTTACTTCTACGGCCAAGGTGCCTGGTGTGACTCAGTCTTATGCAGATCATGTGAAGCGACATAGCTCTGTGTCCTTTGAGAACGTGTGGCTTAACAAACCAGGGGAGCCTCTGGCTTCTGCCAGCAAAGAACAACTAAGCACTACGGTTCAGAACAGCTCAACAGCCTTCGAAAACGGTATGAATTACATTGATTTGGACTTGGTCAAGGATTTTAACAACCCAGAGTGGACCTCCTCACAGCCTCTAACCTCTCATCAACCTCATGCAAGTACCTCTGGTGATGAAGATCTGAGTGCATACGCAAGCATCACCTTCCAGAAGCCAGATGAGTTAAGAAGCAATCCTGTAGAAAGGGAAGAATGA

>Hom_sa2 [Human (Homo sapiens) IRS2]

ATGGCGAGCCCGCCGCGGCACGGGCCGCCCGGGCCGGCGAGCGGAGACGGCCCCAACCTCAACAACAACAACAACAACAACAACCACAGCGTGCGCAAGTGCGGCTACCTGCGCAAGCAGAAGCATGGCCACAAGCGCTTCTTCGTGCTGCGCGGACCCGGCGCGGGCGGCGACGAGGCGACGGCGGGCGGGGGGTCGGCGCCGCAACCGCCGCGGCTCGAGTACTACGAGAGCGAGAAAAAGTGGCGGAGCAAGGCAGGCGCGCCGAAACGGGTGATCGCTCTCGACTGCTGCCTGAACATCAACAAGCGCGCCGACGCCAAGCACAAGTACCTGATCGCCCTCTACACCAAGGACGAGTACTTCGCCGTGGCCGCCGAGAACGAGCAGGAGCAGGAGGGCTGGTACCGCGCGCTCACCGACCTGGTCAGCGAGGGCCGCGCGGCCGCCGGAGACGCGCCCCCCGCCGCCGCGCCCGCCGCGTCCTGCAGCGCCTCCCTGCCCGGCGCCCTGGGCGGCTCTGCCGGCGCCGCCGGGGCCGAGGACAGCTACGGGCTGGTGGCTCCCGCCACGGCCGCCTACCGTGAGGTGTGGCAGGTGAACCTGAAGCCCAAGGGTCTGGGCCAGAGCAAGAACCTGACGGGGGTGTACCGTCTGTGCCTGTCTGCGCGCACCATCGGCTTCGTGAAGCTCAACTGCGAGCAGCCGTCGGTGACGCTGCAGCTCATGAACATCCGCCGCTGCGGCCACTCGGACAGCTTCTTCTTCATCGAGGTGGGCCGCTCGGCCGTCACAGGCCCCGGCGAGCTGTGGATGCAGGCGGACGACTCGGTGGTGGCGCAGAACATCCACGAGACCATCCTGGAGGCCATGAAGGCGCTCAAGGAGCTCTTCGAGTTCCGGCCGCGCAGTAAGAGCCAATCGTCGGGGTCGTCGGCCACGCACCCCATCAGCGTCCCCGGCGCGCGCCGCCACCACCACCTGGTCAACCTGCCCCCCAGCCAGACGGGCCTGGTGCGCCGCTCGCGCACCGACAGCCTGGCCGCCACCCCGCCGGCGGCCAAGTGCAGCTCGTGCCGGGTGCGCACCGCCAGCGAGGGCGACGGCGGCGCGGCGGCGGGAGCGGCGGCCGCGGGCGCCAGGCCGGTGTCGGTGGCTGGGAGCCCCCTGAGCCCCGGGCCGGTGCGCGCGCCCCTGAGCCGCTCGCACACCCTGAGCGGCGGCTGCGGCGGCCGCGGGAGCAAGGTGGCGCTGCTGCCGGCAGGGGGCGCGCTGCAACACAGCCGCTCCATGTCCATGCCCGTGGCGCACTCGCCGCCCGCCGCCACCAGCCCCGGCTCCCTGTCGTCCAGCAGCGGCCACGGCTCGGGCTCCTACCCGCCGCCGCCCGGCCCGCACCCGCCTCTGCCGCATCCGCTGCACCACGGCCCCGGCCAGCGGCCCTCCAGCGGCAGCGCCTCCGCCTCGGGCTCCCCCAGCGACCCCGGCTTCATGTCCCTGGACGAGTACGGCTCCAGCCCAGGCGACCTGCGCGCCTTCTGCAGCCACCGAAGCAACACGCCCGAGTCCATCGCGGAGACGCCCCCGGCCCGAGACGGCGGCGGCGGCGGTGAGTTCTACGGGTACATGACCATGGACAGGCCCCTGAGCCACTGTGGCCGCTCCTACCGCCGGGTCTCGGGGGACGCGGCCCAGGACCTGGACCGAGGGCTGCGCAAGAGGACCTACTCCCTGACCACGCCAGCCCGGCAGCGGCCGGTGCCCCAGCCCTCCTCTGCCTCGCTGGATGAATACACCCTGATGCGGGCCACCTTCTCGGGCAGCGCGGGCCGCCTCTGCCCGTCCTGCCCCGCGTCCTCTCCCAAGGTGGCCTACCACCCCTACCCAGAGGACTACGGAGACATCGAGATCGGCTCCCACAGGAGCTCCAGCAGCAACCTGGGGGCAGACGACGGCTACATGCCCATGACGCCCGGCGCGGCCCTCGCGGGCAGTGGGAGCGGCAGCTGCAGGAGCGACGACTACATGCCCATGAGCCCCGCCAGCGTGTCCGCCCCCAAGCAGATCTTGCAGCCCAGGGCCGCCGCCGCCGCCGCCGCCGCCGTGCCTTCTGCGGGGCCTGCGGGGCCAGCACCCACCTCTGCGGCGGGCAGGACATTCCCGGCGAGCGGGGGCGGCTACAAGGCCAGCTCGCCCGCCGAGAGCTCCCCCGAGGACAGTGGGTACATGCGCATGTGGTGCGGTTCCAAGCTGTCCATGGAGCATGCAGATGGCAAGCTGCTGCCCAACGGGGACTACCTCAACGTGTCCCCCAGCGACGCGGTCACCACGGGCACCCCGCCCGACTTCTTCTCCGCAGCCCTGCACCCCGGCGGGGAGCCGCTCAGGGGCGTTCCCGGCTGCTGCTACAGCTCCTTGCCCCGCTCCTACAAGGCCCCCTACACCTGTGGCGGGGACAGCGACCAGTACGTGCTCATGAGCTCCCCCGTGGGGCGCATCCTGGAGGAGGAGCGTCTGGAGCCTCAGGCCACGCCAGGGCCCAGCCAGGCGGCCAGCGCCTTCGGGGCCGGCCCCACGCAGCCCCCTCACCCTGTAGTGCCTTCGCCCGTGCGGCCTAGCGGCGGCCGCCCGGAGGGCTTCTTGGGCCAGCGCGGCCGGGCGGTGAGGCCCACGCGCCTGTCCCTGGAGGGGCTGCCCAGCCTGCCCAGCATGCACGAGTACCCACTGCCACCGGAGCCCAAGAGCCCCGGCGAGTACATCAACATCGACTTTGGCGAGCCCGGGGCCCGCCTGTCGCCGCCCGCGCCTCCCCTGCTGGCGTCGGCGGCCTCGTCCTCCTCGCTCTTGTCCGCCAGCAGCCCGGCCTCGTCGCTGGGCTCAGGCACCCCGGGCACCAGCAGCGACAGCCGGCAGCGGTCTCCGCTCTCCGACTACATGAACCTCGACTTCAGCTCCCCCAAGTCTCCTAAGCCGGGCGCCCCGAGCGGCCACCCCGTGGGCTCCTTGGACGGCCTCCTGTCCCCCGAGGCCTCCTCCCCGTATCCGCCGTTGCCCCCGCGTCCGTCCGCGTCCCCGTCGTCGTCTCTGCAGCCGCCGCCACCGCCGCCGGCCCCGGGGGAGCTGTACCGCCTGCCCCCCGCCTCGGCCGTTGCCACCGCCCAGGGCCCGGGCGCCGCCTCATCGTTGTCCTCGGACACCGGGGACAATGGTGACTACACCGAGATGGCTTTTGGTGTGGCCGCCACCCCGCCGCAACCTATCGCGGCCCCCCCGAAGCCAGAAGCTGCCCGCGTGGCCAGCCCGACGTCGGGCGTGAAGAGGCTGAGCCTCATGGAGCAGGTGTCGGGAGTCGAGGCCTTCCTGCAGGCCAGCCAGCCCCCGGACCCCCACCGCGGCGCCAAGGTCATCCGCGCAGACCCGCAGGGGGGCCGCCGCCGCCACAGTTCCGAGACCTTCTCCTCCACCACGACGGTCACCCCCGTGTCCCCGTCCTTCGCCCACAACCCCAAGCGCCACAACTCGGCCTCCGTGGAAAATGTCTCTCTCAGGAAAAGCAGCGAGGGCGGCGTGGGTGTCGGCCCTGGAGGGGGCGACGAGCCGCCCACCTCCCCACGACAGTTGCAGCCGGCGCCCCCTTTGGCACCGCAGGGCCGGCCGTGGACCCCGGGTCAGCCCGGGGGCTTGGTCGGTTGTCCTGGGAGCGGTGGATCGCCCATGCGCAGAGAGACCTCTGCCGGCTTCCAGAATGGTCTCAACTACATCGCCATCGACGTGAGGGAGGAGCCCGGGCTGCCACCCCAGCCGCAGCCGCCGCCGCCGCCGCTTCCTCAGCCGGGAGACAAGAGCTCCTGGGGCCGGACCCGAAGCCTCGGGGGTCTCATCAGCGCTGTGGGCGTCGGCAGCACCGGCGGCGGGTGCGGGGGGCCGGGTCCCGGTGCCCTGCCCCCTGCCAACACCTACGCCAGCATTGACTTCTTGTCCCACCACTTGAAGGAGGCCACCATCGTGAAAGAGTGA

>Nom_le2 [Gibbon (Nomascus leucogenys) Irs2]

ATGNTCAACAACAACCACAGCGTGCGCAAGTGCGGCTACTTGCGCAAGCAGAAGCACGGCCACAAGCGATTATTCGTGCTGCGCGGGCCCGGCGCGGGCGGCGACGAGGCGACGGTTGGCGGGGGGTCGGCGCCGCAGCCGCCGCGGCTCGAGTACTACGAGAGCGAGAAAAAGTGGCGGAGCAAGGCAGGCGCGCCGAAGCGGGTGATCGCGCTCGACTGCTGCCTGAACATCAACAAGCGCGCCGACGCCAAGCACAAGTACCTGATCGCCCTCTACACCAAGGACGAGTACTTCGCCGTGGCCGCCGAGAACGAGCAGGAGCAGGAGGGCTGGTACCGCGCGCTCACCGACCTGGTCAGCGAGGGCCGCGCGGCCGCCGGAGACGCGCCCCCCACCGCCGCGCCCGCCGCGTCCTGCAGCGCCTCCCTGCCCGGCGCCCTGGGCGGCTCTGCCGGCGCCGCCGGGGCCGAGGACAGCTACGGGCTGGTGGCGCCCGCCACAGCCGCCTACCGCGAGGTGTGGCAGGTGAACCTGAAGCCCAAGGGTCTGGGCCAGAGCAAGAACCTGACAGGCGTGTACCGTCTGTGCCTGTCGGCGCGCACCATCGGCTTCGTGAAGCTCAACTGCGAGCAGCCGTCGGTGACGCTGCAGCTCATGAACATCCGCCGCTGCGGCCACTCGGACAGCTTCTTTTTCATCGAGGTGGGCCGCTCAGCCGTCACAGGCCCCGGCGAGCTGTGGATGCAGGCGGACGACTCGGTGGTGGCGCAGAACATCCACGAGACCATCCTGGAGGCCATGAAGGCGCTCAAGGAGCTCTTCGAGTTCCGGCCGCGCAGCAAGAGCCAGTCGTCGGGGTCGTCGGCCACACACCCCATCAGCGTCCCCGGCGCGCGCCGCCACCACCACCTGGTCAACCTGCCCCCTAGCCAGACAGGCCTGGTGCGCCGCTCGCGCACCGACAGCCTGGCCGCCACCCCGCCGGCGGCCAAGTGCAGCTCGTGCCGGGTGCGCACCGCCAGCGAGGGCGACGGCGGCGCGGCGGGGGCGGCGGCCGCGGGCGCCAGGCCGGTGTCGGTGGCTGGGAGCCCCCTGAGCCCCGGGCCGGTGCGCGCGCCCCTGAGCCGCTCGCACACCCTGAGCGGCGGCTGCGGCGGCCGCGGGAGCAAGGTGGCGCTGCTGCCGGCAGGGGGCGCGCTGCAGCACAGCCGCTCCATGTCAATGCCCGTGGCGCACTCGCCGCCCGCCGCCACCAGCCCCGGCTCCCTGTCGTCCAGCAGCGGCCACGGCTCGGGCTCCTACCCGCCGCCGCCCGGCCCGCACCCGCCCCTGCCGCATCCGCTGCACCACGGCCCCGGCCAGCGGCCCTCCAGCGGCAGCGCCTCCGCCTCGGGCTCCCCCAGCGACCCCGGCTTCATGTCCCTGGACGAGTACGGCTCCAGCCCAGGCGACCTGCGCGCCTTCTGCAGTCACCGAAGCAACACGCCCGAGTCCATCGCGGAGACGCCCCCAGCCCGAGACGGCGGCGGCGGCGGTGAGTTCTACGGGTACATGACCATGGACAGGCCCCTGAGCCACTGTGGCCGCCCCTACCGCCGGGTCTCGGGGGACGCGGCCCAGGACCTGGACCGAGGGCTGCGCAAGAGGACCTACTCCCTGACCACACCGGCCCGGCAGCGGCCGGTGCCCCAGCCCTCCTCTGCCTCGCTGGATGAGTACACTCTGATGCGGGCCACCTTCTCGGGCAGCGCGGGCCGCCTCTGCCCGTCCTGCCCCGCGTCCTCTCCCAAGGTGGCCTACCATCCCTACCCAGAGGACTACGGAGACATCGAGATCGGCTCCCACAGGAGCTCCAGCAGCAACCTGGGCGCAGACGACGGCTACATGCCCATGACGCCCGGCGCGGCCCTCGCGGGCAGTGGGAGCGGCAGCTGCAGGAGCGACGACTACATGCCCATGAGCCCCGCCAGCGTGTCCGCCCCCAAGCAGATCTTGCAACCCAGGGCCGCCGCCGCCGCCGCCGTGCCCCCTGCGGGGCCTGCGGGGCCAGCGCCCACCTCTGCGGGGGGCAGGACATTCCCGGCGAGCGGGGGTGGCTACAAGGCCAGCTCGCCCGCCGAGAGCTCCCCCGAGGACAGTGGGTACATGCGCATGTGGTGCGGCTCCAAGCTGTCCATGGAGCATGCAGATGGCAAGCTGCTGCCCAACGGGGACTACCTCAACGTGTCCCCCAGCGACGCGGTCACCACGGGCACCCCGCCCGACTTCTTCTCCGCAGCCCTGCACGCCGGCGGGGAGCCGCTCAGGGGCGTTCCCGGCTGCTGCTACAGCTCCTTGCCTCGCTCCTACAAGGCCCCGTACACCTGTGGCGCGGACAGCGACCAGTACGTGCTCATGAGCTCCCCGGTGGGGCGCATCCTGGAGGAGGAGCGGCTGGAGCCTCAGGCCACGCCAGGGCCCAGCCAGGCGGCCAGCGCCTTCGGGGCCGGCCCCACGCAGCCCCCTCACCCCGTAGTGCCTTCGCCCGTGCGGCCCAGCGGCGGCCTAGGCGGCGGCCGCCCGGAGGGCTTCTTGGGCCAGCGCGGCCGGGCGGTGCGGCCCACGCGCCTGTCCCTGGAGGGGCTGCCCAGCCTGCCCAGCATGCACGAGTACCCACTGCCGCCGGAGCCCAAGAGCCCCGGCGAGTACATCAACATCGACTTCGGCGAGTCCGGGGCCCGTCTGTCGCCGCCCGCGCCTCCCCTGCTGGCGTCGGCGGCCTCGTCCTCCTCGCTCTTGTCCGCCAGCAGCCCGGCCTCGTCGCTGGGCTCAGGCACCCCGGGCACCAGCAGCGACAGCCGGCAGCGCTCTCCGCTCTCCGACTACATGAACCTCGACTTCAGCTCCCCCAAGTCTCCTAAACCGGGCGCCCCAAGCGGCCACCCCGTGGGCTCCTTGGACGGCCTCCTGTCCCCCGAGGCCTCCCCGTATCCGCCGCTGCCCCCGCGTCCGTCCGCGTCCCCGTCGTCGTCCCTGCAGCCGCCGCCACCGCCGCCGCCCCCGGGGGAGCTGTACCGCCTGCCCCCCGCCTCGGCCGCTGCCACTGCCCAGGGCCCGGGCGCCGCCTCATTGTCGTCCTCGGACACCGGGGACAATGGTGACTACACCGAGATGGCTTTTGGTGTGGCCGCCACCCCGCCGCAACCCATCGCGGCTCCCCCGAAGCCAGAAGCTGCCCGCGTGGCCAGCCCGACGTCGGGCGTGAAGAGGCTGAGCCTCATGGAGCAGGTGTCAGGGGTCGAGGCCTTCCTGCAGGCCAGCCAGCCCCCGGACCCCCACCGCGGTGCCAAGGTCATCCGCGCAGACCCGCAGGGGGGCCGCCGCCGCCACAGTTCCGAGACCTTCTCCTCCACCACGACGGTCACCCCCGTGTCCCCGTCCTTCGCCCACAACCCCAAGCGCCACAACTCGGCCTCCGTANGAAATGTCTCTCTCAAGAAAAGCAGCGAGGGCGGTGTGGGTGTCGGCCCTGGAGGGGGCGACGAGCCGCCCACCTCCCCCCGACAGTTGCAGCCGGCGCTCCCGGCGCCCCCTTTGGCACCGCAGGGCCGGCCGTGGACCCCGGGTCAGCCCGGGGGCTTGATCGGTTGTCCTGGGAGCGGTGGATCGCCAATGCGCAGAGAGACCTCTGCCGGCTTCCAGAATGGTCTCAACTACATCGCCATCGACGTGAGGGAGGAGCCCGGGCTGCCACCCCAGCCGCAGCCGCAGCCGCCGCCGCCGCTTCCTCAGCCGGGAGACAAGAGCTCCTGGGGTCGGACCCGAAGCCTCGGGGGTCTCATCAGCGCTGTGGGCGTCGGCAGCACTGGCGGCGGGTGCGGGGGGCCGGGTCCCGGTGCCCTGCCCCCTGCCAACACCTACGCCAGCATTGACTTCTTGTCCCACCACTTGAAGGAGGCCACCATCGTGAAAGAGTGA

>Mac_mu2 [Rhesus monkey (Microcebus murinus) Irs2]

ATGGCGAGCCCGCCGCGGCACGGGCCGCCCGGGCCGGCGAGCGGAGACGGCCCCAACCTCAACAACAACAACAACAACAACAACCACAGCGTGCGCAAGTGCGGCTACCTGCGCAAGCAGAAGCACGGCCACAAGCGCTTCTTCGTGCTGCGCGGGCCCGGCACGGGCGGCGACGAGGCGACGGCGGGAGGGGGGTCGGCGCCGCAGCCGCCGCGGCTCGAGTACTACGAGAGCGAGAAAAAGTGGCGGAGCAAGGCAGGCGCGCCGAAGCGGGTGATCGCGCTCGACTGCTGCCTGAACATCAACAAGCGCGCCGACGCCAAGCACAAGTACCTGATCGCCCTCTACACCAAGGACGAGTACTTCGCCGTGGCCGCCGAGAACGAGCAGGAGCAGGAGGGCTGGTACCGCGCGCTCACCGACCTGGTCAGCGAGGGCCGCGCGGCCGCCGCAGACGCGCCCCCCACCGCCGCGCCCGCCGCGTCCTGCAGCGCCTCTCTGCCCGGCGCCCTGGGCGGCTCTGCCGGCGCCGAGGACACCTACGGGCTGGTGGCGCCCGCCACGGCCGCCTACCGCGAGGTGTGGCAGGTGAACCTGAAGCCCAAGGGTCTGGGCCAGAGCAAGAACCTGACGGGCGTGTACCGTCTGTGCCTGTCGGCGCGCACCATCGGCTTCGTGAAGCTCAACTGCGAGCAGCCGTCGGTGACGCTGCAGCTCATGAACATCCGCCGCTGCGGCCACTCGGACAGCTTCTTCTTCATCGAGGTGGGCCGCTCGGCCGTCACGGGTCCCGGCGAGCTGTGGATGCAGGCGGATGACTCGGTGGTGGCGCAGAACATACACGAGACCATCCTGGAGGCCATGAAGGCGCTCAAAGAGCTCTTCGAGTTCCGGCCACGCAGCAAGAGCCAGTCGTCGGGGTCGTCGGCCACACACCCTATCAGCGTCCCCGGCGCGCGCCGCCACCACCACCTGGTCAACCTACCCCCCAGCCAGACGGGCTTGGTGCGCCGTTCGCGCACCGACAGCCTGGCCGCCACCCCGCCGGCGGCCAAGTGCAGCTCGTGCCGGGTGCGCACCGCCAGCGAGGGCGACGGCGGCGCGGCGGCGGGGGCAGCGGCCGCGGGCGCCAGGCCAGTGTCGGTGGCTGGGAGCCCCCTGAGCCCCGGGCCGGTGCGCGCGCCCCTGAGCCGCTCGCACACCCTGAGCGGCGGCTGCGGCGGCCGCGGGAGCAAGGTGGCGCTGCTGCCGGCAGGGGGCGCGCTGCAGCACAGCCGCTCCATGTCCATGCCTGTGGCGCACTCGCCGCCCGCCGCCACCAGCCCCGGCTCCCTGTCGTCCAGCAGCGGCCACGGCTCGGGCTCCTACCCGCCGCCGCCGGGCCCGCACCCGCCCCTGCCGCACCCACTGCACCACGGCCCCGGCCAGCGGCCCTCCAGCGGCAGCGCCTCCGCCTCGGGTTCCCCCAGCGACCCCGGCTTCATGTCCCTGGACGAGTACGGCTCCAGCCCCGGCGACCTGCGCGCCTTCTGCAGCCACCGGAGCAACACGCCCGAGTCCATCGCGGAAACGCCCCCTGCGCGAGACGGCAGCGGCGGCGGTGAGTTCTACGGGTACATGACCATGGACAGGCCCCTGAGCCACTGTGGCCGCCCCTACCGCAGGGTCTCGGGAGACGCGGCCCAGGACCTGGACCGAGGGCTGCGCAAGAGGACCTACTCCCTGACCACGCCGGCCCGGCAGCGGCCGGTGCCCCAGCCCTCCTCCGCCTCTCTGGATGAGTACACCCTGATGCGGGCCACCTTCTCCGGAAGCGCAGGCCGCCTCTGCCCGTCCTGCCCCGCGTCCTCTCCCAAGGTGGCCTACCACCCCTACCCAGAGGACTACGGAGATATCGAGATCGGCTCCCACAGGAGCTCCAGCAGCAACCTGGGTGCAGACGACGGCTACATGCCCATGACGCCTGGCGCGGCCCTCGCGGGCAGTGGGAGCGGCAGCTGCAGGAGCGACGACTACATGCCCATGAGCCCTGCCAGCGTGTCCGCCCCCAAGCAGATCTTGCAGCCCAGGGCCGCCGCCGCTGCCGCCGCCACCGTGCCCCCTGCGGGGCCTGCGGGGCCAGCGCCCACCTCTGCGGCGGGCAGGACATTCCCGGCGAGCGGGGGCGGTTACAAGGCCAGCTCGCCGGCCGAGAGCTCTCCCGAGGACAGCGGGTACATGCGCATGTGGTGCGGCTCCAAGCTGTCCATGGAGCACGCGGACGGCAAGCTGCTGCTCAACGGGGACTACCTCAACGTGTCCCCCAGCGACGCGGTCACCACGGGCACCCCGCCCGACTTCTTCTCCGCAGCCCTGCACGCCGGCGGGGAGCCGCTCAGGGGCGTTCCTGGCTGCTGCTACAGCTCCTTGCCCCGCTCCTACAAGGCCCCCTACACCTGCGGTGGGGACAGCGACCAGTACGTGCTCATGAGCTCCCCCGTGGGGCGCATCCTGGAGGAGGAGCGGCTGGAGCCTCAGGCCACGCCAGGGCCCAGCCAGGCGGCCAGCGCCTTCGGGACCGGCCCCACGCAGCCCCCCCACCCCGTAGTGCCTTCGCCCGTGCGACCCAGCGGCGGCCGCCCCGAGGGCCTCCTGGGCCAGCGCGGCCGGGCTGTGCGGCCCACGCGCCTGTCCCTGGAGGGGCTGCCCAGCCTGCCCAGCATGCACGAGTACCCACTGCCGCCAGAGCCCAAGAGCCCCGGCGAGTACATCAACATCGACTTTGGCGAGCCCGGGGCCCGCCTGTCGCCGCCCGCGCCTCCCCTGCTGGCGTCAGCGGCCTCGTCCTCCTCGCTCTTGTCCGCCAGCAGCCCGGCCTCGTCGCTGGGCTCAGGCACCCCGGGCACCAGCAGCGACAGCCGGCAGCGCTCCCCGCTCTCTGACTACATGAACCTCGACTTCAGCTCCCCCAAGTCTCCCAAGCCGGGCGCCCGGAGCGGCCACCCCGTGGGCTCCTTGGACGCCCTCCTGTCCCCTGAGGCCTCCTCCCCGTATCCGCCGCTGCCCCCGCGTCCGTCCGCATCCCCGTCGTCGTCCCTGCAGCCGCCACCGCCACCACCGCCCCCCGGGGAGCTGTACCGCTTGCCCCCTGCTTCGGCCACTGCCACCGTCCAGGGCCCAGGCGCCGCCTCGTCCTCGGACACCGGAGACAATGGTGACTACACCGAGATGGCTTTCGGTGTGGCCGCCACCCCGCCGCAACCCATCGCGGCCCCCTCGAAGCCAGAAGCTGTCCGCGTGGCCAGCCCGACGTCGGGCGTGAAGAGGCTGAGCCTCATGGAGCAGGTGTCGGGGGTCGAGGCCTTCCTGCAGGCCAGCCAGCCCCCGGACCCCCACCGCGGCGCCAAGGTCATCCGCGCAGACCCACAGGGGGGCCGCCGCCGCCACAGCTCCGAGACATTCTCCTCCACCACGACGGTCACCCCCGTGTCCCCGTCGTTCGCCCACAACCCCAAGCGCCACAACTCGGCCTCCGTGGAAAATGTGTCTCTCAGGAAAAGCAGCGAGGGCGGCGTGGGCGGCGTCCCTGGAGGGGGCGACGAGCCGCCCACCTCCCCCAGACAGTTGCAGCCGGCGCCCCCCTTGGCACCGCAGGGCCGGCCTTGGACCCCGGGTCAGCCCGGGGGCTTGGTCGGTTGTCCTGGGAGCGGTGGATCACCAATGCGCAGAGAGACCTCTGCCGGCTTCCAGAATGGTCTCAACTACATCGCCATCGACGTGAGGGAGGAGCCCGGGCTGCCACCCCAGCCGCAGCCGCAGCCGCAGTCGCCGCTTCCTCAGCCGGGAGACAAGAACTCCTGGGGTCGGACCCGAAGCCTCGGGGGTCTCATCAGCGCTGTGGGCGTCGGCAGCACCGGTGGCGGGTGCGGGGGGCCAGGTCCCGGTGCCCTGCCCCCTGCCAACACCTACGCTAGCATTGACTTCTTGTCCCACCACTTGAAGGAGGCCACCATCGTGAAAGAGTGA

>Chl_sa2 [African green monkey (Chlorocebus sabaeus) Irs2]

ATGGCGAGCCCGCCGCGGCACGGGCCGCCCGGGCCGGCGAGCGGAGACGGCCCCAACCTCAACAACAACAACAACAACAACAACCACAGCGTGCGCAAGTGCGGCTACCTGCGCAAGCAGAAGCACGGCCACAAGCGCTTCTTCGTGCTGCGCGGGCCCGGCACGGGCGGCGACGAGGCGACGGCGGGAGGGGGGTCGGCGCCGCAGCCGCCGCGGCTCGAGTACTACGAGAGCGAGAAAAAGTGGCGGAGCAAGGCAGGCGCGCCGAAGCGGGTGATCGCGCTCGACTGCTGCCTGAACATCAACAAGCGCGCCGACGCCAAGCACAAGTATCTGATCGCCCTCTACACCAAGGACGAGTACTTCGCCGTGGCCGCCGAGAACGAGCAGGAGCAGGAGGGCTGGTACCGCGCGCTCACCGACCTGGTCAGCGAGGGCCGCGCGGCCGCCGCAGACGCGCCCCCCACCGCCGCGCCCGCCGCGTCCTGCAGCGCCTCCCTGCCCGGCGCCCTGGGCGGCTCTGCCGGCGCCGCCGGGGCCGAGGACAGCTACGGGCTGGTGGCGCCCGCCACGGCCGCCTACCGCGAGGTGTGGCAGGTGAACCTGAAGCCCAAGGGTCTGGGCCAGAGCAAGAACCTGACGGGCGTGTACCGTCTGTGCCTGTCGGCGCGCACCATCGGCTTCGTGAAGCTCAACTGCGAGCAGCCGTCGGTGACGTTGCAGCTCATGAACATCCGCCGCTGCGGCCACTCGGACAGCTTCTTCTTCATCGAGGTGGGCCGCTCGGCCGTCACGGGTCCCGGCGAGCTGTGGATGCAGGCGGATGACTCGGTGGTGGCGCAGAACATACACGAGACCATCCTGGAGGCCATGAAGGCGCTCAAAGAGCTCTTCGAGTTCCGGCCACGCAGCAAGAGCCAGTCGTCGGGGTCGTCGGCCACACACCCTATCAGCGTCCCCGGCGCGCGCCGCCACCACCACCTGGTCAACCTACCCCCCAGCCAGACGGGCTTGGTACGCCGTTCGCGCACCGACAGCCTGGCCGCCACGCCGCCGGCAGCCAAGTGCAGCTCGTGCCGGGTGCGCACCGCCAGCGAGGGCGACGGCGGCGCGGCGGCGGGGGCAGCGGCCGCGGGCGCCAGGCCAGTGTCGGTGGCTGGGAGCCCCCTGAGCCCCGGGCCGGTGCGCGCGCCCCTGAGCCGCTCGCACACCCTGAGCGGTGGCTGCGGCGGCCGCGGGAGCAAGGTGGCGCTGCTGCCGGCAGGGGGCGCGCTGCAGCACAGCCGCTCCATGTCCATGCCTGTGGCGCACTCGCCGCCCGCCGCCACCAGCCCCGGCTCCCTGTCGTCCAGCAGCGGCCACGGCTCGGGCTCCTACCCGCCGCCGCCGGGCCCGCACCCGCCCCTGCCGCACCCACTGCACCACGGCCCCGGCCAGCGGCCCTCCAGCGGCAGCGCCTCCGCCTCGGGCTCCCCCAGCGACCCCGGCTTCATGTCCCTGGACGAGTACGGCTCCAGCCCCGGCGACCTGCGCGCCTTCTGCAGCCACCGGAGCAACACGCCCGAGTCCATCGCGGAAACGCCCCCTGCGCGAGACGGCAGCGGCGGCGGTGAGTTCTACGGGTACATGACCATGGACAGACCCCTGAGCCACTGTGGCCGCCCCTACCGCAGGGTCTCGGGAGACGCGGCCCAGGACCTGGACCGAGGGCTGCGCAAGAGGACCTACTCCCTGACCACGCCGGCCCGGCAGCGGCCGGTGCCCCAGCCCTCCTCCGCCTCGCTGGATGAGTACACCCTGATGCGGGCCACCTTCTCGGGCAGCGCAGGCCGCCTCTGCCCGTCCTGCCCCGCGTCCTCTCCCAAGGTGGCCTACCACCCCTACCCAGAGGACTACGGAGATATCGAGATCGGCTCCCACAGGAGCTCCAGCAGCAACCTGGGTGCAGACGACGGCTACATGCCCATGACGCCTGGCGCGGCCCTCGCGGGCAGTGGGAGCGGCAGCTGCAGGAGCGACGACTACATGCCCATGAGCCCTGCCAGCGTGTCCGCCCCCAAGCAGATCTTGCAGCCCAGGGCCGCCGCCGCTGCCGCCGCCACCGTGCCCCCTGTGGGGCCTGCGGGGCCAGCGCCCACCTCTGCGGCGGGCAGGACATTCCCGGCGAGCGGAGGCGGTTACAAGGCCAGCTCGCCGGCCGAGAGCTCTCCCGAGGACAGCGGGTACATGCGCATGTGGTGCGGCTCCAAGCTGTCCATGGAGCACGCGGACGGCAAGCTGCTGCTCAACGGGGACTACCTCAACGTGTCCCCCAGTGACGCGGTCACCACGGGCACCCCGCCCGACTTCTTCGCAGCCCTGCACGCCGGCGGGGAGCCGCTCAGGGGCGTTCCTGGCTGCTGCTACAGCTCCTTGCCCCGCTCTTACAAGGCCCCCTACACCTGCGGTGGGGACAGCGACCAGTACGTGCTCATGAGCTCTCCCGTAGGGCGCATCCTGGAGGAGGAGCGGCTGGAGCCTCAGGCCACGCCAGGGCCCAGCCAGGCAGCCAGCGCCTTCGGGGCCGGCCCCACGCAGCCCCCTCACCCCGTAGTGCCTTCGCCCGTGCGACCCAGCAGCGGCCGCCCCGAGGGCTTCCTGGGCCAGCGCGGCCGGGCTGTGCGGCCCACACGCCTGTCCCTGGAGGGGCTGCCCAGCCTGCCCAGCATGCACGAGTACCCACTGCCGCCAGAGCCCAAGAGCCCCGGCGAGTACATCAACATCGACTTTGGCGAGCCCGGGGCCCGCCTGTCGCCGCCCGCGCCTCCCCTGCTGGCGTCGGCGGCCTCGTCCTCCTCGCTCTTGTCCGCCAGCAGCCCGGCCTCGTCGCTAGGCTCAGGCACCCCGGGCACCAGCAGCGACAGCCGGCAGCGCTCCCCGCTCTCTGACTACATGAACCTCGACTTCAGCTCCCCCAAGTCTCCCAAGCCAGGCGCCCGGAGCGGCCACCCCGTGGGCTCCTTGGATGCCCTCCTGTCCCCTGAGGCCTCCTCCCCATATCCGCCACTGCCCCCGCGTCCGTCCGCATCCCCGTCGTCATCCCTGCAGCCGCCACCGCCACCACCGCCCCCCGGGGAGCTGTACCGCCTGCCCCCTGCTTCGGCCGCTGCCACCGTCCAGGGCCCAGGCGCCGCCTCGTCCTCGGACACCGGAGACAATGGTGACTACACCGAGATGGCTTTCGGTGTGGCCGCCACCCCGCCGCAGCCCATCGCGGCCCCCGCGAAGCCAGAAGCTGTCCGCGTGGCCAGCCCGACGTCGGGCGTGAAGAGGCTGAGCCTCATGGAGCAGGTGTCGGGGGTCGAGGCCTTCTTGCAGGCCAGCCAGCCCCCGGACCCCCACCGCGGCGCCAAGGTCATCCGCGCAGACCCACAGGGGGGCCGCCGCCGCCACAGCTCCGAGACCTTCTCCTCCACCACGACGGTCACCCCCGTGTCCCCGTCTTTCGCCCACAACCCCAAGCGCCACAACTCGGCCTCCGTGGAAAATGTGTCTCTCAGGAAAAGCAGCGAGGGCGGCGTGGGCGGTGTCCCTGGAGGAGGCGACGAACCGCCCACCTCCCCCAGACAGTTGCAGCCGGCGCCCCCCTTGGCACCGCAGGGCCGGCCTTGGACCCCGGGTCAGCCCGGGGGCTTGGTCGGTTGTCCTGGGAGCGGTGGATCACCAATGCGCAGAGAGACCTCTGCCGGCTTCCAGAATGGTCTCAACTACATCGCCATCGACGTGAGGGAGGAGCCCGGGCTGCCACCCCAGCCGCAGCCGCAGCCGCAGTCGCCGCTTCCTCAGCCGGGAGACAAGAACTCCTGGGGTCGGACCCGAAGCCTCGGGGGTCTCATCAGCGCTGTGGGCGTCGGCAGCACCGGTGGCGGGTGCGGGGGGCCAGGTCCCGGTGCCCTGCCCCCTGCCAACACCTACGCTAGCATTGACTTCTTGTCCCACCACTTGAAGGAGGCCACCATCGTGAAAGAGTGA

>Mac_ne2 [Pig-tailed macaque (Macaca nemestrina) Irs2]

ATGGCGAGCCCGCCGCGGCACGGGCCGCCCGGGCCGGCGAGCGGAGACGGCCCCAACCTCAACAACAACAACAACAACAACAACCACAGCGTGCGCAAGTGCGGCTACCTGCGCAAGCAGAAGCACGGCCACAAGCGCTTCTTCGTGCTGCGCGGGCCCGGCACGGGCGGCGACGAGGCGACGGCGGGAGGGGGGTCGGCGCCGCAGCCGCCGCGGCTCGAGTACTACGAGAGCGAGAAAAAGTGGCGGAGCAAGGCAGGCGCGCCGAAGCGGGTGATCGCGCTCGACTGCTGCCTGAACATCAACAAGCGTGCCGACGCCAAGCACAAGTACCTGATCGCCCTCTACACCAAGGACGAGTACTTCGCCGTGGCCGCCGAGAACGAGCAGGAGCAGGAGGGCTGGTACCGCGCGCTCACCGACCTGGTCAGCGAGGGCCGCGCGGCCGCCGCAGACGCGCCCCCCACCGCCGCGCCCGCCGCGTCCTGCAGCGCCTCTCTGCCCGGCGCCCTGGGCGGCTCTGCCGGCGCCGAGGACACCTACGGGCTGGTGGCGCCCGCCACGGCCGCCTACCGCGAGGTGTGGCAGGTGAACCTGAAGCCCAAGGGTCTGGGCCAGAGCAAGAACCTGACGGGCGTTTACCGTCTGTGCCTGTCGGCGCGCACCATCGGCTTCGTGAAGCTCAACTGCGAGCAGCCGTCGGTGACGCTGCAGCTCATGAACATCCGCCGCTGCGGCCACTCGGACAGCTTCTTCTTCATCGAGGTGGGCCGCTCGGCCGTCACGGGTCCCGGCGAGCTGTGGATGCAGGCGGATGACTCGGTGGTGGCGCAGAACATACACGAGACCATCCTGGAGGCCATGAAGGCGCTCAAAGAGCTCTTCGAGTTCCGGCCACGCAGCAAGAGCCAGTCGTCGGGGTCGTCGGCCACACACCCTATCAGCGTCCCCGGCGCGCGCCGCCACCACCACCTGGTCAACCTACCCCCCAGCCAGACGGGCTTGGTGCGCCGTTCGCGTACCGACAGCCTGGCCGCCACCCCGCCGGCGGCCAAGTGCAGCTCGTGCCGGGTGCGCACCGCCAGCGAGGGCGACGGCGGCGCGGCGGCGGGGGCAGCGGCCGCGGGCGCCAGGCCAGTGTCGGTGGCTGGGAGCCCCCTGAGCCCCGGGCCAGTGCGCGCGCCCCTGAGCCGCTCGCACACCCTGAGCGGCGGCTGCGGCGGCCGCGGGAGCAAGGTGGCGCTGCTGCCGGCAGGGGGCGCGCTGCAGCACAGCCGCTCCATGTCCATGCCTGTGGCGCACTCGCCGCCCGCCGCCACCAGCCCCGGCTCCCTGTCGTCCAGCAGCGGCCACGGCTCGGGCTCCTACCCGCCGCCGCCGGGCCCGCACCCGCCCCTGCCGCACCCACTGCACCACGGCCCCGGCCAGCGGCCCTCCAGCGGCAGCGCCTCCGCCTCGGGTTCCCCCAGCGACCCCGGCTTCATGTCCCTGGACGAGTACGGCTCCAGCCCCGGCGACCTGCGCGCCTTCTGCAGCCACCGGAGCAACACGCCCGAGTCCATCGCGGAAACGCCCCCTGCGCGAGACGGCAGCGGCGGTGGTGAGTTCTACGGGTACATGACCATGGACAGGCCCCTGAGCCACTGTGGCCGCCCCTACCGCAGGGTCTCGGGAGACGCGGCCCAGGACCTGGACCGAGGGCTGCGCAAGAGGACCTACTCCCTGACCACGCCGGCCCGGCAGCGGCCGGTGCCCCAGCCCTCCTCCGCCTCGCTGGATGAGTACACCCTGATGCGGGCCACCTTCTCCGGCAGCGCAGGCCGCCTCTGCCCGTCCTGCCCCGCGTCCTCTCCCAAGGTGGCCTACCACCCCTACCCAGAGGACTACGGAGATATCGAGATCGGCTCCCACAGGAGCTCCAGCAGCAACCTGGGTGCAGACGACGGCTACATGCCCATGACGCCTGGCGCGGCCCTCGCGGGCAGTGGGAGCGGCAGCTGCAGGAGCGACGACTACATGCCCATGAGCCCTGCCAGCGTGTCCGCCCCCAAGCAGATCTTGCAGCCCAGGGCCGCCGCCGCTGCCGCCGCCACCGTGCCCCCTGCGGGGCCTGCGGGGCCAGCGCCCACCTCTGCGGCGGGCAGGACATTCCCGGCGAGCGGGGGCGGTTACAAGGCCAGCTCGCCGGCCGAGAGCTCTCCCGAGGACAGCGGGTACATGCGCATGTGGTGCGGCTCCAAGCTGTCCATGGAGCACGCGGACGGCAAGCTGCTGCTCAACGGGGACTACCTCAACGTGTCCCCCAGCGACGCGGTCACCACGGGCACCCCGCCCGACTTCTTCTCCGCAGCTCTGCACGCCGGCGGGGAGCCGCTCAGGGGCGTTCCTGGCTGCTGCTACAGCTCCTTGCCCCGCTCCTACAAGGCCCCCTACACCTGCGGTGGGGACAGCGACCAGTACGTGCTCATGAGCTCCCCCGTGGGGCGCATCCTGGAGGAGGAGCGGCTGGAGCCTCAGGCCACGCCAGGGCCCAGCCAGGCGGCCAGCGCCTTCGGGGCCGGCCCCACGCAGCCCCCGCACCCCGTAGTGCCTTCGCCCGTGCGACCCAGCGGCGGCCGCCCCGAGGGCCTCCTGGGCCAGCGCGGCCGGGCTGTGCGGCCCACGCGCCTGTCCCTGGAGGGGCTGCCCAGCCTGCCCAGCATGCACGAGTACCCACTGCCGCCAGAGCCCAAGAGCCCCGGCGAGTACATCAACATCGACTTTGGCGAGCCCGGTGCCCGCCTGTCGCCGCCCGCGCCTCCCCTGCTGGCGTCGGCGGCCTCGTCCTCCTCGCTCTTGTCCGCCAGCAGCCCGGCCTCGTCGCTGGGCTCAGGCACCCCGGGCACCAGCAGCGACAGCCGGCAGCGCTCCCCGCTCTCTGACTACATGAACCTCGACTTCAGCTCCCCCAAGTCTCCCAAGCCGGGCGCCCGGAGCGGCCACCCCGTGGGCTCCTTGGACGCCCTCCTGTCCCCTGAGGCCTCCTCCCCGTATCCGCCGCTGCCCCCGCGTCCGTCCGCATCCCCGTCGTCGTCCCTGCAGCCGCCACCGCCACCACCGCCCCCCGGGGAGCTGTACCGCTTGCCCCCTGCTTCGGCCACTGCCACCGTCCAGGGCCCAGGCGCCGCCTCGTCCTCGGACACCGGAGACAATGGTGACTACACCGAGATGGCTTTCGGTGTGGCCGCCACCCCGCCGCAACCCATCGCGGCCCCCGCGAAGCCAGAAGCTGTCCGCGTGGCCAGCCCGACGTCGGGCGTGAAGAGGCTGAGCCTCATGGAGCAGGTGTCGGGGGTCGAGGCCTTCCTGCAGGCCAGCCAGCCCCCGGACCCCCACCGCGGCGCCAAGGTCATCCGCGCAGACCCACAGGGGGGCCGCCGCCGCCACAGCTCCGAGACCTTCTCCTCCACCACGACGGTCACCCCCGTGTCCCCGTCTTTCGCCCACAACCCCAAGCGCCACAACTCGGCCTCCGTGGAAAATGTGTCTCTCAGGAAAAGCAGCGAGGGCGGCGTGGGCGGCGTCCCTGGAGGGGGCGACGAGCCGCCCACCTCCCCCAGACAGTTGCAGCCGGCGCCCCCCTTGGCACCGCAGGGCCGGCCTTGGACCCCGGGTCAGCCCGGGGGCTTGGTCGGTTGTCCTGGGAGCGGTGGGTCACCAATGCGCAGAGAGACCTCTGCCGGCTTCCAGAATGGTCTCAACTACATCGCCATCGACGTGAGGGAGGAGCCCGGGCTGCCACCCCAGCCGCAGCCGCAGCCGCAGTCGCCGCTTCCTCAGCCGGGAGACAAGAACTCCTGGGGTCGGACCCGAAGCCTCGGGGGTCTCATCAGCGCTGTGGGCGTCGGCAGCACCGGTGGCGGGTGCGGGGGGCCAGGTCCCGGTGCCCTGCCCCCTGCCAACACCTACGCTAGCATTGACTTCTTGTCCCACCACTTGAAGGAGGCCACCATCGTGAAAGAGTGA

>Cer_at2 [Sooty mangabey (Cercocebus atys) Irs2]

ATGGCGAGCCCGCCGCGGCACGGGCCGCCCGGGCCGGCGAGCGGAGACGGCCCCAACCTCAACAACAACAACAACAACAACAACCACAGCGTGCGCAAGTGCGGCTACCTGCGCAAGCAGAAGCACGGCCACAAGCGCTTCTTCGTGCTGCGCGGGCCCGGCACGGGCGGCGACGAGGCGACGGCGGGAGGGGGGTCGGCGCCGCAGCCGCCGCGGCTCGAGTACTACGAGAGCGAGAAAAAGTGGCGGAGCAAGGCAGGCGCGCCGAAGCGGGTGATCGCGCTCGACTGCTGCCTGAACATCAACAAGCGCGCCGACGCCAAGCACAAGTACCTGATCGCCCTCTACACCAAGGACGAGTACTTCGCCGTGGCCGCCGAGAACGAGCAGGAGCAGGAGGGCTGGTACCGCGCGCTCACCGACCTGGTCAGCGAGGGCCGCGCGGCCGCCGCAGACGCGCCCCCCACCGCCGCGCCCGCCGCGTCCTGCAGCGCCTCCCTGCCCGGCGCCCTGGGCGGCTCTGCCGGCGCCGCCGGGGCCGAGGACAGCTACGGGCTGGTGGCGCCCGCCACGGCCGCCTACCGCGAGGTGTGGCAGGTGAACCTGAAGCCCAAGGGTCTGGGCCAGAGCAAGAACCTGACGGGCGTGTACCGTCTGTGCCTGTCGGCGCGCACCATCGGCTTCGTGAAGCTCAACTGCGAGCAGCCGTCGGTGACGCTGCAGCTCATGAACATCCGCCGCTGCGGCCACTCGGACAGCTTCTTCTTCATCGAGGTGGGCCGCTCGGCCGTCACGGGTCCCGGCGAGCTGTGGATGCAGGCGGATGACTCGGTGGTGGCGCAGAACATACACGAGACCATCCTGGAGGCCATGAAGGCGCTCAAAGAGCTCTTCGAGTTCCGGCCACGCAGCAAGAGCCAGTCGTCGGGGTCGTCGGCCACACACCCTATCAGCGTCCCCGGCGCGCGCCGCCACCACCACCTGGTCAACCTACCCCCCAGCCAGACGGGCTTGGTGCGCCGTTCGCGCACCGACAGCCTGGCCGCCACCCCGCCGGCGGCCAAGTGCAGCTCGTGCCGGGTGCGCACCGCCAGCGAGGGCGACGGCGGCGCGGCGGCGGGGGCAGCGGCCGCGGGCGCCAGGCCAGTGTCGGTGGCTGGGAGCCCCTTGAGCCCCGGGCCGGTGCGCGCGCCCCTGAGCCGCTCGCACACCCTGAGCGGCGGCTGCGGCGGCCGCGGGAGCAAGGTGGCGCTGCTGCCGGCAGGGGGCGCGCTGCAGCACAGCCGCTCCATGTCCATGCCTGTGGCGCACTCGCCGCCCGCCGCCACCAGCCCCGGCTCCCTGTCGTCCAGCAGCGGCCACGGCTCGGGCTCCTACCCGCCGCCGCCGGGCCCGCACCCGCCCCTGCCGCACCCACTGCACCACGGCCCCGGCCAGCGGCCCTCCAGCGGCAGCGCCTCCGCCTCGGGCTCCCCCAGCGACCCCGGCTTCATGTCCCTGGACGAGTACGGCTCCAGCCCCGGCGACCTGCGCGCCTTCTGCAGCCACCGGAGCAACACGCCCGAGTCCATCGCGGAAACGCCCCCTGCGCGAGACGGCAGCGGCGGCGGTGAGTTCTACGGGTACATGACCATGGACAGGCCCCTGAGCCACTGTGGCCGCCCCTACCGCAGGGTCTCGGGAGACGCGGCCCAGGACCTGGACCGAGGGCTGCGCAAGAGGACCTACTCCCTGACCACGCCGGCCCGGCAGCGGCCGGTGCCCCAGCCCTCCTCCGCCTCGCTGGATGAGTACACCCTGATGCGGGCCACCTTCTCCGGCAGCGCAGGCCGCCTCTGCCCGTCCTGCCCCGCGTCCTCTCCCAAGGTGGCCTACCACCCCTACCCAGAGGACTACGGAGATATCGAGATCGGCTCCCACAGGAGCTCCAGCAGCAACCTGGGTGCAGACGACGGCTACATGCCCATGACGCCTGGCGCGGCCCTCGCGGGCAGTGGGAGCGGCAGCTGCAGGAGCGACGACTACATGCCCATGAGCCCTGCCAGCGTGTCCGCCCCCAAGCAGATCTTGCAGCCCAGGGCCGCCGCCGCTGCCGCCGCCACCGTGCCCCCTGCGGGGCCTGCGGGGCCAGCGCCCACCTCTGCGGCGGGCAGGACATTCCCGGCGAGCGGGGGCGGTTACAAGGCCAGCTCGCCGGCCGAGAGCTCTCCCGAGGACAGCGGGTACATGCGCATGTGGTGCGGCTCCAAGCTGTCCATGGAGCACGCGGACGGCAAGCTGCTGCTCAACGGGGACTACCTCAACGTGTCCCCCAGCGACGCGGTCACCACCGGCACCCCGCCCGACTTCTTCTCCGCAGCCCTGCACGCCGGCGGGGAGCCGCTCAGGGGCGTTCCTGGCTGCTGCTACAGCTCCTTGCCCCGCTCCTACAAGGCCCCCTACACCTGCGGTGGGGACAGCGACCAGTACGTGCTCATGAGCTCCCCCGTGGGGCGCATCCTGGAGGAAGAGCGGCTGGAGCCTCAGGCCACGCCAGGGCCCAGCCAGGCGGCCAGCGCCTTCGGGACAGGCCCCACGCAGCCCCCTCACCCCGTAGTGCCTTCGCCCGTGCGACCCAGTGGCGGCCGCCCCGAGGGTTTCCTGGGCCAGCGCGGCCGGGCTGTGCGGCCCACACGCCTGTCCCTGGAGGGGCTGCCCAGCCTGCCCAGCATGCACGAGTACCCACTGCCGCCAGAGCCCAAGAGCCCCGGCGAGTACATCAACATCGACTTTGGCGAGCCCGGGGCCCGCCTGTCGCCGCCCGCGCCTCCCCTGCTGGCGTCGGCGGCCTCGTCCTCCTCGCTCTTGTCCGCCAGCAGCCCGGCCTCGTCGTTGGGCTCAGGCACCCCGGGCACCAGCAGCGACAGCCGGCAGCGCTCCCCGCTCTCTGACTACATGAACCTCGACTTCAGCTCCCCCAAGTCTCCCAAGCCGGGCGCCCGGAGCGGCCACCCCGTGGGCTCCTTGGACGCCCTCCTGTCCCCTGAGGCCTCCTCCCCGTATCCGCCGCTGCCCCCGCGTCCGTCCGCATCCCCGTCGTCGTCGTCCCTGCAGCCGCCACCGCCACCACCGCCCCCCGGGGAGCTGTACCGCTTGCCCCCTGCTTCGGCCGCTGCCACCGTCCAGGGCCCAGGCGCCGCCTCGTCCTCGGACACCGGAGACAATGGTGACTACACCGAGATGGCTTTCGGTGTGGCCGCCACCCCGCCGCAACCCATCGCGGCCCCCGCGAAGCCAGAAGCTGTCCGCGTGGCCAGCCCGACGTCGGGCGTGAAGAGGCTGAGCCTCATGGAGCAGGTGTCGGGGGTCGAGGCCTTCCTGCAGGCCAGCCAGCCCCCGGACCCCCACCGCGGCGCCAAGGTCATCCGCGCAGACCCACAGGGGGGCCGCCGCCGCCACAGCTCCGAGACCTTCTCCTCCACCACGACGGTCACCCCCGTGTCCCCGTCTTTCGCCCACAACCCCAAGCGCCACAACTCGGCCTCCGTGGAAAATGTGTCTCTCAGGAAAAGCAGCGAGGGCGGCGTGGGCGGCGTCCCTGGAGGGGGCGACGAGCCGCCCACCTCCCCCAGACAGTTGCAGCCGGCGCCCCCCTTGGCACCGCAGGGCCGGCCTTGGACCCCGGGTCAGCCCGGGGGCTTGGTCGGTTGTCCTGGGAGCGGTGGATCACCAATGCGCAGAGAGACCTCTGCCGGCTTCCAGAATGGTCTCAACTACATCGCCATCGACGTGAGGGAGGAGCCCGGGCTGCCACCCCAGCCGCAGCCGCAGCCGCAGTCGCCGCTTCCTCAGCCGGGAGACAAGAACTCCTGGGGTCGGACCCGAAGCCTCGGGGGTCTCATCAGCGCTGTGGGCGTCGGCAGCACCGGTGGCGGGTGCGGGGGGCCAGGTCCCGGGGCCCTGCCCCCTGCCAACACCTACGCTAGCATTGACTTCTTGTCCCACCACTTGAAGGAGGCCACCATCGTGAAAGAGTGA

>Mic_mu2 [Mouse lemur (Microcebus murinus) Irs2]

ATGGCCAGCCCGCCGCTGCACGGGCCCCCGGGGCCGGCGAGCGGAGACGGCCCCAACCTCAACAACAACAACAACAACAACCACGGCGTGCGCAAGTGCGGCTACCTGCGCAAGCAGAAGCACGGCCACAAGCGCTTCTTCGTGCTGCGCGGGCCTGGCGCGGGCAGCGACGAAGCGACAGCGACAGCGGGCGGGGGGTCGGCGCCGCAGCCGCCGCGGCTCGAGTACTATGAGAGCGAGAAGAAGTGGCGGAGCAAGGCGGGCGCCCCGAAGCGGGTGATCGCGCTCGACTGCTGCCTGAACATCAACAAGCGCGCCGACGCCAAGCACAAGTACCTGATCGCCCTCTACACCAAGGACGAGTACTTCGCGGTGGCAGCAGAGAACGAGCAGGAGCAGGAGGGCTGGTATCGCGCGCTCACCGACTTGGTCAGCGAGGGCCGCGCGGGCGCCGGCGACGCGCCCTCCAACGCCGCGGCCACCTCCGGGTCCTGCAGCGCCTCCTTGCCGGGCGCCCTGGGCGGCTCGGCCGGCGCCGCCGGGGCGGATGACAGCTACGGGCTGGTGTCTCCCGCCACGGCCGCCTACCGCGAGGTGTGGCAGGTGAACCTGAAGCCCAAGGGTCTGGGCCAGAGCAAGAACCTGACGGGCGTGTACCGCCTGTGCCTGTCGGCGCGCACCATAGGCTTCGTGAAGCTCAACTGCGAGCAGCCGTCGGTGACGCTGCAGCTCATGAACATCCGCCGCTGCGGCCACTCGGACAGCTTCTTCTTTATTGAGGTGGGCCGCTCGGCGGTCACCGGCCCCGGTGAGCTGTGGATGCAGGCCGACGACTCGGTGGTGGCGCAGAACATCCACGAGACCATCCTGGAGGCCATGAAGGCGCTCAAGGAGCTCTTCGAGTTCCGGCCGCGCAGCAAGAGCCAGTCGTCAGGGTCGTCAGCCACGCACCCCATCAGTGTCCCCGGCGCGCGGCGCCATCACCACCTGGTCAACCTGCCCCCGAGTCAGACTGGCCTGGTGCGCCGCTCCCGCACCGACAGCCTGGCCGCCACCCCGCCGGCCGCCAAGTGCAACTCCTGTCGAGTGCGCACCGCCAGCGAGGGCGACGGTGGCGCGGCAGCGGGGACCGGGGCTGCGGGCGGCAGGCCGGTGTCGGTGGCGGGGAGCCCCCTGAGCCCCGGGCCGGTGCGCGCGCCCCTGAGCCGCTCGCACACCCTGAGCGGCGGCTGCGGTGGCCGCGGGAGCAAAGTGACGCTGGCGCCGGCAGGGGGCGCCCTGCAACACAGCCGCTCCATGTCCATGCCGGTGGCGCACTCGCCCCCGACTGCCACCAGCCCCGGCAGCCTGTCGTCCAGCAGCGGGCACGGCTCAGGCTCCTACCCGCCGCCTCCGGGACCCCACCCGCACCTGCCACACCCCCTGCACCACCCCCCAGGCCAGCGGCCCTCCAGCGGGAGCGCCTCAGCCTCCGGCTCCCCCAGCGACCCCGGCTTCATGTCCCTGGACGAGTACGGCTCCAGCCCTGGCGACCTGAGAGCCTTCTGCAGCCACAGGAGCAACACGCCCGAGTCCATCGCGGAGACACCCCCGACCAGGGACGGCGGCGGGGGCGAGCTGTATGGGTACATGACCATGGACAGGCCCCTGAGCCACTGCGGCCGCCCCTACCGCAGGGTCTCAGGGGACGGCGTGCAGGACTTGGACAGAGGACTGAGGAAGAGGACTTACTCGCTGACCACACCTGCCCGGCAGCGGCCCGTGCCGCAGCCCTCCTCAGCCTCCTTGGATGAATACACCCTGATGCGGGCCACCTTCTCCGGCAGCTCAGGCCGCCTGTGCCCGTCGTGCCCTGCGTCTTCTCCCAAAGTGACCTACCACCCCTACCCGGAGGACTACGGGGACATCGAGATTGGATCCCACAGGAGCTCCAGCAGCAACCTGGGCGCTGACGACGGCTACATGCCCATGACCCCTGGCGTGGCCCTCATGGGCAGTGGCGGGGGCAGCTGCAAGAGCGACGATTACATGCCCATGAGCCCCACCAGTGTGTCTGCCCCAAAGCAGATCCTGCAGCCCAGGGCGGCCACTGCAGCCTTGCCTCCCGCGGGAGCCGCCGCGCCAGCGCCCGCCGCTGCAGCCAGCAGGACCTTTGCGGGGACCGGGGGCGGCTACAAGACCAGCTCCCCCGCGGAGAGCTCCCCCGAGGACAGTGGGTACATGCGGATGTGGTGTGGTTCCAAGCTGTCCATGGAGAATGCCGACAGCAAGCTGCTTCCCAACGGGGACTACCTCAACATGTCCCCCAGCGACGTGGGCACCTCCGGTACTCCACCCGACTTCTTCTCTGCAGCTTTGCACGCCGGGGGCGAGATGCTCAAAGGCGTCCCAGGCTACTGTTACAGCTCTCTGCCCCGCTCCTACAAGGCGCCATACACCTGCGGCGGGGGCAACGACCAGTATGTGCTCATGAGCGCCCCCGTGGGGCGGATCCTGGAAGAGGAGAGGCTGGAGCCACAGGCCACCCTGGGGACCACTCCATCGGCCAGCGCCTTTGGGGTCGGTGGGGGTGGCCACACCCAGCCTCATCACCCAGTGCCTTCACCCGTGAGGCCGAGTGGCAGTGGCCGTCCAGAGGGCTTCCTGAGCCAGCGCTGTCGAGCCGTGAGGCCCACGCGGCTGTCCTTGGAGGGGCTGCAGACCCTGCCCAGCATGCACGAGTACCCTTTGCCGCCAGAGCCCAAGAGCCCCGGCGAGTACATCAACATTGACTTTGGCGAGGCGGGAGCCCGCCTGTCGCCGCCTGCCCCTCCGCTGCTGGCCTCGGCGGCCTCATCCTGCTCGCTGCTGTCCGCTGGCAGCCCAGCCTCGTCCCTGGGCTCGGGCACGCCGGGCACCAGCAGTGACAGCCGGCAGCGCTCCCCGCTCTCCGACTACATGAACCTTGATTTCAGCTCGCCCAAGTCACCCAAGCCTGGCACCCAGAGCGGGGACCCCGTGGGCTCCTTGGACGCCCTCCTCTCCCCTGAGGCCTCCTCTCCATACCCACCGCTGCCCCCGCGCCCTCCGGTCTCTCCAGCGTCCCTGCAGCAGCCGGCCCCCCCGCCACCCCCAGGGGAGCTGTACCGCCTGCCTCCAGCTTCGGCTGTGGCCACCACCCAGGGCCCTGGCGCTGCCTCGTCATTGTCCTCCGAGAATGGGGACAATGGTGACTACACCGAGATGGCCTTTGGCGTGGCTGCCACCCCGCCACAACCTATCGCGGCTCCCCCAAAGCCCGAGGGTGCCCGCGTGACCAGCCCTACGTCCGGCGTGAAGAGGCTGAGTCTTATGGATCAGGTGTCGGGGGTCGAGGCCTTCCTGCAGGCCAGCCAGCCCCCAGACCCACACCGGGGCGCCAAGGTCATCCGCGCAGACCCACAGGGGGGGCGCCGCCGCCACAGCTCGGAAACTTTCTCCTCCACTACAACCGTCACCCCCGTGTCCCCATCCTTCGCCCACAACCCCAAGCGCCACAACTCGGCCTCAGTGGAAAACGTGTCTCTCAGGAAAAGCAGCGAGGGCAGTGGCAGCGTCCTCAGTGGGGGCCCAGGAGGCGGTGAAGAGGCACCCACGTCCCCCAGACAGCTACAGCCGTCACTCCCGGTGCCCGCTCTCCCGCCGCAGGCCCGCCCTCGGGCCCCAGCTCAGCCAGGGGGCTTGGTCGGCTGCCCTGGGGGCAGTGGTTCTCCAATGCGCAGAGAGACCTCTGCCGGCTTCCAGAATGGCCTCAACTACATCGCCATCGACGTGAGGGACGAGCCGGGCCTGACCCAGCCGCAGCCGCAGCCCGGAGACAAGAGCTCCTGGGGGCGGACCCGTAGTCTTGGAGGCCTCATCAGCACTGTGGGCGGTGGCGGCAGCGGGGGCTGCGGGGGGCCGGGCCCTGGCGCCCTGCCCTCTGCCAGCACCTACGCCAGCATTGACTTCTTGTCCCATCATTTGAAGGAGGCCACGATTGTGAAAGAGTGA

>Pro_co2 [Coquerel's sifaka (Propithecus coquereli) Irs2]

ATGGCCAGCCCGCCGCTGCACGGGCCCCCGGGGCCGGCGAGTGGAGACGGCCCCAATCTCAACAACAACAACAACAACAACCACGGCGTGCGCAAGTGCGGCTACCTGCGCAAGCAGAAGCACGGCCACAAGCGCTTCTTCGTGCTCCGCGGGCCCGGCGCGGGCAGCGATGAAGCGACAGCGGGCGGGGGGTCGGCGCCGCAGCCGCCGCGGCTCGAGTACTATGAGAGCGAGAAGAAGTGGCGGAGCAAGGCGGGCGCCCCGAAGCGGGTGATCGCGCTCGACTGCTGCCTGAACATCAACAAGCGCGCCGACGCCAAGCACAAGTACCTGATCGCCCTCTACACCAAGGACGAGTACTTCGCGGTGGCGGCAGAGAACGAGCAGGAGCAGGAGGGCTGGTATCGCGCGCTTACCGACTTGGTCAGCGAGGGCCGGTCGGGCGCCGGCGACGCGCCCCCCAACGCCGCGGCCACCTCCGGGTCCTGCAGCGCCTCTTTGCCGGGTGCCCTGGGCGGCTCGGCCGGCGCTGCCGGGGCCGATGACAGCTACGGGCTGGTGTCTCCCGCCACGGCCGCCTACCGCGAGGTGTGGCAGGTGAACCTGAAGCCCAAGGGTCTGGGCCAGAGCAAGAACCTGACGGGCGTGTACCGCCTGTGCCTGTCGGCGCGCACCATAGGCTTCGTGAAGCTCAACTGCGAGCAGCCGTCGGTGACGCTGCAGCTCATGAACATCCGCCGCTGCGGCCACTCGGACAGCTTCTTCTTCATCGAGGTGGGTCGCTCGGCGGTCACTGGCCCCGGCGAGCTGTGGATGCAGGCTGACGACTCGGTGGTGGCGCAGAACATCCATGAGACCATCCTGGAGGCCATGAAGGCGCTCAAGGAGCTCTTCGAGTTCCGTCCGCGCAGCAAGAGCCAGTCGTCAGGGTCGTCGGCCACGCACCCCATCAGTGTCCCAGGCGCGCGGCGCCACCACCACCTGGTCAACCTGCCCCCCAGTCAGACTGGCCTGGTGCGCCGCTCCCGCACCGACAGTCTGGCCGCCACCCCACCGGCTGCCAAGTGCAGCTCATGTCGAGTGCGCACAGCCAGCGAGGGCGACGGTGGCGCGGCAGCGGGGACCGGGGCTGGGGGCGGCAGGCCGGTGTCGGTGGCCGGGAGCCCCCTGAGCCCCGGGCCGGTGCGCGCGCCCCTGAGCCGCTCGCACACCCTGAGCGGCGGCTGCGGTGGCCGCGGGAGCAAAGTGACGCTGGCACCGGCAGGGGGCGCCCTGCAACACAGCCGCTCCATGTCAATGCCGGTGGCGCACTCGCCTCCGACCGCCACCAGCCCCGGCAGCCTGTCGTCCAGCAGCGGGCACGGCTCGGGCTCCTACCCGCCGCCTCCGGGCCCCCACCCGCACCTGCCGCACCCCCTGCACCACCCTCCAGGCCAGCGGCCCTCCAGCGGCAGTGCCTCAGCCTCCGGCTCCCCCAGCGACCCCGGCTTCATGTCCCTGGACGAGTACGGCTCCAGCCCTGGTGACCTGAGAGCCTTCTGTAGCCACAGGAGCAACACGCCCGAGTCCATTGCGGAGACCCCCCCGGCCAGGGACAGCAGCGGGGGCGAGCTGTATGGGTACATGACCATGGACAGGCCCCTGAGCCACTGTGGCCGCCCCTACCGCAGAGTCTCAGGAGAGGGGGTCCAGGACTTGGACAGAGGGCTGAGGAAGAGGACTTACTCCCTGACCACACCTGCCCGGCAGCGGCCGGTGCCCCAGCCCTCCTCGGCCTCCTTGGATGAATACACCCTGATGCGGGCCACCTTCTCAGGCAGCTCAGGCCGCCTGTGCCCATCGTGCCCCGCATCTTCTCCCAAAGTGACTTACCACCCCTACCCGGAGGACTACGGGGACATCGAGATTGGATCCCACAGGAGCTCCAGTAGTAATCTGGGGGCGGACGACGGCTACATGCCCATGACCCCTGGCGTGGCCCTCATGGGCAGTGGCGGTGGCAGCTGCAAGAGCGACGATTACATGCCCATGAGCCCCACCAGCGTGTCTGCCCCAAAGCAGATCCTGCAGCCCAGGGCGGCCACTGCAGCCTTGCCTCCCACAGGAGCCGCCATGCCAGCGCCCACCTCTGCGGCCAGCAGGACCTTTGCAGGGAACGGCGGCAGCTACAAGACCAGCTCCCCCGCGGAGAGCTCCCCTGAGGACAGTGGGTACATGCGGATGTGGTGTGGTTCCAAGCTGTCCATGGAGAATGCCGACAGCAAGCTGCTTCCCAACGGGGACTACCTCAACATGTCCCCCAGCGACGCGGGCACCTCCGGTACTCCACCCGACTTCTTCTCTGCAGCCTTGCACGCTGGGGGCGAGATGCTCAAAGGCGTCCCTGGCTACTGCTACAGCTCTCTGCCCCGCTCCTACAAGGCCCCATACACCTGTGGCGGGGGCAACGACCAGTATGTGCTCATGAGCGCCCCCGTGGGGCGGATCCTGGAAGAGGAGAGGCTGGAGCCGCAGGCCACCCCAGGGAGCACTCCATCGGCCAGCGCCTTTGGGGCTAGTGGGGGTGGCCACACCCAGCCTCATCACCCAGTAGTGCCTTCGCCCGTCAGGCCGAGTGGCAGCGGCCGCCCAGAGGGCTTCCTGAGCCAGCGCTGTCGGGCCGTGAGGCCCACGCGGCTGTCTCTGGAGGGGCTGCAGACCCTGCCCAGCATGCAGGAGTACCCGCTGCCGCCAGAGCCCAAAAGCCCCGGCGAGTACATCAACATTGACTTTGGCGAGGCGGGAGCCCGCCTGTCGCCGCCTGCCCCCCCGCTGTTGGCGTCGGCGGCCTCGTCCTGCTCGCTGCTGTCTGCTGGCAGCCCAGCCTCGTCCCTGGGCTCCGGCACGCCGGGCACCAGCAGTGACAGCCGGCAGCGCTCCCCGCTCTCTGACTACATGAACCTGGACTTCAGCTCGCCCAAGTCACCCAAGCCGGGCACCCAGAGTGCGGACCCCGTGGGCTCCTTGGACGCCCTCCTCTCCCCCGAGGCCTCTTCTCCATACCCGCCGCTGCCCCCGCGCCCTCCCGCCTCGACAGCGTCCTTGCAGCAGCCGCCGCCCCCGCCACCCCCAGGGGAGCTGTACCGCCTGCCTCCGGCTTCAGCTGTGGCCGCCACCCAGGGCCCTGGCGCTGCCTCGTCGTTGTCCTCCGAGACTGGGGACAATGGTGACTACACCGAGATGGCCTTTGGCGTGGCTGCCACCCCGCCACAACCTATCGCGGCCCCCCCAAAGCCGGAAGGTGCCCGAGTGATCAGCCCTACGTCTGGCGTGAAGAGGCTGAGTCTTATGGATCAGGTGTCGGGGGTCGAGGCTTTCCTGCAGGCCAGCCAGCCCCCAGACCCACACCGGGGCGCCAAGGTCATCCGCGCAGACCCACAAGGGGGCCGCCGCCGCCACAGCTCGGAGACCTTCTCCTCGACTACGACCGTCACCCCCGTGTCCCCATCCTTCGCCCACAACCCTAAGCGCCACAACTCGGCCTCAGTGGAAAACGTCTCTCTCAGGAAAAGCAGCGAGGGCAGTGGCGGCGTCCTTGGTGGGGGCCCAGGAGGCGGCGAAGAGGCACCCATGTCCCCCAGCAAGCTGCAGCCGTCACTCCCGGTGCCCTCTCTCCCACCGCAGGCCCGCCCACGGGCCCCAGCTCAGCCAGGGGGCTTGGTCGGCTGCCCCGGGGGCAGTGGTTCTCCAATGCGCAGAGAGACTTCTGCCGGCTTCCAGAATGGTCTCAACTACATTGCCATCGACGTGAGGGATGAGCCGGGGCTGAGCCAGCCGCAACCGCAGCCAGGAGAGAAGAGTTCCTGGGGGCGGACCCGTAGCCTTGGGGGTCTCATCAGCGCTGTGGGCGGTGGCAGCAGCGGGGTGTGTGGGGGGCCAGGCCCTGGCGCCCTGCCCTCTGCCAACACCTACGCCAGCATTGACTTCTTGTCCCATCACTTGAAGGAGGCCACGATTGTGAAAGAGTGA

>Mus_mu2 [Mouse (Mus musculus) Irs2]

ATGGCTAGCGCGCCCCTGCCTGGGCCCCCCGCGTCGGCGGGCGGGGACGGCCCGAACCTCAATAACAACAACAACAACAACAACCACAGCGTGCGCAAGTGCGGCTACCTGCGCAAGCAGAAGCACGGCCACAAGCGCTTTTTCGTGTTGCGCGGCCCCGGCACGGGCGGCGACGAGGCATCCGCGGCTGGGGGGTCGCCGCCGCAGCCTCCGCGGCTGGAGTACTACGAGAGCGAGAAGAAGTGGAGGAGCAAGGCGGGCGCGCCGAAGCGAGTGATCGCGCTCGACTGCTGTCTGAACATCAACAAGCGCGCGGACGCCAAGCACAAGTACCTGATCGCCCTCTACACCAAGGACGAGTACTTCGCTGTAGCGGCGGAGAACGAGCAGGAGCAGGAGGGCTGGTACCGCGCACTCACCGACTTGGTCAGCGAAGGCCGCTCTGGCGAGGGGGGCTCGGGCACCACCGGAGGCTCTTGCAGCGCCTCTCTCCCGGGCGTCCTGGGCGGCTCAGCGGGCGCCGCTGGCTGCGATGACAACTACGGGCTCGTGACACCCGCCACGGCCGTCTACCGCGAGGTGTGGCAGGTGAACCTGAAACCTAAGGGACTGGGCCAGAGCAAGAACCTGACTGGTGTATACCGCCTATGCCTGTCTGCGCGCACCATCGGCTTCGTGAAGCTCAATTGCGAACAGCCGTCGGTGACGCTGCAGCTTATGAACATTCGCCGCTGCGGCCACTCGGACAGCTTCTTCTTCATCGAGGTGGGCCGTTCGGCCGTCACCGGTCCCGGGGAGCTGTGGATGCAAGCCGACGACTCGGTGGTGGCGCAGAACATCCATGAGACCATCCTAGAAGCTATGAAGGCACTCAAAGAGCTCTTCGAGTTCCGGCCTCGCAGCAAGAGTCAGTCGTCCGGGTCGTCAGCCACGCATCCCATCAGCGTGCCGGGCGCGCGCCGCCACCACCACCTAGTCAACCTACCCCCTAGCCAGACCGGCCTGGTGCGCCGCTCGCGCACTGACAGCCTGGCGGCCACCCCCCCAGCAGCCAAGTGCACTTCGTGCCGGGTTCGTACGGCCAGCGAGGGCGACGGCGGCGCGGCAGGCGGGGCCGGGACGGCAGGAGGCAGGCCGATGTCGGTGGCAGGGAGCCCCCTGAGTCCCGGGCCGGTGCGCGCGCCCCTTAGCCGCTCGCACACCCTGAGCGCCGGCTGCGGAGGCCGCCCGAGCAAAGTGACTCTGGCGCCGGCAGGGGGAGCCCTGCAACACAGCCGCTCCATGTCCATGCCCGTGGCGCACTCACCTCCTGCAGCCACCAGCCCAGGCAGCCTGTCCTCCAGCAGTGGGCACGGCTCGGGCTCCTACCCGCTGCCACCTGGCTCCCACCCGCACCTGCCTCATCCACTGCATCACCCCCAAGGCCAGCGTCCGTCCAGCGGTAGTGCCTCCGCCTCGGGCTCCCCCAGCGACCCGGGTTTCATGTCCCTTGACGAGTATGGCTCCAGCCCTGGCGACCTGAGAGCCTTCAGTAGCCACAGGAGCAACACACCCGAGTCAATAGCGGAGACCCCGCCAGCCAGAGATGGCAGTGGGGGCGAACTCTATGGGTACATGAGCATGGATAGACCCCTGAGCCACTGTGGCCGCCCTTACCGTAGGGTCTCAGGGGATGGGGCCCAGGACCTGGATAGAGGACTGAGGAAGAGGACTTATTCCCTAACCACGCCTGCCAGGCAGCGGCAGGTACCTCAGCCTTCCTCTGCCTCTCTAGATGAATACACTCTCATGAGGGCCACCTTCTCTGGTAGTTCAGGTCGCCTCTGCCCATCCTTCCCTGCGTCCTCTCCCAAAGTGGCCTACAACCCTTACCCAGAGGACTATGGAGACATTGAGATTGGTTCTCACAAGAGTTCCAGCAGTAACCTGGGGGCAGATGATGGCTACATGCCCATGACCCCTGGGGCAGCCCTTAGGAGTGGTGGTCCCAATAGCTGCAAGAGCGATGACTACATGCCCATGAGCCCCACAAGCGTGTCTGCTCCCAAGCAGATCCTGCAGCCACGCTTGGCAGCGGCCTTGCCCCCTTCCGGAGCAGCCGTGCCAGCACCCCCTTCAGGGGTGGGCAGGACCTTCCCAGTAAACGGAGGTGGCTACAAAGCCAGCTCCCCAGCGGAGAGCTCCCCAGAAGACAGTGGGTACATGCGAATGTGGTGTGGCTCCAAGCTGTCTATGGAGAACCCAGACCCTAAGCTACTCCCCAACGGGGACTACCTCAACATGTCCCCCAGCGAGGCAGGCACTGCAGGGACCCCACCTGACTTCTCAGCAGCTTTGCGTGGAGGCAGTGAAGGCCTCAAAGGCATCCCGGGCCACTGCTACAGCTCTTTGCCCCGCTCTTATAAGGCTCCCTGTTCCTGCAGCGGAGACAATGACCAGTATGTGCTCATGAGCTCCCCTGTGGGCCGGATCTTGGAAGAGGAGAGACTGGAGCCCCAGGCCACCCCAGGGGCTGGCACCTTTGGGGCAGCTGGTGGTAGTCATACCCAGCCTCATCACTCAGCAGTGCCTTCCTCCATGAGGCCGAGTGCCATCGGTGGCCGCCCTGAGGGCTTCCTGGGCCAGCGATGTCGGGCAGTGCGGCCTACACGCCTATCGCTAGAGGGACTGCAGACCCTTCCCAGCATGCAAGAGTACCCTCTACCCACAGAGCCCAAGAGCCCTGGCGAGTACATCAACATTGACTTTGGTGAGGCAGGTACCCGTCTGTCTCCGCCTGCCCCCCCACTACTGGCATCCGCGGCCTCATCTTCTTCACTGCTCTCAGCTAGTAGTCCTGCTTCATCCCTGGGTTCAGGAACCCCAGGCACCAGCAGCGACAGCCGGCAGCGCTCTCCACTCTCTGACTATATGAACCTGGACTTCAGTTCTCCCAAGTCCCCCAAGCCTAGCACCCGCAGTGGGGACACAGTAGGCTCCATGGATGGCCTTCTCTCTCCAGAGGCTTCATCCCCATACCCACCACTGCCCCCACGTCCTTCCACTTCCCCTTCCTCCTTACAGCAGCCTCTGCCACCTGCCCCGGGAGACCTATACCGCCTGCCTCCAGCATCAGCTGCCACTTCCCAGGGTCCCACTGCTGGCTCCTCAATGTCCTCCGAGCCTGGGGATAATGGTGACTATACCGAGATGGCCTTTGGTGTGGCTGCAACCCCGCCACAACCTATCGTGGCACCTCCAAAGCCAGAAGGTGCCCGAGTGGCCAGTCCCACATCGGGCTTGAAGCGGCTAAGTCTCATGGATCAGGTATCTGGGGTGGAGGCTTTCCTTCAAGTCAGCCAGCCCCCTGACCCCCACCGGGGTGCTAAGGTCATCCGTGCAGACCCACAGGGGGGACGTCGTCGCCACAGTTCAGAGACCTTTTCCTCTACCACCACCGTCACCCCAGTGTCCCCATCCTTTGCCCACAATTCCAAGCGCCACAATTCGGCCTCTGTGGAAAATGTCTCACTCAGGAAAAGCAGTGAAGGCAGCAGTACCCTGGGAGGAGGTGATGAGCCGCCCACATCCCCAGGACAGGCACAGCCCTTGGTGGCTGTGCCCCCAGTGCCACAGGCTAGGCCGTGGAACCCCGGTCAGCCCGGAGCTTTGATTGGCTGTCCTGGAGGCAGCAGTTCTCCCATGCGCAGAGAGACCTCCGTGGGTTTCCAGAACGGCCTCAACTATATCGCCATCGATGTGAGAGGCGAGCAGGGGTCCTTGGCGCAGTCTCAGCCGCAGCCAGGAGACAAGAACTCCTGGAGCCGGACCCGTAGCCTTGGGGGGCTCCTCGGCACCGTCGGAGGCTCTGGCGCCAGCGGAGTGTGTGGGGGTCCAGGCACTGGAGCTTTGCCCTCTGCCAGCACCTATGCAAGCATCGACTTCCTGTCCCATCACTTGAAGGAAGCCACAGTCGTGAAAGAGTGA

>Rat_no2 [Rat (Rattus norvegicus) Irs2]

ATGGCTAGCGCGCCCCTGCCTGGGCCCCCCGCGTCGGCGGGCGGGGACGGCCCGAACCTCAATAACAACAACAACAACAACAACAACCACAGCGTGCGCAAGTGCGGCTACCTGCGCAAGCAGAAGCACGGCCACAAGCGCTTCTTCGTGCTGCGCGGCCCGGGCACCGGCGGCGAGGAGGCAGCCGCGGCTGGGGGGTCGCCGCCGCAGCCTCCGCGGCTGGAGTACTATGAGAGCGAGAAGAAGTGGAAGAGCAAGGCGGGCGCGCCGAAGCGAGTGATCGCGCTCGACTGCTGTCTGAACATCAACAAGCGCGCGGACGCCAAGCACAAGTACCTGATCGCCCTCTACACCAAGGACGAGTACTTCGCCGTAGCGGCGGAGAACGAGCAGGAGCAGGAGGGCTGGTACCGCGCACTCACCGACTTGGTCAGCGAAGGCCGCTCTGGCGATGGGGGCTCCGGCACCACGGGCGGCTCTTGCAGCGCCTCTCTCCCGGGGGCCCTGGGCGGCTCGGCGGGCGCCGCTGGCTGCGATGACAACTACGGGCTCGTGACGCCCGCCACGGCAGTCTACCGCGAGGTGTGGCAGGTGAACCTGAAGCCTAAGGGACTGGGCCAGAGCAAGAACCTGACCGGTGTATACCGCCTATGCCTGTCTGCGCGCACCATCGGCTTCGTGAAGCTCAATTGCGAACAGCCGTCGGTGACGCTGCAGCTTATGAACATTCGCCGCTGCGGCCACTCGGACAGCTTCTTCTTCATCGAGGTGGGCCGTTCAGCCGTCACCGGTCCCGGCGAGCTGTGGATGCAAGCCGATGACTCGGTGGTGGCGCAGAACATCCATGAGACCATCCTGGAGGCCATGAAGGCACTCAAGGAGCTCTTCGAGTTCCGGCCTCGCAGCAAGAGTCAGTCGTCTGGGTCGTCTGCCACGCACCCCATCAGCGTACCGGGCGCGCGCCGCCACCACCACCTAGTCAACCTACCCCCTAGCCAGACGGGCCTGGTGCGCCGCTCTCGCACTGATAGCCTGGCGGCCACCCCGCCAGCAGCCAAGTGCACTTCGTGCCGGGTTCGTACAGCCAGCGAGGGTGACGGCGGCGCGGCAGGCGGAGCCGGGACGGCAGGAGGCAGGCCGATGTCGGTGGCAGGGAGCCCCCTGAGTCCCGGGCCGGTGCGCGCGCCCCTTAGCCGCTCGCACACCCTGAGCGCCGGCTGCGGAGGCCGCCCGAGCAAAGTGGCTCTGGCGCCGGCAGGGGGAGCCCTACAACACAGCCGCTCCATGTCCATGCCCGTGGCGCACTCGCCGCCTGCAGCCACCAGCCCAGGCAGCCTGTCCTCCAGCAGTGGGCATGGCTCAGGCTCCTACCCGCTGCCTCCTGGTTCCCACCCGCACCTGCCTCATCCGCTCCATCACCCCCAATGCCAACGTCCGTCCAGCGGAAGTGCCTCCGCCTCGGGCTCCCCCAGCGACCCGGGTTTCATGTCCCTTGACGAGTATGGCTCCAGCCCTGGTGACCTGAGAGCCTTCAGTAGCCACAGGAGCAACACACCTGAGTCCATCGCGGAGACCCCGCCAGCCAGGGACGGCAGTGGGGGCGAGCTCTATGGGTATATGAGCATGGATAGGCCCCTGAGCCACTGTGGCCGCCCTTACCGTAGGGTCTCTGGGGATGGGGCCCAGGATCTGGACAGAGGACTGAGGAAGAGGACTTACTCCCTAACCACGCCTGCCCGGCAGCGGCAGGTTCCTCAGCCTTCCTCTGCCTCTCTAGACGAATACACTCTCATGCGGGCCACCTTCTCTGGCAGTTCAGGTCGCCTCTGCCCATCCCTCCCTGCGTCCTCTCCCAAAGTGGCCTACAACCCTTACCCAGAGGACTATGGAGACATTGAGATTGGTTCTCACAAGAGTTCCAGCAGTAACCTGGGGGCAGATGATGGCTACATGCCCATGACCCCTGGGGCAGCCCTCAGGAGTGGTGGCCCCAATAGCTGCAAGAGCGATGACTACATGCCCATGAGCCCCACCAGCGTGTCTGCCCCTAAGCAGATCCTGCAACCACGTTCGGCAGCGGCCTTGCCCCCCTCTGGAGCAGCCGTGCCAGCACCCCCTTCAGGGGCGGGCAGGACTTTCCCAGTGAACGGAGGCGGCTACAAAGCCAGCTCCCCAGCGGAGAGCTCCCCAGAAGATAGCGGGTACATGCGAATGTGGTGTGGCTCCAAGCTGTCCATGGAGAACCCAGACCCTAAGCTGCTCCCCAATGGGGACTACCTCAACATGTCCCCCAGTGAGGCAGGCACCGCAGGGACCCCACCTGACTTCTTCTCAGCAGCTTTGCGTCCAGGCGGTGAGGCCCTCAAAGGCGTCCCTGGCCACTGCTACAGCTCTTTGCCCCGCTCTTACAAGGCTCCCTGTACTTGCGGTGGTGGAGACAACGACCAGTACGTGCTCATGAGCTCCCCTGTGGGTCGGATTTTGGAAGAGGAGAGACTGGAGCCCCAGGCCACCCCAGGGGCTGGCACATTTGGGGCAGCTGGTGGTAGTCATACCCAGCCTCATCACTCAGCAGTGCCTTCCTCCATGAGACCAAGTGGCATCGTTGGCCGACCTGAGGGCTTCCTGGGCCAGCGCTGTCGGGCAGTGCGGCCCACACGCCTTTCGCTAGAGGGACTGCAGACCCTTCCCAGCATGCAAGAGTACCCTCTACCCACTGAGCCCAAGAGCCCTGGCGAGTACATCAACATTGACTTTGGTGAAGGGGGTACCCGTCTGTCTCCGCCTGCTCCCCCACTACTGGCATCAGCGGCCTCCTCCTCTTCACTGCTCTCAGCCAGTAGTCCTGCTTCATCCCTGGGTTCAGGTACCCCAGGCACCAGCAGTGACAGCCGGCAGCGCTCTCCGCTCTCTGACTATATGAACCTGGACTTCAGTTCTCCCAAGTCCCCTAAGCCTAGCACCCGCAGTGGGGACACAGTGGGCTCCATCGATGGCCTTCTCTCTCCAGAAGCCTCATCCCCATACCCACCGCTGCCCCCGCGTCCTTCTGCTTCCCCTTCCTCCTTACAGCAGCCTCTGCCACCTGCCCCAGGAGACCTATACCGCCTGCCTCCAGCAACAGCTGCCACATCCCAGGGTCCCACTGCTGGCTCCTCAATGTCCTCTGAGCCTGGGGATAATGGTGACTATACCGAGATGGCCTTTGGTGTGGCTGCTACCCCGCCACAACCTATTGCGGCACCCCCGAAGCCAGAAGGTGCCCGAGTGACCAGTCCCACATCAGGCTTGAAGCGGCTAAGTCTCATGGATCAGGTATCTGGGGTAGAGGCCTTCCTTCAAGTCAGCCAGCCCCCTGATCCCCACCGGGGCGCTAAGGTCATCCGTGCAGACCCGCAGGGGGGACGTCGTCGCCACAGTTCAGAGACCTTCTCCTCTACCACCACTGTCACCCCAGTGTCCCCATCCTTTGCTCACAATTCCAAGCGCCACAATTCGGCCTCTGTGGAAAACGTCTCTCTCAGGAAAAGCAGTGAAGGCAACAGCATCCTGGGAGGAAGTGATGAGCCATCCACATCCCCAGGACAGGCACAGCCCTCGGCAGGTGTGCCCCCAGCGCCACAGGCTAGGCCATGGAACCCCGGTCAGCCTGGAGCTTTGATTGGCTGTCCTGGAGGCAGCAGTTCTCCCATGCGCAGAGAGACCTCCGTGGGTTTCCAGAACGGCCTCAACTATATCGCCATCGATGTGAGAGGTGAGCAGGGGTCCTTGGCGCAGTCTCAGCCTCAGCATCCACAGCCAGGAGACAAGAACTCCTGGGGCCGGACCCGTAGCCTTGGGGGGCTCCTCGGTACCGTTGGAGGCTCTGGCACCAGTGGAGTGTGTGGGGGTCCAGGCACTGGAGCCTTACCCTCTGCCAGCACCTACGCAAGCATCGACTTCTTGTCCCATCACTTGAAAGAAGCCACCGTGGTGAAAGAGTAA

>Mes_au2 [Golden hamster (Mesocricetus auratus) Irs2]

ATGGCTAGCGCGCCCCTTCCTGGGCCCCCCGCGCCGGCGGGTGGGGACGGCCCGAACCTCAATAACAACAACAACAACAACAACCACAGCGTGCGCAAGTGCGGCTACCTGCGCAAGCAGAAGCACGGCCACAAGCGCTTCTTCGTGCTGCGCGGCCCCGGCACAGGCGGAGACGAGGCGGCCGCGGCTGGGGGGTCGCCGCCGCAGCCGCCGCGGCTGGAGTACTACGAGAGCGAGAAGAAGTGGAGGAGCAAGGCGGGCGCGCCGAAGCGAGTGATCGCGCTCGACTGCTGCCTGAACATCAACAAGCGCGCGGACGCCAAGCACAAGTACCTGATCGCCCTCTACACCAAGGACGAGTACTTCGCCGTGGCGGCGGAGAACGAGCAGGAGCAGGAGGGCTGGTACCGCGCCCTCACCGACTTGGTCAGCGAGGGTCGCTCTGGCGATGGGGGCTCGGGCATCGCGGCCACCGGCGGGTCCTGCAGCGCCTCTCTTCCTGGCGTCCTGGGTGGCTCGGCAGGCGCCGCTGGCTCCGATGACAACTACGGGCTCGTGACGCCCGCCACGGCAGTCTACCGCGAGGTGTGGCAGGTGAACCTGAAGCCCAAGGGCCTGGGCCAGAGCAAAAATCTGACCGGTGTCTACCGCCTATGCCTGTCTGCGCGCACCATCGGCTTCGTGAAGCTCAATTGCGAACAGCCGTCGGTGACGCTGCAGCTTATGAACATCCGCCGCTGCGGCCACTCGGACAGCTTCTTCTTCATCGAGGTGGGCCGCTCGGCGGTCACCGGCCCCGGCGAGCTGTGGATGCAAGCCGACGACTCGGTGGTGGCACAGAACATCCACGAGACCATCCTGGAGGCCATGAAGGCTCTTAAGGAGCTCTTTGAGTTCCGGCCTCGAAGCAAGAGTCAGTCGTCCGGATCGTCGGCCACGCACCCCATCAGCGTGCCGGGCGCGCGCCGCCACCATCACCTGGTCAACCTGCCCCCTAGCCAGACCGGCCTGGTGCGCCGCTCGCGCACCGACAGCCTGGCGGCCACCCCGCCAGCAGCCAAGTGCACCTCGTGTCGGGTCCGTACGGCCAGCGAGGGCGATGGCGGCGCGGCGGCCGGGGCCGGGGCAGCGGGAGGCAGGCCTATGTCGGTAGCCGGGAGCCCCCTGAGTCCCGGGCCAGTGCGCGTGCCCCTGAGTCGCTCGCACACCCTGAGCGGCGGCTGCGGAGGCCGCCCGAGCAAAGTGACGCTGGCGCCGGCAGGGGGCGCCCTGCAACACAGCCGCTCCATGTCCATGCCCGTGGCGCACTCGCCGCCTGCAGCCACCAGCCCTGGCAGCCTATCCTCCAGCAGTGGGCACGGCTCAGGCTCCTACCCGCTTCCTCCCGGTCCCCACCCGCACCTGCCTCACCCGCTGCACCACCCCCAAGGCCAGCGTCCGTCCAGCGGCAGTGCCTCAGCCTCCGGCTCCCCTAGTGACCCGGGTTTCATGTCACTTGACGAGTATGGCTCCAGCCCTGGCGACCTCAGAGCCTTCAGTAGCCACAGGAGCAACACGCCCGAGTCAATCGCGGAGACCCCGCCAGCCAGGGACGGCAGTGGGGGCGAGCTCTATGGGTACATGAGCATGGACAGACCCCTGAGCCACTGTGGCCGCCCGTACCGTAGGGTCTCTGGGGATGGGGCTCAGGACCTGGACAGAGGACTGAGGAAGAGGACTTATTCCCTGACCACGCCTGCCCGGCAGCGGCCAGTACCCCAGCCTTCCTCTGCCTCCCTGGATGAATACACTCTGATGCGGGCCACCTTCTCTGGCAGTTCCGGACGCCTCTGCCCACCCTTCCCCGCGTCCTCTCCTAAACTGGCCTACCACCCTTACCCGGAGGACTATGGGGACATTGAGATTGGGTCTCACAAGAGTTCCAGCAGTAACTTGGGGGCAGATGATGGCTACATGCCCATGACCCCTGGGGCGGTCCTCAGGAGCGGTGGCCCCGGTAGCTGCAAGAGTGACGATTATATGCCCATGAGCCCCACCAGCGTGTCTGCCCCCAAGCAGATCATGCAGCCGAGGTCAGTGGCCGCTACAGCTTTGCCCCCCTCGGGAGGAGCAGTGCCAGCACCCGCCTCGGGGCCAGGCAGAACCTTCCCGGTGAACGGAGGCAGCTACAAAGCCAGCTCCCCAGCGGAGAGCTCCCCAGAGGATAGCGGGTACATGCGGATGTGGTGTGGGTCCAAGCTGTCCATGGAGAACGCAGACCCAAAGCTGCTCCCCAACGGGGACTACCTCAACATGTCCCCCAGCGAGGCTGGCGCTGCGGGCACGCCGCCCGACTTTTTCTCAGCAGCTTTGCGTGGAAGCGGTGAGGGCCTCAAAGGTGTCCCTGGCCACTGCTACAGCTCTTTGCCGCGCTCTTACAAGGCTCCCTATTGCAGTGGGGACAGCGACCAGTATGTGCTCATGAGCTCCCCTGTGGGGCGAATCTTGGAAGAGGAAAGGCTGGAGCCCCAGGCCACCCCAGGGACCTCCCAGTTGACTGGCACCTTTGGGGCAGCTGGGGTCGGCCATACCCAGCCTCACCACTCAGCAGTGCCTTCCCCCATGAGGCCTAGTGGCAGCAGCGTTGGCCGCCCCGAGGGCTTCCTGGGCCAGCGCTGTAGGGCAGTGCGACCCACGCGCTTGTCCCTGGAGGGACTGCAGACCCTTCCCAGCATGCAAGAGTACCCTCTGCCCGCAGAGCCCAAGAGCCCAGGCGAGTACATCAACATTGACTTCGGTGAGGCAGGTACCCGCCTGTCCCCGCCTGCCCCTCCACTGCTGGCGTCGGCGGCTTCATCCTCTTCTCTGCTCTCGGCCAGCAGCCCTGCTTCATCCCTGGGCTCCGGTACCCCAGGCACCAGCAGTGACAGCCGGCAGCGCTCCCCGCTCTCCGACTACATGAACTTGGACTTCAGCTCTCCCAAGTCCCCTAAGCCTGGCACCCACAGTGGGGGCGCAGCGGGCTCCATGGATGGCCTCCTCTCCCCAGAGGCCTCATCCCCGTACCCCCCACTGCCCCCACGTCCTTCTGCGCCCCCCTCTTCCCTGCAGCAGCCTCTGCCGCCTGCCCCAGGAGAGCTGTACCGCCTGCCTCCCGTGTCAGCTGCCACCTCCCAGGGACCCACTGCTGGCTCCTCCAAGTCCTCGGAGCCTGGGGATAATGGTGACTACACCGAGATGGCCTTTGGTGTGGCTGCCACCCCGCCACAACCTATTGCGGCACCCCCAAAGCCAGAAGGTGCCCGAGTGACCAGTCCCACATCTGGCTTGAAGCGCCTGAGTCTCATGGATCAGGTTTCTGGGGTGGAGGCCTTTCTTCAAGTCAGCCAGCCCCCTGACCCCCACCGGGGTGCCAAGGTCATCCGTGCAGACCCACAGGGGGGGCGGCGGCGTCACAGCTCAGAGACCTTCTCCTCTACGACCACAGTCACCCCAGTGTCCCCCTCCTTTGCCCACAACTCCAAGCGCCACAGTTCTGCCTCCGTGGAAAACGTCTCTCTCAGGAAAAGCAGTGAAGGCAGTGGCTCCCTGGGAGGAAACGATGAGCCGCCCACCTCCCCAGGACAGGCACAGCCCTCGCTGGCTGTGCCCCCAGGGCCACAGGCTAGGCCATGGAACCCAGGTCAGCCTGGAGCTTTGATTGGCTGTCCGGGAGGCAGCAGCTCTCCCATGCGCAGAGAGACCTCTGTGGGTTTCCAGAACGGCCTCAACTATATCGCCATCGACGTGAGGGATGAGCAGGGGTCCTTATCGCAGACTCAGCAGCCGCATCCCCAGCCAGGAGACAAGAGCTCCTGGGGCCGGACCCGAAGCCTCGGGGGGCTCCTCGGCACCGTGGCAAGCACTGGCAGCAGTGGGGTGTGTGGGGGCCCGGGCACTGGAGCCTTGTCCTCTGCCAGCACCTATGCCAGCATCGACTTCCTGTCCCATCACTTGAAGGAAGCCACAGTCGTGAAAGAGTGA

>Mic_oc2 [Prairie vole (Microtus ochrogaster) Irs2]

ATGGCTAGCGCGCCCCTACCCGGGGCCCCCGCGCCGGCGGTAGGGGAGGGCCCGAACCTCAATAACAACAACAACAACAACAACCACAGCGTGCGCAAGTGCGGCTACCTGCGCAAGCAGAAGCACGGCCACAAGCGCTTCTTCGTGCTGCGCGGCCCCGGCACGGGCGGCGACGATGCAGCCGCGGCTGGGGGGTCGCCGCCGCTGCCACCGAGGCTGGAGTACTATGAGAGCGAGAAGAAGTGGAGGAGCAAGGCGGGCGCGCCGAAGCGAGTGATCGCGCTCGACTGCTGCCTGAACATCAACAAGCGCGCGGACGCCAAGCACAAGTACCTGATCGCCCTCTACACCAAGGACGAGTACTTCGCCGTGGCGGCGGAGAACGAGCAGGAGCAAGAGGGCTGGTACCGCGCGCTCACCGACTTGGTCAGCGAGGGTCGCTCTGGCGATGGGGGCTCGGGCACCGCGGCCACCGGCGGGTCCTGCAGCGCCTCTCTCCCGGGCGTCCTGGGCGGCTCGGCGGGTGCCGCTGGCTCCGATGACAACTACGGGCTCGTGACGCCCGCCACGGCAGTCTACCGTGAGGTGTGGCAGGTGAACCTGAAGCCCAAGGGCCTGGGTCAGAGCAAAAACCTGACTGGTGTATACCGTCTATGTCTGTCTGCGCGCACCATCGGCTTCGTGAAGCTCAATTGCGAACAGCCGTCGGTGACGCTGCAGCTTATGAACATTCGCCGCTGCGGCCACTCAGACAGCTTCTTCTTCATCGAAGTGGGCCGCTCTGCCGTCACCGGCCCCGGCGAGCTGTGGATGCAAGCCGACGACTCGGTGGTGGCGCAGAACATCCACGAGACCATCCTGGAGGCCATGAAGGCTCTCAAGGAGCTCTTTGAGTTCCGGCCTCGCAGCAAGAGTCAGTCGTCCGGGTCGTCGGCCACCCACCCCATCAGTGTGCCGGGCGCGCGCCGCCACCACCACCTGGTCAACTTGCCCCCTAGCCAGACCGGCCTGGTGCGCCGCTCACGCACCGACAGCCTGGCGGCCACCCCGCCAGCAGCCAAGTGCACCTCATGTCGGGTCCGTACGGCCAGCGAGGGCGATGGCGGCGCGGCAGCCGGGGCCGGGACAGCGGGAGGCAGGCCGATGTCGGTGGCCGGGAGCCCCCTGAGTCCTGGGCCGGTGCGCGCGCCCCTTAGTCGCTCGCACACCCTGAGCGGCGGCTGCGGAGGCCGCCCGAGCAAAGTGACGCTGGCGCCGGCAGGGGGCGCCCTGCAACACAGCCGTTCCATGTCCATGCCCGTGGCGCACTCACCGCCCGCTGCCACCAGCCCCGGCAGCCTATCCTCCAGCAGTGGACACGGCTCGGGCTCCTACCCGCTGCCTCCAGGCCCCCACCCGCACCTGCCTCATCCGCTGCACCACCCCCAAGGCCAGCGTCCATCCAGCGGCAGTGCCTCTGCCTCTGGCTCCCCCAGTGACCCTGGTTTTATGTCCCTTGATGAGTATGGCTCCAGCCCTGGCGACCTGAGAGCCTTCAGTAGCCACCGGAGCAACACACCCGAGTCAATCGCGGAGACCCCGCCAGCCAGGGACGGCAGTGGGGGCGAGCTCTATGGGTACATGAGCATGGATAGACCCCTGAGCCACTGTGGCCGCCCTTACCGTAGGGTCTCTGGGGATGGGGCCCAGGACCTGGACAGAGGGCTGAGGAAGAGGACTTATTCCTTGACCACACCTGCCCGACAGCGGCCAGTACCCCAGCCTTCCTCTGCCTCCCTTGATGAGTATACTCTCATGCGGGCCACCTTCTCTGGCAGTTCAGGTCGCCTCTGCCCATCCTTCCCCGTGTCTTCTCCCAAAGTGGCCTATCACCCTTACCCAGAGGACTATGGAGACATTGAGATTGGCTCCCACAAGAGTTCCAGCAGTAACCTGGGGGCAGATGATGGTTACATGCCCATGACCCCTGGGGCTGCCCTTAGGAGTGTTGGCCCCAGTAGCTGCAAGAGTGACGACTACATGCCCATGAGCCCCACCAGCGTGTCTGCCCCCAAGCAGATCCTGCAGCCGAGGTCAGCGGCGGCAGCAGCCTTGCCACCCTCGGGAGCTGCAGTGCCAGCACCCCCTTCAGGGGCAGGCAGGACCTTCCCCTTGAACGGAGGCAGCTACAAAGCCAGCTCCCCAGCAGAGAGCTCTCCGGAGGATAGCGGGTACATGCGAATGTGGTGTGGCTCCAAGCTGTCCATGGAGAACACAGACCCAAAGCTGCTCCCCAACGGGGACTACCTCAACATGTCCCCCAGCGAGGCTGGCACCGCGGGCACGCCACCCGACTTCTTCTCAGCAGCTTTGCGTGGAGGGGGAGAGGGCCTCAAGGGTGTCCCTGGACACTGCTACAGCTCTCTGCCCCGCTCTTACAAGGCCCCCTATTGTAGTGGGGACAGTGACCAATATGTGCTCATGAGCTCCCCTGTGGGGCGAATCTTGGAAGAGGAGAGGCTGGAACCCCAGGCCACCTCTGGAACCTCCCAGTTGGCTGGCACCTTTGGGGCAGCTGGGGGTGGCCATACCCAGCCTCACCACTCAGCAGTGCCTTCCTCCATGAGGCCAAGTGGCGGCAGTGGCCGCCCTGAGGGCTTCCTGGGCCAGCGCTGTCGGGCAGTGCGACCCACGCGCTTGTCGCTGGAGGGACTGCAGACCCTTCCCAGCATGCAAGAGTACCCTTTACCCTCAGAGCCCAAGAGCCCTGGCGAGTATATCAACATTGACTTTGGTGAGGCGGGTACCCGTCTGTCCCCGCCTGCCCCCCCACTGCTGGCATCAGCGGCTTCATCCTCTTCGTTGCTTTCAGCCAGTAGTCCCGCTTCATCCCTGGGTTCAGGCACCCCAGGCACCAGCAGTGACAGCCGGCAGCGCTCCCCACTCTCTGACTATATGAACTTGGACTTCAGTTCTCCCAAGTCCCCTAAACCTGTCGCTCATAGTGGAGACACAGTAGGCTCCATGGATGGCCTTCTCTCCCCAGAGGTCTCATCTCCATACCCACCTCTGCCCCCACGTCCTTCTGCCTCCCCCTCTTCCCTGCAGCAGCCTCTGCCACCTGCCCCAGGAGAGCTCTACCGCTTGCCTCCAGCATCAGTTGCCACTTCCCAGGGCCCCACTGCTGGCTCCTCAACATCCTCAGAGCCTGGGGATAATGGTGACTATACCGAGATGGCCTTTGGTGTGGCTGCTACCCCGCCACAACCTATCGCAGCACCCCCAAAGCCAGAAGGTGCCCGAGTAACCAGTCCCACATCTGGCTTGAAGCGCCTAAGTCTCATGGATCAGGTGTCTGGGGTGGAGGCCTTTCTTCAAGTCAGCCAGCCCCCTGATCCCCACCGGGGTGCCAAGGTCATCCGTGCAGACCCACAGGGGGGACGACGTCGCCACAGCTCAGAGACCTTCTCCTCCACCACCACAGTCACCCCAGTGTCCCCCTCCTTCGCCCACAACTCTAAGCGCCACAATTCGGCCTCTGTGGAAAACGTCTCTCTCAGGAAAAGCAGCGAAGGTAGTTGCATCCTGGGAGGAAGTGATGAGCCGCCCACATCCCCAGGACAGGCACAGCCATCGCTGGCTGTGCCTCCAGCCCCGCAGGCTAGGCCATGGAACCCAGGTCAACCTGGGGCTTTGATTGGCTGTCCTGGAGGCGGCAGTTCTCCCATGCGCAGAGAGACCTCTGTGGGTTTCCAGAACGGCCTCAACTACATCGCCATTGACGTGAGGGATGAGCAGGGGTCCCTGTCGCCATCTCAGCCACAGCACCCACAGCCTGGAGACAAGAGTTCCTGGGGCCGGACCCGTAGCCTTGGGGGACTTCTCAGCACCGTGGGAAGCACTGGCACCGGCGGGGTGTGTGGGGGCCCGGGCCCCGGAGCCTTGCCCTCTGCCAGCACCTACGCAAGCATTGACTTCTTGTCCCACCACTTGAAGGAAGCCACAGTCGTGAAAGAGTGA

>Per_ma2 [Prairie deer mouse (Peromyscus maniculatus bairdii) Irs2]

ATGGCTAGCGCGCCCCTGCCTGGGCCCCCCGCGCCAGCGGGCGGGGACGGCCCGAACCTCAACAACAACAACAACAACAACCACAGCGTGCGCAAGTGCGGCTACCTGCGCAAGCAGAAGCACGGCCACAAGCGCTTCTTCGTGCTGCGCGGCCCCGGCACGGGCGGCGACGAGGCAGCCGCGGCCGGGGGGTCGCCACCGCTGCCGCCGCGGCTGGAGTACTACGAGAGCGAGAAGAAGTGGAGGAGCAAGGCGGGCGCGCCGAAGCGAGTGATCGCGCTCGACTGCTGCCTGAACATCAACAAGCGCGCGGACGCCAAGCACAAGTACCTGATCGCCCTCTACACCAAGGACGAGTACTTCGCCGTGGCGGCGGAAAACGAGCAGGAGCAGGAGGGCTGGTACCGCGCGCTCACCGACTTGGTCAGCGAGGGTCGCTCTGGCGATGGGGGCTCGGGCATCGCGGCCACCGGCGGGTCCTGCAGCGCCTCTCTCCCAGGCGTCCTGGGCGGCTCGGCGGGCGCCGCTGGCTCCGATGACTACGGGCTCATAACGCCCGCCACGGCAGTCTACCGCGAGGTGTGGCAGGTGAACCTGAAGCCCAAGGGTCTGGGCCAGAGCAAAAACCTGACGGGTGTATACCGCCTATGCCTGTCTGCGCGCACCATCGGCTTCGTGAAGCTCAATTGCGAGCAGCCGTCGGTGACGCTGCAGCTTATGAACATCCGCCGCTGCGGCCACTCGGACAGCTTCTTCTTCATCGAGGTGGGCCGCTCGGCCGTCACCGGCCCCGGCGAGCTGTGGATGCAAGCCGACGACTCGGTGGTGGCGCAGAACATCCATGAGACCATCCTGGAGGCCATGAAGGCGCTCAAGGAGCTCTTTGAGTTCCGGCCTCGCAGCAAGAGTCAGTCGTCCGGGTCGTCGGCCACGCACCCCATCAGCGTGCCGGGCGCGCGCCGCCACCATCACCTGGTCAACCTACCCCCTAGCCAGACCGGCCTGGTGCGCCGCTCGCGCACCGACAGCCTGGCGGCCACCCCGCCAGCAGCCAAGTGCACCTCGTGTCGGGTCCGTACGGCCAGTGAGGGCGACGGCGGCGCGGCGGCCGCGGCCGGGACGGCGGGAGGCAGACCGATGTCGGTGGCCGGGAGCCCCCTGAGTCCCGGGCCGGTGCGCGCGCCCCTGAGCCGCTCGCACACCCTGAGCGCCGGCTGCGGAGGCCGGCCGAGCAAAGTCACGCTGGCGCCGGCAGGGGGCGCCCTGCAACACAGCCGCTCCATGTCCATGCCCGTGGCGCACTCGCCGCCTGCAGCCACCAGCCCCGGCAGCCTGTCCTCCAGCAGTGGGCACGGCTCGGGCTCCTACCCGCTCCCCCCCGGCCCCCACCCGCATCTGCCTCATCCGCTGCACCACCCCCAAGGCCAGCGTCCGTCCAGCGGCAGTGCCTCCGCGTCCGGCTCCCCCAGTGACCCGGGTTTCATGTCCCTTGACGAGTATGGCTCCAGCCCTGGCGACCTGAGAGCCTTCAGTAGCCACAGGAGCAACACGCCCGAGTCAATCGCGGAGACCCCGCCAGCTAGGGATGGCAGTGGGGGCGAGTTCTATGGGTACATGAGCATGGATAGACCCCTGAGCCACTGCGGCCGCCCTTACCGTAGGGTCTCTGGGGATGGGGCCCAGGACCTGGACAGAGGACTGAGGAAGAGGACTTACTCCCTGACTACACCTGCCCGGCAGAGGCCAGTACCCCAGCCTTCCTCTGCCTCCCTGGATGAGTATACTCTCATGCGGGCCACCTTCTCTGGGAGTTCAGGTCGCCTCTGCCCATCTTTCCCGGCATCCTCTCCCAAAATGGCCTACCACCCTTACCCAGAGGACTATGGAGACATTGAGATTGGCTCTCACAAGAGTTCCAGCAGTAACCTGGGGGCAGATGATGGCTACATGCCCATGACCCCTGGGGCGGCCCTCAGGAGTGGTGGCCCCAGTAGCCGCAAGAGTGATGATTACATGCCCATGAGCCCCACCAGTGTATCTGCCCCCAAGCAGATCCTGCAGCCCAGGTCAGCGGCGGCAGCCTTGCCGCCCTCAGGAGCAGCAGGGCCAGCGCCCCCTTCAGGACCAGGGAGGACCTTCCCAGTGAACGGGGGCAACAACTACAAAGCCAACTCCCCAGCGGAGAGCTCCCCAGAAGATAGCGGCTACATGCGCATGTGGTGTGGCTCCAAGCTGTCCATGGAGAACGCAGACCCAAAGCTGCTCCCCAACGGGGACTACCTCAATATGTCCCCCAGCGAGGCTGGTGCTGCCGCGGGCACGCCACCTGACTTCTTCTCAGCGGCTTTGCGCGGAGGTGGCGAGGGCCTCAAAGGTGTCCCTGGCCACTGCTACAGCTCTCTGCCCCGCTCTTACAAGGCCCCCTATTCTTGCAGTGGGGACAGTGACCAGTATGTGCTCATGAGCTCCCCTGTTGGGCGAATCTTGGAAGAGGAGAGGCTGGAACCCCAGGCCACCCCAGGGACCTCCCAGTTGGCCGGCGCCTTTGGGGCAGCTGGGGGTGGCCATACCCAGCCTCATCACTCAGCAGTGCCTTCCCCCATGAGGCCGAGTGGTAGCGGTGGCCGCCCTGAGGGCTTCCTGGGCCAGCGCTGTCGGGCAGTAAGACCCACGCGCTTATCTCTGGAGGGACTGCAGACCCTTCCTAGCATGCAAGAGTACCCTCTACCCACAGAGCCCAAGAGCCCTGGCGAGTACATCAACATTGACTTCGGGGAGGCGGGCACCCGTCTGTCCCCGCCTGCCCCGCCTCTGCTGGCCTCAGCGGCTTCGTCCTCCTCGCTGCTTTCAGCCAGTAGCCCTGCCTCGTCCCTGGGTTCGGGTACCCCGGGCACTAGCAGTGATAGCCGCCAGCGCTCCCCGCTCTCCGACTATATGAACTTGGACTTCAGTTCTCCCAAGTCCCCTAAGCCTGGCACCCATAGTGGGGACACAGTGGGCTCCATGGATGGCCTTCTCTCCCCAGAGGTCTCATCCCCATACCCACCACTGCCCCCTCGCCCTTCGGCCTCCCCCTCTTCCCTGCAGCAGCCTCTGCCACCCGCCCCGGGAGAGCTCTACCGCCTGCCTCCAGCACCCGCTGCCACTTCCCAGGGCCCCGCTGCTGCTGGCCCCTCATCGTCCTCGGAGCCTGGGGATAATGGTGACTATACCGAGATGGCCTTTGGTGTGGCTGCCACCCCGCCACAACCTATTGCGGCACCCCCAAAGCCCGAAGGTGCCCGAGTGGCCAGTCCCACATCGGGCTTGAAGCGCCTAAGTCTCATGGATCAGGTGTCTGGGGTAGAGGCCTTTCTTCAAGTCAGCCAGCCCCCTGATCCCCACCGGGGTGCCAAGGTCATCCGTGCGGACCCACAGGGCGGGAGACGTCGCCACAGTTCAGAGACCTTCTCCTCTACCACCACCGTCACCCCTGTGTCCCCGTCCTTCGCCCACAACTCCAAGCGCCACAATTCGGCCTCGGTGGAAAACGTCTCTCTCAGGAAAAGCAGCGAAGGCGGCAGTGGCATCCTGGGAGGAAGCGATGAGCCACCCACATCCCCGGGACAGGCACAGCCCTCGCCAGCTGTGCCACCGGCACCGCAGGCTAGGCCGTGGAACCCGGGCCAGCCCGGAGCTTTGATTGGCTGCCCCGGAGGCAGCAGTTCTCCCATGCGCAGAGAGACCTCCGTGGGTTTCCAGAACGGCCTCAACTATATCGCCATTGACGTGAGTGATGAGCCGGGGTCCTTGTCGCCGTCTCAGCCGCAGCATCCACAGCCAGGAGACAAGAGCTCCTGGGGCCGGACCCGTAGCCTTGGGGGGCTCCTCAGCTCCGTCGGAAGCACTGGCGCCAGCGGGGTGTGTGGGGGCCCAGGCACTGGAGCCTTGCCCTCAGCCAGCACCTACGCAAGCATCGACTTCTTGTCCCATCACTTGAAGGAAGCCACAGTCGTGAAAGAGTGA

>Het_gl2 [Naked mole rat (Heterocephalus glaber) Irs2]

ATGGCGAGCCCGCCGCGGCAGGGAGCCCCTGGGCCAGCGGGCGGTGACGGCCCCAACCTGAATAACAACAACAACAACAACAACAACAACCACGGCGTGCGCAAGTGCGGCTACCTGCGCAAGCAGAAGCACGGCCACAAGCGCTTCTTCGTGCTGCGCGGGCCCGGCGCGGGCGGGGACGACGGGGGCGCGGGCGGGGGGCCGGCGGCGCCGCAGCCCCCGCGGCTCGAGTACTACGAGAGCGAGAAGAAGTGGCGGAGCAAGGCGGGCGCGCCGAAGCGGGTGATCGCGCTCGACTGCTGCCTGAATGTCAACAAGCGCGCCGACGCCAAGCACAAGTACCTGATCGCCCTCTACACCAAGGACGAGTACTTCGCTGTGGCGGCCGAGAACGAGCAGGAGCAGGAGGGCTGGTACCGCGCGCTCGCCGACCTGCTCAGCGAGGGCCGCGCGGCCGCCGCCGGCGACGCGCCCCCTGTGGCCACGGCCCCCGCCGCCACACCCTGCAGCGCCCCCCTGCCGGGGGCGCTGGGCGGCTCGGCCGAGGACGCGGACGGGCCGGGGGCGTCCTCGGCCTCCGCCTACCGCGAGGTGTGGCAGGTGAACCTGAAGCCCAAGGGCCTGGGCCAGAGCAAGAACCTGACGGGCGTGTACCGCCTGTGCCTGTCGGCGCGCACCATCGGCTTCGTGAAGCTCAACTGCGAGCAGCCGTCCGTGACGCTGCAGCTCATGAACATCCGCCGCTGCGGCCACTCGGACAGCTTCTTCTTCATAGAGGTGGGGCGCTCGGCCGTCACGGGCCCCGGCGAGCTGTGGATGCAGGCCGACGACTCGGTGGTGGCGCAGAACATCCACGAGACCATCCTGGAGGCCATGAAGGCGCTCAAGGAGCTCTTCGAGTTCCGCCCGCGCAGCAAGAGCCAGTCCTCGGGCTCGTCGGCCACTCACCCCATCAGTGTGCCCGGCGCGCGCCGCCACCACCACCTGGTGCACCTGCCCCCGAGCCAGACCGGCCTGGTGCGCCGCTCGCGCACCGACAGCCTGGCCGCAACCCCGCCCGCCGCCAAGGGCGCCCCGTGTCGCGTGCGTACGGCCAGCGAGGGCGACGGCGGCGCCGCGGCCGGGGCCGGGGCCGGGGCGGCGGTGTCTGCGGCGGGCGCGCGTCCAGGCTCGGTGGCCGGGAGCCCCCTGAGCCCCGGACCGGCGCGCGCGCCCCTGAGCCGCTCGCACACGCTGAGCGCCGGCTGCGGGGCCCGGGGCGGCAAGGCGGGCCTGGCTCCGGCAGGGGGCGCGCTGCAGCACAGCCGCTCCATGTCCATGCCTGTGGCGCACTCTCCCCCTGCTGCCACCAGCCCGGGCAGTCTGTCGTCCAGCAGCGGCCACGGCTCGGCCTCCTACCCGCTGCCGCCCGGCCCGGCCCCGCCGCTGCCGCACCCCCTGCCCCACCCGCTGGGACAGCGACCCTCCAGCGGCAGCGCCTCGGCCTCGGGCTCTCCGAGCGACCCGGGCTTCATGTCCCTGGACGAGTACGGGTCTAGCCCGGGCGACCTGCGAGCCTTCTGCAGCAGCCACCGCAGCAACACGCCCGAGTCCATCGCGGAGACGCCCCCGGCGCGGGATGGCGCGGTGGGCGAGCTGTACGGGTACATGAGCATGGACAGGCCCTCGGGCCCCTTGGGACGCCCCTACCGCAGGGTCTCGGGGGACGCGGCGCAGGACCTGGACCGGGGCCTGCGAAAGCGGACCTACTCGCTGACCACGCCCGCGCGCCAGCGCCCCGTGCCCCAGCCGTCGTCCGCCTCGCTGGATGAGTACACGCTCATGCGGGCCACCTTCTCGGGCAGCTCCGGCCGCCTGTGCCCGCCCTTTCCTGCGTCTTCGCCCAAAGTGGCCTACCACCCCTATCCGGAGGACTATGGGGACATCGAGATCGGCTCACACCGGAGCTCCAGCAGCAACCTGGGGGCGGACGATGGCTACATGCCGATGACCCCTGGTGCAGCTCTGCTGGGCAGTGCCAGCGACGACTACATGCCCATGAGCCCCACCAGCGTGTCTGCCCCGAAGCAGATCCTGCATCCGAGGGCTGCACCGGCCGCGGCCCTGCCCCCCTCGGCGCCACCGGTGCCAGCTGCCCCCTCCCCAGCGGGCAAGACCTTCCCCGGGCCTGTGGGAGGCTACAAGGCCGGCTCACCTGCGGAGAGCTCTCCAGAGGACAGCGGGTACATGCGCATGTGGTGTGGGTCCAAGCTGTCTGTGGAGAGCGCCGACCCCAAGCTGCTGGCCAGCGGGGACTACCTCAACATGTCCCCCAGCGAGGCGGGCCCGGCCGGCACCCCGCCCGACTTCTTCTCCGCAGCTCTGCAGGGCGCTGGGGAGGGGCTCAGAAGTGTCCCCGGCTGCTGCTACAGCTCCTTGCCCCGCTCCTACAAGGGTCCTTACCTGTGTGGCGGGGCAGACAGTGACCAGTACGTGCTCATGAGCTCCCCGGTGGGCCGGATCCTGGAGGAGGAGGGACTGGAGTCCCCGGCCACCCCAGGGGCTCCCCCATGGACGGGCAGGGACAGCCACATCCAGCCGCATCACCTAGCAGCAGCAGTGCCTGCCCCCATGAGGCCAGGCAGCAACGCTGGTGGGCGCCCTGAGGGCTTCTTTGGCCAGCGCTGCCGGGCAGTGCGCCCCACACGCCTGTCCCTAGAGGGCTTGCAGACGCTGCCCAGCATGCATGAGTACCCCCTGCCCCCAGAGCCCAGGAGCCCTGGCGAGTACATCAACATTGACTTCGGGGAGGCGGGCCCCCGGCTGTCCCCGCCGGCCCCTTCGCTGCTGGCGTCTGCGGCCTCACCCTCCCCGCTGCTGTCTGCCAGCAGCCCGGCCTCCTCCCTCTCCCTGGGCTCAGGCACGCCGGGCACCAGCAGTGACAGCAGACAGCGCTCCCCGCTGTCCGACTACATGAACCTGGGCTTCAGCTCCCCCAAGTCCCCCAAGCCGGGGGTCCTGTCCCCCGAGGCCGCTTCCCCATACCCGCCGCTGCCCCCTCGTCCTGCGGCCTCCCTGTCCTCCCTGCAGCAGCAGCCCCCACCGCCGGTCCCCGGGGAGCTGTACCGCCTGCCTCCCGCACCCAGTGCGGCCCCTGCCTCCCAGGGCCCCAGCGCTGGTGCCTCGGTGTCCTCCCAGACTGCTGACAGCAGCGACTACGCCGAGATGGCCTTCGGCCTGGCGGCGCCCCCGAAGCCGGAAGGTGCCCGAGTGAGCAGCCCCACGGCGGGCCTGAAGCGGCTGAGCCTCAGGGGTCAGGGGTCGGGGCTGGAGGCCTTCCTGCAGGCTGGCCAGCCCCCCGACCCGCACCGGGGCGCCAAGGTCATCCGCGCAGACCCGCAGGGGGGCCGCCGCCGCCACAGCTCGGAGACTTTCTCCTCCACTGCCACGGTCACCCCCGTGTCCCCATCCTTCGCTCACAACCCCAAGCGCCACAGCTCGGCCTCTGTGGAGAACGTTTCTCTCCGGAAGAGCAGTGAGAGTGGTGGCGGTGGGGCTGAGGAGCCGCTCACCGCCCCAGGACACTCGACGTTGGCGCGGCCCAGGGCCTCCGGTCAGCCCGGGGGCCTGGGTGGCTGCCCGGCGGGCGGCGGCTCCCCCATGCGCAGAGAAGCCCCCGTGGCTTTTCCGAAAGGCCTCAGCTACATCGCCATCAACGTGAAGGAGGAGGCAGGGCTGTCCCCGCAGATGCCGCCCCCGCAGCCAGGGGACAAGACCTCGTGGGGCCGGACCCGGAGCCTGGGGGGCCTCCTCAGCGCGGTAGGGGGCAGCGGCAGCAGTGGGGCTTGTGGGGGCCCAGGCCCTGCCGCCCTGTCCTCCCCCAACAGCTACGCCAGCCTGGACTTCCTGTCCCATCACCTGAAGGAAGCCACGGCCGTGAAAGAGTGA

>Ovi_arm2 [Mouflon sheep (Ovis aries musimon) Irs2]

ATGGCGAGCCCGCCGGAGCACGGGCCCCCCGGGCCGGCGGGCGGGGACGGCCCCAACCTCAACAACAACAACAACAACAACAACCACAGCGTGCGCAAGTGCGGCTACCTGCGCAAGCAGAAGCACGGCCACAAGCGCTTCTTCGTGCTGCGCGGGCCCGGCGCGGGCGGCGACGAGGCGGGCGGGGGCCCGGCGCCGCAGCCGCCGCGGCTCGAGTACTACGAGAGCGAGAAGAAGTGGCGGAGCAAGGCGGGCGCCCCGAAGCGGGTCATCGCGCTCGACTGCTGCCTGAACATCAACAAGCGCGCCGACGCCAAGCACAAGTACCTGATCGCCCTCTACACCAAGGACGAGTACTTCGCCGTGGCAGCCGAGAACGAGCAGGAGCAGGAGGGCTGGTACCGCGCGCTCACCGACCTGGTCAGCGAGGGCCGCGCGGGCGCCGGCGACGCGCCCCCCGCCGCCGCCGCCACCTCCGGGTCCTGCAGCGCCTCCCTGCCCGGCGCCCTGGGCGGCTCGGCGGGCGCCGCCGCGGCCGATGACAGCTACGGGCTGGTGGCGCCCGCCACGGCCGCCTACCGCGAGGTGTGGCAGGTAAACCTGAAGCCCAAGGGCCTGGGCCAGAGCAAGAACCTGACGGGCGTGTACCGCCTGTGCCTGTCGGCGCGCACCATCGGCTTCGTGAAGCTGAACTGCGAGCAGCCGTCGGTGACGCTGCAGCTCATGAGCATCCGCCGCTGCGGCCACTCGGACAGCTTCTTCTTCATCGAGGTGGGCCGCTCGGCCGTGACGGGCCCCGGCGAGCTGTGGATGCAGGCGGACGACTCGGTGGTGGCGCAGAACATCCACGAGACCATCCTGGAGGCCATGAAGGCGCTCAAGGAGCTCTTCGAGTTCCGGCCGCGCAGCAAGAGCCAGTCGTCCGGCTCGTCGGCCACGCACCCCATCAGCGTGCCCGGTGCGCGCCGCCACCACCACTTGGTCAACCTGCCCCCGAGCCAGACGGGGCTGGTGCGCCGCTCGCGCACCGACAGCCTGGCCGCCACCCCGCCCGCCGCCAAGTGCAGCGCGTGCCGGGTGCGCACAGCCAGCGAGGGCGACGGCGGCGCCGCGGCGGGGGCGGCGGCGGGGGCCGGGGCGGCGGCGGGCAGCCCCCTGAGCCCGGGCCCGGTGCGCGCGCCCCTGAGCCGCTCGCACACGCTGAGCGGCGGCCGCGCGGGCAAGGCGGCGCTGGCGCCGGCAGGGGGCGGCCTGCAGCACAGCCGCTCCATGTCCATGCCCGTGGCGCACTCGCCCCCGGCGGCTACCAGCCCCGGCAGCCTGTCGTCCAGCAGCGGGCACGGCTCGGGCTCCTACCCGCCGCCCCCCGGCCCGCACCCGCACCTGCAGCACCCCCTGCACCCCCAGCGCCCTTCCAGCGGCAGCGCCTCGGCCTCGGGCTCCCCCAGCGACCCCGGCTTCATGTCCTTGGACGAGTATGGCTCCAGCCCCGGGGACCTGAGGGTCTACTGCGGCCACCGGAGCAACACGCCCGAGTCCATCGCTGAGACGCCCCCGGCGCGGGACGGCAGCGCGGGCGAGCTGTACGGCTACATGACCATGGAGCGGCCGCTGAGCCACTGCGGCGGCCGCGCCTACCGCAGGGTCTCCGGGGACGGCGCCCCAGACTTGGACCGAGGGCTGAGAAAGCGGACTTACTCGCTGACCACGCCTGCCCGGCAGCGGCCCGCGCCCCAGCCGTCCTCCGCGTCCCTGGATGAGTACACCTTGATGCGGGCCACCTTCTCGGGCAGTTCCGGCCGCCTGTGCCCATCCTGCCCCGCGTCTTCTCCCAAAGTGGCCTACCACCCGTACCCTGAGGACTACGGCGACATCGAGATCGGCTCGCACCGCAGCTCCAGCAGCAACCTGGGCACGGACGACGGCTACGTGCCCATGACCCCCGGCGTGGCCCCCCTGCGGACGGGCGGCGGGAGCTGCAGGGGCGACGACTACATGCCCATGAGCCCCACCAGCGTGTCGGCCCCGAAGCAGATCTTGCAGCCGCGGCCCGCACCCGCCGCCTTGCCCCCTGCGGGGGCCGCGGGGCCCACGCCCGCGTCGGCGGCCGGCAGAGCTTTCCCGGGCACTGCGGGCGGCTACAAGACCGGCTCCCCGGCCGAGAGCTCCCCCGAGGACAGCGGGTACATGCGCATGTGGTGTGGTTCCAAGCTGTCCATGGAGAGCGCCGACGGCAAGCTGCTGCCCAACGGGGACTACCTCAACATGTCCCCCAGCGACGCGGGCACCACGGGCACCCCGCCCGACTTCTTCTCGGCCGCGGGGGAGACGCTGCGGGGCATGCCCGGCTACTGCTACAGCTCTCTGCCCCGCTCCTACAAGGCCCCCCACGCCTGCCACGGCGACAGCGACCAGTACGTGCTCATGAGCTCGCCTGTGGGCCGGGTCCTGGAGGAGGAGCAGCTGGAGCCGGCCCCCGGCCCCGCGCAGCCGGCCAGTGCCTTCCTGGCCGCGGCGGCTGGCAGCGGCCACCCCCAGCCACCTCATCCCGCCGTGCCTTCTCCCGGGAGGCTCGGCGGTGGCGGAGGCAGCCGCCCCGACGGCTTCCTGGCCCAGCGCTGCCGGGCAGTGAGGCCCACGCGCCTGTCCCTGGAGGGGCTGCCGGCCCTGCCCCGCATGCACGAGTACCCGCTGCCCCCCGAGCCCAGGAGTCCAGGCGAGTACATTAACATCGACTTCGGGGAGGCCGGCGCGCGCCTGTCGCCGCCCGCGCCCCCACTCCTGGCCTCGGCCGCCTCGTCGTCCTCGCTGTTGTCCGCCAGCAGCCCGGCCTCGTCCCTGGGCTCCGGCACCCCGGGCACGAGCGGGGACAGCCGGCAGCGCTCCCCGCTCTCCGACTACATGAACCTCGACTTCAGCTCGCCTAAGTCGCCGCAGCCGGGCGCCCAGGGCCGCGACCCCGTAGGCTCCTTGGACGCCCTGTTGTCCCCCGAGGCCTCCGTGTACCCGCCACTGCCCCCGCGCCCGGCCGCCCCCTCCTCGGCCCTGCAGCCGCCGCCGCCGCCGCCCCCGCCAGGCGAGCTGTACCGCCTGCCTGCGGCACCTGCCTCCAAGGGCCCGGGCGCGGCCTCCTCGTCCTCGGACACGGGGGACAATGGTGACTACACCGAGATGGCCTTTGGCGTGGCCGCCACCCCGCCACAACCAATTGTGGCGCCCCCAAAGCCCGACGGTGCCCGCGTGAGCAGCCCGGTGTCAGGCCTCAAGAGGCTGAGCCTCATGGATCAGGTGTCGGGAGTCGAGGCCTTCCTGCAGGCTGGCCAGCCCCCAGATCCGCACCGGGGGGCCAAGGTCATCCGCGCGGACCCGCAGGGGGGCCGCCGCCGCCACAGCTCCGAGACCTTCTCCTCGACCACCACTGTGACCCCCGTGTCCCCGTCCTTCGCTCACACCCCCAAGCGTCACAACTCGGCCTCGGTGGAGAACGTCTCTCTCAGGAAAGGCAGCGAAGGAGGCGGCGGCGGGGGCAGCAGCGGCATCCTGGGTGGGTGCGATGAGCCCCCCTCGTCGCCCCGCCAGTTGCCCCCGCCGACACCTCAGCAGGCGCGGGCCTGGACGCCGGCTCAGCCCGGCGGCGGCCTGGTCGGCTGTCCCGGGGGCACCAGCTCGCCGATGCGCCGGGAGACCTCCACCGGCTTCCAGAACGGCCTCAACTACATCGCCATCGACGTGAGGGACGAGCCGGGGCTGTCGCCGCCCCTGCAGCAGCACCCGCACGCGCAGACGGGCGACAGGAGCGCCTGGGGCCGCACCCGGAGCCTCGGGGGTCTCATCAGCGCCGTGGGCGCCGGCAGCCCTGCCGCGGTGTGCGGAGGGCCGGGCCCTGGCGCCCTGCCCGCGGCCAACGCCTACGCCAGCATCGACTTCTTGACGCACCACCTGAAGGAGGCCACGGTGGTGAAAGAGTGA

>Orc_or2 [Killer whale (Orcinus orca) Irs2]

ATGGCGAGCCCGCCGATGAACGGGCGCCCCGGGCCGGCGGGTGGCGACGGCCCCAACCTCAACCTCAACCACAACAACAACAACAACAACAGCGGCGTGCGCAAGTGCGGCTACCTGCGCAAGCAGAAGCACGGCCACAAGCGCTTCTTCGTGCTGCGCGGACCCGGAGCGGGCGGCGACGAGGCGGCGGCGACAGCGGGCGGGGGGCCGGCGCCGCAGCCGCCGCGGCTCGAGTACTACGAGAGCGAGAAGAAGTGGCGGAGCAAAGCGGGCGCCCCGAAGCGGGTCATCGCGCTCGACTGCTGCCTGAACATCAACAAGCGCGCCGACGCCAAGCACAAGTACCTGATCGCCCTCTACACCAAGGACGAGTACTTCGCAGTGGCGGCCGAGAACGAGCAGGAGCAGGAGGGCTGGTACCGCGCGCTCACCGACCTGGTCAGCGAGGGCCGAGCGAGCGCCGGCGACGCGCCCCCCGCCGCCGCCGCCGCCGCCGCCCCCTCCGCGTCCTGCAGCGCCTCCCTGCCCGGCGCCCTGGGCGGCTCGGCCGGCGCCGCCGTCGCCGCCGCGGCCGATGACAGCTACGGGCTGGTGGCGCCCGCCACGGCCGCCTACCGTGAGGTGTGGCAGGTGAACCTGAAGCCCAAAGGCCTGGGCCAGAGCAAGAACCTGACGGGCGTGTACCGCCTGTGCCTGTCGGCGCGCACCATCGGCTTCGTGAAGCTCAACTGCGAGCAGCCGTCGGTGACGCTGCAGCTGATGAACATCCGCCGCTGCGGTCACTCGGACAGCTTCTTCTTCATCGAGGTGGGCCGCTCGGCCGTGACGGGCCCCGGTGAGCTGTGGATGCAGGCAGACGACTCGGTGGTGGCGCAGAACATCCACGAGACCATCCTGGAGGCCATGAAGGCGCTCAAGGAGCTCTTCGAGTTCCGGCCGCGCAGCAAGAGCCAGTCGTCCGGCTCGTCGGCCACGCACCCCATCAGCGTCCCCGGCGCGCGTCGCCACCACCACCTGGTCAACCTGCCCCCCAGCCAGACGGGCCTGGTGCGCCGCTCGCGCACCGACAGCTTGGCCGCCACACCGCCGGCCGCCAAGTGCAGCTCGTGCCGGGTGCGCACGGCCAGCGAGGGCGACGGCGGCGTGGCGGCGGGGGCCGGGGCGGCGGGCGGCAGGCCGGTGTCGGTGGCGGGGAGCCCCCTGAGCCCGGGGCCGGTGCGCGCGCCCCTGAGCCGCTCGCACACCCTGAGCGGCGGCTGCGGCGGCCGCGCGAGCAAGGTGACGCTGGCGCCGGCAGGGGGCGCCCTGCAGCACAGCCGCTCCATGTCCATGCCCGTGGCGCACTCGCCCCCGGCGGCCGCCACCAGCCCCGGCAGCCTGTCGTCCAGCAGCGGGCACGGCTCGGGCTCCTACCCGCCGCCCCCGGGCCCGCACCCGCACATGCAGCACCCCCTGCACCCCCAGCGACCCTCCAGCGGCAGCGCCTCGGCCTCGGGCTCCCCCAGCGACCCTGGCTTCATGTCCCTGGACGAGTACGGTTCGAGCCCTGGGGACCTGAGAGCCTTCTGCAGTCTCAGGAGCAACACGCCCGAGTCCATCGCCGAGACGCCCCCGGCCAGGGACGGCAGCGGGGGCGAGCTGTACGGGTACATGACCATGGACCGGCCCCTGAGCCACTGCGGCCGACCCTACCGCAGAGTCTCCGGGGACGGGGCCCAGGACTTGGACAGGGGGCTGAGGAAGAGGACCTACTCCCTGACCACGCCTGCCCGGCAGCGGCCAGTGCCCCAGCCCTCCTCCGCGTCCCTGGATGAATACACCCTGATGCGGGCCACCTTCTCCGGCAGCTCAGGCCGCCTCTGCCCGTCGTGCCCCGCGTCCTCCCCCAAAGTGGCCTACCACCCCTACCCCGAGGACTACGGCGACATCGAGATCGGGTCGCACAGGAGCTCCAGCAGTAACCTGGGCACGGACGACGGCTACATGCCCATGACCCCCGGCGTGGCCCTCGCGGGCGGCGGCGGGAGCTGCAAGAGCGACGACTACATGCCCATGAGCCCCACCAGCGTGTCCGCCCCGAAGCAGATCCTGCAGCCGCGCGCCGCCGTCGCGGCCTTGCCCCCCACGGGAGCCGCGGGGCCCACGCCCGCGTCTGCCGCCAGCAGGGCCTTCCCGGGGAGCGGGGGCGGCTACAAGACCAGCTCCCCGGCCGAGAGCTCCCCGGAGGACAGCGGGTACATGCGCATGTGGTGCGGAGGCTCCAAGCTGTCCGTGGAGAGCGCCGCCGACGGCAGGCTGCTTCCAAACGGGGACTACCTCAACATGTCCCCCGGCGACGCGGGAGCCTCGGGCACCCCGCCCGACTTCTTCTCGGCCGCCCTGCACGCCGGCGGCGGCGGGGAGATGCTCCGGGGCGTCCCCGGCTACTGCTACAGCTCCCTGCCGCGCTCCTACAAGGCCGCCTACACCTGCAACGGGGACAACGACCAGTACGTGCTCATGAGCTCCCCCGTGGGGCGCATCCTGGAGGAGGAGCGGCTGGAGTCGACGGCCGGCCCGGGGTCCACGCAGCCAGCCGGCGCCTTCGCGGCCGGGGCAGGGGGCGGCGGCGGCCACCCGCAGCCACCCCACCCGGCGGTGCCTTCGCCCGGGAGGCCCGGTGGCAGCGGCAGCGGCCGCCTGGAGGGCTTCCTGGGCCAGCGCTGCCGGGCCACGCGGCCCACGCGCCTGTCCCTGGAGGGGCTGCAGACCCTGCCCCGCATGCACGAGTACCCCCTGCCGCCCGAGCCCAAGAGCCCGGGCGAGTACATCAACATCGACTTCGGCGAGGCGGGCGCGCGCCTGTCGCCGCCCGCGCCCCCGCTGCTGGCCTCGGCCGCCTCGTCGTCGTCGCTGCTGTCCGCCGGCAGCCCGGCCTCATCGCTGGGCTCGGGCACCCCGGGCACGAGCGGCGACAGCCGGCAGCGCTCCCCGCTCTCCGACTACATGAACCTCGACTTCAGCTCGCCCAAGTCACCCCAGCCGGGCGGCCAGGCCGGGGACCCCGTGGGCTCTCTGGACGCCCTCCTGTCCCCCGAGGCCTCCGCGTACCCGCCGCTGCCCCCGCGCCCCGCCGCCCCCTCCTCGGCCCTGCAGCCGGCGCCCCCGCCGCCCCCGCCCGGAGAGCTGTACCGCCTGCCTCCGGCACCACCCTCCCAGGGCCCTGGCGCGGCCTCCTCTCCATCCTCGGGGGCGGGCGACAGCGGCGACTACACCGAGATGGCCTTCGGCGTGGCTGCCACGCCGCCGCAACCGATCGCCGCGCCCCCGAAGCCCGACGGGGCCCGCGTGAGCAGCCCCGTGTCCGGCCTGAAGAGGCTAAGCCTCATGGACCAGTTGTCGGGGGTCGAGGCCTTCCTGCAAGCCAGCCAGCCCCCAGACCCGCACCGCGGGGCCAAGGTCATCCGTGCGGACCCGCAAGGGGGCCGCCGCCGCCACAGCTCGGAGACCTTCTCCTCGACCACCACTGTGACCCCCGTGTCCCCGTCCTTCGCCCACAACCCCAAGCGCCACAACTCGGCCTCGGTGGAGAACGTGTCTCTCAGGAAAAGCGGCGAAGGGGGCGGCGGCAGCGTCCTGGGTGGCGGTGACGAGCCCCCCACGTCCCCCCGCCAGTTGCCGCCGCCGCAACACCCACAGGCGCGGCCCTGGACGCCGAGCCAGCCCGGGGGCTTGGTCGGCTGCCCCGGGGGCAGTGGCTCGCCGATGCGCCGGGAGACTTCTGCTGGCTTCCAGAACGGCCTCAACTACATCGCCATCGACGTGAGGGACGAGCCGGGGCTGTCGCCGTCCCCGCAGCAGCCTCAGCAGCATCCTCAGACGGGAGACAAGAGCGCCTGGGGCCGGACCCGGAGCCTCGGGGGCCTCATCGGCGCGGTAGGGGCCGGCAGCACCGGCGGGGTGTGTGTGGGGCCCGGCCCTGGCGCCCTGCCCTCCGCCAGCACCTACGCCAGCCTCGACTTCTTGACGCATCACCTGAAAGAGGCCACGGTCGTGAAAGAGTGA

>Obo_ro2 [Pacific walrus (Odobenus rosmarus divergens) Irs2]
[truncated: 806,032 more chars]
