# Supplementary material for: Evolution of the vertebrate insulin receptor substrate (Irs) gene family
Source: BMC Evol Biol. 2017 Jun 23;17:148. doi: 10.1186/s12862-017-0994-z (PMC5482937; doi:10.1186/s12862-017-0994-z)
Supplement: Supplementary file 11 — This file is in Excel format. JS Divergence Scores from alignments of Irs proteins from 10 vertebrates. (PDF 499 kb) [file 12862_2017_994_MOESM7_ESM.pdf]

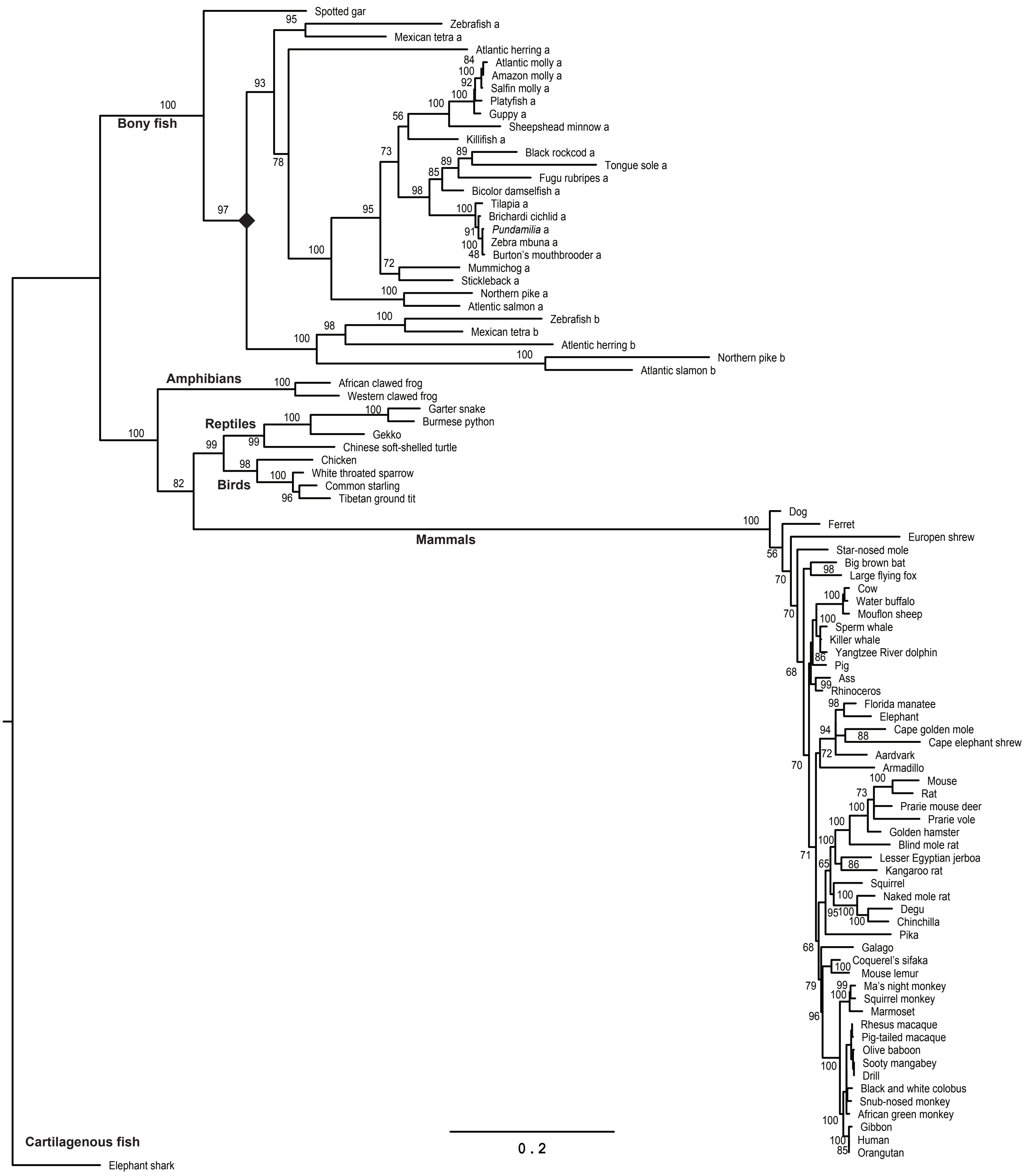

Figure S5. Phylogeny of vertebrate *Irs4* sequences. Phylogeny of Insulin receptor substrate 4 (*Irs4*) sequences from diverse vertebrate species generated by Maximum likelihood. Phylogeny was rooted with sequences from cartilaginous fish (Elephant shark). Numbers at the nodes are the proportion of bootstraps supporting the nodes. Branch lengths are proportional to the amount of inferred change, with the scale bar at the bottom. Major lineages of vertebrates are labeled on their ancestral lineage. The diamond on the fish lineage indicates a gene duplication.
