# Supplementary material for: Evolution of the vertebrate insulin receptor substrate (Irs) gene family
Source: BMC Evol Biol. 2017 Jun 23;17:148. doi: 10.1186/s12862-017-0994-z (PMC5482937; doi:10.1186/s12862-017-0994-z)
Supplement: Supplementary file 12 — This file is in PDF format. Alignment of Irs protein sequences with conserved residues highlighted. (PDF 450 kb) [file 12862_2017_994_MOESM8_ESM.pdf]

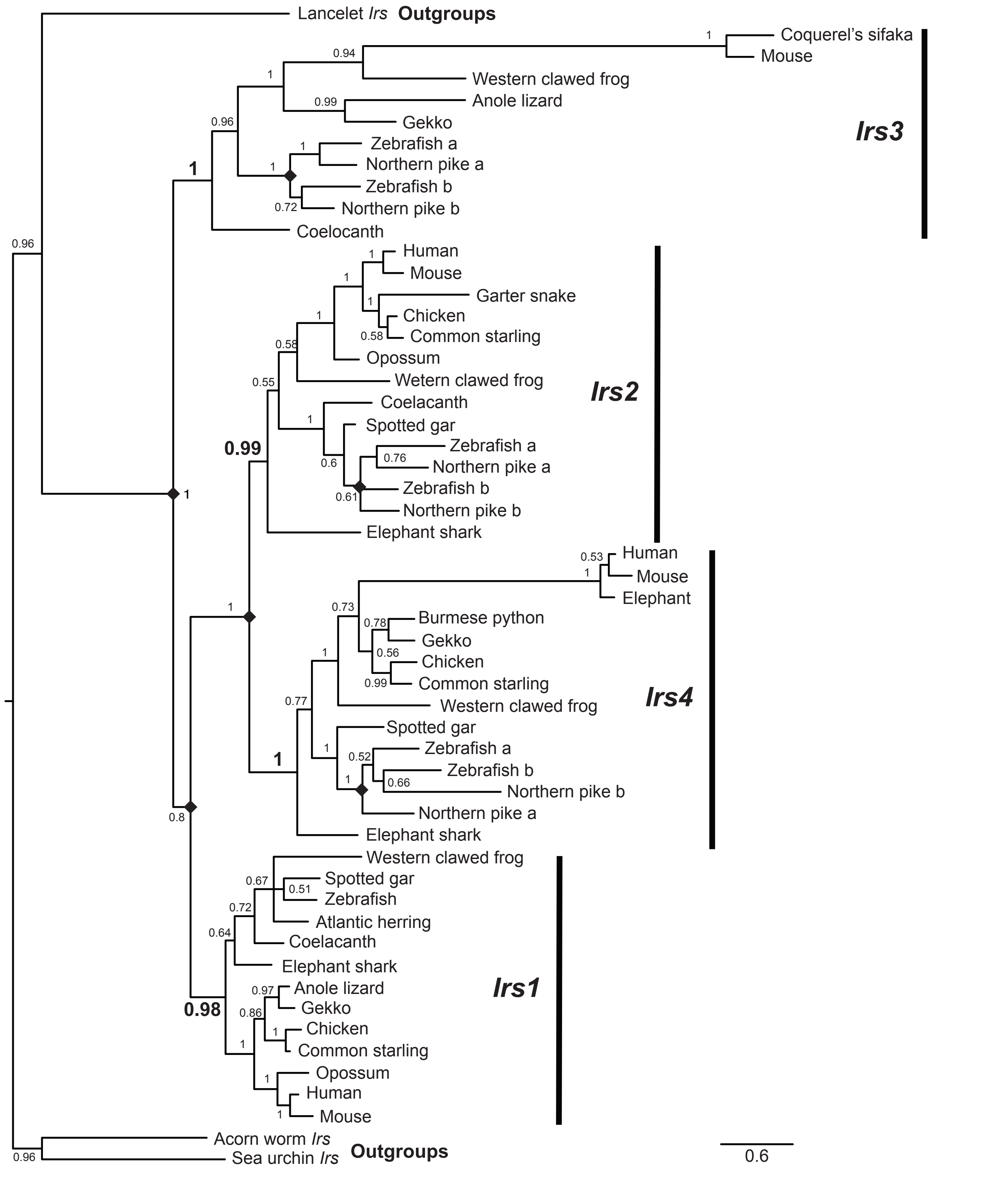

Figure S6. Phylogeny of vertebrate Insulin receptor substrate (*Irs*) gene family sequences. Phylogeny of *Irs* sequences from diverse vertebrate species generated by the Bayesian method. A similar phylogeny was generated by Maximum liklihood (see Figure 1). Phylogeny was rooted with sequences from acorn worm, sea urchin and lancelets (labeled as outgroups). Selected *Irs* sequences were chosen to represent the diversity of vertebrates, with different vertebrate *Irs* genes identified on the right. Similar results were obtained if other *Irs* sequences were used. Numbers at the nodes indicate posterior probabilities. Branch lengths are proportional to the inferred amount of change, with the scale bar at the bottom right. Diamonds indicate gene duplication events.
