# Supplementary material for: Evolution of the vertebrate insulin receptor substrate (Irs) gene family
Source: BMC Evol Biol. 2017 Jun 23;17:148. doi: 10.1186/s12862-017-0994-z (PMC5482937; doi:10.1186/s12862-017-0994-z)
Supplement: Supplementary file 13 — This file is in PDF format. ModelFinder results for the coding sequences used in the phylogenetic analyses. (PDF 224 kb) [file 12862_2017_994_MOESM9_ESM.pdf]

```
                                                Irs3:1
                                            ---------->
                       9363      9373      9383      9393
                          :         :         :         :
9362: ------------------CGCTGTATTGCCTTCGGCTCCGGATGAAGCCC   Mouse_lemur
8988: CAGCCCCCTTCCTCCAAC.A..T..............GG.A.CA.....G   Bushbaby
9269: CTGCCTC--TTCTGCAGC.A..T...........C.C.T.A.........   Human
9269: CTGCCTC--TTCTGCAGC.A..T...........C.C.T.A.........   Chimpanzee
8769: CTGCCTC--TTCTGCAGC.A..T.............C.T.A.........   Gorilla
9270: CTGCCTC--TCCTGCAGC.A..T.............C.T.A.........   Oranguatan
8798: CTGCCTC--TCCTGCAGC.A..T.............C.T.A.........   Gibbon
9350: CTGCCTC--TCCTGCAGT.A..T.............C.T.A.........   Macaque
9325: CTGCCTC--TCCTGCAGT.A................C.T.A.........   Olive_Baboon
8792: CTACCCT--TCCTGTGGCTA..T..C........AGC.T..........T   Sq_monkey
9222: CCACCCT--TCCTGTGGCTA..---........CAGC.T..........G   Marmoset
```

```
                        Irs3:1
      ------------------------------------------------->
             9403      9413      9423      9433      9443
                :         :         :         :         :
9394: GCAGGCGGCAGCCCCACGGAAAGCCCAGGGTCCAAGTGCGCCGACGTGTC   Mouse_lemur
9038: .T.A..---......----.G..T.....TG.-------.TT...T..C.   Bushbaby
9317: .........G.....GG...G....GG.AA.G-------....C....C.   Human
9317: .........G.....GG...G....GG.AA.G-------....C....C.   Chimpanzee
8817: .........G.....GG...G....GG.AA.G-------....C....C.   Gorilla
9318: A........G.....GG...G....GGCAA.G-------....T....C.   Oranguatan
8846: .........G.....GG...G...TGG.AA.G-------...AC....C.   Gibbon
9398: A.GAA....G.....GG...G....GG.AA.G-------...AC.A..C.   Macaque
9373: A.GA..A..G.....GG...G....GG.AA.G-------....C.A..C.   Olive_Baboon
8840: ........TG.....GG..C......G.A....C.A.AG..G.CT...C.   Sq_monkey
9267: ........TG.....GG..CG.....G.A....C.A.AG....C....C.   Marmoset
```

```
                        Irs3:1
      ------------------------------------------------->
             9453      9463      9473      9483      9493
                :         :         :         :         :
9444: CCTCGGCTCTCCGCTGCCCTGGGCCTACCCGGCCGACGTGCGACTCTGCG   Mouse_lemur
9074: .......C.G..A..A..G.....T.T......AA..C..A.G....CT.   Bushbaby
9360: ....A..CAG................G...T...........GT......   Human
9360: ....A..CAG................G...T...........G.......   Chimpanzee
8860: ....A..CAG................G...T......A...AG.......   Gorilla
9361: ....A..CAG................G...T...........G.......   Oranguatan
8889: ....A..CAG.T..........A...G...T...........G.......   Gibbon
9441: ....A..CAG................G...T..A...............C   Macaque
9416: ....A..CAG................G...T..A...............C   Olive_Baboon
8890: ....A..C.G................G..T......T..C..GT......   Sq_monkey
9317: ....A.TC.G................G..T.........C..GT......   Marmoset
```

```
                        Irs3:1
      ------------------------------------------------->
             9503      9513      9523      9533      9543
                :         :         :         :         :
9494: GCCACCTGCGAAAGCAGAAGTCCCAGCGCCGCCGCTTCTTCGCCCTGCGC   Mouse_lemur
9124: A.........G.....A....TA.T.............C.T.....A...   Bushbaby
9410: ..........---------------------------------------.   Human
9410: ..........---------------------------------------.   Chimpanzee
8910: ..........---------------------------------------.   Gorilla
9411: ..........---------------------------------------.   Oranguatan
8939: ........T.---------------------------------------.   Gibbon
9491: ..........---------------------------------------.   Macaque
9466: ..........---------------------------------------.   Olive_Baboon
8940: .....T....---------------------------------------.   Sq_monkey
9367: .....T....---------------------------------------.   Marmoset
```

Irs3:1

```
                9553           9563           9573                     9580
9544: ACCGACCCCCCGCGCCTCGAGTGTTATGAGAGCGA-------------GA  Mouse_lemur
9174: ...A....G.A..A....A..A...........A.TGGCCCGGCGCAA..  Bushbaby
9421: G.A.....G.....T.C........CC........-------------.C  Human
9421: G.A.....G.....T.C........CC........-------------.C  Chimpanzee
8921: G.A.....G.....T.C........CC........-------------.C  Gorilla
9422: G.A.....G.....T.C........CCC.......---------------  Oranguatan
8950: G.A.....G.....T.C........CCA.A.....-------------.C  Gibbon
9502: G.A.....G.....T.C.....C..C.........-------------TC  Macaque
9477: G.A.....G.....T.C.....C..C.........-------------TC  Olive_Baboon
8951: ..A....GG.....T.C.....A..CC........-------------.C  Sq_monkey
9378: ..A...AAG.....T.C.....A..CCT.......-------------.C  Marmoset
```

Irs3:1

```
                9590           9600           9610           9620      9630
9581: AGCAGTTCCGCGGTGGCCAAGCGCGGCCCCAGCGCAGCGTGAGCCTGGTG  Mouse_lemur
9224: ..T.T...G....CCA..G..AA.A....T.....CA.....A..C..CA  Bushbaby
9458: ..A....T....CGT...G........T..........C.........C.  Human
9458: ..A....T....CGT...G...C.........A.....C.........C.  Chimpanzee
8958: ..A....T...ACGT...G................G..C.T.......C.  Gorilla
9457: -.A....T....CGT...G...................C.........C.  Oranguatan
8987: ..A....T....CG....G.....A.......-----------.....C.  Gibbon
9539: ..A....T..G.CG....GG..A..........T....C.A.......C.  Macaque
9514: ..A....T..G.CG...AG....G.........T....C.A.......C.  Olive_Baboon
8988: ..A..C..A.A.TGTT..G.........T.........C......C..C.  Sq_monkey
9415: ..-----.TCA.TGTT..G.........T.G.G.....C......C..C.  Marmoset
```

Irs3:1

```
                9640           9650           9660           9670      9680
9631: GGCGCGTGCACCATCAGCAAGCGCGCGGACGCGCGCCAGCGCCACCTGAT  Mouse_lemur
9274: A....T....A-----------------------..........G.....  Bushbaby
9508: ..T..........C.......T....A..T..........A.........  Human
9508: ..T..........C.......T....A.............A.........  Chimpanzee
9008: ..T..........C.......T....A.............A.........  Gorilla
9506: .............C.......T....A.............A.........  Oranguatan
9026: .............C.......T....A.............A.........  Gibbon
9589: ........--------A.........A.......--....A.........  Macaque
9564: ................A.........A.......--....A.........  Olive_Baboon
9038: ...........T.C.......T....A..T..A..........G.G....  Sq_monkey
9460: ...........T.C.......T....A..T..A..........G......  Marmoset
```

Irs3:1

```
                9689           9699           9709           9719      9729
9681: CGTCCTG-TACACGCGCGACAGCAGCCTGGGCGTGGCGGCGGCCAGCGAA  Mouse_lemur
9301: ......CT.....T.............G....A......A.........T  Bushbaby
9558: ......C-.....C...............C.............AG....G  Human
9558: ......C-.....C...............C.............AG....G  Chimpanzee
9058: ......C-.....C...............C.............AG....G  Gorilla
9556: ..C...C-.....C...............C.............AG....G  Oranguatan
9076: ......C-.....CT.......A......C.........T...AG....G  Gibbon
9629: .C....C-.....C.A.....A.......C.............AG....G  Macaque
9612: .C....C-.....C.A.....A.......C.............AG....G  Olive_Baboon
9088: ....----.....C...............T....A..T..A........G  Sq_monkey
9510: ......C-.....C....A.........T.T..A.....A......C.G  Marmoset
```

```
                                 Irs3:1
      ------------------------------------------------->
            9739      9749      9759      9769      9779
9730: GCGGAGCAGCAGGCGTGGTACTGCGCCCTGCTCGAGGTGCGCGCCGCGGC  Mouse_lemur
9351: ......TT.T......AC...A.......A...T...C.T.......CA.  Bushbaby
9607: .................A...AA.A............A.....GT.AT..  Human
9607: .................A...AA.A............A.....GT.AT..  Chimpanzee
9107: .................A...AA.A............A.....GT.AT..  Gorilla
9605: .................A...AA..............A.....GT..C..  Oranguatan
9125: .................A...AA...........G..A.....GT..C..  Gibbon
9678: .................A...AA..............C.....GT..C..  Macaque
9661: .................A...AA..............C.....GT..C..  Olive_Baboon
9134: ......G..............AA..............CA.TA.G...C..  Sq_monkey
9559: ..-...G...........C..AA..T.......CG..CA.TA.G...C..  Marmoset

     Irs3:1
      -->
            9789      9799      9809      9816
9780: TGGTGAGGCCCGAGCCCTGGGCTGCAAGGGG---CTGGGGGGATCGTGG-  Mouse_lemur
9401: CT...-------.....C........G....---TCA...A..C...A.G  Bushbaby
9657: C................C.....T.CG....--C..C.....C.TC...C  Human
9657: C................C.....T.CG....--C..C.....C.TC...C  Chimpanzee
9157: C................C.....T.CG....--C..C.....C.TC...C  Gorilla
9655: C..C..A..........C.....T.CG....--C.CC.....C.TC...C  Oranguatan
9175: C................CA....T.CG....--C.CC.....C.T....C  Gibbon
9728: C---------...........T...CG...A--C.CC.....C.T....C  Macaque
9711: C.----------.........T...CG...A--C.CT.....C.T....C  Olive_Baboon
9184: CA........G.....TG.......C..A.C--CTCC...C.C.T....C  Sq_monkey
9608: CA.........-..T..C.......CGA...GCC.CC.....C.T....C  Marmoset

            9834                9843      9852      9862
9826: -GGGGTTTATCTGTAG-----------CCCAGGTCA-CGAAAATTCAAAC  Mouse_lemur
9441: TTT.......TCC...CCTGTCTCTCCT........CT....G.......  Bushbaby
9705: T.T.....C..C..G.CTCGCCCCTCC........GC.....G.......  Human
9705: T.T.....C..C..G.CTCGCCCCTCC........GC.....G.......  Chimpanzee
9205: T.T.....C..C..G.CTCGCCCCTCC........GC.....G.......  Gorilla
9703: T.T........C..G.CTCGCCCCTCC........GC.....G.......  Oranguatan
9223: T.T.....C..C..G.CTCGCCCCTCC........GC.....G.......  Gibbon
9767: T.T.....C..C..G.CTCGCCCCTGC.........C.....G.......  Macaque
9749: T.T.....C..C..G.CTCGCCCCTGC.........C.....G.......  Olive_Baboon
9232: T.T.....C....AG.CTCGCCCCTCC.........C.....G.......  Sq_monkey
9657: T.T.....T..C..G.ATCGCCCCTCC.........C.....G.......  Marmoset

            9871      9881      9891      9897      9907
9863: ATT-AAGTGTTTACCTGTCTTCAGCTCTCT-GG-GCA--GTGATTGTAAC  Mouse_lemur
9491: ...A..-CA..C..AA.....A....G.TA-..-..CAA....C.C....  Bushbaby
9755: ...A...G.---G.AG..T.AG..T....C-..-...              Human
9755: ...A...G.---G.AG..T.AG..T....C-..-...              Chimpanzee
9255: ...A...G....G.AG..T.AG..T....C-..C..G--...GC.      Gorilla
9753: ...A...G....G.AG..T.AG..T....C-C.-...              Oranguatan
9273: ...A...G....G.AG..T.AG..T....C-..-...              Gibbon
9817: ...A...G....G.A...T.AG..T....C-..-...              Macaque
9799: ...A...G....G.A...T.AG..T....C-..-...              Olive_Baboon
9282: ...A...GA...G.AG..T.AG..T....                      Sq_monkey
9707: ...A...G....G.AG..T.AG..T....CT..-..               Marmoset
```

```
                                                    Irs3:2
                                                  ------->
             9917      9927      9937      9947      9957
 9908: CTCCCGGGCCAGGAGCGCTTTGTGACCAGCCCATTGTCTCCCTAGGACCC  Mouse_lemur
 9538: ...T...T..G.TGC.C.C..........A......AA....C.A.T.A.  Bushbaby

                               Irs3:2
       -------------------------------------------------->
             9967      9977      9987      9997     10007
 9958: AGCTCCTACGAGGACCCTGGGACCTGGATCCTCTCTCCGTTCCAGGACGT  Mouse_lemur
 9588: ..T..-----------------..AC...T..--....A...........  Bushbaby
 9580:                        ..........G............TG..  Sq_monkey
10007:                        ..........G............TG..  Marmoset

                               Irs3:2
       -------------------------------------------------->
            10017     10027     10037     10047     10057
10008: CTGGCCCGTGACGCTGCGGCCCAAAGGGCTGGGGCGGACCCGAGGCCTGG  Mouse_lemur
 9619: ....T.T........AT.......T.......A.......T.........  Bushbaby
 9607: .....T.C................GTT......-----------------  Sq_monkey
10034: .....T.C................GTT....C..G.............A.  Marmoset

                               Irs3:2
       -------------------------------------------------->
            10067     10077     10087     10097     10107
10058: GCAGCGGCGGCTACCGCTTGTGCCTGGGTTCGGGGGCTCTGAGTCTGCTG  Mouse_lemur
 9669: ....T..T......TT.CC..................C.....C.....A  Bushbaby
 9640: ----T............C.....T....----------------------  Sq_monkey
10084: .---A..........A.C...A.T....----------------------  Marmoset

                               Irs3:2
       -------------------------------------------------->
            10117     10127     10137     10147     10157
10108: CGGAAGCCCAGGGGCAGGGGCTTCCGGGATAACCGGGCATTGCCGCCGCC  Mouse_lemur
 9719: T........CA.A.T..-.....G.A...C.C.GA.....CA....A...  Bushbaby
 9664: ------.T.CAC.....A..T.C..T...---------------------  Sq_monkey
10109: ------.T.C.......A..T.C..T...---------------------  Marmoset

                               Irs3:2
       -------------------------------------------------->
            10167     10177     10187     10197     10207
10158: CGCCCTGCGCCTGCCCCTGCTCAGCGTGCGCCGCTGCGGCCACGCAGACT  Mouse_lemur
 9768: T......TA..G............A.G.T...C...T.............  Bushbaby
 9688: -....G..A...............T.A...GG.....A.....AGG....  Sq_monkey
10133: -.......A...............T.A....T...........C...G.-  Marmoset

                               Irs3:2
       -------------------------------------------------->
            10217     10227     10237     10247     10257
10208: CTTTCTTCTTCCTGGAGCTCGGCCGCTCGGCACCCACGGGTCCCGGGGAG  Mouse_lemur
 9818: ..---...........T..T....T..G.TTG..T.T.T..A.T......  Bushbaby
 9737: G.C--.................TT.......---...---...G......  Sq_monkey
10181: -----..T.......T.......T....A..---.G.---....A.....  Marmoset
```

```
                                 Irs3:2
       ------------------------------------------------->
            10267     10277               10294     10304
10258: CTGTGGTTGCAGGCGCCCGACGCTGTGG---TGGCCCAAAACATTCACGA  Mouse_lemur
 9865: ....A.C..............-.-....---..A......G.....GT..  Bushbaby
 9779: ......C.....AA.......A.-....CTA.........G.........  Sq_monkey
10220: ...A..C......A.......A.-.CA.CGA.........G.........  Marmoset

                                 Irs3:2
       ------------------------------------------------->
            10314     10324     10334     10344     10354
10305: GACCGTCCTGGCCGCCATGAAGCGACTCGGGAGCGGTGGGGTCGGTGCCA  Mouse_lemur
 9910: ...A......---------------------------------A...G..  Bushbaby
 9828: ....A......TG.....A...T........GT......A....C..G..  Sq_monkey
10269: ....T....C.TG.........T........G.......A..G.C..G..  Marmoset

                                 Irs3:2
       ------------------------------------------------->
            10364     10374     10384
10355: GGGCCGAACCACTGCCCAGGGATTGTCCAACGAGCGC-------------  Mouse_lemur
 9927: ...AT.T....G.......A...CCC..G.....G..-------------  Bushbaby
10136:             ...T......CCC...T.T....A.-------------  Human
10119:             ...T......CCC...T.T....A.-------------  Chimpanzee
 9624:             ...T......CCC...T.T....A.-------------  Gorilla
10107:             ...T....A.CCC...T.T....A.-------------  Oranguatan
 9625:             ...T......CCT...T.T....A.-------------  Gibbon
10176:              ..T......CCC...T.T....AT-------------  Olive_Baboon
 9878: .A..T..GT..........A..CCA.G.G.T......-------------  Sq_monkey
10319: .A..T..GT..........A-.CC..G.G.T....ATGTAGCTGTGCAGA  Marmoset

                                 Irs3:2
       ------------------------------------------------->
            10392     10402     10412     10422     10431
10392: ---------CTCCAGACTCTCAGTCCCCCAACCTTATGAG-ACCTCGGTC  Mouse_lemur
 9964: ---------..A.......----............--AT.A.A.....C.  Bushbaby
10161: ---------..T.......C.T..T.....T..C.--...-...C.A.CT  Human
10144: ---------..T.......C.T........T..C.--...-...C.A.CT  Chimpanzee
 9649: ---------..T.......C.T...T....T..C.--...-...C.A.CT  Gorilla
10132: ---------..T.......C.T........T..C.--...-...C.ACCT  Oranguatan
 9650: ---------..T.......C.T........T..C.--...-...C...CT  Gibbon
10200: ---------..T.......-------....T..C.--...-...C...CT  Olive_Baboon
 9915: ---------..T.......C.T........T..C.--...-.......CT  Sq_monkey
10368: GTGTTCAGA..T.......C.T........T..C.--...-.......CT  Marmoset

                                 Irs3:2
       ------------------------------------------------->
            10441     10451     10461     10471     10481
10432: TCCGCGGCCCAATCAAGCAGCCTGAGCCGTCGGGGGTGCCTGGGTGAGAG  Mouse_lemur
 9999: ..T.........G.................T..A.........A......  Bushbaby
10199: ..T....A...G.....TG............CA.....T.....A...G.  Human
10182: ..T....A...G.....TG............C......T.....A...G.  Chimpanzee
 9687: ..T....A...G.....TG............C......T.....A...G.  Gorilla
10170: ..T........G......G............A......T.....A...G.  Oranguatan
 9688: ..T..A.....G......G...................T.....A...G.  Gibbon
10231: ..T........G.....TG.........A..A......T...C.A...G.  Olive_Baboon
 9953: .TT....G.T.G.....TG.........A.T.......T.....A...G.  Sq_monkey
10415: .TT......T.GC...CTG.........A.T.......T.A...AC..G.  Marmoset
```

Irs3:2

```
                 10491     10501     10511     10521     10531
10482: GATCAAGCAAGCAACCCTTGGGACCCCCGCGAGGCTAGGGGCGGCAGCCT  Mouse_lemur
10049: ....C.....A....T..CA..GA...AA.....A...A..T.T....T.  Bushbaby
10249: ....G........G.....AA....TTG........G...A.A.......  Human
10232: ....G........G.....AA....TTG........G...A.A.......  Chimpanzee
 9737: ....G........G.....AA....TTG........G.....A.......  Gorilla
10220: .............G.....AA....TTG........G.....A.....T.  Oranguatan
 9738: ....G.T......G.....AA....TTG........G.....A.......  Gibbon
10281: ...                                                 Olive_Baboon
10286:      ........G....CAA....TTG..A.....G..T..A.......  Olive_Baboon
10003: A.C.C........GT...GAA..T.TTG..A.A...GT.A.G....A...  Sq_monkey
10465: A.C.C......T.GT...GAA..T..T-..A.A...G..A.G..T.A...  Marmoset
```

Irs3:2

```
                 10541     10551     10561     10571     10581
10532: TGCACTCTAAGGCGCTGGATCAGGGGGGGAGCTACATAAACATGGGAGCC  Mouse_lemur
10099: ...G........G.A....-TC..CT...GAG.G............----  Bushbaby
10299: .CT..C......G.------.T...T...G.........C..A......A  Human
10282: .CT..C......G.------.G...T...G.........C..A......A  Chimpanzee
 9787: .CT..C.C....G.------.G...T...G.........C..A......A  Gorilla
10270: CCT..C.C..T.G.------.G...T...G.........C..A......G  Oranguatan
 9788: CCT..C.C....G.------.G..AT...G.........C..A.....TG  Gibbon
10331: CCT..C.C....G.------.....T...G.........C..A.....TG  Olive_Baboon
10053: ..T..C.C....G.------.G...T...G.........C..A.....TG  Sq_monkey
10514: ..T..C.CG...G.------TG...T...G.........C..A.....TG  Marmoset
```

Irs3:2

```
                 10591     10601     10611     10621     10631
10582: GGGAGTGACTACGAGCCCATGGGGGGCGGCGCAGCGGGCGGCTACACGGT  Mouse_lemur
10144: -------------............C.....A.T.....A.A...GTA..  Bushbaby
10343: ...........T........                                Human
10326: ..C.................                                Chimpanzee
 9831: ............A.......                                Gorilla
10314: ....................                                Oranguatan
 9832: ............C.......                                Gibbon
10375: ........................                            Olive_Baboon
10097: ..T.........A......CA..                             Sq_monkey
10558: ..T.........A.......A..                             Marmoset
```

Irs3:2

```
                 10641     10651                             10669
10632: GATGGCGTCCCCAGGCCTTTGTCCGGCCA------------CTCCTGCTC  Mouse_lemur
10181: ...A..T.......A-........A....CCTCCAGCGCCG....C....  Bushbaby
```

Irs3:2

```
                 10679     10689                             10697
10670: CTGGCCAACCTCTCCAGGAC----------------------TGGGGGGA  Mouse_lemur
10230: TCCA...GT.G..T.....T----------------------......A-  Bushbaby
10345:               ....GTGGGGGCTACATAACCAAGGGAG.....A.-  Olive_Baboon
```

Irs3:2

```
                   10707     10716     10726     10736     10746
10698: CACCGAATACGTGCCC-ATGAACCGCTTTCCGCCAGGGTCCTTTTCCTTG  Mouse_lemur
10257: ....A....TA.....A...G.TT...C..T...............T.C.  Bushbaby
10380: ---T..C....A....-...G-----------------------------  Olive_Baboon
```

Irs3:2

```
                   10756     10766     10776     10786     10796
10747: AGCTCGCTGCACCACTCCTACAAGTCCGGAGCTGGGAAGCCTGGACCCGG  Mouse_lemur
10307: .....C....C..GT..........T..-----------------...T..  Bushbaby
10359:            ...T..........T..-.T.CA.G.........A.A.  Human
10342:            ...T..........T..-.T.CA.G.........A.A.  Chimpanzee
 9847:            ...T..........T..-.T.CA.G.........A.A.  Gorilla
10330:            ...T......G.......-.T.CA.G.........A.A.  Oranguatan
 9848:            ...T..............-.T.CA.G.........A.A.  Gibbon
10397: --------.G....T..........T..-...CA.G...........A.  Olive_Baboon
10121:          ..T.TC...........A-.T....G..TT.......A.  Sq_monkey
10582:          ..T.TC...........A-.T....G..TT.......A.  Marmoset
```

Irs3:2

```
                   10806
10797: GCTCCGGAGC----------------------------------------  Mouse_lemur
10342: ......AG.G----------------------------------------  Bushbaby
10397: ..C.A....T----------------------------------------  Human
10380: ..C.A....T----------------------------------------  Chimpanzee
 9885: ..C.A....T----------------------------------------  Gorilla
10368: ..C.A....T----------------------------------------  Oranguatan
 9886: ..C.A....T----------------------------------------  Gibbon
10438: ..CAA.....----------------------------------------  Olive_Baboon
10160: ..C.A..G..----------------------------------------  Sq_monkey
10621: ..C.A..G.TACCCATCGTGGTGTGGGGGGTGGTTGGGGATGGCAGGGG  Marmoset
```

Irs3:2

```
                   10815     10825     10835     10845     10855
10807: -GCCCATCGCAGCGCTGGGGGCAGCTGGGGACTGCCAAGGGCTCAGCCCT  Mouse_lemur
10352: -.........T...T..A...T.........CATT.GA....G....T.  Bushbaby
10407: -.........GAT.TG....CTG.......TG.GT.G..A......G..  Human
10390: -.........GAT.TG....CTG.......TG.GT.G..A......G..  Chimpanzee
 9895: -..........AT.TG----------.....TG.GT.G..A......G..  Gorilla
10378: -.......ATGAT.TG....CTG........G.GT.G..A......G..  Oranguatan
 9896: -.........GAT.TG....CTG........G.GT.G..A.....GG..  Gibbon
10448: -.......A.GAT.TG....CTG........G.GT.G.........G..  Olive_Baboon
10170: -........TGAT..A.....TG........G.GTGG.........A..  Sq_monkey
10671: C........TG.T.TG.....TG.......TG.G..G.........A..  Marmoset
```

```
                               Irs3:2
       ------------------------------------------------------------------>
                10864     10874     10884     10893     10903
                    :         :         :         :         :
10856: GTTCCC-TGTCGCCGTCCGAGCTGGCCGGGAAGC-ACGTGTACACCGAGT   Mouse_lemur
10401: ......TC.C...G...G...T....GA.....T-..AC..G..TGT...   Bushbaby
10456: .C...--..C.C..C..A........TA..G..TG.A....G....A...   Human
10439: .C...--..C.C..C..A........TA..G..TG.A....G....A...   Chimpanzee
 9934: .C...--..C.C..C..A........T...G..TG.A....G....A...   Gorilla
10427: .C...--..CAC..C..A........T...G..TG.A....G....A...   Oranguatan
 9945: .C...--..C.C..C..A........T...G..TG.A....G....A...   Gibbon
10497: .C...--..G.C..C..G........T...G..G-.A....G..T.A...   Olive_Baboon
10219: .C...--.A..C.GC.T.........T...G..TT.A....G..T.A.A.   Sq_monkey
10721: .C...--..C.C..C.TGC.......T...G..TT.A....G.CT.A...   Marmoset

                               Irs3:2
       ------------------------------------------------------------------>
                10913     10923                         10941     10951
                    :         :                             :         :
10904: ACGCGGCCCCAAACTGCGTAGGAATAGCT--TCTGCCGGGCCAGAGCCCC   Mouse_lemur
10450: ..CA.......GG....AG...GTATTGCCGA.......A..G...GT..   Bushbaby
10504: TAT........G.GA..C.C....CC.GC--A.C...A............   Human
10487: TAT.C......G.GA..C.C....C..GC--A.C...A............   Chimpanzee
 9982: TAT........G.GA..C.C....C..GC--A.CA..A............   Gorilla
10475: TAT........G.GA..C.CA...C..GC--A.C...A..------...T   Oranguatan
 9993: TAT........GCGA..C.CA...C..GC--A.C...A.T..........   Gibbon
10544: TAC......--G.GA..C.CCA..C..TC--A.C...A............   Olive_Baboon
10267: TT..A.....GG.GA..C.G....C.TG.--A.CA..A...TG.......   Sq_monkey
10769: TT..A..T...G.GA..C.G....C.TGC--A.CA..A...TG.......   Marmoset

                               Irs3:2
       ------------------------------------------------------------------>
                10961     10971     10981     10991     11001
                    :         :         :         :         :
10952: CAGACGGCCATCTCAACTACGTCGACTTGGACCTGGTCCCTCCTCTGGAG   Mouse_lemur
10500: .....A..TGC...T.......-----....T......G...T...T...   Bushbaby
10552: TGACGA..TGC....G.........TC............T.T......G.   Human
10535: TGACGA..TGC....G.........TC............T.T......G.   Chimpanzee
10030: TGACGA..TGC....G.........TC............T.T......G.   Gorilla
10518: GTTCGA..TGC....G......----C............T.T......G.   Oranguatan
10041: TGACAA..TGC....G...T..T..TC..........T.T.T......G.   Gibbon
10590: TGACGA..TGC....G...T..T...C............T.T......G.   Olive_Baboon
10315: TGAC-A..TGC....GT.....G...C..............T......G.   Sq_monkey
10817: TGAC-A..TGC....GT.....G...C..............T......G.   Marmoset

                               Irs3:2
       ------------------------------------------------------------------>
                11011     11021     11030     11040     11050
                    :         :         :         :         :
11002: GCGCGCGGCGACGTCCCCGGGGTCC-GCAAGCTCCTCCCGCACAGCTACG   Mouse_lemur
10545: .AA....A..TG...T.......GG-...T.........A..T..T...T   Bushbaby
10602: ..A.TT..T.........A...C..-A..C.....G...A..T.....T.   Human
10585: ..ATTT..T.........A...C..-...C.....G......T.....T.   Chimpanzee
10080: ..A.TT..T.........A...C..-...C.....G......T.....T.   Gorilla
10564: ..A.TT..A.........A...C..-...C.....G......T.....T.   Oranguatan
10091: ..A.TT..T.........A......-...C.....G......T.....T.   Gibbon
10640: ..A.TT..T..T......A...C.T-...C.....G.....GT......A   Olive_Baboon
10364: ..A.T.T....A..........C..T.A.CA...TG..T..........A   Sq_monkey
10866: ..A.T.T....A....T...CCC.T-.A.CA...TGT.T..........T   Marmoset
```

```
                    Irs3:2
         ------------------------------->
                11060     11070     11080     11090
                    :         :         :         :
11051: CGCGCATCGAGTTCTAGAACCTCCAGGAGGCGCAGGTGAGGAAACCCGG-   Mouse_lemur
10594: T.T.T..G..A...........G..........C.........G...G..C   Bushbaby
10651: ..-.TGAG...AC..GAG..--.....CA..T.-----------------   Human
10634: .A-.TGAG...AC..GAG..--.....CA..T.-----------------   Chimpanzee
10129: ..-.TGAG...AC..GAG..--.....CA..T.-----------------   Gorilla
10613: ..G....T..........G.TC..G.....T..T.........G.T.T.AG   Oranguatan
10140: ..G....G..........G.TC.TG.....T..CA.......G...T.AG   Gibbon
10689: .AG....T............TC..C........C.........G...T.AG   Olive_Baboon
10414: ..-....T.CA.....A.G.TC.TG......A.CA.......G...T.AG   Sq_monkey
10915: ..G....T..AG......G.TC..G......A.CA.......G...T.AC   Marmoset

                          11104     11114         11128
                              :         :             :
11100: ----------------TCAGCCGCGGATGCACGAGGA-----CGGGG-GA   Mouse_lemur
10644: AG-------------..C.GTC.....TAGTTG.C.GATGGAA....A.G   Bushbaby
10681: ---------------..TC.T..T..CTG.T.G...GGTGGTT.A..-..   Human
10664: ---------------..TC.T..T..CTG.T.G...GGTGGTT.A..-..   Chimpanzee
10159: ---------------..TC.T..T..CTG.T.G...GGTGGTT.A..-..   Gorilla
10663: ACCCAGGCAGCTC--..TC.T..T..CTG.T.G...G-TGGTT.A..-..   Oranguatan
10190: ACCCAGGCAGCTC--..TC.T..T..CTG.T.G....GTGGTT.A..-..   Gibbon
10739: ACCCAGGCAGCTC--..TC.T..T..CTG.TTG...GGTGGTG.A..-..   Olive_Baboon
10463: ACCTGGGTATC----..TC....T..CTG.T.TT.AG--GGTT.ACA-..   Sq_monkey
10965: ACCTGGGTCTCTCTC..TC....T..CTA...GT.AG--GGTTCAC.-..   Marmoset
```

Figure S7. Alignment of *Irs3* genomic sequences. An alignment of *Irs3* genomic sequences from diverse primates was generated using MultiPipMaker [56,57]. Dots (.) in the alignment indicate bases identical to those in the top line, while dashes (-) are gaps introduced to maximize similarity. Blank spaces are regions that have no similarity to the mouse lemur (labeled Mouse_lemur) that was used as the master sequence for the alignment. Annotation of the *Irs3* gene is shown above the alignment, based on alignment of the mouse lemur gene with those from other diverse mammals (results not shown). The arrows above the sequence represent the coding exons and are labeled Irs3:1 and Irs3:2 and starts from the initiator ATG and ends with the TAG termination codon. Sq_monkey is squirrel monkey.
