# Supplementary material for: Evolution of the vertebrate insulin receptor substrate (Irs) gene family
Source: BMC Evol Biol. 2017 Jun 23;17:148. doi: 10.1186/s12862-017-0994-z (PMC5482937; doi:10.1186/s12862-017-0994-z)
Supplement: Supplementary file 3 — This file is in Word format. Coding sequences for full-length and near full-length Irs genes from diverse vertebrates and outgroups. (PDF 200 kb) [file 12862_2017_994_MOESM12_ESM.pdf]

```
                                                                                                               >>>>>                                              Y    Pleckstrin Homology (PH) Domain
Mus_mu1    ---------- ---------- ---------- ---------- ---------- ----MASPP- ---------- ---------- -D---TDGFS DVRKVGYLRK PKSMHKRFFV LRAASEAGGP ---------- ---ARLEYYE NEKKWR--HK SSA------- ---------- PKRSIPLESC FNINKRADSK NKHLVALYTR
Rat_no1    ---------- ---------- ---------- ---------- ---------- ----MASPP- ---------- ---------- -D---TDGFS DVRKVGYLRK PKSMHKRFFV LRAASEAGGP ---------- ---ARLEYYE NEKKWR--HK SSA------- ---------- PKRSIPLESC FNINKRADSK NKHLVALYTR
Mes_au1    ---------- ---------- ---------- ---------- ---------- ----MASPP- ---------- ---------- -D---TDGFS DVRKVGYLRK PKSMHKRFFV LRAASEAGGP ---------- ---ARLEYYE NEKKWR--HK SSA------- ---------- PKRSIPLESC FNINKRADSK NKHLVALYTR
Mic_oc1    ---------- ---------- ---------- ---------- ---------- ----MASPP- ---------- ---------- -D---TEGFS DVRKVGYLRK PKSMHKRFFV LRAASETGGP ---------- ---ARLEYYE NEKKWR--HK SSA------- ---------- PKRSIPLESC FNINKRADSK NKHLVALYTR
Per_ma1    ---------- ---------- ---------- ---------- ---------- ----MASPP- ---------- ---------- -D---TDGFS DVRKVGYLRK PKSMHKRFFV LRAASEAGGP ---------- ---ARLEYYE NEKKWR--HK SSA------- ---------- PKRSIPLESC FNINKRADSK NKHLVALYTR
Pro_co1    ---------- ---------- ---------- ---------- ---------- ----MASPP- ---------- ---------- -E---TDGFS DVRKVGYLRK PKSMHKRFFV LRAASEAGGP ---------- ---ARLEYYE NEKKWR--HK SSA------- ---------- PKRSIPLESC FNINKRADSK NKHLVALYTR
Mic_mu1    ---------- ---------- ---------- ---------- ---------- ----MASPP- ---------- ---------- -E---TDGFS DVRKVGYLRK PKSMHKRFFV LRAASEAGGP ---------- ---ARLEYYE NEKKWR--HK SSA------- ---------- PKRSIPLESC FNINKRADSK NKHLVALYTR
Ovi_arm1   ---------- ---------- ---------- ---------- ---------- ----MASPP- ---------- ---------- -E---TDGFS DVRKVGYLRK PKSMHKRFFV LRAASEAGGP ---------- ---ARLEYYE NEKKWR--HK SSA------- ---------- PKRSIPLESC FNINKRADSK NKHLVALYTR
Orc_or1    ---------- ---------- ---------- ---------- ---------- ----MASPP- ---------- ---------- -E---TDGFS DVRKVGYLRK PKSMHKRFFV LRAASEAGGP ---------- ---ARLEYYE NEKKWR--HK SSA------- ---------- PKRSIPLESC FNINKRADSK NKHLVALYTR
Xen_tr1    ---------- ---------- ---------- ---------- ---------- ----MASPT- ---------- ---------- -DPQAQENFS DVRKVGYLRK PKSMHKRFFV LRSASES-GL ---------- ---ARLEYYE NEKKWR--HK SGA------- ---------- PKRSIPLESC FNINKRADSK NKHLVALYTK
                                                                      $                                                  $$ $$$$                                 $$  $   $$  $                                 $ $$ $       $ $
Mus_mu2    ---------- ---------- ---------- ---------- ---------- ----MASAPL P----GPPAS AGGDGP--NL -NNNNNNNNH SVRKCGYLRK QKHGHKRFFV LRGPGTGGDE --ASAAGGSP PQPPRLEYYE SEKKWR--SK AGA------- ---------- PKRVIALDCC LNINKRADAK HKYLIALYTK
Rat_no2    ---------- ---------- ---------- ---------- ---------- ----MASAPL P----GPPAS AGGDGP--NL NNNNNNNNNH SVRKCGYLRK QKHGHKRFFV LRGPGTGGEE --AAAAGGSP PQPPRLEYYE SEKKWK--SK AGA------- ---------- PKRVIALDCC LNINKRADAK HKYLIALYTK
Mes_au2    ---------- ---------- ---------- ---------- ---------- ----MASAPL P----GPPAP AGGDGP--NL -NNNNNNNNH SVRKCGYLRK QKHGHKRFFV LRGPGTGGDE --AAAGGGSP PQPPRLEYYE SEKKWR--SK AGA------- ---------- PKRVIALDCC LNINKRADAK HKYLIALYTK
Mic_oc2    ---------- ---------- ---------- ---------- ---------- ----MASAPL P----GAPAP AVGEGP--NL -NNNNNNNNH SVRKCGYLRK QKHGHKRFFV LRGPGTGGDD --AAAGGGSP PLPPRLEYYE SEKKWR--SK AGA------- ---------- PKRVIALDCC LNINKRADAK HKYLIALYTK
Per_ma2    ---------- ---------- ---------- ---------- ---------- ----MASAPL P----GPPAP AGGDGP--NL --NNNNNNNH SVRKCGYLRK QKHGHKRFFV LRGPGTGGDE --AAAGGGSP PLPPRLEYYE SEKKWR--SK AGA------- ---------- PKRVIALDCC LNINKRADAK HKYLIALYTK
Pro_co2    ---------- ---------- ---------- ---------- ---------- ----MASPPL H----GPPGP ASGDGP--NL --NNNNNNNH GVRKCGYLRK QKHGHKRFFV LRGPGAGSDE --ATAGGGSA PQPPRLEYYE SEKKWR--SK AGA------- ---------- PKRVIALDCC LNINKRADAK HKYLIALYTK
Mic_mu2    ---------- ---------- ---------- ---------- ---------- ----MASPPL H----GPPGP ASGDGP--NL --NNNNNNNH GVRKCGYLRK QKHGHKRFFV LRGPGAGSDE ATATAGGGSA PQPPRLEYYE SEKKWR--SK AGA------- ---------- PKRVIALDCC LNINKRADAK HKYLIALYTK
Ovi_arm2   ---------- ---------- ---------- ---------- ---------- ----MASPPE H----GPPGP AGGDGP--NL -NNNNNNNNH SVRKCGYLRK QKHGHKRFFV LRGPGAGGDE ----AGGGPA PQPPRLEYYE SEKKWR--SK AGA------- ---------- PKRVIALDCC LNINKRADAK HKYLIALYTK
Orc_or2    ---------- ---------- ---------- ---------- ---------- ----MASPPM N----GRPGP AGGDGPNLNL -NHNNNNNNS SVRKCGYLRK QKHGHKRFFV LRGPGAGGDE AAATAGGGPA PQPPRLEYYE SEKKWR--SK AGA------- ---------- PKRVIALDCC LNINKRADAK HKYLIALYTK
Xen_tr2    ---------- ---------- ---------- ---------- ---------- ----MASP-- H----SPVQP LLPVSL--NL -NHNNNNNSS SVKKCGYLRK QKHGHKRFFV LREAGEGCPA ---------- ---ARLEYYE SEKKWR--SK SAA------- ---------- AKRVISLDSC LNINKRADAK NKYLIALYTK
                                                                                                       $$$$       $ $ $$ $$$ $$$$$                               $$$ $$  $                                     $  $ $$        $
Mus_mu3    ---------- ---------- ---------- ---------- ---------- -----MKPAG T----GPTVS SGGECTDVSL GSPFPWPCLP DVRLCGHLRK QKSQRRRFFV LRADP----- ---------- ---PRLECYE SEKKFLASGC RPP------- ---------R PRRTVSLEGA CTISKRADAR QRHLIVIYTS
Rat_no3    ---------- ---------- ---------- ---------- ---------- -----MKPAG T----GPTVS SGGESTDVSL GPPFPWPCPP DVRLCGHLRK QKSQRRRFFV LRADP----- ---------- ---PRLECYE SEKKFLASGC RPP------- ---------R PRRTVSLEGA CTISKRADAR QRHLIVIYTS
Mes_au3    ---------- ---------- ---------- ---------- ---------- -----MKPAG A----GPAVS PEAESPDAFL GPPFPWPCPG DVRLCGHLRK QKSQRRRFFV LRADP----- ---------- ---PRLECYE NEKKFLASGC RPP------- ---------R PRRSVSLEGA CTISKRADAR QRHLIVVYTS
Mic_oc3    ---------- ---------- ---------- ---------- ---------- -----MNPAG T----GPTVS PGAESTDVSL GPPFPWPCPP DVRLCGHLRK QKSQRRRFFV LRADP----- ---------- ---PRLECYE SEKKFLASGC RPP------- ---------R PRRSVSLEGA CTISKRADAR QRHLIVVYTS
Per_ma3    ---------- ---------- ---------- ---------- ---------- -----MKPAG SGPTAGPTVS PGAESTDASL GPPFPWPCPP DVRLCGHLRK QKSQRRRFFV LRADP----- ---------- ---PRLECYE SEKKFLAGGC RPP------- ---------R PRRSVSLEGA CTISKRADAR QRHLIVVYTS
Pro_co3    ---------- ---------- ---------- ---------- ---------- -----MKPAG G----GPTAS PESKCANTSL GLPLPWACPD DVRLCGHLRK QKSQRRRFFA LRTDP----- ---------- ---PRLECYE SEKQFRSGRA ---------- ---------R PQRSVSLVGA CTISKRADAR QRHLVVLYTR
Mic_mu3    ---------- ---------- ---------- ---------- ---------- -----MKPAG G----SPTES PGSKCADVSL GSPLPWAYPA DVRLCGHLRK QKSQRRRFFA LRTDP----- ---------- ---PRLECYE SEKQFRGGQA ---------- ---------R PQRSVSLVGA CTISKRADAR QRHLIVLYTR
Ovi_arm3   ---------- ---------- ---------- ---------- ---------- -----MKP-- G----DPTKT PEAESAAMAL GPPRPWVCPA DVRLCGHLRK QKSQRRRFFA LRADP----- ---------- ---PRLECYE SEKKFRAGRT ---------- ---------P PKFSVSLGGA CTISKRMDAR QRHLIVLYTR
Orc_or3    ---------- ---------- ---------- ---------- ---------- -----MKP-- G----GPTTT PEFESADVAL GPPRPWACPA DVRLCGHLRK QKSQRRRFFV LRAYP----- ---------- ---QRLECYE SEKKFRAGRA ---------- ---------P PKFSVSLEGA CTISKRVDAR QRHLIVLYTR
Xen_tr3    ---------- ---------- ---------- ---------- ---------- ---------- ---------- ---------- --------ME DIKKCGYLRK QKSMRKRYFV LRCPGTRGP- ---------- ---ARLEYYE NEKKFRVAEG SGG------- ---------- PRGVLNLEEA FGVNKRSDAK KRHLLVIYTR
                                                                       $                                          $$$$$$ $$$$$$$$$                             $$$$$ $$$                                         $   $$ $   $$$    $$
Mus_mu4    MASCSFSGHQ ALRRLRASAA AAASAALAAV ATTPLLSSGT RTALIGTGSS CPGAMWLSTA T----GSRSD SESEEEDLPV GD-------- EVCKRGYLRK QKHGHRRYFV LKLETADAP- ---------- ---ARLEYYE NARKFRHSVR AAAAAAEAAA SGAAVPALIP PRRVIILYQC FSVSQRADAR YRHLIALFTQ
Rat_no4    ---------- ---------- ---------- ---------- ---------- ----MWLSPA T----GSRSD SESDEEDLPV GD-------- EVCKRGYLRK QKHGQRRYFV LKLETEDAP- ---------- ---ARLEYYQ NARKFRLSVR AAAAAAAAAA SGAAVPALIP PRRVIILYQC FSVSQRADAR YRHLIALFTQ
Mes_au4    ---------- ---------- ---------- ---------- ---------- ----MWLSTA T----GSRSD SESEEEDLPV GD-------- EVCKRGYLRK QKHGHRRYFV LKLETEDAP- ---------- ---ARLEYYE NARKFRHSVR AAAAAAAAAA SGAAVPALIP PRRVITLYQC FSVSQRADAR YRHLIALFTQ
Mic_oc4    ---------- ---------- ---------- ---------- ---------- ----MWLPTA T----GSRSD SESEEEDLPV GD-------- EVCKRGYLRK QKHGHRRYFV LKLETEDAP- ---------- ---ARLEYYE NARKFRHSVR AAAAAAAAAA SGAPIPSLIP PRRVFILHQC FSVSQRADAR YRYLIALFTQ
Per_ma4    MASCSFAGDR ALRRLRA-AA AAASAALAAV ATTPLLSSGT PTALIGTGSS CPGAMWLPTA T----GSRSD SESEEEDLPV GD-------- EVCKRGYLRK QKHGHRRYFV LKLETEDAP- ---------- ---ARLEYYE NARKFRHSVR AAAAAAEAAA SGAAVPALIP PRRVIILDQC FSVSQRADAR YRHLIALFTQ
Pro_co4    MASCSFARDQ ATRRLRG-AA AAAAAALTAV ATTPLLSSGT PTALIGTGSS CPGAMWLSTA T----GSRSD SESEEEDLPV GA-------- EVCKRGYLRK QKHGHRRYFV LKLETEDAP- ---------- ---ARLEYYE NARKFRHSVR AAAAAAAAAA SGSAVPALIP PRRVITLYQC FSVSQRADAR YRYLIALFTQ
Mic_mu4    MASCSFARDQ ATRRLRG-AA AAAAALTAA ATTPLLSS-T PTALIGTGSS CPGAMWLSTA T----GSRSD SESEEED-PV GA-------- EVCKRGYLRK QKHGHRRYFV LKLETA-AP- ---------- ---ARLEYYE NARKFRHSVR AAAAAAAAAA SGAAVPALIP PRRVITLYQC FSVSQRADAR YRYLIALFTQ
Ovi_arm4   MASCSFARDQ ATRRLRA-AA AATAAALAAA ATTPFLSSGT PTAPIGTGSS CPGAMWLSTA T----GSRSD SESDEEDLPV GD-------- EVCKRGYLRK QKHGHRWRYFV LKLETEDAP- ---------- ---ARLEYYE NARKFRHSVR TAAAAAAAAA SGATVPALIA PRRVITLYQC FSVSQRADAR YPHLIALFTQ
Orc_or4    MASCSFARDQ ATRRLRA-AA AATAAALAAV APTPLLSSGT PTALIGTGSS CPGAMWLSTA T----GSRSD SESDEEDLPV GD-------- EVCKRGYLRK QKHGHRRYFV LKLETADAP- ---------- ---ARLEYYE NARKFRHSVR AAAAAAAAAA SGAAVPALIP PRRVITLYQC FSVSQRADAR YRHLIALFTQ
Xen_tr4    ---------- ---------- MAGVLCPEED RSSRAAVRML PPQPCPDESG GYRRMSGPTL S----QVQEE PAVEPPQPPL AASLAED--- DVRKRGYLRK QKHGHKRYFV LRSQSHLGP- ---------- ---ARLEYYD NARKFRSGQR SGC------- ---------H PKRVIPLYLC FTASRRADAK NKHLVALYTK
                                                                                                             $$$$ $$ $$$$  $$$                            $$$$$  $  $$$                                    $ $  $$$ $           $$
                                                                                                              ######### ########## ##                        ###### #####                              ##   # # ########## ##########
```

```
            PH Domain        Y   <<<<<                                                                     >>>>>                     PhosphoTyrosine Binding (PTD) Domain
Mus_mu1    DEHFAIAADS EAEQDSWYQA LLQLHNRAKA H-----HDGA ----GGGC-- -GGSCSGS-S GV--GEAG-- ----EDLSY- D-TGPGPAFK EVWQVILKPK GLGQTKNL-I GIYRLCLTSK TISFVK---- ------LNSE AAAVVLQLMN IRRCGHSENF FFIEVGRSAV TGPGEFWMQV DDSVVAQNMH ETILEAMRAM
Rat_no1    DEHFAIAADS EAEQDSWYQA LLQLHNRAKA H-----HDGA ----GGGC-- -GGSCSGS-S GV--GEAG-- ----EDLSY- D-TGPGPAFK EVWQVILKPK GLGQTKNL-I GIYRLCLTSK TISFVK---- ------LNSE AAAVVLQLMN IRRCGHSENF FFIEVGRSAV TGPGEFWMQV DDSVVAQNMH ETILEAMRAM
Mes_au1    DEHFAIAADN EAEQDSWYQA LLQLHNRAKA H-----HDGA ---GGGGC-- -GGSCSGS-S GV--GEAG-- ----EDLSY- D-TGPGPAFK EVWQVILKPK GLGQTKNL-I GIYRLCLTSK TISFVK---- ------LNSE AAAVVLQLMN IRRCGHSENF FFIEVGRSAV TGPGEFWMQV DDSVVAQNMH ETILEAMRAM
Mic_oc1    DEHFAIAADS EAEQDSWYQA LLQLHNRAKA H-----HDGA ---GGGGC-- -GGSCSGS-S GV--GEAG-- ----EDLSY- D-TGPGPAFK EVWQVILKPK GLGQTKNL-I GIYRLCLTSK TISFVK---- ------LNSE AAAVVLQLMN IRRCGHSENF FFIEVGRSAV TGPGEFWMQV DDSVVAQNMH ETILEAMRAM
Per_ma1    DEHFAIAADS EAEQDSWYQA LLQLHNRAKA H-----HDGA -----GGC-- -GGSCSGS-S GV--GEAG-- ----EDLSY- D-TGPGPAFK EVWQVILKPK GLGQTKNL-I GIYRLCLTSK TISFVK---- ------LNSE AAAVVLQLMN IRRCGHSENF FFIEVGRSAV TGPGEFWMQV DDSVVAQNMH ETILEAMRAM
Pro_co1    DEHFAIAADS EAEQDSWYQA LLQLHNRAKG H-----HDGA AAPGVGGG-- -GGSCSGS-S GL--GEAG-- ----EDLSYG D-APPGPAFK EVWQVILKPK GLGQTKNL-I GIYRLCLTSK TISFVK---- ------LNSE AAAVVLQLMN IRRCGHSENF FFIEVGRSAV TGPGEFWMQV DDSVVAQNMH ETILEAMRAM
Mic_mu1    DEHFAIAADS EAEQDSWYQA LLQLHNRAKG H-----HDGA AASGLGGG-- -GGSCSGS-S GL--GEAG-- ----EDLSYG D-APPGPAFK EVWQVILKPK GLGQTKNL-I GIYRLCLTSK TISFVK---- ------LNSE AAAVVLQLMN IRRCGHSENF FFIEVGRSAV TGPGEFWMQV DDSVVAQNMH ETILEAMRAM
Ovi_arm1   DEHFAIAADS EAEQDSWYQA LLQLHNRAKG H-----HDGA AAPGAGGG-- -GGSCSGS-S GL--GEAG-- ----EDLSYG D-VPPGPAFK EVWQVILKPK GLGQTKNL-I GIYRLCLTSK TISFVK---- ------LNSE AAAVVLQLMN IRRCGHSENF FFIEVGRSAV TGPGEFWMQV DDSVVAQNMH ETILEAMRAM
Orc_or1    DEHFAIAADS EAEQDSWYQA LLQLHNRAKG H-----HDGA AAPGAGGG-- -GGSCSGS-S GL--GEAG-- ----EDLSYG D-VPPGPAFK EVWQVILKPK GLGQTKNL-I GIYRLCLTSK TISFVK---- ------LNSE AAAVVLQLMN IRRCGHSENF FFIEVGRSAV TGPGEFWMQV DDSVVAQNMH ETILEAMRAM
Xen_tr1    EECFAIAAEC EQEQDGWYQA LVDLHNRGKT HHQHHNHDGA ---------- -------T-N GVHDGLNG-- ----DDV-YG EVTPPGLAFK EVWQVIMKPK GLGQLKNL-V GIYRLCLTNR TISLVK---- ------LNSD AAAVVLQLMN IRRCGHSENF FFIEVGRSAV TGAGEFWMQV DDSVVAQNMH ETILEAMKAL
                            $$                                                                                 $$                        $                                 $ $$  $$$ $  $$ $$              $$$$        $$$$       $
Mus_mu2    DEYFAVAAEN EQEQEGWYRA LTDLVSEGRS --------GE GGSGT--T-- -GGSCSASLP GVLGGSAG-- -AAGCDDNYG LVTPATAVYR EVWQVNLKPK GLGQSKNL-T GVYRLCLSAR TIGFVK---- ------LNCE QPSVTLQLMN IRRCGHSDSF FFIEVGRSAV TGPGELWMQA DDSVVAQNIH ETILEAMKAL
Rat_no2    DEYFAVAAEN EQEQEGWYRA LTDLVSEGRS --------GD GGSGT--T-- -GGSCSASLP GALGGSAG-- -AAGCDDNYG LVTPATAVYR EVWQVNLKPK GLGQSKNL-T GVYRLCLSAR TIGFVK---- ------LNCE QPSVTLQLMN IRRCGHSDSF FFIEVGRSAV TGPGELWMQA DDSVVAQNIH ETILEAMKAL
Mes_au2    DEYFAVAAEN EQEQEGWYRA LTDLVSEGRS --------GD GGSGIAAT-- -GGSCSASLP GVLGGSAG-- -AAGSDDNYG LVTPATAVYR EVWQVNLKPK GLGQSKNL-T GVYRLCLSAR TIGFVK---- ------LNCE QPSVTLQLMN IRRCGHSDSF FFIEVGRSAV TGPGELWMQA DDSVVAQNIH ETILEAMKAL
Mic_oc2    DEYFAVAAEN EQEQEGWYRA LTDLVSEGRS --------GD GGSGTAAT-- -GGSCSASLP GVLGGSAG-- -AAGSDDNYG LVTPATAVYR EVWQVNLKPK GLGQSKNL-T GVYRLCLSAR TIGFVK---- ------LNCE QPSVTLQLMN IRRCGHSDSF FFIEVGRSAV TGPGELWMQA DDSVVAQNIH ETILEAMKAL
Per_ma2    DEYFAVAAEN EQEQEGWYRA LTDLVSEGRS --------GD GGSGIAAT-- -GGSCSASLP GVLGGSAG-- -AAGSDD-YG LITPATAVYR EVWQVNLKPK GLGQSKNL-T GVYRLCLSAR TIGFVK---- ------LNCE QPSVTLQLMN IRRCGHSDSF FFIEVGRSAV TGPGELWMQA DDSVVAQNIH ETILEAMKAL
Pro_co2    DEYFAVAAEN EQEQEGWYRA LTDLVSEGRS ------GAGD APPNAAAT-- -SGSCSASLP GALGGSAG-- -AGAADDSYG LVSPATAAYR EVWQVNLKPK GLGQSKNL-T GVYRLCLSAR TIGFVK---- ------LNCE QPSVTLQLMN IRRCGHSDSF FFIEVGRSAV TGPGELWMQA DDSVVAQNIH ETILEAMKAL
Mic_mu2    DEYFAVAAEN EQEQEGWYRA LTDLVSEGRA ------GAGD APSNAAAT-- -SGSCSASLP GALGGSAG-- -AAGADDSYG LVSPATAAYR EVWQVNLKPK GLGQSKNL-T GVYRLCLSAR TIGFVK---- ------LNCE QPSVTLQLMN IRRCGHSDSF FFIEVGRSAV TGPGELWMQA DDSVVAQNIH ETILEAMKAL
Ovi_arm2   DEYFAVAAEN EQEQEGWYRA LTDLVSEGRA ------GAGD APPAAAAT-- -SGSCSASLP GALGGSAG-- -AAAADDSYG LVAPATAAYR EVWQVNLKPK GLGQSKNL-T GVYRLCLSAR TIGFVK---- ------LNCE QPSVTLQLMS IRRCGHSDSF FFIEVGRSAV TGPGELWMQA DDSVVAQNIH ETILEAMKAL
Orc_or2    DEYFAVAAEN EQEQEGWYRA LTDLVSEGRA ------SAGD APPAAAAAAA PSASCSASLP GALGGSAGAA VAAAADDSYG LVAPATAAYR EVWQVNLKPK GLGQSKNL-T GVYRLCLSAR TIGFVK---- ------LNCE QPSVTLQLMN IRRCGHSDSF FFIEVGRSAV TGPGELWMQA DDSVVAQNIH ETILEAMKAL
Xen_tr2    DEYFAVAADS EQEQESWYRA LTELLSTGRG ---------- AKEACAGT-- -GGCCCC--P GTASCSSSCT NHLALTEDYG LITPANAVKR EMWQVNLKPR GLGQIKNM-T GVYRLCLSAR SISFVR---- ------LNSE TPSVTLQLMN IRRCGHSDNY FFVEVGRSAS TGPGEIWMQV DDTVVAQSIH ETILEAMKAM
           $ $$        $ $  $$                                                                                  $$ $        $         $  $                            $    $ $   $$$  $                     $$$        $          $
Mus_mu3    DSSLGVAAAS EAEQQAWYSA LLEVRATAAA ---------- ------AAAT AMGFSPQEAP E--------- ---------- --SWIFAPFQ DVWPVTLRSK GLGRAQGLSS GSYRLCLGSG ALSLLRKPGS KGRSDSRATP PPVLRLSLLS VRRCGHADSF FFLELGRSAP IGPGELWLQA PDAVVAQSIH ETVLAAMKRL
Rat_no3    DSSLGVAAAS EAEQQTWYSA LLEVRATAAA ---------- ------AAAT AMGFSPQEAP E--------- ---------- --SWIFAPFQ DVWPVTLRSK GLGRAPGLSS GSYRLCLGSG ALSLLRKPGS KGRSDSRATP PPVLRLSLLS VRRCGHADSF FFLELGRSAP IGPGELWLQA PDAVVAQSIH ETVLAAMKRL
Mes_au3    DSSLGVAAAS EAEQQAWYSA LLEVRASAAA ---------- ------AAAT AMGLSPQEAP E--------- ---------- --SWIFAPFQ DVWPVTLRSK GLGRVRGLSS GSYRLCLGSG ALSLLRKPGS KGKSDSRASP PPALRLSLLS VRRCGHADSF FFLELGRSAP IGPGELWMQA PDAVVAQSIH ETVLAAMKRL
Mic_oc3    DSSLGVAAAS EAEQQAWYSA LLEVRASAAA ---------- ------AAAT AMGLSPQEAP E--------- ---------- --SWIFAPFQ DVWPVTLRSK GLGRAPGLSS GTYRLCLGSG ALSLLRKPGS KGRSDSRASP PPALRLSLLS VRRCGHADSF FFLELGRSAP TGPGELWMQA PDAVVAQNIH ETVLAAMKRL
Per_ma3    DSSLGVAAAS EVEQQVWYSA LLEVRASAAA ---------- ------AAAT AMGLSPREAP E--------- ---------- --SWIFAPFQ DVWPVTLRSK GLGRARGLSS GSYRLCLGSG SLSLLRKPGS KGRSDSRASP PPALRLSLLS VRRCGHADSF FFLELGRSAP TGPGELWMQA PDAVVAQSIH ETVLAAMKRL
Pro_co3    DSSLGVAAAS ETEQQAWYSA LLEVRAA-AG ---------- ------EARA -PGPSSHEDP G--------- ---------- --TWILSPFQ DIWPVTLRPK GLGRTRGLGS GGYRLCLGSG ALSLLRKPGG RGSRHTRALP PPALRLPLLS VRRCGHADSF FFLELGRSAP TGPGEVWLQA PDAVVAQNIH ETVLAAMKRL
Mic_mu3    DSSLGVAAAS EAEQQAWYCA LLEVRAA-A- ---------- ---------- --GPSSYEDP G--------- ---------- --TWILSPFQ DVWPVTLRPK GLGRTRGLGS GGYRLCLGSG ALSLLRKPRG RGFRDNRALP PPALRLSLLS VRRCGHADSF FFLELGRSAP TGPGELWLQA PDAVVAQNIH ETVLAAMKRL
Ovi_arm3   DRSLGVAAAS EAEQQAWYSA LLEARAA--- ---------- ---------- --AGPSSHEDP G--------- ---------- --AWILAPFQ DVWPVTLRPK GLGRARGLGS GGYSLCLGSG VLSLLRKPGG RGSGATGASP PPALRLSLLS VRRCGHADAF FFLELGRSAS IGPGELWLQA PDAVVAQSIH ETVLAAMKRI
Orc_or3    DRSLGVAAAS EAEQQAWYSA LLQARAAAAG ---------- ------EARA PAGGGAGEDP G--------- ---------- --AWILAPFQ DVWPVTLRPK GLGRARGLGS GDYCLCLGSG VLSLLRKPGG RSSRASRPSP PPALRLSLLS VRRCGHADAF FFLELGRSAP TGPGELWLQA PDAVVAQSIH ETVLAAMKRI
Xen_tr3    DGGLGVSADG EEEQDEWYQA LIEVQAQARA L--------- ---------- -SSSPDAP-- ---------- ---------- --AWPGPAFR EVWQVSVRPR GLGQTRNL-S GIYRLCLAER TLGLLR---- ------LRSE NPSVTLQLMN VRRCGHSDNY FFVEVGRSAV TGPGELWMQV EDSVVAQNIH ETILEAMKSL
           $          $  $$ $$ $                                                                       $    $$ $ $$$ $       $                       $                           $$$$$$ $ $ $$ $ $$      $$$ $ $   $ $$$$$$$       $$
Mus_mu4    DEYFAMVAEN ESEQESWYLL LSRLILESKR ---------- ------RRCG TLGALPDGEP AALAAAA--- ---------- --AAEPPFYK DWQQVVVKPR GLGHRKEL-S GVFRLCLTDE EVVFVR---- ------LNTE VASVVVQLLS IRRCGHSEQY FFLEVGRSTV IGPGELWMQV DDSVVAQNMH ELFLEKMRAL
Rat_no4    DEYFAMVAEN ESEQESWYLL LSRLILESKR ---------- ------RRCG TLGAQPDGEP AALAAAA--- ---------- --AAEPPFYK DWQQVVVKPR GLGHRKEL-S GVFRLCLTDE EVVFVR---- ------LNTE VASVVVQLLS IRRCGHSEQY FFLEVGRSTV IGPGELWMQV DDSVVAQNMH ELFLEKMRAL
Mes_au4    DEYFAMVAEN ESEQESWYLL LSRLILESKR ---------- ------RRCG TLDAQPDGEP AALAAAA--- ---------- --AAEPPFYK DWQQVVVKPR GLGHRKEL-S GVFRLCLTDE EVVFVR---- ------LNTE VASVVVQLLS IRRCGHSEQY FFLEVGRSTV IGPGELWMQV DDCVVAQNMH ELFLEKMRAL
Mic_oc4    DEYFALVAEN QSEQESWYSL LSRLILESKR ---------- ------RRCG LPGAQPDGEP DALEAAA--- ---------- --AAGPCFYK DVWQVVIKPR GLGHRKEL-S GVFRLCLTDE EVLFVR---- ------LNTE VASLIVQLMS IRRCGHSDQY FFLEVGRSTT IGPGELWMQV DDCVVAQNMH ELFLKKMRAL
Per_ma4    DEYFAMVAEN ESEQESWYLL LSRLILESKR ---------- ------RRCG TLGAQPDGEP GALAAAA--- ---------- --AAEPRFYK DWQQVVVKPR GLGHRKEL-S GVFRLCLTDE EVVFVR---- ------LNTE VASVVVQLLS IRRCGHSEQY FFLEVGRSTV IGPGELWMQV DDSVVAQNMH ELFLEKMRAL
Pro_co4    DEYFAMVAEN ESEQESWYLL LSRLILESKR ---------- ------RRCG TLGAQPDGEP AALAAAA--- ---------- --AAEPPFYK DVWQVIVKPR GLGHRKEL-S GVFRLCLTDE EVVFVR---- ------LNTE VASVVVQLLS IRRCGHSEQY FFLEVGRSTV IGPGELWMQV DDCVVAQNMH ELFLEKMRAL
Mic_mu4    DEYFAMVAEN ESKQESWYLL LSRLILESKR ---------- ------RRCG TLGAHPEGKP AALAAPA--- ---------- --AAEPPFYK KG?QVIVK-R GLGHRKEL-S GVFRLCLTDE EVVFVR---- ------LNTE VASVVVQLLS IRRCGHSEQY FFLEVGRSTV IGPGELWMQV DDCVVAQNMH ELFLEKMRAL
Ovi_arm4   NEYFAMVAEN ESEQESWYLL LSRLILESKR ---------- ------RRCG TLGAQPEGEP AALAAAA--- ---------- --AVEPPFYK DVWQVIVKPR GLGHRKEL-S GVFRLCLTDE EVLFVR---- ------LNTE VASVVVQLLS IRRCGHSEQY FFLEVGRSTV IGPGELWMQV DDCVVAQNMH ELFLEKMRAL
Orc_or4    DEYFAMVAEN ESEQESWYLL LSRLILESKR ---------- ------RRCG TPGAQPDGEP AALAAAA--- ---------- --AAEPPFYK DWQQVVVKPR GLGHRKEL-S GVFRLCLTDE EVVFVR---- ------LNTE VASVVVQLLS IRRCGHSEQY FFLEVGRSTV IGPGELWMQV DDCVVAQNMH ELFLEKMRAL
Xen_tr4    DEYFAMAAES EQEQDAWYQA LSELINESKG ---------- ---------- -------ACL DTEELEENYG T-LRPGTVFK EVWQVNVKPR GLGQAKNL-S GVYRLCLSSK AVHLVK---- ------LNSD VACVHLLLMN IRRCGHSENY FFIEVGRSSS TGAGELWMQV DDCVVAQHMH ETFLETMKAL
           $$$$ $        $  $$         $                                                                        $$    $    $ $   $   $$ $                       $              $$$$$$$$$$ $$ $ $$       $$$$$$  $$$$$$$$$$ $ $   $
           ##### ## # # ### ## # #                                                                         ## ########## ####       # #####                        #  #   ##### ########## ########## ########## ########## #########
```

```
                 PTB Domain                                   <<<<<
Mus_mu1    SD--EFRPRS KSQSS-SSCS NPISVPL--R RHHLNNPPPS QVGLTRRSRT E-SITATSPA SMVG--GKPG SFRVRASSDG EGTM---SR- -------PAS VDGSPVSPST NRTHAHRHRG SS-------- --RLHP---P LN---HSRSI PMPSSRCSP- --SATSP--- ---------- ---------- ----------
Rat_no1    SD--EFRPRS KSQSS-SSCS NPISVPL--R RHHLNNPPPS QVGLTRRSRT E-SITATSPA SMVG--GKPG SFRVRASSDG EGTM---SR- -------PAS VDGSPVSPST NRTHAHRHRG SS-------- --RLHP---P LN---HSRSI PMPSSRCSP- --SATSP--- ---------- ---------- ----------
Mes_au1    SD--EFRPRS KSQSS-SSCS NPISVPL--R RHHLNNPPPS QVGLTRRSRT E-SITATSPA SMVG--GKPG SFRVRASSDG EGTM---SR- -------PAS VDGSPVSPST NRTHAHRHRG SS-------- --RLHP---P LN---HSRSI PMPSSRCSP- --SATSP--- ---------- ---------- ----------
Mic_oc1    SD--EFRPRS KSQSS-SSCS NPISVPL--R RHHLNNPPPS QVGLTRRSRT E-SITATSPA SMVG--GKPG SFRVRASSDG EGTM---SR- -------PAS VDGSPVSPST NRTHAHRHRG SS-------- --RLHP---P LN---HSRSI PMPSSRCSP- --SATSP--- ---------- ---------- ----------
Per_ma1    SD--EFRPRS KSQSS-SGCS NPISVPL--R RHHLNNPPPS QVGLTRRSRT E-SITATSPA SMVG--GKPG SFRVRASSDG EGTM---SR- -------PAS VDGSPVSPST NRTHAHRHRG SS-------- --RLHP---P LN---HSRSI PMPSSRCSP- --SATSP--- ---------- ---------- ----------
Pro_co1    SD--EFRPRS KSQSS-SNCS NPISVPL--R RHHLNNPPPS QVGLTRRSRT E-SITATSPA SMVG--GKPG SFRVRASSDG EGTM---SR- -------PAS VDGSPVSPST NRTHAHRHRG SA-------- --RLHP---P LN---HSRSI PMPSSRCSP- --SATSP--- ---------- ---------- ----------
Mic_mu1    SD--EFRPRS KSQSS-SNCS NPISVPL--R RHHLNNPPPS QVGLTRRSRT E-SITATSPA SMVG--GKPG SFRVRASSDG EGTM---SR- -------PAS VDGSPVSPST NRTHAHRHRG SA-------- --RLHP---P LN---HSRSI PMPSSRCSP- --SATSP--- ---------- ---------- ----------
Ovi_arm1   SD--EFRPRS KSQSS-SNCS NPISVPL--R RHHLNNPPPS QVGLTRRSRT E-SITATSPA SLVG--GKQG SFRVRASSDG EGTM---SR- -------PAS VDGSPVSPST NRTHAHRHRG SS-------- --RLHP---P LN---HSRSI PMPSSRCSP- --SATSP--- ---------- ---------- ----------
Orc_or1    SD--EFRPRS KSQSS-SNCS NPISVPL--R RHHLNNPPPS QVGLTRRSRT E-SITATSPA SLVG--GKQG SFRVRASSDG EGTM---SR- -------PAS VDGSPVSPST NRTHAHRHRG SS-------- --RLHP---P LN---HSRSI PMPSSRCSP- --SATSP--- ---------- ---------- ----------
Xen_tr1    SD--EFRPRS KSQSS-SNCS NPISVPL--R RHHLNHPPPS QVGLNRRART E-SVTATSPA GGVAKHGSSS SFRVRASSDG EGTM---SR- -------PAS MEGSPVSPSA SRAQSHRHRG SS-------- --RLHP---P LN---HSRSI PMPATRCSP- --SATSP--- ---------- ---------- ----------
               $ $     $     $   $        $$ $  $$  $                                     $                              $                  $$   $  $   $     $$   $ $

Mus_mu2    KELFEFRPRS KSQSSGSSAT HPISVPGARR HHHLVNLPPS QTGLVRRSRT D-SLAATPPA AKC------T SCRVRTASEG DGGA---AGG AGTAGGRPMS VAGSPLSPGP VRAPLSRSHT LSAGCGGRPS KVTLAPAGGA LQ---HSRSM SMPVAHSPP- --AATSP--- ---------- ---------- ----------
Rat_no2    KELFEFRPRS KSQSSGSSAT HPISVPGARR HHHLVNLPPS QTGLVRRSRT D-SLAATPPA AKC------T SCRVRTASEG DGGA---AGG AGTAGGRPMS VAGSPLSPGP VRAPLSRSHT LSAGCGGRPS KVALAPAGGA LQ---HSRSM SMPVAHSPP- --AATSP--- ---------- ---------- ----------
Mes_au2    KELFEFRPRS KSQSSGSSAT HPISVPGARR HHHLVNLPPS QTGLVRRSRT D-SLAATPPA AKC------T SCRVRTASEG DGGA---AAG AGAAGGRPMS VAGSPLSPGP VRVPLSRSHT LSGGCGGRPS KVTLAPAGGA LQ---HSRSM SMPVAHSPP- --AATSP--- ---------- ---------- ----------
Mic_oc2    KELFEFRPRS KSQSSGSSAT HPISVPGARR HHHLVNLPPS QTGLVRRSRT D-SLAATPPA AKC------T SCRVRTASEG DGGA---AAG AGTAGGRPMS VAGSPLSPGP VRAPLSRSHT LSGGCGGRPS KVTLAPAGGA LQ---HSRSM SMPVAHSPP- --AATSP--- ---------- ---------- ----------
Per_ma2    KELFEFRPRS KSQSSGSSAT HPISVPGARR HHHLVNLPPS QTGLVRRSRT D-SLAATPPA AKC------T SCRVRTASEG DGGA---AAA AGTAGGRPMS VAGSPLSPGP VRAPLSRSHT LSAGCGGRPS KVTLAPAGGA LQ---HSRSM SMPVAHSPP- --AATSP--- ---------- ---------- ----------
Pro_co2    KELFEFRPRS KSQSSGSSAT HPISVPGARR HHHLVNLPPS QTGLVRRSRT D-SLAATPPA AKC------S SCRVRTASEG DGGA---AAG TGAGGGRPVS VAGSPLSPGP VRAPLSRSHT LSGGCGGRGS KVTLAPAGGA LQ---HSRSM SMPVAHSPP- --AATSP--- ---------- ---------- ----------
Mic_mu2    KELFEFRPRS KSQSSGSSAT HPISVPGARR HHHLVNLPPS QTGLVRRSRT D-SLAATPPA AKC------N SCRVRTASEG DGGA---AAG TGAAGGRPVS VAGSPLSPGP VRAPLSRSHT LSGGCGGRGS KVTLAPAGGA LQ---HSRSM SMPVAHSPP- --TATSP--- ---------- ---------- ----------
Ovi_arm2   KELFEFRPRS KSQSSGSSAT HPISVPGARR HHHLVNLPPS QTGLVRRSRT D-SLAATPPA AKC------S ACRVRTASEG DGGA---AAG AAAGAG---A AAGSPLSPGP VRAPLSRSHT LS---GGRAG KAALAPAGGG LQ---HSRSM SMPVAHSPP- --AATSP--- ---------- ---------- ----------
Orc_or2    KELFEFRPRS KSQSSGSSAT HPISVPGARR HHHLVNLPPS QTGLVRRSRT D-SLAATPPA AKC------S SCRVRTASEG DGGV---AAG AAAGGGRPVS VAGSPLSPGP VRAPLSRSHT LSGGCGGRAS KVTLAPAGGA LQ---HSRSM SMPVAHSPP- --AAATSP-- ---------- ---------- ----------
Xen_tr2    RELVEFRPRS KSQSSSS--- NPISVPG--R RHHLNQLPPS QTGLTRRTRT D-----FPST SVCV--SANT SSRLRTASEG DNLS---NR- ---------T VTGSPLSPSM LRAPLSRSHT LS--LCGRNT K--LVPIPGP LQ---HSRSM SMPVPRSPP- --TAASP--- ---------- ---------- ----------
               $$$$$ $ $                $$     $  $                                                                         $ $                     $   $$$$$ $$$

Mus_mu3    GS-------- ---NAA---- ---------- ---------- -------GKA EPPPQGNPPK ---------- ---------- ---------- ---------- ---------- ---------- ---------- ---------- ---------- -----SVPAA PTPTPYEIP- -ASAAQA--- ---------- ---------- ----------
Rat_no3    GS-------- ---SAAS--- ---------- ---------- -------GKA E-APLGDSPK ---------- ---------- ---------- ---------- ---------- ---------- ---------- ---------- ---------- -----GASAV PAPAPYEIPA LALAAQA--- ---------- ---------- ----------
Mes_au3    GS-------- ---GATS--- ---------- ---------- -------DEA E-PLSGDFPD ---------- ---------- ---------- ---------- ---------- ---------- ---------- ---------- ---------- -----SASAA PAPVPYETP- -ASVTQP--- ---------- ---------- ----------
Mic_oc3    GS-------- ---SAAS--- ---------- ---------- -------GKD E-PPSANPPK ---------- ---------- ---------- ---------- ---------- ---------- ---------- ---------- ---------- -----SASAA PPRVPYETP- -ASAAQP--- ---------- ---------- ----------
Per_ma3    GS-------- ---SGSS--- ---------- ---------- -------GKD E-PPSGEPPK ---------- ---------- ---------- ---------- ---------- ---------- ---------- ---------- ---------- -----SASAA PAPAPYETP- -TSAAQP--- ---------- ---------- ----------
Pro_co3    GS-------- ---GGGG--- ---------- ---------- -------GRA E-PLPRDSPT ---------- ---------- ---------- ---------- ---------- ---------- ---------- ---------- ---------- -----SASRL SVPQPYETS- -ASAAQS--- ---------- ---------- ----------
Mic_mu3    GS-------- ---GGVG--- ---------- ---------- -------ARA E-PLPRDCPT ---------- ---------- ---------- ---------- ---------- ---------- ---------- ---------- ---------- -----SASRL SVPQPYETS- -VSAAQS--- ---------- ---------- ----------
Ovi_arm3   GD-------- ---SGAV--- ---------- ---------- -------GRA E-PLPRKPPT ---------- ---------- ---------- ---------- ---------- ---------- ---------- ---------- ---------- -----GAFRP SVPQSYETP- -DSAAQT--- ---------- ---------- ----------
Orc_or3    GD-------- ---SGAG--- ---------- ---------- -------GRA E-PLPRKPPT ---------- ---------- ---------- ---------- ---------- ---------- ---------- ---------- ---------- -----SASTP SVPQSYETP- -ASAAQS--- ---------- ---------- ----------
Xen_tr3    SE--EFRPRT KSQSLSS--- TPISVPS--R RHHPNPPPPS QVGISRRSRA E-T------- ---------- ---------- ---------- ---------- ---------P IENSPV---- ---------- ---------- ---------P KP---HSLSK DY-------- --SVQSP--- ---------- ---------- ----------

Mus_mu4    CA-DEYRARC RSYSI-SIGA HLLTLLS--T RRHLGLLPLE PGGWLRRYGL E--------- ---------Q FCRLRAIRER -EEMLFTRRF ISPREPPPPF RRGRGHLPRA RR-------- ---------- ---------- -----SRRAA SVPPSLFRR- --SAPSPG-R IPQPEDVPND RAR---EASG SSGNTEE--
Rat_no4    CA-DEYRARC RSYSI-SIGA HLLTLLS--T RRHLGSLPLE PGGWFRRSRF E--------- ---------Q FCHLRAICHR -EDMLFTRRF IAPREPPPPF KRGRGHLPRA RR-------- ---------- ---------- -----SRRAV SVPPSFPRR- --SAPSPG-R IRQPEDARNH RAC---QASG SSGSNSEE--
Mes_au4    CA-DEYRARC RSYSI-TVGA HLLTLLS--T RRHLGLLPLE PGGWLRRSRF E--------- ---------Q FCRLRVIGDR EEEMLFTRRF R-PSEPLPPS RRGRGHLPRA RR-------- ---------- ---------- -----SRRAV SVPPSFSHR- --LAPSPPVR VSHTEEVSHN RAC---EASG SNSGNSEE--
Mic_oc4    CA-DEYRARC RSYSI-SIGG HLLTLLS--T RRHLGPHQLE PCGWFRRYRL E--------- ---------H LSHLRAISDR -EEMLFNRRF K-PSEPLPPS RLGRGHLPRA RR-------- ---------- ---------- -----SRRAV SVPPSFSHR- --LAPSPPVR VSHHEEV--- ---------- SNSSNSAE--
Per_ma4    CA-DEYRARC RSYSI-SVGA HLLTLLS--T RRHLGLLPLE PGGWLRRPRF E--------- ---------Q FCHLRAIGNR -EEMLFTRRL I-PNEPLLPS RRGRGHLPRA RR-------- ---------- ---------- -----SRRAV SVPPSFFRP- --VAPSPRVH VSHPDEVFND RAC---EASG SSSGNSEE--
Pro_co4    CA-DEYRARC RSYSI-CIGA HLLTLLS--T RRHLGLLPLE PGGWLRRSRF D--------- ---------Q FYHLRALGDR EDEMLFSRRF LTPSEPVPAS RRGRPHLPRV RW-------- ---------- ---------- -----SRRAI SVPACIFRR- --LSPSP-VR APNPAEAPND GAGQSSEA-- SGSGNSGEEG
Mic_mu4    CA-DEYRARC RSYSI-SIGA HLLTLLS--T RRHLGLLPIE PGGWFRRSRF E--------- ---------Q FYHFRALGDG EDEMLFSRHL VTREEPVIPS RRGRPHLPRM RR-------- ---------- ---------- -----SRRAI SIPGDLFHR- --LSSST-VR APITAEAPND GAGQSSEA-- SGSGNSGEEG
Ovi_arm4   CA-DEYRARC RSYSI-SIGG HLLTLLS--T RRHLDMLPLE PGGWLRRSRF E--------- ---------Q FCHLWAIGDG EDEMLFARRY ITPSEPVPRS RRGRLHLPRG RR-------- ---------- ---------- -----PRRAT SVPASFFRH- --TAPSP-VR ILYPAEALHD RDPVSSEASG SGSGNSGEEG
Orc_or4    CA-DEYRARC RSYSI-SIGA HLLTLLS--T RRHLDMLPLE PGGWLRRSRF E--------- ---------Q FCHLRAIGDG EDELLARRY ITPSEPVEPS RRGRLHLPRA RR-------- ---------- ---------- -----SRRAI SVPASFFRR- --VAPSP-VR GPHPAEAPSE GACLSSEAPG SGSGNSGEEG
Xen_tr4    KAYSEFRPRS KSQSSGT--- NPISFIT--T RRYLGNLPPS QTGLQRRSRT E-SVVGTPPS ------AKNN SFRFRTSSEG EGTM---TRP FR-------S VTGSLSHLNT ARINLGKQEG AG-------- ----RYVRAP FNSGYHSRSA SLPVSHFP-- --SATSP--- ---------- ---------- ----------
                $ $$   $               $ $$
                                                                                                                                                              #


Mus_mu1    ---------- ---------- -------VSL SSSSTSGHGS TSDCLFP--- ---------- --RRS---SA SVSGSPSDGG FISSDEYGSS PCDF-RS--S FRSVTPDSLG HTPPAR--GE EELSNYICMG GKGASTLAAP NGHYILSRGG NGHRYIPGAN LGTSPALPGD EAAGAADLDN RFRKRTHSAG TS-PT----I
Rat_no1    ---------- ---------- -------VSL SSSSTSGHGS TSDCLFP--- ---------- --RRS---SA SVSGSPSDGG FISSDEYGSS PCDF-RS--S FRSVTPDSLG HTPPAR--GE EELSNYICMG GKGASTLTAP NGHYILSRGG NGHRYIPGAT MGTSPALTGD EAAGAADLDN RFRKRTHSAG TS-PT----I
Mes_au1    ---------- ---------- -------VSL SSSSTSGHGS TSDCLFP--- ---------- --RRS---SA SVSGSPSDGG FISSDEYGSS PCDF-RS--S FRSVTPDSLG HTPPAR--GE EELSNYICMG GKGASTLTAP NGHYILSRGG NGHRYIPGAN LGTSPALTGD EATSAADLDN RFRKRTHSAG TS-PT----I
Mic_oc1    ---------- ---------- -------VSL SSSSTSGHGS TSDCLFP--- ---------- --RRS---SA SVSGSPSDGG FISSDEYGSS PCDF-RS--S FRSVTPDSLG HTPPAR--GE EELSNYICMG GKGASTLTAP NGHYILSRGG NGHRYLPGAN LGTSPALTGD EATSAADLDN RFRKRTHSAG TS-PT----I
Per_ma1    ---------- ---------- -------VSL SSSSTSGHGS TSDCLFP--- ---------- --RRS---SA SVSGSPSDGG FISSDEYGSS PCDF-RS--S FRSVTPDSLG HTPPAR--GE EELSNYICMG GKGASTLAAP NGHYVLSRGG NGHRYVPGAS LGTSPALTGD EAASAADLDN RFRKRTHSAG TS-PT----I
Pro_co1    ---------- ---------- -------VSL SSSSTSGHGS TSDCLFP--- ---------- --RRS---SA SVSGSPSDGG FISSDEYGSS PCDF-RS--S FRSVTPDSLG HTPPAR--GE EELSNYICMG GKGASTLTAP NGHYILSRGG NGHRYIPGAG LGTSPALAGD EAASAADLDN RFRKRTHSAG TS-PT----I
Mic_mu1    ---------- ---------- -------VSL SSSSTSGHGS TSDCLFP--- ---------- --RRS---SA SVSGSPSDGG FISSDEYGSS PCDF-RS--S FRSVTPDSLG HTPPAR--GE EELSNYICMG GKGASTLTAP NGHYILSRGG NGHRYIPGAG LGTSPALAGD EAASAADLDN RFRKRTHSAG TS-PT----I
Ovi_arm1   ---------- ---------- -------VSL SSSSTSGHGS TSDCLFP--- ---------- --RRS---SA SVSGSPSDGG FISSDEYGSS PCDF-RS--S FRSVTPDSLG HTPPAR--GE EELSNYICMG GKGASTLTAP SGHYILPRGG NGHRYVPAAG LGTSPALPGE EAAGAADLDN RFRKRTHSAG TS-PT----I
Orc_or1    ---------- ---------- -------VSL SSSSTSGHGS TSDCLFP--- ---------- --RRS---SA SVSGSPSDGG FISSDEYGSS PCDF-RS--S FRSVTPDSLG HTPPAR--GE EELSNYICMG GKGASTLTAP NGHYILPRSG NGPRYIPAAG LGTSPALTGD EAASAADLDN RFRKRTHSAG TS-PT----I
Xen_tr1    ---------- ---------- -------VSL SSSSTSGHGS TSDCMCP--- ---------- --RRS---SA SVSGSPSDGG FISSDEYGSS PCDF-RS--S FRSVTPDSLG HTPPAR---E EELNNYICMG KSGSHLQRSQ QQRYQLSRG- ---------- ---------- --EEHPDFDK VFRKRTHSSG TSPPT----V
                                            $     $                                           $     $$$$ $                $ $            $$ $                                                        $

Mus_mu2    ---------- ---------- -------GSL S--SSSGHGS GSYPLPPGSH PHLPHPLHHP QGQRPSSGSA SASGSPSDPG FMSLDEYGSS PGDL-RAFSS HRSNTPESIA ETPPARDGSG GELYGYMSMD RP---LSHC- GRPYRRVSG- ---------- ---------- --DGAQDLDR GLRKRTYSLT TP--------
Rat_no2    ---------- ---------- -------GSL S--SSSGHGS GSYPLPPGSH PHLPHPLHHP QCQRPSSGSA SASGSPSDPG FMSLDEYGSS PGDL-RAFSS HRSNTPESIA ETPPARDGSG GELYGYMSMD RP---LSHC- GRPYRRVSG- ---------- ---------- --DGAQDLDR GLRKRTYSLT TP--------
Mes_au2    ---------- ---------- -------GSL S--SSSGHGS GSYPLPPGPH PHLPHPLHHP QGQRPSSGSA SASGSPSDPG FMSLDEYGSS PGDL-RAFSS HRSNTPESIA ETPPARDGSG GELYGYMSMD RP---LSHC- GRPYRRVSG- ---------- ---------- --DGAQDLDR GLRKRTYSLT TP--------
Mic_oc2    ---------- ---------- -------GSL S--SSSGHGS GSYPLPPGPH PHLPHPLHHP QGQRPSSGSA SASGSPSDPG FMSLDEYGSS PGDL-RAFSS HRSNTPESIA ETPPARDGSG GELYGYMSMD RP---LSHC- GRPYRRVSG- ---------- ---------- --DGAQDLDR GLRKRTYSLT TP--------
Per_ma2    ---------- ---------- -------GSL S--SSSGHGS GSYPLPPGPH PHLPHPLHHP QGQRPSSGSA SASGSPSDPG FMSLDEYGSS PGDL-RAFSS HRSNTPESIA ETPPARDGSG GEFYGYMSMD RP---LSHC- GRPYRRVSG- ---------- ---------- --DGAQDLDR GLRKRTYSLT TP--------
Pro_co2    ---------- ---------- -------GSL S--SSSGHGS GSYPPPPGPH PHLPHPLHHP PGQRPSSGSA SASGSPSDPG FMSLDEYGSS PGDL-RAFCS HRSNTPESIA ETPPARDGSG GELYGYMTMD RP---LSHC- GRPYRRVSG- ---------- ---------- --EGVQDLDR GLRKRTYSLT TP--------
Mic_mu2    ---------- ---------- -------GSL S--SSSGHGS GSYPPPPGPH PHLPHPLHHP PGQRPSSGSA SASGSPSDPG FMSLDEYGSS PGDL-RAFCS HRSNTPESIA ETPPTRDGGG GELYGYMTMD RP---LSHC- GRPYRRVSG- ---------- ---------- --DGVQDLDR GLRKRTYSLT TP--------
Ovi_arm2   ---------- ---------- -------GSL S--SSSGHGS GSYPPPPGPH PHLQHPLHP- --QRPSSGSA SASGSPSDPG FMSLDEYGSS PGDL-RVYCG HRSNTPESIA ETPPARDGSA GELYGYMTME RP---LSHCG GRAYRRVSG- ---------- ---------- --DGAPDLDR GLRKRTYSLT TP--------
Orc_or2    ---------- ---------- -------GSL S--SSSGHGS GSYPPPPGPH PHMQHPLHP- --QRPSSGSA SASGSPSDPG FMSLDEYGSS PGDL-RAFCS LRSNTPESIA ETPPARDGSG GELYGYMTMD RP---LSHC- GRPYRRVSG- ---------- ---------- --DGAQDLDR GLRKRTYSLT TP--------
Xen_tr2    ---------- ---------- -------ISL SGSSSSGHGS TSDPLIP--- ---------- --PQSFSCSA SASGSPSDTG FMSLDEYSSS PGEIARVYLS CPSGTPDSAS ATPPHH---- GELNGYMSME RQFPGVCSC- ----RHLEG- ---------- ---------- --SVA---EK CARKRTYSLT TP--------
                                            $                                     $    $$    $                                         $$ $                                               $    $

Mus_mu3    ---------- ---------- -------RSP ------SERA KQDYFKP--- ---------- --LERMGSTH SY-KGLDLGG ---------- ---------- ---------- ---------- ----NYITMG ---------- ---------- ---------- ---------- ---------- ---------- ----------
Rat_no3    ---------- ---------- -------RSP ------GERA KQDYVNP--- ---------- --LERMGSAP SY-RGPDLGG ---------- ---------- ---------- ---------- ----DYIAMG ---------- ---------- ---------- ---------- ---------- ---------- ----------
Mes_au3    ---------- ---------- -------RSP ------GERS KQDH-KT--- ---------- --LERMGPTP SS-KGLDLGG ---------- ---------- ---------- ---------- ----DYITMG ---------- ---------- ---------- ---------- ---------- ---------- ----------
Mic_oc3    ---------- ---------- -------RSP ------GERA KQDYLKT--- ---------- --LGRMGPAP SY-KELDLGG ---------- ---------- ---------- ---------- ----DYITMG ---------- ---------- ---------- ---------- ---------- ---------- ----------
Per_ma3    ---------- ---------- -------RSP ------GERE KQDYLKA--- ---------- --LERLGPAP SY-KGLDLDG ---------- ---------- ---------- ---------- ----DYIAMG ---------- ---------- ---------- ---------- ---------- ---------- ----------
Pro_co3    ---------- ---------- -------SSL SRRGCLGERI KQTTLRT--- ---------- --PVRLGAAA LYSKRLDPGG ---------- ---------- ---------- ---------- ----GYINMR ---------- ---------- ---------- ---------- ---------- ---------- ----------
Mic_mu3    ---------- ---------- -------SSL SRRGCLGERI KQATLGT--- ---------- --PARLGAAA LHSKALDQGG ---------- ---------- ---------- ---------- ----SYINMG ---------- ---------- ---------- ---------- ---------- ---------- ----------
Ovi_arm3   ---------- ---------- -------SEL ---NRRNGRS E-----T--- ---------- --LARLGEAA SNTARLERGG ---------- ---------- ---------- ---------- ----SYITMG ---------- ---------- ---------- ---------- ---------- ---------- ----------
Orc_or3    ---------- ---------- -------SSL YRPGRLSERS EQATLKT--- ---------- --LARPGAAA SHPEGLELGG ---------- ---------- ---------- ---------- ----VYITKG ---------- ---------- ---------- ---------- ---------- ---------- ----------
Xen_tr3    ---------- ---------- -------EEE ---------- ---------- ---------- --EEEKGARV ETNESSADYG SASSDEYGSS PGVL-EA--- -----PVFLP PSPGPR---- --ETNYISMA LYG------- ---------- ---------- ---------- ---------- ---RRSLVME ---PI----S

Mus_mu4    --KDKEGEEG NRGDCIPMNN WGSGNGRGSG GGRGSSGQGS SS-------- ---------- --QG------ SGGRQGSGGG Q------GSG GQG-AG---- -GNQCSGNGQ GTAGGHGSG- ----G----G GHGSGGGQRP GDGHGSGGG- -------KN- -----SGSGK NSD-DGDRGK SVKKRSYFGK FTQSK----Q
Rat_no4    --KDKEGEEG NGGACMPMNN WGSGNGRGSG GGQGSSGQGS SS-------- ---------- --QS------ SGGRQGSGGG Q------GSG GQG-AG---- -GNQRSGNGQ GTAGGHGSG- ----G----G GHGSGGGQGP GDGHGSGGG- -------KNS GGSKGSGSGK NSD-DGDRGK SVKKRSYFGK FTQSKQQQQQ
Mes_au4    --KDKESEEA NEGDYMPMNN WGSGNGRGSG GGQGSSGQGS SS-------- ---------- --QSSGGNQC SGGRQAS-GG QGSGG-RGSG GQG-AG---- -GNQCSGSGQ G-AGGHGSGG GQRPG----G GHGSGGGRGP GDGHGSGGG- -------KNS GGGKGSGSGK NSD-DGDRGK SLKKRSYFAK FTQSK----Q
Mic_oc4    --KDKDAEEG NEGDYMPMNN WGSGNGRGSG GGQGSSGQGS SS-------- ---------- --QSSGGNQY SGGRQGSGGG Q------GSG GQGAAG---- -GNQYSGNGQ GTAGGHGSGG GQRG-----G GHGSSGGQGP GDGRGSSGG- -------KNP GGAKGSGSGK NSD-DGDHGK SLKKRSYFGK FTQNK----Q
Per_ma4    --KDKESEEG NGGDYMPMNN WGSGNGRGSG GGQGSSGQGT SS-------- ---------- --QSSGGNQC SGGRQGSGGG QGSGG-QGSG GQG-AG---- -GNQCSGN-- ----GHGSGG GQRPG----G GHGSSGGQGP GDGHGSGGG- -------KNS GGGKGSGSGK NSN-EGDRGK SLKKRSYFSK FTQSK----Q
Pro_co4    NPQGKEGQEG REGDYMPMNN WGSGNGRGSG GGQGSSGQGS SS-------- ---------- --QSSGGNQC SGGGQGSRGG QGSSGSQGSG GQG-SG---- -GNQCSGNGQ GTAG--GSGG GQGAG----G GHGSGGGRGP GDGHGSGSG- -------KNS GGGKSSGSGK GAEGDGERGK SLKKRSYFGK LTQSK----Q
Mic_mu4    NPQGREDQEG NGGDYMPMNN WGNGNGRGSG GGQGSSGQGS SS-------- ---------- --QSSGGNQC SGGGQGSRGG ------QGSS GQG-AG---- -GNQCSGNGQ GTAG--GSGG GQGAG----G GRGSGSGRGP GDGHGSGGG- -------KNS GGGKGSGSGK GAEGDGERGK SLKKRSYFGK LTQSK----Q
Ovi_arm4   GPQGKEDQKE NEGDYMPMNN WGSGNGRGSG SGQGSSGQGS SG-------- ---------- --QSSGGSQC SGRGHGSGGG QGSSSSHGSS GSG-SGDQSA GGNQCSGDGQ GTA-GHGSSS GQGAG----G GHGSGRGQGP GDGHGSGGG- -------KNS GGGKGSGSGK GPEGNGDRGK SLKKRSYFGK LTQGK----Q
Orc_or4    NPQGKEDQEG NGGDYMPMNN WGSGNGRGSG GGQGSSGQGS SS-------- ---------- --QSSGGSQC SGRGHGSGGG QGSSGSQGSR ---------- -GNQCSGDGQ GTSGGHGSGS GQGAG----G GHGSGRGQGP GDGHGSGRG- -------RNS GGGKGSGSGK GSEGNGDHGK SLKKRSYFGK LTQSK----Q
Xen_tr4    ---------- ---------- -------ISV C--SSSGHGS ASETL----- ---------- --TRPS--SS SVCGSPSDGG FISSDEYGSS PGDL-RYF-R VRSNTPDSLG NTPPIQ--EE NTLSDYMSMS THSHPDSK-- ---------- ---------- ---------- --DDYMEADK CFRKRTYSLT KPTKS----A
                        $$ $$                $
                                                                                 #
```

```
                                                                                                         pY                      pY
Mus_mu1     SHQKTP-SQS SVASI----E EYTEMMPAAY PPGGGSGGRL --PGYRHSAF VPTHSYPEEG LEMHHLERRG -GHHRPDTSN LHTDDGYMPM SPGVAPVPSN R--KGNGDYM PMSPKSV--- ------SAPQ QIINPIRRHP QRVDPNGYMM MSPSGSCS-- -PDIGGGSSS SS-SISAAPS GS--SYGKPW TNGVGGHHTH
Rat_no1     SHQKTP-SQS SVVSI----E EYTEMMPAAY PPGGGSGGRL --PGYRHSAF VPTHSYPEEG LEMHHLERRG -GHHRPDSSN LHTDDGYMPM SPGVAPVPSN R--KGNGDYM PMSPKSV--- ------SAPQ QIINPIRRHP QRVDPNGYMM MSPSGSCS-- -PDIGGGSCS SS-SISAAPS GS--SYGKPW TNGVGGHHTH
Mes_au1     SHQKTP-SQS SVASI----E EYTEMMPAAY PPGGGSGGRL --PSYRHSAF VPTHSYPEEG LEMHPLERRG -GHHRPDTSS LHTDDGYMPM SPGVAPVPSN R--KGNGDYM PMSPKSV--- ------SAPQ QIINPIRRHP QRVDPNGYMM MSPSGSCS-- -PDI-GGGSS SS-S-GAAAS GS--SYGKPW TNGVGGHHSH
Mic_oc1     SHQKTP-SQS SVASI----E EYTEMMPAAY PPGGGSGGRL --PSYRHSAF VPTHSYPEEG LEMHPLERRG -GHHRPDTST LHTDDGYMPM SPGVAPVPSN R--KGNGDYM PMSPKSV--- ------SAPQ QIINPIRRHP QRVDPNGYMM MSPSGSCS-- -PDIGGGGSS SS-S-SAAPS GS--SYGKPW TNGVGGHHPH
Per_ma1     SHQKTP-SQS SVASI----E EYTEMMPAAY QPGGGSGGRL --PSYRHSAF VPTHSYPEES LEMHPLERRG -GHHRPD--- --TDDGYMPM SPGVAPVPSN R--KGNGDYM PMSPKSV--- ------SAPQ QIINPIRRHP QRVDPNGYMM MSPSGSCS-- -PDIGGGSSS SS-S-GAAPS GS--SYGKPW ANGVGGHHPH
Pro_co1     SHQKTP-SQS SVASI----E EYTEMMP-AY PPGGGSGGRL --PGHRHSAF VPTHSYPEEG LEMHPLEPQG -GHHRPDTST LHTDDGYMPM SPGVAPVPGS R--KGSGDYM PMSPKSV--- ------SAPQ QIINPIRRHP QRVDPNGYMM MSPSGSCS-- -PDLGGGPSS ---SSSAAPS GS--SYGKLW TNGVGSHHSP
Mic_mu1     SHQKTP-SQS SVASI----E EYTEMMP-AY PPGGGSGGRL --PGHRHSAF VPTHSYPEEG LEMHPMEPQG -GHHRPDTST LHTDDGYMPM SPGVAPVPSS R--KSSGDYM PMSPKSV--- ------SAPQ QIINPIRRHP QRVDPNGYMM MSPSGSCS-- -PDLGGGPSS ---SSSAAPS GS--SYGKLW TNGVGGHHSP
Ovi_arm1    SHQKTP-SQS SVASI----E EYTEMMP-AY PPGGGSGGRV --PSYRHSAF VPTHSYPEEG LEMHPLERRV -GHHRPDTSS LHTDDGYMPM SPGVAPVPGS R--KGSGDYM PMSPKSV--- ------SAPQ QIINPIRRHP QRVDPNGYMM MSPSGSCS-- -PDIGGGPSS GGSSSGAAPS GS--SYGKLW TNGVGGHHSH
Orc_or1     SHQKTP-SQS SVASI----E EYTEMMP-AY PPGGGSGGRV --PSYRHSAF VPTHSYPEEG LEMHPLERRV -GHHRPDTSN LHTDDGYMPM SPGVAPVPGS R--KGSGDYM PMSPKSV--- ------SAPQ QIINPIRRHP QRVDPNGYMM MSPSGSCS-- -PDIGGGPSS GG-SSGAAPS GS--SYGKLW TNGVGGHHSH
Xen_tr1     SHQKTP-SQS ---SI----E EYTEMMP-AH PV------RL --TSFRHSAF VPTYSYPEEC LDLHLEGSRA -NH------- --TDDGYMPM SPGVAPVPTK -----SNDYM PMSPKSV--- ------SAPQ QIINP-RRHS A-VDSNGYMM MSPSGSCS-- -PD------- ---S------ -T--NYSKIW TNGT------
             $$ $                  $$$                        $  $    $ $                                 $$$$                    $$ $$ $                $ $       $       $ $$$ $ $   $                                   $
Mus_mu2     ARQRQV-PQP SSASL----D EYTLMRA--- -TFSGSSGRL CPSFPASSPK VAYNPYPEDY GDIEI----- -GSHKSSSSN LGADDGYMPM TPGAALRSGG PNSCKSDDYM PMSPTSV--- ------SAPK QILQPRL--A AALPPSGAAV PAPPSGVGRT FPVNGG-GYK ASSPAESSPE DS--GYMRMW C-G-------
Rat_no2     ARQRQV-PQP SSASL----D EYTLMRA--- -TFSGSSGRL CPSLPASSPK VAYNPYPEDY GDIEI----- -GSHKSSSSN LGADDGYMPM TPGAALRSGG PNSCKSDDYM PMSPTSV--- ------SAPK QILQPRS--A AALPPSGAAV PAPPSGAGRT FPVNGG-GYK ASSPAESSPE DS--GYMRMW C-G-------
Mes_au2     ARQRPV-PQP SSASL----D EYTLMRA--- -TFSGSSGRL CPPFPASSPK LAYHPYPEDY GDIEI----- -GSHKSSSSN LGADDGYMPM TPGAVLRSGG PGSCKSDDYM PMSPTSV--- ------SAPK QIMQPRSVAA TALPPSGGAV PAPASGPGRT FPVNGG-SYK ASSPAESSPE DS--GYMRMW C-G-------
Mic_oc2     ARQRPV-PQP SSASL----D EYTLMRA--- -TFSGSSGRL CPSFPVSSPK VAYHPYPEDY GDIEI----- -GSHKSSSSN LGADDGYMPM TPGAALRSVG PSSCKSDDYM PMSPTSV--- ------SAPK QILQPRSAAA AALPPSGAAV PAPPSGAGRT FPLNGG-SYK ASSPAESSPE DS--GYMRMW C-G-------
Per_ma2     ARQRPV-PQP SSASL----D EYTLMRA--- -TFSGSSGRL CPSFPASSPK MAYHPYPEDY GDIEI----- -GSHKSSSSN LGADDGYMPM TPGAALRSGG PSSRKSDDYM PMSPTSV--- ------SAPK QILQPRS-AA AALPPSGAAG PAPPSGPGRT FPVNGGNNYK ANSPAESSPE DS--GYMRMW C-G-------
Pro_co2     ARQRPV-PQP SSASL----D EYTLMRA--- -TFSGSSGRL CPSCPASSPK VTYHPYPEDY GDIEI----- -GSHRSSSSN LGADDGYMPM TPGVALMGSG GGSCKSDDYM PMSPTSV--- ------SAPK QILQPRA-AT AALPPTGAAM PAPTSAASRT FAGNGG-SYK TSSPAESSPE DS--GYMRMW C-G-------
Mic_mu2     ARQRPV-PQP SSASL----D EYTLMRA--- -TFSGSSGRL CPSCPASSPK VTYHPYPEDY GDIEI----- -GSHRSSSSN LGADDGYMPM TPGVALMGSG GGSCKSDDYM PMSPTSV--- ------SAPK QILQPRA-AT AALPPAGAAA PAPAAAASRT FAGTGG-GYK TSSPAESSPE DS--GYMRMW C-G-------
Ovi_arm2    ARQRPA-PQP SSASL----D EYTLMRA--- -TFSGSSGRL CPSCPASSPK VAYHPYPEDY GDIEI----- -GSHRSSSSN LGTDDGYVPM TPGVAPLRTG GGSCRGDDYM PMSPTSV--- ------SAPK QILQPRP-AP AALPPAGAAG PTPASAAGRA FPGTAG-GYK TGSPAESSPE DS--GYMRMW C-G-------
Orc_or2     ARQRPV-PQP SSASL----D EYTLMRA--- -TFSGSSGRL CPSCPASSPK VAYHPYPEDY GDIEI----- -GSHRSSSSN LGTDDGYMPM TPGVA-LAGG GGSCKSDDYM PMSPTSV--- ------SAPK QILQPRA-AV AALPPTGAAG PTPASAASRA FPGSGG-GYK TSSPAESSPE DS--GYMRMW CGG-------
Xen_tr2     GRQRMAPPHV SSASL----D EATLVRA--- -TYKGSSGLL -----SSSPK VSYQPYPEDY ADVEI----- -GRQ------ ---DDGYMPM SHGVG----- ---VSSSDYV PMSPASV--- ------SAPQ QILQPRC--- ---------- ------CSET F--------- -----EGSQE DSPDGYMRMW C-G-------
                                                                       $$$  $$                            $  $ $                  $$ $$ $               $  $                                                              $ $
Mus_mu3     ---------- ---------- ---------- ---------- ---------- ---------- ---------- ---------- --VRNDYVHM GGGEA----- ------GDYM WMAPPGL-PP TPAR--ADPN KQLEDCE--- ---------- ---------- ---------- ---------- ---------- ----------
Rat_no3     ---------- ---------- ---------- ---------- ---------- ---------- ---------- ---------- --MRNDYVHM -GGKA----- ------AEYM WMAPPGLPPP TPPR--VDPR KEPEDCE--- ---------- ---------- ---------- ---------- ---------- ----------
Mes_au3     ---------- ---------- ---------- ---------- ---------- ---------- ---------- ---------- --ARDDYMRM -GGEA----- ------GDYM WMAPPSL-PP TPAR--VDPG RSLQDCE--- ---------- ---------- ---------- ---------- ---------- ----------
Mic_oc3     ---------- ---------- ---------- ---------- ---------- ---------- ---------- ---------- --VRDDYVHM AGGEV----- ------GDYM LMAPPGL-PP IPAK--IAPG KSLQDCE--- ---------- ---------- ---------- ---------- ---------- ----------
Per_ma3     ---------- ---------- ---------- ---------- ---------- ---------- ---------- ---------- --VRDDYVHM GGGEA----- ------GDYM WMAPPGL-PP TPAR--VALG KSLQDSE--- ---------- ---------- ---------- ---------- ---------- ----------
Pro_co3     ---------- ---------- ---------- ---------- ---------- ---------- ---------- ---------- --AGSDYEPM GGGEA----- ------GGYT VMASPGL-CP ATASAAPAPS QPLQDWG--- ---------- ---------- ---------- ---------- ---------- ----------
Mic_mu3     ---------- ---------- ---------- ---------- ---------- ---------- ---------- ---------- --AGSDYEPM GGGAA----- ------GGYT VMASPGL-CP AT----PAPG QPLQDWG--- ---------- ---------- ---------- ---------- ---------- ----------
Ovi_arm3    ---------- ---------- ---------- ---------- ---------- ---------- ---------- ---------- --AGSDYEPM KGGQA----- ------GGYV VMEPPGL--P ASTK--AAPR QPLQDGG--- ---------- ---------- ---------- ---------- ---------- ----------
Orc_or3     ---------- ---------- ---------- ---------- ---------- ---------- ---------- ---------- --ARSDYEPM GGGQA----- ------SGYV VMATPGL--P ASAK--AASH QLLQDGG--- ---------- ---------- ---------- ---------- ---------- ----------
Xen_tr3     TNANVPAEEG SRLTLFQEED NYAMM----- ---------- ---------- ---------- ---------- -GQREPR--- --QETGYMPM LPGS------ ---NRSQDYM PMTPTSI--- ------SPP- ---------- APVEMAGYVM MSPLGSCS-- -PEIERLSWP PSQEVSAGSS DS-------- ----------
                                                                                                          $  $                        $
Mus_mu4     QQTLPPPPPP PPA------- ------AGAT GGKGKSGGRF RLYFCADRG- TKERKEAKEV RDMETSGGAT RGPYRARAFD EDEDDPYVPM RPGVA----- APLACSSDYM PMAPQNS--- ------SAST K----RHSRS PFEDSRGYMM MFPRVSPPPP VPSAPK-APD TNKGDDSKDN DS-------- ----------
Rat_no4     QQTLPPPPPP PPA------- ------AGAT GGKGKSGGRF RLYFCADRG- TKERKEAKEV RNMETSQGTT RGSLRARALY EDEDDPYVPM RPGVA----- APLACSSDYM PMAPQNS--- ------SAPT K----RHSKS PFEDSRGYMM MFPRVSPPPP APSARK-APD THKEDDSKDN DS-------- ----------
Mes_au4     QQMLPPPPPP PPP------- ------AGAT GGKSKSGGRF RLYFCADRGA TKERKEAKEV RDRETPEGAT RGPHRARAFD EDEDDPYVPM RPGVA----- APLACSSDYM PMAPQYS--- ------FASK K----RHSRS PFEDSRGYMM MFPRVSPPPP VPSPPK-APD TNKEDDSKDN DS-------- ----------
Mic_oc4     QQMLPPPPPP PPPPS----- ------TGAT GGKGKSGGRF RLYFCADKGA TKERKEVKEV RDMETLEGAT RAPYRARAFD EDEDDPYVPM RPGVA----- APLACSSDYM LMAPQNS--- ------SASK K----GHSKS PFEDSRGYMM MFPRVSPLPP APSPPK-APD TNEKDDSKDN DS-------- ----------
Per_ma4     QQMLPPPPPP PPT------- ------AGAA GGKGKSGGRF RLYFCADRGA TKERKEAKEM REMESSEGAT RGPHRARAFD EDEDDPYVPM RPGVA----- APLACSSDYM PMAPQNS--- ------SASK K----RHSRS PFEDSRGYMM MFPRVSPPPP APSPPK-APD TNKEDDSKDN DS-------- ----------
Pro_co4     QQMSGPPPPP PPPPP----- ------AGAT GGKAKSGGRF RLYFCADKGA TKERKEAKEV KHAEIPEGAA RGPYRARAFD EDEDDPYVPM RPGVA----- APLASSSDYM PMAPQNV--- ------SAS- K----RHSRS PFEDSRGYMI MFPRVNP-PP VPSPPK-TPD PNKEDDSKDN DS-------- ----------
Mic_mu4     QQMSGPPPPP PPPPP----- ------AGAT GGKGKSVGRF RLYFCADRGA TKERKEAKEV KPAEIPEGAA RGPYRARAFD EDEDDPYVPM RPGVA----- APLASSSDYM PMAPQNA--- ------SAS- K----RHSRS PFEDSRGYMM MFPRVSP-PP VPSPPK-TPD PNKEDDSKDN DS-------- ----------
Ovi_arm4    QQM--PPPPP PPPPP----- ------GAAT GGKGKSGGRF RLYFCADRGA TKERKEAKEV KDTEPPEGAA RGPHRARAFD EDEDDPYVPM RPGVA----- APLASSSDYM PMAPQNV--- ------SVSK K----RHSRS PFEDSRGYMM MFPRVSP-PP VPSPPK-APD PAKEDDSKDN DS-------- ----------
Orc_or4     QQM--PPPSP PPPPP----- ------AAAT GGKGKSGGRF RLYFCADRGA TKERKEAKEV KDAETPEGAA RGPHRARAFD EDEDDPYVPM RPGVA----- APLSSSSDYM PMAPQNV--- ------SVSK K----RHSRS PFEDSRGYMM MFPRVSP-PP APSPAK-APD PDKEDDSKDN DS-------- ----------
Xen_tr4     SQQKSQ---- AAISLDEDSE EINKQFA--- ---------- ----YAESPK LKDSSHVEDY SN-GIIDSVC -NHSRSKA-- --SDDGYMPM MPSN------ ---SYDSDYL PMAPKTV--- ------SAPK QI----NRCP SQGDSKGYMM MFPINSS--- -PVKNVFGGA ATKSNIEKLS NG--GYMDMS YGN-------
                                                                                                       $$ $ $$ $$                $$$ $$ $                                   $$$$ $$$
                                                                                                         #####  ##               ### ####


Mus_mu1     ALPHAKPPVE S-GGGKLLPC TGDYMNMSPV GDS-NTSSPS --ECY----- ---------- ----YGPEDP QHKPVLSYYS LPRSFKHTQR PGEPEEGAR- HQHLRLSSS- ---------- -----SGRLR YTATAEDSSS STSSDSLGGG YCGARPESSL THP-HHHVLQ PHLP-----R KVDTAAQTNS RLARPTRLSL
Rat_no1     ALPHAKPPVE S-GGGKLLPC TGDYMNMSPV GDS-NTSSPS --ECY----- ---------- ----YGPEDP QHKPVLSYYS LPRSFKHTQR PGEPEEGAR- HQHLRLSSS- ---------- -----SGRLR YTATAEDSSS STSSDSLGGG YCGARPESSV THP-HHHALQ PHLP-----R KVDTAAQTNS RLARPTRLSL
Mes_au1     ALPHSKPPIE S-GGGKLLPC TGDYMNMSPV GDS-NTSSPS --ECY----- ---------- ----YGPEDP QHKPVLSYYS LPRSFKHTQR PGEPEEGAR- HQHLRLSSS- ---------- -----SGRLL YTATAEDSSS STSSDSLGGG YCGARPEAGL PHP-HHHALQ PHGP-----R KVDTAAQTNR RLARPTRLSL
Mic_oc1     ALPHSKAPAE S-GGGKLLPC TGDYMNMSPV GDS-NTSSPS --ECY----- ---------- ----YGPDNP QHKPVLSYYS LPRSFKHTQR PGEPEEGAR- HQHLRLSSS- ---------- -----SGRLL YTATAEDSSS STSSDSLGGS YCGARPESGL THP-HHHVLQ PHLP-----R KVDTAAQTNN RLARPTRLSL
Per_ma1     ALPHSKPPAE S-GGGKLLPC TSDYMNMSPV GDS-NTSSPS --ECY----- ---------- ----YGPEDP QHKPVLSYYS LPRSFKHTQR PGEPEEGARH HQHLRLSSS- ---------- -----SGRLL YTTTAEDSSS STSSDSLGGG YCGARPESGL PHP-HHHVLQ PHLP-----R KVDTAAQTNS RLARPTRLSL
Pro_co1     ALPHPKPPVE S-SGGKLLPC TGDYMNMSPV GDS-NTSSPS --GCY----- ---------- ----YGPEYP QQKPVLSYYS LPRSFKHTQR PGEPEEGAR- HHHLRLSSS- ---------- -----SGRLL YAATAEDSSS STSSDSLGGG YCGARLEPGL PHA-HHQVLQ PHLP-----R KVDTAAQTNS RLARPTRLSL
Mic_mu1     ALPHPKPPVE S-SGGKLLPC TGDYMNMSPV GDS-NTSSPS --GCY----- ---------- ----YGPEYP QHKPVLSYYS LPRSFKHTQR PGEPEEGAR- HQHLRLSSS- ---------- -----SGRLL YAAAAEDSSS STSSDSLGGG YCGARLEPGL PHAHHHQVLQ PHLP-----R KVDTAAQTNS RLARPTRLSL
Ovi_arm1    ALPHPKLPVE S-GSGKLLSC TGDYMNMSPV GDS-NTSSPS --DCY----- ---------- ----YGPEDP QHKPVLSYYS LPRSFKHTQR PGELEEPRH- HQHLRLSSS- ---------- -----SGRLL YAAAAEDSSS STSSDSLGGG YCGARPEPGL PHL-HHQVLQ AHLP-----R KVDTAAQTNN RLARPTRLSL
Orc_or1     ALPHPKLPVE S-SSGKLLSC TGDYMNMSPV GDS-NTSSPS --DCY----- ---------- ----CGPEDP QHKTVLSYYS LPRSFKHTQR PGELEEGAR- HQHLRLSSS- ---------- -----SGRLL YAAAAEDSSS STSSDSLGGG YCAARPEPGL PHL-HHQVLQ PHLP-----R KVDTAAQTNS RLARPTRLSL
Xen_tr1     ---NPKLSID S-IEGK-LPC S-DYINMSPA SGS-TTSTPP --DSY----- ---------- ----LNSIEE STKPVYSYFS LPRSFKHVHR KSE------- DGNLRITAN- ---------- -----SGHNL Y--TEDSSSS STSSDSLGGQ ---------- ---------D PQQP-----R KGETCIQ-GK RLTRPTRLSL
                            $                                                               $ $
Mus_mu2     ----SKLSME N-PDPKLLP- NGDYLNMSPS EAG-TAGTPP --D-F----- -------SAA LRGG--SEGL KGIPGHCYSS LPRSYKAPCS C-SGDN---- DQYVLMSSP- ---------- -----VGRIL EEERLEPQAT PG----AGTF GA---AGGSH TQPHHSAVPS SMRPSAIG-G RPEGFLGQRC RAVRPTRLSL
Rat_no2     ----SKLSME N-PDPKLLP- NGDYLNMSPS EAG-TAGTPP --DFF----- -------SAA LRPG--GEAL KGVPGHCYSS LPRSYKAPCT CGGGDN---- DQYVLMSSP- ---------- -----VGRIL EEERLEPQAT PG----AGTF GA---AGGSH TQPHHSAVPS SMRPSGIV-G RPEGFLGQRC RAVRPTRLSL
Mes_au2     ----SKLSME N-ADPKLLP- NGDYLNMSPS EAG-AAGTPP --DFF----- -------SAA LRGS--GEGL KGVPGHCYSS LPRSYKAPY- C-SGDS---- DQYVLMSSP- ---------- -----VGRIL EEERLEPQAT PGTSQLTGTF GA---AGVGH TQPHHSAVPS PMRPSGSSVG RPEGFLGQRC RAVRPTRLSL
Mic_oc2     ----SKLSME N-TDPKLLP- NGDYLNMSPS EAG-TAGTPP --DFF----- -------SAA LRGG--GEGL KGVPGHCYSS LPRSYKAPY- C-SGDS---- DQYVLMSSP- ---------- -----VGRIL EEERLEPQAT SGTSQLAGTF GA---AGGGH TQPHHSAVPS SMRPSGGS-G RPEGFLGQRC RAVRPTRLSL
Per_ma2     ----SKLSME N-ADPKLLP- NGDYLNMSPS EAGAAAGTPP --DFF----- -------SAA LRGG--GEGL KGVPGHCYSS LPRSYKAPYS C-SGDS---- DQYVLMSSP- ---------- -----VGRIL EEERLEPQAT PGTSQLAGAF GA---AGGGH TQPHHSAVPS PMRPSGSG-G RPEGFLGQRC RAVRPTRLSL
Pro_co2     ----SKLSME N-ADSKLLP- NGDYLNMSPS DAG-TSGTPP --DFF----- -------SAA LHAG--GEML KGVPGYCYSS LPRSYKAPYT C-GGGN---- DQYVLMSAP- ---------- -----VGRIL EEERLEPQAT PGSTPSASAF GA---SGGGH TQPHHPVVPS PVRPSGS--G RPEGFLSQRC RAVRPTRLSL
Mic_mu2     ----SKLSME N-ADSKLLP- NGDYLNMSPS DVG-TSGTPP --DFF----- -------SAA LHAG--GEML KGVPGYCYSS LPRSYKAPYT C-GGGN---- DQYVLMSAP- ---------- -----VGRIL EEERLEPQAT LGTTPSASAF GV---GGGGH TQPHHP-VPS PVRPSGS--G RPEGFLSQRC RAVRPTRLSL
Ovi_arm2    ----SKLSME S-ADGKLLP- NGDYLNMSPS DAG-TTGTPP --DFF----- -------SAA ------GETL RGMPGYCYSS LPRSYKAPHA C-HGDS---- DQYVLMSSP- ---------- -----VGRVL EEEQLE--PA PGPAQPASAF LA-AAAGSGH PQPPHPAVPS PGRLGGGGGS RPDGFLAQRC RAVRPTRLSL
Orc_or2     ----SKLSVE SAADGRLLP- NGDYLNMSPG DAG-ASGTPP --DFF----- -------SAA LHAGGGGEML RGVPGYCYSS LPRSYKAAYT C-NGDN---- DQYVLMSSP- ---------- -----VGRIL EEERLESTAG PGSTQPAGAF AAGAGGGGGH PQPPHPAVPS PGRPGGSGSG RLEGFLGQRC RATRPTRLSL
Xen_tr2     ----PK---- ----SRTVP- V--HQRLSPA ESL-TSFTPP --DYFYMQSK NSCCETNSVA ---------- ------CRSI SP-------- ---AEV---- EPYVMMRSP- ---------- -----ASS-- ---------- ---------- ---------- ---------- ---------- ------SQHI RIARPSRLA-
                                                                                                                   $
Mus_mu3     ---------- ---------- STEYVPM--- ----NRFLPG ---------- ---------- ---------- ---------- -PFYYELKAR ESE------- ---------- ---------- -----LGHPG AHCSLRDRWR PTVAQP---- ---------- ---------- ---------- ---------- RSSQGSELS-
Rat_no3     ---------- ---------- STEYMPM--- ----NRFLPG ---------- ---------- ---------- ---------- -PLYYEFKAR EPE------- ---------- ---------- -----HGHSS AQCSIRDRWR PMVAQP---- ---------- ---------- ---------- ---------- RSSQGSELS-
Mes_au3     ---------- ---------- NTEYVPM--- ----NRFLPG ---------- ---------- ---------- ---------- -PFYYELKAR GPE------- ---------- ---------- -----LGHQG ALCSVGDRWG PTEAQP---- ---------- ---------- ---------- ---------- RSSQLSELS-
Mic_oc3     ---------- ---------- STEYMPM--- ----NRLSPG ---------- ---------- ---------- ---------- -PFYYEFKAK EPE------- ---------- ---------- -----LGHRG TLCSVRDRWR PTEAPT---- ---------- ---------- ---------- ---------- CSSQLSEVS-
Per_ma3     ---------- ---------- GTEYMPM--- ----NRFLPG ---------- ---------- ---------- ---------- -PLYYELKAR DPE------- ---------- ---------- -----PGHQG FPRSIRDRWG PTEAQA---- ---------- ---------- ---------- ---------- HSSQRSELS-
Pro_co3     ---------- ---------- DTEYVPM--- ----NRFLPG ---------- ---------- ---------- ----SFSLSS LPHSYKSGAG KPG------- ---------- ---------- -----PGLPG AHRSVGGSWG LAGVQP---- ---------- ---------- ---------- ---------- CSLPPSELA-
Mic_mu3     ---------- ---------- DTEYVPM--- ----NRFPPG ---------- ---------- ---------- ----SFSLSS LHHSYKSGAG KPG------- ---------- ---------- -----PGLRS AHRSAGGSWG LPRAQP---- ---------- ---------- ---------- ---------- CSLSPSELA-
Ovi_arm3    ---------- ---------- STEYVTM--- ----SCCAPR ---------- ---------- ---------- ----SF--PF LPLSF--AAG EPE------- ---------- ---------- -----LGLQG AHLGLRGGWG RAGAQP---- ---------- ---------- ---------- ---------- SLQPPSELA-
Orc_or3     ---------- ---------- VTEYVSM--- ----SLCAPR ---------- ---------- ---------- ----SFSSSF LPLSYRPGAG EPG------- ---------- ---------- -----PGLQG PHLGVGDDWG SAGAQR---- ---------- ---------- ---------- ---------- CLQPPSELA-
Xen_tr3     ---------- ---------H ASDYMNMWSL SRS-ASSTPP PQEAF----- ---------- ---LSSPGGP CRVPA-SYRS LPRSYK---- ---------- ----MEPQP- ---------- -----SAR-- -------ASC SSSSDSLEEV NAG------- ---------- ---------- ---------- KNRRPLSISI
                         $  $
Mus_mu4     ---------- ---------- DSDYMFMAPG AGA-IPKNPP ---------- ---------- ----NAQGGS SSKSWSSYFS LPSPFQSSPL GQSDH----- SEYVPMLPGK FLG------- -----SGLHK EASFSQGTKN VSSKPSTEAS FSKPEDKGSS AKPSDDVPPM N--------- ---------- KAKEPNHLSF
Rat_no4     ---------- ---------- DSDYMFMAPG AGA-IPKNPP ---------- ---------- ----NPQGGS SSKSWSSYFS LPSPFQSSPL GQSNH----- SEYVPMLPGK FLG------- -----SSLDK HASFSQGTKD VASKPSTEGS FSKPEDKGSS ARPSDDVPPM N--------- ---------- QAKEPNRLSF
Mes_au4     ---------- ---------- DSDYMFMAPG AGA-IPKHPP ---------- ---------- ----NPQGGS SSKSWSSYFS LPSPFQSSPL GQSDH----- SEYVPMLPGK FLG------- -----RGLDK EASFSQGNKD AASKPSTEGS FSKPGDSGSL AKLSDDEPPV N--------- ---------- KANRPNRLSF
Mic_oc4     ---------- ---------- DSDYMFMAPG AGA-IPKNPP ---------- ---------- ----NPQGGS SSKSWSSYFS LPSSFHSSPL GQSDH----- SEYVPMLPGK FLG------- -----RGLDK EPSFRPSTKD VASE--TEWS FSKPGDRGSP AKPSDDEFPV N--------- ---------- KAEGLNRLSF
Per_ma4     ---------- ---------- DSDYMFMAPG AGA-IPKNPP ---------- ---------- ----NPQGGS SSKSWSSYFS LPSPFQSSPL GQSDH----- SEYVPMLPGK FLG------- -----RGLDK EASFSQGAKD VASKPSTEGS FSKPGDRGSP AKPSDDEPPE N--------- ---------- KAKRPNRLSF
Pro_co4     ---------- ---------- DSDYMFMAPG AGA-IPKNPK ---------- ---------- ----NAQGGS SSKSWSSYFS LPNPFRSSPL GQSDH----- SEYVPMLPGK FLG------- -----RGLEK EVSSNWGPKH AASKLSVEGL FSKPGDRGAP SKPLDDGSPK N--------- ---------- KAKRANRLSF
Mic_mu4     ---------- ---------- DSDYMFMAPG AGA-IPKNPK ---------- ---------- ----NAEGGS SSKSWSSYFS LPNPFRSSPL GQSDH----- SEYVPMLPGK FLG------- -----RGLEK EVSSNWGTKH AASKPSVEGL FSKPADRGAP SKPLDDGPPK N--------- ---------- KAKRLNRLSF
Ovi_arm4    ---------- ---------- DSDYMFMAPG AGA-IPKNPR ---------- ---------- ----NPQGGS SSKSWSSYFS LPNPFRSSPL GQSDH----- SEYVPMLPGK FLG------- -----KGLEK EVLSNRGPKD TASKPSVEGS FSKPRDGGSP SKRSGDGPPK H--------- ---------- KAKRPNRLPF
Orc_or4     ---------- ---------- DSDYMFMAPG AGA-IPKNPR ---------- ---------- ----NPQGGS SSKSWSSYFS LPNAFRSSPL GQSDH----- SEYVPMLPGK FLG------- -----KGLDK EASSNRGPKD VTSKPSVEGS FSKPRDEGSP NKRSGDGPPK N--------- ---------- KTKRPNRLPF
Xen_tr4     ---------- ---------- SIKQINDSNV TNNNNSRGLS ---------- ---------- ---------- ------SYFS LPRSFKNLTK QNIDH----- NEYVPMSSPG KLLHLGAENG VGVCKEGAVN DIAKTELKSS GNILDQ---- ---------- ---------- ---------- ---------- QVKRPTKLTL
                         $$$$                                                    $$$$$$  $             $       $$
                         #  #                                                            #  #
```

```
Mus_mu1     -GDPKASTLP RVRE------ ---QQQQQQS SLHPPEPKSP ---------- GEYVNIEFGS GQPGYL---- ---------- ------AGPA TSRSSPSVRC P---PQLHPA PR-EETGSEE YMNMDLG--- ---------- ---------- -------PGR RATWQ-ESGG V--------- --ELGRIGPA PP--GSATVC
Rat_no1     -GDPKASTLP RVRE-----Q QQQQQQQQQS SLHPPEPKSP ---------- GEYVNIEFGS GQPGYL---- ---------- ------AGPA TSRSSPSVRC L---PQLHPA PR-EETGSEE YMNMDLG--- ---------- ---------- -------PGR RATWQ-ESGG V--------- --ELGRVGPA PP--GAASIC
Mes_au1     -GDPKASTLP RVRE------ ---QQQQQQP SLHPPEPKSP ---------- GEYVNIEFGS GQHGYL---- ---------- ------AGPA TSHSSPSVRC P---PQLHPA PR-EETGSEE YMNMDLG--- ---------- ---------- -------PGR RATWQ-ESGG A--------- --ELGRVGPA PP--GAATIG
Mic_oc1     -GDPKASTLP RVRE------ ----QPPQQP SLHPPEPKSP ---------- GEYVNIEFGS GQPGYL---- ---------- ------AGPT TSNSSPSVRC P---PQLHPA PR-EETGSEE YMNMDLG--- ---------- ---------- -------PGR RATWQ-ESGG I--------- --ELGRVGPA PP--GAANIC
Per_ma1     -GDPKASTLP RVREQQQQQQ QQQQQQQQQS SLHPPEPKSP ---------- GEYVNIEFGS GQPGYL---- ---------- ------AGSA TSHSSPSVRC P---PQLHPA PR-EETGSEE YMNMDLG--- ---------- ---------- -------PGR RATWQ-ESGG V--------- --ELGRVGPA PP--GAATIC
Pro_co1     -GDPKASTLP RARE-----Q -----QQ--P LLHPPEPKSP ---------- GEYVNIEFGS DQPGYL---- ---------- ------SGPM ASHSSPSVRC P---SQLQPT PREEETGTEE YMKMDLG--- ---------- ---------- -------PGR RAAWP-ESSR V--------- --EMGRVGPT PP--GATSVC
Mic_mu1     -GDPKASTLP RARE-----Q -----QQ--P LLHPPEPKSP ---------- GEYVNIEFGS DQPGYL---- ---------- ------SGPV ASHSSPSVRC P---SQLQPT PREEETGTEE YMKMDLG--- ---------- ---------- -------PGR RAAWQ-ESSG V--------- --EMGRVGPA PP--GATSVC
Ovi_arm1    -GDPKASTLP RARE-----Q -----QQPPP LLLPPEPKSP ---------- GEYVNIEFGS DQPGYL---- ---------- ------SGPV ASHSSPSIRC P---SQLQPA PREEETGAEE YMNMDLG--- ---------- ---------- -------PGR RATWQ-ESVG V--------- --QPGRVGPA PP--GAASVC
Orc_or1     -GDPKASTLP RARE-----Q PQPQPQPPPP LLPPPEPKSP ---------- GEYVNIEFGS DQPGYL---- ---------- ------SGPG ASHSSPSVRC P---SQLQPA PREEETGAEE YMNMDLG--- ---------- ---------- -------PGR RAAWR-ESAG V--------- --QPGSMGPA PP--GAASVC
Xen_tr1     ENSSKASTLP RARE------ ---------P AL-PPEPKSP ---------- GEYVNIEFND K---VF---- ---------- ------SGGL M----PSM-C PLPFVQSRVV PQRENL--SE YMNMDLG--- ---------- ---------- ---------- --VWRAKTSY A--------- --STSSSSYE PPYKPVSSVC
                                                   $  $              $ $                                                                           $

Mus_mu2     EGLQ---TLP SMQE------ ---------Y PL-PTEPKSP ---------- GEYINIDFGE AGTRLSPPAP PLLASAASSS SLLSA-SSPA SSLGSGTPGT SSDSRQRSPL --------SD YMNLDFS--- ---------- ---------- -------SPK SPKPSTRSGD TVGSMDGLLS PEASSPYPPL PP--------
Rat_no2     EGLQ---TLP SMQE------ ---------Y PL-PTEPKSP ---------- GEYINIDFGE GGTRLSPPAP PLLASAASSS SLLSA-SSPA SSLGSGTPGT SSDSRQRSPL --------SD YMNLDFS--- ---------- ---------- -------SPK SPKPSTRSGD TVGSIDGLLS PEASSPYPPL PP--------
Mes_au2     EGLQ---TLP SMQE------ ---------Y PL-PAEPKSP ---------- GEYINIDFGE AGTRLSPPAP PLLASAASSS SLLSA-SSPA SSLGSGTPGT SSDSRQRSPL --------SD YMNLDFS--- ---------- ---------- -------SPK SPKPGTHSGG AAGSMDGLLS PEASSPYPPL PP--------
Mic_oc2     EGLQ---TLP SMQE------ ---------Y PL-PSEPKSP ---------- GEYINIDFGE AGTRLSPPAP PLLASAASSS SLLSA-SSPA SSLGSGTPGT SSDSRQRSPL --------SD YMNLDFS--- ---------- ---------- -------SPK SPKPVAHSGD TVGSMDGLLS PEVSSPYPPL PP--------
Per_ma2     EGLQ---TLP SMQE------ ---------Y PL-PTEPKSP ---------- GEYINIDFGE AGTRLSPPAP PLLASAASSS SLLSA-SSPA SSLGSGTPGT SSDSRQRSPL --------SD YMNLDFS--- ---------- ---------- -------SPK SPKPGTHSGD TVGSMDGLLS PEVSSPYPPL PP--------
Pro_co2     EGLQ---TLP SMQE------ ---------Y PL-PPEPKSP ---------- GEYINIDFGE AGARLSPPAP PLLASAASSC SLLSA-GSPA SSLGSGTPGT SSDSRQRSPL --------SD YMNLDFS--- ---------- ---------- -------SPK SPKPGTQSAD PVGSLDALLS PEASSPYPPL PP--------
Mic_mu2     EGLQ---TLP SMHE------ ---------Y PL-PPEPKSP ---------- GEYINIDFGE AGARLSPPAP PLLASAASSC SLLSA-GSPA SSLGSGTPGT SSDSRQRSPL --------SD YMNLDFS--- ---------- ---------- -------SPK SPKPGTQSGD PVGSLDALLS PEASSPYPPL PP--------
Ovi_arm2    EGLP---ALP RMHE------ ---------Y PL-PPEPRSP ---------- GEYINIDFGE AGARLSPPAP PLLASAASSS SLLSA-SSPA SSLGSGTPGT SGDSRQRSPL --------SD YMNLDFS--- ---------- ---------- -------SPK SPQPGAQGRD PVGSLDALLS PEASV-YPPL PP--------
Orc_or2     EGLQ---TLP RMHE------ ---------Y PL-PPEPKSP ---------- GEYINIDFGE AGARLSPPAP PLLASAASSS SLLSA-GSPA SSLGSGTPGT SGDSRQRSPL --------SD YMNLDFS--- ---------- ---------- -------SPK SPQPGGQAGD PVGSLDALLS PEASA-YPPL PP--------
Xen_tr2     --LP---TLP SMSE------ ---------H ---PPESSSP --------PG GEYIHIYFGE TRPQESLSVP P--PTTATES VLLSAGGSPV SSSSSGS--- ---GPQRSPV --------SD YMNLDFC--- ---------- ---------- ---------- ---------- ---------- ---------- PP--------
                        $                                                                                                      $  $           $ $$$

Mus_mu3     ---------- ---------- ---------- ---------- ---------- GDYMSI---- ---------- ---------- ---------- ---------- ---------- --------PD YVGTD----- ---------- ---------- ---------- ---------- ---------- ---------- ----------
Rat_no3     ---------- ---------- ---------- ---------- ---------- GDYMYI---- ---------- ---------- ---------- ---------- ---------- --------PD Y---P----- ---------- ---------- ---------- ---------- ---------- ---------- ----------
Mes_au3     ---------- ---------- ---------- ---------- ---------- GDYMYI---- ---------- ---------- ---------- ---------- ---------- --------PD CVGMS----- ---------- ---------- ---------- ---------- ---------- ---------- ----------
Mic_oc3     ---------- ---------- ---------- ---------- ---------- GDYMYI---- ---------- ---------- ---------- ---------- ---------- --------PD CVGMS----- ---------- ---------- ---------- ---------- ---------- ---------- ----------
Per_ma3     ---------- ---------- ---------- ---------- ---------- GDYMYI---- ---------- ---------- ---------- ---------- ---------- --------PD YVGMN----- ---------- ---------- ---------- ---------- ---------- ---------- ----------
Pro_co3     ---------- ---------- ---------- ---------- ---------- GKYVYIEYAA ---------- ---------- ---------- ---------- ---------- --------PD CVGMA----- ---------- ---------- ---------- ---------- ---------- ---------- ----------
Mic_mu3     ---------- ---------- ---------- ---------- ---------- GKHVYTEYAA ---------- ---------- ---------- ---------- ---------- --------PN CVGIA----- ---------- ---------- ---------- ---------- ---------- ---------- ----------
Ovi_arm3    ---------- ---------- ---------- ---------- ---------- GEYVCIEYAA ---------- ---------- ---------- ---------- ---------- --------AD YIGMG----- ---------- ---------- ---------- ---------- ---------- ---------- ----------
Orc_or3     ---------- ---------- ---------- ---------- ---------- GEYVCIEYAA ---------- ---------- ---------- ---------- ---------- --------AD YIGMG----- ---------- ---------- ---------- ---------- ---------- ---------- ----------
Xen_tr3     DSWNTGTLSG NYRR------ ---------- ---PPSP--- ---------- GEYVSIHFRA PQKKTS---- ---------- ------GKEA IDAPRESVSM E---EQPSAM PN------GD YTEMDFV--- ---------- ---------- -------PHR TLQPHVKTNE ERGTNGEN-- --AFKPVGKL LPALEKTSSE

Mus_mu4     IAKGTQV--- ---------- ---------- ---KPKPLNP TQERREAAGS RDYINIDFIK RERLVL---- ---------- -------PSS AQGLPDMRGV VTDPAPTAF- --------SG YLNVEFGVPF PNPTIRLSDL LRVLPGANSI PLAGTRW--- PFPGSAIGSI V--EAGEYIE VIFNPAMTPA MS--------
Rat_no4     IAKGNKI--- ---------- ---------- ---KPRPLNP TQEQREADSS NDYINIDFVK REHLVL---- ---------- -------APS AQGLPEFWGV ITNARPSAF- --------SR YVKVEFGAPF PNPTVRLSEL LRVIPGANPI PLAGARWPFR PFPGNAVGSI L--EGGEYIE VIFNPALTPA VP--------
Mes_au4     IEKGNKI--- ---------- ---------- ---KPKPVKP TQEQRGADSS HDYINIDFIK REGPMP---- ---------- -------APT AQGLPYSWDL IADTRRTGF- --------PS YLNIEFGVPF PNPAIRLSDL LRVLPGANSI SLAGARWPFR PFPGSATGGI V--GEGEYIQ VIFNPAMRPI VP--------
Mic_oc4     IAEGNNI--- ---------- ---------- ---KAKPVNP TQEQRGADSS HDYVNIDFIK RELREP---- ---------- -------ALS AQGLPDSWGL IIGPRRTGI- --------SS YLNVEVGVPF PNSAIRLSDL LRVLPGASSI SLARTRWPFY PFPGNATGSI V--EEGEYIE LFFNPVMRPI VP--------
Per_ma4     IAKGNKI--- ---------- ---------- ---NPNPVEP TQEQRGADSS RDYINIDFIK RERQVP---- ---------- -------APP AQGLPDPWGL IADSRLSAF- --------SS YLNIEIGVPF SNPTIRLSDL LRVLPGANTI YLAGARWPFR LFPGNATGSI M--EEGEYIE VIFNPAIRPV VP--------
Pro_co4     ITKGNKI--- ---------- ---------- ---KPKPQKP THEQREADGS SDYVNIDFTK RDSNRP---- ---------- -------VPF IQGLPDSWGI IADPRQSAF- --------SN YVNIEFGVPF LNSANHLLYL LR---SVGPL FLYGARWPLR LIPYRATGSN ANEEAGNYIE VIFNPAMTPA VP--------
Mic_mu4     ITKGNKI--- ---------- ---------- ---KPKPQKP THEQREADGS SDYVNIDFTK RDSNRP---- ---------- -------VPF IQGLPDSWGI IVDPRQSAF- --------YN YVNVEFGVPF LNS-NDLLYL LR---GAGPL FLDGTRWPLR LVPLSATGSN ANEEEGDYIE VIFNPAMTPF VP--------
Ovi_arm4    MTKGNKV--- ---------- ---------- ---KPKPQKP TQEQREADSP RGYVNIDFPK RGYNMP---- ---------- -------DPC LQRLPGVWGI ITDPRQFAF- --------SN YVNIEFGVPF PNPAGNLSDL FRAIPGANPF FLDG------ -----ATGSS ANREEGDYIE VTFNPAMTPA VP--------
Orc_or4     ITKGNKI--- ---------- ---------- ---KPKPQKP THEQKEADST SGYIKIDFPK RGSNMP---- ---------- -------APC IQRLPGLWGI IADPRQFAF- --------SN YVNVEFGVPF PNPANDLSDL LRAIPGANPF SLDGARWPLP PLPPSATGNS ANEEEGDYIE VIFNPAMTPA VP--------
Xen_tr4     -GIRGSNTIP RMFD------ ---------H SA-PAEPTSP ---------- GEYINIDFSD KASSTP---- ---------- ------YSLS AEGSPSSLGS SCDHRQ-SPL --------SD YMSVDID--- ---------- ---------- ---------- --VQSPKATA ELSNSLTDIS SYACTVVSRM QP--------
                                                                          $                                                                                                                                                      $
                                                                     ####                                                                           #


Mus_mu1     RPTRSVPNSR ---------G DYMTMQIGCP RQSYVDTSP- ---VAPV--- ----SYADMR T---GIAAEK ASLPRPTGAA PPP-----SS T--ASSSVTP QGATAEQATH SSLLGGPQGP GGMSAFTRVN LSPNHNQSAK VIRA-DTQGC RRRHSSETFS ---APTRAGN TVPFGAGAAV GGS----GGG GGGGSEDVKR
Rat_no1     RPTRSVPNSR ---------G DYMTMQIGCP RQSYVDTSP- ---VAPV--- ----SYADMR T---GIAAEK VSLPRTTGAA PPP-----SS TASASASVTP QGA-AEQAAH SSLLGGPQGP GGMSAFTRVN LSPNHNQSAK VIRA-DTQGC RRRHSSETFS ---APTRAGN TVSFGAGAA- -------GGG SGGGSEDVKR
Mes_au1     RPTRAVPSSR ---------G DYMSMQIGCP RQSYVDTSP- ---VAPV--- ----SYADMR T---GIAAEK VSLPRTTGAA PSA-----SS TASDSPA-AP QG-AAEQAAH SSLLGGPQGP GGMSAFTRVN LSPNHNQSAK VIRA-DTQGC RRRHSSETFT ---APTPAGN TVSFGAGAAV GGS----GGG SSSSSEDVKR
Mic_oc1     RPTRSVPSSR ---------G DYMTMQIGCP RQSYVDTSP- ---VAPV--- ----SYADMR T---GLAAEK VSLPRAAGAA PSS-----SS TASASPA-AP QG-AAEQAAH SSLLGGPQGP GSVSAFTRVN LSPNHNQSAK VIRA-DTQGC RRRHSSETFS ---APTRAGH TVSFGAGAAG AGS----GGG -GGSSEDVKR
Per_ma1     RPTRSVPSSR ---------S DYMTMQIGCP RQSYVDTSS- ---VAPV--- ----NYADMR T---GIAAEK VSLPRTTGAA PSS-----SS TASASPA-AP QGAAAEQAAP SSLLGGPQGP GGMSAFTRVN LSPNHNQSAK VIRA-DTQGC RRRHSSETFS ---APTHAGN TVSLGAGAAV GGSGGGGGGG SSSSSEDVKR
Pro_co1     RPTRAVPSSR ---------G DYMTMQMGCP RQSYVDTSP- ---VAPV--- ----SYADMR T---GMAAQE VSLPRAATAA PSS-----SS AASASPA-AP QG-TAELAAR SSLLGGPQGP GGMSAFTRVN LSPNRNQSAK VIRA-DPQGC RRRHSSETFS STPSATRVGN TVPFGGGAAA GAS----SGG G-SSSEDVKR
Mic_mu1     RPTRAVPSSR ---------G DYMTMQMGCP RQSYVDTSP- ---VAPV--- ----SYADMR T---GIAARE VSLPRAATAA PSS-----SS AASASPA-AP QG-TAELAAQ ASLLGGPQGP GGMSAFTRVN LSPNRNQSAK VIRA-DPQGC RRRHSSETFC STPSATRAGN TVPLGGGAAV GAS----SGG GSSSSEDGKR
Ovi_arm1    RPTRAVPSSR ---------G DYMTMQMGGP RQSYVDTSP- ---VAPI--- ----SYADMR T---GV-VED ASLPGATADA PSS-----SS TASVSST-AP PG-TGELAAR SALLGGP--- ---SAFTRVN LSPNRNQSAK VIRA-DPQGC RRRHSSETFS STPSATRVGN TVPFGAGAAV AGS----GGG SSSSAEDVKR
Orc_or1     RPTRAVPSSR ---------G DYMTMQMGCP RQSYVDTSP- ---VAPI--- ----SYADMR TGGGGL-VEE ASLPGAAAAT PSS-----SS KASASPA-AP QG-AEELAAR SSLLGGPQGP GGSSAFTRVN LSPNRNQSAK VIRA-DPQGC RRRHSSETFS STPSATRAGN TVPFGAGAA- AGG----GGG GSSSTEDVKR
Xen_tr1     -PTETCSSSR PPIRGKTNSR DYMSMQLSAL CSDYSQVPPT RITAKPITLS SNKSNYAEMS S---GGVSDN IP------AI PQT-----SN S--------- ---SLSEASR SSLLGQGSGP ---SAFTRVS LSPNRNQSAK VIRAGDPQG- RRRHSSETFS STPTTARVTS ---------- ---------- GPVSGEDVKR
                                      $                                                                                                                      $  $$                  $

Mus_mu2     RPSTSPSSLQ QPL-PPAPGD LY---RLPPA SA-ATSQGP- TAGSSMSSEP GDNGDYTEMA F---GVAA-- -TPPQPIVAP PKP-----EG ARVASP---- ---TSGLKRL S-LMDQVSGV ---EAFLQVS QPPDPHRGAK VIRA-DPQGG RRRHSSETFS STTTVTPV-- ---------- ---------- SPSFAHNSKR
Rat_no2     RPSASPSSLQ QPL-PPAPGD LY---RLPPA TA-ATSQGP- TAGSSMSSEP GDNGDYTEMA F---GVAA-- -TPPQPIAAP PKP-----EG ARVTSP---- ---TSGLKRL S-LMDQVSGV ---EAFLQVS QPPDPHRGAK VIRA-DPQGG RRRHSSETFS STTTVTPV-- ---------- ---------- SPSFAHNSKR
Mes_au2     RPSAPPSSLQ QPL-PPAPGE LY---RLPPV SA-ATSQGP- TAGSSKSSEP GDNGDYTEMA F---GVAA-- -TPPQPIAAP PKP-----EG ARVTSP---- ---TSGLKRL S-LMDQVSGV ---EAFLQVS QPPDPHRGAK VIRA-DPQGG RRRHSSETFS STTTVTPV-- ---------- ---------- SPSFAHNSKR
Mic_oc2     RPSASPSSLQ QPL-PPAPGE LY---RLPPA SV-ATSQGP- TAGSSTSSEP GDNGDYTEMA F---GVAA-- -TPPQPIAAP PKP-----EG ARVTSP---- ---TSGLKRL S-LMDQVSGV ---EAFLQVS QPPDPHRGAK VIRA-DPQGG RRRHSSETFS STTTVTPV-- ---------- ---------- SPSFAHNSKR
Per_ma2     RPSASPSSLQ QPL-PPAPGE LY---RLPPA PA-ATSQGPA AAGPSSSSEP GDNGDYTEMA F---GVAA-- -TPPQPIAAP PKP-----EG ARVASP---- ---TSGLKRL S-LMDQVSGV ---EAFLQVS QPPDPHRGAK VIRA-DPQGG RRRHSSETFS STTTVTPV-- ---------- ---------- SPSFAHNSKR
Pro_co2     RPPASTASLQ QPPPPPPPGE LY---RLPPA SAVAATQGP- GAASSLSSET GDNGDYTEMA F---GVAA-- -TPPQPIAAP PKP-----EG ARVISP---- ---TSGVKRL S-LMDQVSGV ---EAFLQAS QPPDPHRGAK VIRA-DPQGG RRRHSSETFS STTTVTPV-- ---------- ---------- SPSFAHNPKR
Mic_mu2     RPPVSPASLQ QPAPPPPPGE LY---RLPPA SAVATTQGP- GAASSLSSEN GDNGDYTEMA F---GVAA-- -TPPQPIAAP PKP-----EG ARVTSP---- ---TSGVKRL S-LMDQVSGV ---EAFLQAS QPPDPHRGAK VIRA-DPQGG RRRHSSETFS STTTVTPV-- ---------- ---------- SPSFAHNPKR
Ovi_arm2    RPAAPSSALQ PPPPPPPPGE LY---RLPAA PA---SKGP- GAASS-SSDT GDNGDYTEMA F---GVAA-- -TPPQPIVAP PKP-----DG ARVSSP---- ---VSGLKRL S-LMDQVSGV ---EAFLQAG QPPDPHRGAK VIRA-DPQGG RRRHSSETFS STTTVTPV-- ---------- ---------- SPSFAHTPKR
Orc_or2     RPAAPSSALQ PAPPPPPPGE LY---RLPPA PP---SQGP- GAASSPSSGA GDSGDYTEMA F---GVAA-- -TPPQPIAAP PKP-----DG ARVSSP---- ---VSGLKRL S-LMDQLSGV ---EAFLQAS QPPDPHRGAK VIRA-DPQGG RRRHSSETFS STTTVTPV-- ---------- ---------- SPSFAHNPKR
Xen_tr2     SPKSKINS-- ---------- ---------- -----SLGP- ---------- --NSDYTEMS ---------- ---PRKISAC SMSDVSLHET SSLEGA---- ---GSGVQCL TLLMDKIPG- ----NFSLPS PPPDPNRGAK LIRA-DPQGG RRRHSSETFS STTQVTPV-- ---------- ---------- SPSFAHSPKR
                                                                        $  $                                                                                 $$$$          $$$   $$$$$$$ $        $                           $ $ $

Mus_mu3     ---------- ---------- ---------- ---------- ---------- ---------- ---------- -------SAR LGS------- ---------- ---------- ---------- ---------- ---------- ---------- ---------- ---------- ---------- ---------- ----------
Rat_no3     ---------- ---------- ---------- ---------- ---------- ---------- ---------- -------SAR LGS------- ---------- ---------- ---------- ---------- ---------- ---------- ---------- ---------- ---------- ---------- ----------
Mes_au3     ---------- ---------- ---------- ---------- ---------- ---------- ---------- -------SAE PGS------- ---------- ---------- ---------- ---------- ---------- ---------- ---------- ---------- ---------- ---------- ----------
Mic_oc3     ---------- ---------- ---------- ---------- ---------- ---------- ---------- -------GAK PGS------- ---------- ---------- ---------- ---------- ---------- ---------- ---------- ---------- ---------- ---------- ----------
Per_ma3     ---------- ---------- ---------- ---------- ---------- ---------- ---------- -------SVK PGS------- ---------- ---------- ---------- ---------- ---------- ---------- ---------- ---------- ---------- ---------- ----------
Pro_co3     ---------- ---------- ---------- ---------- ---------- ---------- ---------- -------TAR PDP------- ---------- ---------- ---------- ---------- ---------- ---------- ---------- ---------- ---------- ---------- ----------
Mic_mu3     ---------- ---------- ---------- ---------- ---------- ---------- ---------- -------SAG PEP------- ---------- ---------- ---------- ---------- ---------- ---------- ---------- ---------- ---------- ---------- ----------
Ovi_arm3    ---------- ---------- ---------- ---------- ---------- ---------- ---------- -------TAI PEP------- ---------- ---------- ---------- ---------- ---------- ---------- ---------- ---------- ---------- ---------- ----------
Orc_or3     ---------- ---------- ---------- ---------- ---------- ---------- ---------- -------TDI PEP------- ---------- ---------- ---------- ---------- ---------- ---------- ---------- ---------- ---------- ---------- ----------
Xen_tr3     KDSWAVDK-- ---------- ---------- -----ALWP- ---------- ------TTLR EATGIPIESD LGRPEKVLA- PKP------- ---------- ---SAEEPEL KSLVSAVTG- ---CSLNMGQ DPP------- -MRL-DPST- RKRHCSES-- ---------- ---------- ---------- ----------

Mus_mu4     ------FADS AICYDAQTGQ IYVVDPFSEC CMDVSLSPGR CSEPPPVAR- LRREEAQERR RPQSRSQSLF ASTRAAVSAF PTDSLDRDFP AASA------ ---VIAAPAE APLLAVSRAL AVVSALAAAP SIGDVFAGFR AAAGVDSASA RGFQPVA--- GAQAVREFQD LAAG------ ---------- ----------
Rat_no4     ------FADS AICYDTQTGQ IYVVDPFSEC CMDASVSPSR YSEPPPVA-- -RQEEVQERR RPQSGSQSLF ANTRAAVSAF PTDSLDRDFP AASA------ ---VIAAPAG APLLAVSRAL AVASALAAAP STGAVFVGFP VAAG-DSASA RWFQPVANAP GAQAEREVRD IAAG------ ---------- ----------
Mes_au4     ------FADS AIRYDVQTGR IYVVDPFSEC CMDISLSPGR CSEPPPVAR- LLQEEVQERR RPQSRSQSLF ANTRAAVSAF PTDSLDRDFP AASA------ ---AVVIPAE APLLAVSRAL AVVSALAAAP GFGAIFAGYQ TTAGFDSSST RWFQPVANAL GAQAVRGAQD IAAG------ ---------- ----------
Mic_oc4     ------FADR AIRYDALTGE MYVVDPFSEC CMDVSLFPGR YSHPPPVARL LLQEEVQERR RPQSRSQSLF ANIRAAVSAF PTDIFDRDFP SVSA------ ---RDADVTE EPLLAVSQAL AVVSALAAAP GFGVLFGGFQ STARFDSASS RWFQPVANAL GAQVVREVQD ITAG------ ---------- ----------
Per_ma4     ------FADT TIRYDAQTGQ ICVVDPFSEC CMNVSLSPGP FSERPPVAR- LLQEEVQERR RQQSRSQSLF ANTRAAVSAF PTDSLDRDFP AASA------ ---RVAVPAG APLLAVSRAL AVVSALAAAP GFGAFFAGFQ ATARFDSASV RWFQPVANVL GAQAVRGVQD IATG------ ---------- ----------
Pro_co4     ------FADS AIRYDAETGR IYVVDPFSEC CMDISLSPSR CSEPLPVAR- LLQEEEQERR RPQSRSQSFF AAARAAVSAF PTDSLEEELF ASSA------ ---SAAAFAA APTLAVGRVL AAASALAAAP GIGSAAAGWE AAAGFDSASA RWFQPVANAA DAGAVRGTQN SALV------ ---------- ----------
Mic_mu4     ------FADS AIRYDAETGR IYVVDPFSEC CMDISLSPSR CSEPPPVVR- LLQEEERERR RPQSRSQSFF AAARAAVSAF PTDSLEGELS ASPA------ ---SAAAFAA APTLAVGRVL AAASALAAAP SI--VAASWE AAAGLDSASV RWFQPVANAA DARAARGTQN SAFA------ ---------- ----------
Ovi_arm4    ------FADS AIRYDAETGR IYVVDPFSEC CMNISLSPSR CSERPPVAR- LLLREELERR RPQSRSQSFF AAARAAISAF PTDSLERDLS ASFA------ ---AAAAAAA VPTLALGRAL AVASALVAAP GIGAAAAGLE AAAGFDSASV RWFLPVADAA GA-AVRGAQD VAGG------ ---------- ----------
Orc_or4     ------FADS AIRYDAETGR IYVVDPFSEC CMDISLSPSR CSEPPPVAR- LLLEEELERR RPQSRPQSFF AAARAAVSAF PTDSLERDLS ASLA------ ---AAAATA- -PTLAVSRAL AAASTLVAAP GSGAAAAGLE AAAGSDSASV RRFQPAANAA GAEVVRAAQD VAGG------ ---------- ----------
Xen_tr4     ---------- --------NA DYAKLPCGTA CVSKTDNRI- ---------- ---DDYTTMT F------NMA MTPPRSFASE TE------NG TKVDSP---- ---SSIVNRL C--IGDMTSL N--SGFSLPN PLPEPIAGPK VIRA-DQQG- RRRHSSETFS SASTVTTS-- ---------- ---------- SSCFTESGKR
                                      $                                     $ $                   $
```

```
Mus_mu1     HSSASFENVW LRP------- ---------- -GDLGGVSKE SAPVC----- ---------- ---------- ---------- --GAAGGLEK SLNYIDLDL- --AKER-SQD CPSQQQSLPP PPPHQPLGSN EGNSPRRSS- ---------- -------EDL SNYASISFQK QPEDRQ---- ---------- -----
Rat_no1     HSSASFENVW LRP------- ---------- -GDLGGASKE SAPGY----- ---------- ---------- ---------- --GAAGGLEK SLNYIDLDLV KDVKQH-PQD CPSQQQSLPP PPPHQPLGSN EGSSPRRSS- ---------- -------EDL STYASINFQK QPEDRQ---- ---------- -----
Mes_au1     HSSASFENVW LRP------- ---------- -GDLGGASKE TAQVC----- ---------- ---------- ---------- --GAAGGLES SLNYIDLDLA KDVRQR-PQE CPSQQQPLPP PAPHQPPGSN EGSSPRRSS- ---------- -------EDL STYASISFQK PPEDHQ---- ---------- -----
Mic_oc1     HSSASFENVW PRP------- ---------- -GDLGGASKE TAQAR----- ---------- ---------- ---------- --GAAGGLEN TLNYIDLDLA KDVKQR-PQE CPSQQQPLPP PPSHQPSGSN EGSSPRRSS- ---------- -------EDL STYASISFQK QPEDRQ---- ---------- -----
Per_ma1     HSSASFENVW LRP------- ---------- -GDLAGASKE TAPVS----- ---------- ---------- ---------- --GAAGGLEK SLNYIDLDLA KDVRQRPPRE CPSQQQPLPP PPPHQPSGSR EGSSPRRSS- ---------- -------EDL STYASISFQK PPEDRQ---- ---------- -----
Pro_co1     HSSASFENVW LKP------- ---------- -GELGEAPKE SAQVC----- ---------- ---------- ---------- --GAAGGLEN GLNYIDLDLV KDFKQR-PQE RTPQPQPPPP TPPHQPLGSG ESSSTRRSS- ---------- -------EDL SAYASISFQK QPEDRQ---- ---------- -----
Mic_mu1     HSSASFENVW LKP------- ---------- -GELGEAPKE SAQVC----- ---------- ---------- ---------- --GAAGGLEN GLNYIDLDLV KDFKQR-SQE RPAQPQPPLP TPPRQPLGSG ESSSTRRSS- ---------- -------EDL SAYASISFQK QPEDRQ---- ---------- -----
Ovi_arm1    HSSASFENVW LRP------- ---------- -GELGGAPKE LAQLC----- ---------- ---------- ---------- --GAAGGLEN GLNYIDLDLV KDFKQR-PQE RPPQPQPPPL PAPHQPLGSS QSGPTSRSS- ---------- -------EDL SAYASITFQK QPEDLQ---- ---------- -----
Orc_or1     HSSASFENVW LRP------- ---------- -GELGGAPKE LAQVC----- ---------- ---------- ---------- --GAAGGLEN GLNYIDLDLV KDFNQS-PQE RPAQPQPSPP PPPHQPLGRS ESGSTSRSS- ---------- -------EDL SAYASISFQK PPEDLQ---- ---------- -----
Xen_tr1     HSSASFENVW LKP------- ---------- -GEI--ARRD SLQ------- ---------- ---------- ---------- ---PSDHTHN GLNYIDLDLA KDLSGL--DH CNSHQ----- ---------- --SGVSHPS- ---------- -------DDL STYASITFHK LEEHRNQAET EE-------- -----
            $        $

Mus_mu2     HNSASVENVS LRKSSE-GSS -----TL--- -GGGDEPPTS PGQAQPLVAV PPV-PQARPW NPGQP-GALI GCPGGSSSPM RRETSVGFQN GLNYIAIDVR GEQGSL--AQ ---SQ---PQ PGDKNSWSRT RSLGGLLGTV GGSGASGVCG GPGTGALPSA STYASIDFLS HH-LKEATVV KE-------- -----
Rat_no2     HNSASVENVS LRKSSE-GNS -----IL--- -GGSDEPSTS PGQAQPSAGV PPA-PQARPW NPGQP-GALI GCPGGSSSPM RRETSVGFQN GLNYIAIDVR GEQGSL--AQ ---SQPQHPQ PGDKNSWGRT RSLGGLLGTV GGSGTSGVCG GPGTGALPSA STYASIDFLS HH-LKEATVV KE-------- -----
Mes_au2     HSSASVENVS LRKSSE-GSG -----SL--- -GGNDEPPTS PGQAQPSLAV PPG-PQARPW NPGQP-GALI GCPGGSSSPM RRETSVGFQN GLNYIAIDVR DEQGSL--SQ ---TQQPHPQ PGDKSSWGRT RSLGGLLGTV ASTGSSGVCG GPGTGALSSA STYASIDFLS HH-LKEATVV KE-------- -----
Mic_oc2     HNSASVENVS LRKSSE-GSC -----IL--- -GGSDEPPTS PGQAQPSLAV PPA-PQARPW NPGQP-GALI GCPGGGSSPM RRETSVGFQN GLNYIAIDVR DEQGSL--SP ---SQPQHPQ PGDKSSWGRT RSLGGLLSTV GSTGTGGVCG GPGPGALPSA STYASIDFLS HH-LKEATVV KE-------- -----
Per_ma2     HNSASVENVS LRKSSEGGSG -----IL--- -GGSDEPPTS PGQAQPSPAV PPA-PQARPW NPGQP-GALI GCPGGSSSPM RRETSVGFQN GLNYIAIDVS DEPGSL--SP ---SQPQHPQ PGDKSSWGRT RSLGGLLSSV GSTGASGVCG GPGTGALPSA STYASIDFLS HH-LKEATVV KE-------- -----
Pro_co2     HNSASVENVS LRKSSEGSGG -----VLGGG PGGGEEAPMS PSKLQPSLPV PSLPPQARPR APAQP-GGLV GCPGGSGSPM RRETSAGFQN GLNYIAIDVR DEPG-L--SQ ---PQ---PQ PGEKSSWGRT RSLGGLISAV GG-GSSGVCG GPGPGALPSA NTYASIDFLS HH-LKEATIV KE-------- -----
Mic_mu2     HNSASVENVS LRKSSEGSGS -----VLSGG PGGGEEAPTS PRQLQPSLPV PALPPQARPR APAQP-GGLV GCPGGSGSPM RRETSAGFQN GLNYIAIDVR DEPG-L--TQ ---PQ---PQ PGDKSSWGRT RSLGGLISTV GG-GGSGGCG GPGPGALPSA STYASIDFLS HH-LKEATIV KE-------- -----
Ovi_arm2    HNSASVENVS LRKGSEGGGG GGSSGIL--- -GGCDEPPSS PRQLP----- PPTPQQARAW TPAQPGGGLV GCPGGTSSPM RRETSTGFQN GLNYIAIDVR DEPG-L--SP -PLQQHPHAQ TGDRSAWGRT RSLGGLISAV GAGSPAAVCG GPGPGALPAA NAYASIDFLT HH-LKEATVV KE-------- -----
Orc_or2     HNSASVENVS LRKSGEGGGG ----SVL--- -GGGDEPPTS PRQLP----- PPQHPQARPW TPSQP-GGLV GCPGGSGSPM RRETSAGFQN GLNYIAIDVR DEPG-L--SP SPQQPQQHPQ TGDKSAWGRT RSLGGLIGAV GAGSTGGVCV GPGPGALPSA STYASLDFLT HH-LKEATVV KE-------- -----
Xen_tr2     HSSASVENVS TRS------- ---------- -GETLEAPEC ---------- ---------- ---------- ------GSPM CRETSAGFQN GLNYIAME-- -----L--SP ---EKRNLLL LTQASSRGHL PALGLASLDI GS-------- ---------- --YSSIDFLG HNRLKGATTV RE-------- -----
            $                                                                                     $  $       $   $$

Mus_mu3     ---------- ---------- ---------- ---------- ---------- ---------- ---------- ---------- -------LDS CLNYVDLDLV PPLE-VPGAA PGK------- ---------- ---------- ---------- --------SP HSYASIKF-- ---------- ---------- -----
Rat_no3     ---------- ---------- ---------- ---------- ---------- ---------- ---------- ---------- -------LDS CLNYVDLDLV PPLE-VPGAA PGN------- ---------- ---------- ---------- --------SP HSYASIKF-- ---------- ---------- -----
Mes_au3     ---------- ---------- ---------- ---------- ---------- ---------- ---------- ---------- -------LDS CLNYVDLDLV PPLE-VPGAA SGD------- ---------- ---------- ---------- --------SP HSCGSVKF-- ---------- ---------- -----
Mic_oc3     ---------- ---------- ---------- ---------- ---------- ---------- ---------- ---------- -------LDN CLNYVDLDLV PPLE-VPGAA PGE------- ---------- ---------- ---------- --------SP HGYASIKF-- ---------- ---------- -----
Per_ma3     ---------- ---------- ---------- ---------- ---------- ---------- ---------- ---------- -------LDN CLNYVDLDLV PPLE-VPGAA PRE------- ---------- ---------- ---------- --------SP HSYASIKF-- ---------- ---------- -----
Pro_co3     ---------- ---------- ---------- ---------- ---------- ---------- ---------- ---------- -------PDG HLNYVDLDLV PPLEAARGDV PGVPK----- ---------- ---------- ---------- -------LLP HSYARIEF-- ---------- ---------- -----
Mic_mu3     ---------- ---------- ---------- ---------- ---------- ---------- ---------- ---------- -------PDG HLNYVDLDLV PPLE-ARGDV PGVRK----- ---------- ---------- ---------- -------LLP HSYARIEF-- ---------- ---------- -----
Ovi_arm3    ---------- ---------- ---------- ---------- ---------- ---------- ---------- ---------- -------PDH GLNYVDLDLV PSLE-VRGHA PGARS----- ---------- ---------- ---------- --------RP HSYARLEFQN LAEAGSSCSI TPDSQVRFGP PPCP-
Orc_or3     ---------- ---------- ---------- ---------- ---------- ---------- ---------- ---------- -------PDG GLNYVDLDLV PPLE-VQGDA PGARN----- ---------- ---------- ---------- --------RP RSYAHIEFQN LGEAPSSCSI APESQVSLGP TPCLQ
Xen_tr3     ---------- VRP------- ---------- ---------- ---------- ---------- ---------- ------PAPC NGTRAAALEP GLNYIDLDLS KDAAGGVPPP VLVGQGGAPP PKIGFLGGR- ---------- ---------- -------PNI NTYASMDFQM CGELRRGSKD GTEC------ -----
                                                                                                             $$ $

Mus_mu4     ---------- ---------- ---------- ---------- ---------- ---------- ---------- ------WNPG ALNHRA---R GED------- --------LA AGAAAPPPPP RQIWVLRPQE RADSED---D DD-------- -------DDD DIYVRMDFAR RDYRK----- ---------- -----
Rat_no4     ---------- ---------- ---------- ---------- ---------- ---------- ---------- ------WNPG TLNQAA---N GED------- --------LA AGAAAPPPPP RHHRVLRPPE RADSEDN-YD DE-------- -------DDD NIYVRMDFAR RDYKK----- ---------- -----
Mes_au4     ---------- ---------- ---------- ---------- ---------- ---------- ---------- ------WNPG ALNQAA---R GED------- --------LV AGAAAPPPPP RPRRVLRPPE RADSEDNDDD DD-------- -------DDD DIYVRMDFAR RDYKK----- ---------- -----
Mic_oc4     ---------- ---------- ---------- ---------- ---------- ---------- ---------- ------WNHG AGNQAA---G GED------- --------LE AGAAAPPSPP CQRRVLKAPE SEDSEGN--- DD-------- -------DDD DIYVRMDFAR RDYKK----- ---------- -----
Per_ma4     ---------- ---------- ---------- ---------- ---------- ---------- ---------- ------WNPG ALNQAA---R GED------- --------LA ARAAAPPPPP RQRRVLRPPE RADSEDN--- DD-------- -------EDN DIYVRMDFAR RDYKK----- ---------- -----
Pro_co4     ---------- ---------- ---------- ---------- ---------- ---------- ---------- ------SNLG APNPPADLVR GENGAGGALA AAAAAAAAAA AAAAAPPPPP RGPRVPRPPE RGDSD----- DD-------- -------DDD DTYVRMDFAR PDN-KFDSPK RE-------- -----
Mic_mu4     ---------- ---------- ---------- ---------- ---------- ---------- ---------- ------WNLG APNPPADLAR GENGAGG--- --------AA AAAAAPPPPP RGPRVPRPPE RGDSD----- DD-------- -------DDD DIYVRMDFAR PDN-KFDSPK RGW------- -----
Ovi_arm4    ---------- ---------- ---------- ---------- ---------- ---------- ---------- ------SNPR APNPSADLAG GENGAGG--- --------AA AAVAAPPRSP GRGRVPRPPE REDS------ ---------- -------DED DTYVRMDFAR RDNRKFDSPQ RE-------- -----
Orc_or4     ---------- ---------- ---------- ---------- ---------- ---------- ---------- ------SNPR AQNPSADLAG G-NRAGG--- --------AA AAAAAPPPSP GRGRAPRPPE QEDS------ ---------- -------DDD DTYVRMDFAR PDNEKFDSPQ RGW------- -----
Xen_tr4     HSSASFDNVW LKPDENSCEQ ---------- ---------- ---------- ---------- ---------- ------DNKM SRNCSTGFQN GLNYIALSMH DGVCEPASTA CHQHQNGSRN ---------- ---------- ---------- -------LES GGYVSIDFTR SDCLKCPSSR KD-------- -----
                                                                                                                                                                                              $  $$$ $
                                                                                                             #                                                                                #  ##
```

Figure S8 Fig. Alignment of Proteins sequences from vertebrate species that contain all 4 complete coding sequences. Irs protein sequences were aligned by MAFFT at the codon level. The alignment was translated to generate a protein alignment. >>> and <<< indicate the boundaries of the Pleckstrin Homology (PH) and PhosphoTyrosine Binding (PTD) Domains. The **$** below each set of othologs identify residues that have JS Scores in the top 10% (see Additional file 11: Table S3). The **#** below the complete alignment indicate residues that have JS scores in the top 10% for the complete alignment. Y and **pY** above the alignments indicate tyrosine and putative phosphor-tyrosine residues conserved among Irs protein sequences.
